# An 18-Porphyrin Nanoring at the Size Limit for Global Aromaticity

**DOI:** 10.1021/jacs.5c09149

**Published:** 2025-08-28

**Authors:** Jake M. Holmes, Henrik Gotfredsen, Lene Gödde, Janko Hergenhahn, Kavita Rani, Keigo E. Yamada, Jie-Ren Deng, Liv Warwick, Michael Clarke, Matthew Edmondson, James N. O’Shea, Alex Saywell, Harry L. Anderson

**Affiliations:** † Department of Chemistry, 6396 University of Oxford, Chemistry Research Laboratory, Oxford OX1 3TA, U.K.; ‡ School of Physics & Astronomy, 6123 University of Nottingham, Nottingham NG7 2RD, U.K.

## Abstract

What is the size limit for global aromaticity? How large can a macrocyclic π-system be and still exhibit an aromatic ring current around its circumference? We address this question by investigating a π-conjugated butadiyne-linked 18-porphyrin nanoring (diameter 8 nm). This nanoring was synthesized by two different strategies: classical template-directed synthesis, using a radial template with 18 pyridyl binding sites, and Vernier templating, using a small hexapyridyl template. Both strategies are effective when the porphyrins have octyloxy side chains, but classical templating is more effective than Vernier templating when the porphyrins have bulky trihexylsilyl substituents. The size and shape of the nanoring were confirmed by scanning tunneling microscopy. ^19^F NMR oxidation titrations on the nanoring bound to a fluorinated template revealed shoulder signals indicating weak global aromatic and antiaromatic ring currents in the 10+ and 12+ oxidation states, which have Hückel circuits of 242 and 240 π-electrons, respectively. These shoulder signals do not appear in control experiments with a split-ring complex consisting of the linear porphyrin 18-mer bound to the same fluorinated template, confirming that they arise from global ring currents. Nucleus-independent chemical shift calculations using the BLYP35 functional qualitatively predicted that the 18 porphyrin nanoring would exhibit global aromaticity in the 10+ and 14+ oxidation states and antiaromaticity in the 12+ and 16+ oxidation states, in keeping with the experimental results from NMR spectroscopy and with the Hückel rule. The experimental results show that the ring currents in this 18-porphyrin ring are weaker than those in the homologous 12-porphyrin ring by at least a factor of 2.

## Graphical abstract

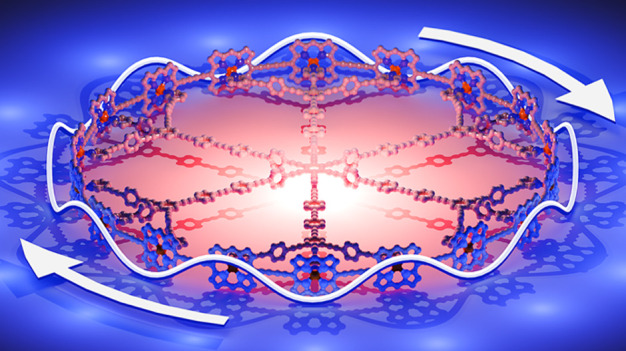

## Introduction

Aromaticity (or antiaromaticity) arises in π-conjugated macrocycles when the wave function of their delocalized electrons resembles, to some extent, that of a particle on a ring,^1^ resulting in increased (or decreased) thermodynamic stability and a diatropic (or paratropic) ring current.^2^ The majority of aromatic molecules have circuits of fewer than 24 π-electrons and diameters of less that 1 nm, suggesting that this phenomenon is mainly limited to small molecules, but a related effect is observed in mesoscopic rings, with diameters of up to 1000 nm,^3^ which makes it interesting to investigate evidence for global aromatic delocalization in large macrocycles.^4^ NMR spectroscopy has demonstrated the presence of global aromatic or antiaromatic ring currents in various large molecular rings.^5–12^ The largest macrocycle yet to display a global ring current is a butadiyne-linked 12-porphyrin nanoring ***c-*** **P12**_**THS**_, with a circuit of 162 π-electrons in its aromatic 6+ oxidation state (Figure 1, *N* = 12).^9^ Here we investigate a butadiyne-linked 18-porphyrin ring, ***c-*** **P18** (Figure 1, *N* = 18). We found that global ring current effects in ***c-*** **P18** are much weaker than in ***c-*** **P12**, but they are discernible in the ^19^F NMR spectra of the 10+ and 12+ oxidation states of the nanoring-template complex, with Hückel circuits of 242 and 240 π-electrons, respectively.

**Figure 1:**
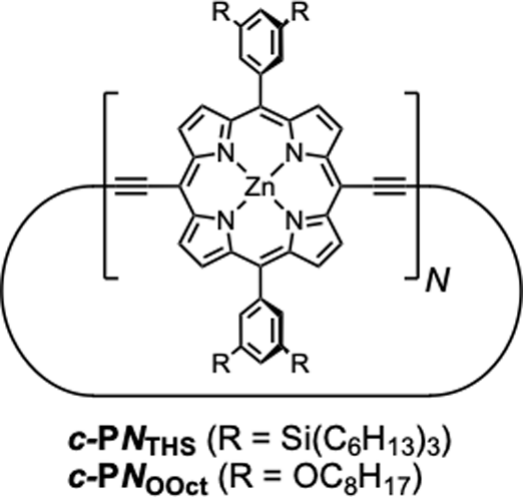
Structure of the ***c-*** **P** ***N*** nanorings with THS or OOct side chains. (*N* is the number of porphyrin units.).

One of the main challenges in investigating aromatic ring currents in ***c-*** **P18** is the synthesis of a suitable template to control the geometry of this nanoring. Aromatic ring currents in smaller nanorings (***c-*** **P** ***N***, where *N* = 5–8) can be investigated without requiring a template, because, when charged, these small nanorings are rigid enough to maintain cylindrical geometries, whereas larger nanorings are so flexible that internal and external nuclei cannot be distinguished by NMR spectroscopy.^11^ Binding a suitable template makes it possible to the probe magnetic shielding inside large nanorings and thus to test for global aromaticity. The roles of the template in these experiments are (1) to prevent rotation of individual porphyrin units, so that internal and external substituents can be distinguished, (2) to position probe nuclei inside the cavity of the nanoring (e.g., CF_3_ groups, for ^19^F NMR), and (3) to hold the macrocycle in a circular open conformation to maximize the cross-sectional area threaded by the magnetic flux. The same templates can also be used to direct the synthesis of nanorings. Here we compare two different templates for studying global aromaticity in ***c*** **-P18** (**T18**_**A**_ and **T18**_**B**_ in Figure 2). We also compare the efficacy of these templates for directing the synthesis of ***c*** **-P18** with trihexylsilyl (THS) or octyloxy (OOct) side chains.

**Figure 2:**
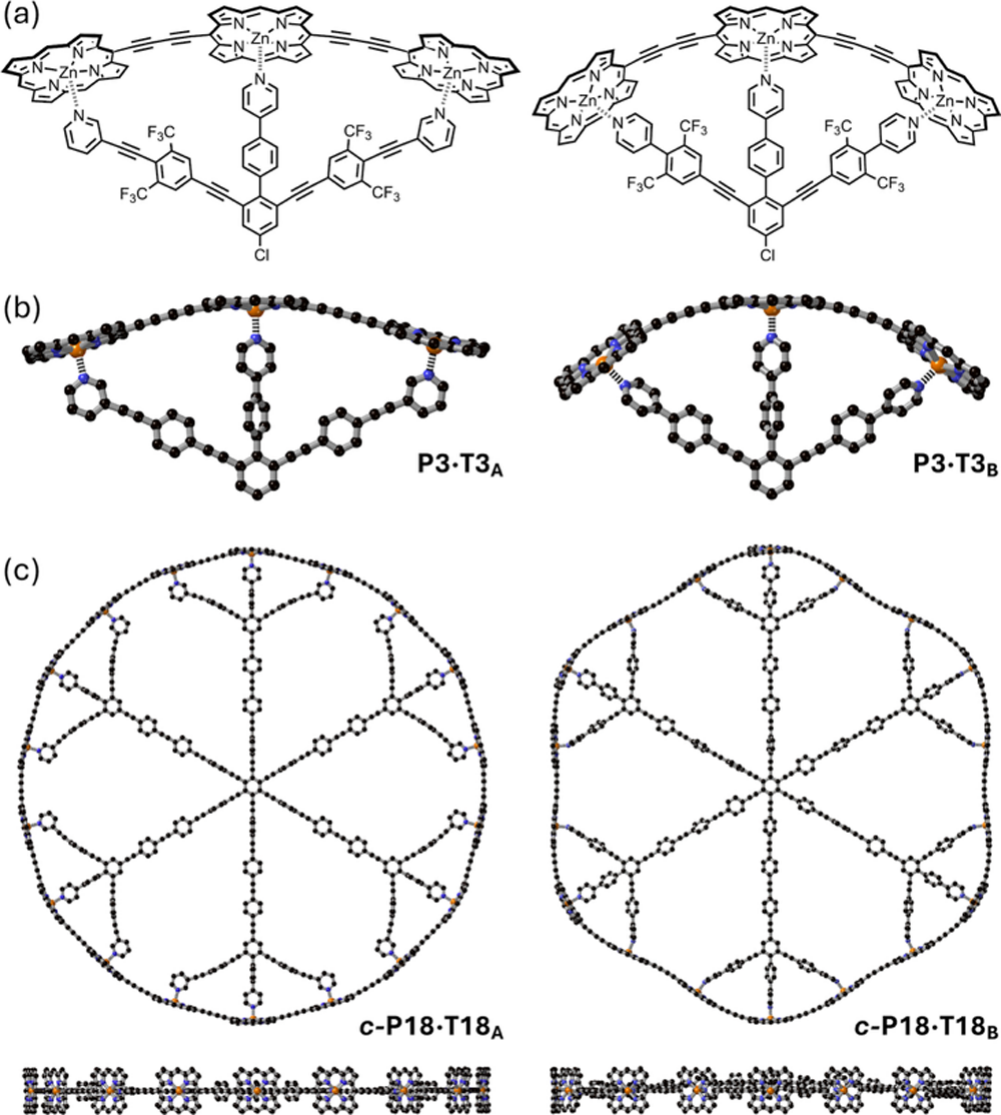
Chemical structures (a) and calculated geometries. (b) DFT (B3LYP/6–31g­(d)) optimized geometries of **P3·T3**_**A**_ and **P3·T3**_**B**_. (c) Semiempirical (PM7) optimized geometries of ***c-*** **P18·T18**_**A**_ and ***c-*** **P18·T18**_**B**_ (two orthogonal views).

## Results and Discussion

### Template Design and Molecular Modeling

We started designing a template for ***c-*** **P18** by considering possible ligands for holding a linear butadiyne-linked zinc porphyrin trimer **P3** in a curved geometry. The geometries of the complexes of **P3** with the two tridentate templates **T3**_**A**_ and **T3**_**B**_ were calculated using density functional theory (DFT; B3LYP/6–31g­(d)), showing that both templates pull **P3** into a curved shape as required in ***c-*** **P18** (Figure 2a,b). Other template designs were also explored computationally and found to give less good fits; see Supporting Information. Next, we expanded both **T3**_**A**_ and **T3**_**B**_ into their respective 18-legged templates (**T18**_**A**_ and **T18**_**B**_) and calculated the geometries of ***c-*** **P18** bound to each template. Due to the large size of these nanoring-template complexes, semiempirical (PM7) calculations were performed, rather than DFT. The in-plane views of the calculated structures of ***c-*** **P18·T18**_**A**_ and ***c-*** **P18·T18**_**B**_ (Figure 2c, bottom) show that the complexes are planar and that the templates fit well inside the cavity of the nanoring. It is noticeable that **T18**_**B**_ warps the geometry of ***c-*** **P18**, whereas **T18**_**A**_ holds it in a more circular shape (Figure 2c). The diagonal Zn···Zn distances in ***c-*** **P18·T18**_**A**_ and ***c-*** **P18·T18**_**B**_ are 7.8 and 8.0 nm, respectively. Templates **T18**_**A**_ and **T18**_**B**_ were designed to include CF_3_ substituents, so that global ring currents in ***c-*** **P18·T18**_**A**_ and ***c-*** **P18·T18**_**B**_ could be probed by ^19^F NMR spectroscopy (as demonstrated previously with related templates^9–12^ ).

### Molecular Dynamics Simulations

We evaluated the conformational flexibility of the proposed template complexes by performing molecular dynamics simulations on **P3** and ***c-*** **P18**, with and without bound templates: **T3**_**A**_ and **T3**_**B**_ for **P3**, and **T18**_**A**_ and **T18**_**B**_ for ***c-*** **P18**. These simulations used the General AMBER force field with modified parameters for zinc ions and porphyrin connections, as previously reported.^13,14^ All simulations were performed in explicit chloroform at 300 K (time step 2 fs; duration 200 ns for **P3** complexes and 50 ns for the ***c-*** **P18** complexes; see Supporting Information for details).

The torsional angle θ between adjacent porphyrin units (Figure 3a) is a key parameter controlling electronic delocalization, because it determines the orbital overlap.^15^ We extracted the probability density for values of θ ranging from 0° to 90° from molecular dynamics simulations, resulting in the data plotted in Figure 3b,c. An ideal template for promoting global aromatic ring currents would hold the nanoring in a geometry with θ = 0°. In the case of **P3**, in the absence of a template there is a slight preference for small dihedral angles (Figure 3b), but a wide range of angles is populated, as shown experimentally for the corresponding dimer.^15^ A surprisingly wide distribution of torsional angles is populated in **P3·T3**_**A**_, and this appears to be a consequence of the *meta*-pyridyl link in **T3**_**A**_, which allows concerted rotation of the bound **T3**_**A**_ ligand about the *C*_2_ symmetry axis of the complex. This rotation tilts the peripheral porphyrin units in opposite directions from the central porphyrin. The *para*-pyridyl side arms in **T3**_**B**_ result in a more rigid complex.

**Figure 3:**
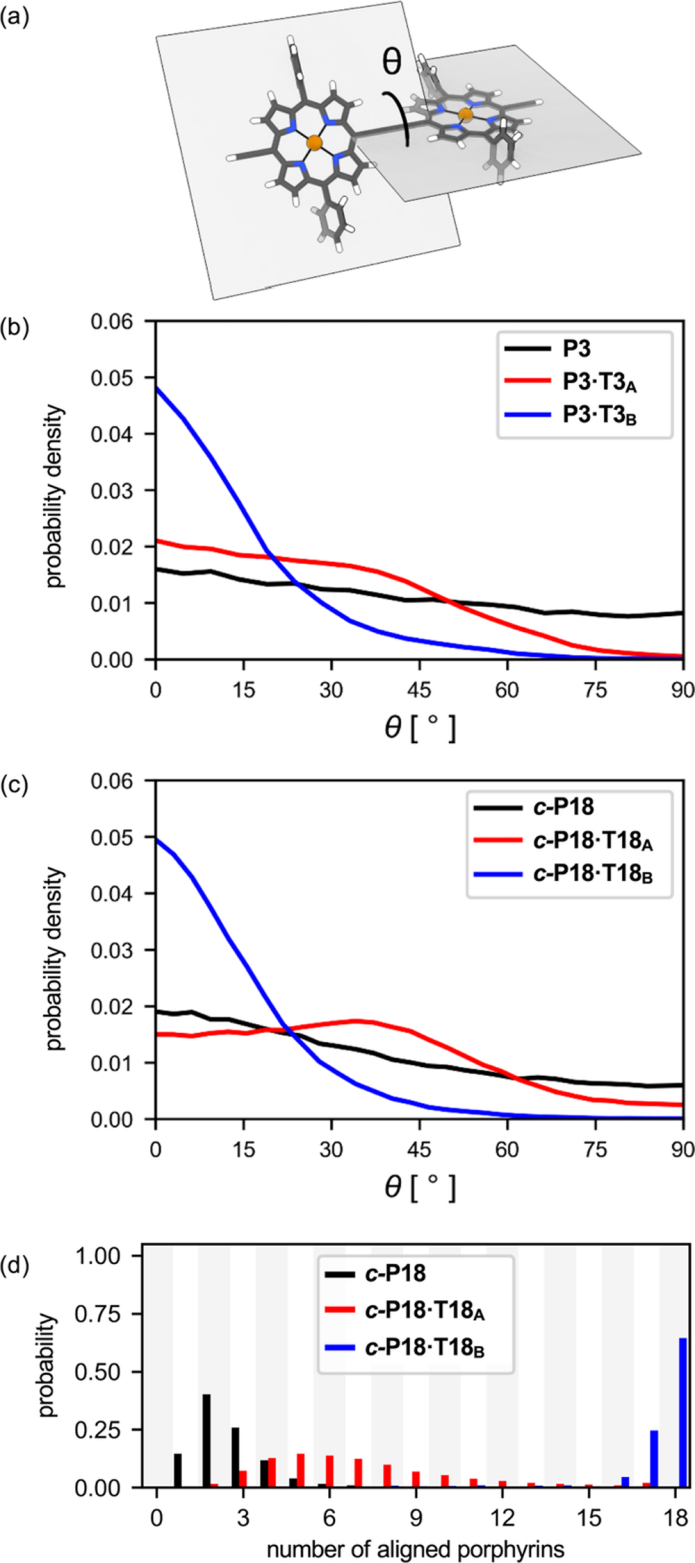
Results from molecular dynamics simulations. (a) Visual depiction of the torsional angle θ between adjacent porphyrin units. (b) Probability density plots for θ in **P3**, **P3·T3**_**A**_, and **P3·T3**_**B**_, and (c) in ***c*** **-P18**, ***c*** **-P18·T18**_**A**_, and ***c*** **-P18·T18**_**B**_ (bin size 4.5°). (d) Distributions of number of coplanar porphyrins for ***c*** **-P18**, ***c*** **-P18·T18**_**A**_, and ***c*** **-P18·T18**_**B**_ (coplanar defined as θ < 45° between adjacent porphyrin units).

Extending this analysis to ***c-*** **P18** reveals a similar trend, but the difference between **T18**_**A**_ and **T18**_**B**_ is more stark (Figure 3c). ***c-*** **P18·T18**_**A**_ shows a preference for θ = 30°–45°, with a lower probability for all torsional angles less than 15° than unbound ***c-*** **P18**. This indicates that binding **T18**_**A**_ disrupts the π-conjugation around the circumference of ***c-*** **P18**, whereas binding to **T18**_**B**_ enhances the π-conjugation.

The alignment of the porphyrin units was assessed by defining a cutoff of 45° in the dihedral angle between adjacent porphyrins, below which they are considered to be coplanar. The distributions in the number of coplanar porphyrins over the course of the simulations in ***c*** **-P18**, ***c*** **-P18·T18**_**A**_ and ***c*** **-P18·T18**_**B**_ are shown in Figure 3d. Binding to **T18**_**A**_ leads to an increased alignment of the porphyrins, but not nearly enough to organize the whole structure, as most conformations only have up to 10 porphyrins aligned. **T18**_**B**_ holds ***c*** **-P18** in a much more π-conjugated geometry, with all 18 porphyrins having θ < 45° with neighboring porphyrin units in most cases.

### Synthesis of Templates **T18_A_** and **T18_B_**

The templates **T3**_**A**_, **T3**_**B**_, **T18**_**A**_ and **T18**_**B**_ were synthesized as shown in Scheme 1. These routes exploit the different reactivity of chlorine, bromine and iodine substituents in intermediates **2** and **5**,^16^ and use triazenes as masked aryl iodides.^17^ The syntheses of **T18**_**A**_ and **T18**_**B**_ follow similar routes, with key intermediates in both pathways being **15**, **16** and either **T3**_**A**_ or **T3**_**B**_. Previously, we used **16** as the core for several other templates.^9,10,13,18^ Linker group **15** was designed to extend the core to the appropriate diameter, based on the **T18**_**A/B**_ models discussed above. *n-*Octyl chains are included in **15** to improve the solubility of the final templates. The aryl chloride substituents in **T3**_**A**_ and **T3**_**B**_ are unreactive toward Suzuki and Sonogashira coupling under most conditions, but they undergo efficient Sonogashira coupling catalyzed by an XPhos-palladium complex, in the absence of copper or amines.^19^ Use of a large excess of the terminal alkyne component resulted in high yields in the 6-fold Sonogashira couplings in the synthesis of **17**, **T18**_**A**_ and **T18**_**B**_. The all-*para*-pyridyl templates, **T3**_**B**_ and **T18**_**B**_, are noticeably more soluble than **T3**_**A**_ and **T18**_**A,**_ which facilitates their purification.

**Scheme 1:**
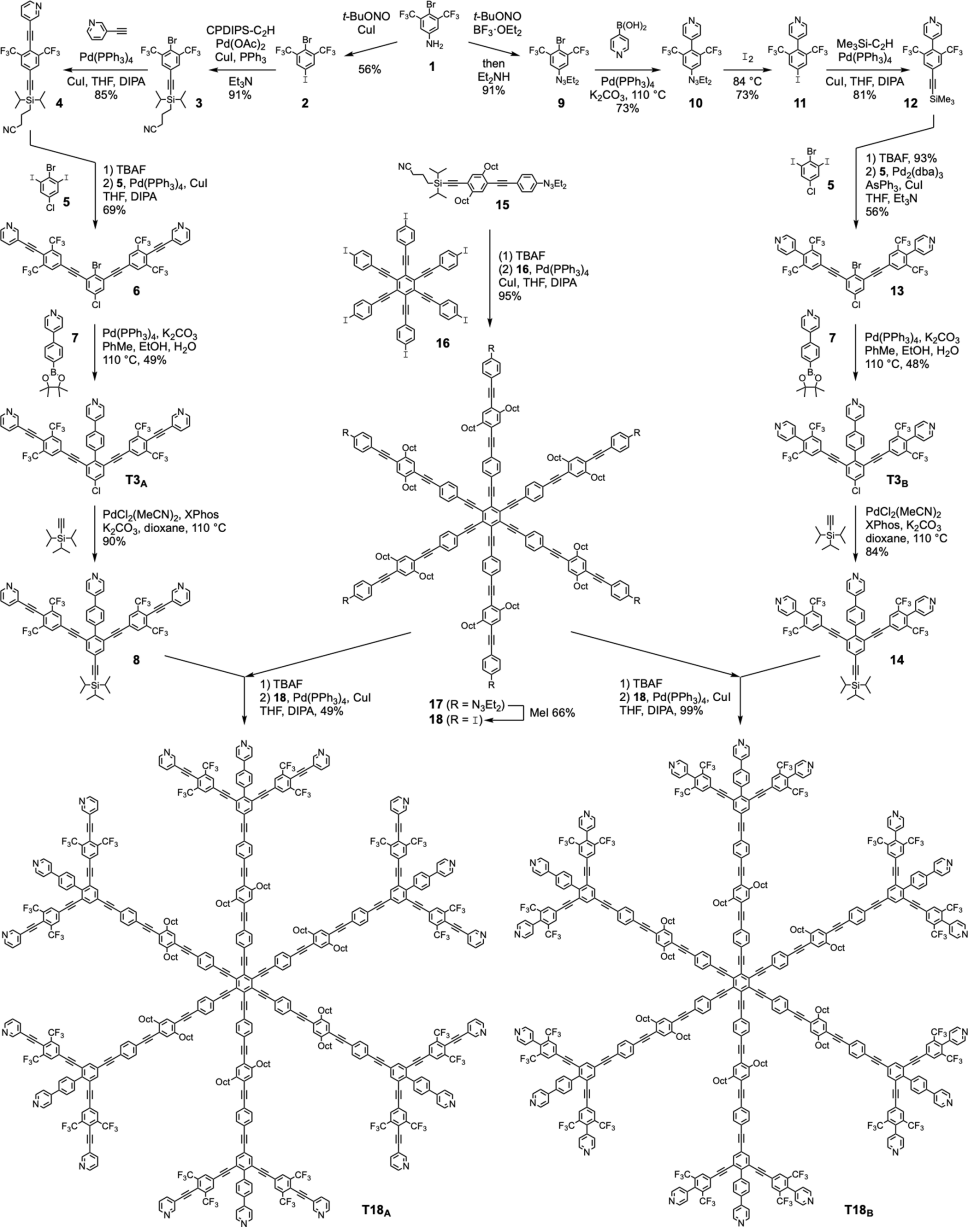
Synthesis of T3_A_, T3_B_, T18_A_, and T18_B_

### Template-Directed Synthesis of ***c*-P18**

Two versions of the nanoring, ***c-*** **P18**_**THS**_ and ***c-*** **P18**_**OOct**_, were synthesized from the linear nonamers, **P9**_**THS**_ and **P9**_**OOct**_, using template-directed palladium-catalyzed Glaser coupling. THS substituents confer high solubility, which is important for low-temperature NMR experiments. On the other hand, OOct substituents seem to be most suitable for scanning probe microscopy, as discussed below. We tested two routes to each of these nanorings: (a) classical template-directed synthesis using either **T18**_**A**_ or **T18**_**B**_ as the template, via formation of **(P9)**_**2**_ **·T18**, and (b) Vernier templated synthesis using **T6** as the template, via formation of **(P9)**_**2**_ **·(T6)**_**3**_ (Figure 4).^20,21^ The efficiency of these template-directed coupling reactions, with **P9**_**THS**/**OOct**_ as the starting material in every case, can be judged from the GPC traces of crude reaction mixtures (Figure 5). Coupling in the absence of a template gives only linear oligomers (Figure 5a,e). The Vernier template route gives poor selectivity with the bulky THS chains (Figure 5b; ***c-*** **P18**_**THS**_/**T6**), presumably because steric hindrance between THS groups destabilizes the **(P9**_**THS**_ **)**_**2**_ **·(T6)**_**3**_ intermediate, as found previously for similar complexes.^17,21^ The main product from coupling **P9**_**THS**_ in the presence of **T6** is ***c-*** **P9**_**THS**_, which is probably formed via the **P9**_**THS**_ **·(T6)**_**2**_ caterpillar-track complex.^22^ In contrast, Vernier templating is highly effective with octyloxy side chains (***c-*** **P18**_**OOct**_/**T6**), giving ***c-*** **P18**_**OOct**_ as the main product (Figure 5f). **T18**_**A**_ and **T18**_**B**_ provide the highest yields of ***c-*** **P18**_**THS**_ (Figure 5c,d), and there is no significant difference in the ability of these two **T18** templates to direct the formation of ***c-*** **P18**_**THS**/**OOct**_.

**Figure 4:**
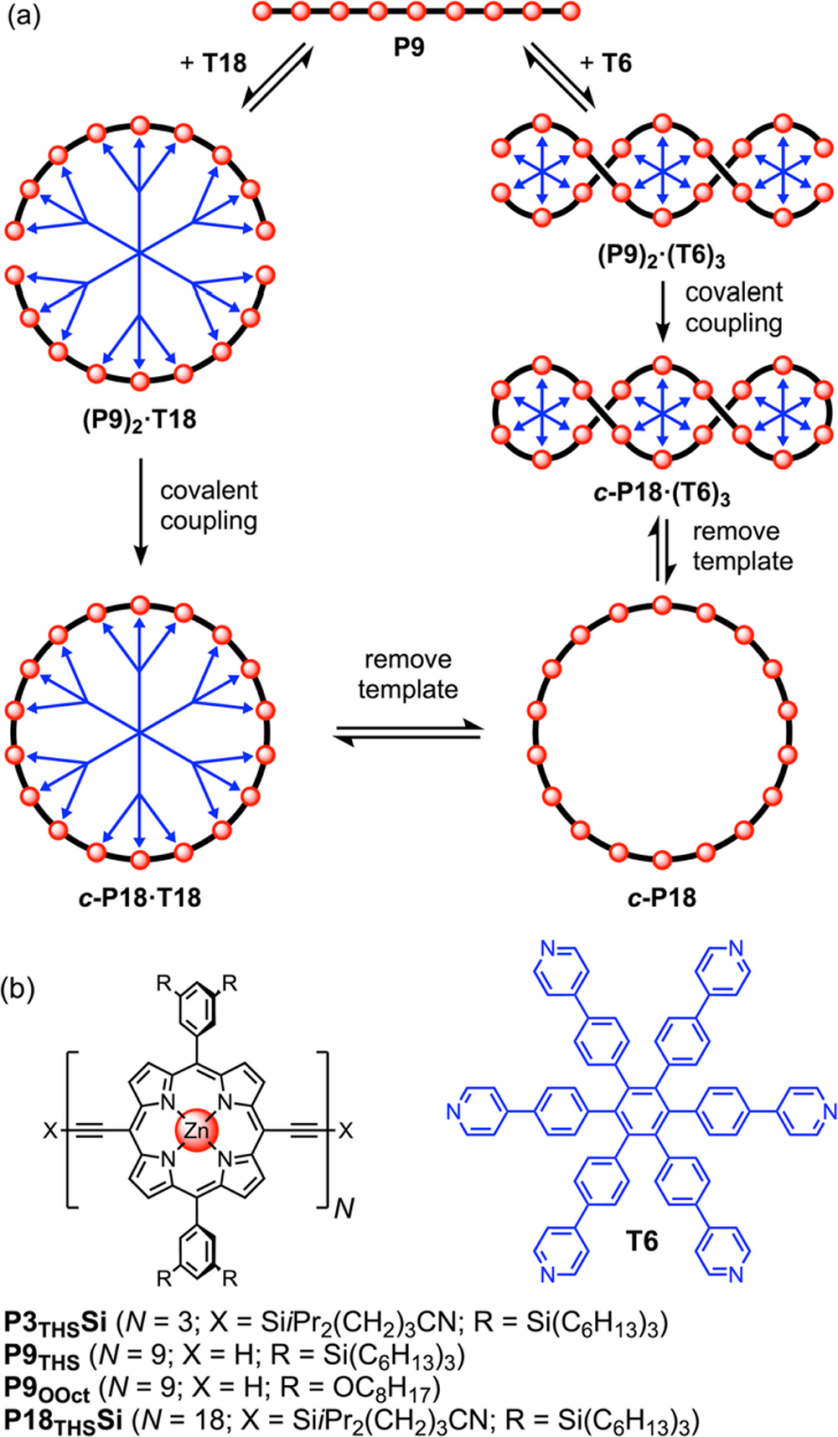
(a) Routes to ***c-*** **P18** by classical template-directed synthesis via **(P9)**_**2**_ **·T18** and by Vernier template-directed synthesis via **(P9)**_**2**_ **·(T6)**_**3**_; (b) chemical structures of **P3**_**THS**_ **Si**, **P9**_**THS**_, **P9**_**OOct**_, **P18**_**THS**_ **Si** and **T6**.

**Figure 5:**
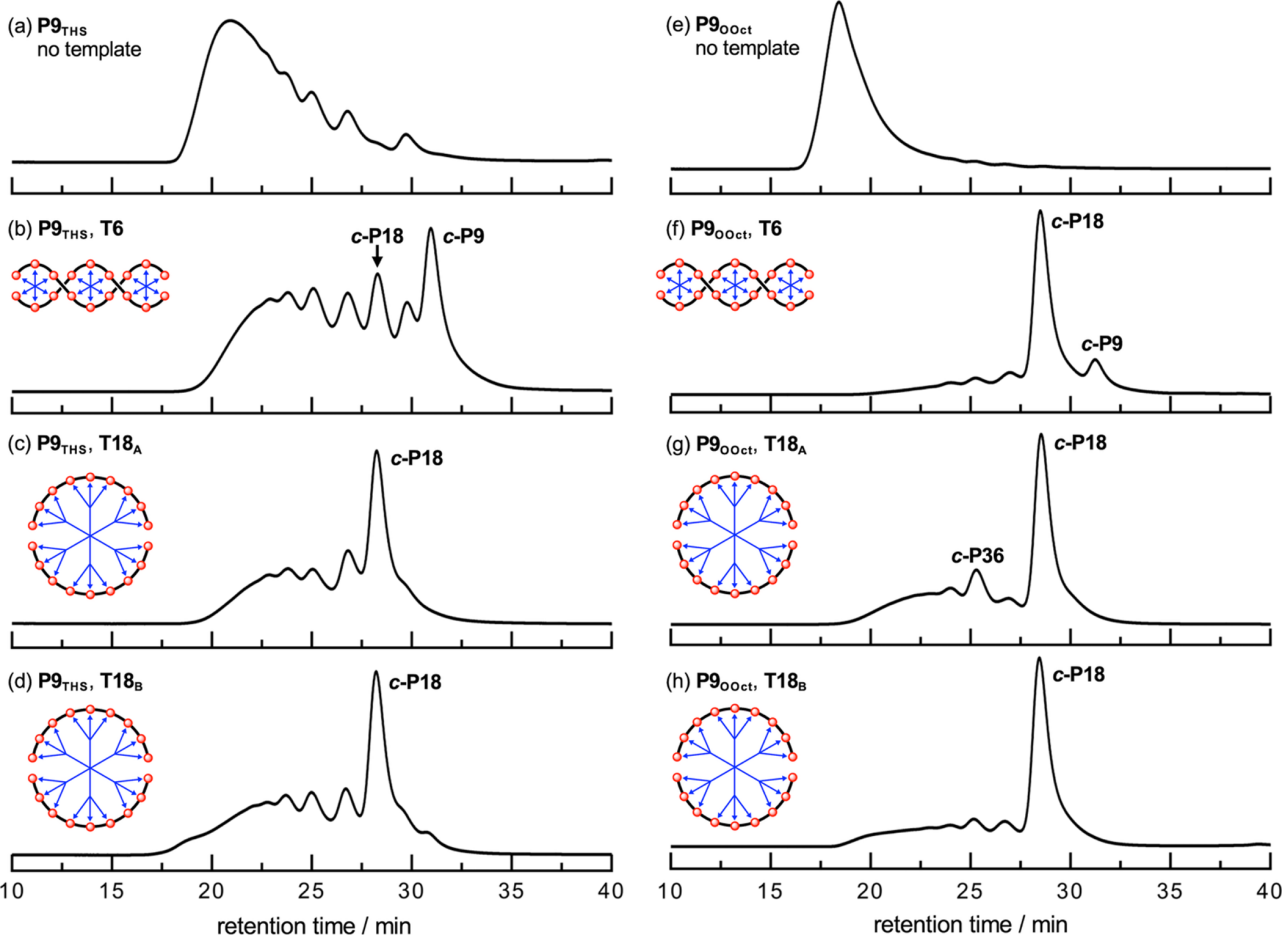
Gel permeation chromatography (GPC) traces (500 nm) of crude reaction mixtures from coupling **P9**_**THS**_ (a–d) and **P9**_**OOct**_ (e–h) in the absence of a template (a, e), in the presence of **T6** (b, f), **T18**_**A**_ (c, g) or **T18**_**B**_ (d, h). Reaction conditions: Pd­(PPh_3_)_2_Cl_2_, CuI, 1,4-benzoquinone in CHCl_3_ 20 °C, 17 h. Analytical GPC analysis performed using JAIGEL-3H-A (8 × 500 mm) and JAIGEL-4H-A (8 × 500 mm) columns in THF + 1% pyridine as eluent with a flow rate of 1.0 mL/min; detection: absorption at 500 nm.

### Imaging

Scanning tunnelling microscopy (STM) provided insights into the dimensions and morphology of the ***c*** **-P18**_**OOct**_ nanoring. These experiments used nanorings with octyloxy rather than THS side chains because octyloxy-substituted porphyrins have previously been found to be suitable for STM on gold surfaces, with the porphyrin lying flat on the surface.^23^ Nanorings were transferred from solution (toluene/methanol, 3:1) onto a Au(111) surface held under vacuum during in situ electrospray deposition.^13,24,25^ In line-with previous studies of homologous macrocycles (***c-*** **P12**_**OOct**_ and ***c-*** **P14**_**OOct**_)^26^ our STM images reveal the presence of single-height and stacked nanorings (Figure 6a). The lower contrast ring (white arrow) is assigned to a single-height species, while the brighter contrast ring (black arrow) is attributed to a stack of two nanorings. ***c*** **-P18**_**OOct**_ is observed to adopt a roughly circular geometry and in high resolution images the 18 porphyrin units within the nanoring can be clearly identified (Figure 6b). The long (*a*) and short (*b*) radii dimensions for ***c-*** **P18**_**OOct**_ were characterized (see SI for details) and the distributions of values of *a* and *b* are plotted in Figure 6c (*n* = 35:18 stacked rings, 17 single-height rings). The average dimensions for the stacked rings are found to be *a* = 3.6 ± 0.2 nm and *b* = 2.9 ± 0.3 nm, similar within error to the average dimensions for the single-height rings, *a* = 3.7 ± 0.3 nm and *b* = 2.7 ± 0.4. The average circumference is calculated to be 21 ± 1 nm (stacked) and 20 ± 1 nm (single-height). The distribution of the measured dimensions indicates a greater spread in flattening factor (*f*) for the single-height rings as compared to the stacked rings; in agreement with the previously reported enhanced mechanical stiffness for stacked rings.^26^

**Figure 6:**
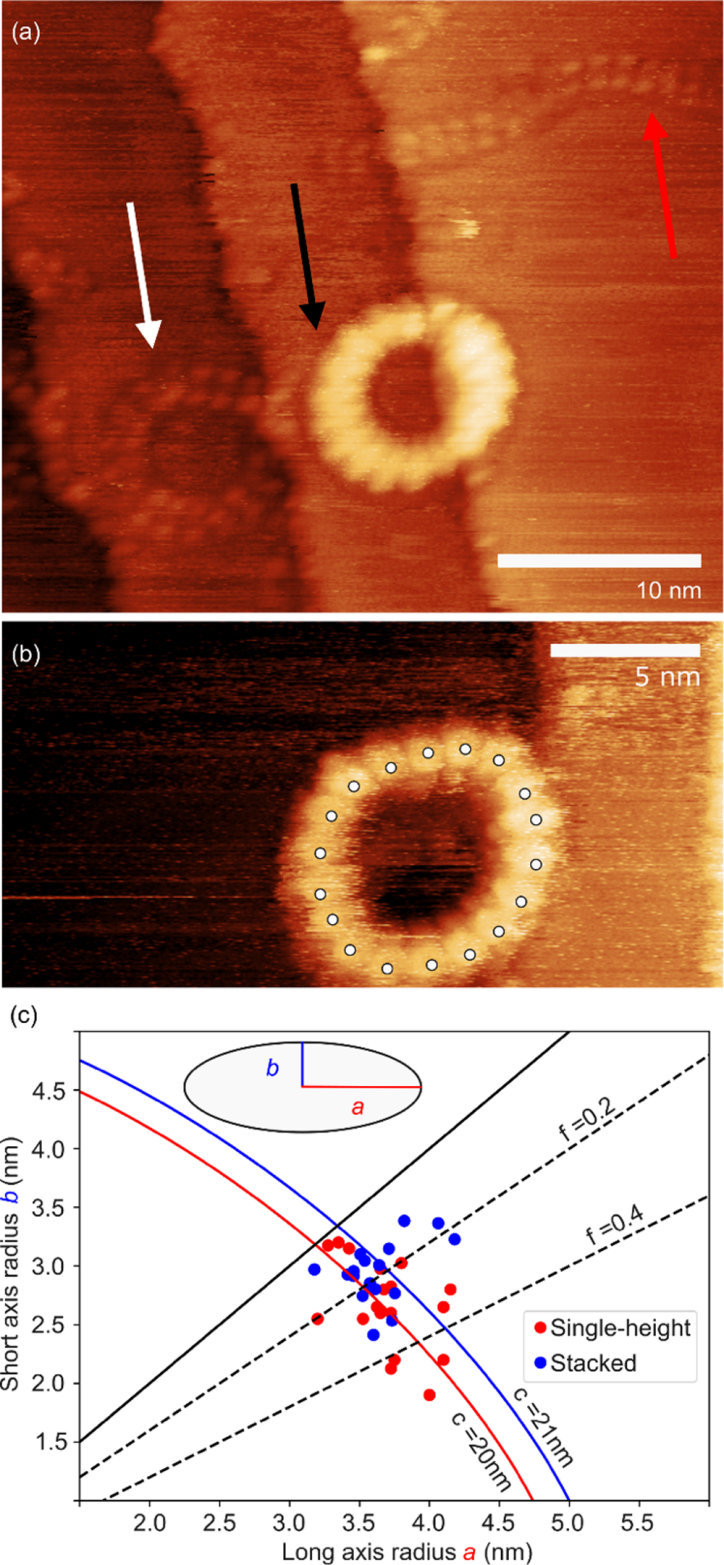
STM topographs of porphyrin nanoring ***c-*** **P18** deposited onto Au(111) via electrospray deposition. (a) High-resolution image of ***c-*** **P18** highlighting the difference in contrast between a vertical ‘stack’ of 2 nanorings (black arrow) and a single ring (white arrow); a linear section assigned to a broken ring is also shown (red arrow). (b) High-resolution image of ***c*** **-P18** where the individual porphyrin units within the ring can be identified (white dots indicate porphyrin units). (c) Plot of experimentally measured long (*a*) versus short (*b*) radii of ***c*** **-P18** (*n* = 35:18 stacked rings, 17 single-height rings). *f* = 1 −*b*/*a* indicates the ellipticity or flattening factor of the nanorings. The blue and red arcs correspond to ellipses of fixed circumference calculated using Ramanujan’s approximations – the values are equivalent to the average circumference. The solid black line indicates circular geometries (*a* = *b*, *f* = 0). The dotted lines represent *f* = 0.2 and 0.4, the shape becoming more elliptical with increasing *f* value. Imaging parameters: sample bias = −2 V, set-point current = 18 pA.

### UV–vis-NIR Spectra of Template Complexes

Mixing the zinc porphyrin trimer **P3**_**THS**_ **Si** (capped at the terminal *meso* positions with CPDIPS-acetylene, Figure 4b) with ligands **T3**_**A**_ or **T3**_**B**_ in chloroform results in immediate formation of the 1:1 complex **P3**_**THS**_ **Si·T3**_**A**_ or **P3**_**THS**_ **Si·T3**_**B**_. Similarly, ***c*** **-P18**_**THS**_ binds **T18**_**A**_ or **T18**_**B**_ to form ***c*** **-P18**_**THS**_ **·T18**_**A**_ or ***c*** **-P18**_**THS**_ **·T18**_**B**_. The UV–vis-NIR spectra of all six complexes are compared in Figure 7. Complexation with either **T3** template shifts the absorption spectrum of **P3**_**THS**_ **Si** to longer wavelengths, as expected,^27^ but there is a surprising difference between the spectra of **P3**_**THS**_ **Si·T3**_**A**_ and **P3**_**THS**_ **Si·T3**_**B**_ (Figure 7a): the bathochromic shift in the Q-band is much greater in the **T3**_**B**_ complex. Similarly, the Q-band of ***c*** **-P18**_**THS**_ **·T18**_**B**_ is red-shifted compared with that of ***c*** **-P18**_**THS**_ **·T18**_**A**_ (Figure 7b). The difference in absorption spectra confirms the conclusion from the molecular dynamics simulations discussed above: the average torsional angle between neighboring porphyrin units is substantially smaller in complexes of **T3**_**B**_ and **T18**_**B**_, compared with **T3**_**A**_ and **T18**_**A**_ (Figure 3), resulting in stronger orbital overlap and more red-shifted absorption spectra. These results indicate that the ***c*** **-P18**_**THS**_ **·T18**_**B**_ complex is more likely to exhibit global ring currents than ***c*** **-P18**_**THS**_ **·T18**_**A**_.

**Figure 7:**
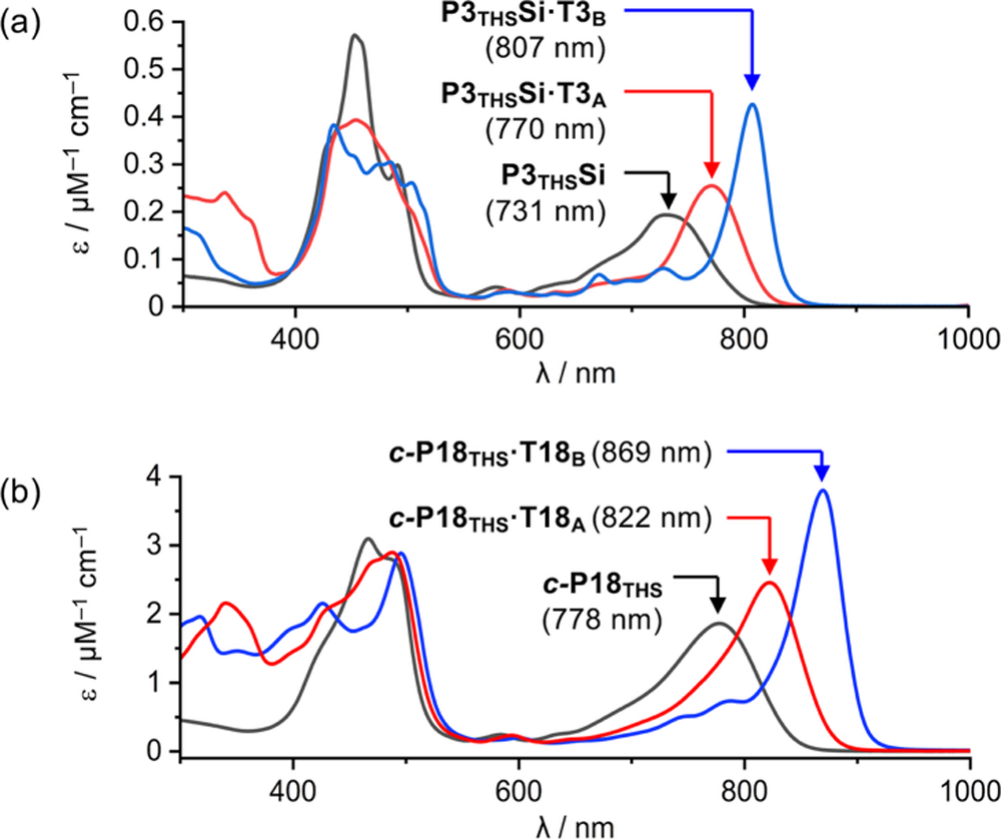
UV–vis-NIR absorption spectra of (a) **P3**_**THS**_ **Si** (black), **P3**_**THS**_ **Si·T3**_**A**_ (red) and **P3**_**THS**_ **Si·T3**_**B**_ (blue), and (b) ***c*** **-P18**_**THS**_ (black), ***c*** **-P18**_**THS**_ **·T18**_**A**_ (red) and ***c*** **-P18**_**THS**_ **·T18**_**B**_ (blue) recorded in CHCl_3_ at 298 K.

### Binding Constants

The formation of the complexes **P3**_**THS**_ **Si·T3**_**A**_, **P3**_**THS**_ **Si·T3**_**B**_, ***c*** **-P18**_**THS**_ **·T18**_**A**_ and ***c*** **-P18**_**THS**_ **·T18**_**B**_ were studied by titrating each template into a solution of the porphyrin host in chloroform at 298 K and monitoring the changes in the UV–vis-NIR spectra. All these titrations confirm the formation of a stable 1:1 complex (*K* > 10^7^ M^–1^; see Supporting Information).

Both the A and B versions of the templates bind strongly to their respective porphyrin partners, with surprisingly similar binding constants. We exploited the large difference in absorption spectra between **P3**_**THS**_ **Si·T3**_**A**_ and **P3**_**THS**_ **Si·T3**_**B**_ to determine the relative affinities of **T3**_**A**_ and **T3**_**B**_ for **P3**_**THS**_ **Si** by carrying out a competition experiment in which the ratio of the two complexes was monitored by UV–vis-NIR spectroscopy while changing the mole ratio of the two **T3** ligands. This experiment gave *K*(**T3**_**A**_)/*K*(**T3**_**B**_) = 6.3, implying that the greater strain in **P3**_**THS**_ **Si·T3**_**B**_ makes it less stable. A similar competition experiment was carried out by titrating ***c*** **-P18**_**THS**_ with a mixture of **T18**_**A**_ and **T18**_**B**_ giving *K*(**T18**_**A**_)/*K*(**T18**_**B**_) = 0.6, showing that **T18**_**B**_ has a slightly higher affinity for ***c-*** **P18**_**THS**_ than **T18**_**A**_.

### Oxidative NMR Titrations for Probing Global Ring Currents

Previously, we probed the global aromatic and antiaromatic ring currents in template complexes of zinc porphyrin nanorings such as ***c-*** **P12**, as a function of oxidation state, by recording ^1^H and ^19^F NMR spectra while titrating with an oxidant.^8–12^ We used the same method to test for ring currents in ***c*** **-P18·T18**_**A**_ and ***c*** **-P18·T18**_**B**_, using thianthrenium tetrakis­(pentafluorophenyl)­borate (ThnBArF_20_) as the oxidant,^12^ carrying out titrations with both nanoring complexes in CDCl_3_ at both 25 °C and −40 °C. In these experiments, the quantity of added oxidant can be reliably confirmed from the ^1^H NMR spectra, because after 18 equiv of oxidant have been added, the presence of excess oxidant results in broadening of the thianthrene signals, due to fast chemical exchange between the neutral species and the radical cation. In contrast to previous work with ***c-*** **P12**, none of these titrations showed well resolved discrete ^19^F NMR CF_3_ resonances for different oxidation states. The room-temperature oxidation titrations provided no evidence for global ring currents. However, for the low-temperature titrations, deconvolution of the CF_3_ resonance during addition of 9 to 13 equiv of oxidant reveals shoulder peaks attributed to 9+, 10+, 11+ and 12+ oxidation states, that rise and decay during the course of the titration, as shown for ***c*** **-P18**_**THS**_ **·T18**_**B**_ in Figure 8a–e. Similar shoulder signals, with 10+ at low δ_F_ and 12+ at high δ_F_, were also observed in the low-temperature oxidation titration of ***c*** **-P18**_**THS**_ **·T18**_**A**_, although the shifts are less pronounced in this case, reflecting the weaker conjugation in the **T18**_**A**_ complex. These spectra provide evidence for weak global ring currents in the 10+ (diatropic; 242 π electrons) and 12+ (paratropic, 240 π electrons) oxidation states. During these titrations, a dominant peak is also observed at −59.2 ppm which does not shift significantly during oxidation (Figure 8a–e). We attribute this ‘residual peak’ to conformations of the nanoring that do not allow a completely π-conjugated pathway around the whole circumference of the nanoring. It appears that only a small fraction of the nanoring molecules are in a suitable conformation to sustain a global ring current.

**Figure 8:**
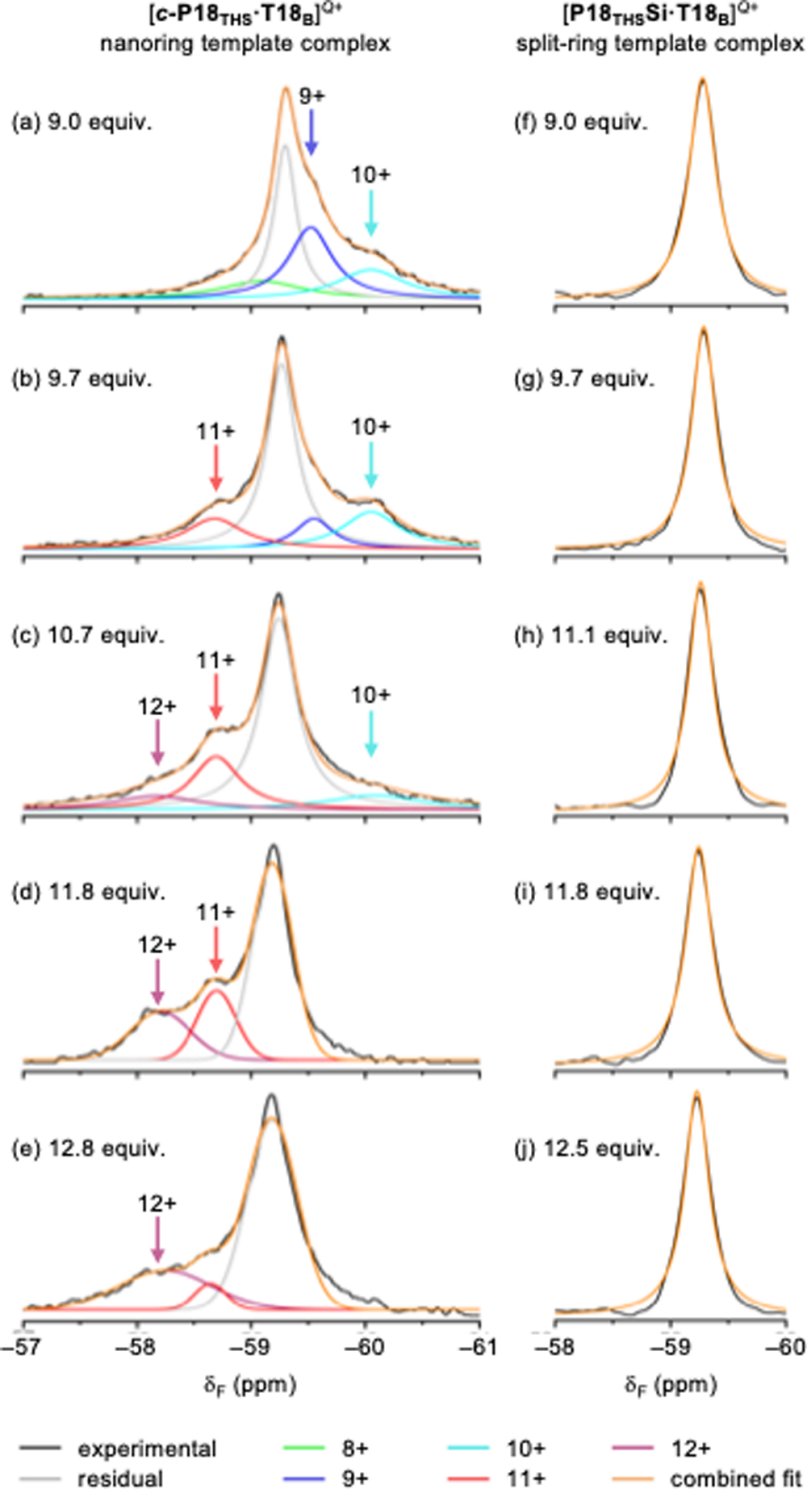
Selected ^19^F NMR spectra from the oxidative titration of ***c*** **-P18**_**THS**_ **·T18**_**B**_ (a–e) and **P18**_**THS**_ **Si·T18**_**B**_ (f–j) with ThnBArF_20_ in CDCl_3_ at −40 °C with 9.0–12.8 equiv of oxidant (470 MHz). The colored curves show deconvolution by fitting multiple Lorentzians, or a single Lorentzian in the case of **P18**_**THS**_ **Si·T18**_**B**_.

In a control experiment, a solution of the 1:1 complex of the linear porphyrin 18-mer **P18**_**THS**_ **Si** (Figure 4b) and **T18**_**B**_ was titrated with ThnBArF_20_ at −40 °C and ^19^F NMR spectra were recorded following an identical procedure to the titration with ***c*** **-P18**_**THS**_ **·T18**_**B**_. The CF_3_ signal recorded for [**P18**_**THS**_ **Si**·**T18**_**B**_]^*Q*+^ (Figure 8f–j) is sharper than that for [***c-*** **P18**_**THS**_·**T18**_**B**_]^*Q*+^ and shows no hint of any shoulder signals, which confirms that the shoulder signals observed for [***c-*** **P18**_**THS**_·**T18**_**B**_]^*Q*+^ are a consequence of the closed macrocyclic π-system, as expected for global ring currents.

We used the Biot-Savart law to estimate the strength of ring current that would account for the experimentally observed shielding of the CF_3_ resonance in [***c-*** **P18**_**THS**_·**T18**_**A/B**_]^10+^ and deshielding in [***c-*** **P18**_**THS**_·**T18**_**A/B**_]^12+^.^4a^ These calculations gave *I*/*B* = −2.4 and −4.2 nA/T for [***c-*** **P18**_**THS**_·**T18**_**A**_]^10+^ and [***c-*** **P18**_**THS**_·**T18**_**B**_]^10+^, respectively, and *I*/*B* = 2.3 and 10.9 nA/T for [***c-*** **P18**_**THS**_·**T18**_**A**_]^12+^ and [***c-*** **P18**_**THS**_·**T18**_**B**_]^12+^, respectively (where *I*/*B* is the ratio of the ring current to the applied magnetic field strength). These values are weaker than for benzene (*I*/*B* = −11.8 nA/T)^28^ and contrast with ***c-*** **P12**_**THS**_ which exhibits a ring current of *I*/*B* = −21.2 nA/T in the 6+ oxidation state.^4a^

### Computational Modeling of Global Aromaticity

Many computational methods have been developed for predicting whether a molecule is aromatic, antiaromatic or nonaromatic.^2,29,30^ For molecules the size of porphyrin nanorings, high-level methods, such as coupled-clusters, are prohibitively slow and expensive, and DFT-based techniques are most appropriate. A widely used approach is to calculate the nucleus-independent chemical shift (NICS),^30^ which measures the magnetic shielding at any point in space around a molecule. For extended π-systems such as porphyrin nanorings, the results of DFT NICS calculations are highly dependent on the choice of density functional.^31,32^ Standard functionals, such as B3LYP, exaggerate electronic delocalization and predict unrealistically large ring currents, due to self-interaction error.^33^ This can be corrected by increasing the amount of Hartee-Fock exact exchange (EE), but too much EE results in over localization, making the predicted ring currents too small. The problem of finding the right balance between localization (too much EE) and delocalization (too little EE) is well illustrated by ***c-*** **P6** ^**6+**^, in which B3LYP (19% EE) overestimates the global ring current and CAM-B3LYP (19–65%) underestimates it, while BLYP35 (35% EE) and LC-ωhPBE­(ω = 0.1) (0–100% EE) both come close to reproducing the shielding effects observed experimentally by NMR spectroscopy.^8,9,31,34,35^

Our attempted optimization of the geometries of ***c-*** **P18**·**T18**_**A**_ and ***c-*** **P18**·**T18**_**B**_ (in the neutral states) by DFT did not converge, as mentioned above, so we were unable to perform NICS calculations on these nanoring-template complexes. Instead, we performed calculations on ***c-*** **P18** (with no template and no aryl solubilizing groups). The geometry of ***c-*** **P18** was optimized using DFT (B3LYP/6–31g­(d)) at each even oxidation state, neutral to 18+. We then calculated NICS(0)_*zz*_, across the plane of the 18 Zn atoms (where *zz* denotes shielding in the *z* direction caused by an external field in the *z* direction, and 0 indicates the *x,y*-plane, *z* = 0) using the two functionals that work well for ***c-*** **P6** ^**6+**^, BLYP35 and LC-ωhPBE­(ω = 0.1). Maps of the NICS values in oxidation states 10+, 12+, 14+ and 16+ are similar for the two functionals, as shown in Figure 9 (for BLYP35) and Figure S40 (for LC-ωhPBE­(ω = 0.1)). These plots indicate that the 10+ (242 electrons) and 14+ (238 electrons) states are aromatic, and that the 12+ (240 electrons) and 16+ (236 electrons) states are antiaromatic, in accord with the Hückel rule. These results agree qualitatively with the experimental observation of a global aromatic ring current in the 10+ oxidation state and an antiaromatic ring current in the 12+ oxidation state, presented above. The experimental shielding effects are smaller than those predicted computationally, which may indicate that these functionals do not include sufficient EE, or it may reflect the fact that the geometries of [***c-*** **P18**·**T18**_**A**_]^*Q*+^ and [***c-*** **P18**·**T18**_**B**_]^*Q*+^ are different from those of ***c-*** **P18** ^*Q*+^. Another factor is that the geometries of ***c-*** **P18** ^*Q*+^ were optimized with B3LYP, not with the functional used for the NICS calculations, to reduce the cost of the calculations. It should be emphasized that, while the NICS calculations on the 10+ and 12+ oxidation states agree with the experimental evidence for weak global diatropic and paratropic ring currents, respectively, our conclusions about the aromaticity of these systems are based entirely on the experimental NMR data, not on the computational results.^36^

**Figure 9:**
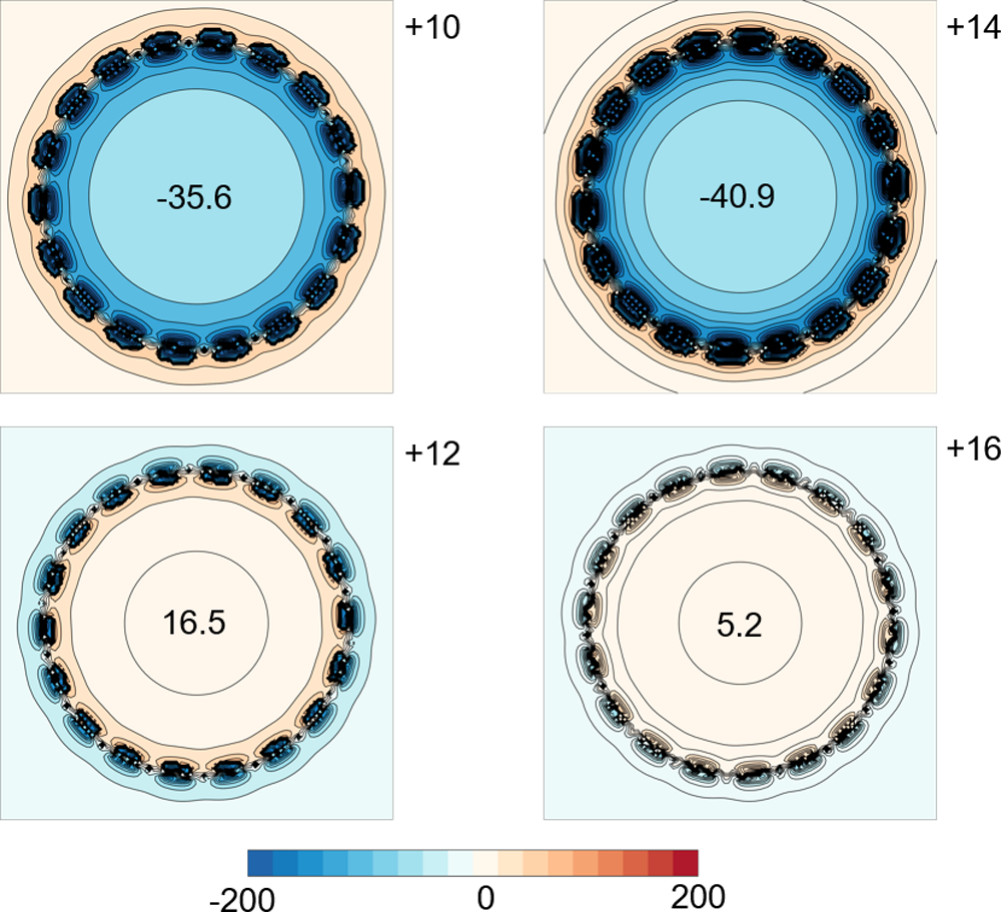
Plots of NICS(0)_*zz*_ in the *xy*-plane of ***c*** **-P18** in the +10, + 12, + 14, and +16 states (geometries optimized with B3LYP; NICS calculated with BLYP35). Blue denotes shielding and red deshielding. NICS(0)_*zz*_ values at the center of each ring are marked in the center of each plot.

## Conclusions

We have synthesized two 18-legged templates (**T18**_**A**_ and **T18**_**B**_) for binding an 18-porphyrin nanoring ***c-*** **P18**. The two templates are equally effective for directing the synthesis of this nanoring and bind the nanoring with almost equal affinities. The template with all-*para* pyridyl substituents, **T18**_**B**_, holds the nanoring in a more π-conjugated geometry, with smaller dihedral angles between neighboring porphyrins, resulting in a more red-shifted absorption spectrum. The low-temperature ^19^F NMR spectra of both template complexes, in the presence of 9–18 equiv of oxidant, show shoulder peaks indicating the formation of oxidation states exhibiting diatropic or paratropic global ring currents. These shoulder peaks do not appear in control experiment with a split-ring complex of linear 18-mer bound to **T18**_**B**_, confirming that they arise from global ring currents. Only a fraction of the ***c-*** **P18** nanoring molecules appear to adopt the right conformation to sustain a global ring current, and the strength of global ring currents in ***c-*** **P18**_**THS**_·**T18**_B_ in the 10+ and 12+ oxidation state are *I*/*B* = −4.2 and +10.9 nA/T, which is weaker than that of benzene,^29^ and much weaker than that of ***c-*** **P12** in the 6+ oxidation state.^4a,9^ These results indicate that ***c-*** **P18** defines the upper size limit to global aromaticity in butadiyne-linked porphyrin nanorings.

The decline in the magnitude of the global ring current in large nanorings can be attributed to conformational disorder, which interrupts the coherence of the electronic wave function around the circumference of the ring. Thus, in the 10+ and 12+ oxidation states of ***c-*** **P18**_**THS**_·**T18**_B_, evidence for global ring currents is only detected at low temperatures (−40 °C rather than 25 °C) and only a small fraction of the nanoring molecules are in a suitable conformation to sustain a global ring current. In general, for all types of π-conjugated macrocycles, aromatic stabilization energy decreases with increasing ring size, as understood at the simplest level from the fact that the energy levels of a Frost-Musulin diagram become closer together, and also by the onset of pseudo-Jahn–Teller distortions.^11,32,37^ In contrast, the magnitude of the ring current peaks at a certain ring size, then declines.^32,38^ For example, [18]­annulene has a stronger ring current than benzene, but the ring current is weaker in [22]­annulene and larger homologues.^4a^

Cationic butadiyne-linked porphyrin nanorings are currently the largest macrocycles with experimental evidence for global aromatic ring currents, and they seem to reach a limit at ***c-*** **P18** ^10+^ (242 π-electrons, diameter 8 nm). Other more rigid and more strongly coupled macrocycles (such as edge-fused porphyrin nanobelts^32,39^ ) may allow global ring currents to persist in even larger rings.

## Supplementary Material





## Notes

Synthetic procedures, characterization data, and *x,y,z-*coordinates of calculated molecular geometries (PDF)
c-P18 ms xyz coordinates (ZIP)

## Notes

Department of Chemistry, Graduate School of Science, Nagoya University, Chikusa, Nagoya 464-8602, Japan
