# Supplementary material for: An 18-Porphyrin Nanoring at the Size Limit for Global Aromaticity
Source: J Am Chem Soc. 2025 Aug 28;147(36):32840–50. doi: 10.1021/jacs.5c09149 (PMC12426941; doi:10.1021/jacs.5c09149)
Supplement: Supplementary file 1 [file ja5c09149_si_001.pdf]

Supporting Information for

An 18-porphyrin nanoring at the size limit for global aromaticity

Jake M. Holmes,[1] Henrik Gotfredsen,*[1] Lene Gödde,[1] Janko Hergenhahn,[1] Kavita Rani,[1] Keigo E. Yamada,[1] Jie-Ren Deng,[1] Liv Warwick,[2] Michael Clarke,[2] Matthew Edmondson,[2] James N. O'Shea,[2] Alex Saywell,*[2] and Harry L. Anderson*[1]

[1] Department of Chemistry, University of Oxford, Chemistry Research Laboratory, Oxford OX1 3TA, UK
[2] School of Physics & Astronomy, University of Nottingham, Nottingham, NG7 2RD, UK

Correspondence to: henrik_gotfredsen@hotmail.com (H.G.), alex.saywell@nottingham.ac.uk (A.S.) and harry.anderson@chem.ox.ac.uk (H.L.A.)

Table of contents

1. General methods S2
2. Computational modelling S3
3. Molecular dynamics simulations S4
4. Synthesis of known intermediates S6
5. Overview of synthesis S7
6. Synthetic procedures and compound characterization S10
7. Scanning probe microscopy S40
8. UV-visible titrations S42
9. NICS calculation S51
10. Oxidative NMR titrations S53
11. Quantification of ring currents from experimental NMR shifts S64
12. NMR and mass spectra of new compounds S65
13. References S188

xyz-Coordinates of calculated molecular geometries relating to this article are available on Zenodo, https://doi.org/10.5281/zenodo.16038094

Section 1. General methods

Diisopropylamine (DIPA) and dichloromethane for reactions were obtained from an MBraun MBSPS-5-BenchTop solvent purification system kept under nitrogen. Chloroform-*d* for NMR was stored over K_2CO_3 prior to use. All other reagents and solvents were obtained from commercial suppliers and used as received unless otherwise stated. Thin-layer chromatography (TLC) was carried out using commercially available aluminum sheets precoated with silica gel with fluorescence indicator and visualized under UV light at 254 or 360 nm. Purification by column chromatography was carried out on silica gel (SiO_2, 60 Å, 40−63 µm). Petroleum ether (PE) of boiling range: 40−60 °C was used for chromatography. Size exclusion chromatography (SEC) was carried out using Bio-Rad Bio-Beads S-X1 (40–80 µm bead size). Analytical gel permeation chromatography (GPC) was carried out using JAIGEL-3H-A (8 × 500 mm) and JAIGEL-4H-A (8 × 500 mm) columns in THF + 1% pyridine as eluent with a flow rate of 1.0 mL/min. Semi-preparative GPC was carried out on a Shimadzu recycling GPC system equipped with a LC-20 AD pump, SPD-20A UV detector and a set of JAIGEL 3H (20 × 600 mm) and JAIGEL 4H (20 × 600 mm) columns in toluene + 1% pyridine as the eluent at a flow rate of 3.5 mL/min.

^1H and ^{13}C NMR spectra were recorded on either a Bruker AVIII HD 400, a Bruker AVIII HD 500, a Bruker AVII 500 with a $^{13}C(^1H)$ dual cryo-probe, or a Bruker AVIII 600 with a broadband cryo-probe. ^{19}F NMR spectra were recorded on a Bruker AVIIIHD 500 instrument. Chemical shift values are quoted in ppm and coupling constants (J) in hertz to the nearest 0.1 Hz. ^1H and ^{13}C NMR spectra are referenced against the residual solvent peak ($CHCl_3$ δ_H = 7.26 ppm, $CDCl_3$ δ_C = 77.16 ppm; $CHDCl_2$ δ_H = 5.32 ppm, CD_2Cl_2 δ_C = 53.84 ppm, $C_2H_2Cl_4$ δ_H = 6.00 ppm, $C_2D_2Cl_4$ δ_C = 73.78 ppm). ^{19}F NMR spectra are referenced against hexafluorobenzene (δ_F = –164.8 ppm) which was added to the samples. Unless stated otherwise, NMR spectra were recorded at 298 K.

UV-vis-NIR measurements were carried out in a 1 cm path length quartz cuvette at 298 K using either a Perkin-Lambda 20 or a Jasco V770 spectrophotometer. Chloroform (stored over K_2CO_3) was used as solvent.

MALDI-ToF mass spectra were measured using a Bruker MALDI Autoflex Speed instrument. Either dithranol or *trans*-2-[3-(4-*tert*-butylphenyl)-2-methyl-2-propenylidene]malononitrile (DCTB) was used as matrix. ESI spectra were measured at the University of Oxford on a Thermo Orbitrap Exactive mass spectrometer.

Oxidative NMR titrations on porphyrin nanoring complexes were carried out similarly to previous descriptions.[1,2] The nanoring complexes were transferred to J. Young NMR tubes, dissolved in $CDCl_3$, and handled under argon using standard Schlenk techniques. A solution of the oxidant, thianthrenium tetrakis(pentafluorophenyl)borate, ThnBArF, (Thn^+, E_{red} = 0.84 V versus Fc/Fc^+)[3] in $CDCl_3$, kept under argon was used for the titrations. The titrations were carried out using a Schlenk adaptor for the J. Young NMR tubes, enabling the addition of oxidant under a counterflow of argon. For low temperature experiments, while having the NMR tube positioned in the Schlenk adaptor, the lower part of the tube was simultaneously immersed in a dry ice/acetone mixture (–78 °C) in a Dewar to maintain the temperature of the sample. After the first addition of oxidant, the samples were kept at low-temperatures throughout the titration experiments using either a dry ice/acetone Dewar or the N_2 cooling (–40 °C) as used for the NMR probe. Only during transfers (≤ 30 seconds) from the NMR instrument to the Dewar or from the NMR tube adaptor to the Dewar were the samples momentarily subjected to room temperature conditions. Prior to each titration, a ^1H spectrum was recorded at 25 °C to confirm integrity and purity of the porphyrin nanoring complex. At the end of a titration, the oxidized porphyrin nanoring complex was reduced (using decamethylferrocene) back to recover the neutral state, as confirmed by ^1H NMR at 25 °C. The titration endpoint (at which each porphyrin unit is oxidized to the 1+ state) is evident from the disappearance of the neutral oxidant (thianthrene) ^1H signals, resulting from signal broadening caused by exchange between neutral thianthrene and excess thianthrenium.

Section 2. Computational modelling

T3 and **T18** candidates, along with **P3** and ***c*-P18** were constructed and manually docked together using Gaussview.[4] To minimize the cost of geometry optimizations, the CF_3 and C_8H_{17} groups in **T3** and **T18** were replaced with hydrogen atoms, as were the aryl solubilizing groups in **P3** and ***c*-P18**. All structures were initially optimized using the PM7 method[5] in MOPAC,[6] then further refined with DFT calculations using Gaussian16[7] and the B3LYP/6-31g(d) functional/basis set.[8,9] Due to the large size of the ***c*-P18·T18** complex, DFT calculations on these structures did not converge quickly enough to be cost effective. Therefore, the ***c*-P18·T18** complex structures were not refined beyond the PM7 level of theory.

Various designs for the tridentate binding site (**T3**) coordinated to a linear butadiyne-linked porphyrin trimer (**P3**) and shown in Figure S1. Despite giving reasonable geometries for the **P3·T3** complex, the designs **T3**$_D$–**T3**$_F$, containing thiophene and furan moieties were discarded, as they were deemed too flexible.

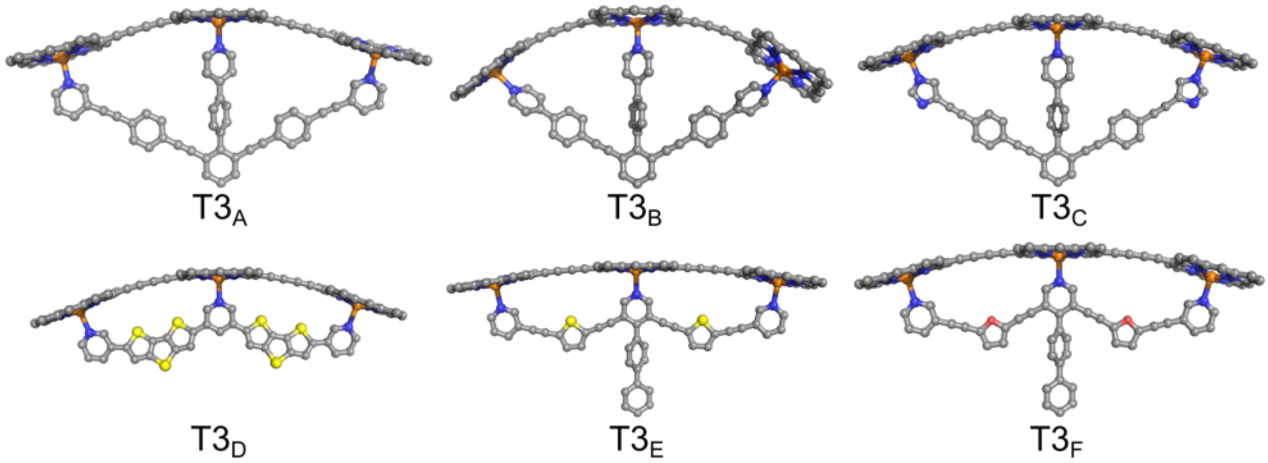

Figure S1. DFT optimized models of **P3·T3** complexes using the B3LYP/6-31g(d) functional/basis set. (Color code: nitrogen blue; zinc: orange; oxygen: red; sulfur: yellow.)

The remaining three designs, **T3**$_{A–C}$, were constructed into full template models and manually docked into ***c*-P18** using HyperChem. Figure S2 shows the optimized models of these complexes, all of which give reasonable geometries. Although the imidazole containing template appears to be a good fit for ***c*-P18**, this design was discarded due to concerns over the proposed synthetic route. Imidazole containing templates have previously been synthesized in our group,[10] but their high polarity can make them difficult to purify.

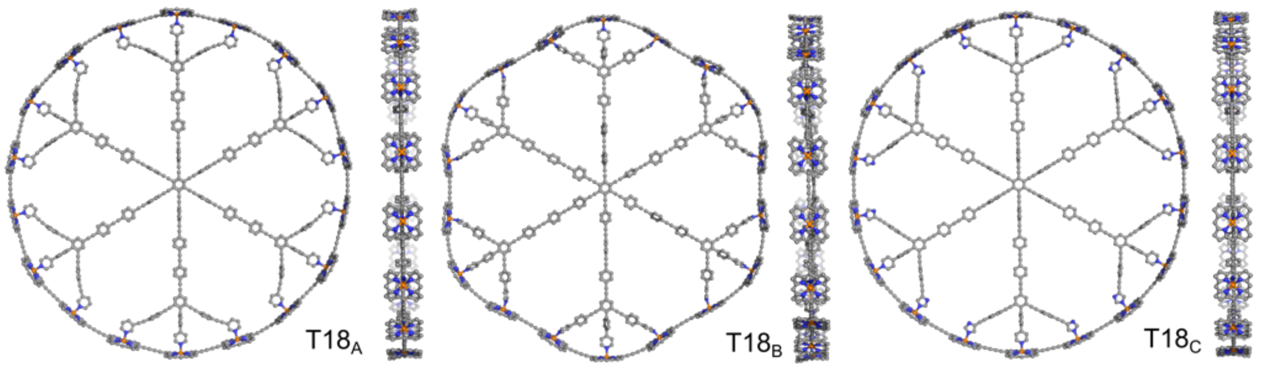

Figure S2. PM7 optimized models of three ***c*-P18·T18** complexes.

To explore whether the all-pyridine design **T18**$_A$ is the optimal design for binding ***c*-P18**, additional models were constructed in which either the distance between **T3** and the center of the template, or the length of the side arms of **T3**, were varied. These optimized structures (PM7, MOPAC. Figure S3) show significant distortion to either the ***c*-P18** ring or template, which indicates that they are poor fits for ***c*-P18**.

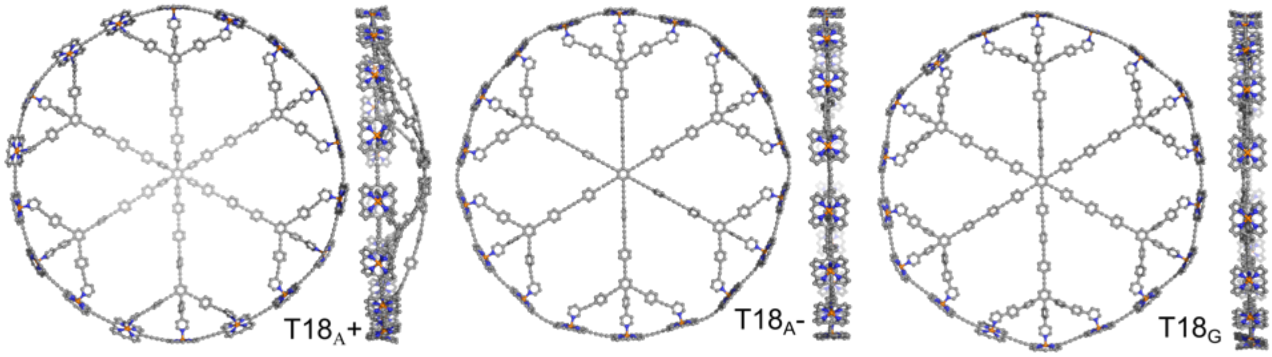

Figure S3. PM7 optimized models of *c*-**P18·T18** complexes each with slight variations in the total size of **T18**. One acetylene per branch replaced with a *para*-phenylene group (**T18A+**). One a *para*-phenylene per branch replaced with an acetylene (**T18A-**). Two acetylenes in side arm of **T3A** substituted for a *para*-phenylene group (**T18G**).

Section 3. Molecular dynamics simulations

Methodology. All molecular dynamics (MD) simulations were performed in an isothermal-isobaric (NPT) ensemble at 300 K and 1 bar with a time step of 2 fs using GROMACS (v. 2019.2).[11] The duration of the MD simulations was 200 ns (10^8 time steps) for the linear porphyrin trimer systems and 50 ns (2.5×10^7 time steps) for the cyclic 18-mer systems. Simulations employed the General AMBER Force Field[12] with modifications to parameters for zinc ions and porphyrin connections as previously published.[13,14] All aryl solubilizing groups were replaced with phenyl substituents to reduce system size and increase the speed of the simulations. Systems were minimized using the steepest descent algorithm for 5000 steps or until the maximum force on any atom was below 1000 kJ mol^{-1} nm^{-1} and subsequently equilibrated using a velocity-rescaling thermostat[15] and Parrinello-Rahman barostat.[16] All simulations were performed in explicit chloroform[17] with three-dimensional periodic boundary conditions. The box sizes were chosen by leaving 1 nm distance between solute and box boundary. Long-range electrostatic interactions were calculated using the particle mesh Ewald method.[18] All bond lengths involving hydrogen atoms were constrained with the LINCS algorithm.[19] All simulations were repeated three times with different random seeds and all resulting trajectories were used for the analysis below.

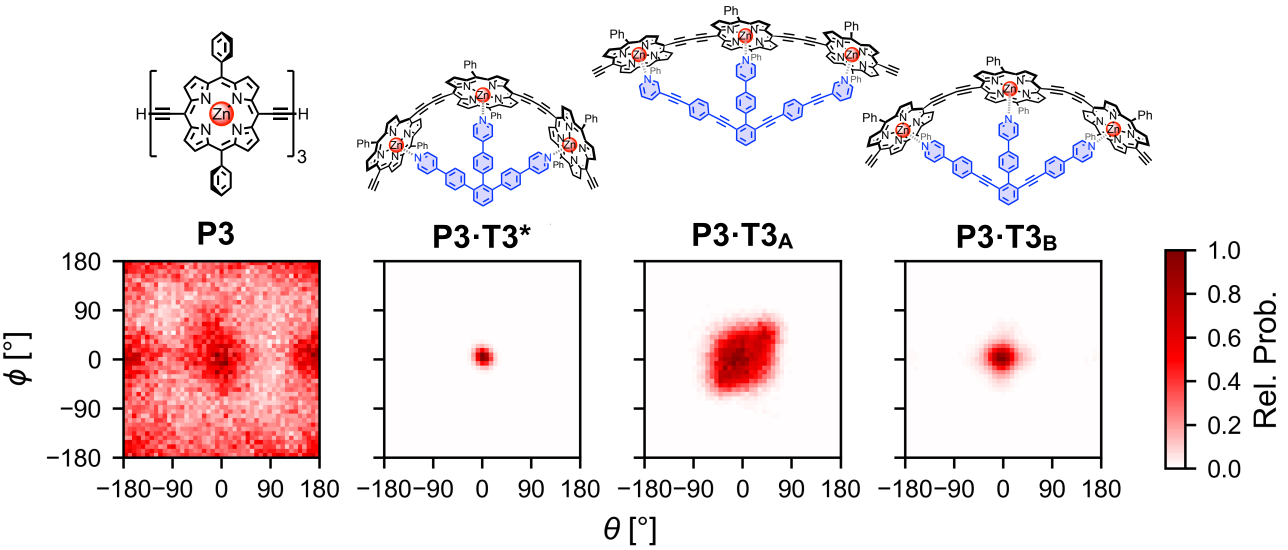

Figure S4. 2D histograms of the dihedral angles between porphyrins in **P3** and with different tridentate ligands.

Linear porphyrin trimer. The binding of different tridentate ligands to **P3** was investigated with molecular dynamics simulations. The free trimer **P3** was simulated as a reference and a 2D-histogram of the two dihedral angles between adjacent porphyrins (Figure S4) shows a broad distribution with maxima centered around 0° and ±180° for both angles. This shows the expected preference for co-planar porphyrins and that rotation around the bond axis happens on the ns time scale. All three ligand complexes that were simulated

show more constrained dihedral angle histograms in comparison to free **P3**. Ligand binding to **P3** inhibits full rotation around the bond-axis, restraining the dihedral angles close to (0°|0°). **T3*** is a very rigid ligand and provides a comparison for the newly designed tridentate ligand in terms of how much they organize the porphyrins in **P3**. The **P3·T3*** complex is exceptionally constrained with a standard deviation of only 10.4°. **T3**$_{\mathbf{A}}$ does not rigidify **P3** to the same extent, giving a much broader distribution of dihedral angles with a standard deviation of 34.0°, showing that the porphyrins can still tilt relative to each other, despite the ligand. The distribution has a noticeable covariance between the two dihedral angles of 343.8 (compared to –11.8 in **P3·T3***) indicating that the two outer porphyrins are more likely to tilt in the same direction. **T3**$_{\mathbf{B}}$ gives a narrower, more constrained distribution with a standard deviation of 19.8°.

Cyclic Porphyrin Oligomers. MD simulations on cyclic porphyrin oligomers were used to assess to what extent a template holds all the porphyrins in an organized arrangement. This is important for ring-current NMR experiments where there needs to be a global conjugated pathway. First, simulations were done on rings that have been studied previously and that are known to have a global ring current in their template-bound form (in certain oxidation states). Alignment of porphyrins was assessed by determining the dihedral angle between adjacent porphyrin units and defining a cut-off of 45° below which they are considered to be co-planar. Figure S5 and S6 shows the distributions of the maximum number of co-planar porphyrins present over the course of the simulations. While porphyrins are mostly well aligned in the free ***c*-P6** ring, all conformations in ***c*-P6·T6** have all six porphyrins aligned during the simulation. Similarly, the 12-porphyrin nanoring complex ***c*-P12·(T6**$_{\mathbf{L}}$**)**$_{\mathbf{2}}$ is more organized than free ***c*-P12**, and the difference is much more pronounced in this larger system compared with ***c*-P6**. While most conformations of free ***c*-P12** have only 2–5 neighboring porphyrins aligned, in ***c*-P12·(T6**$_{\mathbf{L}}$**)**$_{\mathbf{2}}$ many structures have all porphyrins fully aligned, which is consistent with an observable ring current in this system. Simulations of ***c*-P18** show it has similar flexibility to ***c*-P12**. Binding of the template **T18**$_{\mathbf{A}}$ leads to an increased alignment of porphyrins, but not nearly enough to organize the complete structure, as most conformations only have up to 10 porphyrins aligned. **T18**$_{\mathbf{B}}$ introduces much more order into the ***c*-P18** ring, with a higher population of fully organized ring, which is consistent with simulations of the tridentate ligand systems showing that **P3·T3**$_{\mathbf{B}}$ is more rigid than **P3·T3**$_{\mathbf{A}}$. This explains the red-shifted absorption spectrum of ***c*-P18·T18**$_{\mathbf{B}}$ and suggests that this complex is more likely than ***c*-P18·T18**$_{\mathbf{A}}$ to exhibit global ring current effects.

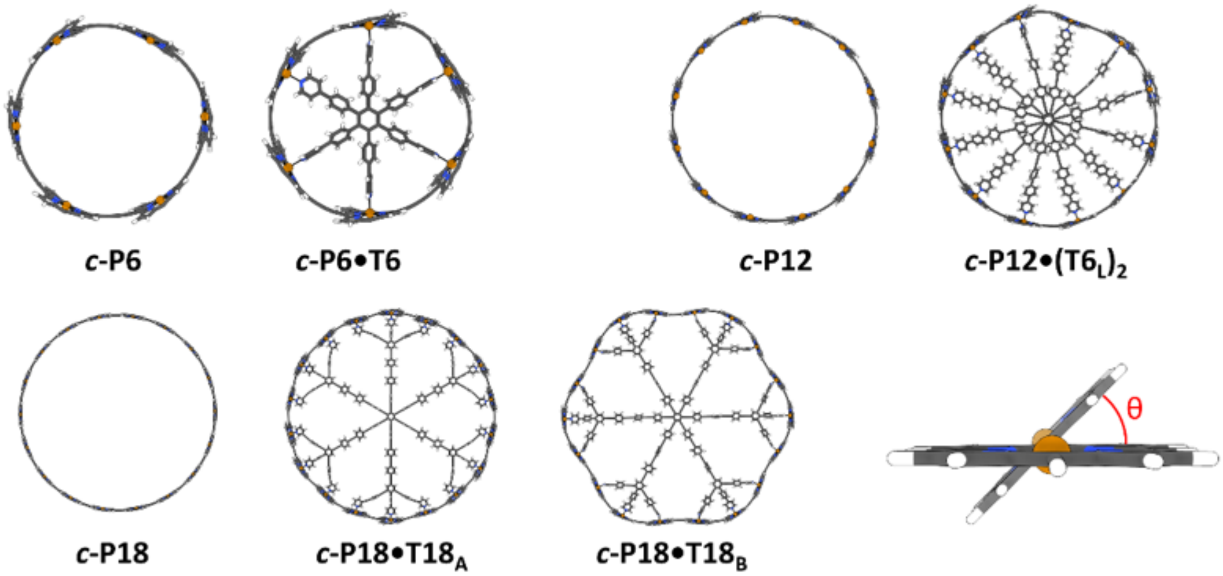

Figure S5. Structures of all cyclic structures that were simulated and of **P2** showing the cut-off angle of 45° that was used to define co-planarity.

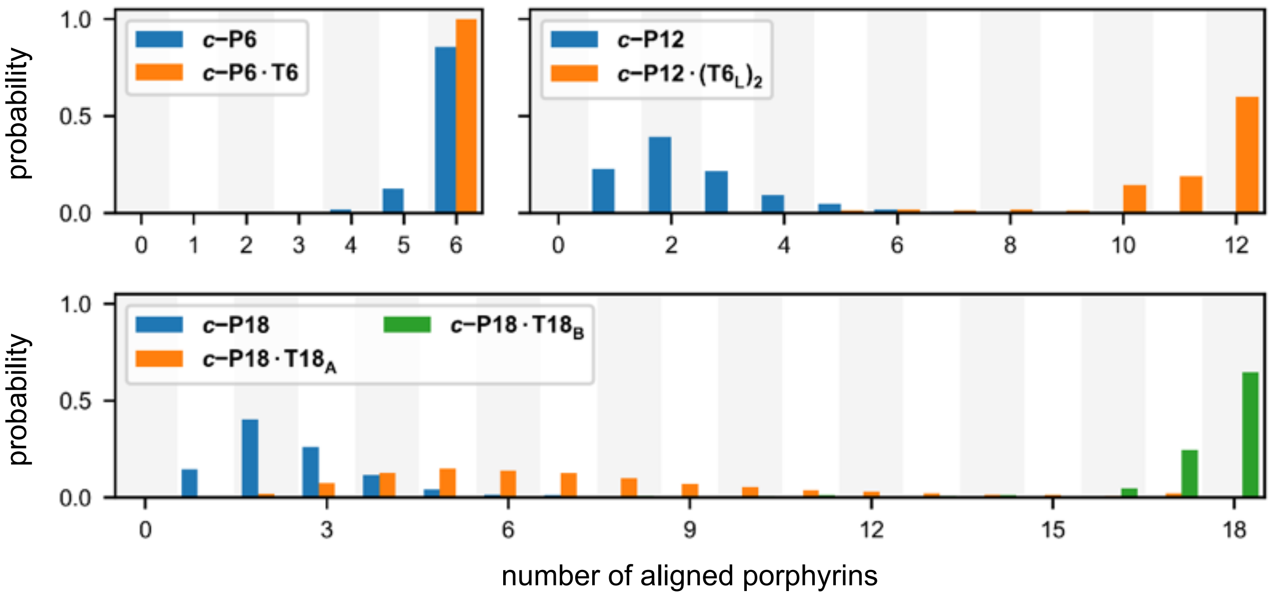

Figure S6. Distributions of number of coplanar porphyrins for different cyclic systems.

Section 4. Synthesis of known intermediates

Porphyrin oligomers, **P3**$_{\mathbf{OOct}}$[20] and **P3**$_{\mathbf{THS}}$,[21] and template precursors, **1**,[22] **S1**,[23] **7**,[24] **S2**,[25] **5**,[25] **S3**,[26] **S4**,[27] **16**,[2] and **T6**,[20] were synthesized using published procedures.

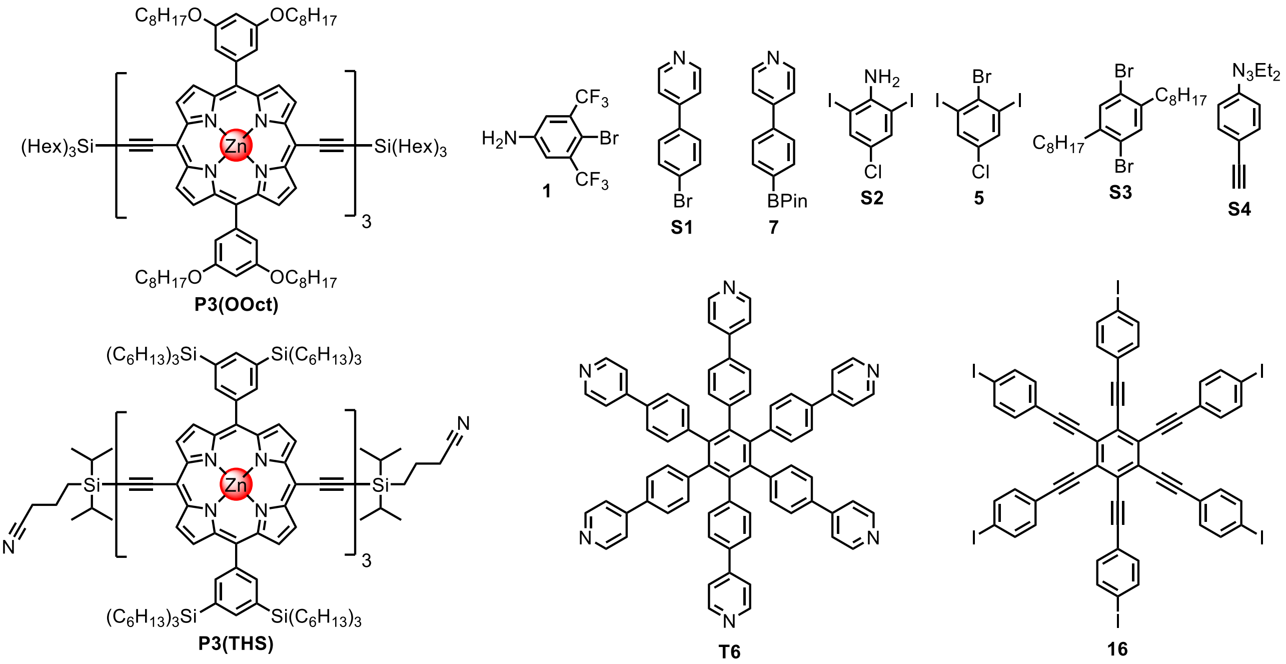

Figure S7. Known compounds synthesized for this project.

Section 5. Overview of synthesis

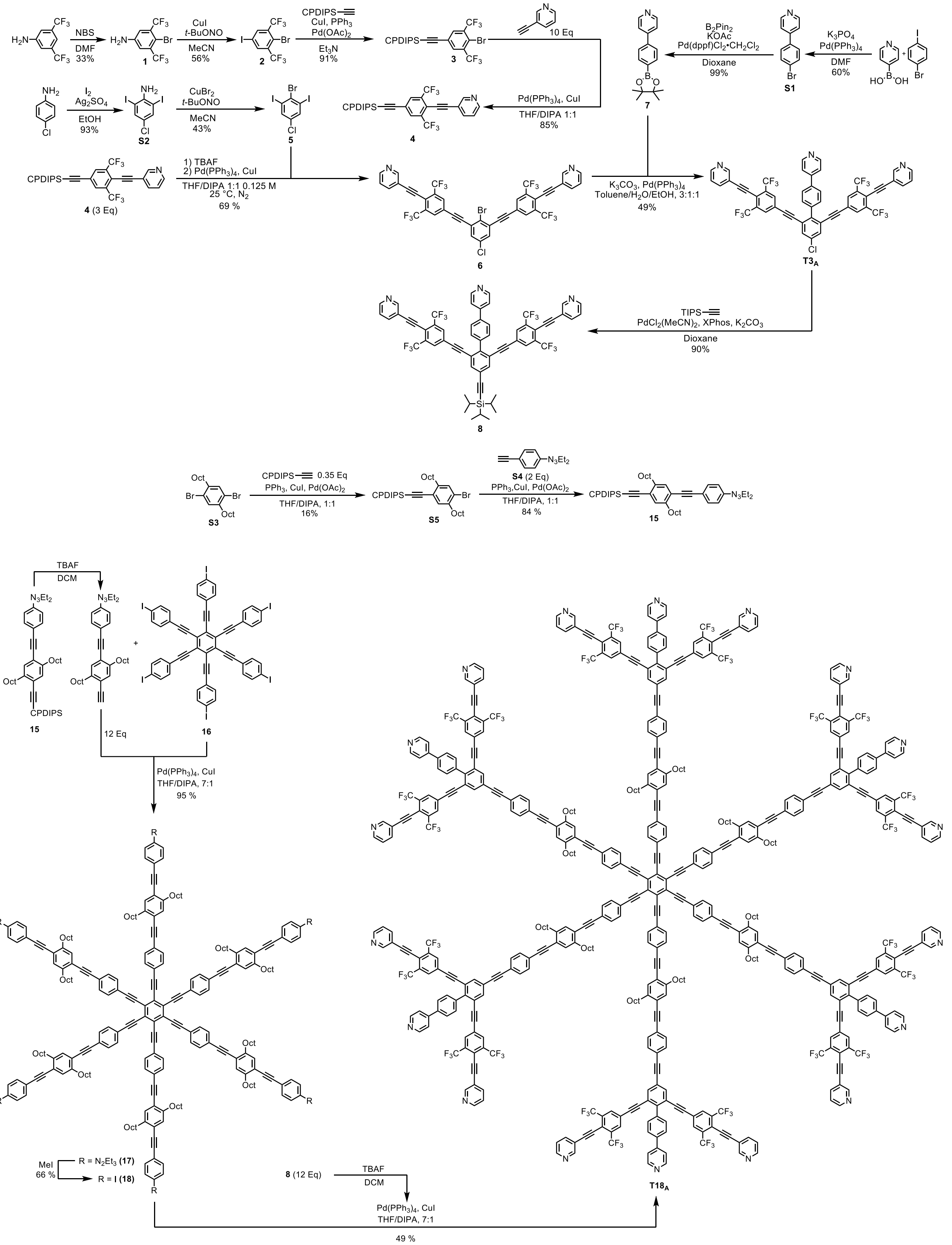

Scheme S1. Synthesis of **T18**$_A$.

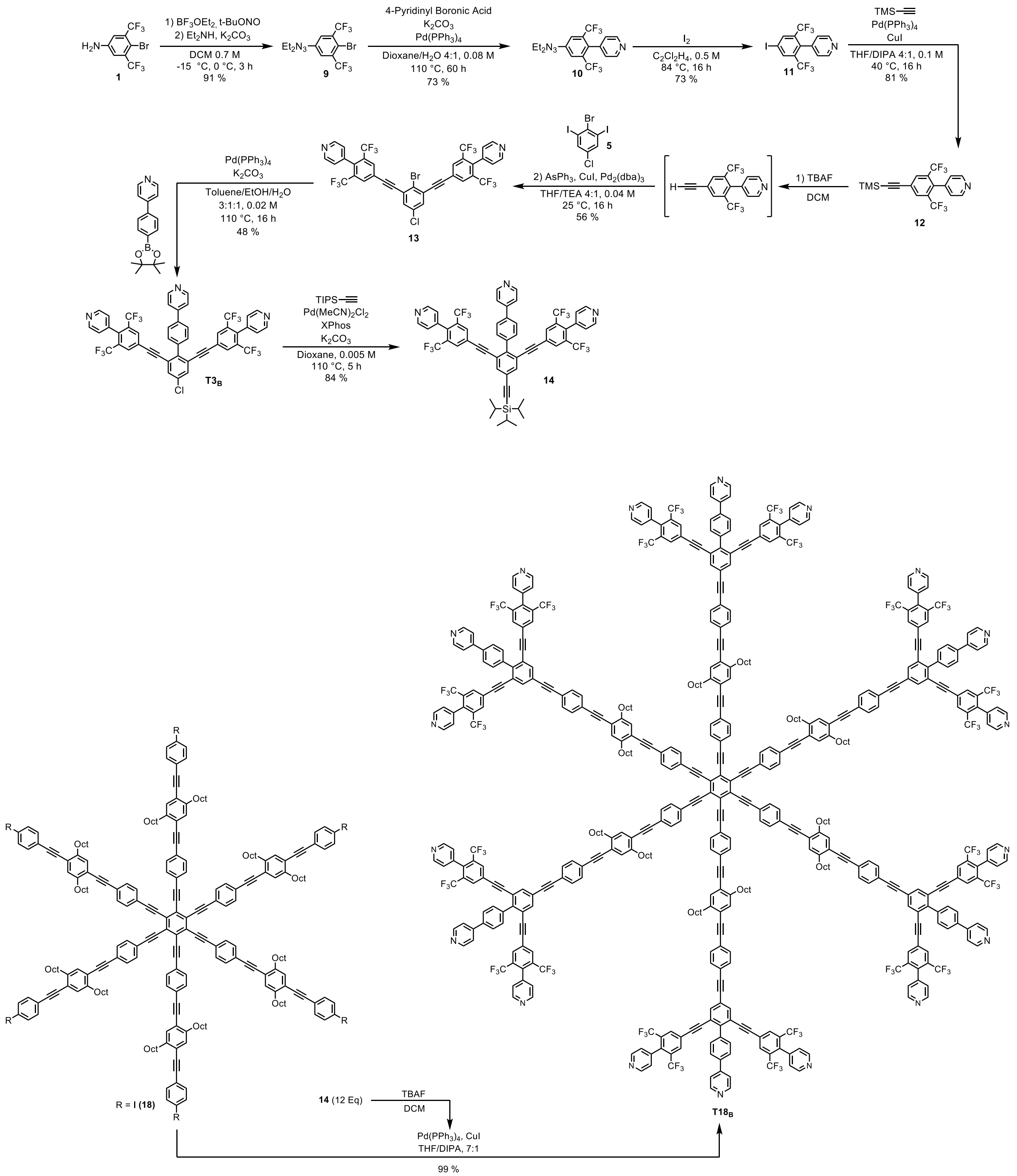

Scheme S2. Synthesis of **T18$_B$**.

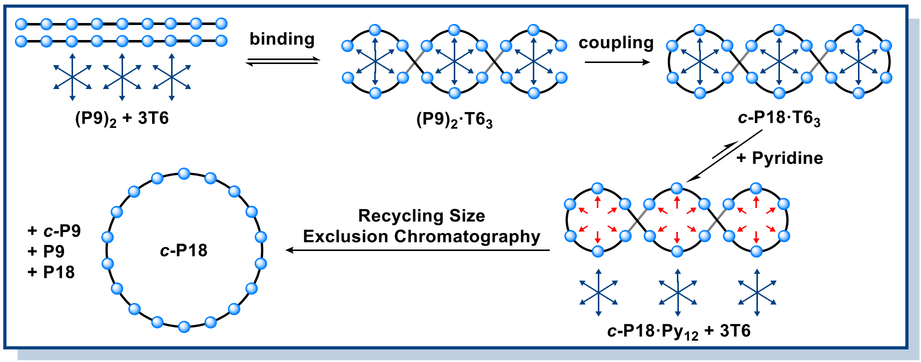

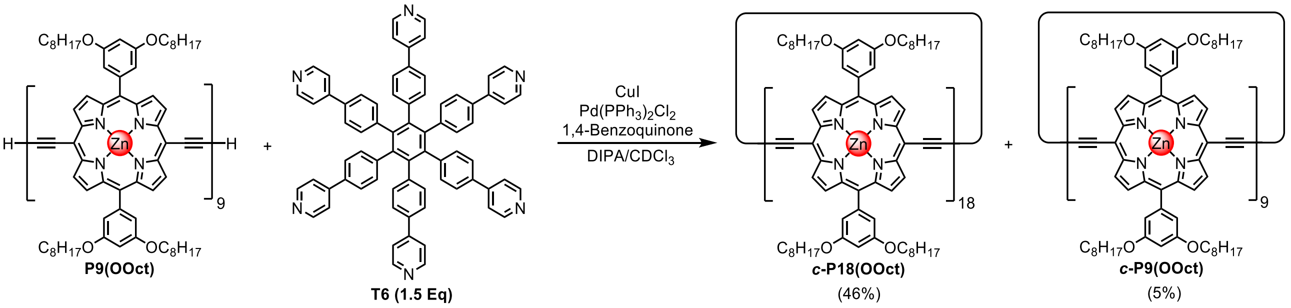

Scheme S3. Vernier template-directed synthesis of ***c*-P18**$_{\mathbf{OOct}}$ nanoring.

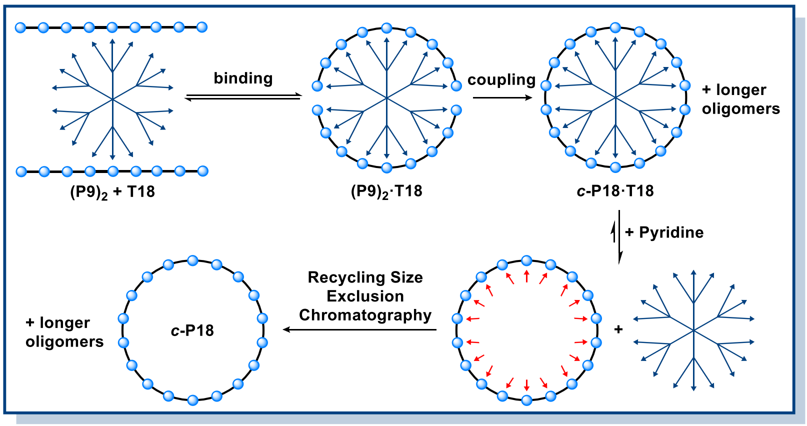

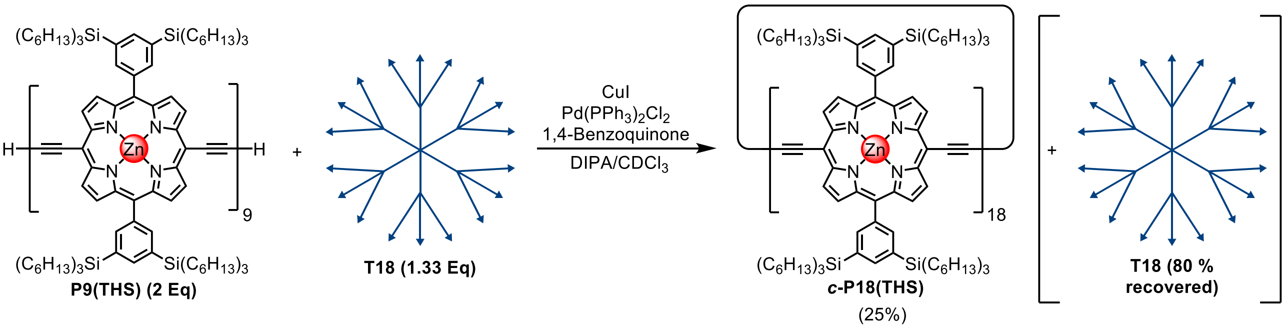

Scheme S4. Template-directed synthesis of ***c*-P18**$_{\mathbf{THS}}$ nanoring.

Section 6. Synthetic procedures and compound characterization

Synthesis of 2-bromo-5-iodo-1,3-bis(trifluoromethyl)benzene (2)

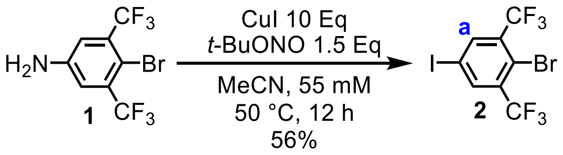

To a stirring solution of 3,5-bis(trifluoromethyl)-4-bromoaniline (**1**) (526 mg, 1 Eq, 1.71 mmol) in MeCN (31 mL) at 0 °C was added CuI (3.25 g, 10 Eq, 17.1 mmol). After 5 min of stirring, *t*-BuONO (305 µL, 1.5 Eq, 2.56 mmol) was added and the reaction vessel removed from the ice bath, then heated to 50 °C for 16 h. After cooling to room temperature, EtOAc (100 mL) was added and the reaction mixture transferred to a separating funnel. The organic phase was then washed with saturated $Na_2S_2O_3 \cdot 5H_2O$ (3 × 100 mL), dried over Na_2SO_4, concentrated and purified by flash column chromatography (SiO_2, PE) to afford compound **2** as a white crystalline solid (339 mg, 56%).

^{1}H NMR (400 MHz, $CDCl_3$) δ_H = 8.16 (s, 2H, **H$_a$**) ppm.
^{19}F NMR (377 MHz $CDCl_3$) δ_F = –62.32 ppm (referenced against C_6F_6 (δ_F –161.64) as an internal standard).
^{13}C NMR (101 MHz, $CDCl_3$) δ_C = 139.96 (q, J_{FC} = 5.9 Hz), 134.30 (q, J_{FC} = 31.5 Hz), 121.60 (q, J_{FC} = 275.7 Hz), 119.05, 91.55 ppm.

Synthesis of 7-(4-bromo-3,5-bis(trifluoromethyl)phenyl)-5,5-diisopropylhept-6-ynenitrile (3)

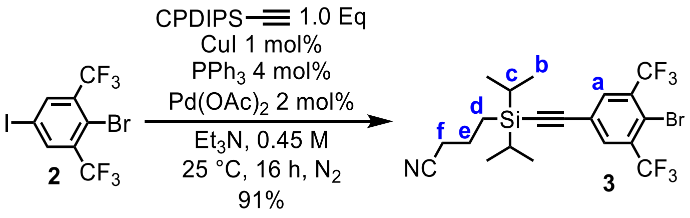

To an oven-dried Schlenk tube was added **2** (250 mg, 1 Eq, 597 µmol), CuI (1.14 mg, 0.01 Eq, 5.97 µmol), PPh_3 (6.26 mg, 0.04 Eq, 23.9 µmol) and $Pd(OAc)_2$ (2.49 mg, 0.02 Eq, 11.9 µmol). The tube was then evacuated/refilled with N_2 (3×). Dry triethylamine (1.33 mL) was added and the reaction mixture freeze-pump-thaw degassed (3×). Under a positive pressure of N_2, CPDIPS-acetylene (138 µL, 1 Eq, 597 µmol) was then added and the reaction mixture stirred at room temperature for 16 h. The resulting pale-yellow suspension was diluted with CH_2Cl_2 (20 mL), concentrated in vacuo and loaded onto a silica gel column (SiO_2, 3.5 cm (*d*) × 14 cm (*h*), PE) for purification by flash column chromatography (0–20% CH_2Cl_2 in PE) to afford **3** as a pale yellow oil (271 mg, 91%).

^{1}H NMR (400 MHz, $CDCl_3$) δ_H = 7.90 (s, 2H, **H$_a$**), 2.44 (t, J = 6.9 Hz, 2H, **H$_f$**), 1.89–1.81 (m, 2H, **H$_e$**), 1.13–1.08 (m, 14H, **H$_b$**, **H$_c$**), 0.91–0.82 (m, 2H, H$_d$) ppm.
^{19}F NMR (377 MHz $CDCl_3$) δ_F = –62.30 ppm (referenced against C_6F_6 (δ_F –161.64) as an internal standard)
^{13}C NMR (101 MHz, CDCl3) δ_C = 133.99 (q, J_{FC} = 5.8 Hz), 133.25 (q, J_{FC} = 31.4 Hz), 123.33, 122.25 (q, J_{FC} = 275.4 Hz), 119.65, 118.80, 103.65, 95.43, 21.24, 20.93, 18.23, 18.00, 11.64, 9.54 ppm.

Synthesis of 5,5-diisopropyl-7-(4-(pyridin-3-ylethynyl)-3,5-bis(trifluoromethyl) phenyl)hept-6-ynenitrile (4)

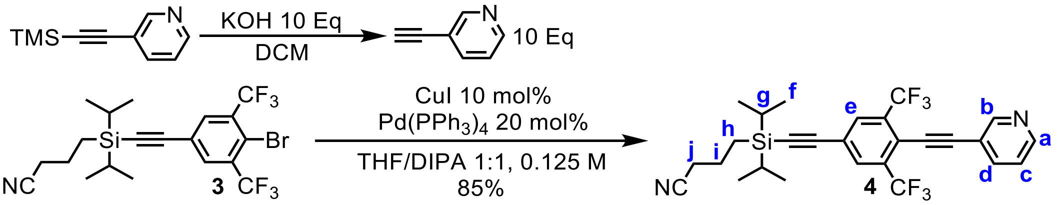

To a stirring solution of KOH (3.15 g, 10 Eq, 56.2 mmol) in CH_2Cl_2 (150 mL) and MeOH (25 mL) was added 3-((trimethylsilyl)ethynyl)pyridine (10.7 mL, 10 Eq, 56.2 mmol). After 1 hour the reaction mixture was passed through a short plug of silica gel (CH_2Cl_2 + 2% MeOH) and concentrated in vacuo to afford 3-ethynylpyridine as a white crystalline solid, which was used in the next step with no further purification. In an oven dried Schlenk tube, 3-ethynylpyridine from the previous step and **3** (2.80 g, 1 Eq, 5.62 mmol) were dissolved in THF (22.5 mL) and DIPA (22.5 mL), then freeze-pump-thaw degassed (3×). After refreezing the solution and under a positive pressure of N_2, CuI (107 mg, 0.1 Eq, 562 μmol) and $Pd(PPh_3)_4$ (1.30 g, 0.2 Eq, 1.12 mmol) were added. The Schlenk tube was then evacuated/refilled with nitrogen (3×) and under a positive pressure of N_2, stirred at 80 °C for 16 h. The crude reaction mixture was cooled to 20 °C then diluted with CH_2Cl_2 (20 mL) and concentrated onto a minimum of silica gel. The desired compound **4** was obtained by flash column chromatography (SiO_2, 10% EtOAc in PE) as a viscous yellow oil (2.47 g, 85% yield).

^{1}H NMR (400 MHz, $CDCl_3$) δ_H = 8.78 (d, J = 2.2 Hz, 1H, **H_b**), 8.61 (dd, J = 4.9, 1.7 Hz, 1H, **H_a**), 7.92 (s, 2H, **H_e**), 7.83 (dt, J = 7.9, 1.9 Hz, 1H, **H_d**), 7.34–7.30 (m, 1H, **H_c**), 2.44 (t, J = 6.9 Hz, 2H, **H_j**), 1.90–1.79 (m, 2H, **H_i**), 1.14–1.07 (m, 14H, **H_g, H_f**), 0.92–0.82 (m, 2H, **H_h**) ppm.
^{19}F NMR (377 MHz $CDCl_3$) δ_F = –62.50 ppm (referenced against C_6F_6 (δ –161.64) as an internal standard)
^{13}C NMR (101 MHz, $CDCl_3$) δ_C = 152.43, 150.02, 138.78, 134.07 (q, J_{FC} = 31.1 Hz), 132.36 (q, J_{FC} = 5.2 Hz), 123.57, 123.28, 122.59 (q, J_{FC} = 275.7 Hz), 119.64, 119.22, 104.36, 100.09, 96.30, 84.0, 77.36, 21.26, 20.94, 18.25, 18.02, 11.66, 9.58 ppm.
HRMS (ESI$^+$) m/z: $[M+H]^+$ calcd. for $C_{27}H_{27}F_6N_2Si^+$ 521.1842; found 521.1835.

Synthesis of T2 (6)

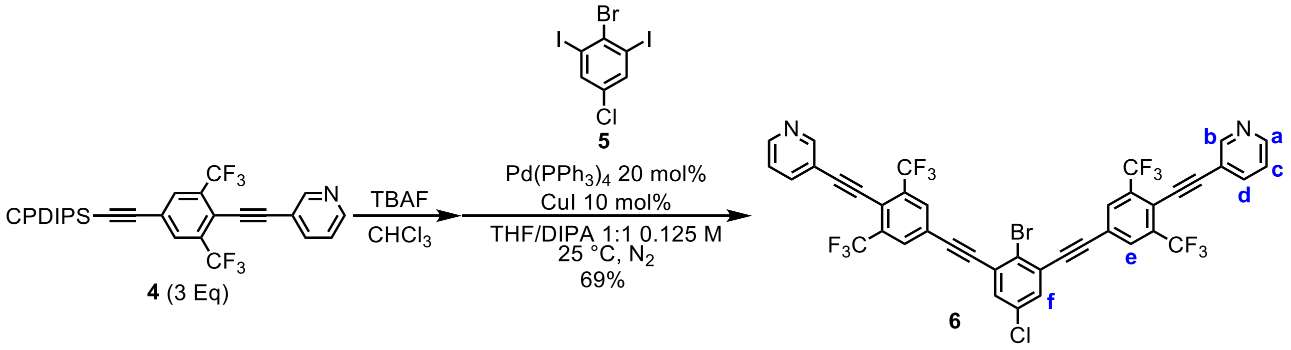

To a stirring solution of **4** (493 mg, 3 Eq, 948 µmol) in chloroform (10 mL) was added TBAF (1.10 mL, 1.0 M in THF, 3.5 Eq, 1.1 mmol). The solution was stirred for 5 min, after which TLC (30% EtOAc in PE) showed full deprotection of the CPDIPS group. The crude reaction mixture was then poured into a mixture of water (20 mL) and acetic acid (63 µL, 3.5 Eq, 1.1 mmol), extracted with chloroform, dried, and concentrated in vacuo.

In an oven dried Schlenk tube, deprotected **4** and 2-bromo-5-chloro-1,3-diiodobenzene (**5**) (140 mg, 1 Eq, 316 µmol) were dissolved in THF (6.32 mL) and DIPA (6.32 mL), then freeze-pump-thaw degassed (3×). After refreezing the solution and under a positive pressure of N_2, CuI (6.0 mg, 0.1 Eq, 31.6 µmol) and $Pd(PPh_3)_4$ (73 mg, 0.2 Eq, 63 µmol) were added. The Schlenk tube was then evacuated/refilled with nitrogen (3×) and under a positive pressure of N_2, stirred at 20 °C for 16 h.

The crude reaction mixture was concentrated in vacuo, then CH_2Cl_2 (10 mL) was added and the mixture sonicated for 5 min. The resulting suspension was filtered to obtain **6** as a fine white powder (188 mg, 69% yield).

^{1}H NMR (400 MHz, $CDCl_3$) δ_H = 8.82 (s, 2H, **H$_b$**), 8.65 (d, J = 4.9 Hz, 2H, **H$_a$**), 8.07 (s, 4H, **H$_e$**), 7.90–7.85 (m, 2H, **H$_d$**), 7.59 (s, 2H, **H$_f$**), 7.36 (dd, J = 7.9, 4.9 Hz, 2H, **H$_c$**) ppm.
^{19}F NMR (377 MHz $CDCl_3$) δ_F = –62.52 ppm (referenced against C_6F_6 (δ_F –161.64) as an internal standard).
^{13}C NMR - Solubility too poor to record spectrum
HRMS (ESI$^+$) m/z: [M+H]$^+$ calcd. for $C_{40}H_{15}BrClF_{12}N_2^+$ 864.9910; found 864.9896.

Synthesis of $T3_A$

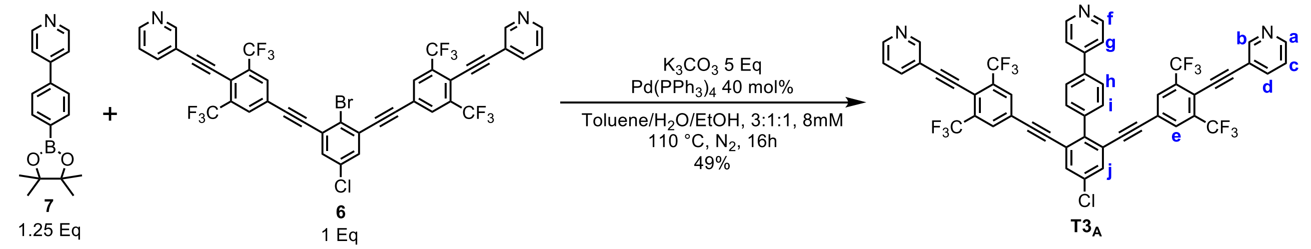

To an oven dried Schlenk tube was added **6** (300 mg, 1 Eq, 346 µmol), 4-(4-(4,4,5,5-tetramethyl-1,3,2-dioxaborolan-2-yl)phenyl)pyridine (**7**) (122 mg, 1.25 Eq, 433 µmol) and potassium carbonate (239 mg, 5 Eq, 1.73 mmol). The Schlenk was stoppered, evacuated/refilled with N_2 (5×), then toluene (26.0 mL), water (8.7 mL) and ethanol (8.7 mL) were added and N_2 bubbled through the resulting solution for 10 min. Under a positive flow of N_2, $Pd(PPh_3)_4$ (80 mg, 0.2 Eq, 69 µmol) was added. The Schlenk was then stoppered, purged with N_2 for 10 min and vigorously stirred at 110 °C.

After 3 h, the reaction was cooled to room temperature and under a positive flow of N_2, $Pd(PPh_3)_4$ (80 mg, 0.2 Eq, 69 µmol) was added. The Schlenk was then stoppered, purged with N_2 for 10 min then vigorously stirred at 110 °C for 16 h.

After cooling to room temperature, the crude reaction mixture was poured into a separating funnel and extracted with chloroform (3 × 20 mL). The combined organic phase was concentrated in vacuo, then purified by flash column chromatography (SiO_2, chloroform to 5% MeOH in chloroform) to yield **$T3_A$** as a fine white powder (160 mg, 49% yield).

^{1}H NMR (600 MHz, $C_2D_2Cl_4$, 373 K) δ_H = 8.83–8.80 (m, 2H, **H_b**), 8.76 (d, J = 5.1 Hz, 2H, **H_f**), 8.66 (dd, J = 4.9, 1.7 Hz, 2H, **H_a**), 7.88 (d, J = 8.2 Hz, 2H, **H_h/H_i**), 7.87–7.84 (m, 2H, **H_d**), 7.78 (s, 2H, **H_j**), 7.74 (d, J = 8.2 Hz, 2H, **H_h/H_i**), 7.70 (s, 4H, **H_e**), 7.62–7.59 (m, 2H, **H_g**), 7.35 (dd, J = 8.0, 4.9 Hz, 2H, **H_c**) ppm.
^{19}F NMR (377 MHz, $CDCl_3$, 298 K) δ_F = –62.65 ppm (referenced against C_6F_6 (δ_F –161.64) as an internal standard).
^{13}C NMR (151 MHz, $C_2D_2Cl_4$, 373 K) δ_C = 152.10, 150.21, 149.76, 147.15, 145.11, 133.95, 133.94 (q, J_{FC} = 31.1 Hz), 133.61, 132.59, 131.37 (q, J_{FC} = 5.3 Hz), 130.69, 126.17, 123.61, 122.97, 122.87, 122.28 (q, J_{FC} = 274.9 Hz) 121.17, 119.01, 118.88, 100.43, 92.03, 91.63, 83.86, 74.04 ppm.
HRMS (ESI$^+$) m/z: $[M+H]^+$ calcd. for $C_{51}H_{23}ClF_{12}N_3^+$ 940.1383; found 940.1363.

Synthesis of 8

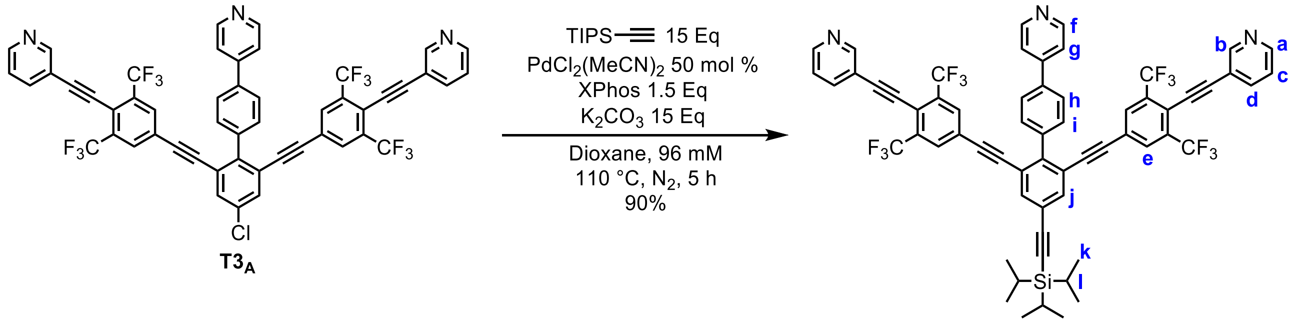

To an oven dried Schlenk tube was added **T3$_A$** (96.0 mg, 1 Eq, 102 µmol), $PdCl_2(MeCN)_2$ (13.2 mg, 0.5 Eq, 51 µmol), potassium carbonate (212.0 mg, 15 Eq, 1.53 mmol) and XPhos (73.0 mg, 1.5 Eq, 153 µmol). The Schlenk was then evacuated and refilled with N_2 (5×) before dioxane (1.06 mL) was added and the resulting solution bubbled with N_2 for 10 min. (Triisopropylsilyl)acetylene (344 µL, 15 Eq, 1.53 mmol) was then added and the reaction stirred at 110 °C for 5 h, after which ESI mass spectrometry showed no evidence of **T3$_A$** remaining and a clear mass peak for **8**. The reaction mixture was allowed to cool to 20 °C, concentrated, then purified by column chromatography (SiO_2, chloroform to chloroform + 1% MeOH) to yield **S10** as a white solid (99.2 mg, 90%).

^{1}H NMR (400 MHz, $CDCl_3$) δ_H = 8.77 (dd, J = 2.1, 0.9 Hz, 2H, **H$_b$**), 8.76–8.73 (m, 2H, **H$_f$**), 8.63 (dd, J = 4.9, 1.7 Hz, 2H, **H$_a$**), 7.87–7.80 (m, 6H, **H$_d$, H$_{h/i}$, H$_j$**), 7.74 – 7.70 (m, 2H, **H$_{h/i}$**), 7.67 (s, 4H, **H$_e$**), 7.60–7.57 (m, 2H, **H$_g$**), 7.33 (ddd, J = 7.9, 4.9, 1.0 Hz, 2H, **H$_c$**), 1.19–1.16 (m, 21H, **H$_k$, H$_l$**) ppm.
^{19}F NMR (377 MHz $CDCl_3$) δ_F = –62.63 ppm (referenced against C_6F_6 (δ_F = –161.64) as an internal standard).
^{13}C NMR (101 MHz, $CDCl_3$) δ_F 152.45, 150.56, 150.08, 145.99, 138.82, 136.43, 134.12 (q, J_{FC} = 31.1 Hz), 131.61 (q, J_{FC} = 5.2 Hz), 131.15, 126.42, 124.03, 123.31, 122.55, 122.53 (q, J_{FC} = 275.7 Hz) 121.65, 119.20, 119.08, 104.29, 100.32, 94.88, 94.22, 92.70, 91.09, 84.07, 18.81, 18.57, 11.40, 11.14 ppm.
HRMS (ESI$^+$) m/z: $[M+H]^+$ calcd. for $C_{62}H_{44}F_{12}N_3Si^+$ 1086.3107; found 1086.3085.

Synthesis of 7-(4-bromo-2,5-dioctylphenyl)-5,5-diisopropylhept-6-ynenitrile (S5)

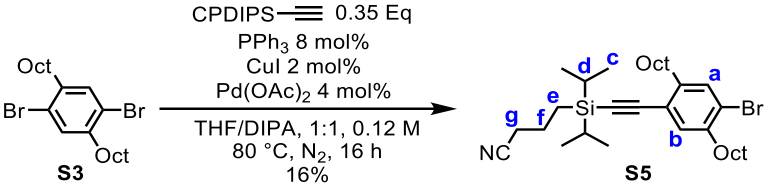

To an oven dried Schlenk tube was added **S3** (1.95 g, 1 Eq, 4.24 mmol), $Pd(OAc)_2$ (38 mg, 0.04 Eq, 169 µmol), PPh_3 (89 mg, 0.08 Eq, 339 µmol) and CuI (16 mg, 0.02 Eq, 85 µmol). The tube was then stoppered and evacuated/refilled with N_2 (5×), before a freeze-pump-thaw degassed mixture of THF (17 mL) and DIPA (17 mL) was added via syringe. 4-(Ethynyldiisopropylsilyl)butanenitrile (341 µL, 0.35 Eq, 1.48 mmol) was then added via syringe and the mixture stirred at 80 °C for 16 h. After cooling to room temperature, the crude reaction mixture was loaded onto a minimum of silica gel then purified by flash column chromatography (PE to 20% CH_2Cl_2 in PE) to yield the **S5** as a yellow oil (405 mg, 16% yield).

^{1}H NMR (400 MHz, $CDCl_3$) δ_H = 7.35 (s, 1H, **H$_a$/H$_b$**), 7.26 (s, 1H, **H$_a$/H$_b$**), 2.72–2.61 (m, 4H, Ar-**CH$_2$**), 2.42 (t, J = 6.9 Hz, 2H, **H$_g$**), 1.91–1.82 (m, 2H, **H$_f$**), 1.64–1.55 (m, 4H, Ar-CH$_2$-**CH$_2$**), 1.38–1.22 (m, 30H, Ar-CH$_2$-**(CH$_2$)$_6$**-CH$_3$), 1.14–1.05 (m, 14H, **H$_c$/H$_d$**), 0.91–0.80 (m, 8H, Ar-(CH$_2$)$_7$-**CH$_3$**, **H$_e$**) ppm.

Synthesis of 7-(4-((4-(3,3-diethyltriaz-1-en-1-yl)phenyl)ethynyl)-2,5-dioctylphenyl)-5,5-diisopropylhept-6-ynenitrile (15)

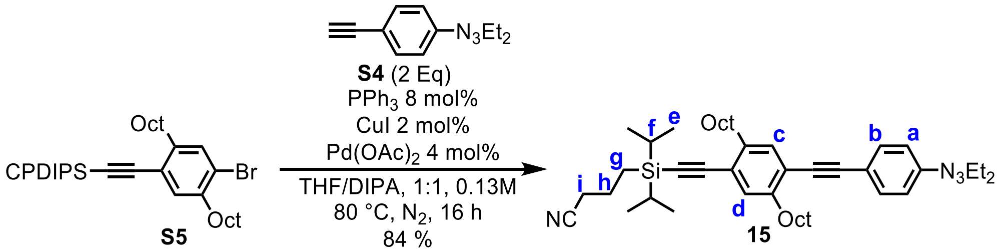

In an oven dried Schlenk tube, **S5** (237 mg, 2 Eq, 1.18 mmol) and **S4** (345 mg, 1 Eq, 588 µmol) were dissolved in THF (2.35 mL) and DIPA (2.35 mL), then freeze-pump-thaw degassed (3×). After refreezing the solution and under a positive pressure of N_2, $Pd(OAc)_2$ (5.28 mg, 0.04 Eq, 23.5 µmol), PPh_3 (12.3 mg, 0.08 Eq, 47.0 µmol) and CuI (2.24 mg, 0.02 Eq, 11.8 µmol) were added. The Schlenk was stoppered and evacuated/refilled with N_2 (5×) then stirred at 80 °C for 16 h. After cooling to room temperature, the crude reaction mixture was concentrated onto a minimum of silica gel then purified by flash column chromatography (SiO_2, PE to 5% EtOAc in PE) to yield **15** as a yellow oil (350 mg, 84%).

^{1}H NMR (400 MHz, $CDCl_3$) δ_H = 7.50–7.45 (m, 2H, **H$_a$/H$_b$**), 7.43–7.38 (m, 2H, **H$_a$/H$_b$**), 7.31 (s, 1H, **H$_c$/H$_d$**), 7.28 (s, 1H, **H$_c$/H$_d$**), 3.79 (q, J = 7.2 Hz, 4H, N-**CH$_2$**), 2.75 (m, 4H, Ar-**CH$_2$**), 2.43 (t, J = 6.9 Hz, 2H, **H$_i$**), 1.94 – 1.82 (m, 2H, **H$_h$**), 1.74–1.57 (m, 2H, Ar-CH$_2$-**CH$_2$**), 1.43–1.22 (m, 30H, Ar-CH$_2$-**(CH$_2$)$_6$**-CH$_3$, N-CH$_2$-**CH$_3$**), 1.16–1.06 (m, 14H, **H$_e$/H$_f$**), 0.91–0.80 (m, 8H, Ar-(CH$_2$)$_7$-**CH$_3$**, **H$_g$**) ppm.
^{13}C NMR (101 MHz, $CDCl_3$) δ_C = 151.22, 142.66, 142.21, 133.00, 132.28, 132.24, 123.48, 121.92, 120.55, 119.80, 119.63, 106.84, 94.98, 93.76, 88.19, 53.54, 34.49, 34.33, 32.03, 30.95, 30.91, 29.80, 29.71, 29.63, 29.44, 22.79, 21.47, 20.94, 18.36, 18.13, 14.23, 11.93, 9.86, 1.14 ppm.
HRMS (ESI$^+$) m/z: [M+H]$^+$ calcd. for $C_{46}H_{71}N_4Si^+$ 707.5443; found 707.5430.

Synthesis of Extended Core (17)

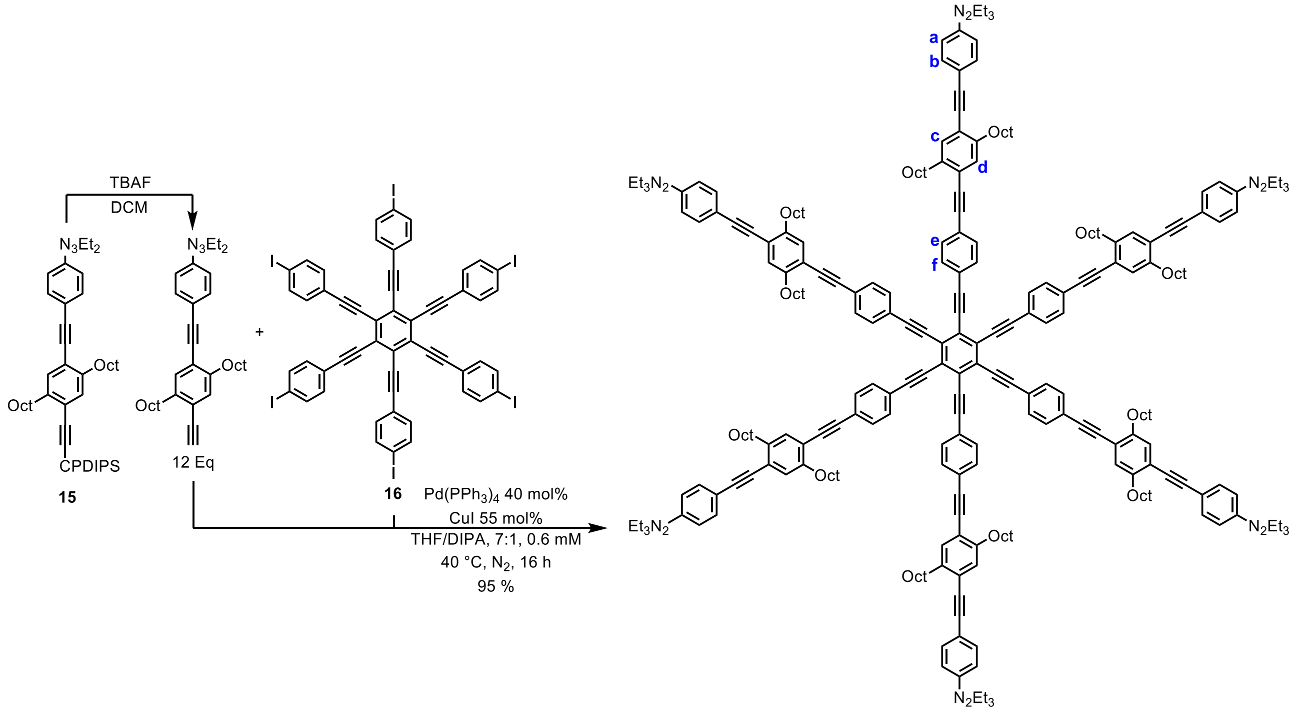

To a stirring solution of **15** (284 mg, 12 Eq, 402 µmol) in CH_2Cl_2 (2 mL) was added TBAF (803 µL, 1.00 M in THF, 24 Eq, 803 µmol). The solution was stirred for 15 min then CH_2Cl_2 (10 mL), water (25 mL) and acetic acid (100 µL) were added. The mixture was then poured into a separating funnel and extracted with chloroform (3 × 10 mL). The organic phase was dried with $MgSO_4$, then concentrated in vacuo and used in the next step without further purification.

In an oven dried Schlenk tube, deprotected **15** from the first step and **16** (48 mg, 1 Eq, 33.5 µmol) were dissolved in THF (48.8 mL) and DIPA (6.97 mL), then freeze-pump-thaw degassed (3×). After refreezing the solution and under a positive pressure of N_2, CuI (3.51 mg, 0.55 Eq, 18.4 µmol) and $Pd(PPh_3)_4$ (15.5 mg, 0.4 Eq, 13.4 µmol) were added. The Schlenk tube was stoppered, evacuated/refilled with N_2 (5×), then stirred at 40 °C for 16 h.

After cooling to room temperature, the crude reaction mixture was concentrated in vacuo then purified by flash column chromatography (SiO_2, 5% EtOAc in PE to elute unreacted starting material and Glaser coupling products, then 100% EtOAc to elute the desired product). The purified material was then passed through a size exclusion column (SX3 biobeads, $CHCl_3$) and concentrated to yield **17** as a yellow film (121.9 mg, 95% yield).

^{1}H NMR (500 MHz, $CDCl_3$) δ_H = 7.65 (d, J = 8.3 Hz, 12H, **H_a/H_b/H_e/H_f**), 7.57 (d, J = 8.3 Hz, 12H, **H_a/H_b/H_e/H_f**), 7.51 (d, J = 8.5 Hz, 12H, **H_a/H_b/H_e/H_f**), 7.43 (d, J = 8.5 Hz, 12H, **H_a/H_b/H_e/H_f**), 7.40 (s, 6H, **H_c/H_d**), 7.39 (s, 6H, **H_c/H_d**), 3.80 (q, J = 7.2 Hz, 24H, N-**CH_2**-CH_3), 2.88–2.80 (m, 24H, Ar-**CH_2**), 1.79–1.68 (m, 24H, Ar-CH_2-**CH_2**), 1.48–1.21 (m, 156H, Ar-CH_2-CH_2-**$(CH_2)_5$**-CH_3, N-CH_2-**CH_3**), 0.91–0.83 (m, 36H, Ar-$(CH_2)_2$-**CH_3**) ppm.
^{13}C NMR (126 MHz, $CDCl_3$) δ_C = 151.23, 142.44, 142.31, 132.52, 132.36, 131.93, 131.74, 127.63, 124.55, 123.49, 122.74, 121.95, 120.59, 119.77, 99.71, 95.11, 93.57, 91.45, 89.17, 88.37, 34.36, 34.28, 32.07, 30.83, 30.80, 29.79, 29.69, 29.67, 29.48, 29.42, 22.83, 22.81, 14.27 ppm.

Synthesis of Iodinated Extended Core (18)

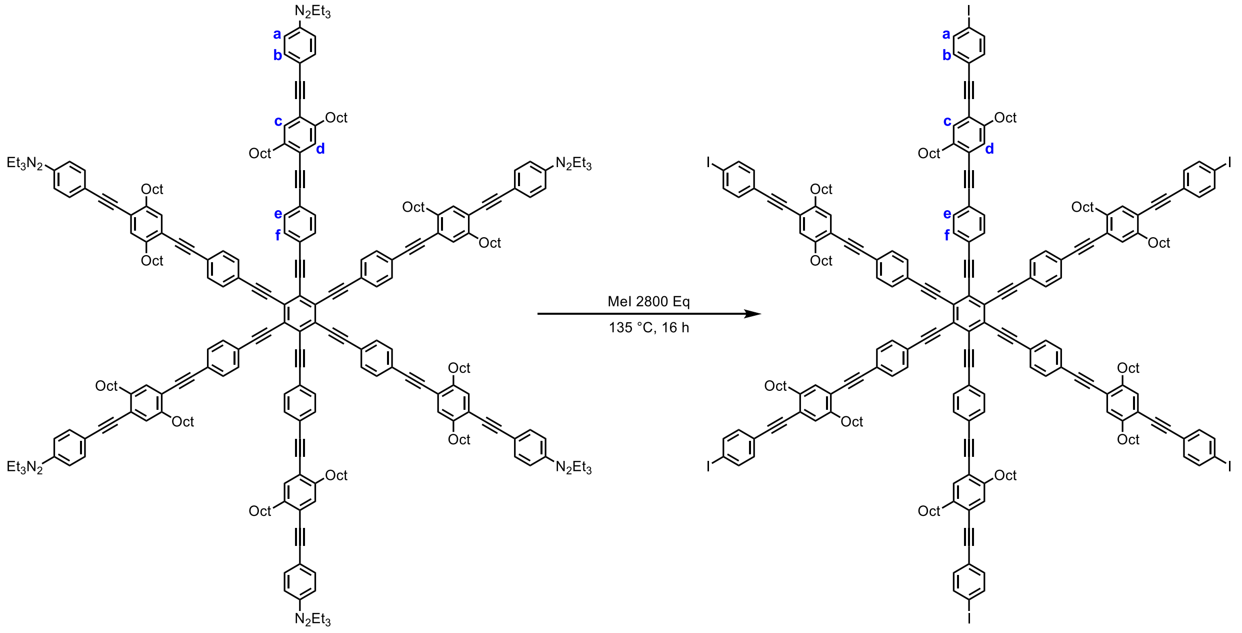

17 **18**

To an oven dried pressure tube was added **17** (109 mg, 1 Eq, 28.9 µmol) and MeI (5.0 mL, 2800 Eq, 80 mmol). The pressure tube was sealed, then stirred at 135 °C for 16 h. After cooling to room temperature, the MeI was removed by a stream of nitrogen, then the crude reaction mixture was purified on a size exclusion column (SX3 biobeads, $CHCl_3$) and concentrated in vacuo to yield **18** as a yellow solid (75 mg, 66%).

^{1}H NMR (500 MHz, $CDCl_3$) δ_H = 7.70 (d, J = 8.4 Hz, 12H, **H_b/H_e/H_f**), 7.63 (d, J = 8.4 Hz, 12H, **H_b/H_e/H_f**), 7.55 (d, J = 8.4 Hz, 12H, **H_b/H_e/H_f**), 7.38 (s, 6H, **H_c/H_d**), 7.36 (s, 6H, **H_c/H_d**), 7.24 (d, J = 8.4 Hz, 12H, **H_a**), 2.84–2.77 (m, 24H, Ar-**CH_2**), 1.74–1.64 (m, 24H, Ar-CH_2-**CH_2**), 1.44–1.20 (m, 120H, Ar-CH_2-CH_2-**$(CH_2)_5$**-CH_3), 0.85 (m, 36H, Ar-$(CH_2)_2$-**CH_3**) ppm.
^{13}C NMR (126 MHz, CDCl3) δ_C = 142.36, 142.34, 137.60, 132.94, 132.40, 132.29, 131.77, 131.55, 127.48, 124.20, 122.98, 122.82, 122.53, 122.49, 99.48, 94.13, 93.77, 93.19, 91.12, 89.92, 89.32, 34.20, 34.14, 31.96, 31.94, 30.72, 30.62, 29.68, 29.60, 29.56, 29.37, 29.33, 22.72, 22.70, 14.18, 14.16 ppm.

Synthesis of template T18$_A$

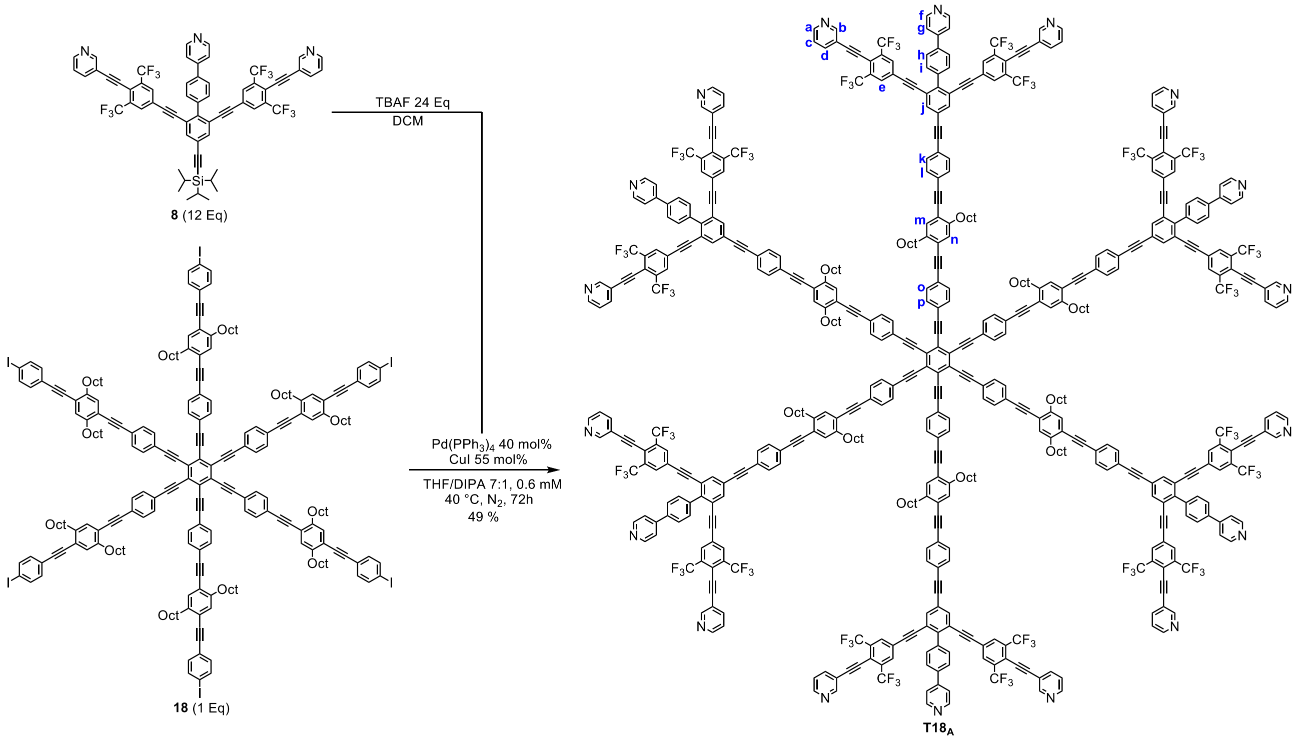

To a stirring solution of **8** (62.1 mg, 12 Eq, 57.3 μmol) in CH_2Cl_2 (2 mL) was added TBAF (115 μL, 1.0 M in THF, 24 Eq, 115 μmol). The solution was stirred for 15 min then CH_2Cl_2 (2mL), water (5 mL) and acetic acid (20 μL) were added. The mixture was then poured into a separating funnel and extracted with chloroform (3 × 10 mL). The organic phase was dried with $MgSO_4$, then concentrated in vacuo and used in the next step without further purification.

In an oven dried Schlenk tube, deprotected **8** and **18** (19.0 mg, 1 Eq, 4.77 μmol) were dissolved in THF (6.96 mL) and DIPA (994 μL), then freeze-pump-thaw degassed (5×). After refreezing the solution and under a positive pressure of N_2, CuI (0.50 mg, 0.55 Eq, 2.62 μmol) and $Pd(PPh_3)_4$ (2.21 mg, 0.4 Eq, 1.91 μmol) were added. The Schlenk tube was stoppered, evacuated/refilled with N_2 (5×), then stirred at 40 °C for 72 h. The crude reaction mixture was allowed to cool, then transferred into a vial and centrifuged. The brown supernatant solution was pipetted off, reduced to half its volume and centrifuged again. The bright yellow precipitate collected from both centrifugations was combined then suspended in MeOH (10 mL), sonicated and centrifuged. This process was repeated three times before the yellow solid was dried under a high vacuum to yield exceptionally clean **T18$_A$** (9.33 mg, 22% yield). The remaining brown supernatant was concentrated in vacuo then purified by flash column chromatography (SiO_2, 10% MeOH in $CHCl_3$ to 15% MeOH + 1% *t*-$BuNH_2$ in $CHCl_3$) to yield additional **T18$_A$** (11.18 mg, 27% yield). Total yield of **T18$_A$** (20.51 mg, 49% yield).

* Both the precipitated and columned **T18$_A$** show no evidence of incomplete coupling, however the VT NMR of the precipitated material is sharper and TLC (10% MeOH, 0.5% $^tBuNH_2$ in $CHCl_3$) cleaner than the columned material *

^{1}H NMR (600 MHz, $C_2D_2Cl_4$, 393 K) δ_H = 8.20–8.16 (m, 12H, **H$_b$**), 8.15–8.12 (m, 12H, **H$_f$**), 8.04–8.00 (m, 12H, **H$_a$**), 7.32 (s, 12H, **H$_j$**), 7.27–7.23 (m, 12H, **H$_h$**), 7.22–7.19 (m, 12H, **H$_d$**), 7.14 (d, *J* = 8.4 Hz, 12H, **H$_i$**), 7.10 – 7.06 (m, 36H, **H$_e$**, **H$_{k/l/o/p}$**), 7.02–6.96 (m, 48H, **H$_g$**, **H$_{k/l/o/p}$**), 6.85–6.81 (m, 12H, **H$_m$**, **H$_n$**), 6.72–6.69 (m, 12H, **H$_c$**), 2.33–2.26 (m, 24H, Ar-**CH$_2$**), 1.23–1.17 (m, 24H, Ar-CH$_2$-**CH$_2$**), 0.94–0.69 (m, 120H Ar-(CH$_2$)$_2$-**(CH$_2$)$_5$**-CH$_3$), 0.32 (dt, *J* = 14.1, 6.8 Hz, 36H, Ar-(CH$_2$)$_7$-**CH$_3$**) ppm.

^{19}F NMR (470 MHz, $CDCl_3$, 298 K) δ_F = –65.64 ppm (referenced against C_6F_6 (δ_F = –161.64) as an internal standard).
^{13}C NMR – (151 MHz, $C_2D_2Cl_4$, 393 K) δ_C = 152.09, 149.94, 149.72, 146.09, 142.29, 138.73, 138.57, 138.12, 135.60, 134.00 (q, J_{FC} = 30.2 Hz), 132.34, 131.58, 131.34, 131.32 (Expected to be CF_3), 130.66, 127.58, 126.11, 124.39, 124.19, 123.61, 122.78, 122.33 (q, J_{FC} = 274.8 Hz), 122.65, 122.16, 121.18, 120.23, 118.90,100.42, 99.72, 93.79, 93.69, 92.62, 91.63, 91.28, 91.18, 91.10, 89.12, 88.72, 83.88, 33.95, 31.58, 30.29, 30.25, 29.33, 29.28, 29.19, 28.93, 28.90, 27.75, 22.28, 13.62.
MALDI-ToF MS *m/z* 8799.827 (calculated for ($[C_{564}H_{396}F_{72}N_{18}]^{\bullet+}$) = **(M)**$^{\bullet+}$: 8788.046).

Synthesis of 9

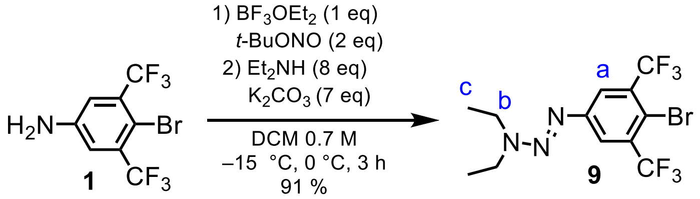

To an oven-dried reaction vessel was added 4-bromo-3,5-bis(trifluoromethyl)aniline (3.00 g, 1 Eq, 9.74 mmol) in DCM (40 mL) and the solution was cooled to –15 °C using dry ice in acetone. Boron trifluoride etherate (2.47 mL, 2 Eq, 19.5 mmol) and *tert*-butyl nitrite (2.32 mL, 2 Eq, 19.5 mmol) in DCM (5 mL) were added and the mixture was stirred for 30 min at –15 °C. The mixture was warmed to 0 °C and stirred for 30 min. Diethylamine (8.06 mL, 8 Eq, 77.9 mmol) and potassium carbonate (9.42 g, 7 Eq, 68.2 mmol) were added sequentially and the mixture was stirred for 2 h at 0 °C. The reaction mixture was poured into water and extracted with ethyl acetate (3 × 50 mL). The organic layers were dried using $MgSO_4$ and concentrated under reduced pressure. The mixture was filtered through a SiO_2 plug (DCM / PE 1:9) to yield a brown solid (3.50 g, 91%).

^{1}H NMR (600 MHz, $CDCl_3$) δ_H = 7.89 (s, 2H, $\mathbf{H_a}$), 3.81 (q, J = 6.1 Hz, 4H, $\mathbf{H_b}$), 1.36 (t, J = 6.1 Hz, 3H, $\mathbf{H_c}$), 1.23 (t, J = 6.1 Hz, 3H, $\mathbf{H_c}$) ppm.
^{19}F NMR (564 MHz $CDCl_3$) δ_F = –61.66 ppm (referenced against C_6F_6 (δ_F –161.64) as an internal standard).
^{13}C NMR (151 MHz, $CDCl_3$) δ_C = 150.59, 133.06 (q, J_{FC} = 32.3 Hz), 122.66 (q, J_{FC} = 6.0 Hz), 122.60 (q, J_{FC} = 274.8 Hz), 112.39, 49.72, 41.92, 14.52, 11.18 ppm.
HRMS (ESI$^+$) *m/z*: $[M+H]^+$ calcd. for $C_{12}H_{13}BrF_6N_3^+$ 392.0192; found 392.0187.

Synthesis of 10

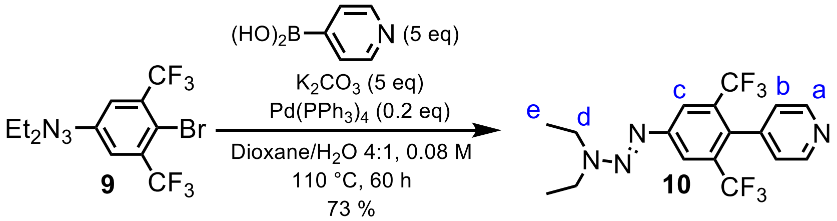

(*E*)-1-(4-Bromo-3,5-bis(trifluoromethyl)phenyl)-3,3-diethyltriaz-1-ene (1.00 g, 1 Eq, 2.55 mmol), pyridin-4-ylboronic acid (1.57 g, 5 Eq, 12.8 mmol) and potassium carbonate (1.76 g, 5 Eq, 12.8 mmol) were added to a Schlenk tube. The tube was evacuated and backfilled with argon. 1,4-Dioxane (25.00 mL) and water (6.25 mL) were added and the mixture was degassed performing 3 cycles of freeze-pump-thaw. $Pd(PPh_3)_4$ (589 mg, 0.2 Eq, 510 µmol), was added and the mixture was refluxed at 110 °C under argon for 2.5 days. The mixture was dissolved in chloroform, washed with water (2 × 50 mL), dried with $MgSO_4$ and concentrated under reduced pressure. Purification by flash column chromatography (EtOAc / PE 1:9) yielded a white solid (719 mg, 73%).

^{1}**H NMR** (600 MHz, $CDCl_3$) δ_H = 8.64 (d, J = 6.1 Hz, 2H, **Ha**), 7.97 (s, 2H, **Hc**), 7.21 (d, J = 6.1 Hz, 2H, **Hb**), 3.84 (q, J = 7.2, 4H, **Hd**), 1.38 (t, J = 6.5 Hz, 3H, **He**), 1.26 (t, J = 7.1 Hz, 3H, **He**) ppm.
^{19}**F NMR** (564 MHz $CDCl_3$) δ_F = –57.23 ppm (referenced against C_6F_6 (δ_F –161.64) as an internal standard).
^{13}**C NMR** (151 MHz, $CDCl_3$) δ_C = 151.37, 148.58, 143.25, 131.80, 131.2 (q, J_{FC} = 30.2 Hz), 125.42, 123.17 (q, J_{FC} = 274.8 Hz), 120.88 (q, J_{FC} = 4.5 Hz), 49.72, 41.92, 14.52, 11.18 ppm.
HRMS (ESI^+) *m/z*: $[M+H]^+$ calcd. for $C_{17}H_{17}F_6N_4^+$ 391.1352; found 391.1362.

Synthesis of 11

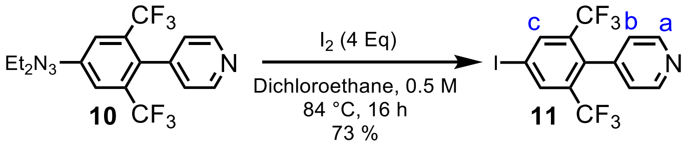

(E)-4-(4-(3,3-Diethyltriaz-1-en-1-yl)-2,6-bis(trifluoromethyl)phenyl)pyridine (600 mg, 1 Eq, 1.54 mmol), I_2 (1.56 g, 4 Eq, 6.15 mmol) and 1,2-dichloroethane (6.00 mL) were added to a Schlenk tube. The reaction mixture was degassed by 3 cycles of freeze-pump-thaw and the tube was backfilled with argon. The reaction mixture was heated to 84 °C and stirred for 16 h It was then allowed to cool to room temperature and washed with a sat. $Na_2S_2O_3$ (3 × 100 mL), then with saturated Na_2CO_3 (2 × 100 mL) and water (2 × 100 mL). The reaction mixture was then dried and evaporated at reduced pressure. Purification by flash column chromatography (EtOAc / PE 1:9) yielded a white solid (467 mg, 73%).

^{1}**H NMR** (600 MHz, $CDCl_3$) δ_H = 8.66 (m, 2H, **Ha**), 8.29 (s, 2H, **Hc**), 7.18 (d, J = 8.8 Hz, 2H **Hb**) ppm.
^{19}**F NMR** (564 MHz $CDCl_3$) δ_F = 57.43 ppm (referenced against C_6F_6 (δ_F –161.64) as an internal standard).
^{13}**C NMR** (151 MHz, $CDCl_3$) δ_C = 148.94, 141.77, 138.50 (q, J_{FC} = 3.0 Hz), 136.62, 132.28 (q, J_{FC} = 30.2 Hz), 124.76, 121.97 (q, J_{FC} = 276.3 Hz), 93.40 ppm.
HRMS (ESI^+) *m/z*: $[M+H]^+$ calcd. for $C_{13}H_7F_6IN^+$ 417.9522; found 417.9506.

Synthesis of 12

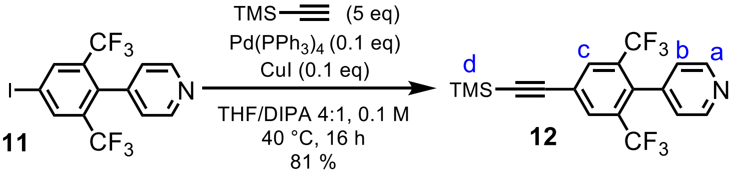

4-(4-Iodo-2,6-bis(trifluoromethyl)phenyl)pyridine (1.5 g, 1 Eq, 3.6 mmol), CuI (69 mg, 0.1 Eq, 360 μmol) were added to a Schlenk tube. The tube was filled with argon and evacuated 3 times. THF (24 mL) and diisopropylamine (6 mL) were added and the mixture was degassed by performing 3 cycles of freeze/pump/thaw. $Pd(PPh_3)_4$ (623 mg, 0.15 Eq, 539 μmol) and trimethylsilyl acetylene (2.5 mL, 5 Eq, 18 mmol) were added to the frozen mixture, and the resulting mixture was stirred for 16 h at 40 °C. The reaction mixture was diluted with dichloromethane and washed with water (3 × 50 mL). Purification by flash column chromatography (EtOAc / PE 1:9) yielded a white solid (1.13 g, 81%).

^{1}**H NMR** (600 MHz, $CDCl_3$) δ_H = 8.68 (br s, 2H, **Ha**), 8.02 (s, 2H, **Hc**), 7.18 (br s, 2H, **Hb**), 0.29 (s, 9 H, **Hd**) ppm.
^{19}**F NMR** (564 MHz $CDCl_3$) δ_F = 57.50 ppm (referenced against C_6F_6 (δ_F –161.64) as an internal standard).
^{13}**C NMR** (151 MHz, $CDCl_3$) δ_C = 148.88, 142.22, 136.43, 132.58 (q, J_{FC} = 4.5 Hz), 131.25 (q, J_{FC} = 30.2 Hz), 124.96, 124.89, 122.7 (q, J_{FC} = 276.33 Hz), 101.40, 99.70, –0.19 ppm.
HRMS (ESI^+) *m/z*: $[M+H]^+$ calcd. for $C_{18}H_{16}F_6NSi^+$ 388.0951; found 388.3949.

Synthesis of 13

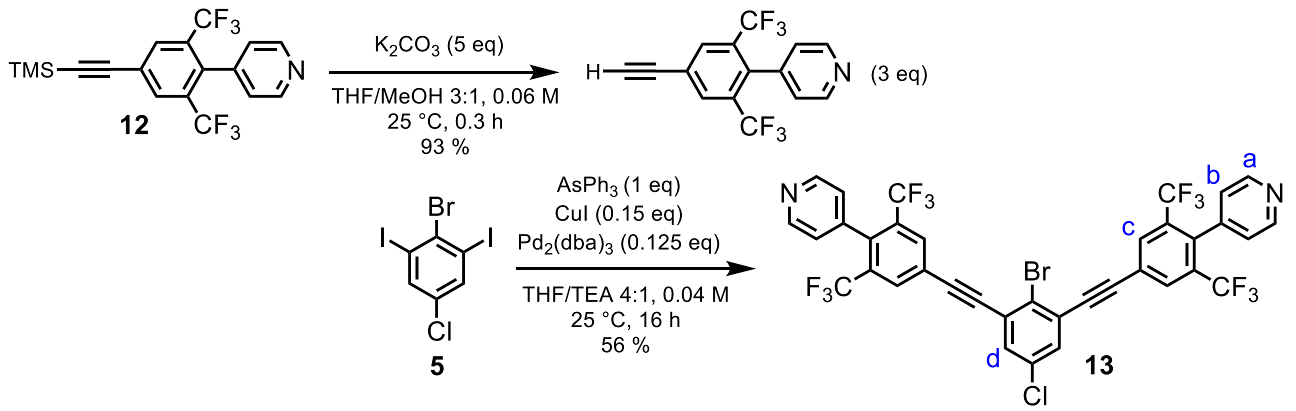

4-(2,6-Bis(trifluoromethyl)-4-((trimethylsilyl)ethynyl)phenyl)pyridine (500 mg, 1 Eq, 1.291 mmol) was dissolved in methanol (5 mL) and THF (15 mL). K_2CO_3 (892 mg, 5 Eq, 6.45 mmol) was added and the reaction mixture was stirred at room temperature. Progress was monitored by TLC in 10% EtOAc in PE and deprotection was complete after 15 min. The reaction mixture was diluted with 20 mL $CHCl_3$, washed with water (3 × 30 mL) and dried over $MgSO_4$ and concentrated under reduced pressure to yield a black solid (93%, 380 mg).

4-(4-Ethynyl-2,6-bis(trifluoromethyl)phenyl)pyridine (186 mg, 3 Eq, 590 µmol), 2-bromo-5-chloro-1,3-diiodobenzene (87 mg, 1 Eq, 197 µmol), triphenylarsine (60 mg, 1 Eq, 197 µmol) and CuI (6.0 mg, 0.15 Eq, 30 µmol) were added to an oven-dried Schlenk tube and evacuated 3 times. A solution of THF (13.0 mL) and triethylamine (3.25 mL) was degassed by performing 3 cycles of freeze-pump-thaw and then added via cannula. $Pd_2(dba)_3$ (22 mg, 0.13 eq, 25 µmol) was added to the frozen mixture and the reaction mixture was stirred vigorously for 16 h. The reaction mixture was diluted with chloroform and washed with water (3 × 30 mL). The organic phase was dried over $MgSO_4$, then filtered and concentrated under reduced pressure. Purification by flash column chromatography (5% EtOAc/$CHCl_3$) yielded a yellow solid (104 mg, 56%).

^{1}H NMR (600 MHz, $CDCl_3$) δ_H = 8.69 (d, J = 5.8 Hz, 4H, **H$_a$**), 8.16 (s, 4H, **H$_c$**), 7.62 (s, 2H, **H$_d$**), 7.22 (d, J = 5.8 Hz, 4H, **H$_b$**) ppm.
^{19}F NMR (564 MHz $CDCl_3$) δ_F = 57.51 ppm (referenced against C_6F_6 (δ_F −161.64) as an internal standard).
^{13}C NMR (151 MHz, $CDCl_3$) δ_C = 148.98, 141.97, 137.34, 133.64, 132.43 (q, J_{FC} = 4.5 Hz), 131.71 (q, J_{FC} = 30.2 Hz), 127.22, 126.80, 124.81, 124.69, 123.88, 122.63 (q, J_{FC} = 276.3 Hz), 92.02, 90.12 ppm.
HRMS (ESI$^+$) m/z: [M+H]$^+$ calcd. for $C_{36}H_{15}BrClF_{12}N_2^+$ 816.9910; found 816.9937.

Synthesis of T3$_B$

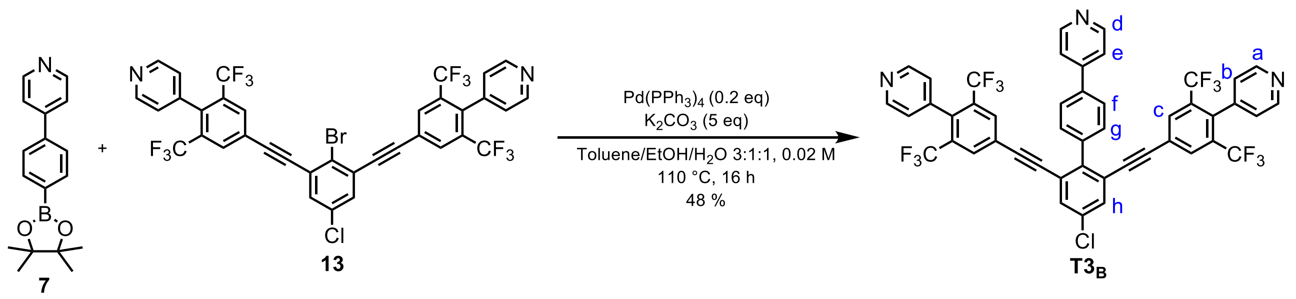

To an oven-dried Schlenk tube were added 4,4'-(((2-bromo-5-chloro-1,3-phenylene)bis(ethyne-2,1-diyl))bis(2,6-bis(trifluoromethyl)-4,1-phenylene))dipyridine (125 mg, 1 Eq, 153 µmol), 4-(4-(4,4,5,5-tetramethyl-1,3,2-dioxaborolan-2-yl)phenyl)pyridine (65 mg, 1.5 Eq, 229 µmol), K_2CO_3 (106 mg, 5 Eq, 764 µmol). Toluene (6 mL), water (2 mL) and ethanol (2 mL) were added and the reaction mixture was degassed performing 3 cycles of freeze-pump-thaw. $Pd(PPh_3)_4$ (35 mg, 0.2 Eq, 31 µmol) was added to the frozen mixture and the reaction mixture was refluxed at 110 °C for 16 h. The mixture was cooled to room

temperature and diluted in chloroform, washed with water (3 × 30 mL), dried over $MgSO_4$ and evaporated. Purification by flash column chromatography (3.5% MeOH in $CHCl_3$) yielded a brown solid (65 mg, 48%).

^{1}H NMR (600 MHz, $CDCl_3$) δ_H = 8.68 (bs, 6H, **H_{a+d}**), 7.84 (d, J = 8.3 Hz, 2H, **H_f**), 7.76 (s, 4H, **H_c**), 7.74 (s, 2H, **H_h**), 7.73 (d, J = 8.3 Hz, 2H, **H_g**), 7.57 (bs, 2H, **H_e**), 7.15 (bs, 4H, **H_b**) ppm.
^{19}F NMR (564 MHz $CDCl_3$) δ_F = 57.59 ppm (referenced against C_6F_6 (δ_F −161.64) as an internal standard).
^{13}C NMR (151 MHz, $CDCl_3$) δ_C = 150.58, 148.93, 147.36, 145.12, 141.86, 138.42, 136.88, 133.88, 133.04, 131.97 (q, J_{FC} = 6.0 Hz), 131.50 (q, J_{FC} = 30.2 Hz), 131.15, 129.89, 126.50, 124.82, 124.04, 123.80, 122.54 (q, J_{FC} = 274.8 Hz), 119.18, 91.21, 90.93 ppm.
HRMS (ESI$^+$) *m/z*: $[M+H]^+$ calcd. for $C_{47}H_{23}ClF_{12}N_3^+$ 892.1383; found 892.1451.

Synthesis of 14

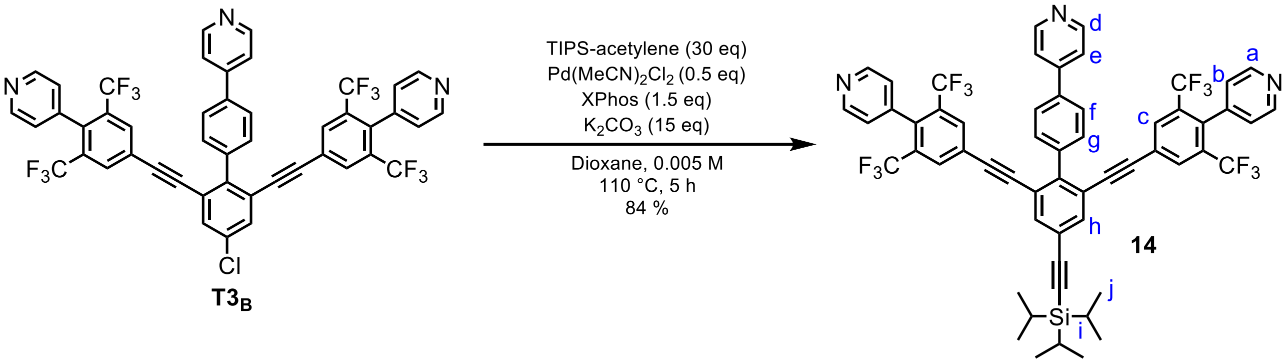

An oven-dried Schlenk tube was evacuated and refilled with N_2 three times. 4'-(((4-Chloro-4'-(pyridine-4-yl)-[1,1'-biphenyl]-2,6-diyl)bis(ethyne-2,1-diyl))bis(2,6-bis(trifluoromethyl)-4,1-phenylene))dipyridine (51 mg, 1 Eq, 57 µmol), XPhos (41 mg, 1.5 Eq, 86 µmol), $Pd(MeCN)_2Cl_2$ (11 mg, 0.5 Eq, 29 µmol) and K_2CO_3 (119 mg, 15 Eq, 857 µmol) were added and the tube was evacuated 3 times. Dry 1,4-dioxane (11.4 mL) was added and the reaction mixture was stirred at room temperature for 15 min. Triisopropylsilyl acetylene (385 µL, 30 Eq, 1.71 mmol) was added and the mixture was refluxed at 110 °C for 5 h. The reaction mixture was cooled to room temperature, diluted with $CHCl_3$ and filtered through a SiO_2 plug (10% MeOH in $CDCl_3$) and concentrated under reduced pressure. Purification by flash column chromatography (5% MeOH in $CDCl_3$) yielded a black solid (50 mg, 84%).

^{1}H NMR (600 MHz, $CDCl_3$) δ_H = 8.67 (bs, 6H, **H_{a+d}**), 7.84 (d, J = 8.1 Hz, 2H, **H_f**), 7.84 (s, 2H, **H_h**), 7.77 (s, 4H, **H_c**), 7.75 (d, J = 8.1 Hz, 2H, **H_g**), 7.57 (bs, 2H, **H_e**), 7.16 (bs, 4H, **H_b**) ppm.
^{19}F NMR (564 MHz $CDCl_3$) δ_F = 57.60 ppm (referenced against C_6F_6 (δ −161.64) as an internal standard).
HRMS (ESI$^+$) *m/z*: $[M+H]^+$ calcd. for $C_{58}H_{44}F_{12}N_3Si^+$ 1038.3107; found 1038.3171.

Synthesis of template T18$_B$

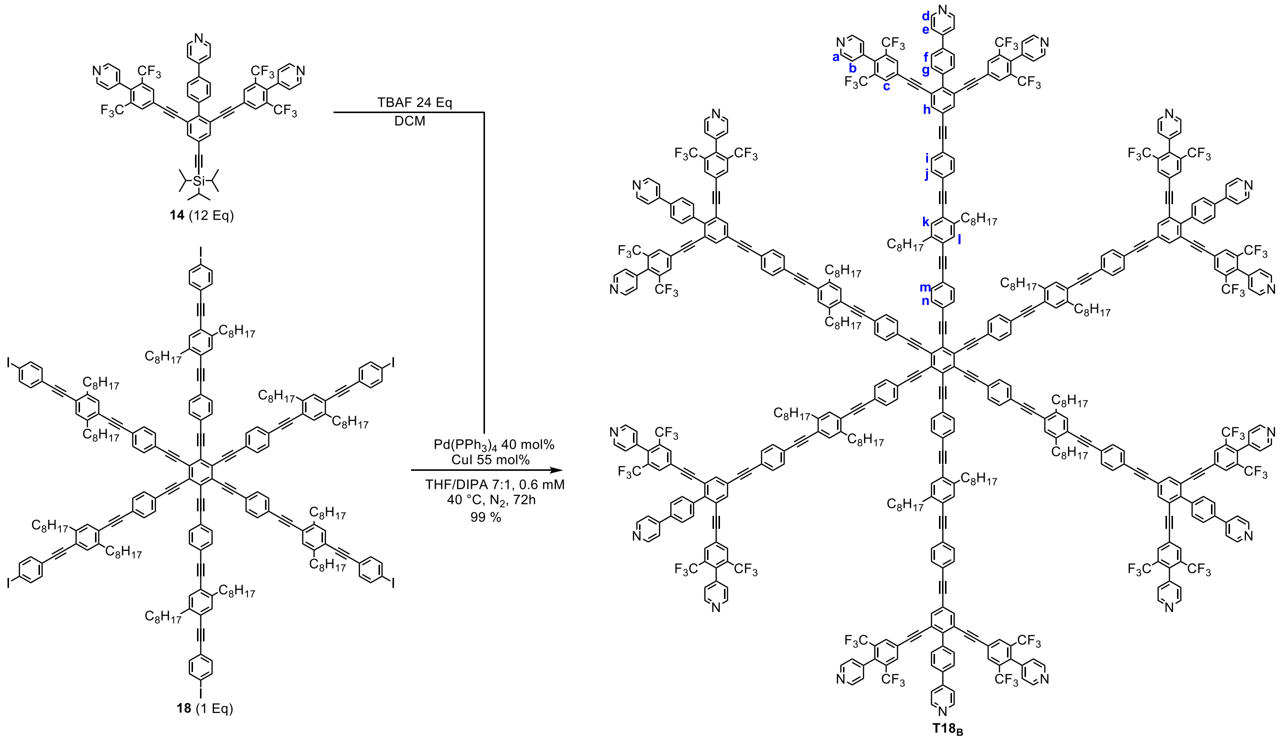

To a stirring solution of **14** (29.1 mg, 12 Eq, 28.0 µmol) in CH_2Cl_2 (5 mL) was added TBAF (84 µL, 1.0 M in THF, 24 Eq, 84 µmol). The solution was stirred for 15 min then CH_2Cl_2 (2 mL), water (5 mL) and acetic acid (10 µL) were added. The mixture was then poured into a separating funnel and extracted with chloroform (3 × 10 mL). The organic phase was dried with $MgSO_4$, then concentrated in vacuo and used in the next step without further purification.

In an oven dried Schlenk tube, deprotected **14** and **18** (9.3 mg, 1 Eq, 2.3 µmol) were dissolved in THF (3.41 mL) and DIPA (487 µL), then freeze-pump-thaw degassed (5×). After refreezing the solution and under a positive pressure of N_2, CuI (0.25 mg, 0.55 Eq, 1.28 µmol) and $Pd(PPh_3)_4$ (1.08 mg, 0.4 Eq, 0.934 µmol) were added. The Schlenk tube was stoppered, evacuated/refilled with N_2 (5×), then stirred at 40 °C for 72 h.

The crude reaction mixture was concentrated and redissolved in a minimum of chloroform + 3% methanol, then purified by flash column chromatography (SiO_2, chloroform + 3% MeOH, then chloroform + 3% MeOH + 1% $^tBuNH_2$) to yield **T18$_B$** as a yellow film (19.7 mg, 99%).

^{1}H NMR (600 MHz, $CDCl_3$) δ_H = 8.72 – 8.69 (m, 12H, **H$_d$**), 8.67 – 8.64 (m, 24H, **H$_a$**), 7.91 (s, 12H, **H$_h$**), 7.85 (d, J = 7.9 Hz, 12H, **H$_f$**), 7.80 – 7.76 (m, 36H, **H$_g$**, **H$_c$**), 7.65 (d, J = 7.3 Hz, 12H, **H$_n$**), 7.59 – 7.52 (m, 48H, **H$_e$**, **H$_i$**, **H$_j$**, **H$_m$**), 7.41 (m, 12H, **H$_k$**, **H$_l$**), 7.14 (d, J = 5.0 Hz, 24H, **H$_b$**), 2.89 – 2.72 (m, 24H, Ar-**CH$_2$**), 1.78 – 1.66 (m, 24H, Ar-CH$_2$-**CH$_2$**), 1.47 – 1.19 (m, 120H, **-CH$_2$-**), 0.92 – 0.80 (m, 36H, **CH$_3$**).
^{19}F NMR (470 MHz, $CDCl_3$) δ_F = **–**57.61 ppm (referenced against C_6F_6 (δ_F = **–**161.64) as an internal standard).
^{13}C NMR – (151 MHz, $CDCl_3$) δ_C = 150.61, 148.93, 147.48, 146.08, 142.60, 141.94, 138.78, 136.70, 136.04, 132.63, 131.92, 131.81, 131.71, 131.57, 131.37, 131.18, 127.66, 126.44, 124.76, 124.50, 124.27, 123.68, 123.48, 122.78, 122.32, 121.65, 121.53, 119.83, 99.75, 93.84, 91.67, 91.50, 91.03, 90.72, 89.18, 88.89, 34.33, 34.29, 32.06, 30.86, 30.80, 29.75, 29.68, 29.46, 29.43, 28.42, 22.83, 14.28.
MALDI-ToF MS m/z 8506.068 (calculated for ($[C_{540}H_{396}F_{72}N_{18}]^{\bullet+}$) = **(M)**$^{\bullet+}$: 8500.046).

Oligomerization of $P3_{OOct}$

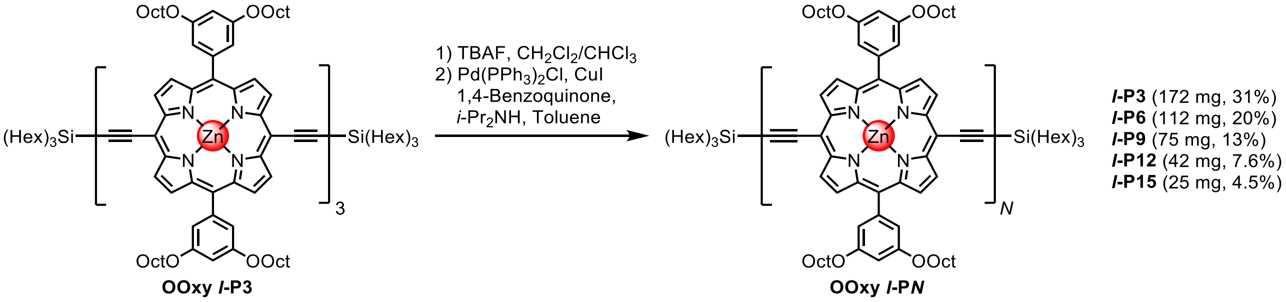

A solution of TBAF (1.0 M in THF, 145 µL, 0.145 mmol) was added to a solution of **$P3_{OOct}$** (553 mg, 0.145 mmol) in $CHCl_3$ (25 mL), CH_2Cl_2 (25 mL), and pyridine (1 mL). The deprotection was monitored by TLC (PE : EtOAc : pyridine = 10 : 1 : 1). Once a desired ratio of bis-deprotected, mono-deprotected, and starting material had been formed, AcOH (20 µL) was added, and the reaction mixture was immediately filtered through a short plug (SiO_2, $CHCl_3$ + 1% pyridine). The filtered mixture was concentrated under reduced pressure and redissolved in toluene (65 mL). A freshly prepared catalyst solution of $Pd(PPh_3)_2Cl_2$ (66 mg, 0.094 mmol), CuI (163 mg, 0.854 mmol), and 1,4-benzoquinone (156 mg, 1.45 mmol) in diisopropylamine (12 mL) and toluene (45 mL) was added to the porphyrin solution, and the reaction mixture was stirred vigorously in an open flask. After one hour, the reaction mixture was filtered through a plug (SiO_2, $CHCl_3$ + 1% pyridine) and concentrated under reduced pressure. The crude oligomer mixture was redissolved in toluene + 1% pyridine (10 mL), passed through a size-exclusion column (Bio-Beads, S-X1, toluene + 1% pyridine) and separated by recycling GPC (toluene + 1% pyridine). Each fraction of separated oligomer was concentrated under reduced pressure and precipitated from MeOH/$CHCl_3$ (ca. 9:1) by layered addition of MeOH onto a solution of oligomer in $CHCl_3$, which yielded each porphyrin oligomer as a solid brown powder.

Isolated yields: **$P3_{OOct}$** (172 mg, 31%), **$P6_{OOct}$** (112 mg, 20%), **$P9_{OOct}$** (75 mg, 13%), **$P12_{OOct}$** (42 mg, 7.6%), **$P15_{OOct}$** (25 mg, 4.5%).

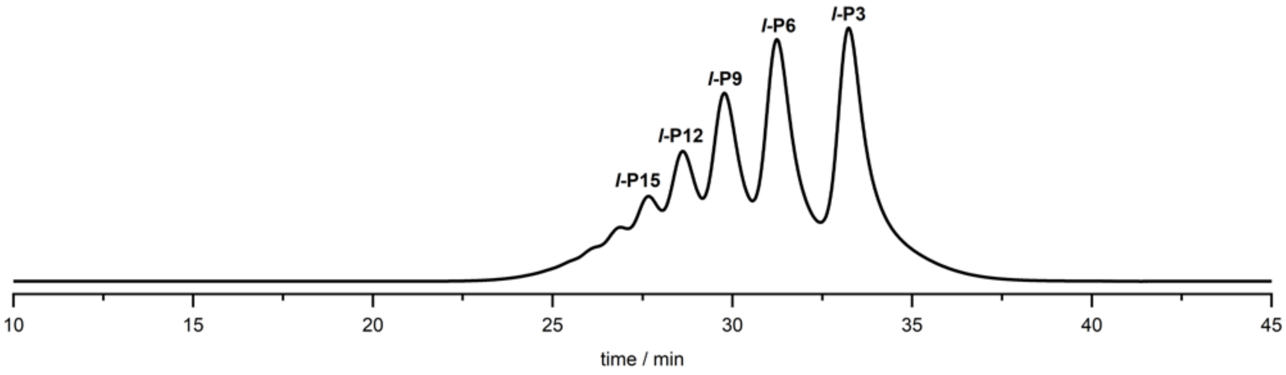

Figure S8. Analytical GPC trace of the crude reaction mixture from alkyne homo-coupling of a partially deprotected mixture of **$P3_{OOct}$** (THF + 1% pyridine, λ = 500 nm).

P3$_{OOct}$:

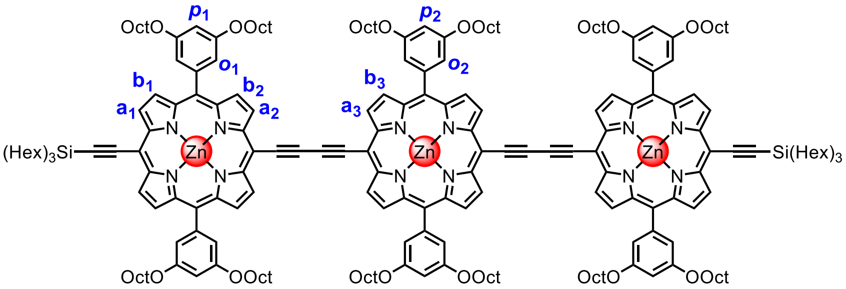

^{1}H NMR (700 MHz, $CDCl_3$, 298 K) δ_H = 9.94–9.79 (m, 8H, $a_2 + a_3$), 9.63 (d, J = 4.4 Hz, 4H, a_1), 9.05 (d, J = 4.4 Hz, 8H, $b_2 + b_3$), 8.95 (d, J = 4.4 Hz, 4H, b_1), 7.40 (d, J = 1.6 Hz, 4H, o_2), 7.37 (d, J = 1.6 Hz, 8H, o_1), 6.93–6.91 (m, 2H, p_2), 6.91–6.90 (m, 4H, p_1), 4.21–4.13 (m, 24H, -OCH_2-), 1.93–1.86 (m, 24H, -CH_2-), 1.80–1.75 (m, 12H, -CH_2-), 1.58–1.49 (m, 36H, -CH_2-), 1.44–1.24 (m, 120H, -CH_2-), 1.04–1.00 (m, 12H, -$SiCH_2$-), 0.91 (t, J = 7.0 Hz, 18H, -CH_3), 0.88–0.84 (m, 36H, -CH_3) ppm.
MALDI-ToF MS m/z 3820.9 (calculated for $[C_{240}H_{324}N_{12}O_{12}Si_2Zn_3]^{\bullet+}$ = **(M)**$^{\bullet+}$: 3821.3).
UV-vis-NIR ($CDCl_3$ +1% pyridine, 298 K) λ_{max} (ε / 10^6 M^{-1} cm^{-1}): 462 (0.47), 499 (0.22), 588 (0.03), 754 (0.17) nm.

P6$_{OOct}$:

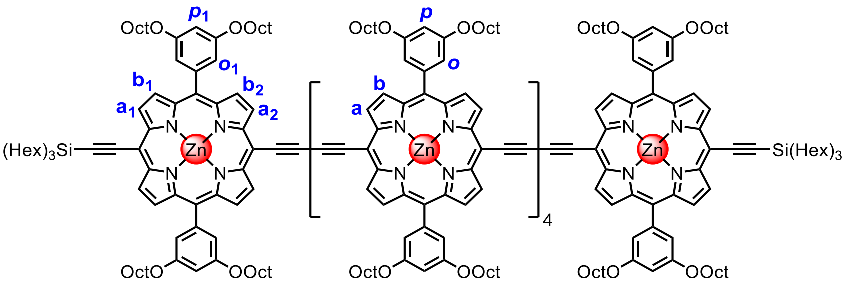

^{1}H NMR (700 MHz, $CDCl_3$, 298 K) δ_H = 9.97–9.80 (m, 20H, a_2 + a), 9.63 (d, J = 4.3 Hz, 4H, a_1), 9.14–9.01 (m, 20H, b_2 + b), 8.96 (d, J = 4.3 Hz, 4H, b_1), 7.42 (d, J = 2.2 Hz, 8H, *o*), 7.42 (d, J = 2.2 Hz, 8H, *o*), 7.37 (d, J = 2.2 Hz, 8H, *o*1), 6.95–6.92 (m, 8H, *p*), 6.92–6.90 (m, 4H, p_1), 4.23–4.14 (m, 48H, -OCH_2-), 1.94–1.87 (m, 48H, -CH_2-), 1.80–1.75 (m, 12H, -CH_2-), 1.58–1.50 (m, 60H, -CH_2-), 1.44–1.24 (m, 216H, -CH_2-), 1.05–1.00 (m, 12H, -CH_2-), 0.91 (t, J = 7.1 Hz, 18H, -CH_3), 0.89–0.84 (m, 72H, -CH_3) ppm.
MALDI-ToF MS m/z 7075.2 (calculated for $[C_{444}H_{570}N_{24}O_{24}Si_2Zn_6]^{\bullet+}$ = **(M)**$^{\bullet+}$: 7075.9).
UV-vis-NIR ($CDCl_3$ +1% pyridine, 298 K) λ_{max} (ε / 10^6 M^{-1} cm^{-1}): 465 (0.83), 498 (0.59), 590 (0.06), 796 (0.40) nm.

P9$_{OOct}$:

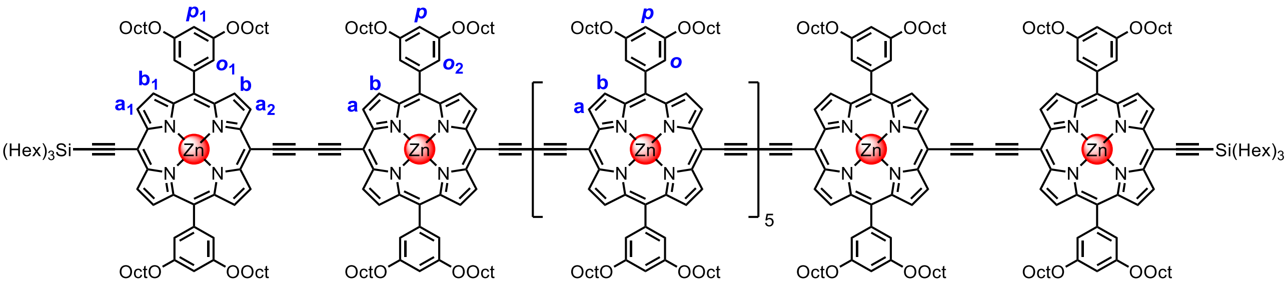

^{1}H NMR (600 MHz, $CDCl_3$, 298 K) δ_H = 9.91–9.87 (m, 28H, a), 9.87 (d, J = 5.1 Hz, 4H, a_2), 9.63 (d, J = 4.5 Hz, 4H, a_1), 9.12–9.04 (m, 32H, b), 8.95 (d, J = 4.5 Hz, 4H, b_1), 7.43–7.42 (m, 20H, *o*), 7.41 (d, J = 2.1 Hz, 8H, o_2), 7.37 (d, J = 2.0 Hz, 8H, o_1), 6.95–6.92 (m, 14H, *p*), 6.91 (t, J = 2.0 Hz, 4H, p_1), 4.31–4.06 (m, 72H, -OCH_2-), 1.96–1.24 (m, 492H, -CH_2-), 1.06–0.82 (m, 126H, -CH_3) ppm.
MALDI-ToF MS m/z 10331 (calculated for $[C_{648}H_{816}N_{36}O_{36}Si_2Zn_9]^{\bullet+}$ = **(M)**$^{\bullet+}$: 10330).
UV-vis-NIR ($CDCl_3$ +1% pyridine, 298 K) λ_{max} (ε / 10^6 M^{-1} cm^{-1}): 469 (1.13), 592 (0.09), 808 (0.63) nm.

P12$_{OOct}$:

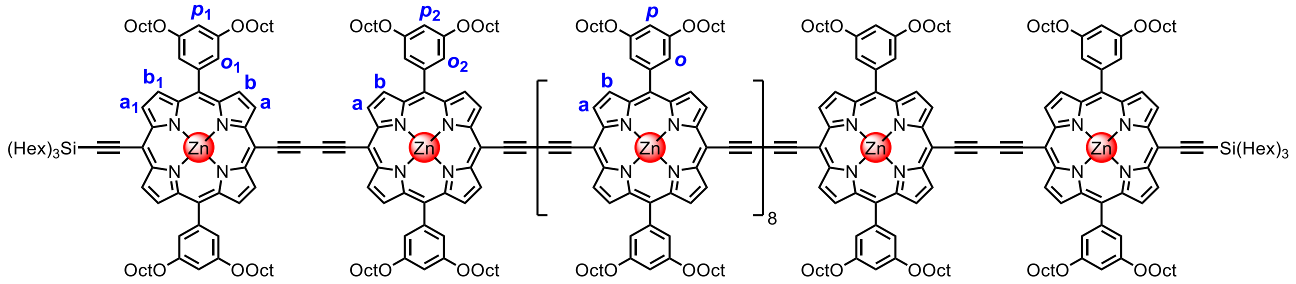

^{1}H NMR (600 MHz, CDCl$_3$, 298 K) δ_H = 9.98–9.81 (m, 44H, a), 9.63 (d, J = 4.5 Hz, 4H, a$_1$), 9.19–9.00 (m, 44H, b), 8.95 (d, J = 4.5 Hz, 4H, b$_1$), 7.44–7.42 (m, 32H, *o*), 7.41 (d, J = 2.3 Hz, 8H, o_2), 7.37 (d, J = 2.3 Hz, 8H, o_1), 6.95–6.93 (m, 16H, *p*), 6.93 (t, J = 2.3 Hz, 4H, p_2), 6.91 (t, J = 2.3 Hz, 4H, p_1), 4.20 (s, 96H, -OCH$_2$-), 1.95–1.87 (m, 96H, -CH$_2$-), 1.81–1.75 (m, 12H, -CH$_2$-), 1.61–1.49 (m, 108H, -CH$_2$-), 1.45–1.24 (m, 408H, -CH$_2$-), 1.04–1.00 (m, 12H, Si-CH$_2$-), 0.95–0.82 (m, 162H, -CH$_3$) ppm.
MALDI-ToF MS *m/z* 13581 (calculated for $[C_{852}H_{1062}N_{48}O_{48}Si_2Zn_{12}]^{\bullet+}$ = **(M)**$^{\bullet+}$: 13584).
UV-vis-NIR (CDCl$_3$ +1% pyridine, 298 K) λ_{max} (ε / 10^6 M^{-1} cm^{-1}): 470 (1.53), 592 (0.12), 811 (0.90) nm.

P15$_{OOct}$:

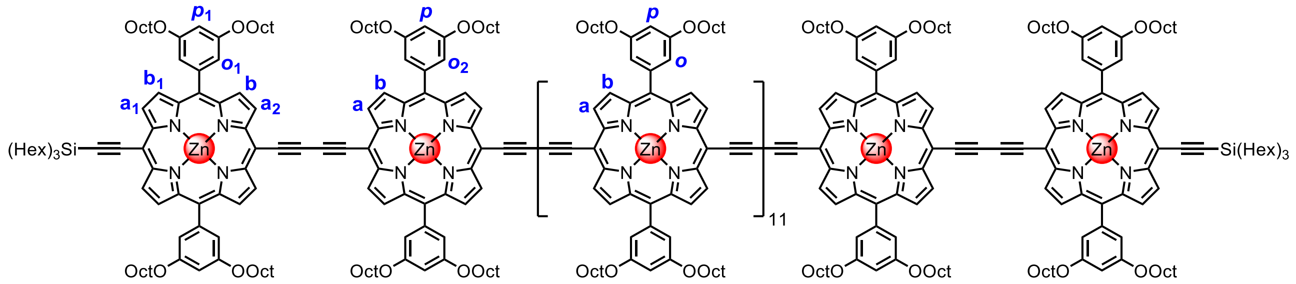

^{1}H NMR (600 MHz, CDCl$_3$, 298 K) δ_H = 9.91–9.87 (m, 52H, a), 9.87 (d, J = 5.0 Hz, 4H, a$_2$), 9.63 (d, J = 4.4 Hz, 4H, a$_1$), 9.13–9.02 (m, 56H, b), 8.95 (d, J = 4.4 Hz, 4H, b$_1$), 7.44–7.42 (m, 44H, *o*), 7.41 (d, J = 2.0 Hz, 8H, o_2), 7.37 (d, J = 2.3 Hz, 8H, o_1), 6.95–6.92 (m, 26H, *p*), 6.91 (t, J = 2.1 Hz, 4H, p_1), 4.38–4.03 (m, 120H, -OCH$_2$-), 1.97–1.23 (m, 768H, -CH$_2$-), 1.04–1.00 (m, 12H, Si-CH$_2$-), 0.93–0.84 (m, 198H, -CH$_3$). ppm.
MALDI-ToF MS *m/z* 16837 (calculated for $[C_{1056}H_{1308}N_{60}O_{60}Si_2Zn_{15}]^{\bullet+}$ = **(M)**$^{\bullet+}$: 16839).
UV-vis-NIR (CDCl$_3$ +1% pyridine, 298 K) λ_{max} (ε / 10^6 M^{-1} cm^{-1}): 471 (1.80), 591 (0.14), 814 (1.11) nm.

Oligomerization of $P3_{THS}$

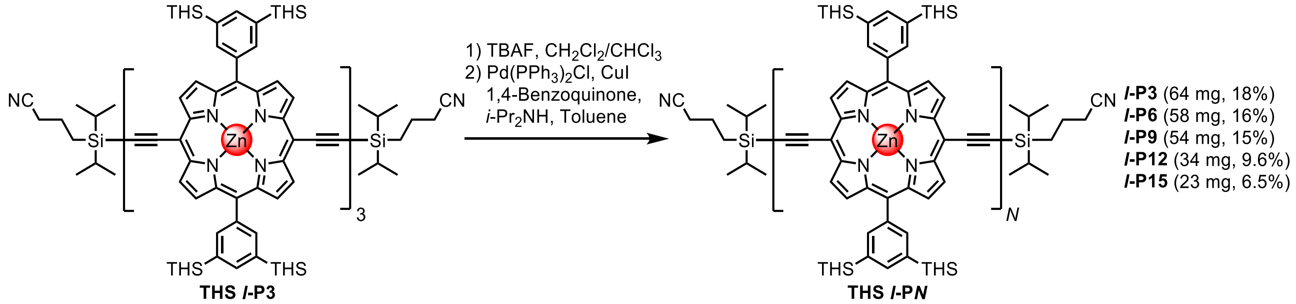

A solution of TBAF (1.0 M in THF, 65 µL, 65 µmol) was added to a solution of **P3(THS)** (356 mg, 65 µmol) in $CHCl_3$ (12 mL), CH_2Cl_2 (12 mL), and pyridine (1 mL). The deprotection was monitored by TLC (CH_2Cl_2 : PE = 1 : 4 + 1% pyridine). Once a desired ratio of bis-deprotected, mono-deprotected, and starting material had been formed, AcOH (10 µL) was added, and the reaction mixture was immediately filtered through a short plug (SiO_2, $CHCl_3$ + 1% pyridine). The filtered mixture was concentrated under reduced pressure and redissolved in toluene (20 mL). A freshly prepared catalyst solution of $Pd(PPh_3)_2Cl_2$ (9.1 mg, 13 µmol), CuI (25 mg, 131 µmol), and 1,4-benzoquinone (53 mg, 521 µmol) in diisopropylamine (9 mL) and toluene (20 mL) was added to the porphyrin solution, and the reaction mixture was stirred vigorously in an open flask. After 1.5 h, the reaction mixture was filtered through a plug (SiO_2, $CHCl_3$ + 1% pyridine) and concentrated under reduced pressure. The crude oligomer mixture was redissolved in toluene + 1% pyridine (5 mL), passed through a size-exclusion column (Bio-Beads, S-X1, toluene + 1% pyridine) and separated by recycling GPC (toluene + 1% pyridine). Each fraction of separated oligomer was concentrated under reduced pressure and precipitated from $MeOH/CHCl_3$ (ca. 9:1) by layered addition of MeOH onto a solution of oligomer in $CHCl_3$, which yielded each porphyrin oligomer as a solid brown powder.

Isolated yields: **$P3_{THS}$** (64 mg, 18%), **$P6_{THS}$** (58 mg, 16%), **$P9_{THS}$** (54 mg, 15%), **$P12_{THS}$** (34 mg, 9.6%), **$P15_{THS}$** (23 mg, 6.5%).

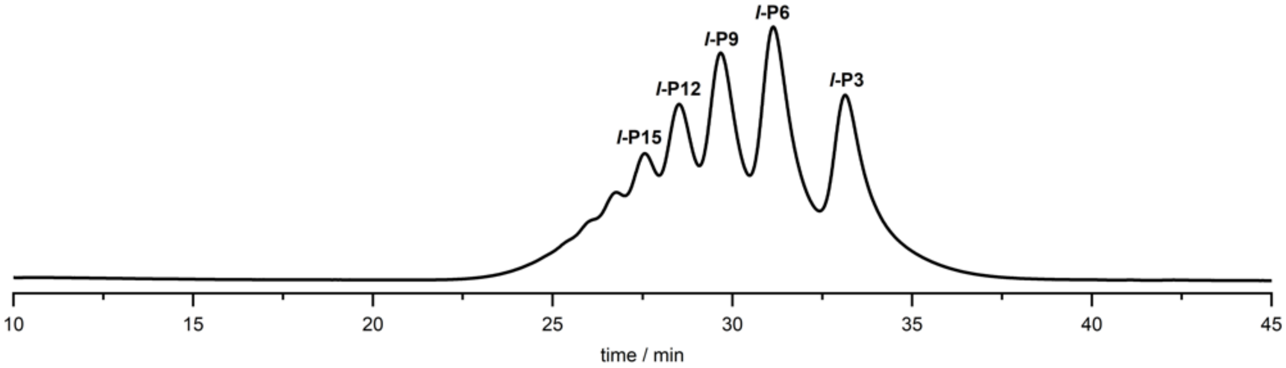

Figure S9. Analytical GPC trace of the crude reaction mixture from alkyne homo-coupling of a partially deprotected mixture of **$P3_{THS}$** (THF + 1% pyridine, λ = 500 nm).

P3$_{THS}$:

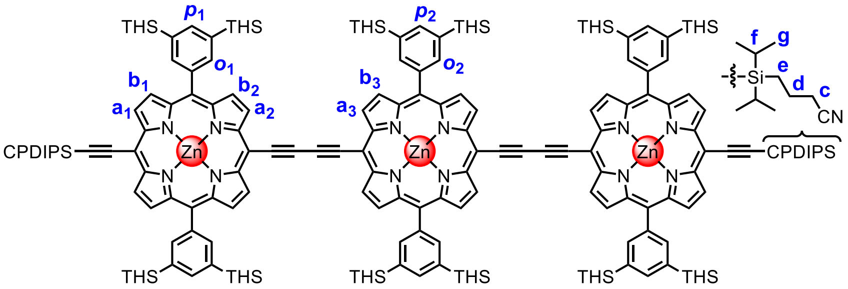

^{1}H NMR (600 MHz, $CDCl_3$, 298 K) δ_H = 9.88–9.82 (m, 8H, $a_2 + a_3$), 9.62 (d, J = 4.5 Hz, 4H, a_1), 8.92 (d, J = 4.5 Hz, 4H, b_2/b_3), 8.90 (d, J = 4.5 Hz, 4H, b_2/b_3), 8.84 (d, J = 4.5 Hz, 4H, b_1), 8.29 (s, 4H, o_1), 8.24 (s, 8H, o_2), 8.00 (s, 2H, p_1), 7.99 (s, 4H, p_2), 2.54 (t, J = 6.9 Hz, 4H, c), 2.23–2.17 (m, 4H, d), 1.54–1.46 (m, 72H, -CH_2-), 1.46–1.35 (m, 100H, -CH_2-, + f + g), 1.34–1.26 (m, 144H, -CH_2-), 1.18–1.14 (m, 4H, e), 0.98–0.91 (m, 72H, -CH_2-), 0.90–0.84 (m, 108H, -CH_3) ppm.
MALDI-ToF MS m/z 5469.9 (calculated for $[C_{344}H_{549}N_{14}Si_{14}Zn_3]^{\bullet+}$ = **(M)**$^{\bullet+}$: 5469.8).
UV-vis-NIR ($CDCl_3$ +1% pyridine, 298 K) λ_{max} (ε / 10^6 M^{-1} cm^{-1}): 453 (0.57), 492 (0.30), 580 (0.04), 637 (0.05), 731 (0.19) nm.

P6$_{THS}$:

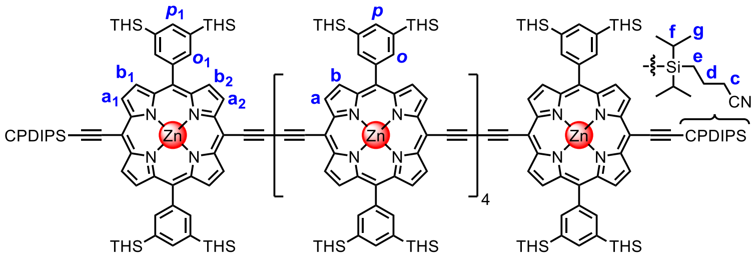

^{1}H NMR (600 MHz, $CDCl_3$, 298 K) δ_H = 9.91–9.83 (m, 20H, a_2 + a), 9.62 (d, J = 4.5 Hz, 4H, a_1), 8.95–8.92 (m, 16H, b), 8.91 (d, J = 4.4 Hz, 4H, b_2), 8.84 (d, J = 4.5 Hz, 4H, b_1), 8.31 (d, J = 0.8 Hz, 8H, o), 8.30 (d, J = 0.8 Hz, 8H, o), 8.25 (d, J = 0.9 Hz, 8H, o_1), 8.02 (s, 4H, p), 8.01 (s, 4H, p), 7.99 (s, 4H, p_1), 2.55 (t, J = 6.9 Hz, 4H, c), 2.23–2.17 (m, 4H, d), 1.57–1.46 (m, 144H, -CH_2-), 1.46–1.36 (m, 172H, -CH_2- + f + g), 1.36–1.26 (m, 288H, -CH_2-), 1.19–1.14 (m, 4H, e), 1.00–0.92 (m, 144H, -CH_2-), 0.91–0.86 (m, 216H, -CH_3) ppm.
MALDI-ToF MS m/z 10577.9 (calculated for $[C_{668}H_{1059}N_{26}Si_{26}Zn_6]^{\bullet+}$ = **(M)**$^{\bullet+}$: 10577.4).
UV-vis-NIR ($CDCl_3$ +1% pyridine, 298 K) λ_{max} (ε / 10^6 M^{-1} cm^{-1}): 465 (0.87), 591 (0.06), 809 (0.42) nm.

P9$_{THS}$:

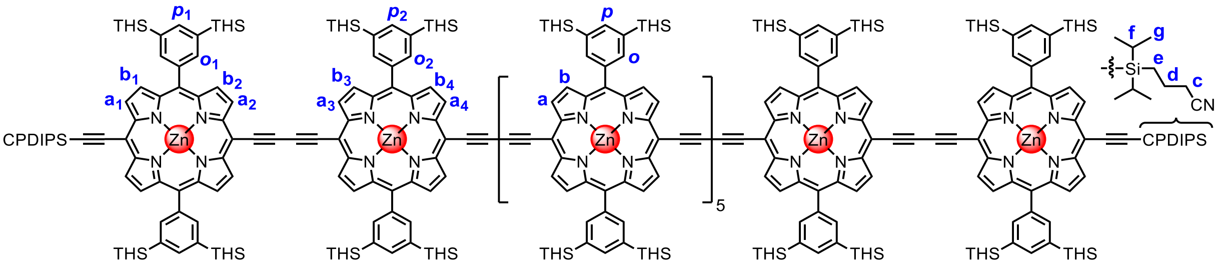

^{1}H NMR (600 MHz, $CDCl_3$, 298 K) δ_H = 9.90–9.86 (m, 28H, $a_3 + a_4$ + a), 9.86 (d, J = 4.4 Hz, 4H, a_2), 9.62 (d, J = 4.4 Hz, 4H, a_1), 8.96–8.91 (m, 28H, $b_3 + b_4$ + b), 8.91 (d, J = 4.4 Hz, 4H, b_2), 8.84 (d, J = 4.4 Hz, 4H, b_1), 8.33–8.30 (m, 20H, o), 8.30 (s, 8H, o_2), 8.25 (s, 8H, o_1), 8.03–8.01 (m, 10H, p), 8.01 (s, 4H, p_2), 7.99 (s, 4H, p_1), 2.54 (t, J = 6.9 Hz, 4H, c), 2.24–2.17 (m, 4H, d), 1.58–1.46 (m, 216H, -CH_2-), 1.46–1.36 (m, 244H, -CH_2- + f + g), 1.36–1.27 (m, 432H, -CH_2-), 1.18–1.15 (m, 4H, e), 0.99 – 0.92 (m, 216H, -CH_2-), 0.91–0.85 (m, 324H, -CH_3) ppm.
MALDI-ToF MS m/z 15687 (calculated for $[C_{992}H_{1569}N_{38}Si_{38}Zn_9]^{\bullet+}$ = **(M)**$^{\bullet+}$: 15684).
UV-vis-NIR ($CDCl_3$ +1% pyridine, 298 K) λ_{max} (ε / 10^6 M^{-1} cm^{-1}): 467 (1.21), 592 (0.09), 818 (0.67) nm.

P12$_{THS}$:

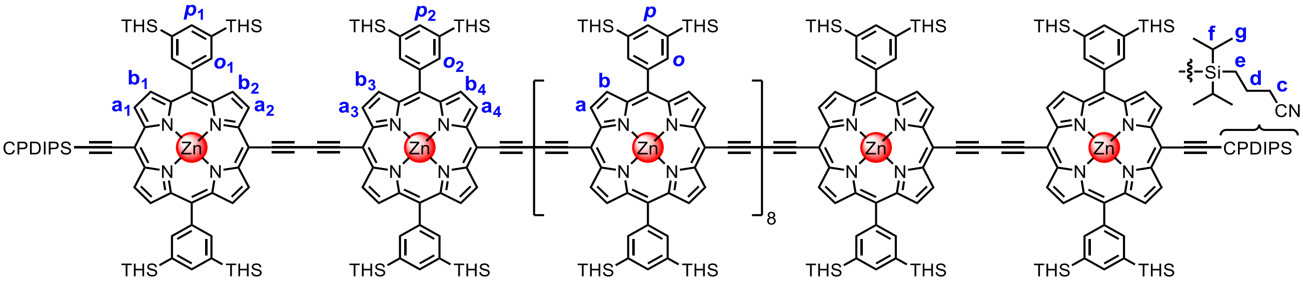

^{1}H NMR (600 MHz, $CDCl_3$, 298 K) δ_H = 9.89–9.86 (m, 40H, $a_3 + a_4$ + a), 9.85 (d, J = 4.4 Hz, 4H, a_2), 9.61 (d, J = 4.4 Hz, 4H, a_1), 8.97–8.91 (m, 40H, $b_3 + b_4$ + b), 8.90 (d, J = 4.4 Hz, 4H, b_2), 8.84 (d, J = 4.4 Hz, 4H, b_1), 8.32–8.30 (m, 32H, *o*), 8.29 (s, 8H, o_2), 8.24 (s, 8H, o_1), 8.03–8.01 (m, 16H, *p*), 8.01 (s, 4H, p_2), 7.99 (s, 4H, p_1), 2.53 (t, J = 6.9 Hz, 4H, c), 2.22–2.11 (m, 4H, d), 1.56–1.46 (m, 288H, -CH_2-), 1.45–1.35 (m, 316H, -CH_2- + f + g), 1.35–1.26 (m, 576H, -CH_2-), 1.17–1.13 (m, 4H, e), 1.00–0.91 (m, 288H, -CH_2-), 0.91–0.83 (m, 432H, -CH_3) ppm.
MALDI-ToF MS *m/z* 20788 (calculated for $[C_{1316}H_{2079}N_{50}Si_{50}Zn_{12}]^{\bullet+}$ = **(M)**$^{\bullet+}$: 20790).
UV-vis-NIR ($CDCl_3$ +1% pyridine, 298 K) λ_{max} (ε / 10^6 M^{-1} cm^{-1}): 468 (1.59), 593 (0.12), 823 (0.94) nm.

P15$_{THS}$:

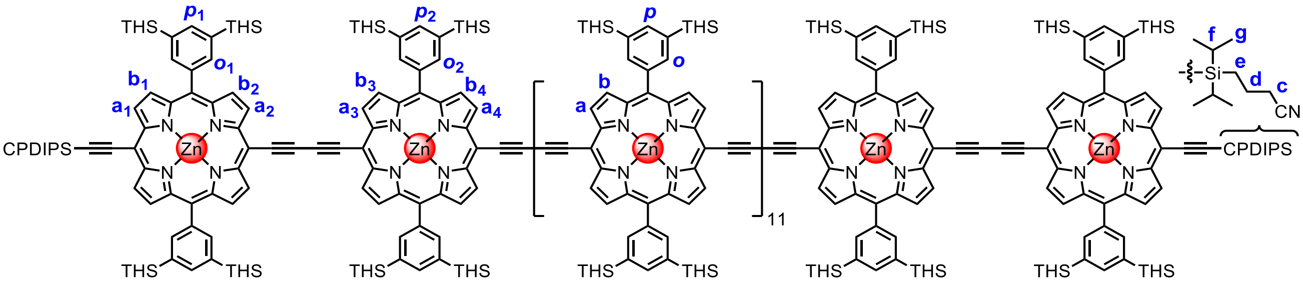

^{1}H NMR (600 MHz, $CDCl_3$, 298 K) δ_H = 9.89–9.86 (m, 52H, $a_3 + a_4$ + a), 9.85 (d, J = 4.4 Hz, 4H, a_2), 9.61 (d, J = 4.4 Hz, 4H, a_1), 8.95–8.91 (m, 52H, $b_3 + b_4$ + b), 8.90 (d, J = 4.4 Hz, 4H, b_2), 8.84 (d, J = 4.4 Hz, 4H, b_1), 8.30 (s, 44H, *o*), 8.29 (s, 8H, o_2), 8.24 (s, 8H, o_1), 8.01 (s, 22H, *p*), 8.01 (s, 4H, p_2), 7.99 (s, 4H, p_1), 2.53 (t, J = 6.9 Hz, 4H, c), 1.55–1.47 (m, 360H, -CH_2-), 1.44–1.36 (m, 388H, -CH_2- + f + g), 1.35–1.27 (m, 720H, -CH_2-), 1.17–1.13 (m, 4H, e), 0.99–0.91 (m, 360H, -CH_2-), 0.91–0.85 (m, 540H, -CH_3) ppm.
MALDI-ToF MS *m/z* 25899 (calculated for $[C_{1640}H_{2589}N_{62}Si_{62}Zn_{15}]^{\bullet+}$ = **(M)**$^{\bullet+}$: 25897).
UV-vis-NIR ($CDCl_3$ +1% pyridine, 298 K) λ_{max} (ε / 10^6 M^{-1} cm^{-1}): 469 (2.08), 592 (0.15), 825 (1.28) nm.

Synthesis of *c*-P18$_{OOct}$ from P9$_{OOct}$ and T6

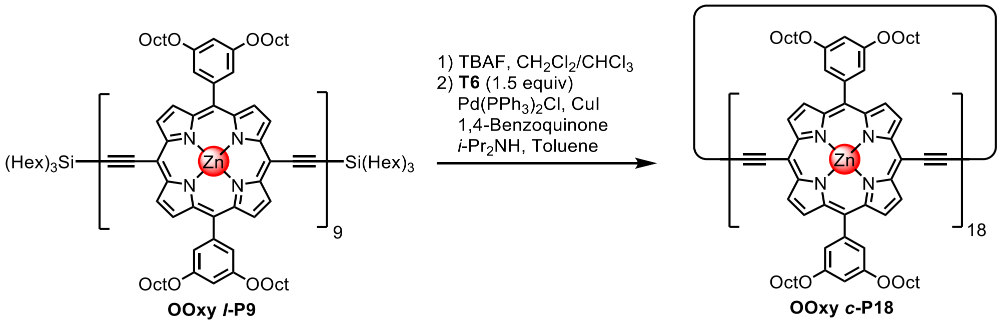

A solution of TBAF (1.0 M in THF, 30 µL, 30 µmol) was added to a solution of **P9$_{OOct}$** (10.0 mg, 0.968 µmol) in CH_2Cl_2 (5.5 mL) and pyridine (55 µL). After one hour, MeOH (0.2 mL) was added and the reaction volume was reduced to 1/5th its original volume using a stream nitrogen. Additional MeOH (10 mL) was added to induce precipitation. The supernatant was removed after centrifugation and the porphyrin oligomer was washed using MeOH (2 × 10 mL) and dried under reduced pressure to give fully deprotected **P9** (9.40 mg, 0.960 µmol) as confirmed by MALDI MS. A solution of **T6** template (1.44 mg, 1.44 µmol, 1.5 equivalents) dissolved in $CDCl_3$ (2.7 mL) and MeOH (0.3 mL) was added to a solution of deprotected **P9** in $CDCl_3$ (9.3 mL) and pyridine (93 µL), and the resulting mixture was sonicated for 10 min and concentrated under reduced pressure. The dry mixture was redissolved in $CDCl_3$ (9.3 mL) and sonicated for 10 min. A catalyst stock solution was prepared of $Pd(PPh_3)_2Cl_2$ (5.3 mg, 7.6 µmol), CuI (8.0 mg, 42 µmol), and 1,4-benzoquinone (18.6 mg, 172 µmol) in diisopropylamine (0.14 mL) and $CDCl_3$ (2.0 mL). Fresh catalyst stock solution (660 µL) was added to the solution of deprotected **P9$_{OOct}$** and **T6**, and the reaction mixture was stirred vigorously in an open flask. After 12 h, freshly prepared catalyst stock solution is added for a second time (660 µL) and the reaction mixture was stirred for 5 h, passed through a short plug (SiO_2, $CHCl_3$ + 1% pyridine), and concentrated under reduced pressure. The crude mixture was redissolved in toluene + 1% pyridine (5 mL), passed through a size-exclusion column (Bio-Beads, S-X1, toluene + 1% pyridine) and separated by recycling GPC (toluene + 1% pyridine). Each isolated oligomer was concentrated under reduced pressure and precipitated from MeOH/$CHCl_3$ (ca. 9:1) by layered addition of MeOH onto a solution of oligomer in $CHCl_3$, which yielded ***c*-P18$_{OOct}$** (4.28 mg, 46%), and ***c*-P9$_{OOct}$** (0.45 mg, 5%) as solid brown powders.

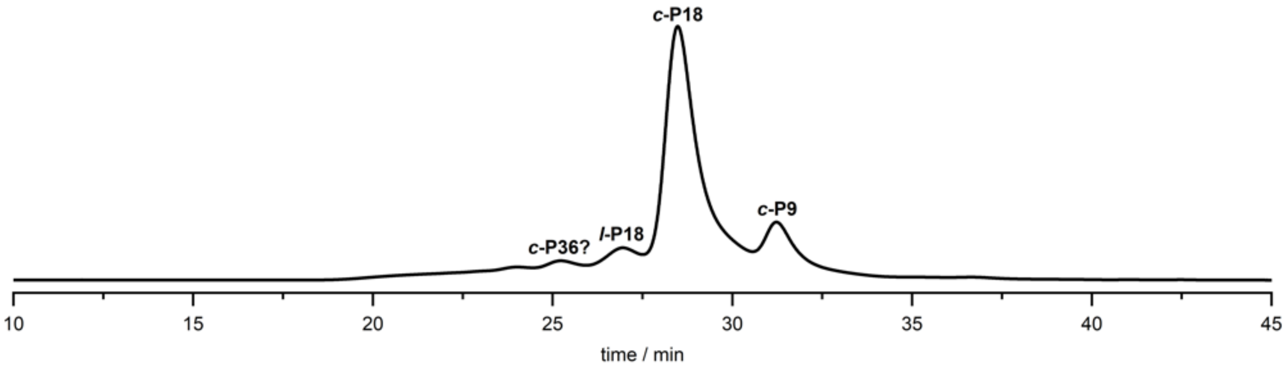

Figure S10. Analytical GPC trace of the crude reaction mixture from Vernier-templated alkyne homo-coupling of fully deprotected **P9$_{OOct}$** with **T6** (THF + 1% pyridine, λ = 500 nm).

Characterization of ***c*-P18$_{OOct}$**:

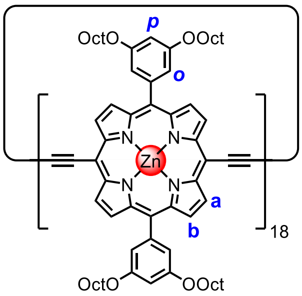

^{1}H NMR (500 MHz, $CDCl_3$, 298 K) δ_H = 9.87 (d, J = 4.6 Hz, 72H, **H$_a$**), 9.07 (d, J = 4.6 Hz, 72H, **H$_b$**), 7.41 (d, J = 1.9 Hz, 72H, **H$_o$**), 6.93 (t, J = 1.9 Hz, 36H, **H$_p$**), 4.32–4.05 (m, 144H, **-OCH$_2$-**), 1.97–1.85 (m, 144H, **-CH$_2$-**), 1.61–1.22 (m, 720H, **-CH$_2$-**), 0.91–0.81 (m, 216H, **-CH$_3$**) ppm.

MALDI-ToF MS *m*/*z* 19449.921 (calculated for $[C_{1224}H_{1476}N_{72}O_{72}Zn_{18}]^{\bullet+}$ = **(M)**$^{\bullet+}$: 19526.142).

UV-vis-NIR ($CDCl_3$ +1% pyridine, 298 K) λ_{max} (ε / 10^6 M^{-1} cm^{-1}): 475 (2.210), 493 (2.023), 597 (0.184), 816 (1.465) nm

Synthesis of *c*-P18$_{THS}$ from P9$_{THS}$ and T18$_A$

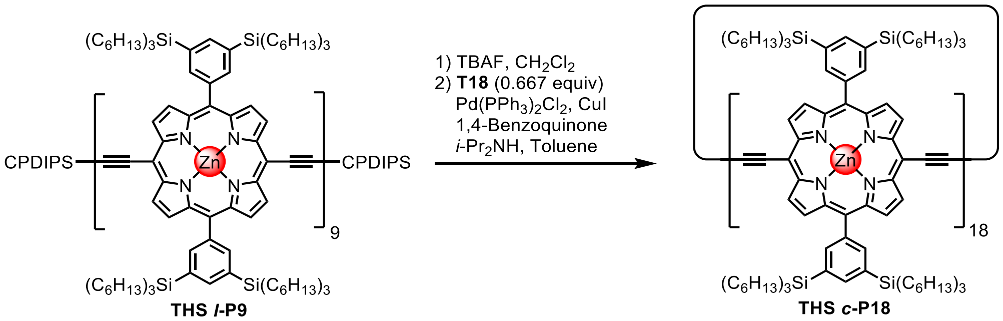

A solution of TBAF (1.0 M in THF, 32 µL, 32 µmol) was added to a solution of **P9$_{THS}$** (16.5 mg, 1.05 µmol) in CH_2Cl_2 (6.1 mL) and pyridine (61 µL). After one hour, MeOH (0.3 mL) was added and the reaction volume was reduced to 1/5th its original volume using a stream nitrogen. Additional MeOH (10 mL) was added to induce precipitation. The supernatant was removed after centrifugation and the porphyrin oligomer was washed using MeOH (2 × 10 mL) and dried under reduced pressure to give fully deprotected **P9$_{THS}$** (16 mg, 1.0 µmol) as confirmed by MALDI MS.

A solution of **T18** template (6.12 mg, 0.696 µmol, 0.667 equivalents) dissolved in $CDCl_3$ (3.93 mL) was added to a solution of deprotected **P9$_{THS}$** (16.0 mg, 1.04 µmol, 1 equivalent) in $CDCl_3$ (9.91 mL) and the resulting mixture was sonicated for 10 min. A catalyst stock solution was prepared of $Pd(PPh_3)_2Cl_2$ (5.3 mg, 7.6 µmol), CuI (8.0 mg, 42 µmol), and 1,4-benzoquinone (18.6 mg, 172 µmol) in diisopropylamine (0.14 mL) and $CDCl_3$ (2.0 mL). Fresh catalyst stock solution (560 µL) was added to the solution of deprotected **P9$_{THS}$** and **T18**, and the reaction mixture was stirred vigorously in an open flask. After 12 h, freshly prepared catalyst stock solution was added for a second time (560 µL) and the reaction mixture was stirred for 5 h, passed through a short plug (SiO_2, $CHCl_3$ + 5% pyridine), and concentrated under reduced pressure. The silica plug was then flushed with a mixture of $CHCl_3$:MeOH:*t*-BuNH (84:15:1, 200 mL), and the collected eluent concentrated and subjected to the same purification procedure as described for **T18**, to recover **T18** (4.88 mg, 80%). The crude reaction mixture was redissolved in toluene + 1% pyridine (5 mL), passed through a size-exclusion column (Bio-Beads, S-X1, toluene + 1% pyridine) and separated by recycling GPC (toluene + 1% pyridine). Each isolated oligomer was concentrated under reduced pressure and precipitated from MeOH/$CHCl_3$ (ca. 9:1) by layered addition of MeOH onto a solution of oligomer in $CHCl_3$, which yielded ***c*-P18$_{THS}$** (4.04 mg, 25%) as a solid brown powder.

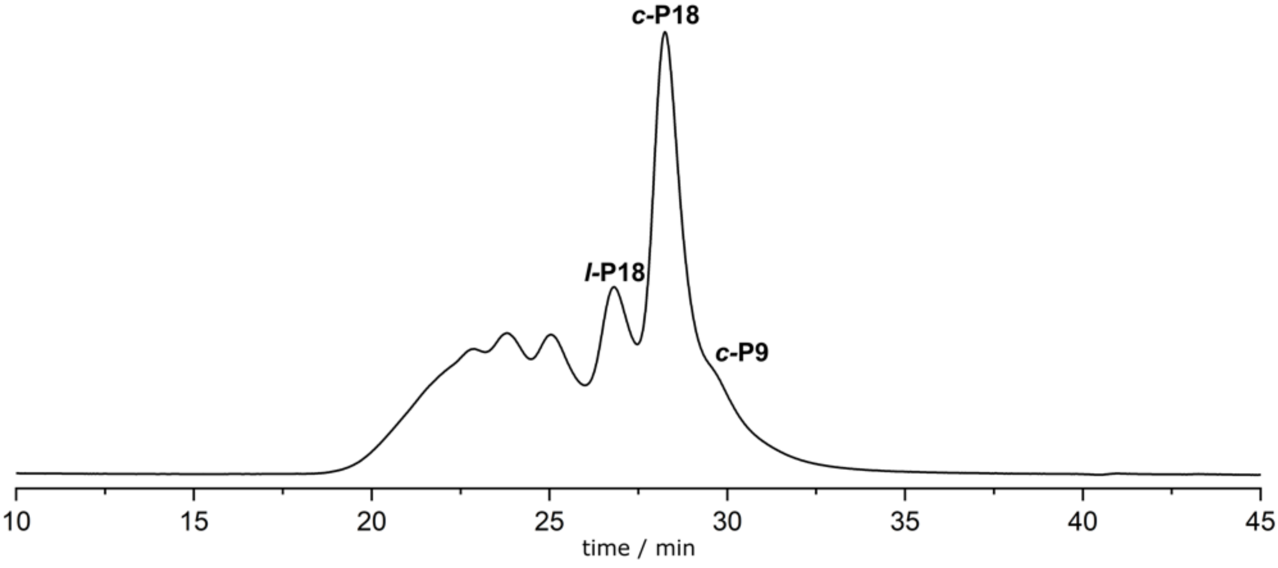

Figure S11. Analytical GPC trace of the crude reaction mixture from Vernier-templated alkyne homo-coupling of fully deprotected **P9$_{THS}$** with **T18** (THF + 1% pyridine, λ = 500 nm).

Characterization of ***c*-P18**$_{THS}$:

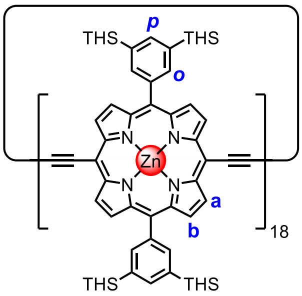

^{1}H NMR (600 MHz, $CDCl_3$) δ 9.86 (d, J = 4.6 Hz, 72H, **H**$_a$), 8.93 (d, J = 4.5 Hz, 72H, **H**$_b$), 8.30 (s, 72H, **H**$_o$), 8.02 (s, 36H, **H**$_p$), 1.57 – 1.48 (m, 216H, Ar-**CH**$_2$), 1.40 (t, J = 7.3 Hz, 216H, **-CH**$_2$**-**), 1.36 – 1.28 (m, 432H, **-CH**$_2$**-**), 1.00 – 0.94 (m, 216H, **-CH**$_2$**-**), 0.92 – 0.86 (m, 324H, **-CH**$_3$).
MALDI-ToF MS m/z 30767.8 (calculated for $[C_{1944}H_{3060}N_{72}Si_{72}Zn_{18}]^{\bullet+}$ = **(M)**$^{\bullet+}$: 30639.26).
UV-vis-NIR ($CDCl_3$ +1% pyridine, 298 K) λ_{max} (ε / 10^6 M^{-1} cm^{-1}): 467 (3.095), 486 (2.803), 777 (1.861) nm.

Summary of optimized macrocyclization reactions using P9$_{OOct}$ or P9$_{THS}$ and either T6, T18$_A$, T18$_B$ or no template

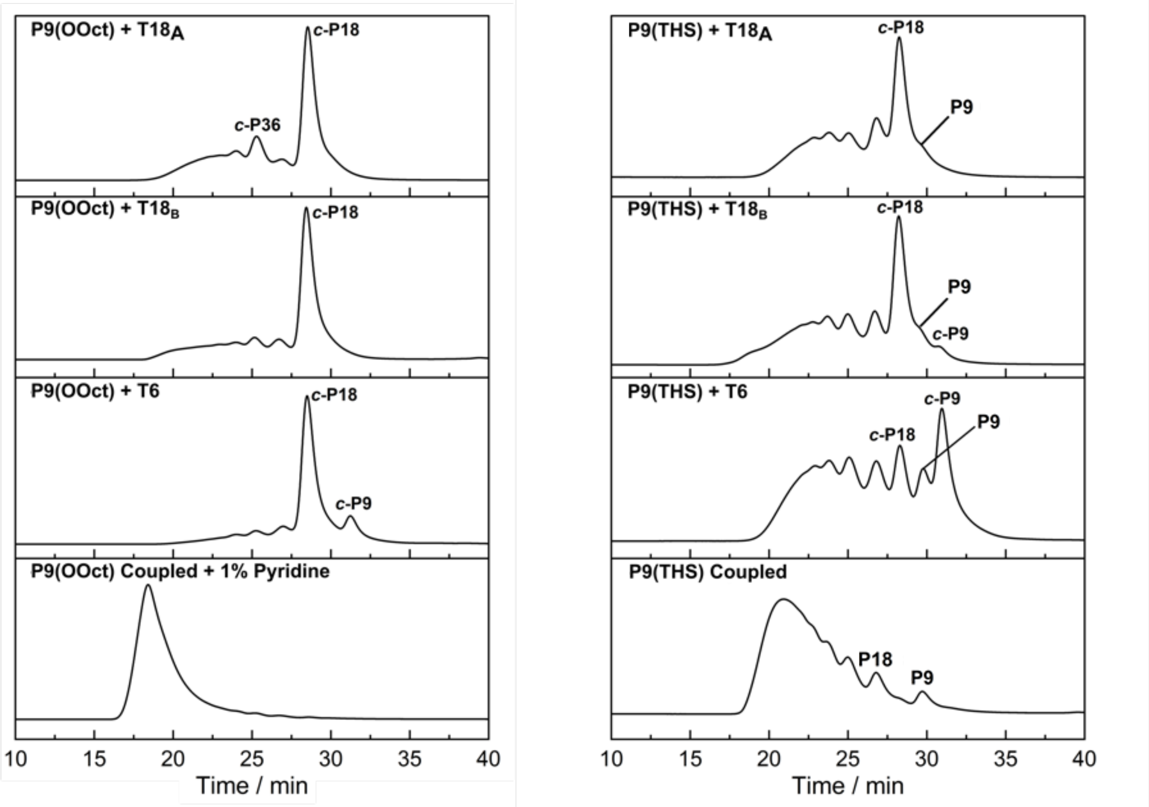

Figure S12. Analytical GPC trace of the crude reaction mixture from templated alkyne homo-coupling of fully deprotected **P9**$_{OOct}$ (left) or **P9**$_{THS}$ (right) with **T6**, **T18**$_A$, **T18**$_B$ or no template (THF + 1% pyridine, λ = 500 nm). **T6** reactions used a 2:3 ratio of **P9**:**T6**. **T18**$_{A/B}$ reactions used a 2:1 ratio of **P9**:**T18**$_{A/B}$.

Formation of *c*-P18$_{OOct}$·T18$_A$

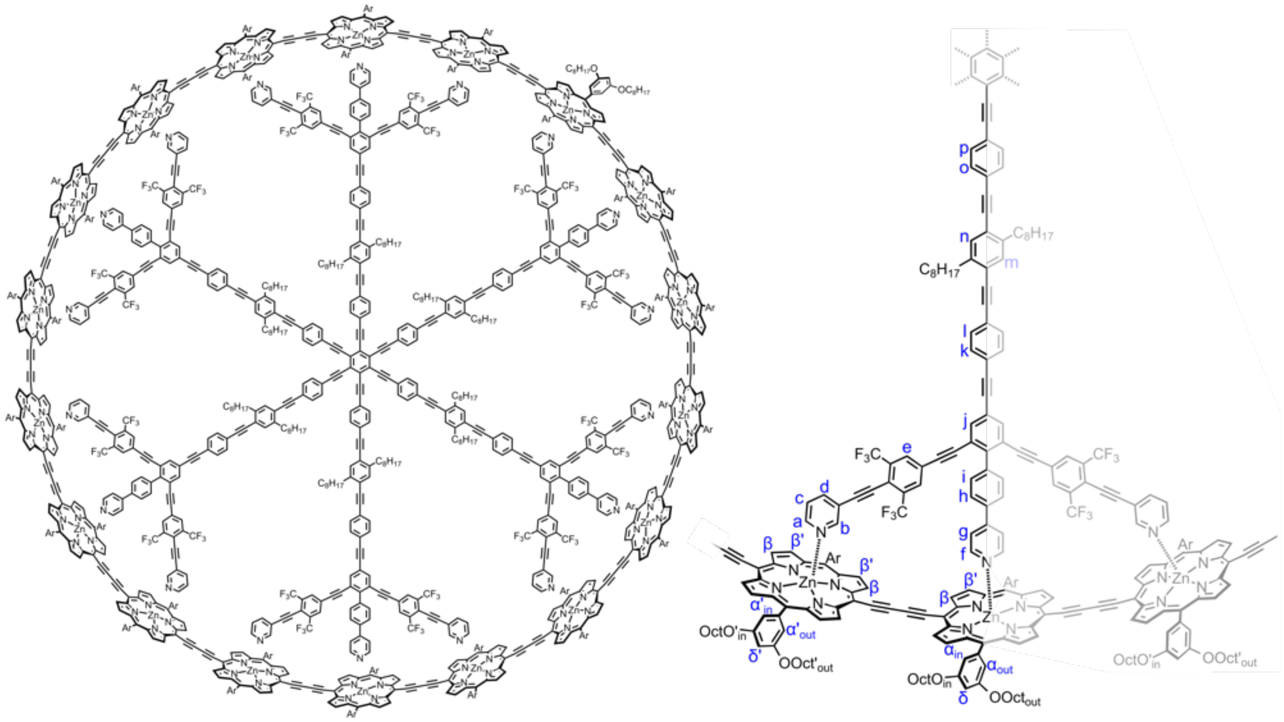

To a dried sample of ***c*-P18$_{OOct}$** (0.60 mg, 1 Eq, 31 nmol) in an NMR tube, was added $CDCl_3$ (450 µL) that had been freshly filtered over Al_2O_3. ^1H and ^{19}F NMR spectra were recorded for this sample after each addition of **T18$_A$** (0.54 mg, 2 Eq, 62 nmol, 3.07×10^{-4} M in $CDCl_3$). The titration was stopped as soon as the free **T18$_A$** fluorine signal was observed ($\delta_F \approx -62.70$ ppm). The NMR sample was dried under a stream of nitrogen then dissolved in 1:1 $CHCl_3$:pentane and passed over a short plug of silica (SiO_2, 1:1 $CHCl_3$:pentane), to remove excess **T18**. Concentrating this fraction yielded the ***c*-P18$_{OOct}$·T18$_A$** 1:1 complex as a green film (0.66 mg, 76% yield).

T18$_A$ retained on the silica plug was recovered by eluting with $CHCl_3$:MeOH:*t*-$BuNH_2$ 84:15:1.

Characterization of ***c*-P18$_{OOct}$·T18$_A$**:
^{1}H NMR (600 MHz, CD_2Cl_2) δ_H = 9.96–9.86 (m, 72H, **H$_\beta$**), 9.15–9.05 (m, 72, **H$_{\beta'}$**), 7.65 (s, 12H, **H$_j$**), 7.65–7.60 (m, 18H, **H$_p$, H$_{\alpha in}$**), 7.56–7.52 (m, 12H, **H$_o$**), 7.47–7.43 (m, 24H, **H$_k$, H$_l$**), 7.43–7.40 (m, 24H, **H$_{\alpha' in}$** + **H$_{\alpha' out}$**), 7.40 (s, 24H, **H$_e$**), 7.35–7.32 (m, 12H, **H$_m$, H$_n$**), 7.32–7.27 (m, 18H, **H$_i$ + H$_{\alpha out}$**), 6.95 (s, 6H, **H$_\delta$**), 6.93 (s, 12H, **H$_{\delta'}$**), 6.87–6.83 (m, 12H, **H$_h$**), 6.57 (d, *J* = 7.7 Hz, 12H, **H$_d$**), 5.97–5.92 (m, 12H, **H$_g$**), 5.77–5.72 (m, 12H, **H$_c$**), 4.33–4.24 (m, 24H, **(OCH$_2$)$_{in}$**), 4.24–4.07 (m, 120H, **(OCH$_2$)$_{out}$** + **(O'CH$_2$)**), 2.87–2.82 (m, 12H, **H$_f$**), 2.81–2.69 (m, 24H, **Ar-CH$_2$**), 2.81–2.76 (m, 12H, **H$_b$**), 2.76–2.69 (s, 12H, **H$_a$**), 2.00–1.80 (m, 144H, OCH$_2$-**CH$_2$-**), 1.70 – 1.14 (m, 888H, **-CH$_2$-**), 0.96 – 0.74 (m, 252H, **-CH$_3$**) ppm.
^{19}F NMR (470 MHz, CD_2Cl_2, 298K) δ_F = –63.19 ppm (referenced against C_6F_6 (δ_F = –162.61) as an internal standard).
MALDI-ToF MS *m/z* 28244.3 (calculated for $[C_{1788}H_{1872}F_{72}N_{90}O_{72}Zn_{18}]^{\bullet+}$ = **(M)**$^{\bullet+}$: 28319.20).
UV-vis-NIR ($CDCl_3$, 298 K) λ_{max} (ε / 10^6 M^{-1} cm^{-1}): 489 (2.396), 812 (2.029) nm.

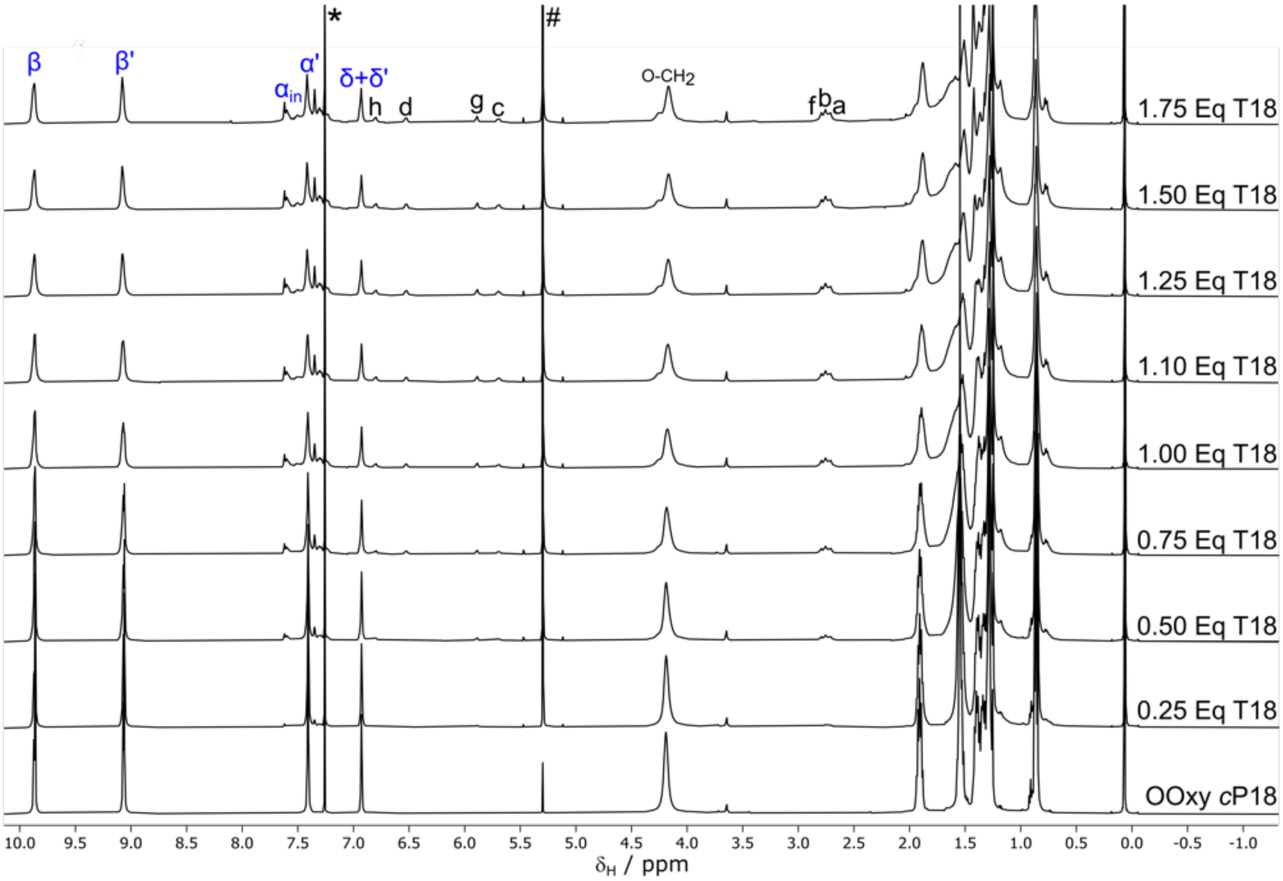

Figure S13. ^{1}H-NMR (500 MHz, $CDCl_3$) spectra from a formation titration of ***c*-P18**$_{\mathbf{OOct}}$**·T18**$_{\mathbf{A}}$.

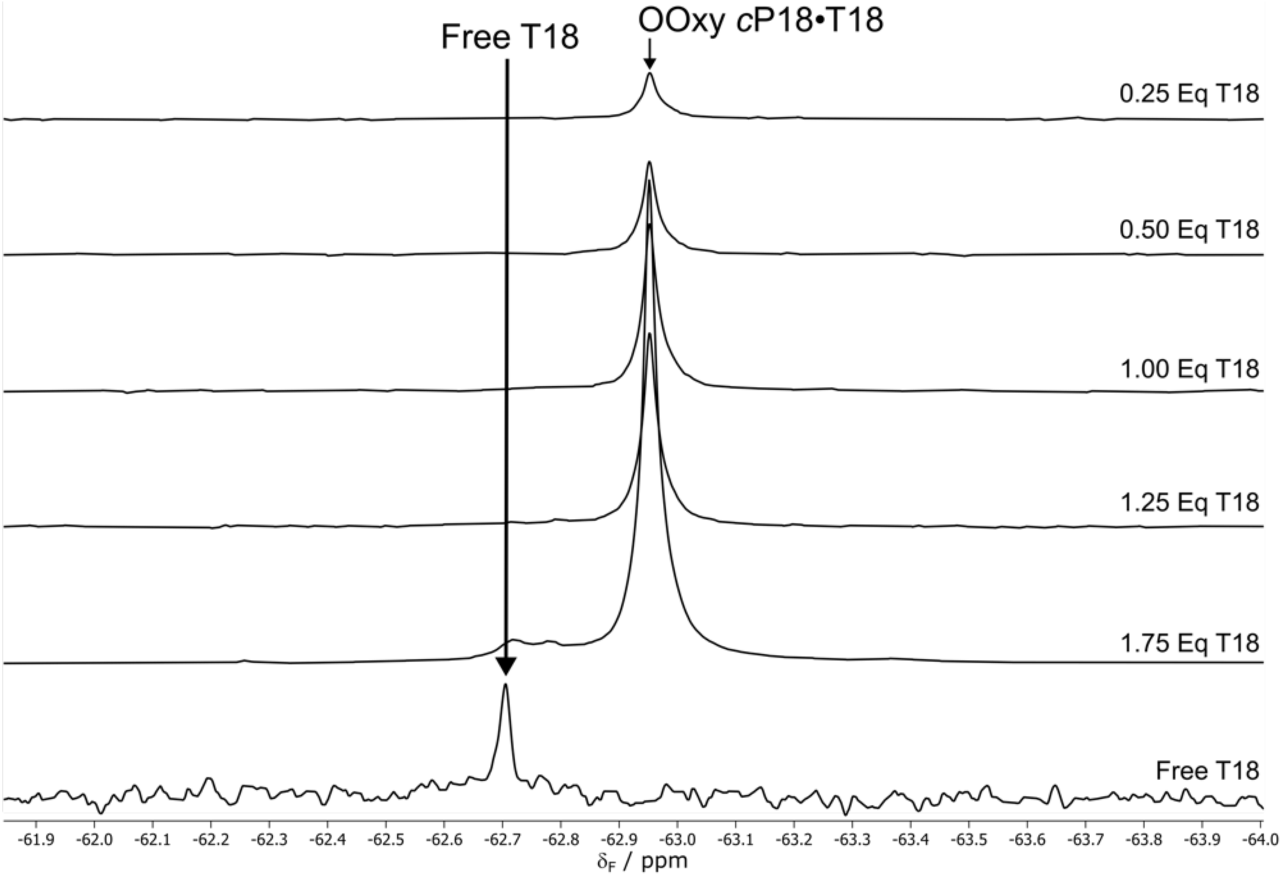

Figure S14. ^{19}F-NMR (471 MHz, $CDCl_3$) spectra from a formation titration of ***c*-P18**$_{\mathbf{OOct}}$**·T18**$_{\mathbf{A}}$.

Formation of *c*-P18$_{\mathbf{THS}}$·T18$_{\mathbf{A}}$:

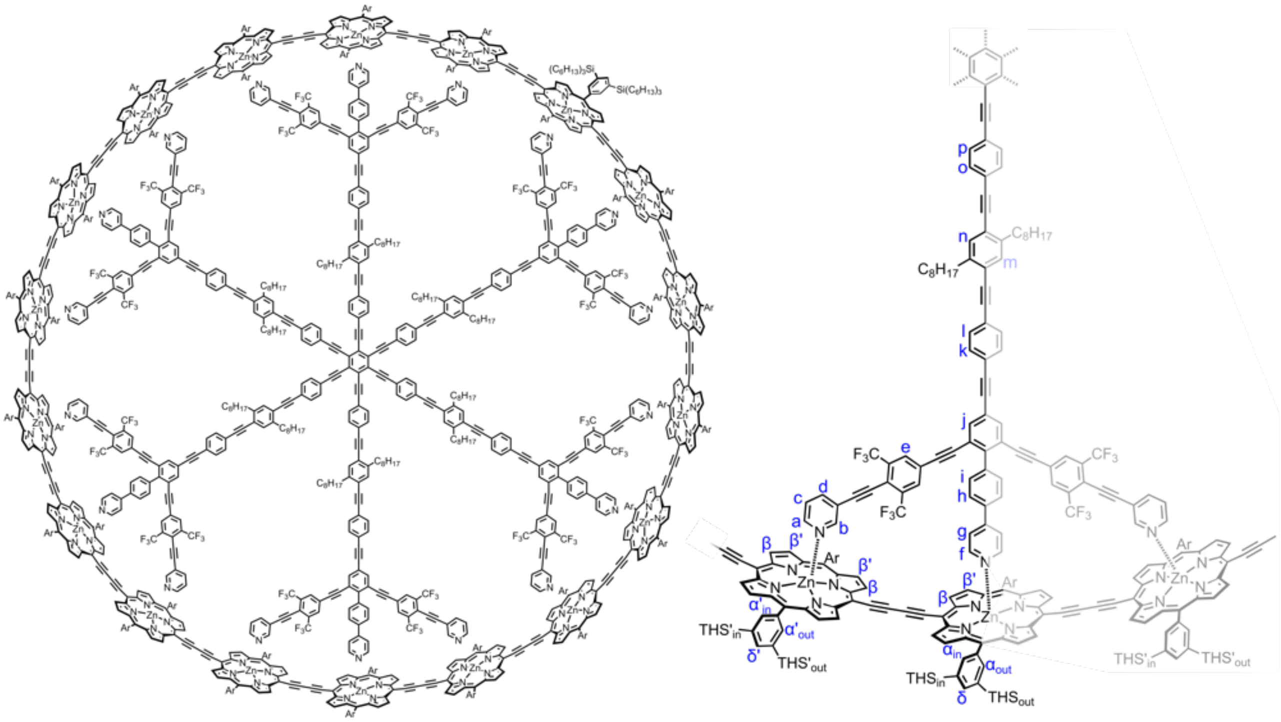

To a dried sample of ***c*-P18$_{\mathbf{THS}}$** (4.04 mg, 1 Eq, 132 nmol) in an NMR tube, was added $CDCl_3$ (450 µL) that had been freshly filtered over Al_2O_3. ^{1}H and ^{19}F NMR spectra were recorded for this sample, and after each addition of **T18$_{\mathbf{A}}$** (1.74 mg, 1.5 Eq, 198 nmol, 6.59×10^{-4} M in $CDCl_3$). The titration was stopped as soon as a the free **T18$_{\mathbf{A}}$** fluorine signal was observed (δ_F = –62.7 ppm). The NMR sample was dried under a stream of nitrogen, then dissolved in 1:1 $CHCl_3$:pentane, and passed over a short plug of silica (SiO_2, 1:1 $CHCl_3$:pentane) to remove excess **T18$_{\mathbf{A}}$**. Concentration of this fraction yielded the ***c*-P18$_{\mathbf{THS}}$·T18$_{\mathbf{A}}$** 1:1 complex as a green film (3.69 mg, 71% yield).

T18 retained on the silica plug was recovered by eluting with $CHCl_3$:MeOH:*t*-BuNH_2 84:15:1.

Characterization of ***c*-P18$_{\mathbf{THS}}$·T18$_{\mathbf{A}}$**:
^{1}H NMR (600 MHz, CD_2Cl_2) δ 9.99 – 9.82 (m, 72H, **H$_\beta$**), 9.08 – 8.92 (m, 72H, **H$_{\beta'}$**), 8.59 – 8.53 (m, 12H, **H$_{\alpha in}$**), 8.38 – 8.28 (m, 48H, **H$_{\alpha'}$**), 8.28 – 8.20 (m, 12H, **H$_{\alpha out}$**), 8.09 (s, 12H, **H$_\delta$**), 8.05 (s, 24H, **H$_{\delta'}$**), 7.69 – 7.61 (m, 24H, **H$_j$**, **H$_p$**), 7.57 – 7.52 (m, 12H, **H$_o$**), 7.48 – 7.42 (m, 24H, **H$_k$**, **H$_l$**), 7.37 – 7.31 (m, 36H, **H$_e$**, **H$_m$**, **H$_n$**), 7.29 – 7.25 (m, 12H, **H$_i$**), 6.84 – 6.80 (m, 12H, **H$_h$**), 6.58 – 6.53 (m, 12H, **H$_d$**), 5.96 – 5.89 (m, 12H, **H$_g$**), 5.74 – 5.68 (m, 12H, **H$_c$**), 2.92 – 2.87 (m, 12H, **H$_f$**), 2.87 – 2.83 (m, 12H, **H$_b$**), 2.82 – 2.68 (m, 36H, **H$_a$**, **Ar-CH$_2$**), 1.68 – 0.73 (m, 2988H, **THS+Octyl**).
^{19}F NMR (470 MHz, CD_2Cl_2, 298K) δ_F = **–**63.21 ppm (referenced against C_6F_6 (δ_F = **–**162.61) as an internal standard).
MALDI-ToF MS *m*/*z* 39468.6 (calculated for $[C_{2508}H_{3456}F_{72}N_{90}Si_{72}Zn_{18}]^{\bullet+}$ = **(M)**$^{\bullet+}$: 39395).
UV-vis-NIR ($CDCl_3$, 298 K) λ_{max} (ε / 10^6 M^{-1} cm^{-1}): 471 (2.767), 488 (2.896), 818 (2.432) nm.

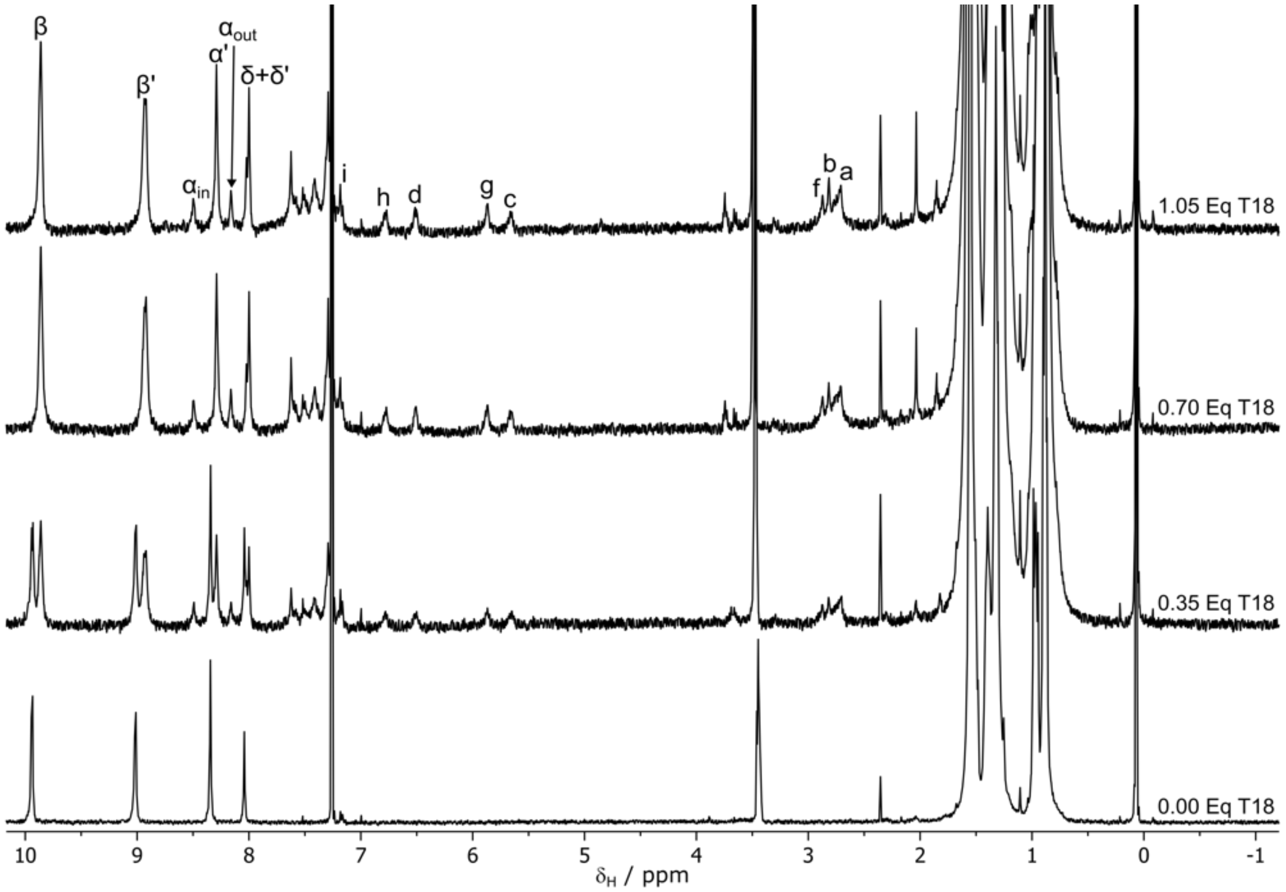

Figure S15. ^{1}H-NMR (400 MHz, $CDCl_3$, 298 K) spectra from a formation titration of ***c*-P18$_{THS}$·T18$_A$**.

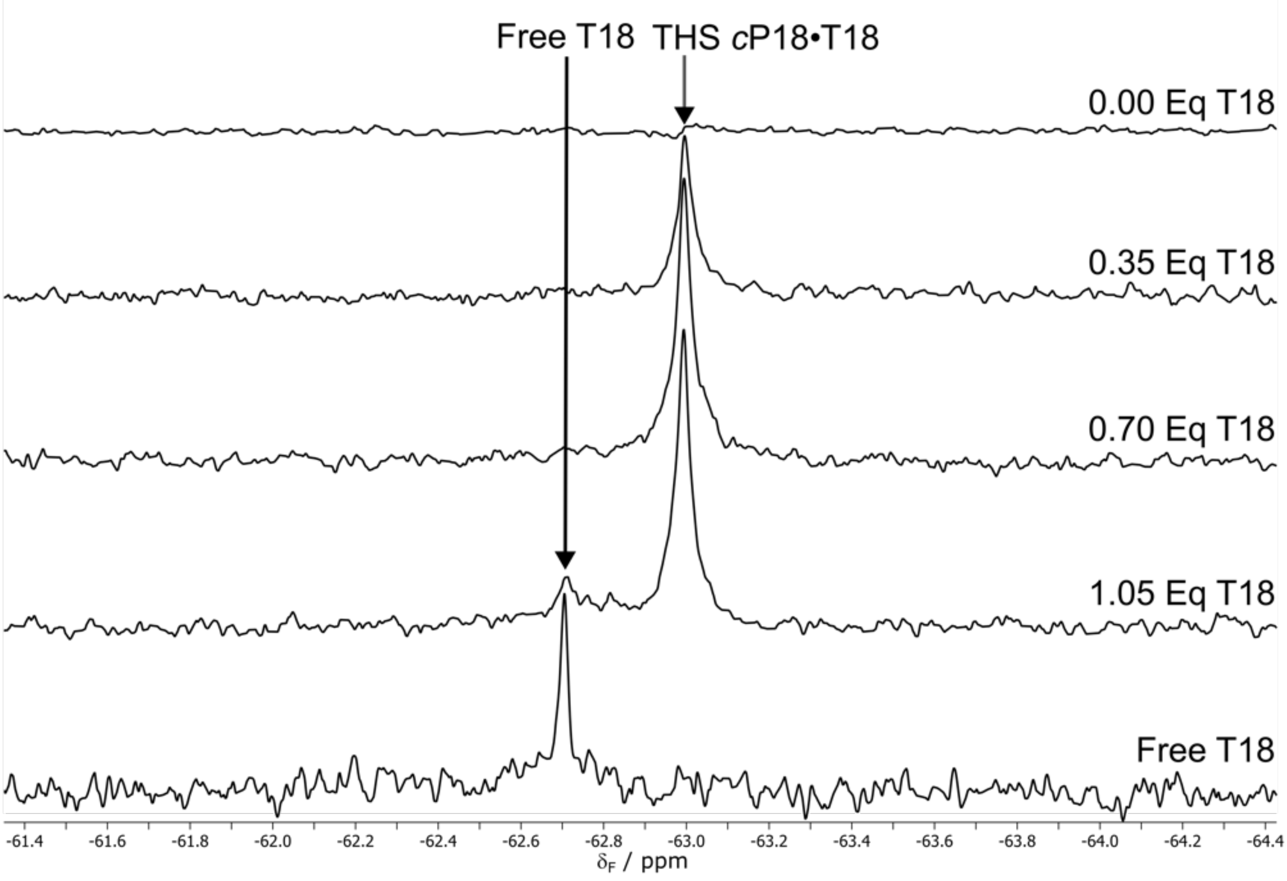

Figure S16. ^{19}F-NMR (377 MHz, $CDCl_3$, 298 K) spectra from a formation titration of ***c*-P18$_{THS}$·T18$_A$**.

Formation of *c*-P18$_{THS}$·T18$_B$:

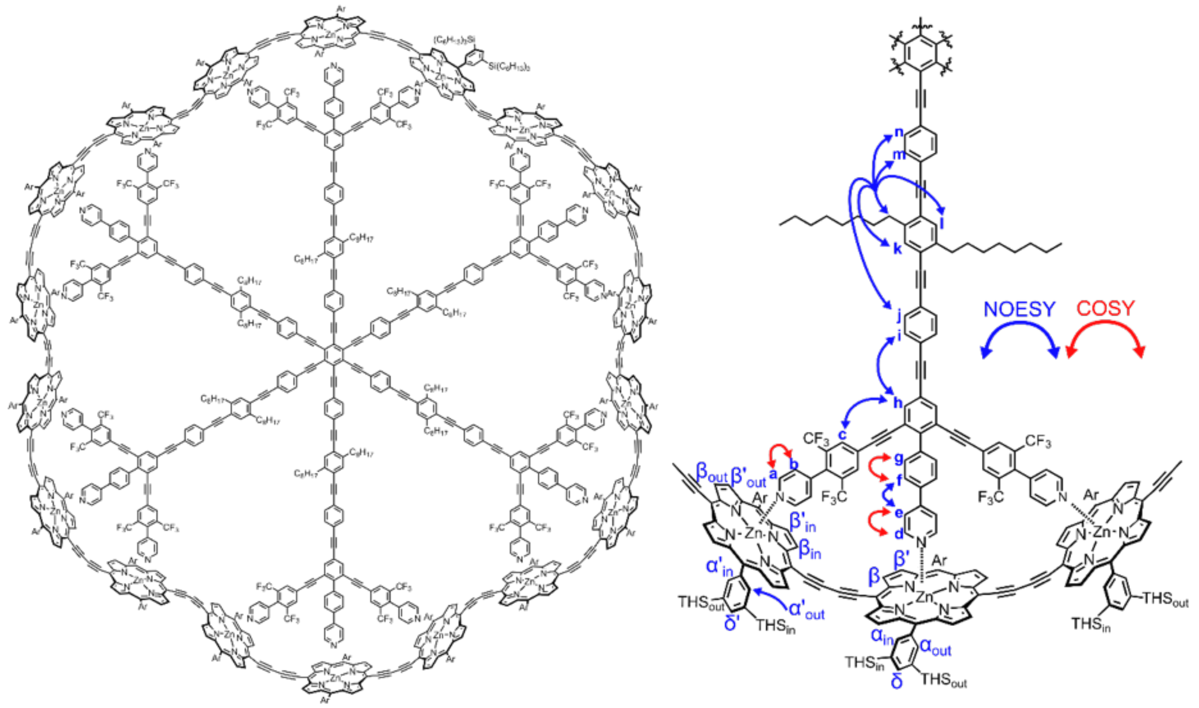

To a dried sample of ***c*-P18$_{THS}$** (0.795 mg, 1 Eq, 26 nmol) in an NMR tube, was added $CDCl_3$ (450 µL) that had been freshly filtered over Al_2O_3. ^{1}H and ^{19}F NMR spectra were recorded for this sample, and after each addition of **T18$_B$** (1.10 mg, 1.1 Eq, 29 nmol, 1.77×10^{-4} M in $CDCl_3$). The titration was stopped as soon as a the free **T18$_B$** fluorine signal was observed. The NMR sample was dried under a stream of nitrogen, then dissolved in 1:1 $CHCl_3$:pentane, and passed over a short plug of silica (SiO_2, 1:1 $CHCl_3$:pentane) to remove excess **T18$_B$**. Concentration of this fraction yielded the ***c*-P18$_{THS}$·T18$_B$** 1:1 complex as a green film (0.77 mg, 76% yield).

T18$_B$ retained on the silica plug was recovered by eluting with $CHCl_3$:MeOH:*t*-BuNH_2 84:15:1.

Characterization of ***c*-P18$_{THS}$·T18$_B$**:
^{1}H NMR (600 MHz, CD_2Cl_2) δ 9.96 (s, 4H, **H$_{\beta out}$**), 9.86 (s, 4H, **H$_\beta$**), 9.74 (s, 4H, **H$_{\beta in}$**), 9.03 (s, 4H, **H$_{\beta' out}$**), 8.95 (s, 4H, **H$_{\beta'}$**), 8.92 (s, 4H, **H$_{\beta' in}$**), 8.55 (s, 2H, **H$_{\alpha in}$**), 8.40 (s, 8H, **H$_{\alpha'}$**), 8.21 (s, 2H, **H$_{\alpha out}$**), 8.11 (s, 6H, **H$_\delta$**, **H$_{\delta'}$**), 7.61 – 7.55 (m, 2H, **H$_{i/j/m/n}$**), 7.53 (s, 2H, **H$_h$**), 7.48 (d, J = 8.9 Hz, 2H, **H$_{i/j/m/n}$**), 7.39 – 7.31 (m, 4H, **H$_{i/j/m/n}$**), 7.28 (s, 1H, **H$_{k/l}$**), 7.25 (s, 1H, **H$_{k/l}$**), 7.19 – 7.14 (m, 2H, **H$_f$**), 7.12 (s, 4H, **H$_c$**), 6.74 – 6.64 (m, 2H, **H$_g$**), 5.81 – 5.73 (m, 2H, **H$_e$**), 5.45 – 5.38 (m, 4H, **H$_b$**), 2.92 – 2.86 (m, 2H, **H$_d$**), 2.87 – 2.79 (m, 4H, **H$_a$**), 2.75 – 2.62 (m, 4H, **Ar-CH$_2$**), 1.65 – 0.79 (m, 2988H, **THS+Octyl**).
^{19}F NMR (470 MHz, $CDCl_3$, 298K) δ_F = –58.67 ppm (referenced against C_6F_6 (δ_F = –161.64) as an internal **standard).**
MALDI-ToF MS *m*/*z* 39279.252 (calculated for $[C_{2484}H_{3456}F_{72}N_{90}Si_{72}Zn_{18}]^{\bullet+}$ = $(M)^{\bullet+}$: 39146.025).
UV-vis-NIR ($CDCl_3$, 298 K) λ_{max} (ε / 10^6 M^{-1} cm^{-1}): 496 (2.883), 870 (3.798) nm.

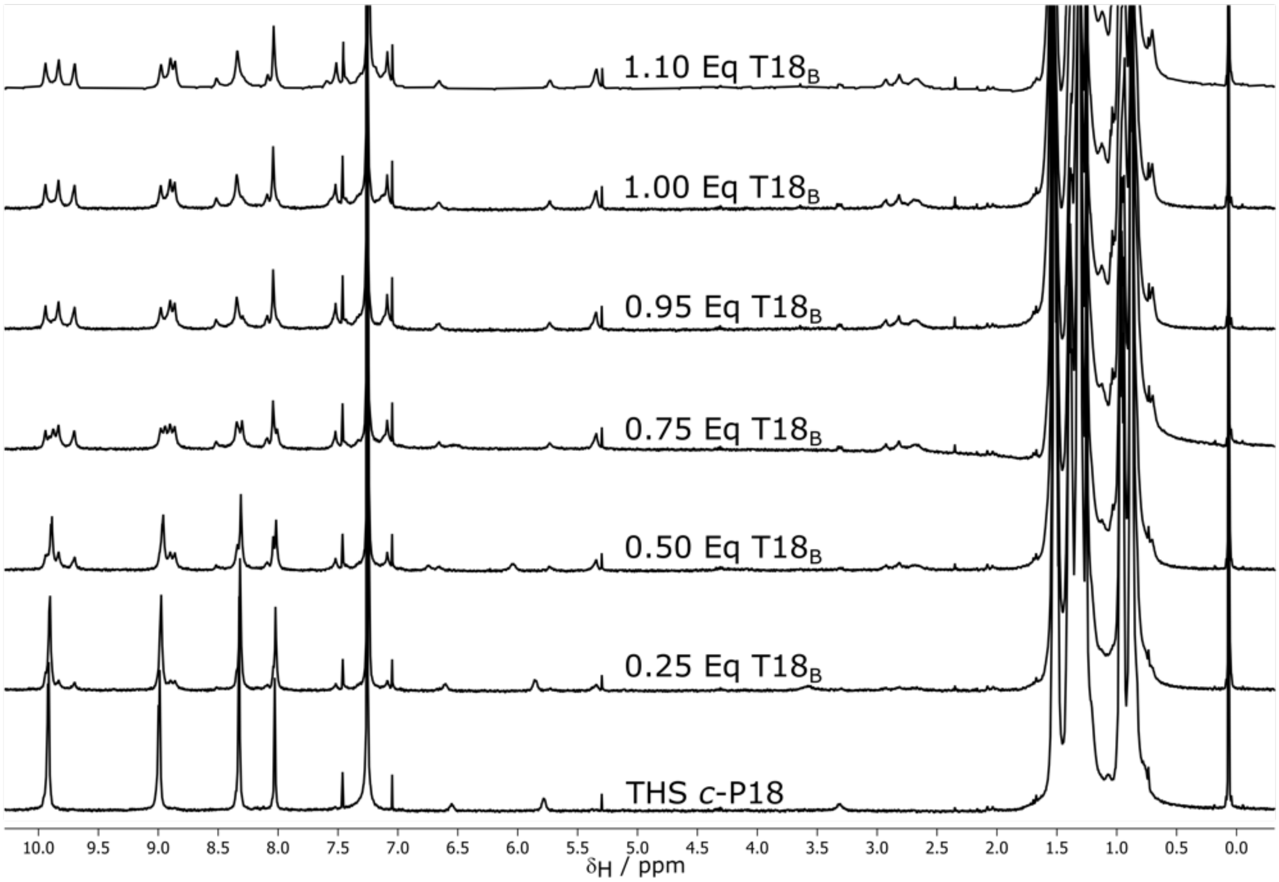

Figure S17. ^{1}H-NMR (400 MHz, $CDCl_3$, 298 K) spectra from a formation titration of ***c*-P18$_{THS}$·T18$_B$**.

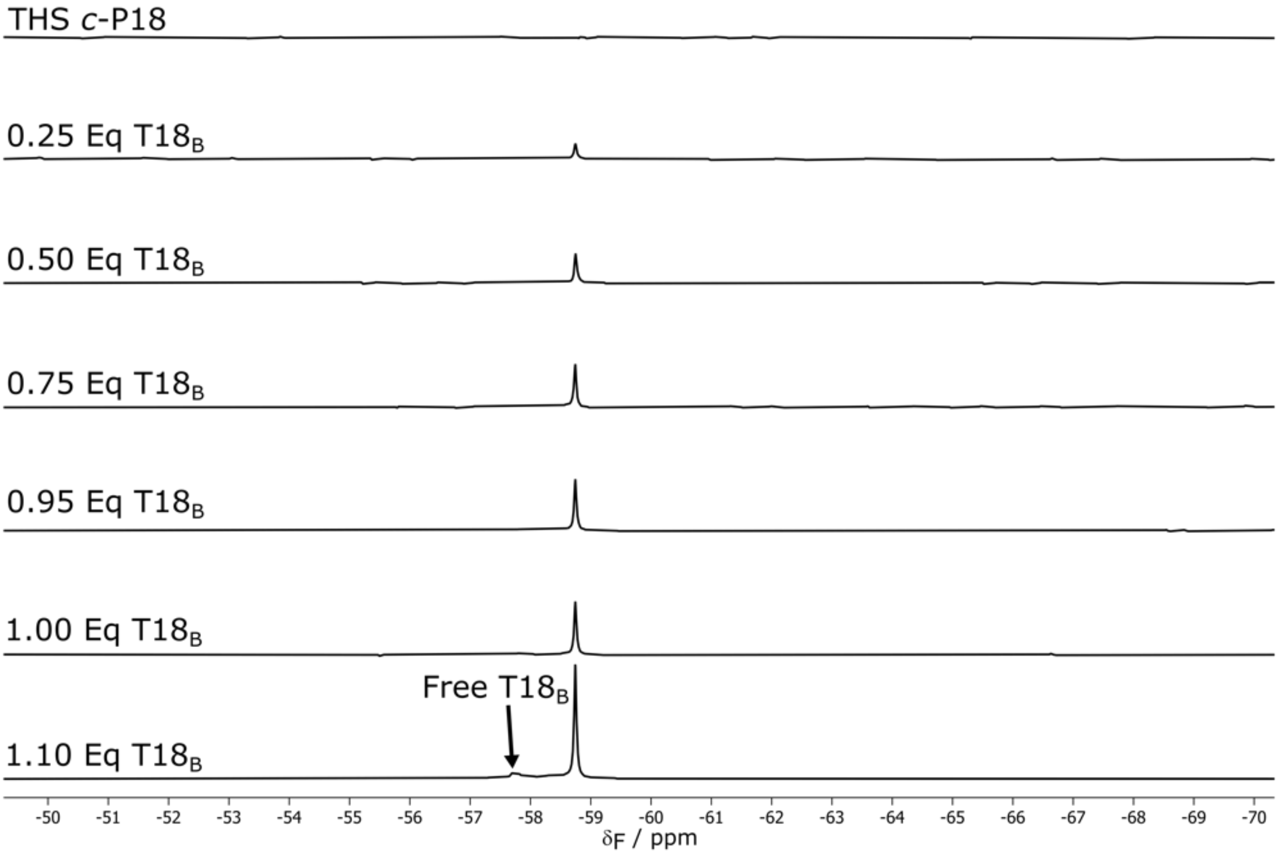

Figure S18. ^{19}F-NMR (377 MHz, $CDCl_3$, 298 K) spectra from a formation titration of ***c*-P18$_{THS}$·T18$_B$**.

Section 7. Scanning probe microscopy

STM parameters. All STM images were acquired with an Omicron STM-1 system operating under ultra-high vacuum (UHV) conditions with a base pressure of 2×10^{-9} mbar. Images were acquired at room temperature in constant current mode using electrochemically etched tungsten tips, coated in gold during tip optimization. Image acquisition parameters (sample bias and current set-point) are stated within figure captions. Au(111) on mica surfaces (Georg Albert PVD GmbH) were prepared by cycles of Ar ion sputtering (0.75 keV for 20 min at a pressure of 9×10^{-6} mbar) and annealing (~500 °C, 5 min).

Electrospray deposition. ***c*-P18** was deposited upon a clean Au(111) substrate via electrospray ionization (Molecularspray Ltd, UHV4i source). A solutions of ***c*-P18** (100 µg/mL) in toluene/methanol (3:1 volume ratio) was deposited using the method described in our previous work,[10,13,28] maintaining a chamber pressure of between 1×10^{-7}–2.5×10^{-7} mbar within the deposition chamber by varying the solvent flow rate between 0.1 and 0.03 mL/hour and using an emitter potential of between 1.2 and 2 kV (relative to the entrance capillary). Sub-monolayer coverages were achieved, as determined by regular XPS measurement of the C 1*s* and Au 4*f* regions and compared with prior depositions where the presence of a sub-monolayer coverage had been characterized by STM. Samples were transported between the STM and XPS UHV systems using a vacuum suitcase operating at a pressure of $< 1 \times 10^{-10}$ mbar.

Nanoring characterization via STM. The dimension of the nanoring structures formed from ***c*-P18** were measured from line profiles acquired along the long- and short axis of the rings (as shown in Figure S19**)**. Peak-to-peak measurements were obtained to determine the separation between the features corresponding to the position of the nanoring circumference. Measurements were performed on data acquired with both 'forward' and 'backward' scan directions. An average of the two measurements was taken, to minimize the effect of drift. The circumference of the rings was estimated using the Ramanujan approximation for the circumference of an ellipse:

$$C \approx \pi[3(a+b) - \sqrt{(3a+b)(a+3b)}],$$

where *C* is the circumference of the ellipse, with *a* and *b* being the radii of the long- and short-axis of the ellipse, respectively.

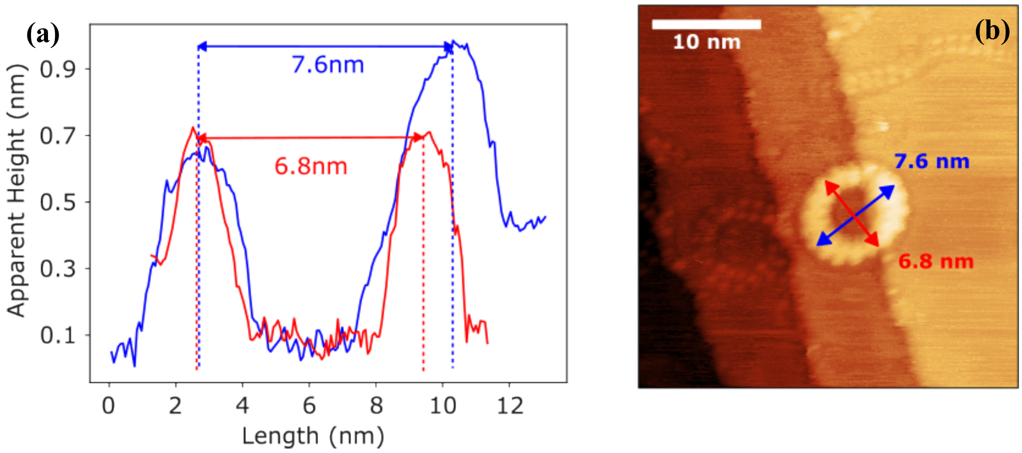

Figure S19. Dimensions of the nanoring structures formed from ***c*-P18**. (a) Line profiles acquired for the long and short axis of a nanoring structure following deposition on Au(111); line profile locations are shown within the forwards scan of the STM image, (b) STM image (bias = –2 V, set-point current = 18 pA). Peak-to-peak separation on the blue and red line profiles are 7.6 nm and 6.8 nm respectively. These measurements were averaged with the same measurements taken on the backwards scan to produce a drift-compensated measurement for *a* and *b*.

The measured dimensions for several ($n = 35$) ***c*-P18** structures (corresponding to 'stacks' of nanorings and single-height species – see below) following electrospray deposition on Au(111) are shown in Figure 6c (main text). For ***c*-P18**, the average dimensions of the nanoring stacks were measured to be $a = 3.6 \pm 0.2$ nm and $b = 2.9 \pm 0.3$ nm, with the average circumference calculated as $C = 21 \pm 1$ nm. The average dimensions of the single-height nanorings were measured to be $a = 3.7 \pm 0.3$ nm and $b = 2.7 \pm 0.4$ nm, with the average

circumference calculated as $C = 20 \pm 1$ nm. The figure highlights the distribution of nanoring dimensions, alongside the average circumference and black solid/ dashed lines representing the flattening factor f:

$$f = \frac{a-b}{a}.$$

From Figure 6c (main text), it can be seen that stacks of ***c*-P18** have a flattening factor of approximately f = 0.2. For single-height rings flattening factors greater than 0.4 are observed.

Sub-monolayer coverage of ***c*-P18** was obtained following electrospray deposition. An overview STM topograph of the surface is shown in Figure S20a. In agreement with our previous work, we observe stacks of nanorings as well as single-height species.[29] Figure S20b shows a stack of ***c*-P18**, a single-height ***c*-P18**, and a linear section of material assigned to a broken **c-P18** nanoring (indicated by black, white, and red arrows, respectively). Within high resolution STM topographs, individual porphyrin units can be resolved within a single ring (Figure S20c): 18 porphyrin units can be clearly identified. The apparent height of the single nanoring and stack are shown in Figure S21: The difference in apparent height between the structures suggests that the stack is formed from two ***c*-P18** rings.[29]

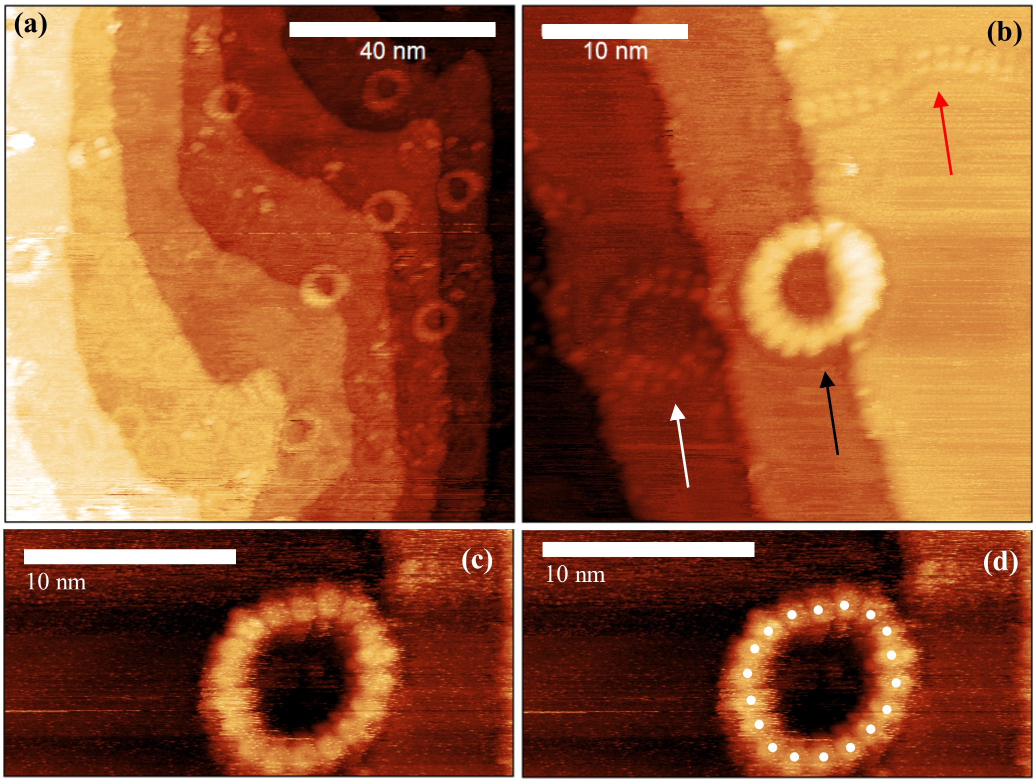

Figure S20. STM topographs of ***c*-P18** on Au(111) following electrospray deposition. (a) Overview image showing the distribution of ***c*-P18** on Au(111) following deposition. (b) High resolution image of ***c*-P18** showing the difference in contrast between a 'stack' of rings (black arrow) and a single ring (white arrow); a linear section of a broken ring (red arrow) is also shown. (c) High resolution image of stack of ***c*-P18** where the individual porphyrin units of the ring can be identified (positions of the porphyrin units are indicated in (d)). [Image parameters: bias = –2 V, set point current = 18 pA.]

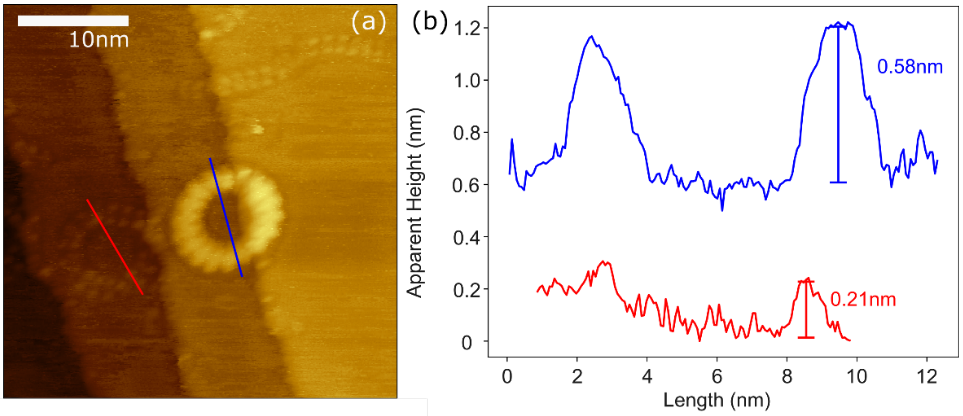

Figure S21. Comparison between a single-height ***c*-P18** nanoring and a stack. (a) STM topograph of ***c*-P18** on Au(111) following electrospray deposition. The position of two line-profiles for a single ***c*-P18** species (2) and a stack (1) are indicated: Line profiles showing the apparent height are shown in (b). [Image parameters: bias = –2 V, set point current = 18 pA.]

Section 8. UV-visible titrations

UV-vis titrations were performed at 298 K using Ag-stabilized $CDCl_3$, which was filtered over aluminum oxide prior to use. In all titrations, the concentration of porphyrin species in the cuvette was kept constant by preparing titrant solutions with the same porphyrin concentration as in the cuvette.

8.1. UV-vis formation titration of $\mathbf{P1_{THS}}$·**Py**

The formation constant of $\mathbf{P1_{THS}}$·**Py** was measured by UV-vis titration of pyridine into the porphyrin monomer (two runs: Figures S23 and S24).

The raw UV-vis data from each titration were fitted to the 1:1 binding isotherm (Eq. 1):

$$\frac{A-A_0}{A_f-A_0} = \frac{(K_f([\mathrm{H}]_0+[\mathrm{L}]_0)+1)-\sqrt{(K_f([\mathrm{H}]_0+[\mathrm{L}]_0)+1)^2-4K_f^2[\mathrm{H}]_0[\mathrm{L}]_0}}{2K_f[\mathrm{H}]_0} \qquad \text{(Eq. 1)}$$

where A is the absorbance of the sample at a given point in the titration, at a specific wavelength, A_0 is the absorbance at the start of the titration, and A_f is the asymptotic final absorbance. K_f is the formation constant, $[H]_0$ is the concentration of porphyrin species (here, $\mathbf{P1_{THS}}$) at the start of the titration and $[L]_0$ is the total concentration of ligand (here, pyridine). A, A_0 and A_f can be values at a single wavelength during the titration, or the difference between two wavelengths that give a large change over the course of the titration (here, 647 nm and 622 nm were selected).

From this analysis the formation constant was determined to be, $K_{py} = (1.47 \pm 0.05) \times 10^4\ M^{-1}$.

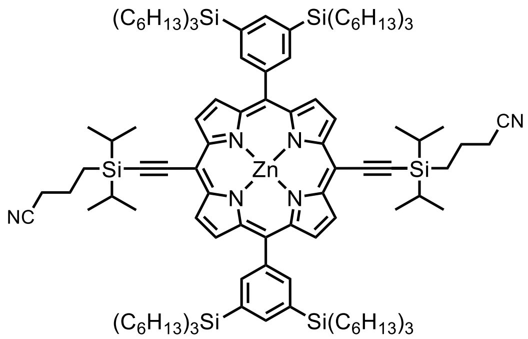

Figure S22. Structure of the porphyrin monomer $\mathbf{P1_{THS}}$ used for the binding titration.

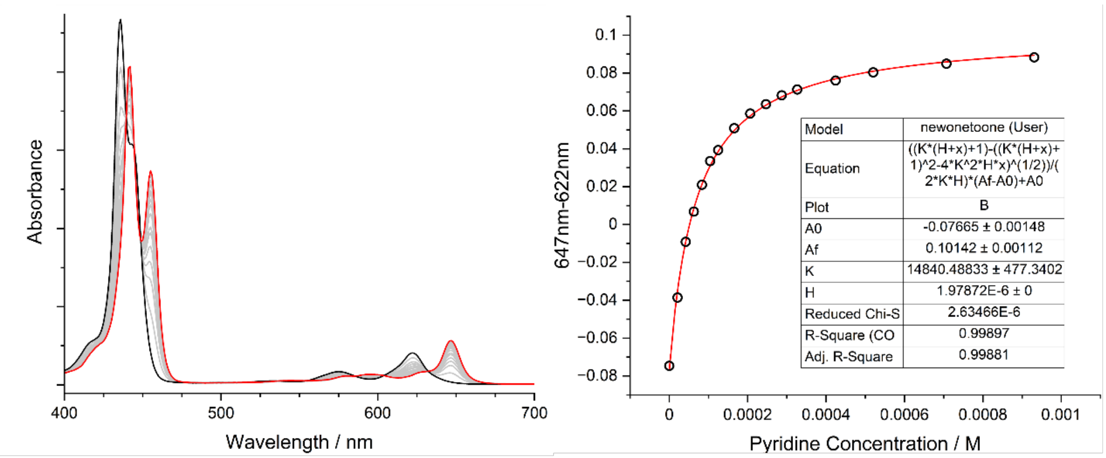

Figure S23. UV-vis titration of pyridine into **P1**$_{\mathbf{THS}}$ ($CDCl_3$, 298 K) (left). Change in Q-band absorbance and fit to a one-to-one binding isotherm, Eq. 1 (right).

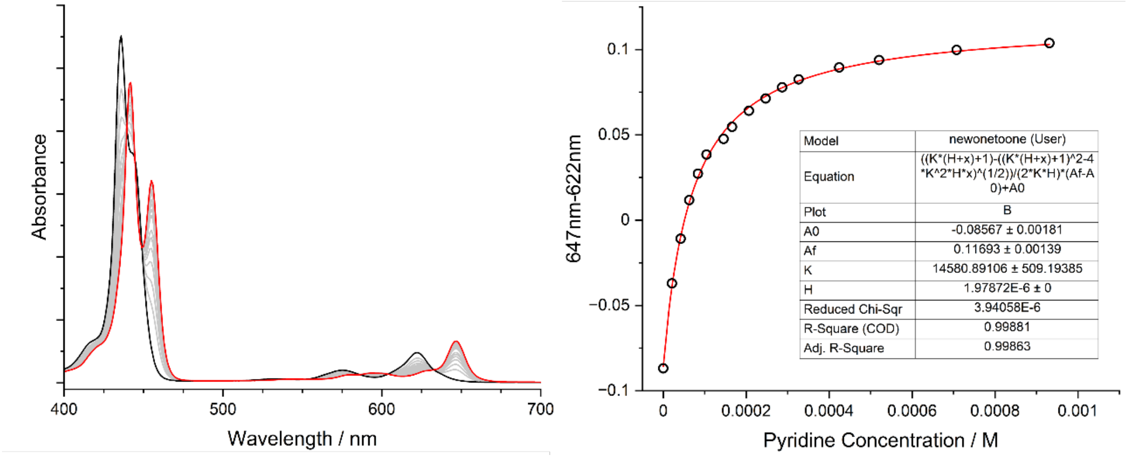

Figure S24. Repeat UV-vis titration of pyridine into **P1**$_{\mathbf{THS}}$ ($CDCl_3$, 298 K) (left). Change in Q-band absorbance and fit to a one-to-one binding isotherm, Eq. 1 (right).

8.2. UV-Vis formation titrations of ***P3***$_{\boldsymbol{THS}}$***·T3***$_{\boldsymbol{A}}$

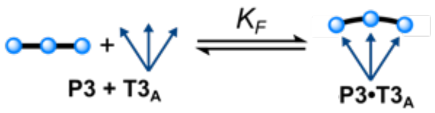

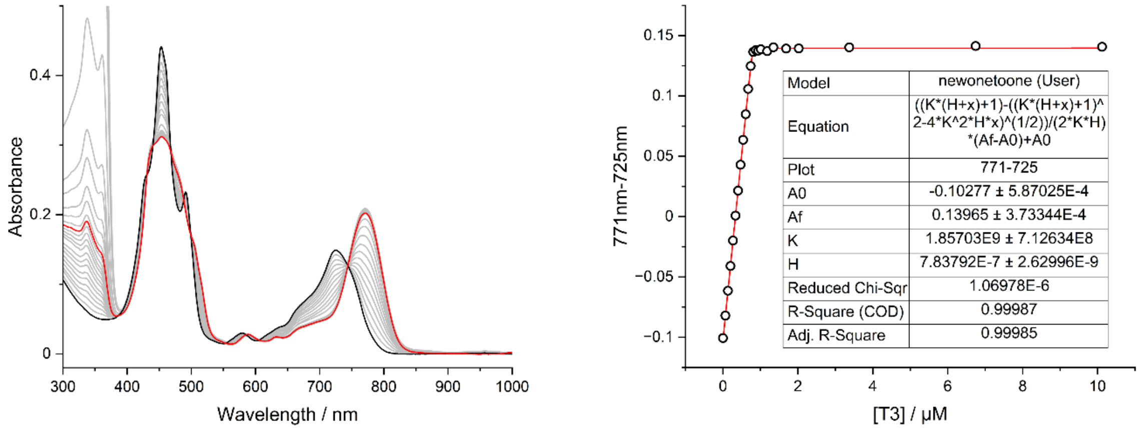

Figure S25. UV-vis titration of **T3**$_{\mathbf{A}}$ into **P3**$_{\mathbf{THS}}$ ($CDCl_3$, 298 K) (left). Change in Q-band absorbance and fit to the one-to-one binding isotherm using Eq. 1 (right).

Fitting the UV-vis data from the formation titration of **P3**$_{\mathbf{THS}}$**·T3**$_{\mathbf{A}}$ to Eq. 1 was used to estimate the formation constant (K_f). However, the binding between **T3**$_{\mathbf{A}}$ and **P3**$_{\mathbf{THS}}$ is very strong ($K_f > 1 \times 10^7$ M^{-1}) and this method only gives a lower limit to the formation constant.

8.3. UV-vis denaturation titration of ***P3***$_{\boldsymbol{THS}}$***·T3***$_{\boldsymbol{A}}$ *with pyridine*

The denaturation of the **P3**$_{\mathbf{THS}}$**·T3**$_{\mathbf{A}}$ complex with pyridine can be monitored by UV-vis spectroscopy, and the data fit to the knockout model shown in Eq. 2, with $n = 3$. Specifically, the change in the Q band absorbance were fit to Eq. 2, where A, A_0 and A_f are defined as in Eq. 1. $[Py]_0$ is the total concentration of pyridine at a given point in the titration, while $[M]_0$ is the initial concentration of porphyrin species (here, **P3**$_{\mathbf{THS}}$**·T3**$_{\mathbf{A}}$**)** and K_{dn} is the denaturation constant. Finally, n is a variable that depends on the number of binding sites between the host and guest; in this case $n = 3$.

$$\frac{A - A_0}{A_f - A_0} = \frac{-K_{dn}[\mathrm{Py}]_0^n + \sqrt{K_{dn}^2[\mathrm{Py}]_0^{2n} + 4K_{dn}[\mathrm{Py}]_0^n[\mathrm{M}]_0}}{2[\mathrm{M}]_0} \qquad \text{(Eq. 2)}$$

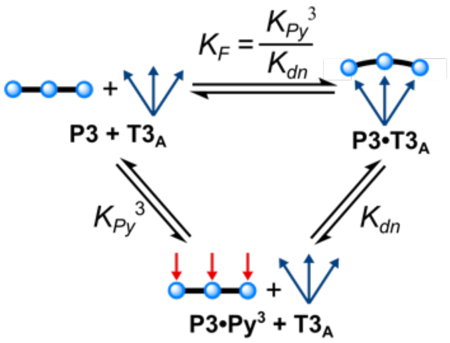

Figure S26. Formation and denaturation of the **P3**$_{\mathbf{THS}}$**·T3**$_{\mathbf{A}}$ complex.

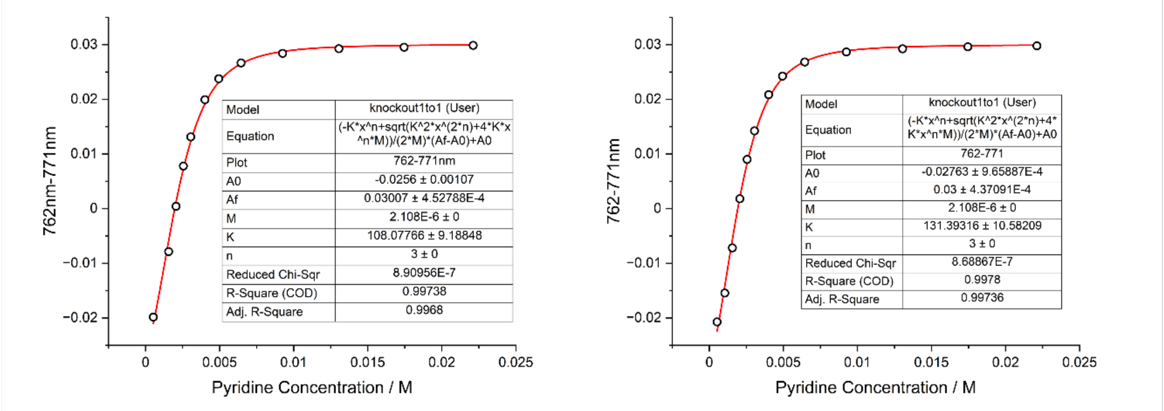

Figure S27. Change in Q band absorbance from UV-vis denaturation titrations of the **P3**$_{\mathbf{THS}}$·**T3**$_{\mathbf{A}}$ complex ($CDCl_3$, 298 K), fit to Eq. 2.

From this analysis the denaturation constant K_{dn} was determined to be 120 ± 15 M^{-2}. This value, along with K_{py} was used to estimate the formation constant of the **P3**$_{\mathbf{THS}}$·**T3**$_{\mathbf{A}}$ complex using Eq. 3 to give $\mathrm{Log}_{10}(K_f) \approx$ 10.4.

$$\mathrm{Log}_{10}(K_f) = 3\ \mathrm{Log}_{10}(K_{py}) - \mathrm{Log}_{10}(K_{dn}) \qquad \text{(Eq. 3)}$$

*8.4. UV-vis formation titration of **P3**$_{\mathbf{THS}}$·**T3**$_{\mathbf{B}}$*

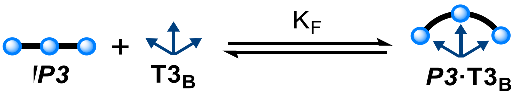

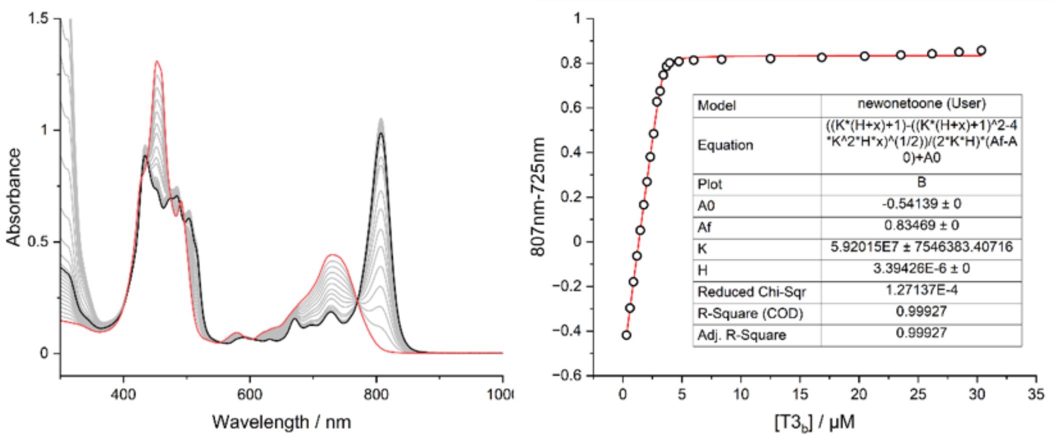

Figure S28. UV-vis titration of **T3**$_{\mathbf{B}}$ into **P3**$_{\mathbf{THS}}$ ($CDCl_3$, 298 K) (left). Binding curve data fit using Eq. 1 (right).

Fitting the UV-vis data from the formation titration of **P3**$_{\mathbf{THS}}$·**T3**$_{\mathbf{B}}$ to Eq. 1 was used to estimate the formation constant (K_f). However, the binding between **T3**$_{\mathbf{B}}$ and **P3**$_{\mathbf{THS}}$ is very strong ($K_f > 1 \times 10^7$ M^{-1}) and this method only gives a lower limit to the formation constant.

*8.5. UV-vis denaturation titration of **P3**$_{\mathbf{THS}}$·**T3**$_{\mathbf{B}}$ with pyridine*

As with **P3**$_{\mathbf{THS}}$·**T3**$_{\mathbf{A}}$, the denaturation of **P3**$_{\mathbf{THS}}$·**T3**$_{\mathbf{B}}$ was monitored by UV-vis spectroscopy and the data were fitted to Eq. 2 to determine the denaturation constant K_{dn}.

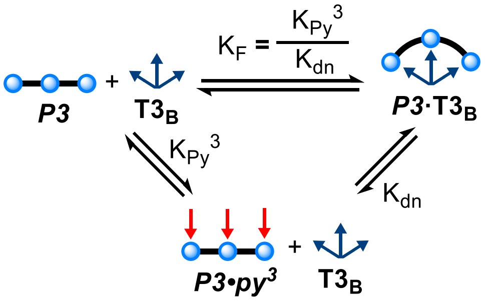

Figure S29. Formation and denaturation of the **P3**$_{\mathbf{THS}}$**·T3**$_{\mathbf{B}}$ complex

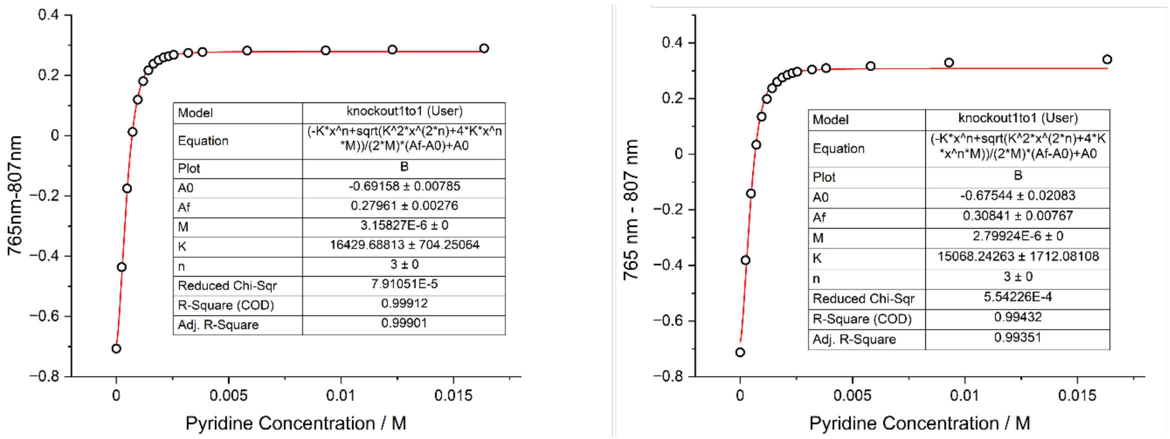

Figure S30. Change in Q band absorbance from UV-vis denaturation titrations of the **P3**$_{\mathbf{THS}}$**·T3**$_{\mathbf{B}}$ complex ($CDCl_3$, 298 K), fit to Eq. 6.2.

From this analysis the denaturation constant K_{dn} is determined to be $(1.6 \pm 0.2) \times 10^4$ M^{-2}. This value, along with K_{py} can then be used to determine the formation constant of the **P3**$_{\mathbf{THS}}$**·T3**$_{\mathbf{B}}$ complex using Eq. 3 to give $\mathrm{Log}_{10}(K_f) \approx 8.3$.

*8.6. Binding competition between **T3**$_{A}$ and **T3**$_{B}$ for **P3**$_{THS}$*

The competition experiment summarized in Figure S31 was used to determine the ratio K_A/K_B (equation 4).

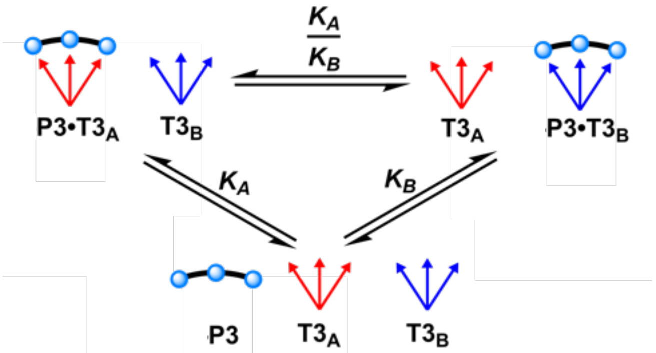

Figure S31. Scheme for binding of **T3**$_{\mathbf{A}}$ and **T3**$_{\mathbf{B}}$ to **P3** used to derive Equation 4.

$$\frac{K_A}{K_B} = \frac{[\mathbf{P3 \cdot T3_A}]}{[\mathbf{P3 \cdot T3_B}]}\frac{[\mathbf{T3_B}]}{[\mathbf{T3_A}]} \qquad \text{(Eq. 4)}$$

This experiment that enables us to test a range of **T3**$_A$: **T3**$_B$ ratios, and sweep from purely **P3**$_{THS}$·**T3**$_A$ to purely **P3**$_{THS}$·**T3**$_B$ in one titration. To set up this experiment two stock solutions were prepared in $CHCl_3$; one containing 2 equivalents of **T3**$_A$ and 1 equivalent of **P3**$_{THS}$, and the other with 2 equivalents of **T3**$_B$ and 1 equivalent of **P3**$_{THS}$. A cuvette was filled with 2.000 mL of the **T3**$_A$ solution and the UV-vis spectrum measured. Aliquots were then removed from the cuvette and replaced with an equal volume of the **T3**$_B$ stock solution. UV-vis spectra were recorded after each replacement until the mole fraction of **T3**$_B$ in solution reached 0.97. Thus, we recorded spectra with the mole fraction of template **T3**$_B$ ranging from 0 to 1, where this mole fraction is defined by Equation 5:

$$\chi_{\mathbf{T3_B}} = \frac{[\mathbf{T3_B}]_0}{[\mathbf{T3_A}]_0+[\mathbf{T3_B}]_0} = \frac{[\mathbf{T3_B}]+[\mathbf{P3{\cdot}T3_B}]}{[\mathbf{T3_A}]+[\mathbf{P3{\cdot}T3_A}]+[\mathbf{T3_B}]+[\mathbf{P3{\cdot}T3_B}]} \qquad \text{(Eq. 5)}$$

The raw UV-vis data from this titration are shown in Figure S32.

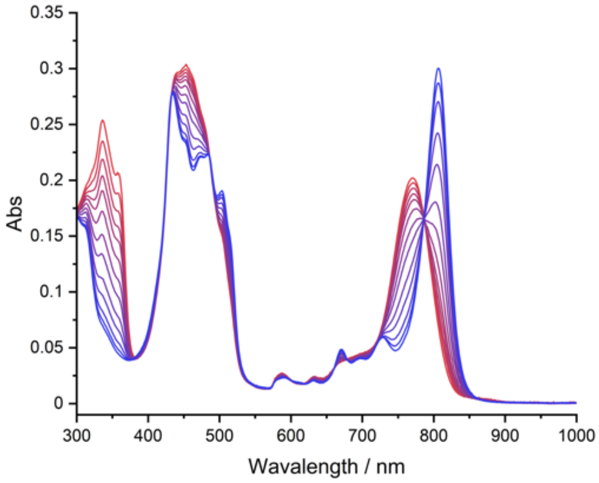

Figure S32. Competition titration between **T3**$_A$ and **T3**$_B$ for **P3**$_{THS}$. (Red trace is pure **P3**$_{THS}$·**T3**$_A$; blue trace is pure **P3**$_{THS}$·**T3**$_B$.) Spectra recorded for solutions in $CHCl_3$ at 298 K.

From the UV-vis spectra of the two stock solutions, a set of calibration curves was produced which shows the predicted UV-vis spectra of the mixtures present during the titration, going from pure **P3**$_{THS}$·**T3**$_A$ to pure **P3**$_{THS}$·**T3**$_B$ in 1% increments. In other words, we calculated the spectra for a mole fraction of the complex **P3**$_{THS}$·**T3**$_B$ ranging from 0 to 1, where this mole fraction is defined by Equation 6:

$$\chi_{\text{complex B}} = \frac{[\mathbf{P3{\cdot}T3_B}]}{[\mathbf{P3}]_0} = \frac{[\mathbf{P3{\cdot}T3_B}]}{[\mathbf{P3}]+[\mathbf{P3{\cdot}T3_A}]+[\mathbf{P3{\cdot}T3_B}]} \qquad \text{(Eq. 6)}$$

The mole fraction (χ_{complexB}) at each point in the titration was then determined by finding the calibration curve with the smallest root-mean square error from the recorded spectrum. χ_{complexB} was then plotted against $\chi_{\mathbf{T3_B}}$ (Figure S33). These data were fitted to Eq 4 to determine K_A/K_B, by calculating curves for different values of K_A/K_B and finding the value that best fits the experimental data. In this analysis, we assume that K_A and K_B are strong enough that **T3** exists entirely as **P3·T3**$_A$ or **P3·T3**$_B$.

This analysis gave $K_A/K_B = 6.25$, which means that a 1:1:1 mixture of **T3**$_A$:**T3**$_B$:**P3** will give 71% **T3**$_A$ bound and 29% **T3**$_B$ bound.

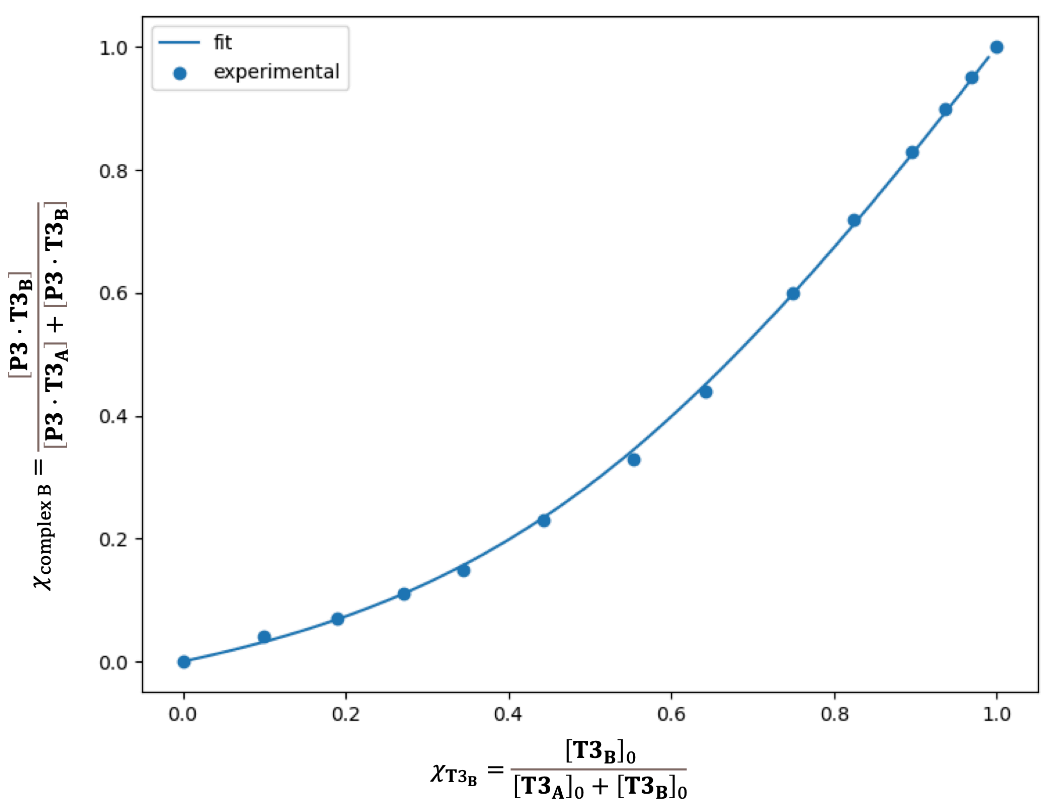

Figure S33. Experimentally determined mole fraction of **P3**$_{\text{THS}}$·**T3**$_{\text{B}}$ (complex B) vs. the mole fraction of template **T3**$_{\text{B}}$ and calculated curve for $K_A/K_B = 6.25$.

*8.7. UV-vis formation titration of **c-P18**$_{THS}$·**T18**$_A$*

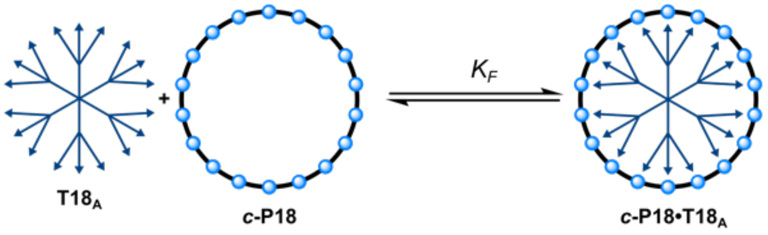

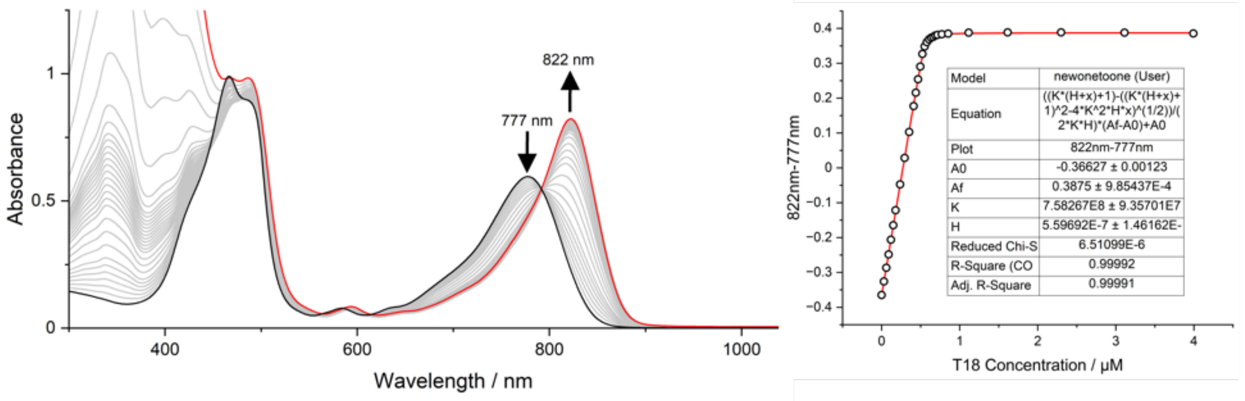

Figure S34. UV-vis titration of **T18**$_{\text{A}}$ into ***c*-P18**$_{\text{THS}}$ ($CDCl_3$, 298 K) (left). Binding curve fitted using Eq. 1. (right).

As with **P3**$_{\text{THS}}$·**T3**$_{\text{A}}$, fitting the UV-vis data from the formation titration of ***c*-P18**$_{\text{THS}}$·**T18**$_{\text{A}}$ to Eq. 1 gives a very strong formation constant ($K_f > 1 \times 10^7\ M^{-1}$).

*8.8. UV-Vis formation titration of **c-P18$_{THS}$·T18$_B$***

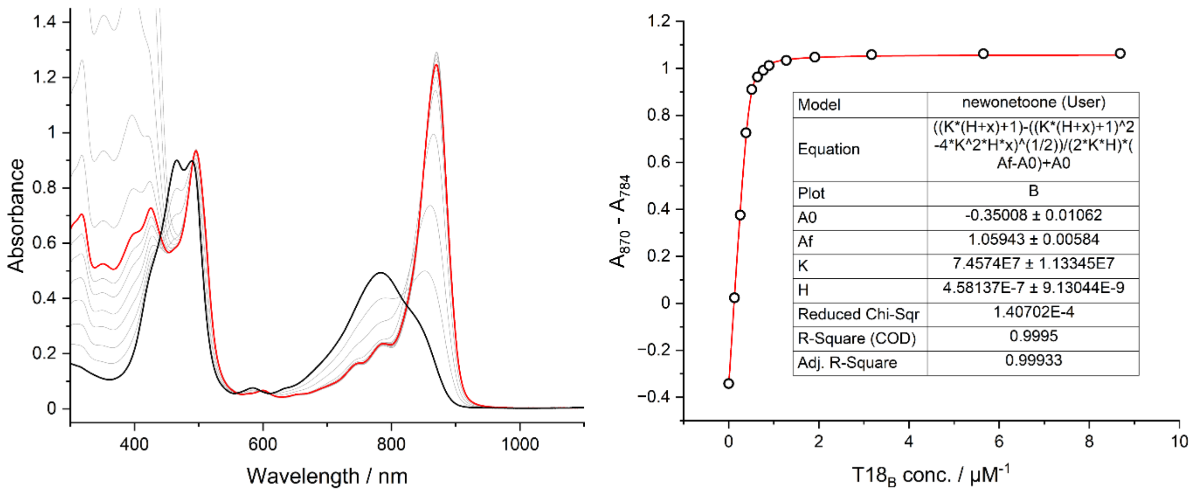

Figure S35. UV-vis titration of **T18$_B$** into ***c*-P18$_{THS}$** ($CDCl_3$, 298 K) (left). Binding curve fitted using Eq. 1. (right).

As with ***c*-P18$_{THS}$·T18$_A$**, fitting the UV-vis data from the formation titration of ***c*-P18$_{THS}$·T18$_B$** to Eq. 1. gives a very strong formation constant ($K_f > 1 \times 10^7$). We attempted to measure K_f for ***c*-P18$_{THS}$·T18$_A$** and ***c*-P18$_{THS}$·T18$_B$** by carrying out denaturation titrations with pyridine, but the resulting binding curves do not fit well to Equation 2 with n = 18. The denaturation of ***c*-P18$_{THS}$·T18$_{A/B}$** with pyridine cannot be modeled as two-state all-or-nothing processes, which makes it difficult to apply the denaturation method to estimate K_f.

*8.9. Binding competition between **T18$_A$** and **T18$_B$** for **c-P18$_{THS}$***

Identical competition titrations to those presented in Section 8.6 were performed using **T18$_A$**, **T18$_B$** and ***c*-P18$_{THS}$**. The raw data from these titrations are displayed in Figure S36 and the fit of the experimental data to Equation 4 is shown in Figure S37. From this analysis, we determined $K_A/K_B = 0.59$. Therefore, in a 1:1:1 mix of **T18$_A$**:**T18$_B$**:***c*-P18$_{THS}$** there is 43% **T18$_A$** bound and 57% **T18$_B$** bound.

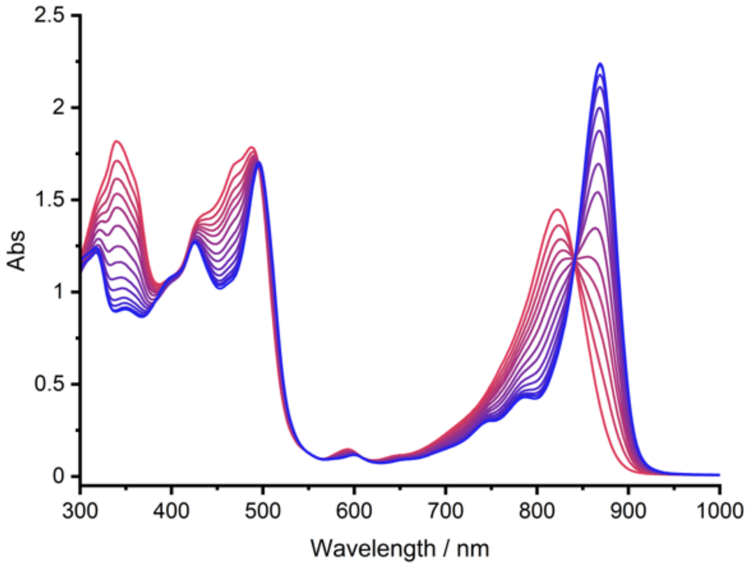

Figure S36. Competition titration between **T18$_A$** and **T18$_B$** for ***c*-P18$_{THS}$**. (Red trace: pure **c-P18$_{THS}$·T18$_A$**; blue trace: pure **c-P18$_{THS}$·T18$_B$**.) Spectra recorded for solutions in $CHCl_3$ at 298 K.

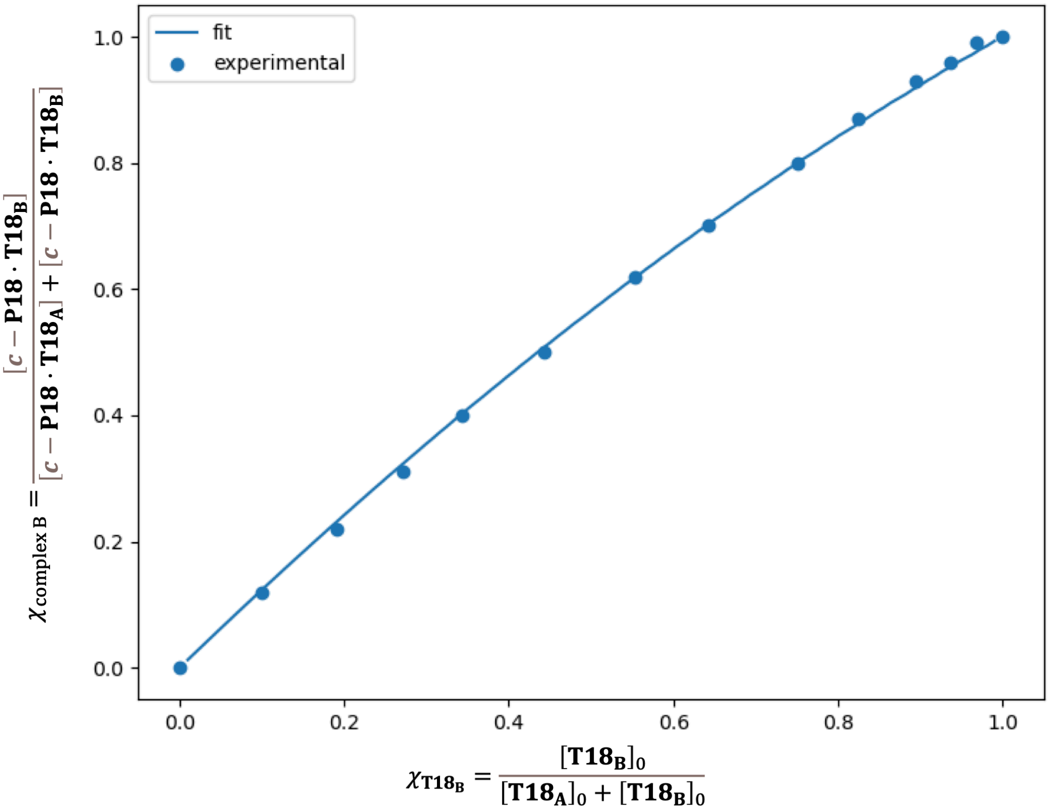

Figure S37. Fit of Equation 4 to the experimentally determined mole fraction of ***c*-P18$_{THS}$·T18$_B$** (complex B) vs. the mole fraction of template **T18$_B$**.

In summary, Sections 8.3, 8.5 and 8.6. indicate that **T3$_A$** binds more strongly to **P3$_{THS}$** than **T3$_B$** (K_A/K_B = 6.25 for **P3$_{THS}$·T3$_{A/B}$**). However, in the nanoring complexes, **T18$_B$** binds more strongly to ***c*-P18$_{THS}$** than **T18$_A$** (K_A/K_B = 0.59 for ***c*-P18$_{THS}$·T18$_{A/B}$**).

Section 9. NICS calculation

The geometry of the template-free ***c*-P18** nanoring was first optimizing in each oxidation state (B3LYP/6-31g(d)), then we overlayed an evenly spaced grid of 101 spectator atoms, directly through the plane of the 18 Zn atoms (Figure S38). NICS calculations on the oxidized nanorings were then performed using either BLYP35 (6-31g(d) or LC-ωhPBE (6-31g(d)) (ω = 0.1) as specified in the figures below, by plotting the negative of the *ZZ* component of the shielding tensor calculated at each spectator atom.

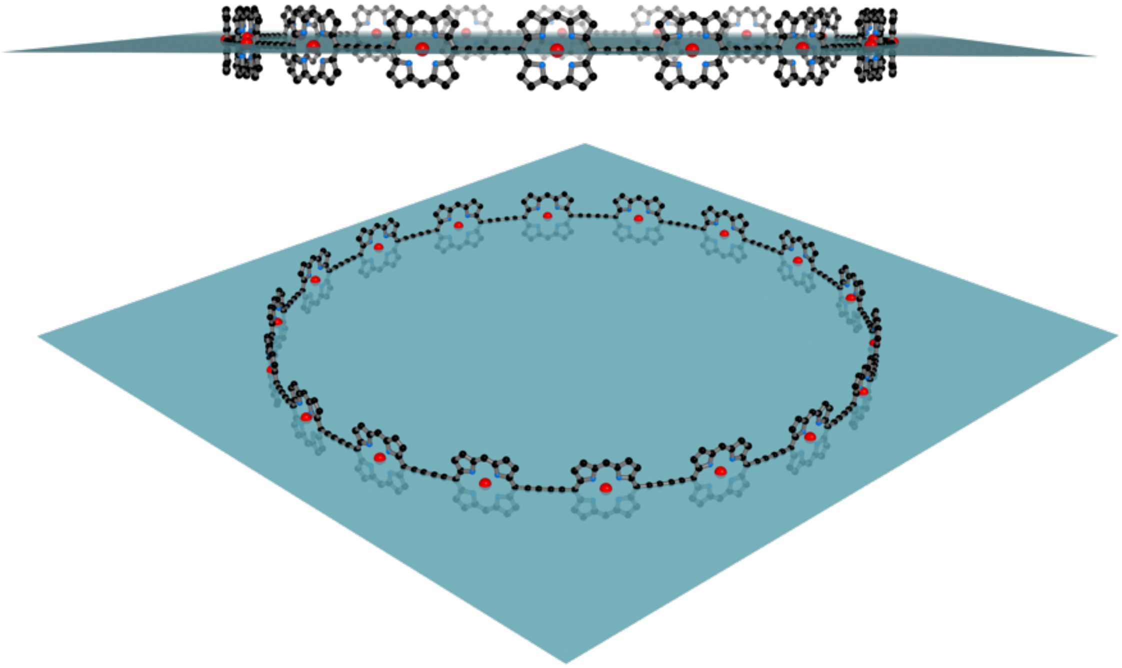

Figure S38. Visualization of the grid of spectator atoms used in NMR calculations. (Spectator atoms identified as the atom type Bz in Gaussian 16.)

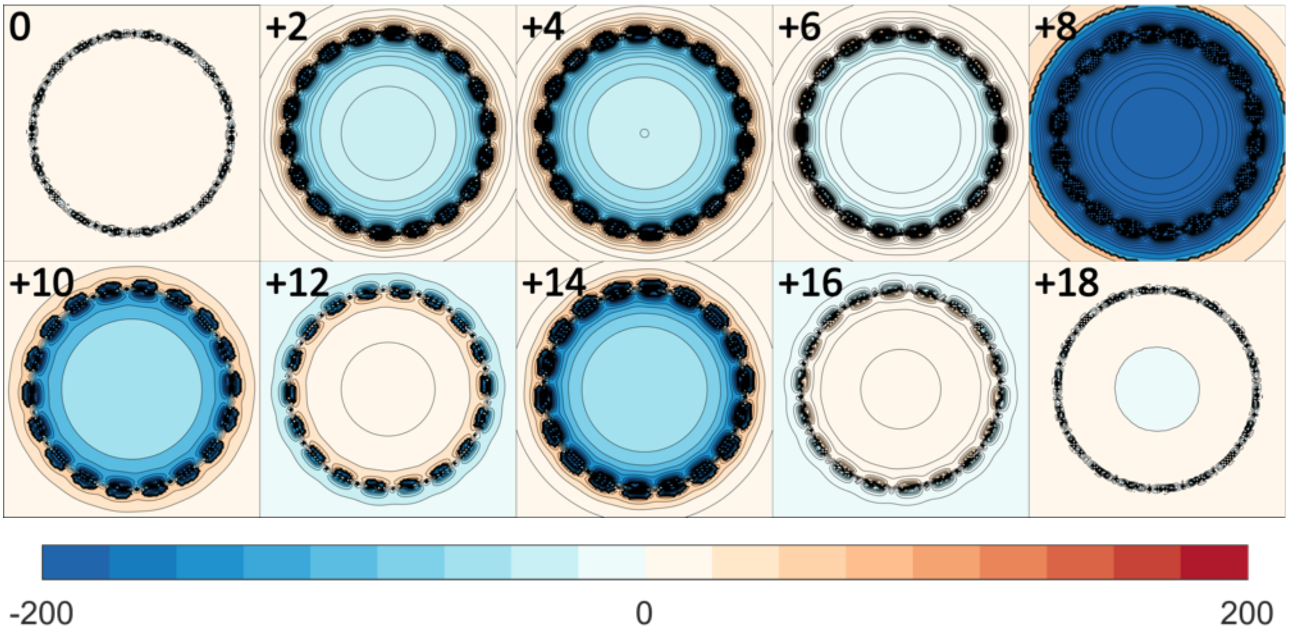

Figure S39. NICS(0)$_{ZZ}$ plots for ***c*-P18** in all even oxidation states from 0 to +18. Nanoring geometries optimized using B3LYP/6-31g(d) and NMR calculations with BLYP35 (6-31g(d)).

The predicted aromaticity of the +4 and +8 states (Figure S39) is not what would be expected from Hückel's rule. As shown in Figure S40, LC-ωhPBE (ω = 0.1) correctly predicts the expected anti-aromaticity of the +8

state, whilst also predicting a more strongly aromatic +6 state and weaker aromaticity in the +2 state. The +4 state however, which is expected to be antiaromatic, is still predicted to be aromatic by the LC-ωhPBE (ω = 0.1) functional.

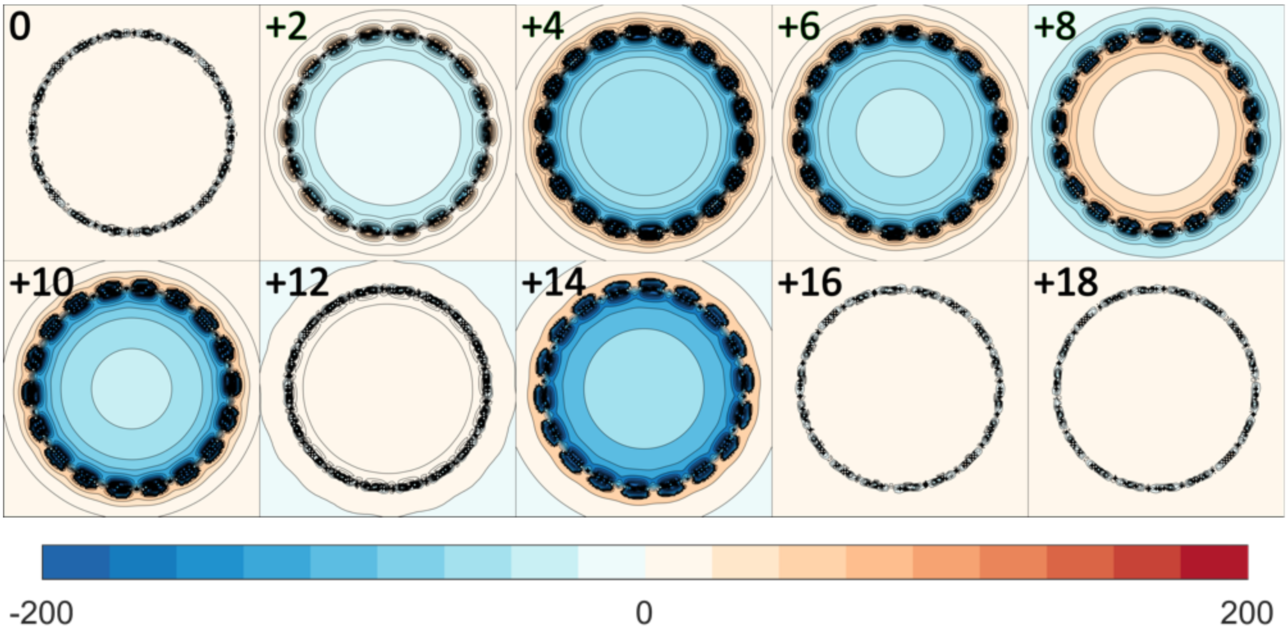

Figure S40. NICS(0)zz plots of ***c*-P18** in all even oxidation states from 0 to +18. Nanoring geometries optimized using B3LYP/6-31g(d) and NMR calculations with LC-ωhPBE (ω = 0.1).

When plotted on a smaller ppm scale (Figure S41), it appears that the 0 and +18 states show local aromaticity at each porphyrin unit, not global aromaticity.

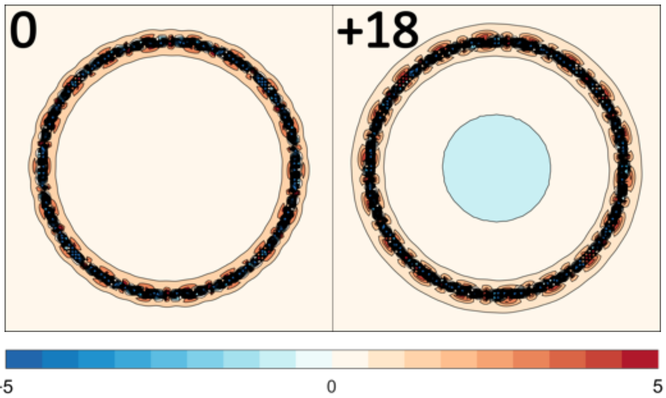

Figure S41. NICS(0)zz plots of ***c*-P18** in the 0 and +18 oxidation states, plotted in the range –5 to +5 ppm. Nanoring geometries optimized using B3LYP/6-31g(d) and NMR calculations with BLYP35/6-31g(d).

Section 10. Oxidative NMR titrations

10.1. Oxidation of ***c*-P18$_{THS}$·T18$_{A}$**

To test for global aromaticity in the ***c*-P18$_{THS}$·T18$_{A}$** complex, a solution of thianthrenium tetrakis(3,5-bis(trifluoromethyl)phenyl)borate (ThnBArF) in $CDCl_3$ was titrated into a solution of ***c*-P18$_{THS}$·T18$_{A}$** in $CDCl_3$ at either 298 K or 233 K. Silver-stabilized $CDCl_3$ was used in this experiment, freshly filtered over aluminum oxide prior to use. The NMR sample of ***c*-P18$_{THS}$·T18$_{A}$** was kept under argon for the duration of the titration, and a solution of ThnBArF was added under a flow of argon. In the case of the 233 K titration, the NMR sample was stored in a dry ice/acetone bath at –78 °C while out of the spectrometer for each addition of ThnBArF.

The end point of the titration was determined by the broadening of the characteristic ^{1}H (7.50 ppm) signal of thianthrene. At this point in the titration, newly added thianthrenium is unable to oxidize the ***c*-P18$_{THS}$·T18$_{A}$** complex higher than +18, so instead it is in rapid exchange with neutral thianthrene present in solution, which broadens the thianthrene signals. The expected oxidation state of the ***c*-P18$_{THS}$·T18$_{A}$** complex at each point in the titration can then be calculated from the equivalents of ThnBArF required to reach the +18 oxidation state, which typically occurs within 3 equivalents of the expected endpoint based on the concentrations of the two compounds (as seen in Figure S42). The ^{1}H and ^{19}F NMR spectra from both titrations are shown below in Figures S42 and S43.

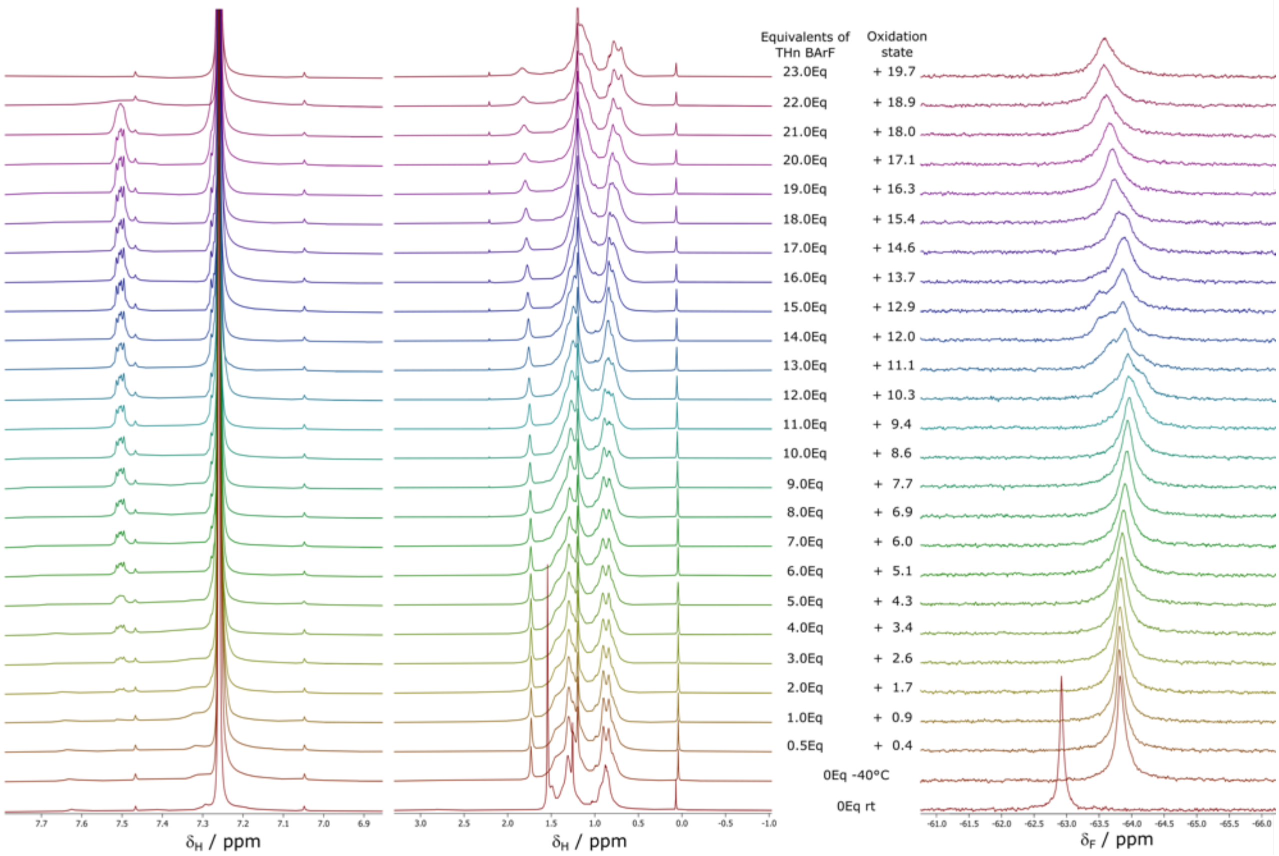

Figure S42. ^{1}H and ^{19}F NMR spectra of ***c*-P18$_{THS}$·T18$_{A}$** at 233 K with increasing equivalents of ThnBArF ($CDCl_3$, ^{1}H 500 MHz / ^{19}F 470 MHz).

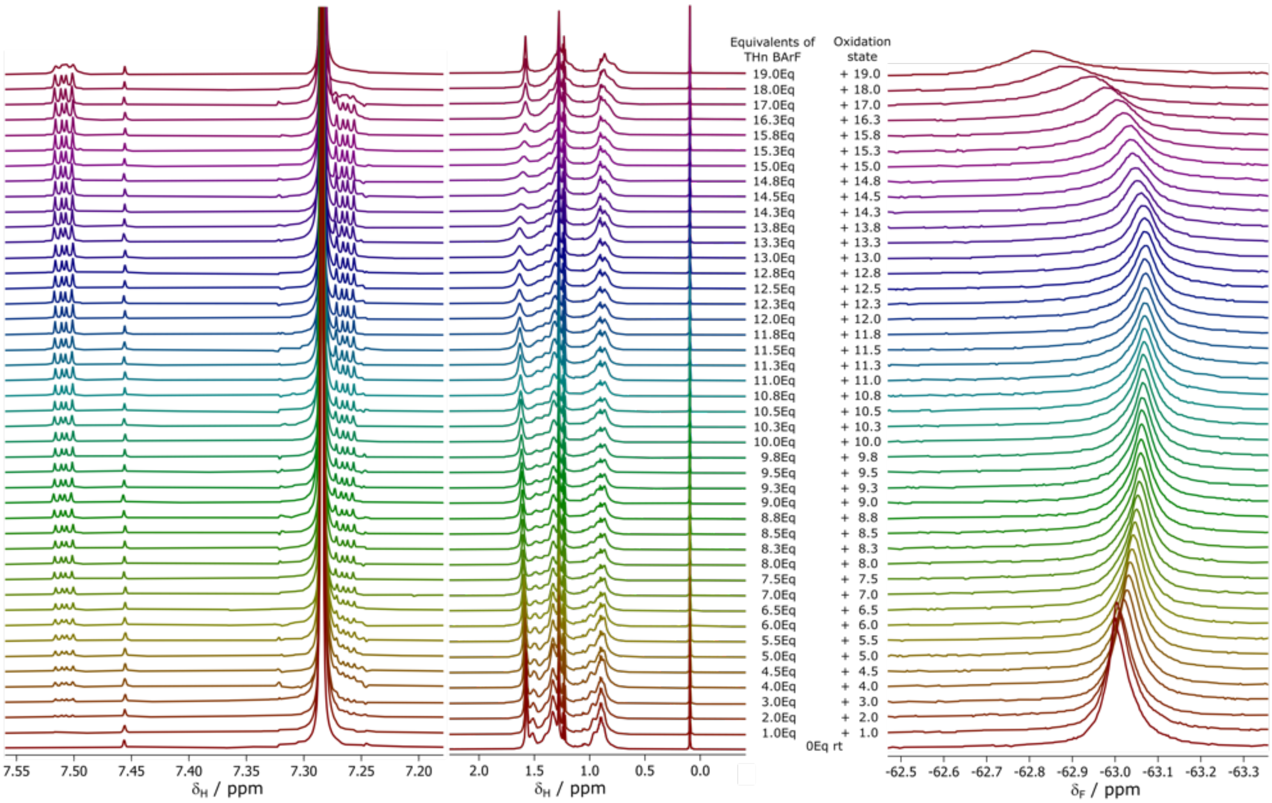

Figure S43. ^{1}H and ^{19}F NMR spectra of ***c*-P18$_{THS}$·T18$_{A}$** at 298 K with increasing equivalents of ThnBArF ($CDCl_3$, ^{1}H 600 MHz / ^{19}F 565 MHz).

In previous studies of global aromaticity in porphyrin-based nanorings, shifts in the ^{19}F NMR spectra during the oxidative titrations were significant enough that that signals from different oxidation states were well resolved, and could be identified and tracked over the titration.[2,3,30] However, the ^{19}F NMR peaks from oxidized ***c*-P18$_{THS}$·T18$_{A}$** are broad and do not show well resolved peaks corresponding to different oxidation states.

We analyzed the ***c*-P18$_{THS}$·T18$_{A}$** 233 K oxidation titration data by deconvolving each spectrum using the Origin software, by fitting multiple Lorentzian peaks to the experimental data (Figure S44). In this process, each ^{19}F NMR spectrum was first cut to a range of –61.0 to –66.5 ppm to isolate the ***c*-P18$_{THS}$·T18$_{A}$** CF_3 signal. The manual deconvolution then started from the spectrum with 12.0 equivalents of oxidant, as this showed two clear oxidation states: +11 at –63.59 ppm and +12 at –63.44 ppm. Inputting four Lorentzian peaks and allowing the fit to optimize identifies these oxidation states, along with the +10 state and a residual peak. From this starting point of 12.0 equivalents, the deconvolution was continued up to 16.3 equivalents and back to +8.6, where we were able to observe the clear build up and decay of the expected oxidation states.

This deconvolution requires every spectrum to have a substantial contribution from a “residual peak” (40% – 100%, Figures S44, S45) which only shifts slightly during the course of the titration and appears to behave like unoxidized complex. As the free **T18$_{A}$** signal comes outside the range of any of the peaks in the titration, we assume that this residual peak comes from **T18$_{A}$** bound to ***c*-P18**. This residual peak gradually becomes deshielded over the course of the titration and it may originate from nanoring complexes in which the conformation does not favor global (anti)aromatic ring currents. This means that each spectrum reflects a collection of nanorings with a distribution of oxidation states, in which some are in appropriate geometries for global ring currents to occur, while others have geometries that favor charge localization giving rise to this “residual peak”.

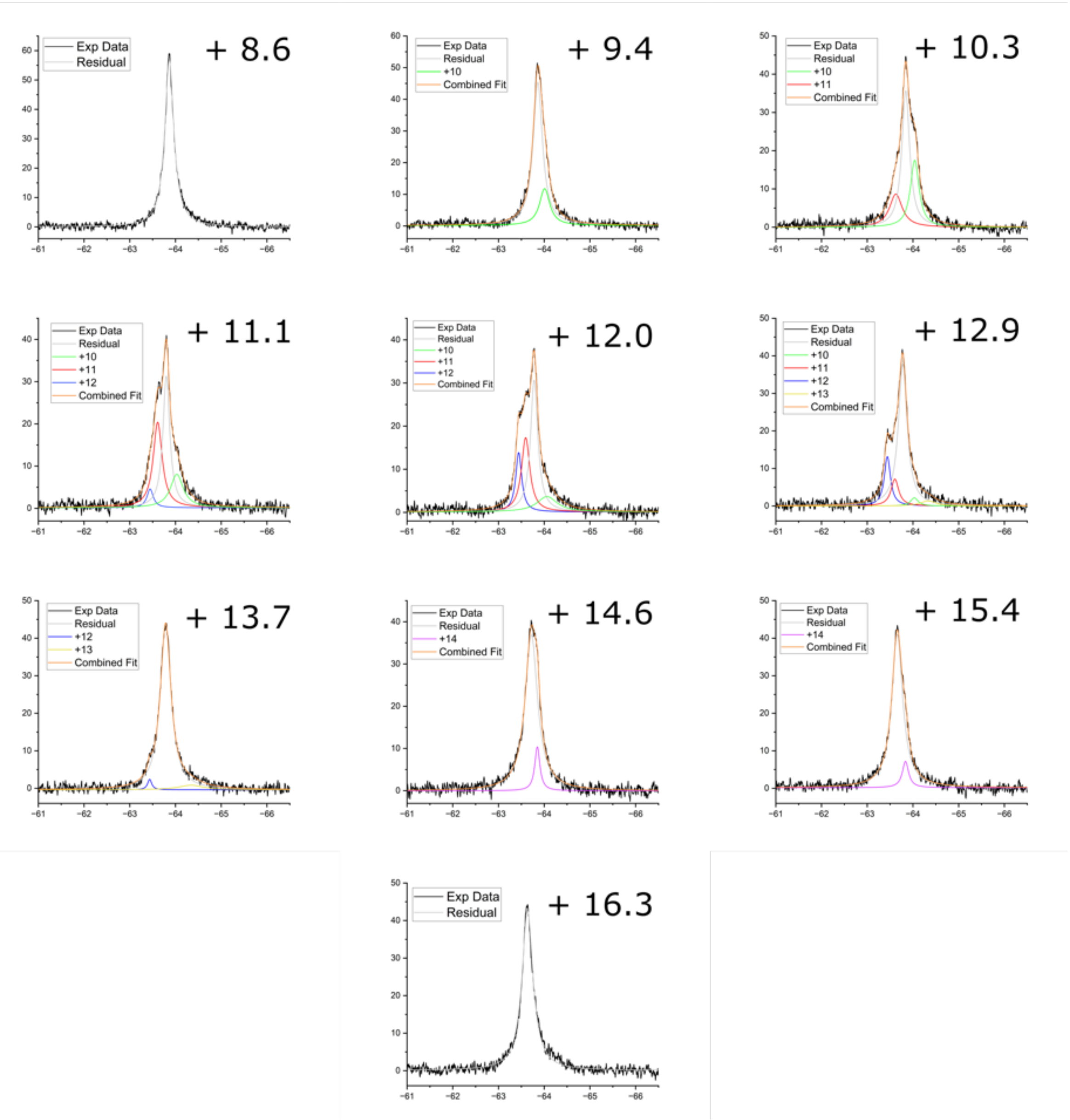

Figure S44. Deconvoluted ^{19}F NMR data from the 233 K oxidative titration of ***c*-P18$_{\mathbf{THS}}$·T18$_{\mathbf{A}}$**. (Data from spectra in Figure S42.) Numbers at the top right of each panel are the equivalents of ThnBArF.

From each deconvoluted spectrum, the relative abundances of each oxidation state and residual contribution can be obtained as the area of the peak describing that state, as a fraction of the total signal. From this analysis (Figure S45), we see the build-up and decay of each oxidation state at positions in the titration, supporting the presence of some modest global aromatic and antiaromatic ring currents in this system.

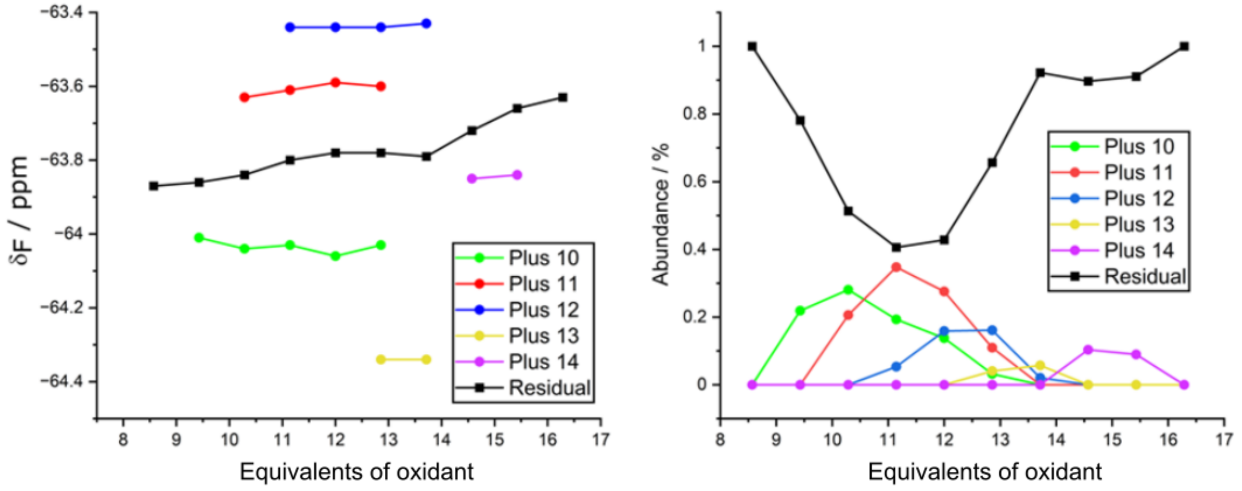

Figure S45. Chemical shift (δ_F, ppm) values of the oxidation states present at each point in the oxidative titration of ***c*-P18**$_{THS}$**·T18**$_{A}$ at 233 K (left) from deconvolution of the spectra (Figure S44). Speciation curve showing the abundance of each oxidation state at each point in the titration along with the abundance of the species contributing to the residual signal (neutral/distorted geometry/non-globally anti/aromatic nanorings) (right). (Data from spectra in Figure S42.)

*10.2. Oxidation of **c-P18**$_{THS}$**·T18**$_{B}$*

Oxidative titrations were performed and analyzed in the manner described in Section 10.1 on the ***c*-P18**$_{THS}$**·T18**$_{B}$ complex, to give the results shown in Figures S46–S53.

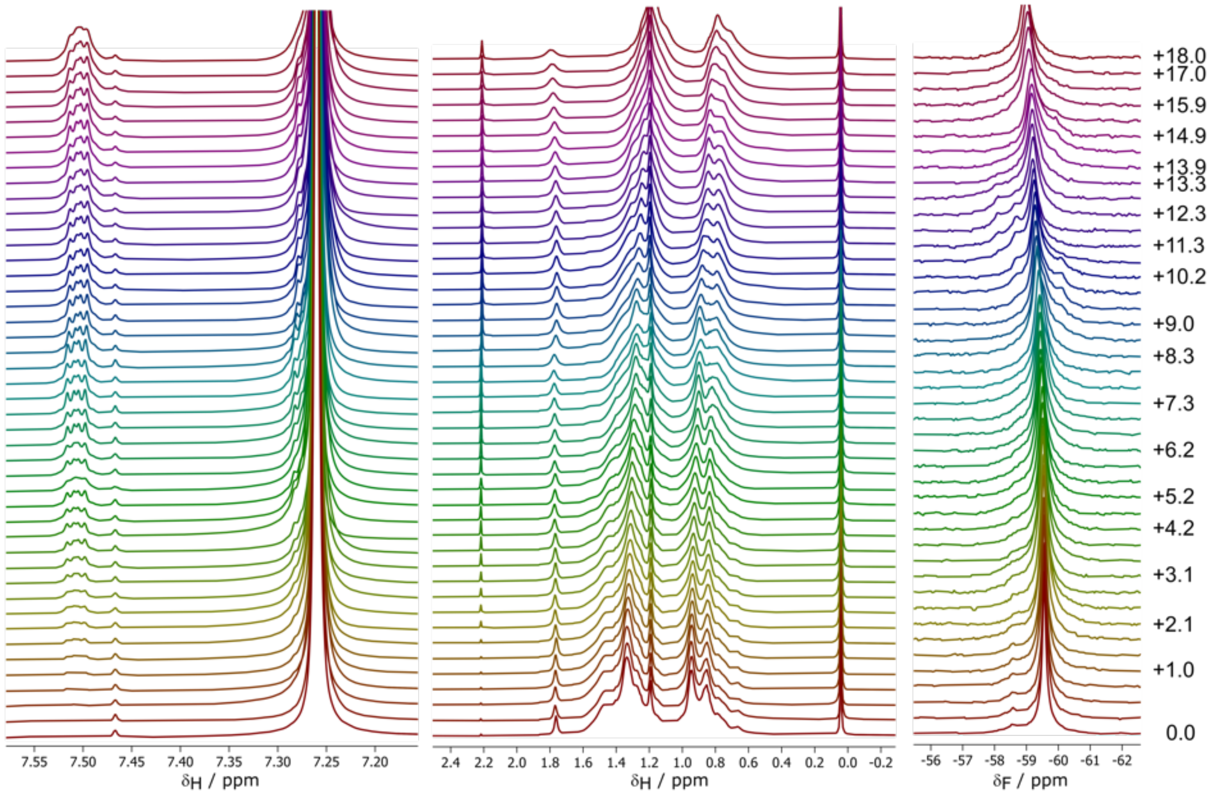

Figure S46. ^{1}H and ^{19}F NMR spectra from the 233 K oxidative titration of ***c*-P18**$_{THS}$**·T18**$_{B}$ (CDCl$_3$, ^{1}H 500MHz / ^{19}F 470 MHz).

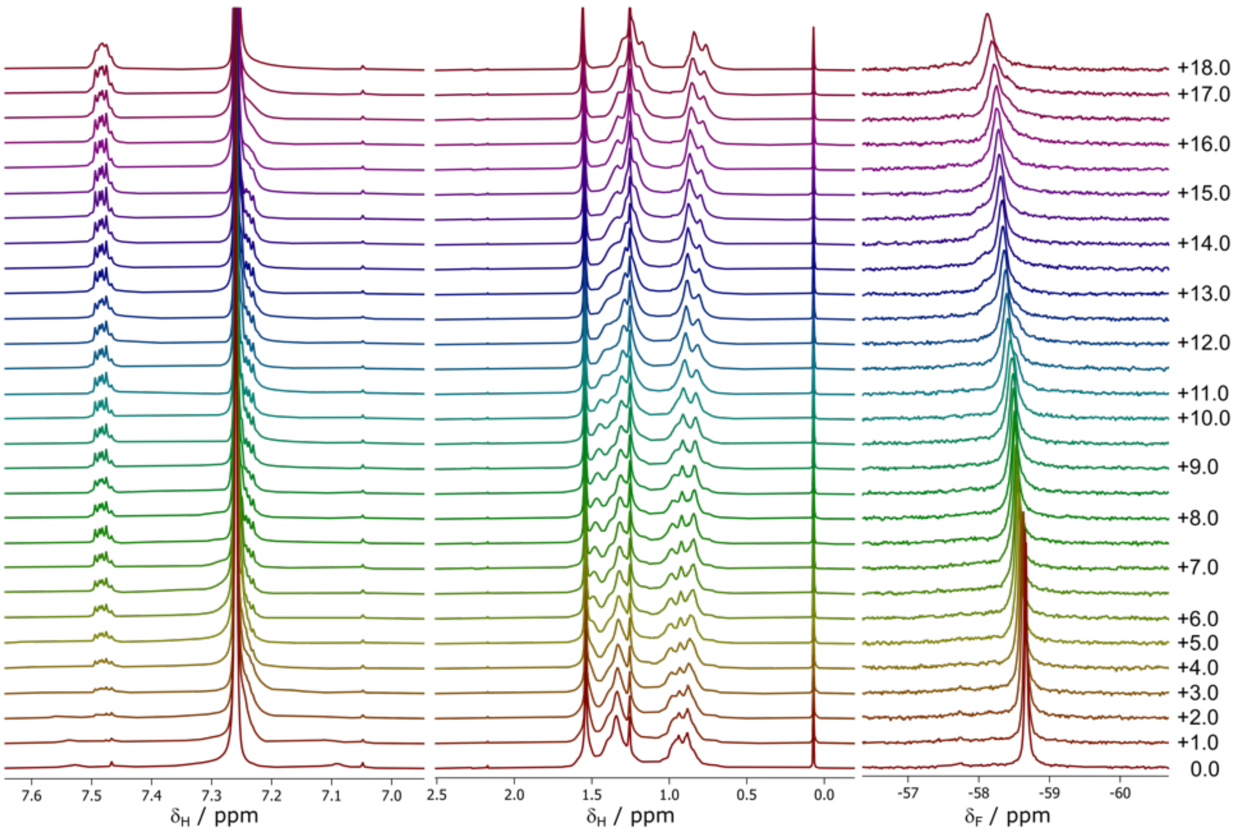

Figure S47. ^{1}H and ^{19}F NMR spectra from the 298 K oxidative titration of ***c*-P18$_{\mathbf{THS}}$·T18$_{\mathbf{B}}$** ($CDCl_3$, ^{1}H 500 MHz / ^{19}F 470 MHz).

In contrast to the ***c*-P18$_{\mathbf{THS}}$·T18$_{\mathbf{A}}$** complex, the spectra of ***c*-P18$_{\mathbf{THS}}$·T18$_{\mathbf{B}}$** show shoulder peaks with a discernable pattern at both 298 K and 233 K, reflecting the stronger π-conjugation in ***c*-P18$_{\mathbf{THS}}$·T18$_{\mathbf{B}}$**. The best data are still obtained at 233 K, and this data set is the focus of our analysis.

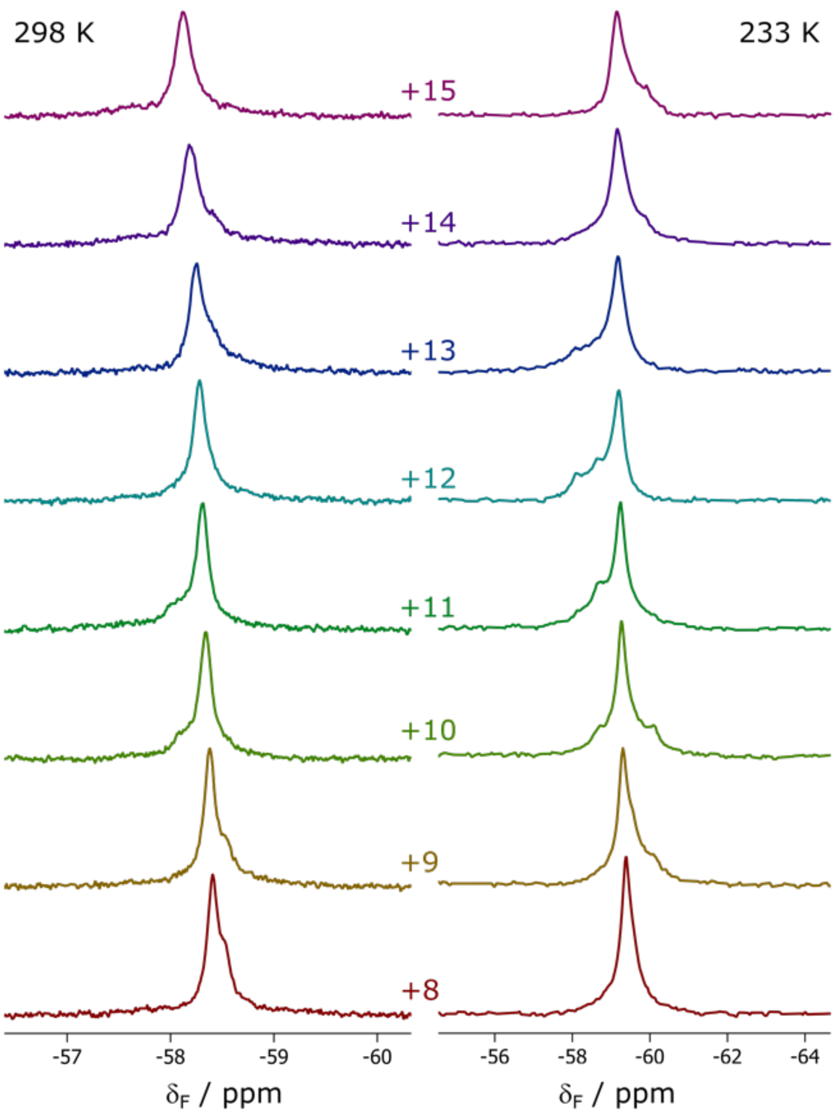

Figure S48. Comparison of the ^{19}F NMR spectra from the 233 K and 298 K oxidative titrations of ***c*-P18$_{THS}$·T18$_B$** in the +8 to +15 region ($CDCl_3$, ^{1}H 500 MHz/ ^{19}F 470 MHz). (Data from spectra in Figures S46 and S47.)

As with the ***c*-P18$_{THS}$·T18$_A$** 233 K titration, spectral deconvolution allowed us to identifying each oxidation state present in the titration of ***c*-P18$_{THS}$·T18$_B$** at 233 K (Figure S49 and S50). In contrast to the ***c*-P18$_{THS}$·T18$_A$** deconvolution, these data shows contributions from low oxidation states (+5 to +9).

The speciation curves from the ***c*-P18$_{THS}$·T18$_B$** titration (Figure S50) agree with the data from the ***c*-P18$_{THS}$·T18$_A$** complex (Figure S45). A comparison of the speciation of the two complexes from the +10 to +14 state is shown in Figure S51.

The trend in the ^{19}F NMR chemical shifts of the oxidation states observed in the ***c*-P18$_{THS}$·T18$_B$** titration (Figure S50) is also similar to that for ***c*-P18$_{THS}$·T18$_A$** (Figure S45). A comparison of the chemical shifts from the two complexes in the +10 to +14 state is shown in Figure S52. The values for each oxidation state were calculated as the average value of the state across all ^{19}F NMR spectra in which it was identified, (referenced to the ^{19}F NMR resonance of the neutral complex at –40 °C). For example, in Figure S49 the +10 state is observed in spectra 4, 5 and 6. The plotted value for ***c*-P18$_{THS}$·T18$_B^{+10}$** in Figure S52 is therefore the average value of the +10 state in spectra 4–6, after subtraction of δ_F for ***c*-P18$_{THS}$·T18$_B$**.

From the comparison of δ_F values in Figure S52, we can see that both complexes show the same trend across the range of oxidation states (negative shift in the +10 and +14 aromatic states, and a positive shift in the antiaromatic +12 state). Note that ***c*-P18**$_{\mathbf{THS}}$·**T18**$_{\mathbf{B}}$ has significantly larger shifts than ***c*-P18**$_{\mathbf{THS}}$·**T18**$_{\mathbf{A}}$.

Figure S53 highlights the trend of oscillating aromatic and antiaromatic states observed in the titration of ***c*-P18**$_{\mathbf{THS}}$·**T18**$_{\mathbf{B}}$.

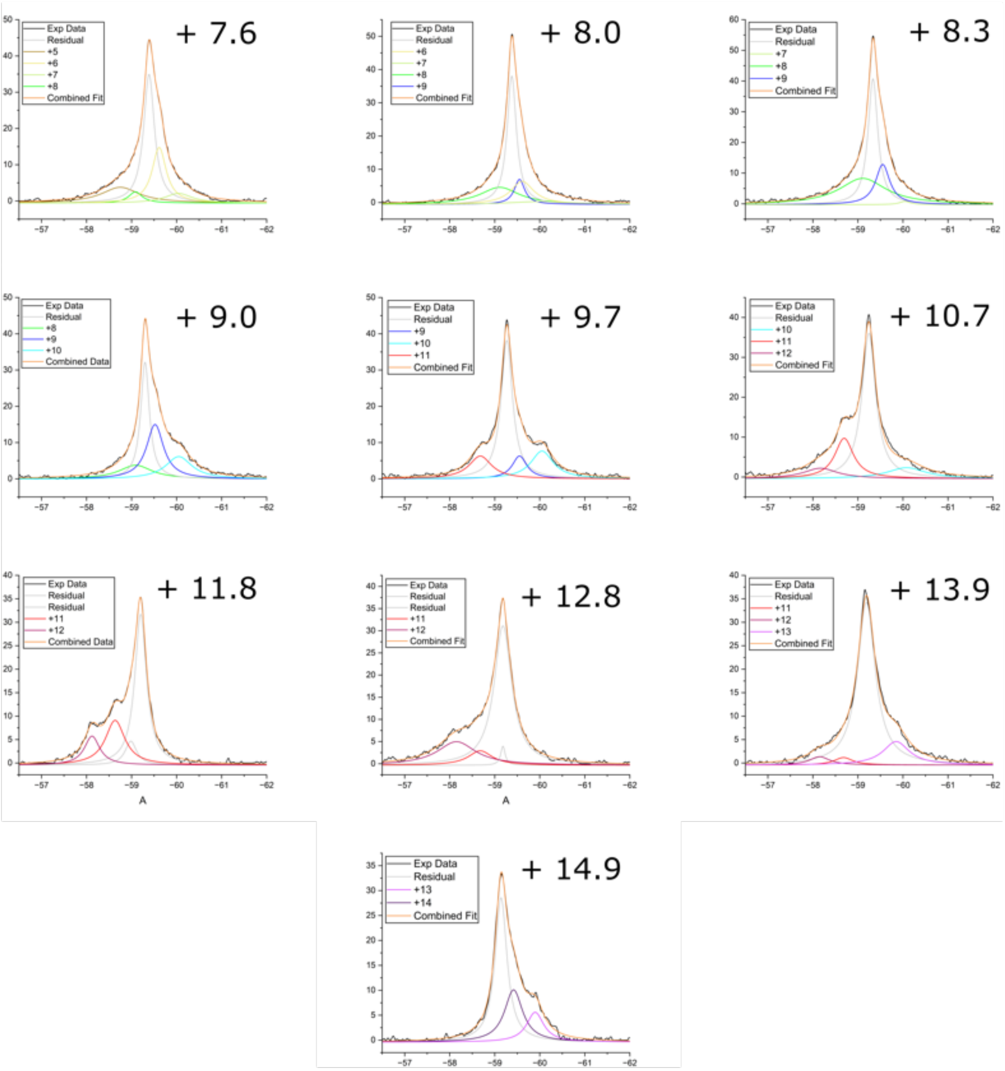

Figure S49. Manually deconvoluted ^{19}F NMR data from the 233 K oxidative titration of ***c*-P18**$_{\mathbf{THS}}$·**T18**$_{\mathbf{B}}$. (Data from spectra in Figure S46.) Numbers at the top right of each panel are the equivalents of ThnBArF.

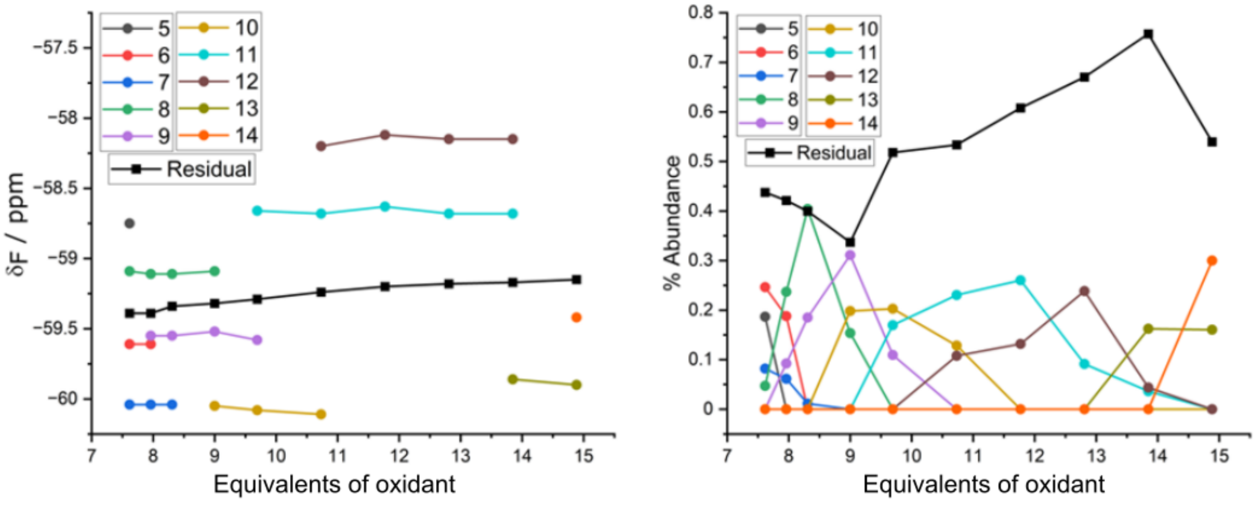

Figure S50. Chemical shift (δ_F, ppm) values of the oxidation states present at each point in the titration (left). Speciation curve showing the abundance of each oxidation state at each point in the titration along with the abundance of the species (neutral/distorted geometry/non-globally anti/aromatic nanorings) contributing to the residual signal (right). Oxidative titrations of ***c*-P18**$_{\mathbf{THS}}$·**T18**$_{\mathbf{B}}$ at 233 K. (Data from spectra in Figure S46.)

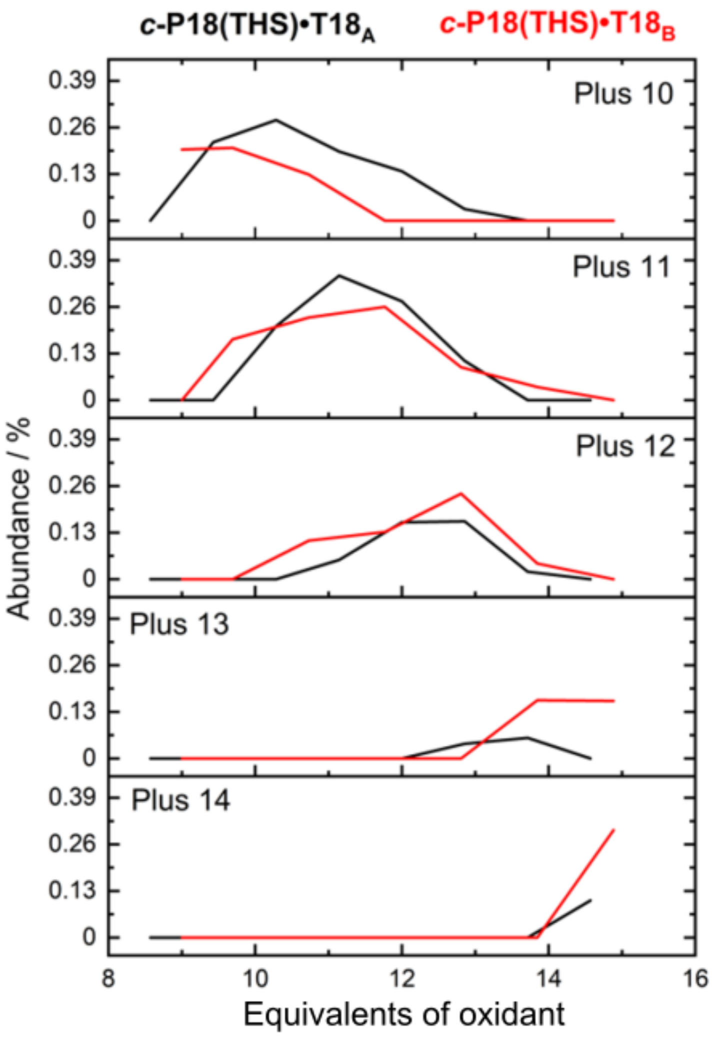

Figure S51. Comparison of the speciation curves for the +10 to +14 oxidation states in the deconvoluted spectra of ***c*-P18**$_{\mathbf{THS}}$·**T18**$_{\mathbf{A}}$ (black) and ***c*-P18**$_{\mathbf{THS}}$·**T18**$_{\mathbf{B}}$ (red) at 233 K. (Data from spectra in Figures S42 and S46.)

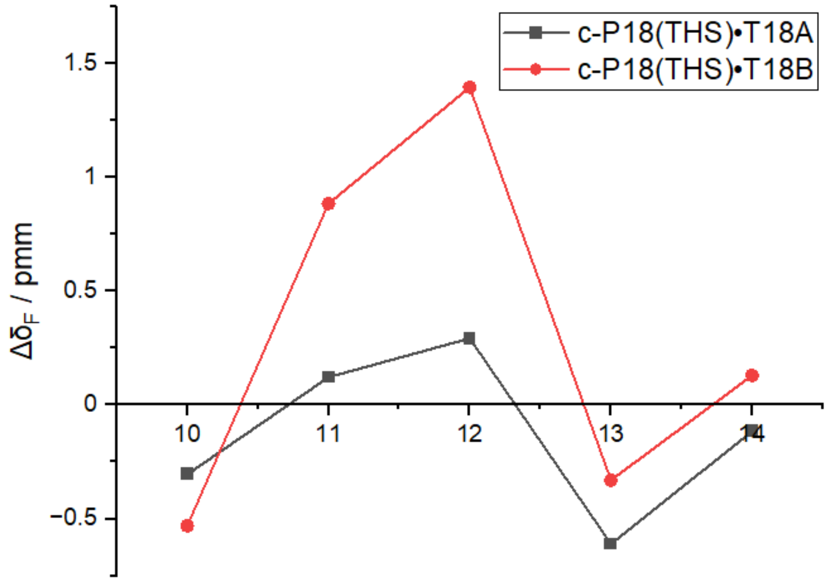

Figure S52. Difference in chemical shift (δ_F, ppm) values between the observed oxidation state and ^{19}F NMR resonance of each neutral complex (at –40 °C) for ***c*-P18**$_{\mathbf{THS}}$**·T18**$_{\mathbf{A}}$ (black) and ***c*-P18**$_{\mathbf{THS}}$**·T18**$_{\mathbf{B}}$ (red)

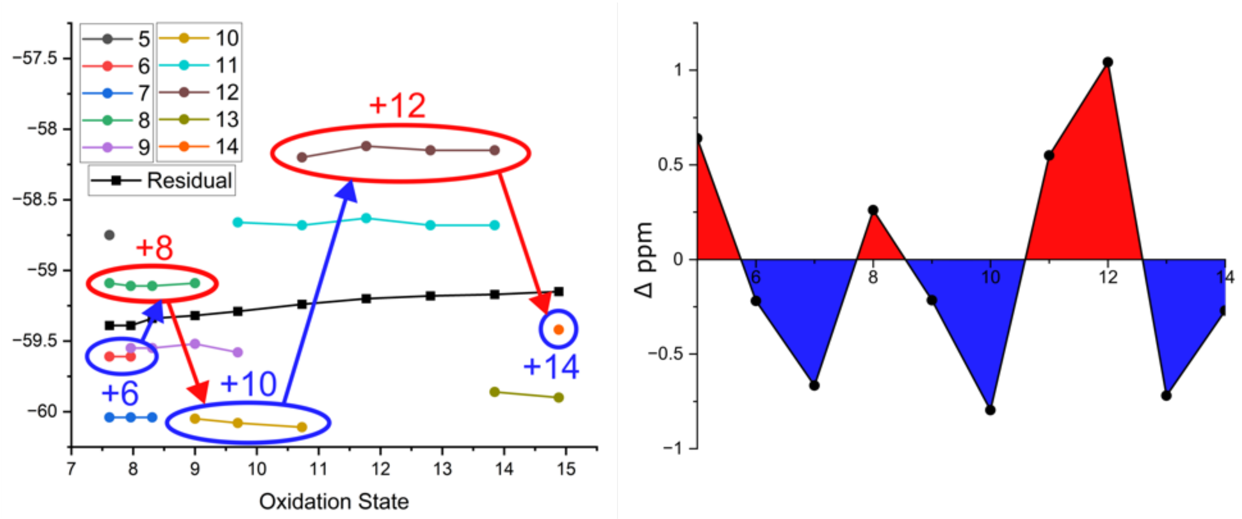

Figure S53. Chemical shift (δ_F, ppm) values of the oxidation states present at each point in the titration with highlighted aromatic states (blue) and antiaromatic states (red) (left). Difference in PPM value between the observed oxidation state and the residual value in the deconvoluted spectra of ***c*-P18**$_{\mathbf{THS}}$**·T18**$_{\mathbf{B}}$ with highlighted aromatic states (blue) and antiaromatic states (red) (right).

10.3. Oxidation of the split-ring complex ***P18***$_{\mathbf{THS}}$***(C***$_{\mathbf{2}}$***CPDIPS)***$_{\mathbf{2}}$***·T18***$_{\mathbf{B}}$

As a control experiment, to test our interpretation of the low-temperature oxidative NMR titrations for ***c*-P18**$_{\mathbf{THS}}$**·T18**$_{\mathbf{A/B}}$, we performed a titration on a 1:1 complex of the linear porphyrin 18-mer bound to the **T18**$_{\mathbf{B}}$ template **P18**$_{\mathbf{THS}}$**(C**$_{\mathbf{2}}$**CPDIPS)**$_{\mathbf{2}}$**·T18**$_{\mathbf{B}}$ at –40 °C. The structure of this complex is shown in Figure S54. We anticipated that this split-ring complex would show no shifted ^{19}F NMR signals for different oxidation states, as it is not able to sustain global ring currents due to the break in the cyclic π-system.

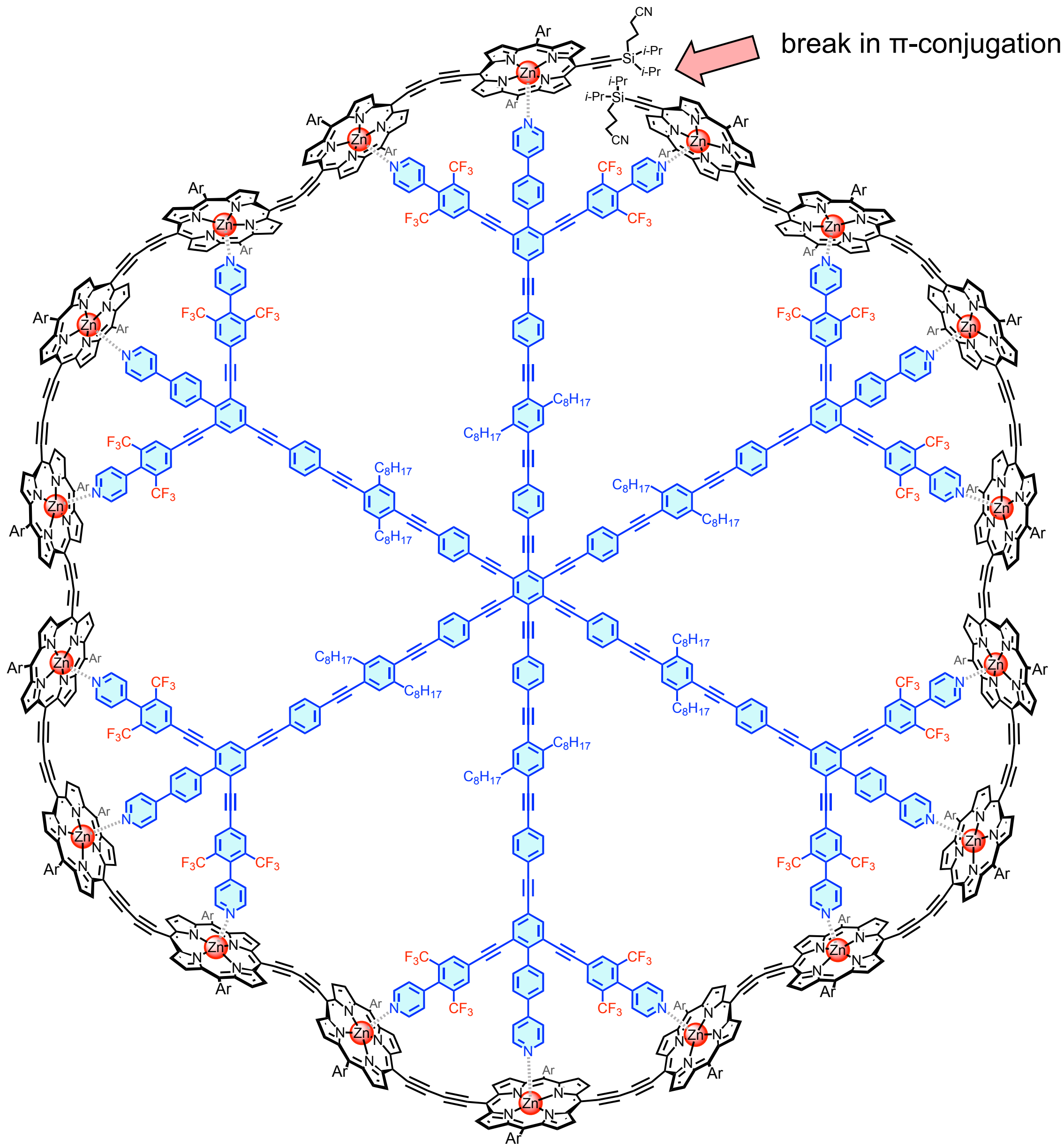

Figure S54. Chemical structure of the split-ring complex **P18$_{THS}$(C$_2$CPDIPS)$_2$·T18$_B$**.

A UV-vis-NIR titration of **P18$_{THS}$(C$_2$CPDIPS)$_2$** with **T18$_B$** (in $CDCl_3$ at 298 K) showed formation of a strong 1:1 complex, **P18$_{THS}$(C$_2$CPDIPS)$_2$·T18$_B$**, and this was confirmed by ^{19}F NMR spectroscopy. The 1:1 complex, **P18$_{THS}$(C$_2$CPDIPS)$_2$·T18$_B$**, is in slow exchange with excess **T18$_B$** template (Figure S55).

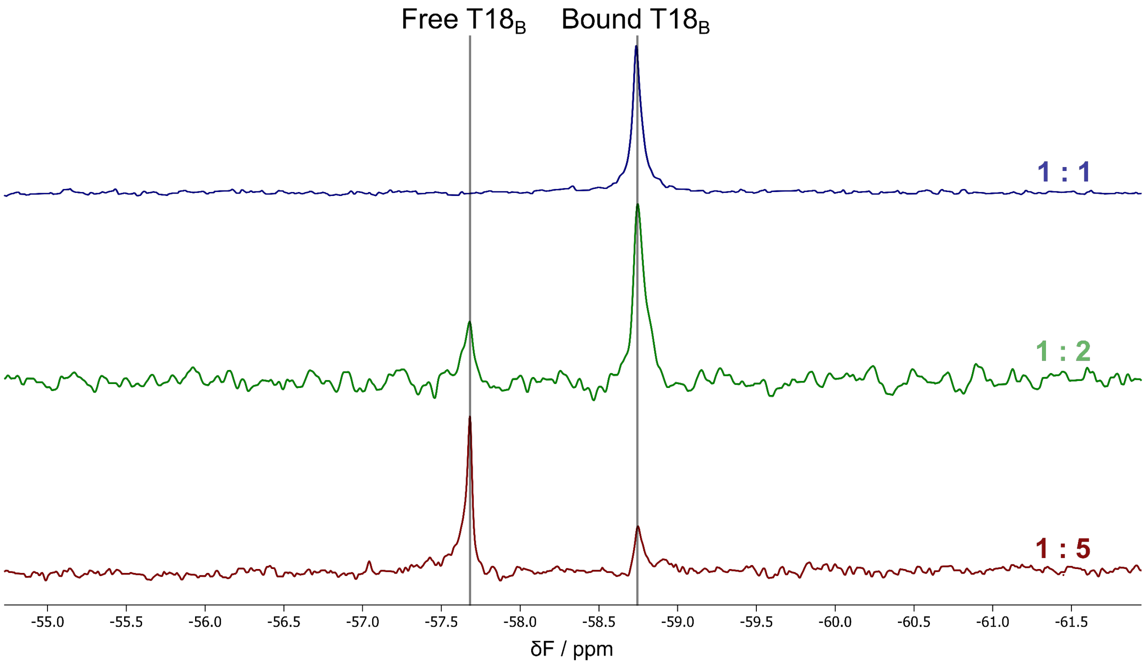

Figure S55. ^{19}F NMR of 1:1, 1:2 and 1:5 stoichiometries of **P18$_{THS}$(C$_2$CPDIPS)$_2$: T18$_B$** in $CDCl_3$ at 298 K (reference: C_6F_6 $\delta_F = -161.64$).

The oxidative NMR experiment was performed on the 1:1 complex, **P18$_{THS}$(C$_2$CPDIPS)$_2$·T18$_B$**, using the experimental set up described above (Sections 10.1 and 10.2). The raw ^{1}H and ^{19}F NMR spectra from this titration are shown in Figure S56, with the average oxidation state of **P18$_{THS}$(C$_2$CPDIPS)$_2$** stated alongside each spectrum, as calculated from the point at which the thianthrene signal first broadens in comparison to the amount of oxidant added. As expected, we observe no additional peaks growing in or decaying. Instead, only a broadening of the ^{19}F signal is observed along with a gradual shift to more positive ppm values over the course of the titration. Due to the featureless spectrum at all points in the titration, each ^{19}F signal can be well described by a single gaussian peak. This control experiment supports the conclusion that the additional peaks that grow in and decay in the titrations of ***c*-P18$_{THS}$·T18$_{A/B}$** are associated with globally aromatic ring currents.

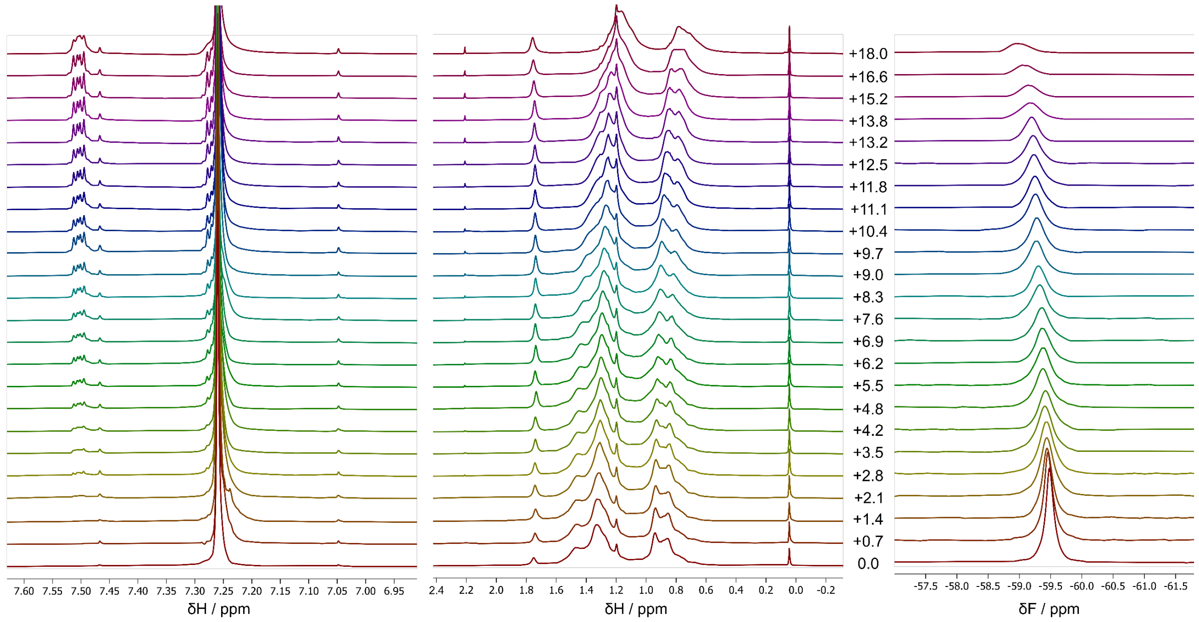

Figure S56. ^{1}H and ^{19}F NMR spectra from the oxidative titration of **P18$_{THS}$(C$_2$CPDIPS)$_2$·T18$_B$** at 233 K in $CDCl_3$. ^{19}F NMR spectra referenced against C_6F_6 $\delta_F = -161.64$.

Section 11. Quantification of Ring Currents from Experimental NMR Shifts

In order to quantify and compare the magnitude of the ring currents in the different oxidation states of ***c*-P18$_{THS}$·T18$_{A/B}$**, we employ the ring current loop model developed by Jirasek et al. (ref. 31; the model and its implementation are described, together with the corresponding MATLAB scripts in the Supplementary Information to this article). In brief, the Biot-Savart law enables one to calculate the magnetic field at any point in space arising from a current flowing between two points in space. Based on the PM7-optimized geometries of the neutral ***c*-P18$_{THS}$·T18$_{A}$** and ***c*-P18$_{THS}$·T18$_{B}$** complexes, we construct a current loop model consisting of linear segments of infinitely thin wire following the conjugated path of the nanoring. We then calculate the ring current geometric factor (RCGF) for the CF_3-probes on the template assuming a fixed ring current of 1 nA, averaged over all the fluorene environments. This gave RCGF = 0.12558 µT/nA for ***c*-P18$_{THS}$·T18$_{A}$** and 0.12735 µT/nA ***c*-P18$_{THS}$·T18$_{B}$**. Finally, the experimentally observed chemical shift difference between the CF_3 -signal for the respective oxidation state and the CF_3 -signal for the neutral complex (Δδ) is divided by the RCGF to give the ring current susceptibility *I*/*B*. For ***c*-P18$_{THS}$·T18$_{A/B}$** the 10+ and the 12+ states are resolved and the calculated ring current susceptibilities are shown in Table S1.

	***c*-P18$_{THS}$·T18$_{A}$**		***c*-P18$_{THS}$·T18$_{B}$**	
Oxidation State	Δδ (ppm)	*I*/*B*	Δδ (ppm)	*I*/*B*
10+	–0.3	–2.4	–0.53	–4.2
12+	0.29	2.3	1.40	10.9

Table S1. Experimentally observed changes in ^{19}F NMR peak position from neutral state in the 10+ and 12+ states of ***c*-P18$_{THS}$·T18$_{A/B}$** and the corresponding ring current susceptibility calculated using the Biot-Savart law and a ring current loop model.

Section 12. NMR and mass spectra of new compounds

Spectra of template intermediates

Compound 2

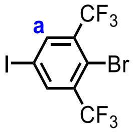

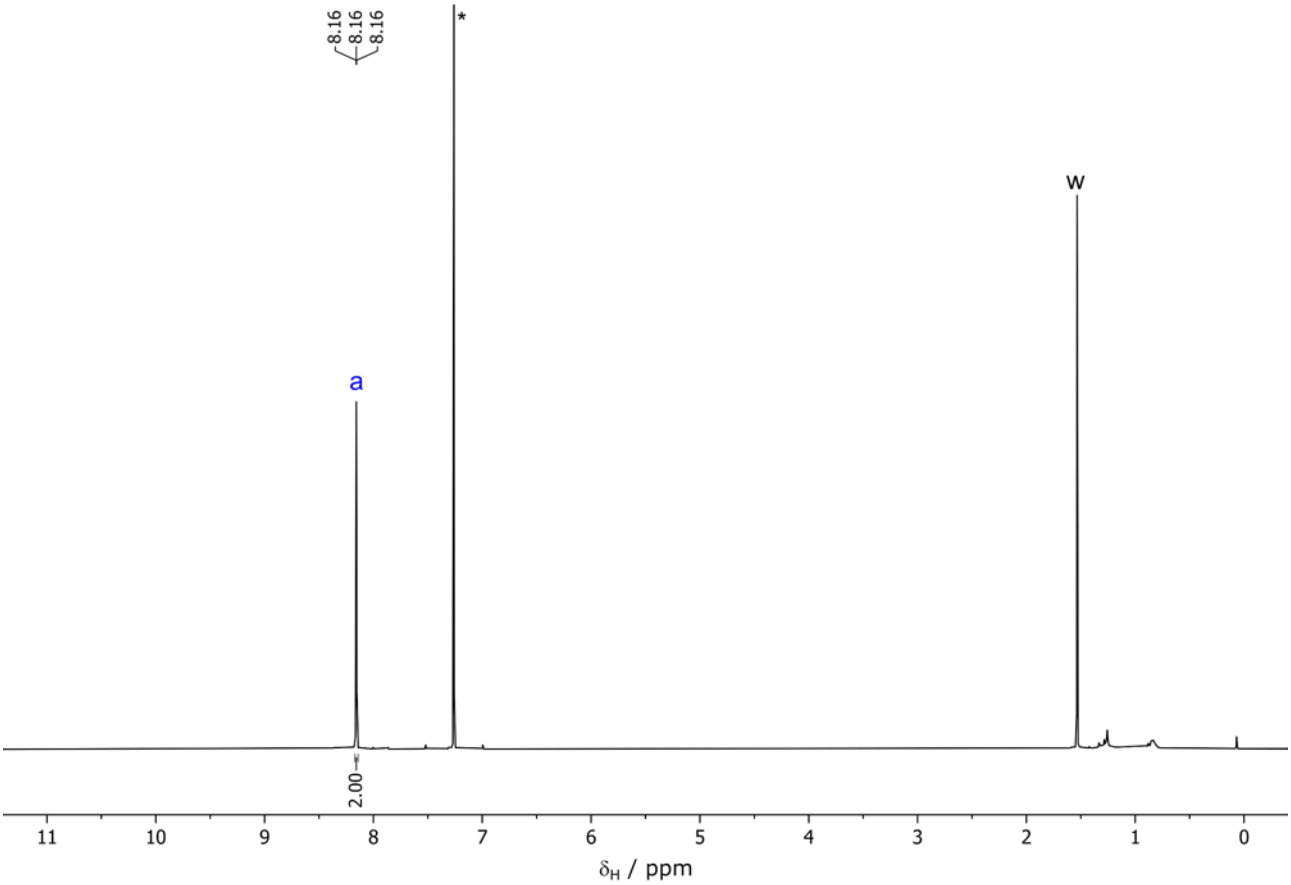

Figure S57. ^{1}H NMR spectrum of **2** (400 MHz, $CDCl_3$, 298 K). * = $CHCl_3$; w = H_2O.

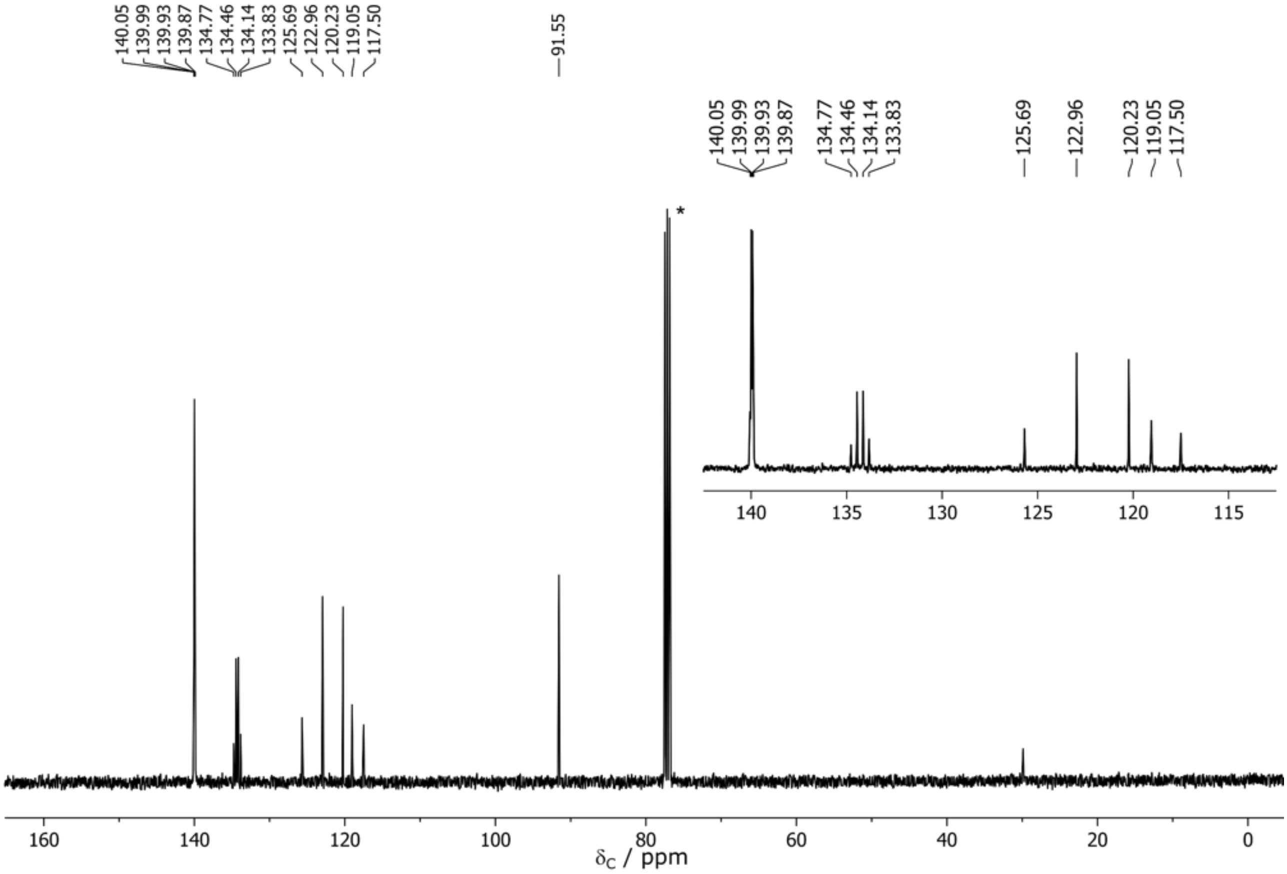

Figure S58. ^{13}C NMR spectrum of **2** (101 MHz, $CDCl_3$, 298 K). * = $CDCl_3$.

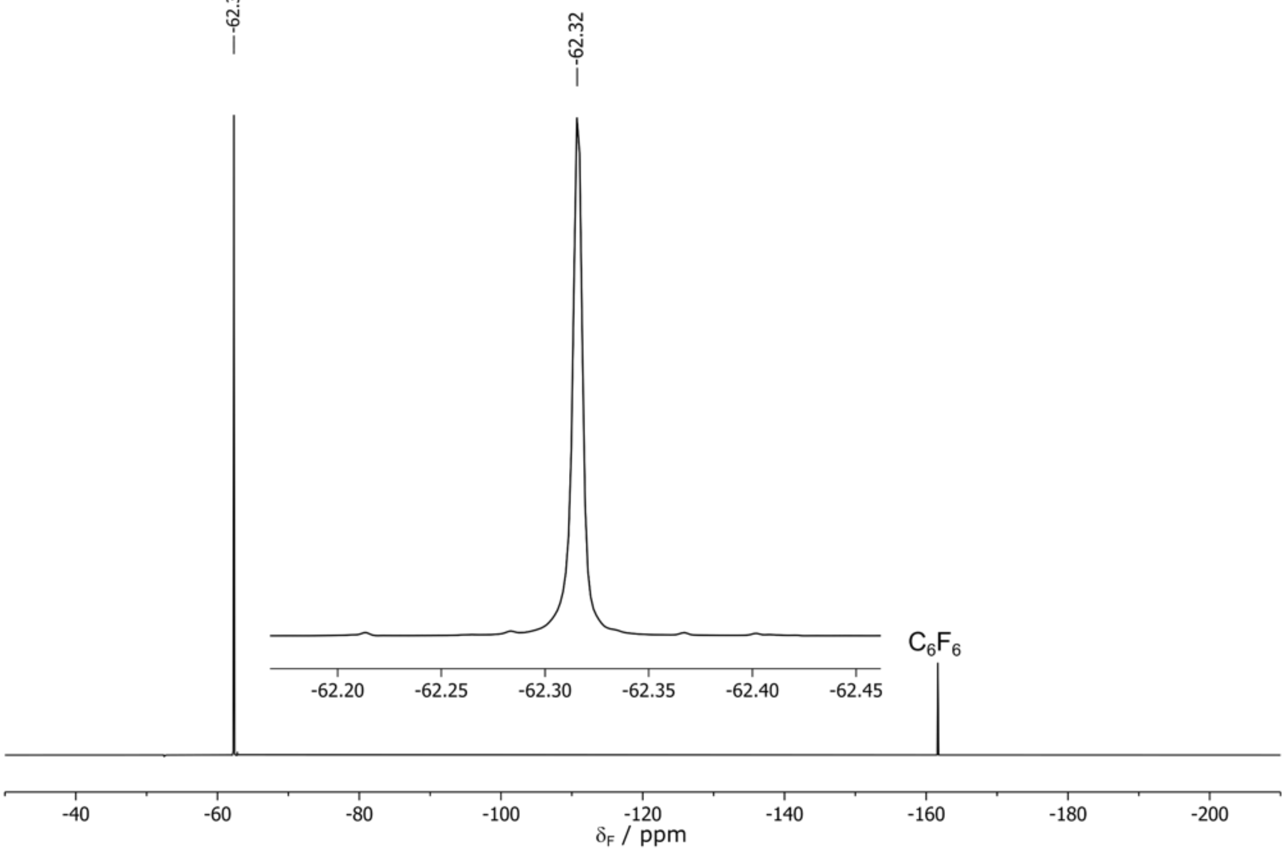

Figure S59. ^{19}F NMR spectrum of **2** (377 MHz, $CDCl_3$, 298 K). Referenced to C_6F_6 (δ_F = −161.64 ppm).

Compound 3

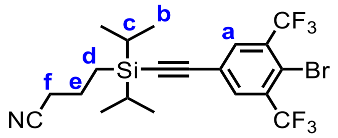

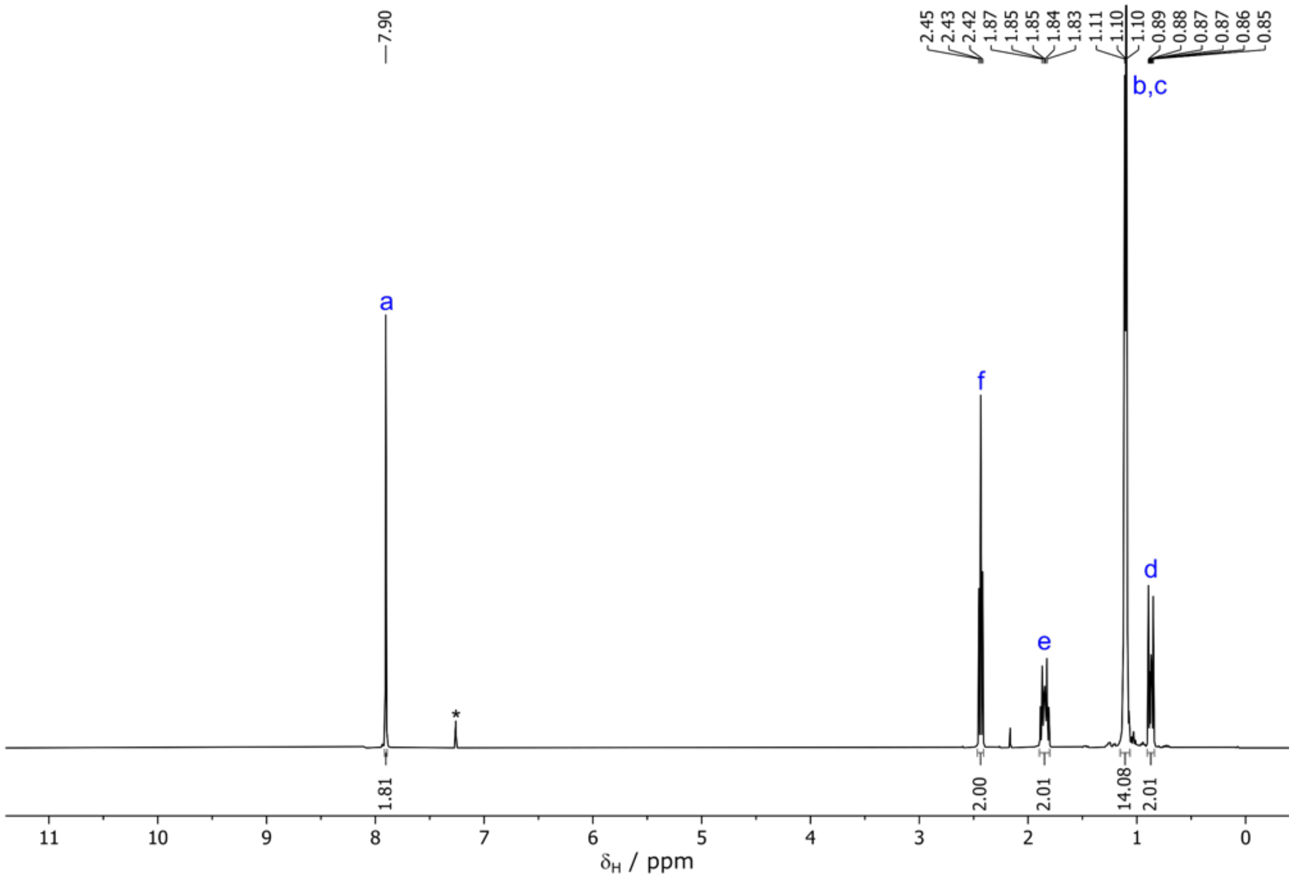

Figure S60. ^{1}H NMR spectrum of **3** (400 MHz, $CDCl_3$, 298 K). * = $CHCl_3$.

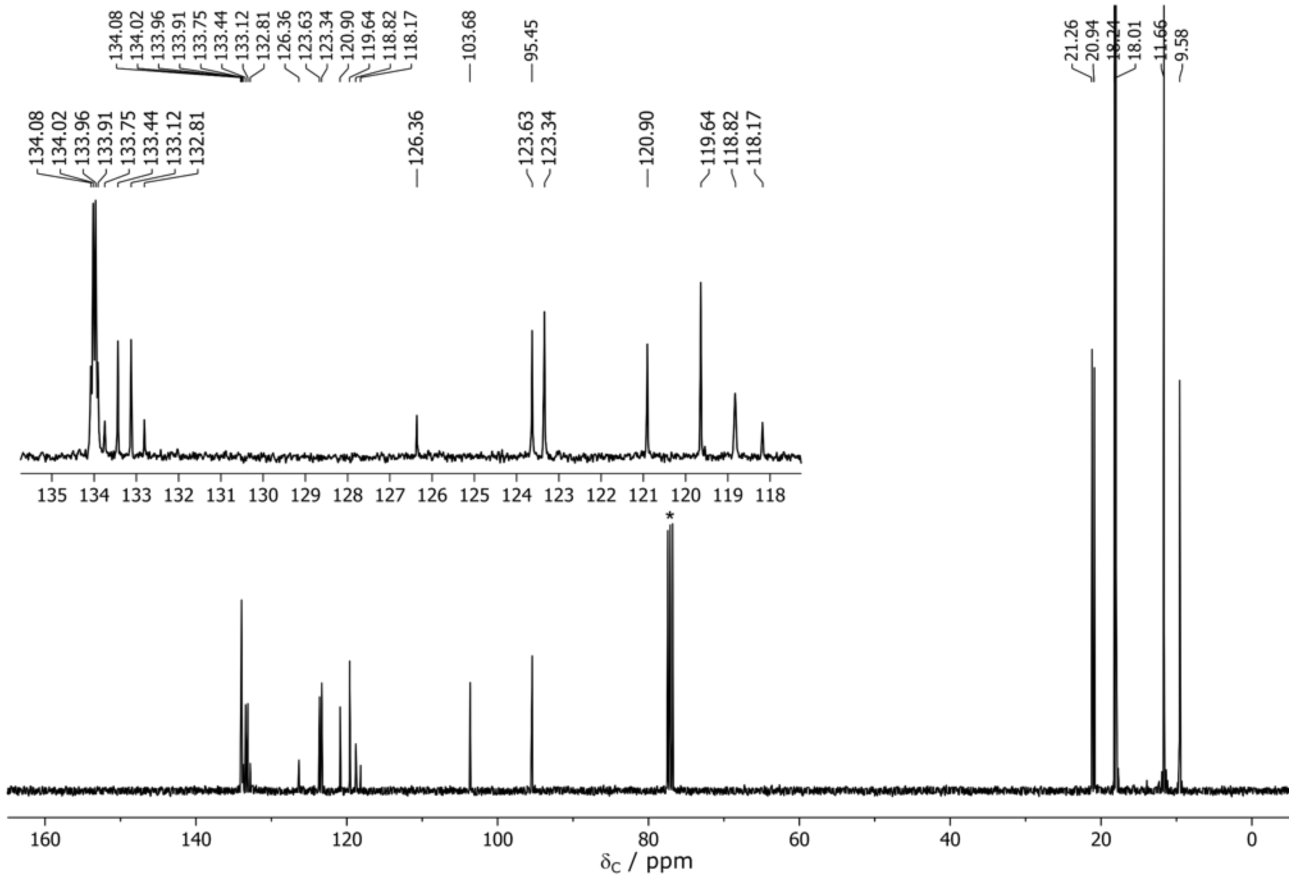

Figure S61. ^{13}C NMR spectrum of **3** (101 MHz, $CDCl_3$, 298 K). * = $CDCl_3$.

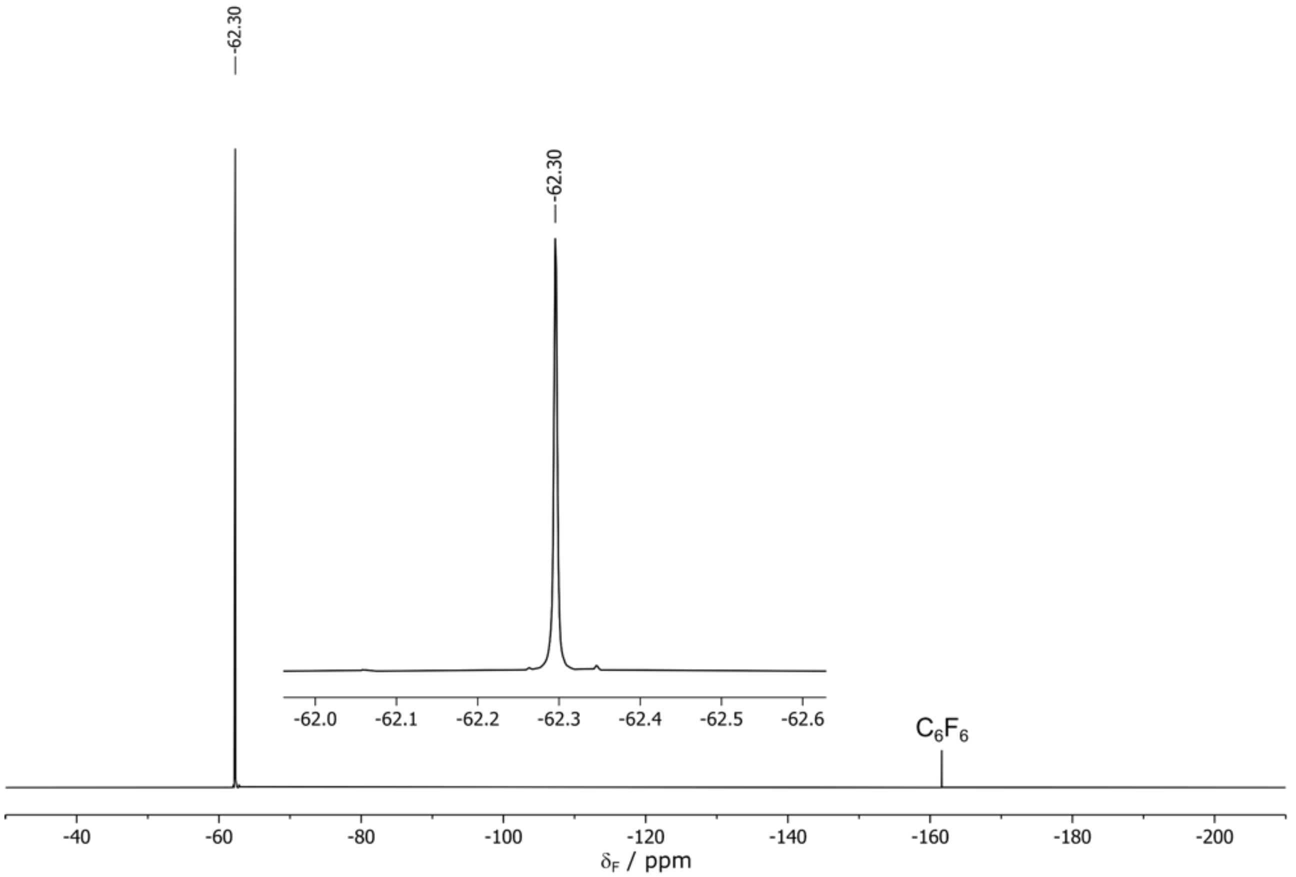

Figure S62. ^{19}F NMR spectrum of **3** (377 MHz, $CDCl_3$, 298 K). Referenced to C_6F_6 ($\delta_F = -161.64$).

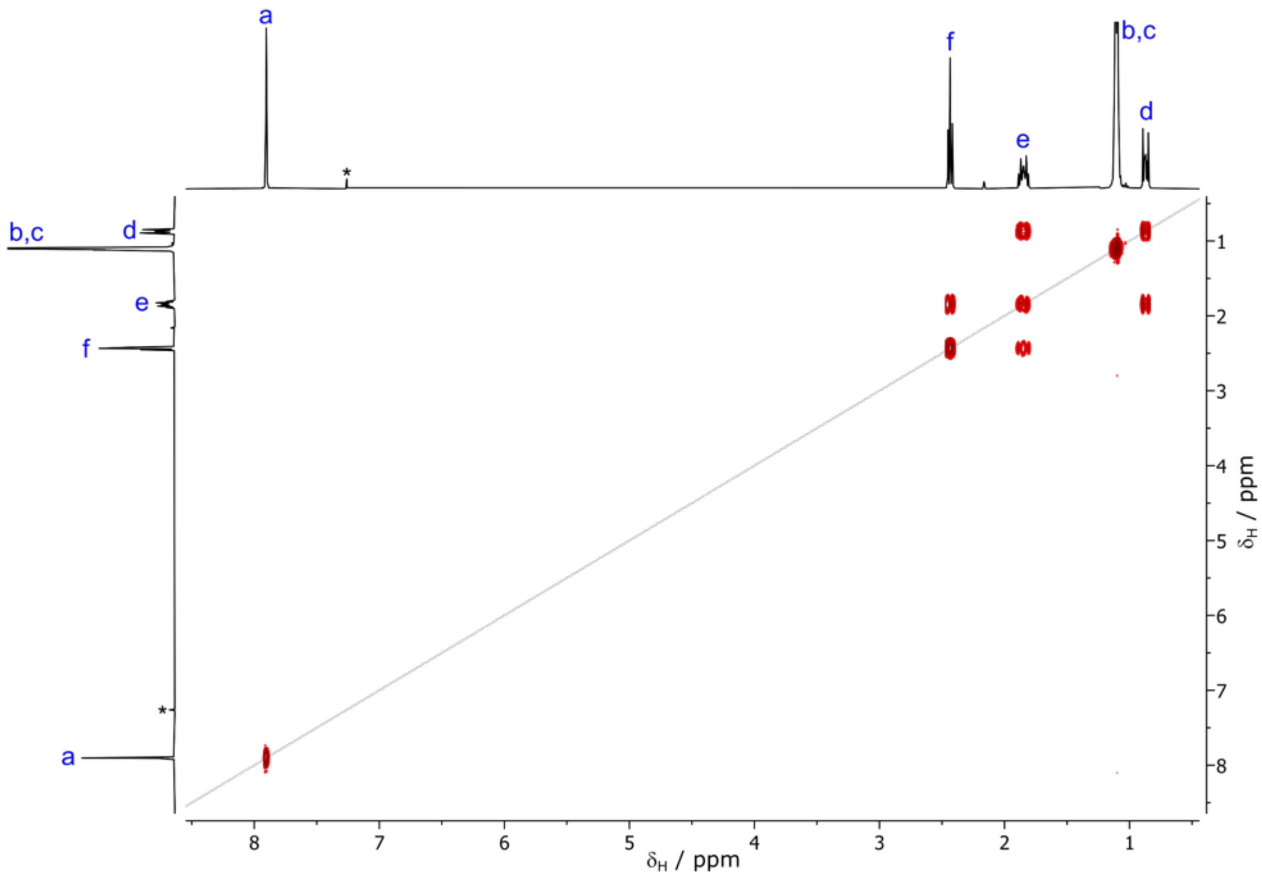

Figure S63. ^{1}H-^{1}H COSY spectrum of **3** (400 MHz, $CDCl_3$, 298 K). * = $CHCl_3$.

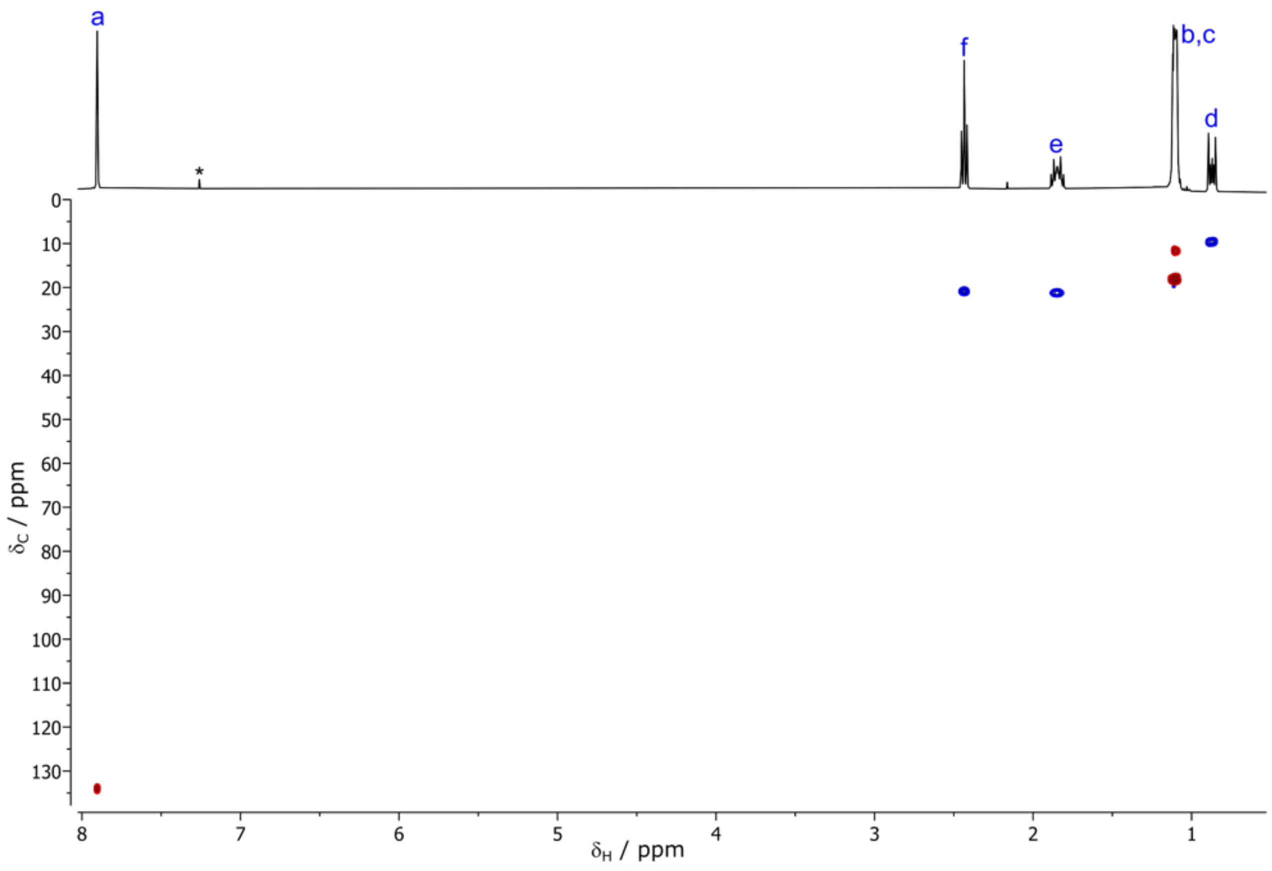

Figure S64. ^{1}H-^{13}C HSQC spectrum of **3** (400 MHz, $CDCl_3$, 298 K). * = $CHCl_3$.

Compound 4

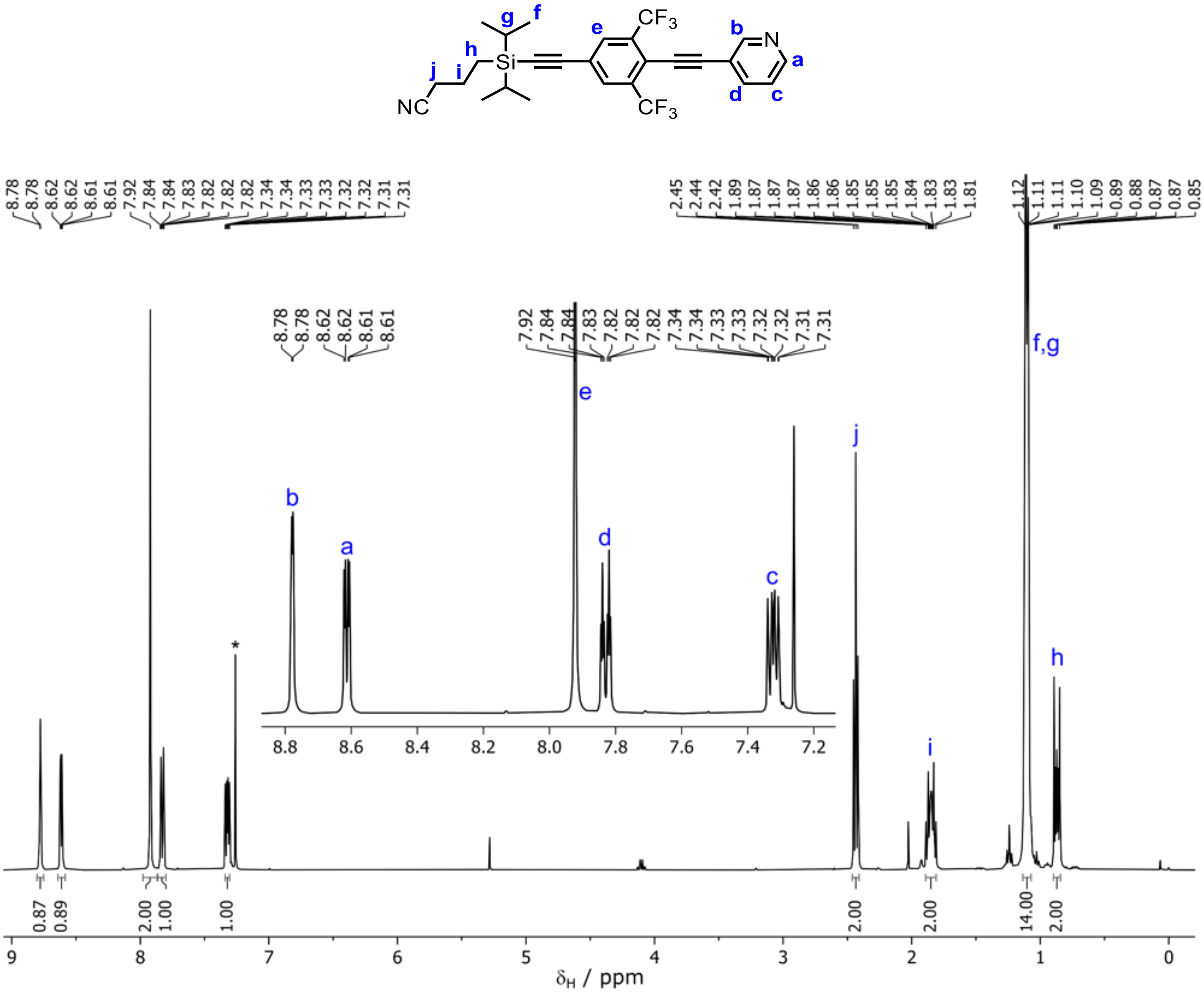

Figure S65. ^{1}H NMR spectrum of **4** (400 MHz, $CDCl_3$, 298 K). * = $CHCl_3$.

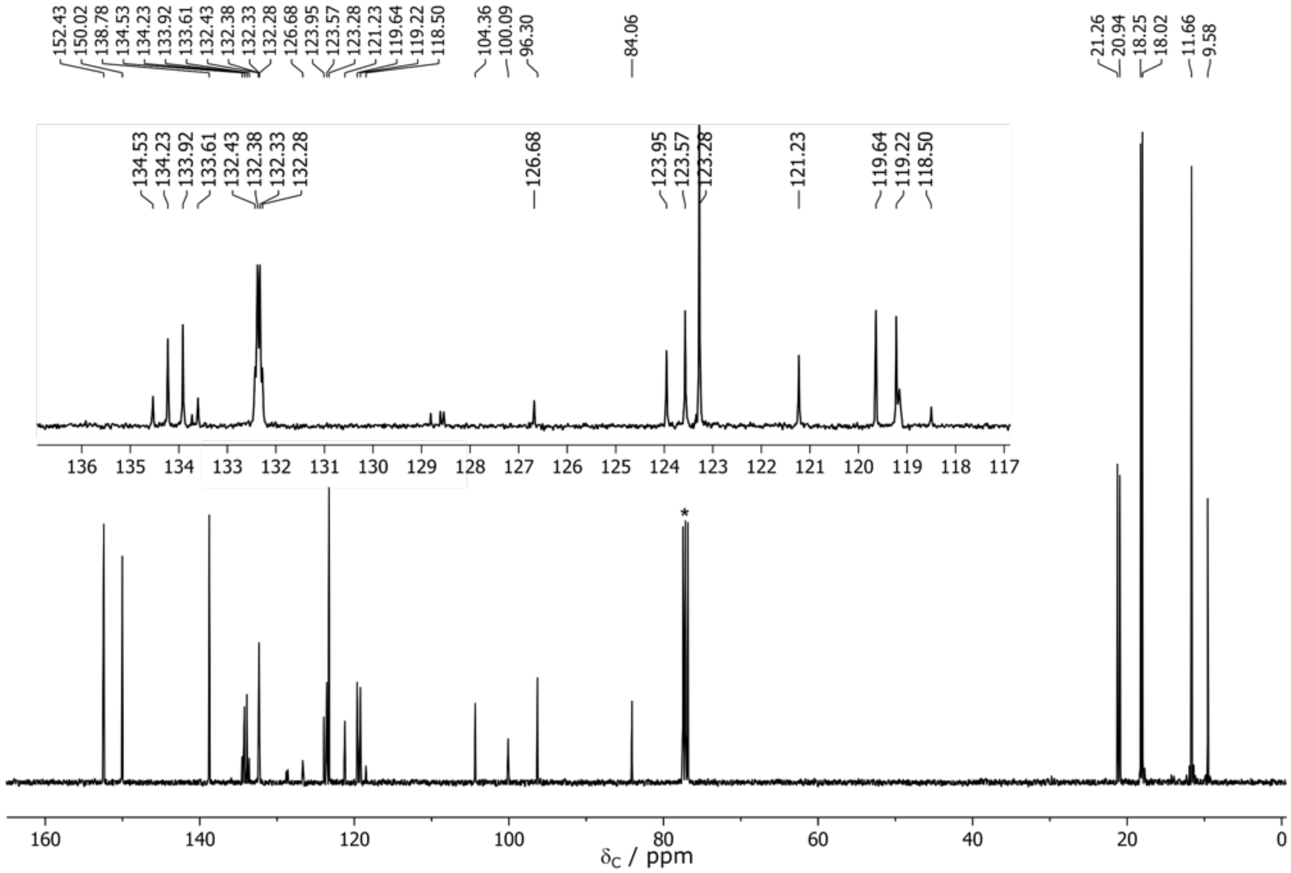

Figure S66. ^{13}C NMR spectrum of **4** (101 MHz, $CDCl_3$, 298 K). * = $CDCl_3$.

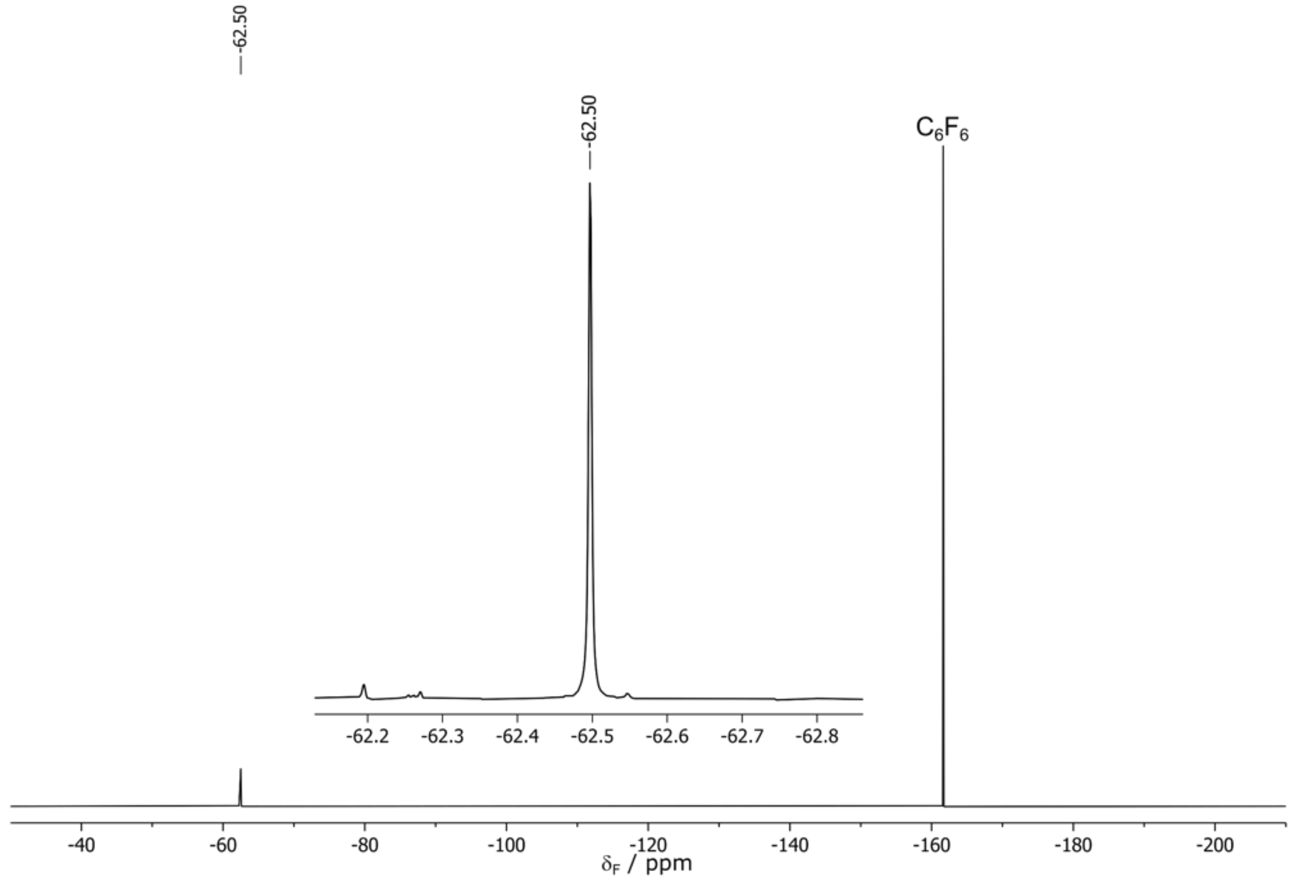

Figure S67. ^{19}F NMR spectrum of **4** (377 MHz, $CDCl_3$, 298 K). Referenced to C_6F_6 (δ_F = −161.64 ppm).

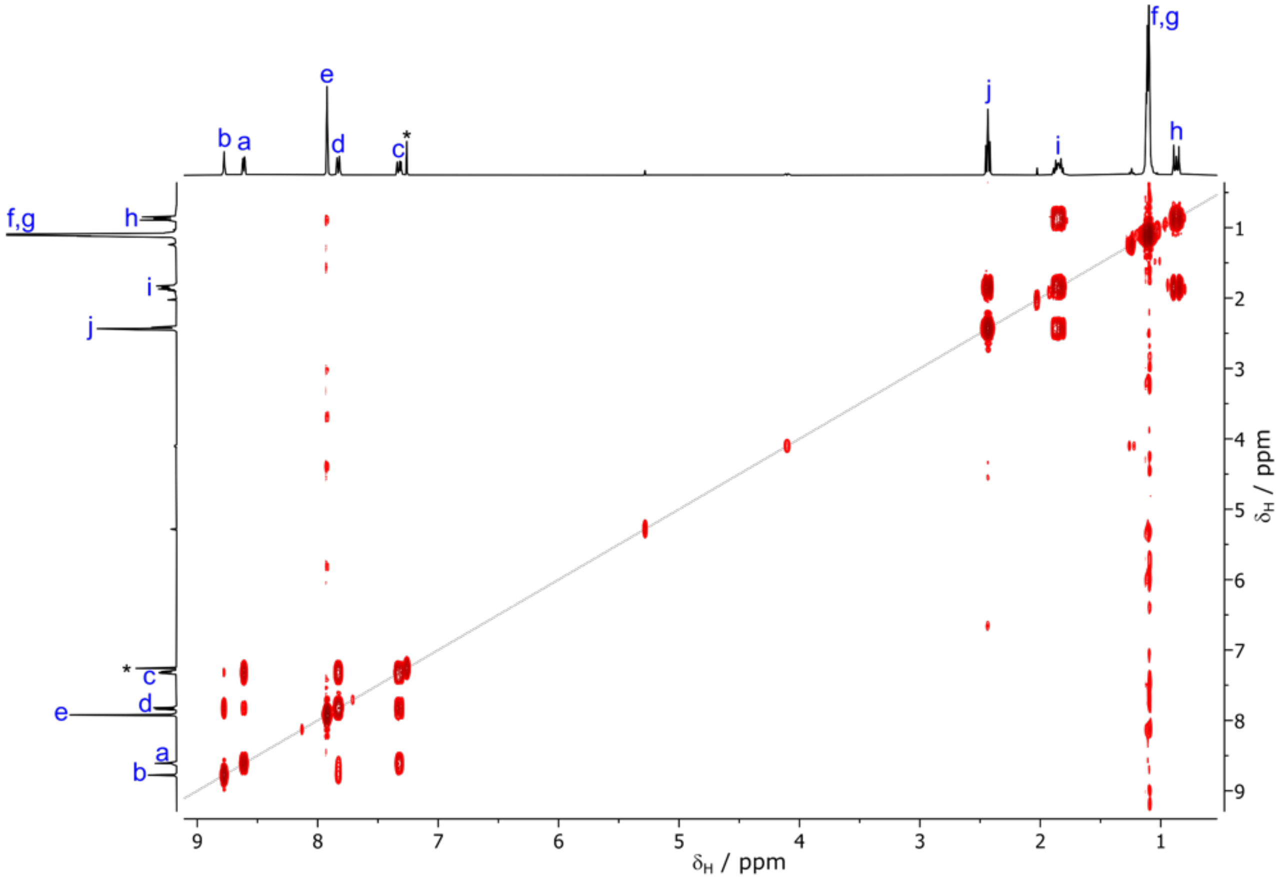

Figure S68. ^{1}H-^{1}H COSY spectrum of **4** (400 MHz, $CDCl_3$, 298 K). * = $CHCl_3$.

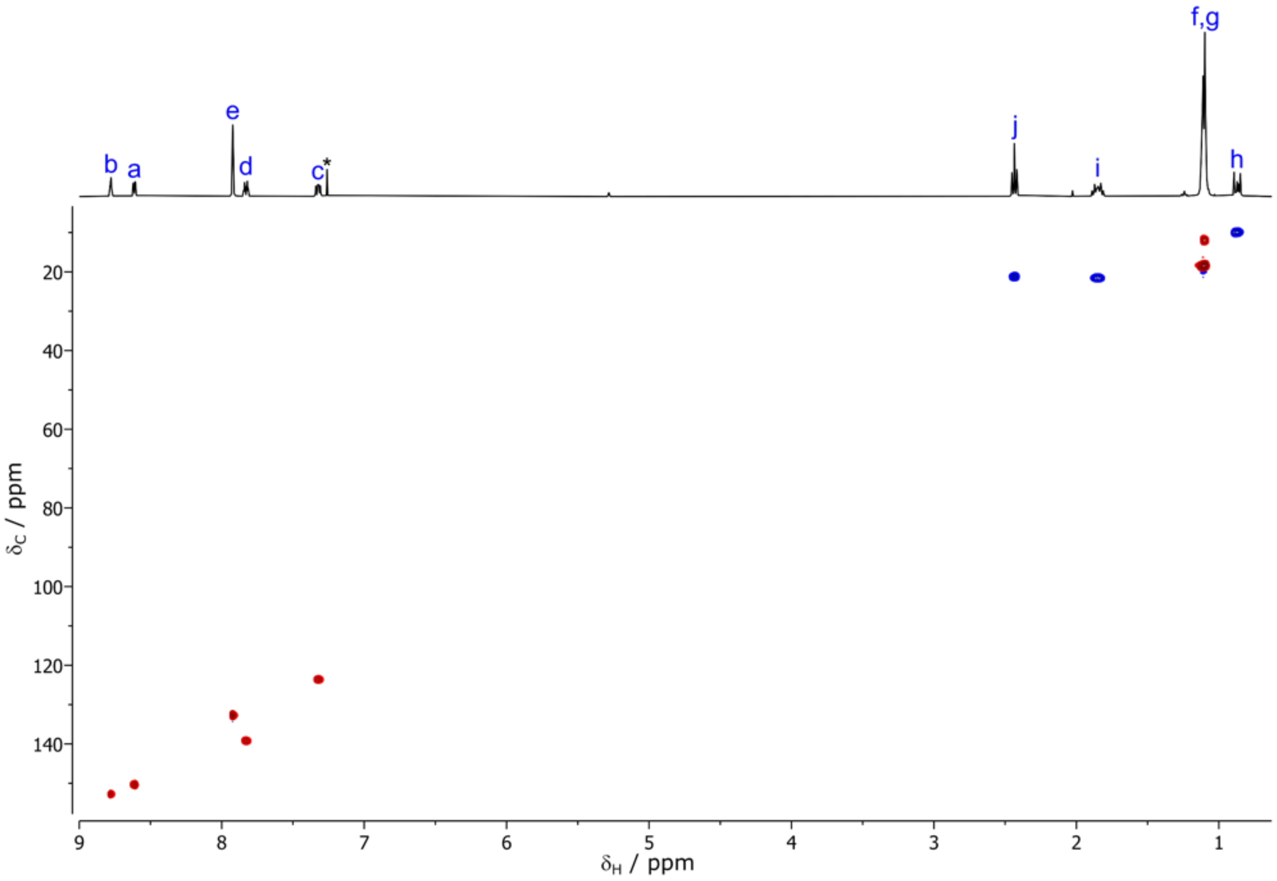

Figure S69. ^{1}H-^{13}C HSQC spectrum of **4** (400 MHz, $CDCl_3$, 298 K). * = $CHCl_3$.

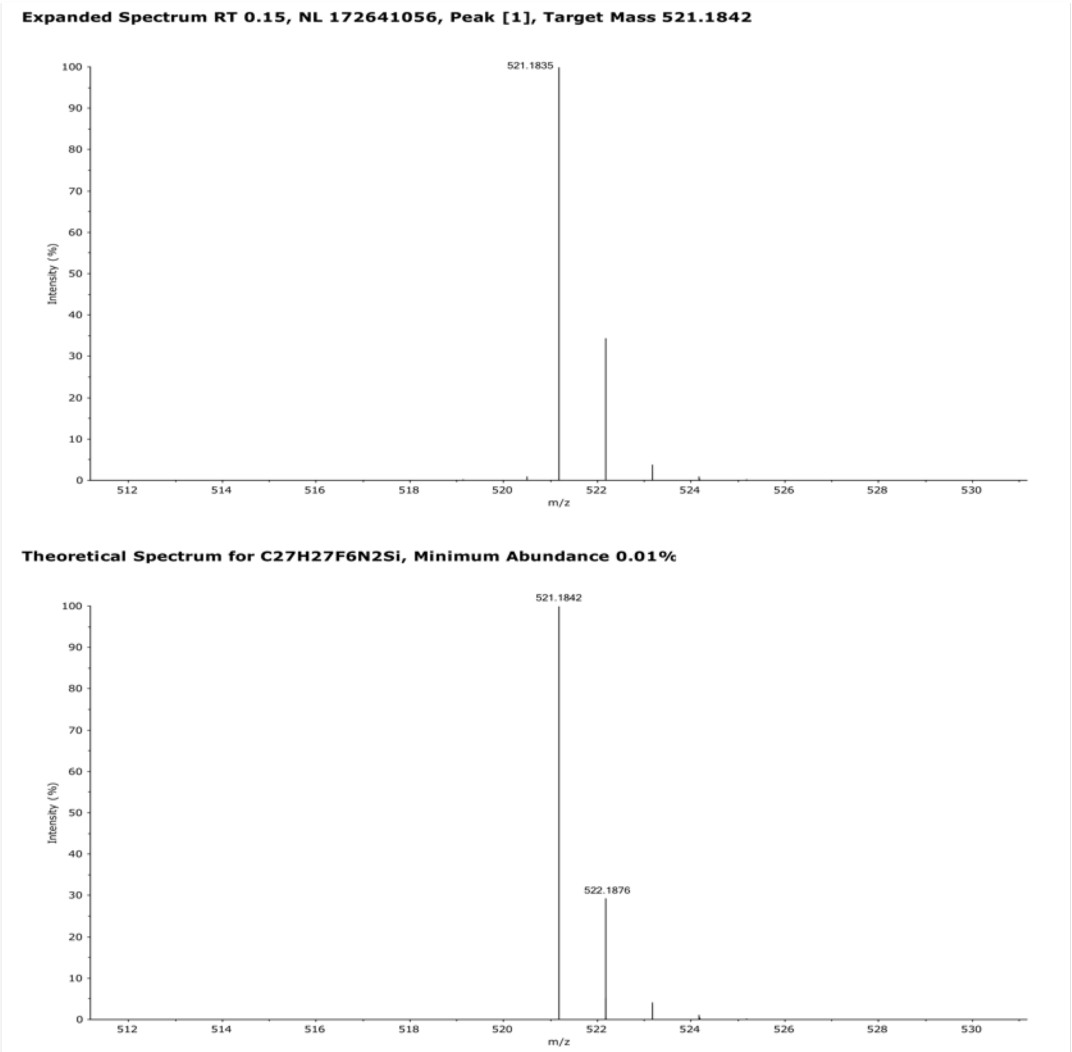

Measured Mass	Calculated Mass	Error (mDa)	Error (ppm)	Formula $[M+H]^+$	Response
521.1835	521.1842	-0.69	-1.33	$C_{27}H_{27}F_6N_2Si$	519834109

Figure S70. High-resolution ESI+ mass spectrum of **4**.

Compound **6**

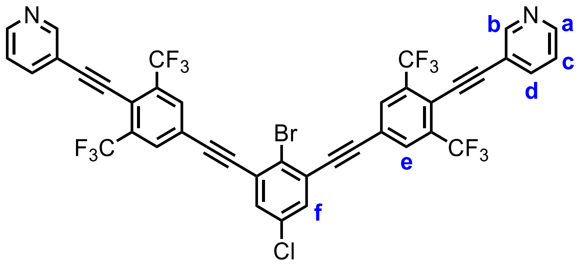

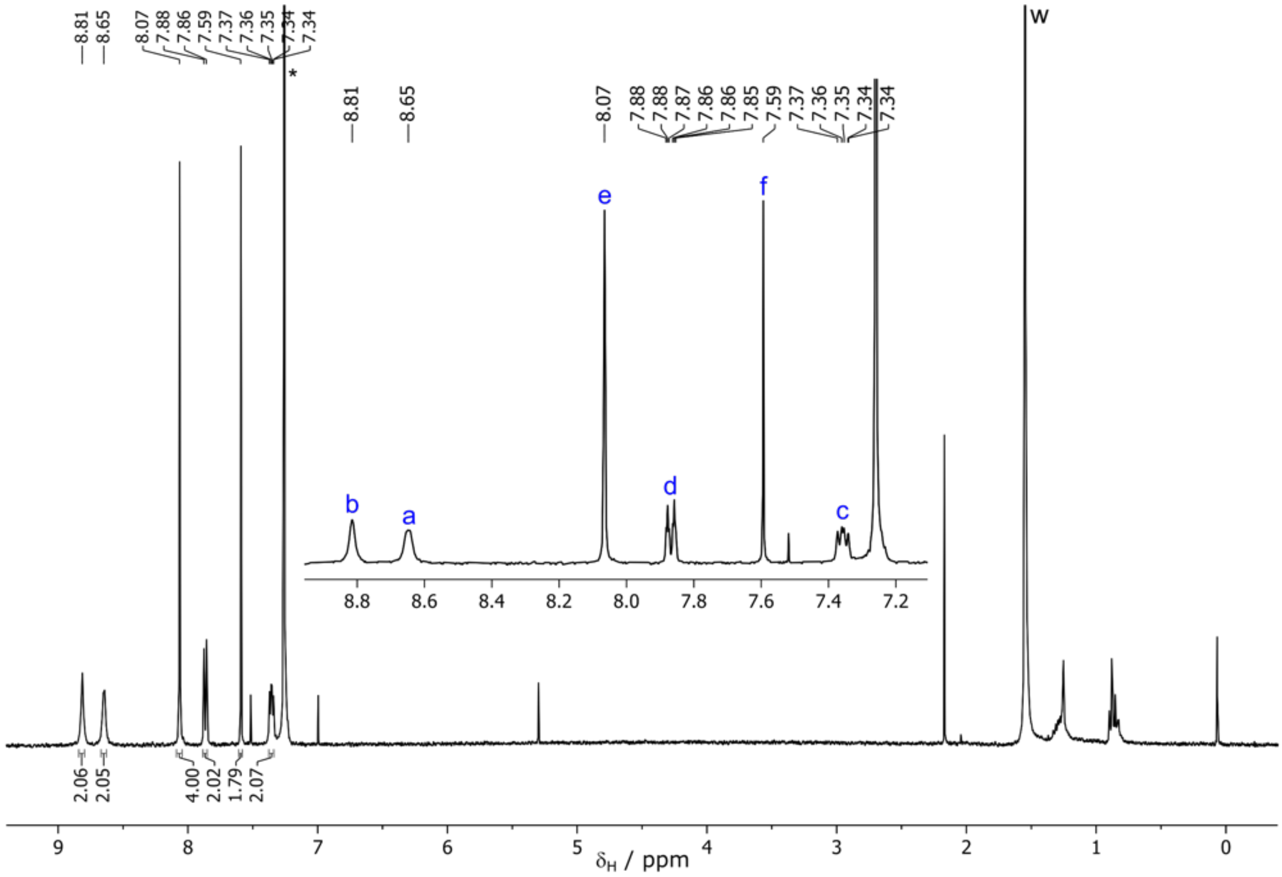

Figure S71. ^{1}H NMR spectrum of **6** (400 MHz, $CDCl_3$, 298 K). * = $CHCl_3$; w = water.

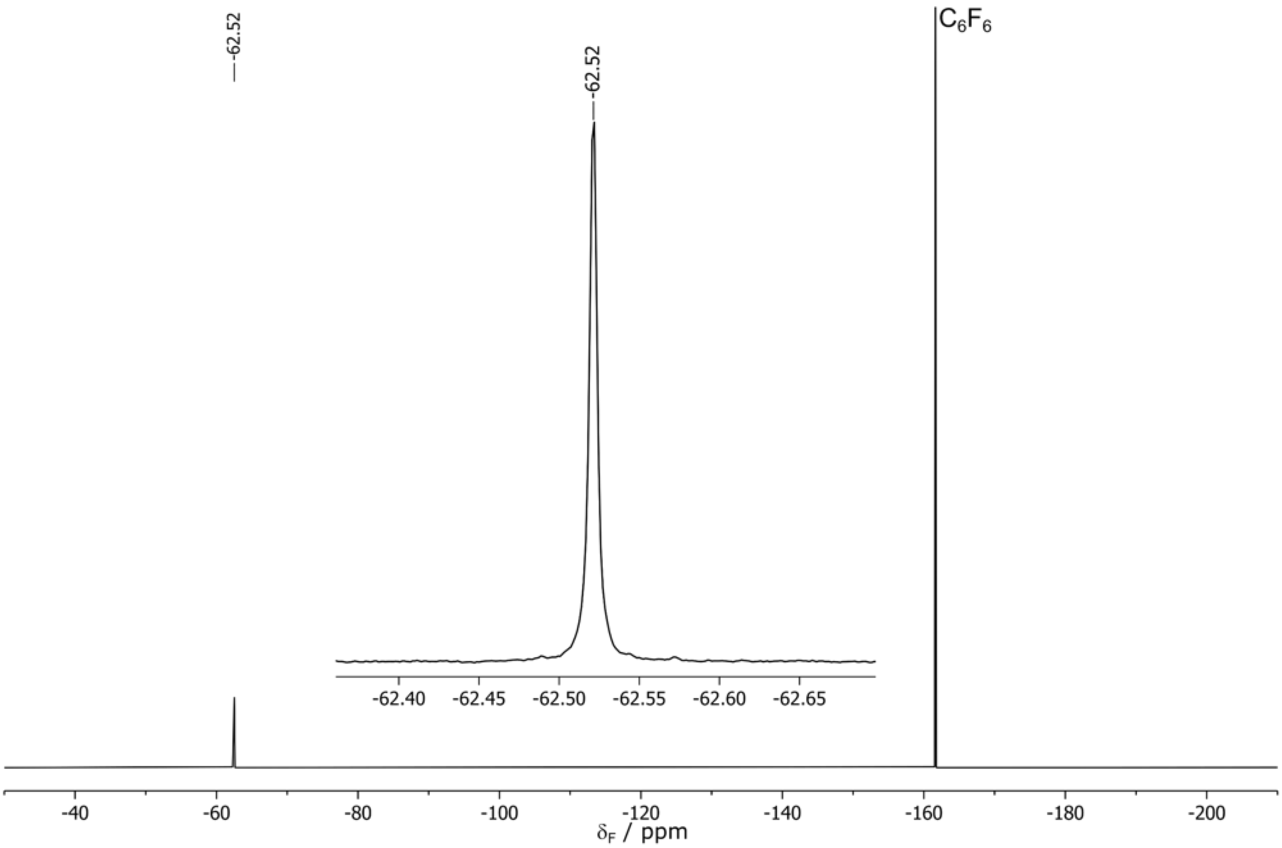

Figure S72. ^{19}F NMR spectrum of **6** (377 MHz, $CDCl_3$, 298 K). Referenced to C_6F_6 (δ_F = −161.64).

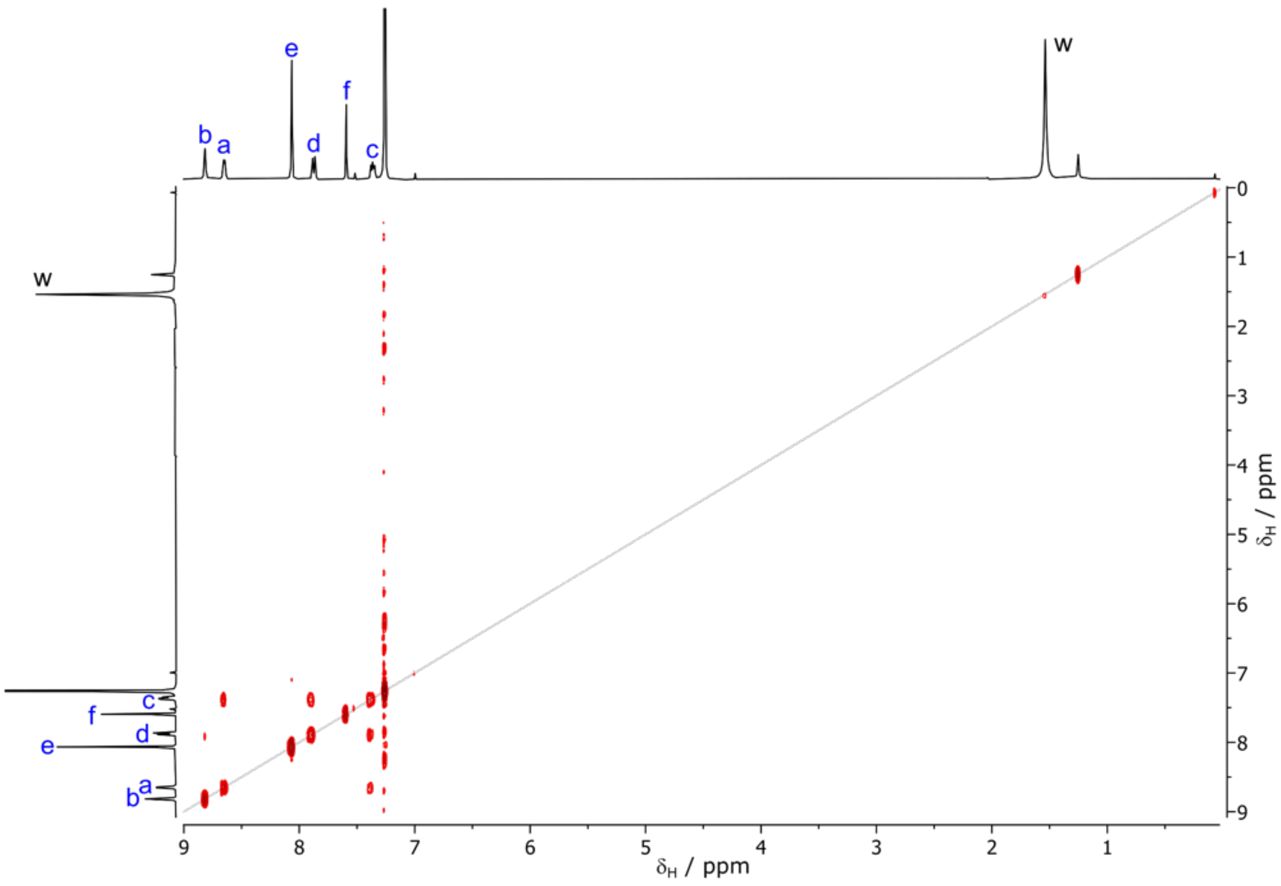

Figure S73. ^1H-^1H COSY spectrum of **6** (400 MHz, $CDCl_3$, 298 K). * = $CHCl_3$; w = water.

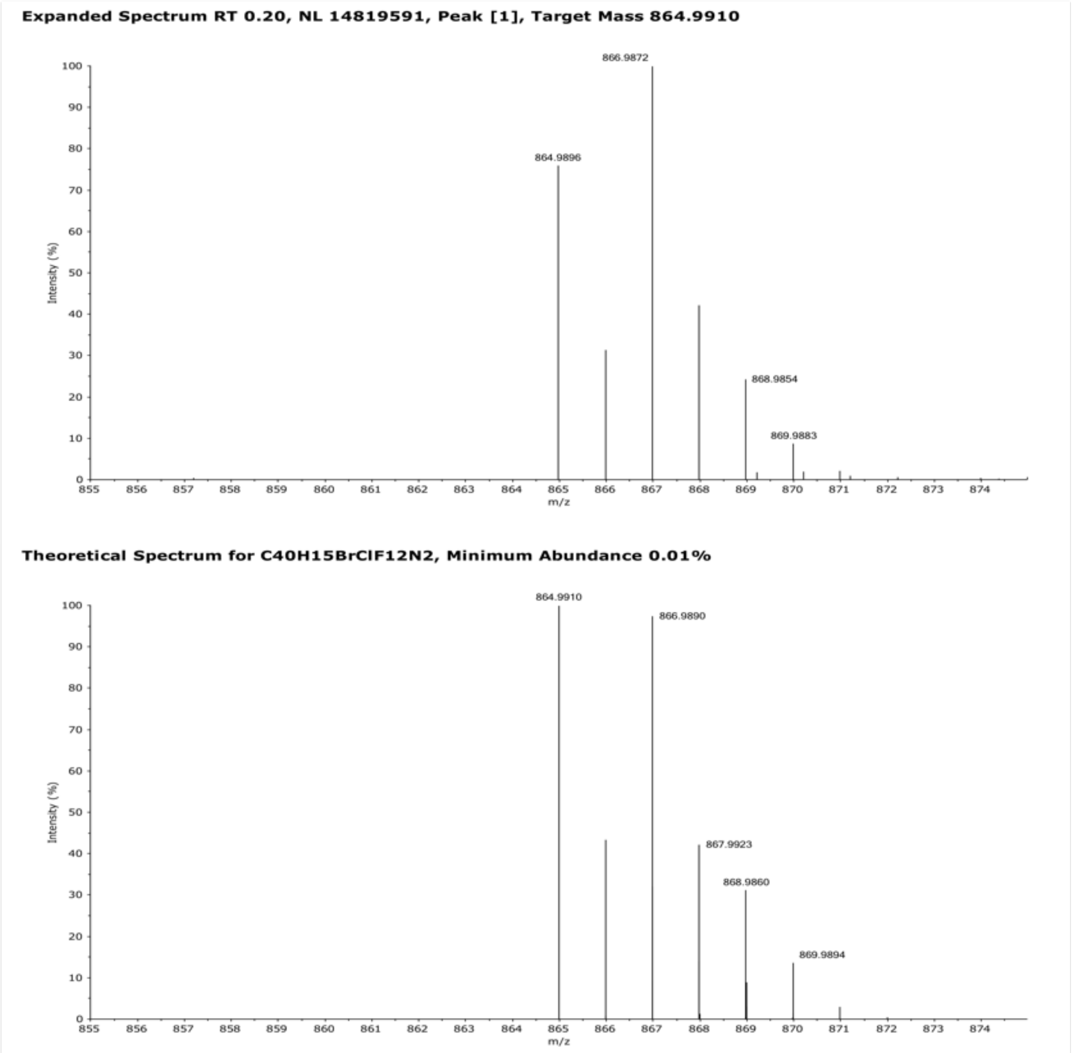

Measured Mass	Calculated Mass	Error (mDa)	Error (ppm)	Formula $[M+H]^+$	Response
864.9896	864.9910	-1.44	-1.67	$C_{40}H_{15}BrClF_{12}N_2$	53171809

Figure S74. High-resolution ESI+ mass spectrum of **6**.

Compound T3$_A$

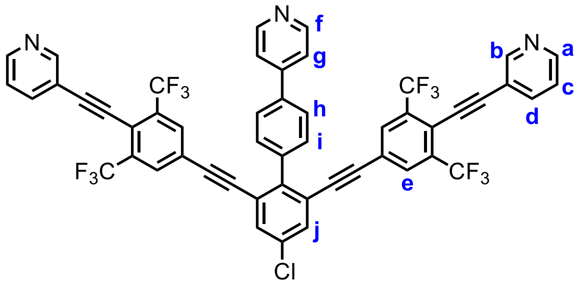

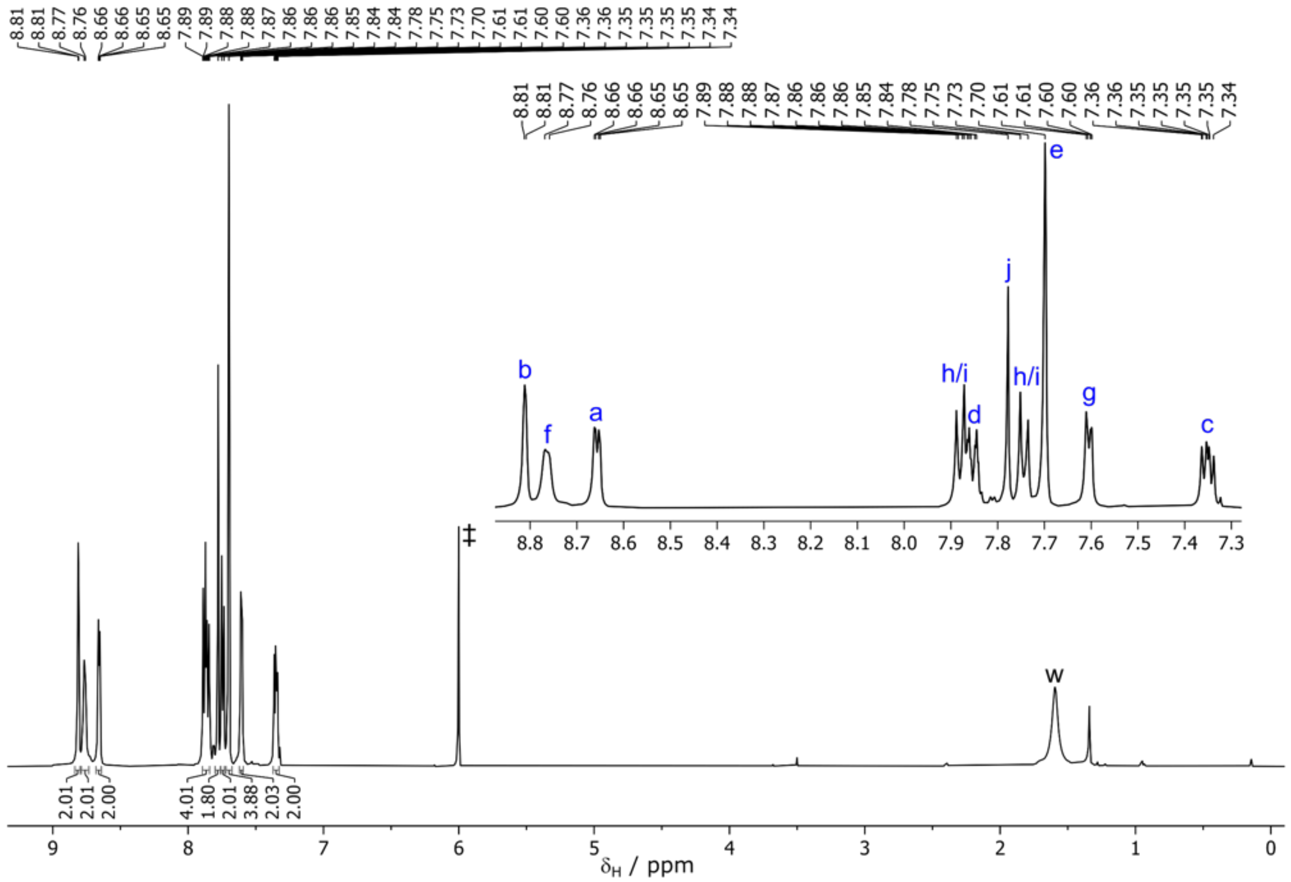

Figure S75. ^{1}H NMR spectrum of **T3$_A$** (600 MHz, $C_2D_2Cl_4$, 373 K). ‡ = C_2HDCl_4; w = water.

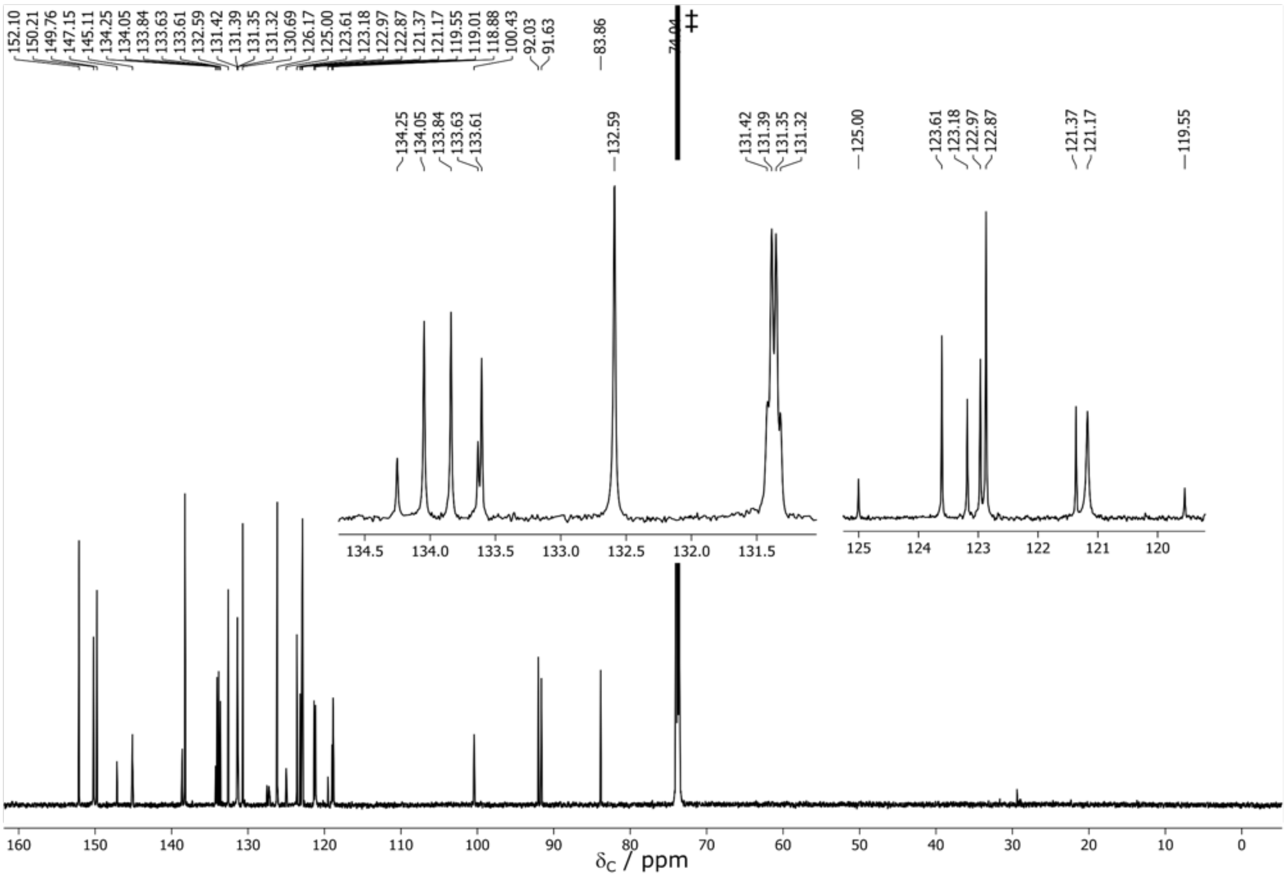

Figure S76. ^{13}C NMR spectrum of $\mathbf{T3_A}$ (151 MHz, $C_2D_2Cl_4$, 373 K). ‡ = $C_2D_2Cl_4$.

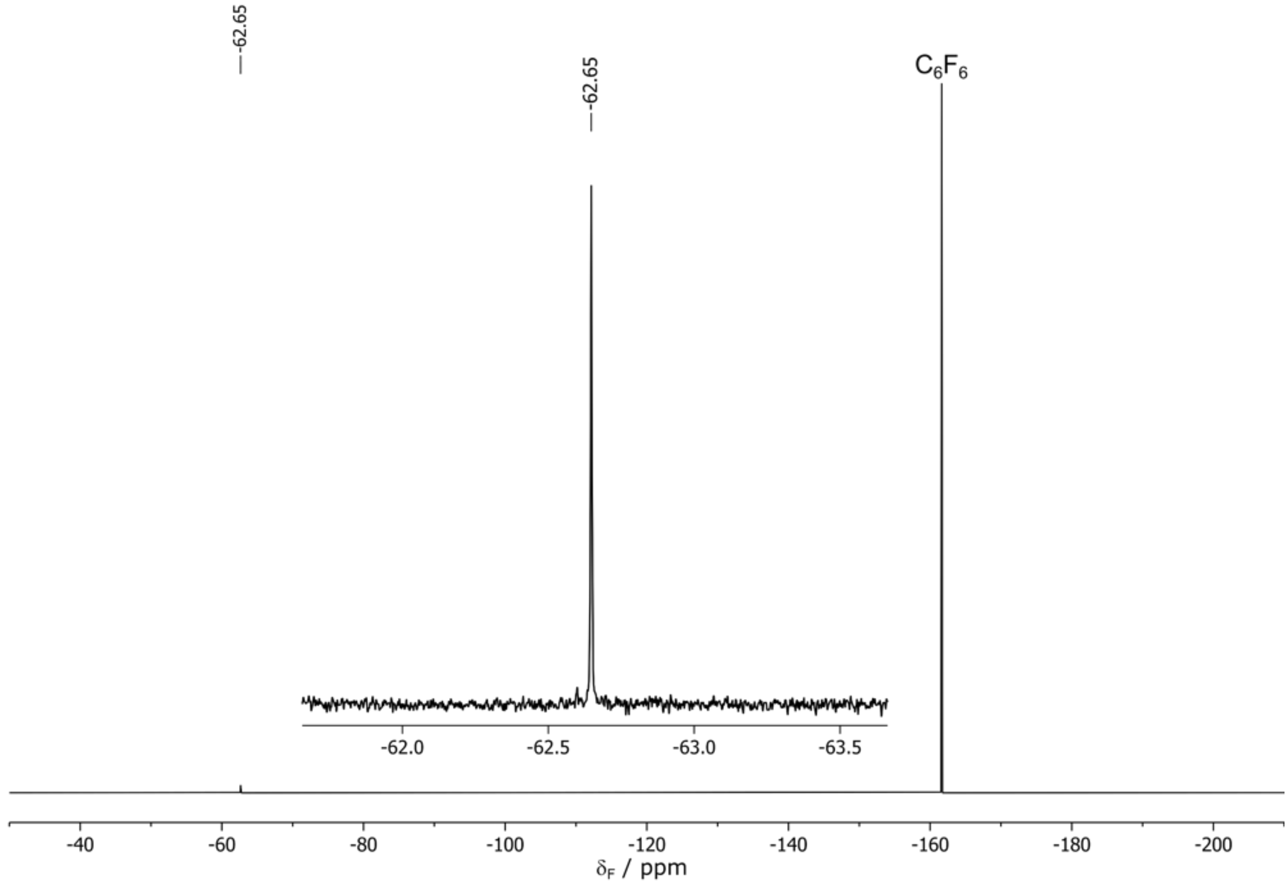

Figure S77. ^{19}F NMR spectrum of $\mathbf{T3_A}$ (377 MHz, $CHCl_3$, 298 K). Referenced to C_6F_6 (δ_F = −161.64 ppm).

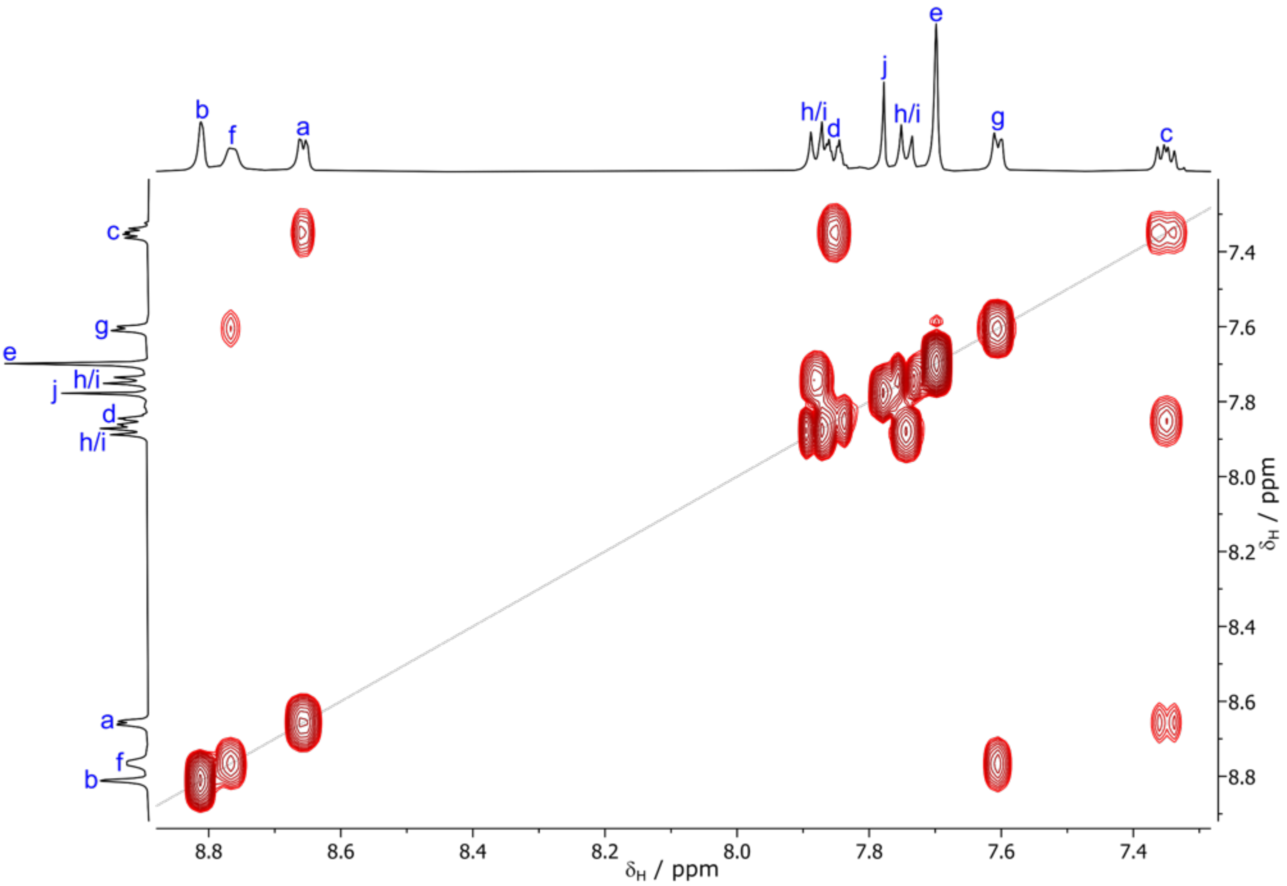

Figure S78. ^{1}H-^{1}H COSY spectrum of **T3**$_{\mathbf{A}}$ (600 MHz, $C_2D_2Cl_4$, 373 K).

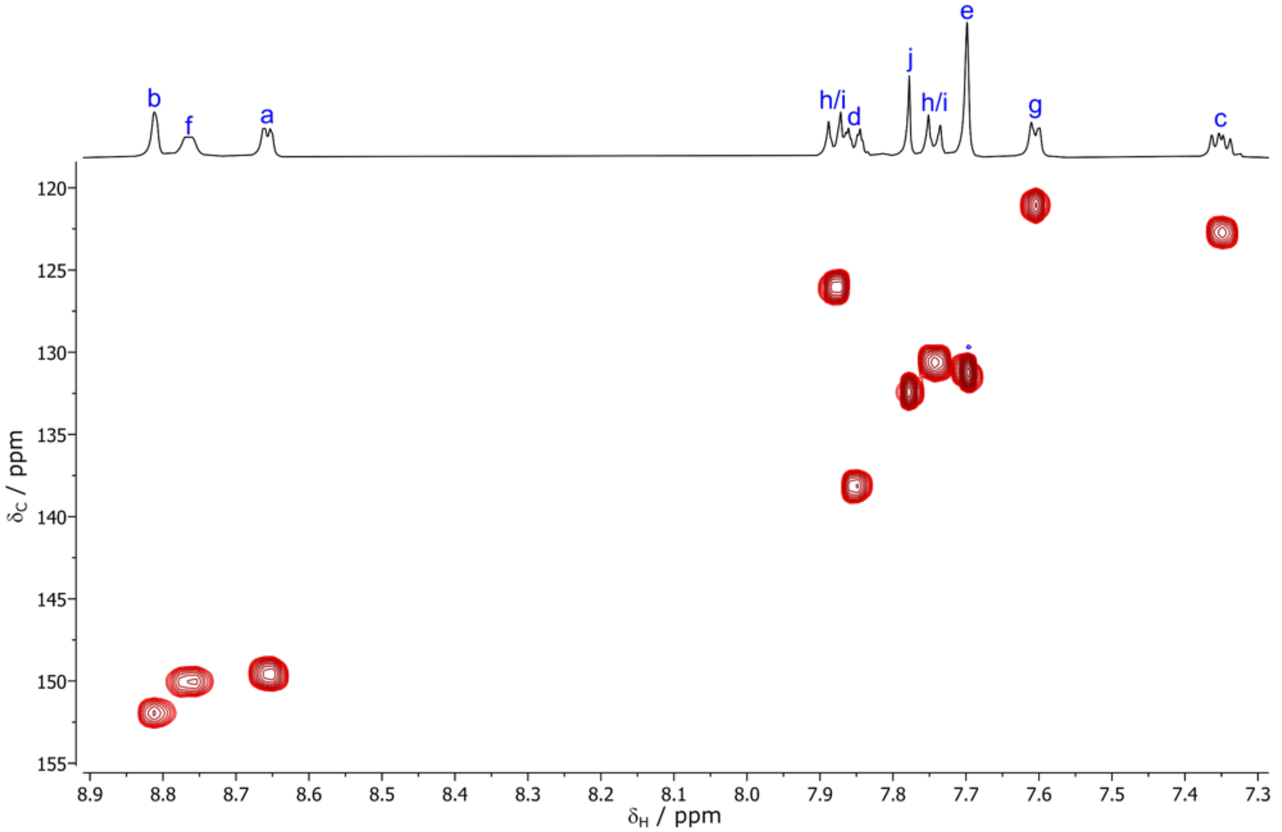

Figure S79. ^{1}H-^{13}C HSQC spectrum of **T3**$_{\mathbf{A}}$ (600 MHz, $C_2D_2Cl_4$, 373 K).

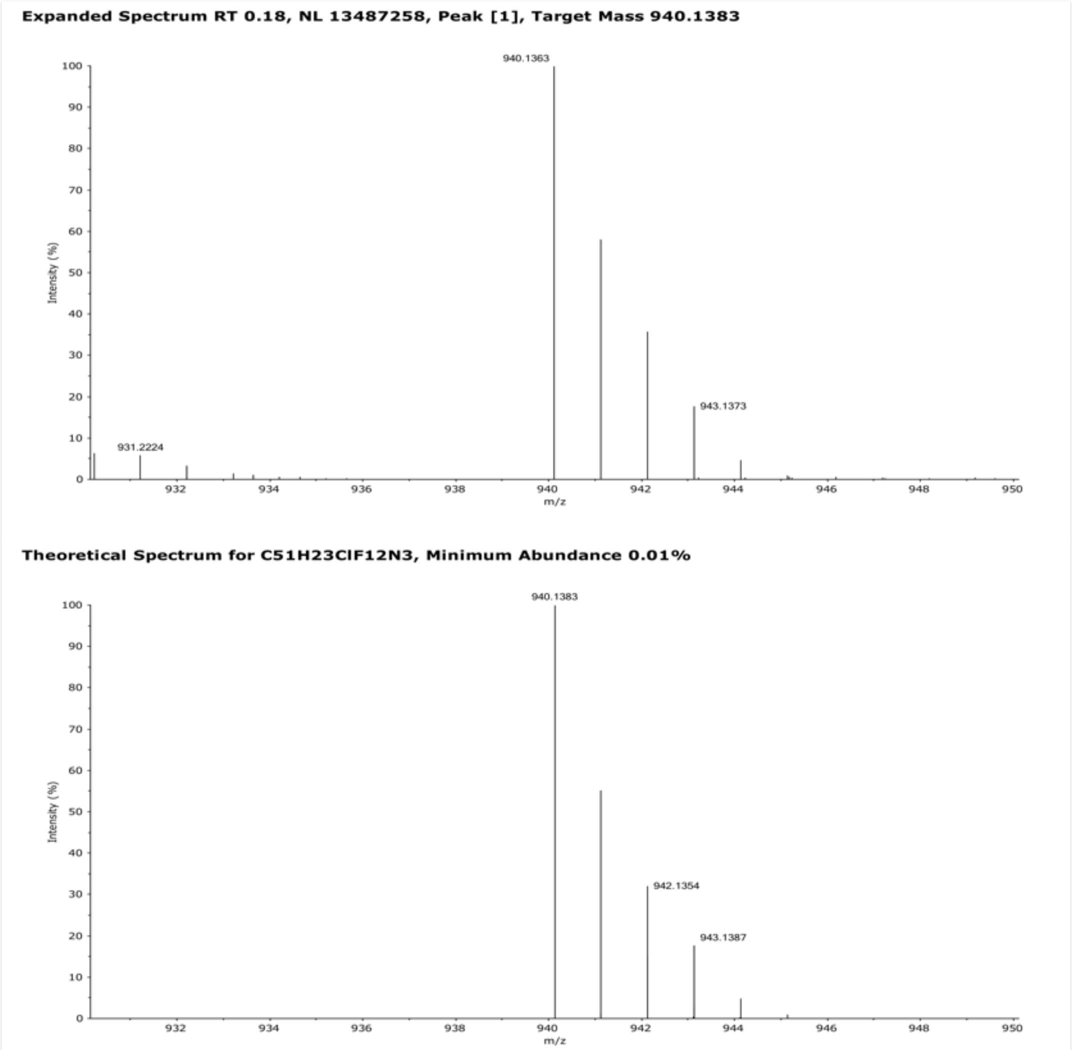

Measured Mass	Calculated Mass	Error (mDa)	Error (ppm)	Formula $[M+H]^+$	Response
940.1363	940.1383	-2.05	-2.18	$C_{51}H_{23}ClF_{12}N_3$	100337567

Figure S80. High-resolution ESI+ mass spectrum of **T3$_{\mathbf{A}}$**.

Compound 8

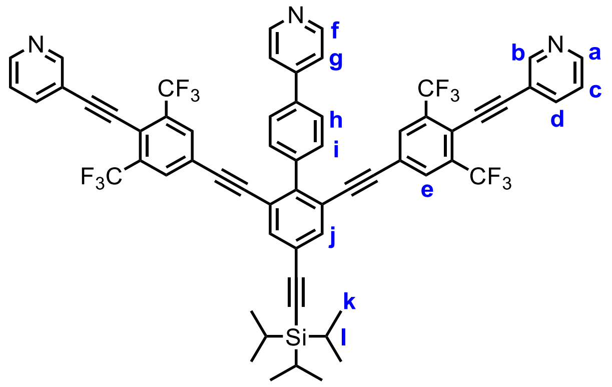

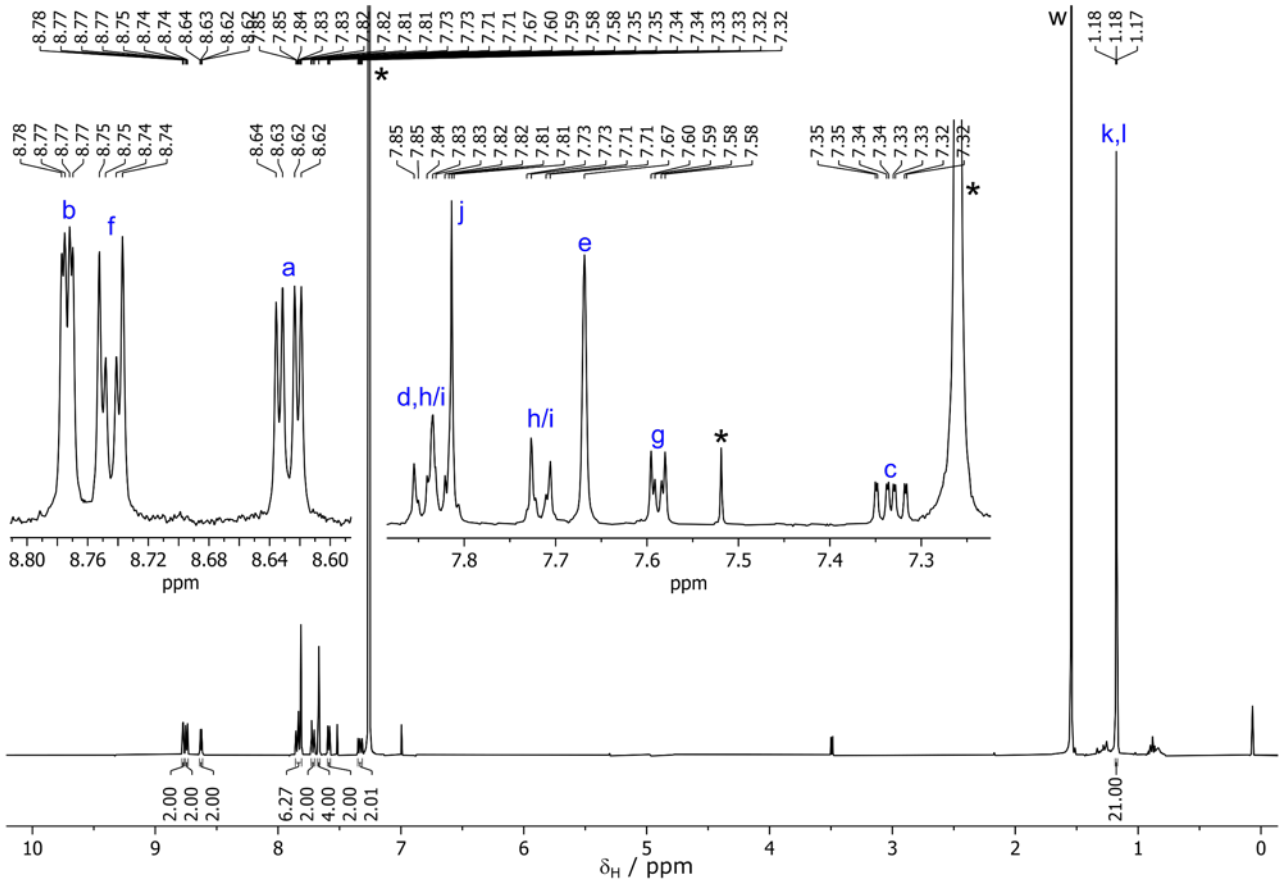

Figure S81. ^{1}H NMR spectrum of **8** (400 MHz, $CDCl_3$, 298 K). * = $CHCl_3$.

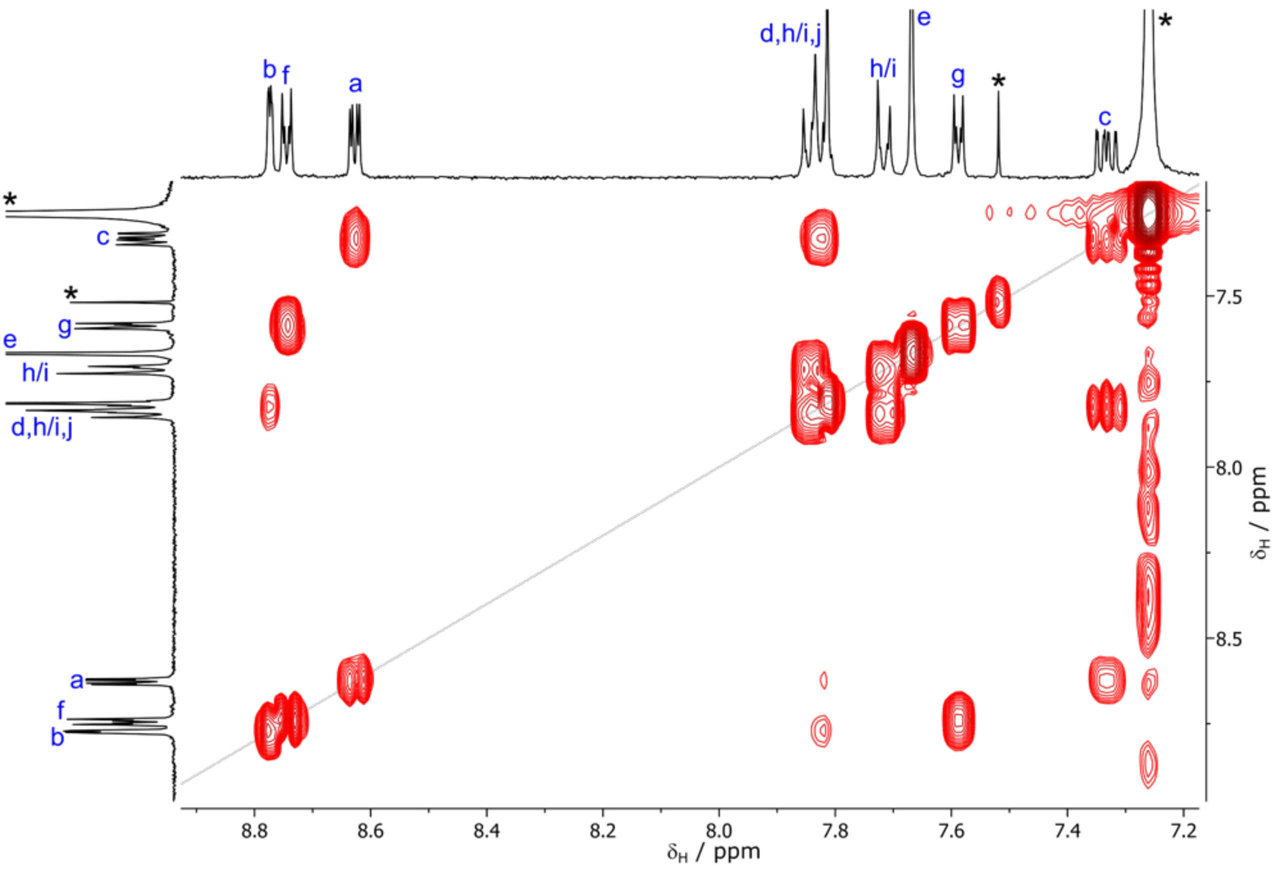

Figure S82. ^{1}H-^{1}H COSY spectrum of **8** (400 MHz, $CDCl_3$, 298 K). * = $CHCl_3$.

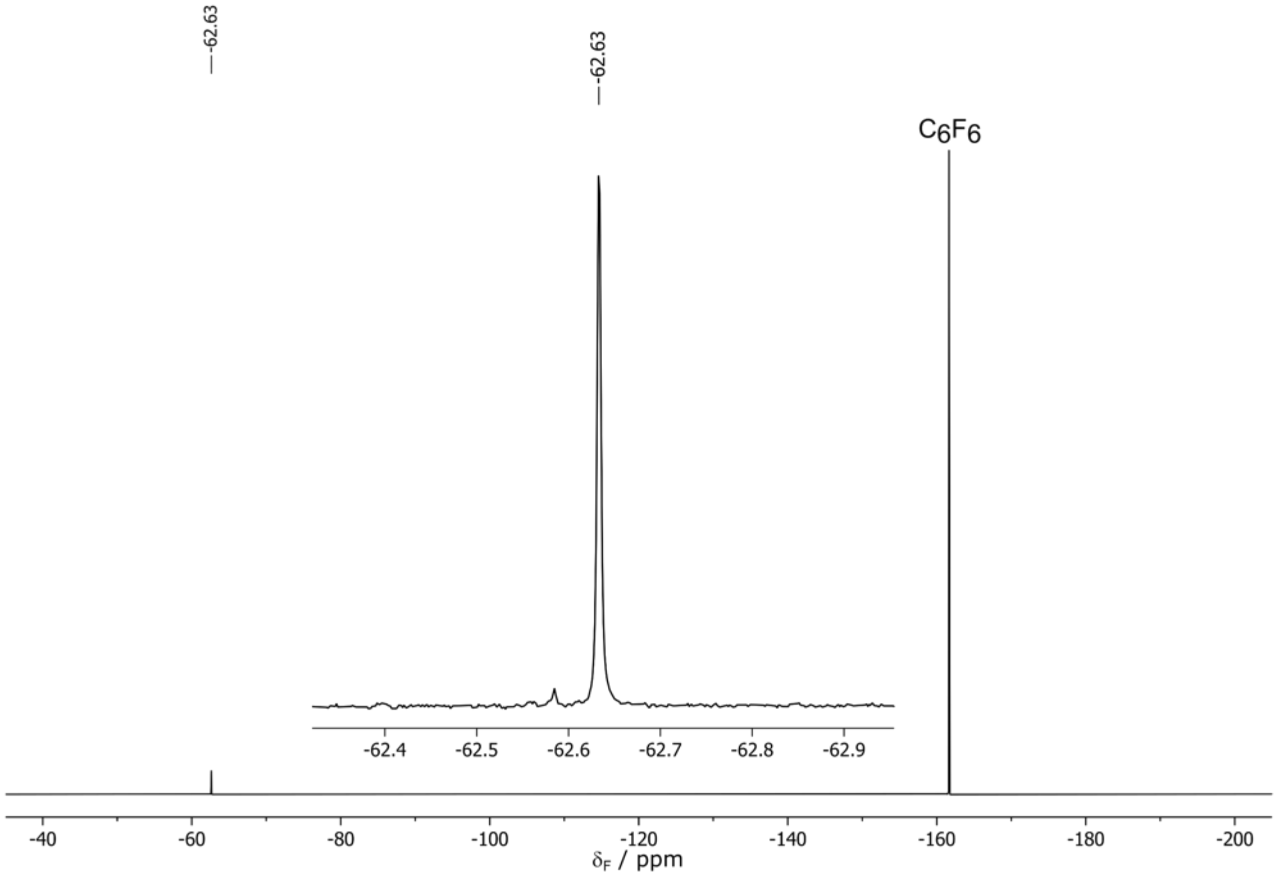

Figure S83. ^{19}F NMR spectrum of **8** (377 MHz, $CDCl_3$, 298 K). Referenced to C_6F_6 (δ_F = –161.64 ppm).

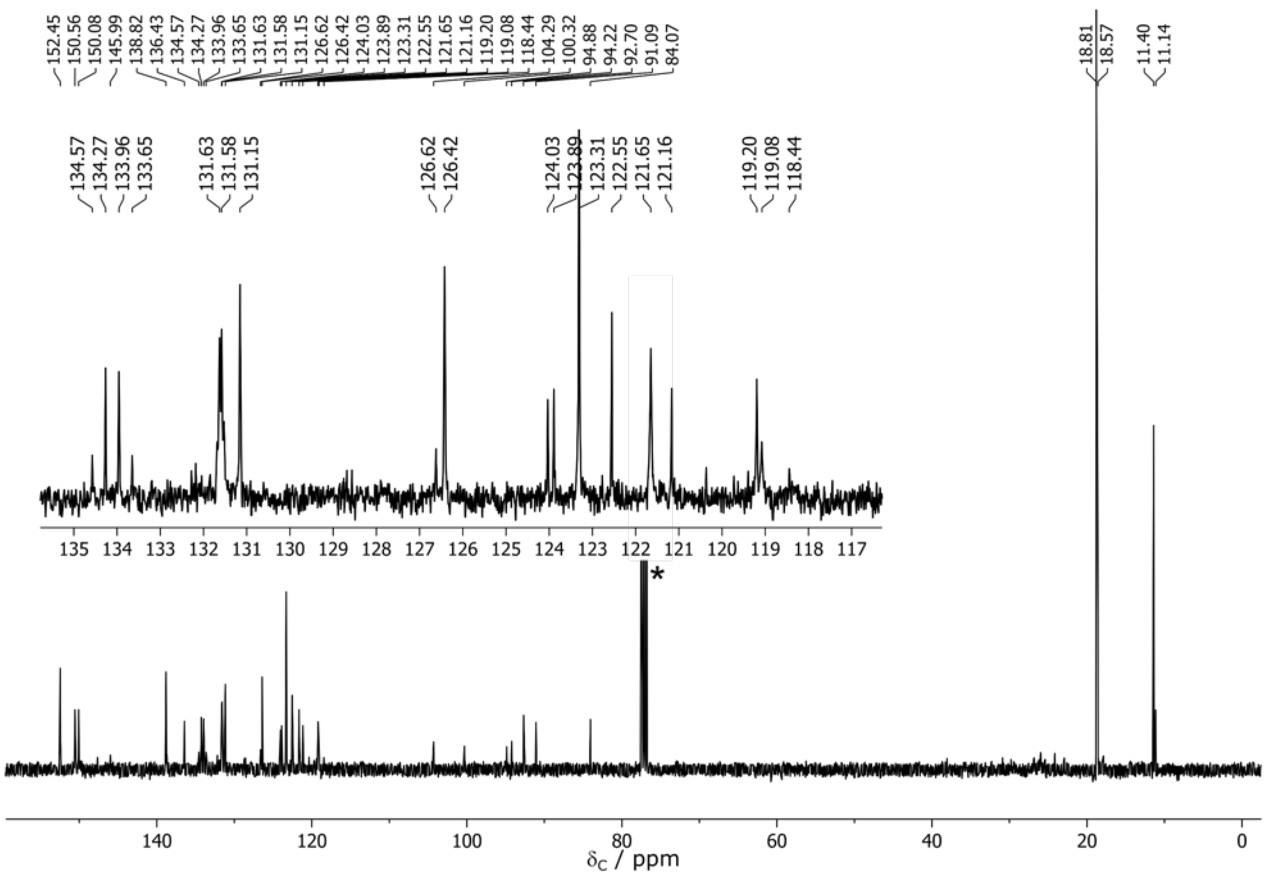

Figure S84. ^{13}C NMR spectrum of **8** (101 MHz, $CDCl_3$, 298 K). * = $CDCl_3$.

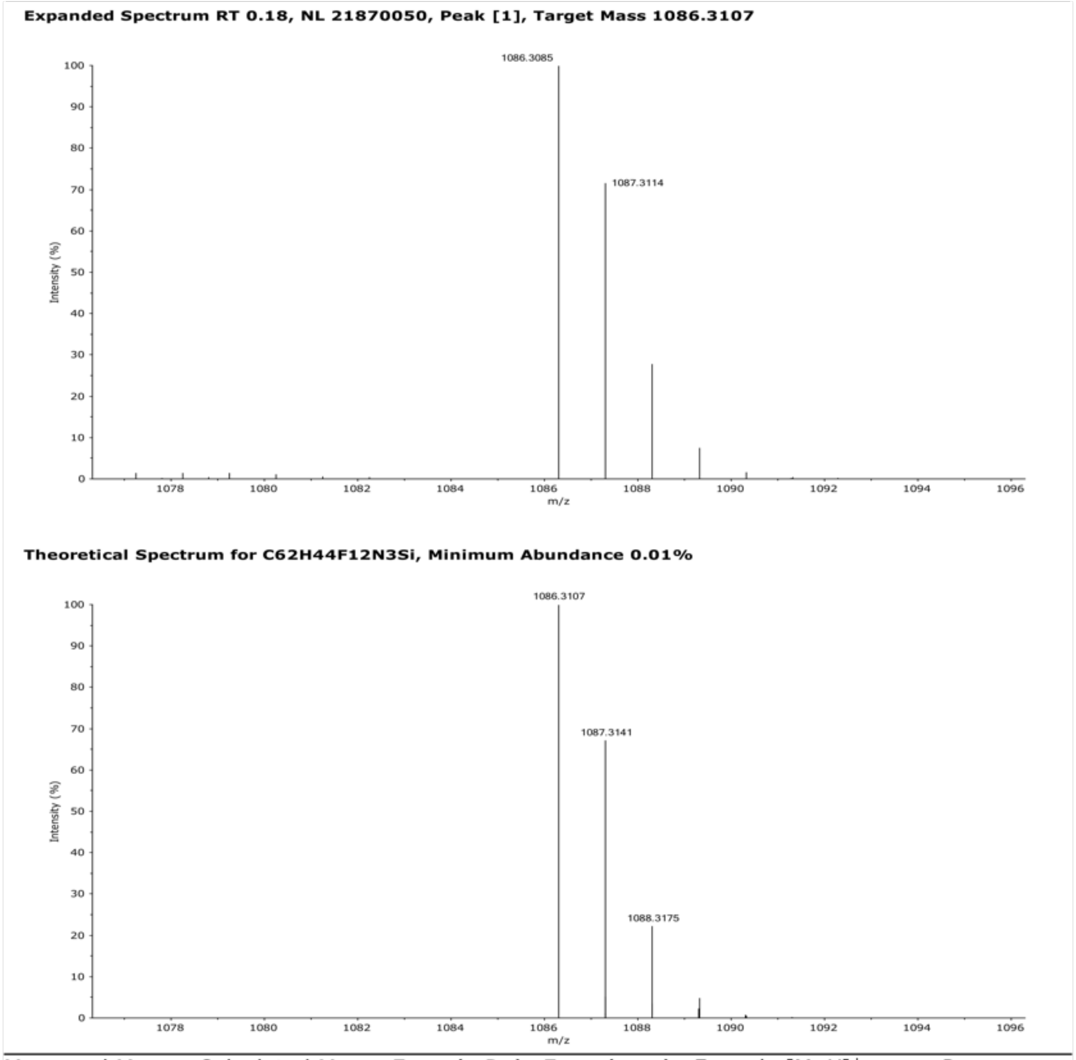

Measured Mass	Calculated Mass	Error (mDa)	Error (ppm)	Formula $[M+H]^+$	Response
1086.3085	1086.3107	-2.27	-2.09	$C_{62}H_{44}F_{12}N_3Si$	175449965

Figure S85. High-resolution ESI+ mass spectrum of **8**.

Compound S5

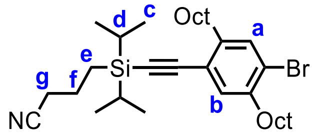

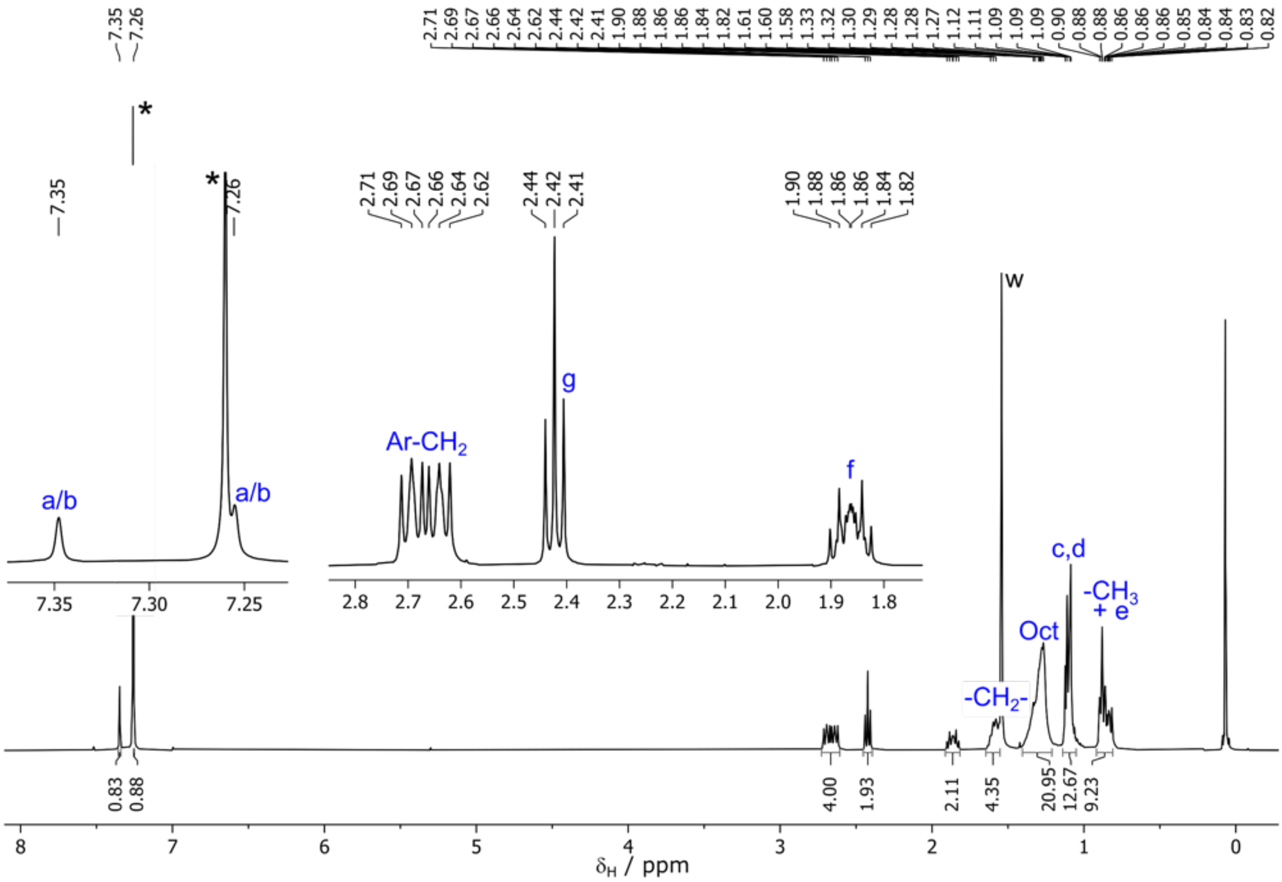

Figure S86. ^{1}H NMR spectrum of **S5** (400 MHz, $CDCl_3$, 298 K). * = $CHCl_3$; w = water.

Compound 15

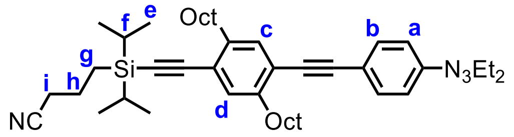

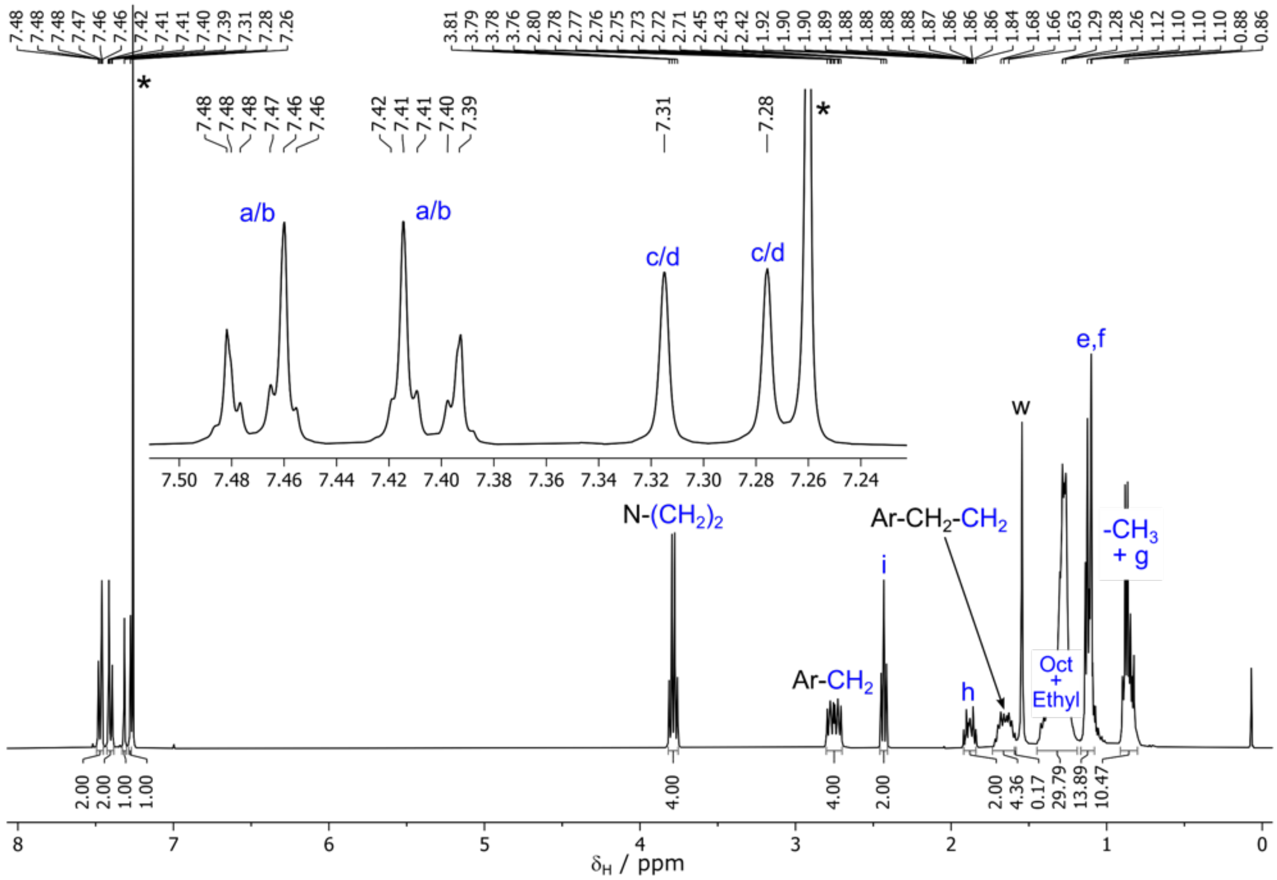

Figure S87. ^{1}H NMR spectrum of **15** (400 MHz, $CDCl_3$, 298 K). * = $CHCl_3$; w = water.

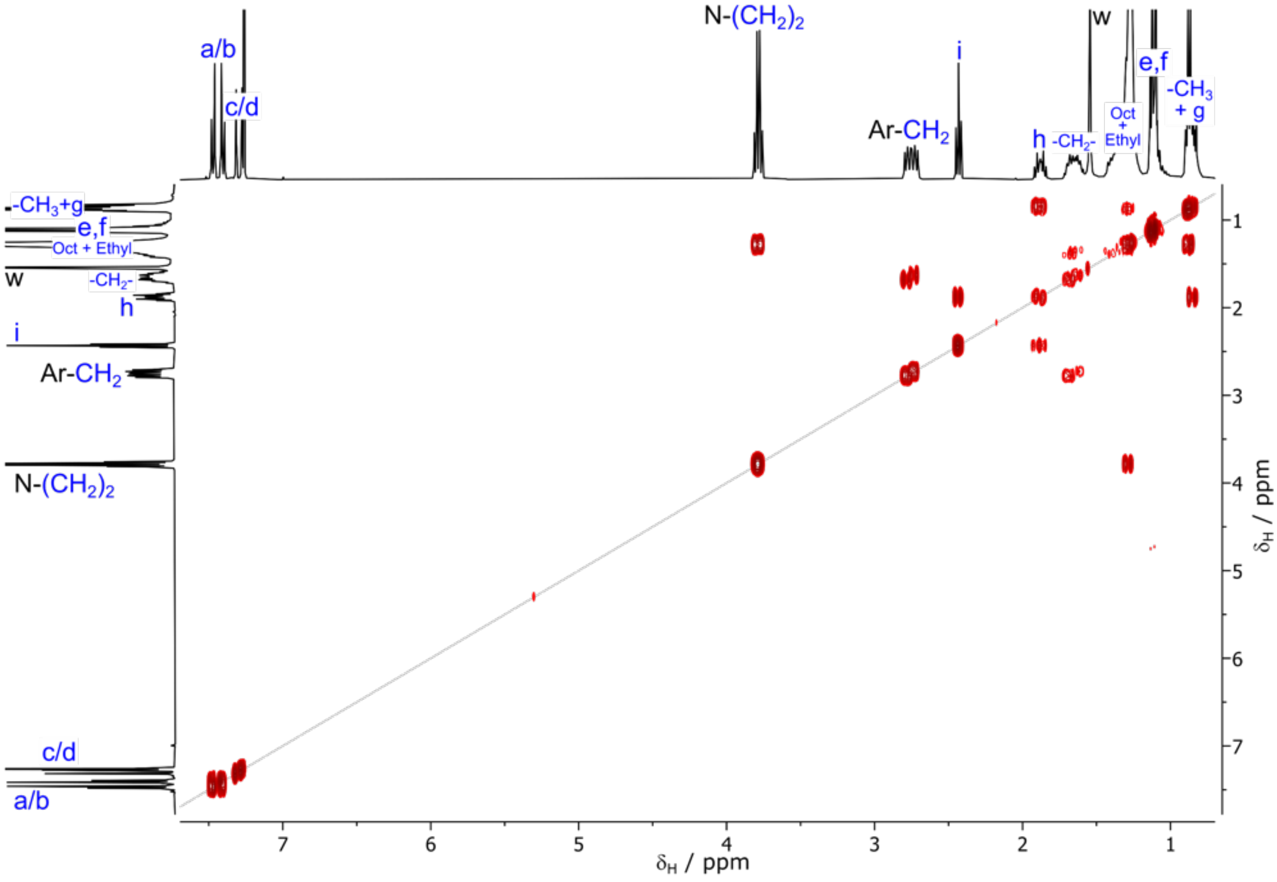

Figure S88. ^{1}H-^{1}H COSY spectrum of **15** (400 MHz, $CDCl_3$, 298 K). * = $CHCl_3$; w = water.

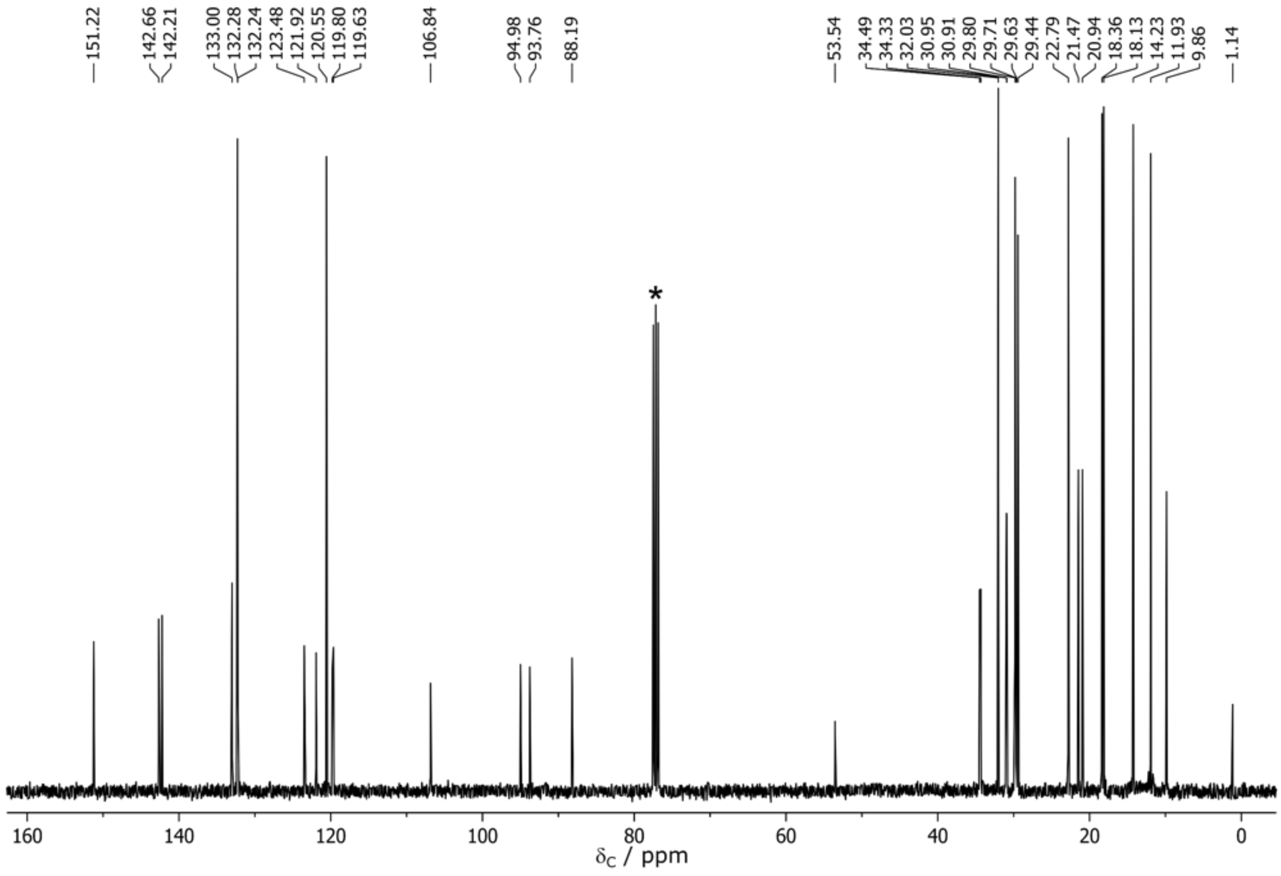

Figure S89. ^{13}C NMR spectrum of **15** (101 MHz, $CDCl_3$, 298 K). * = $CDCl_3$.

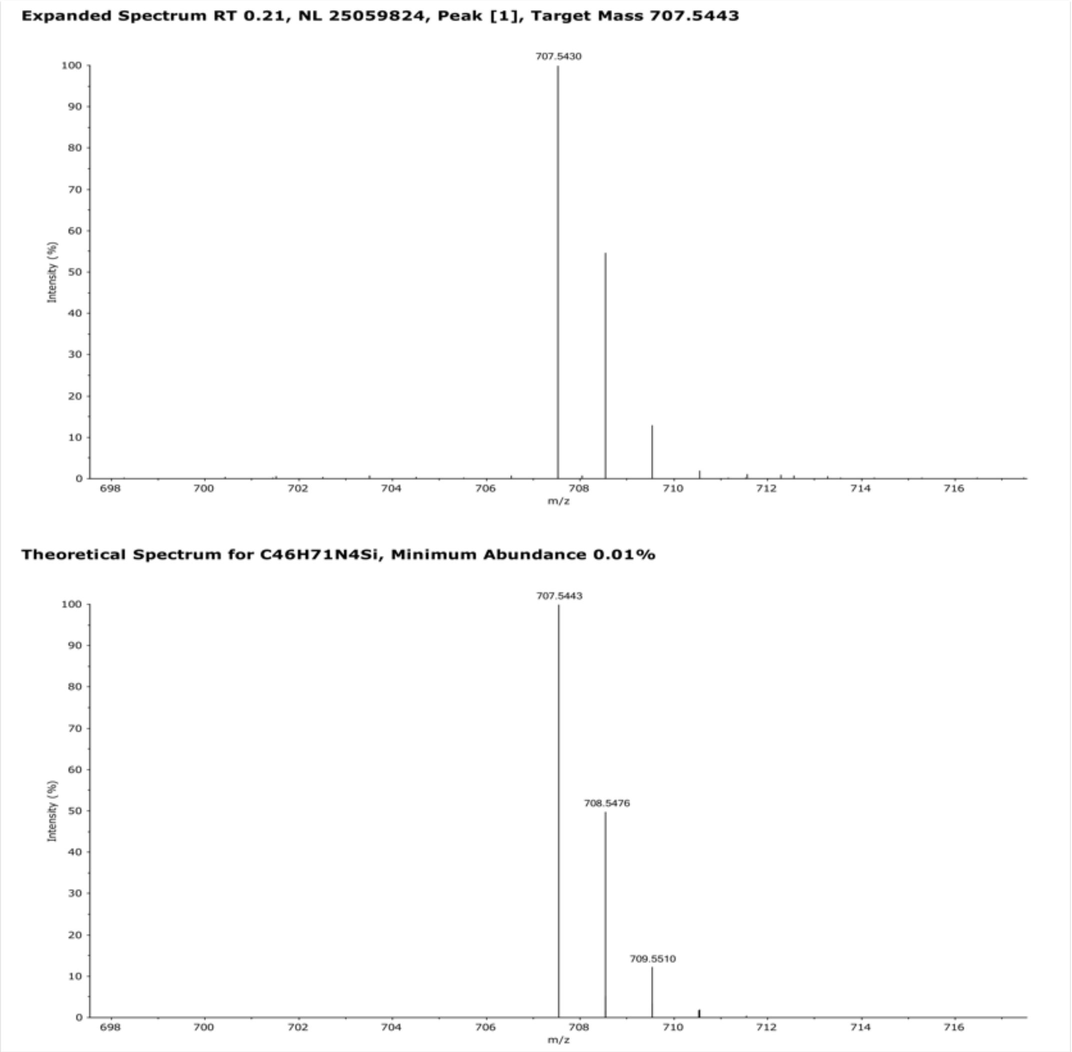

Measured Mass	Calculated Mass	Error (mDa)	Error (ppm)	Formula [M+H]+	Response
707.5430	707.5443	-1.22	-1.73	$C_{46}H_{71}N_4Si$	268211505

Figure S90. High-resolution ESI+ mass spectrum of **15**.

Compound 17

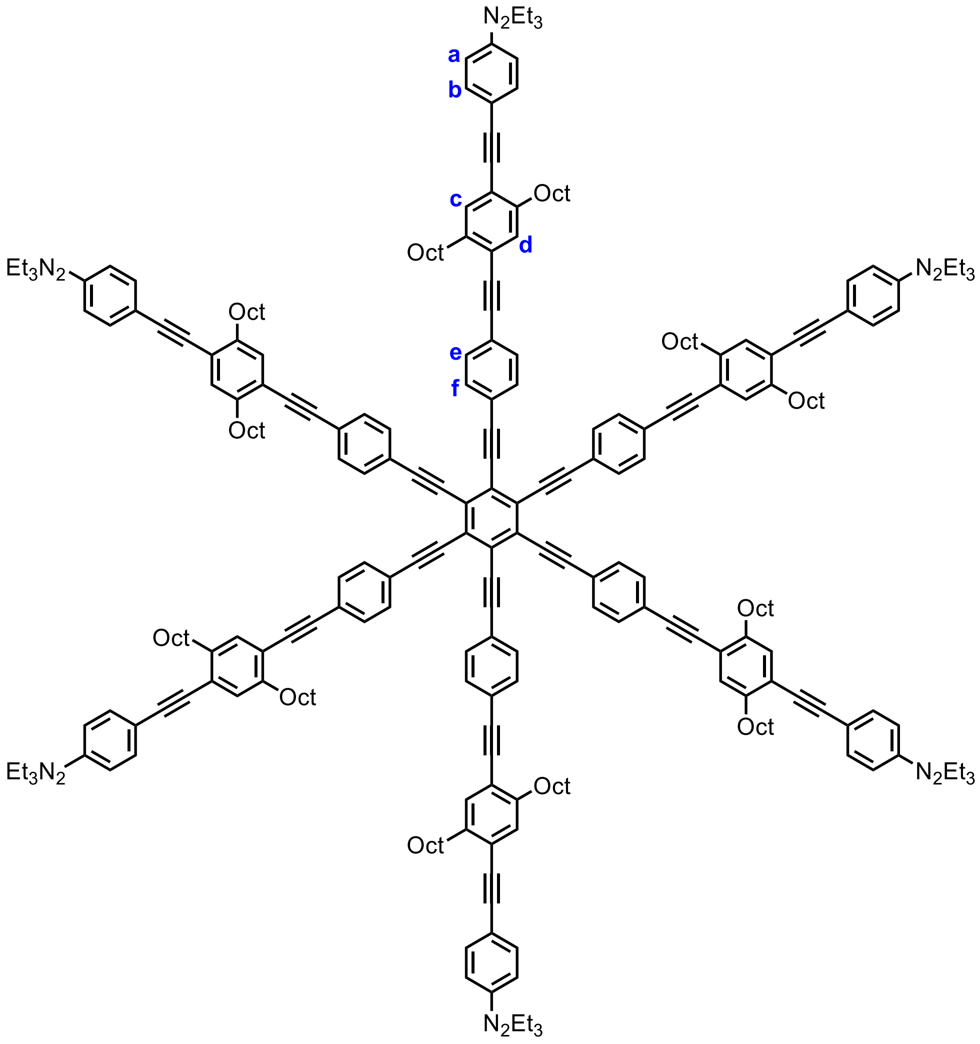
N2Et3
a
b
c
d
Oct
Oct
e
f
Et3N2
N2Et3
Oct
Oct
Oct
Oct
Oct
Oct
Oct
Oct
Oct
Oct
Oct
Oct
Et3N2
N2Et3
N2Et3

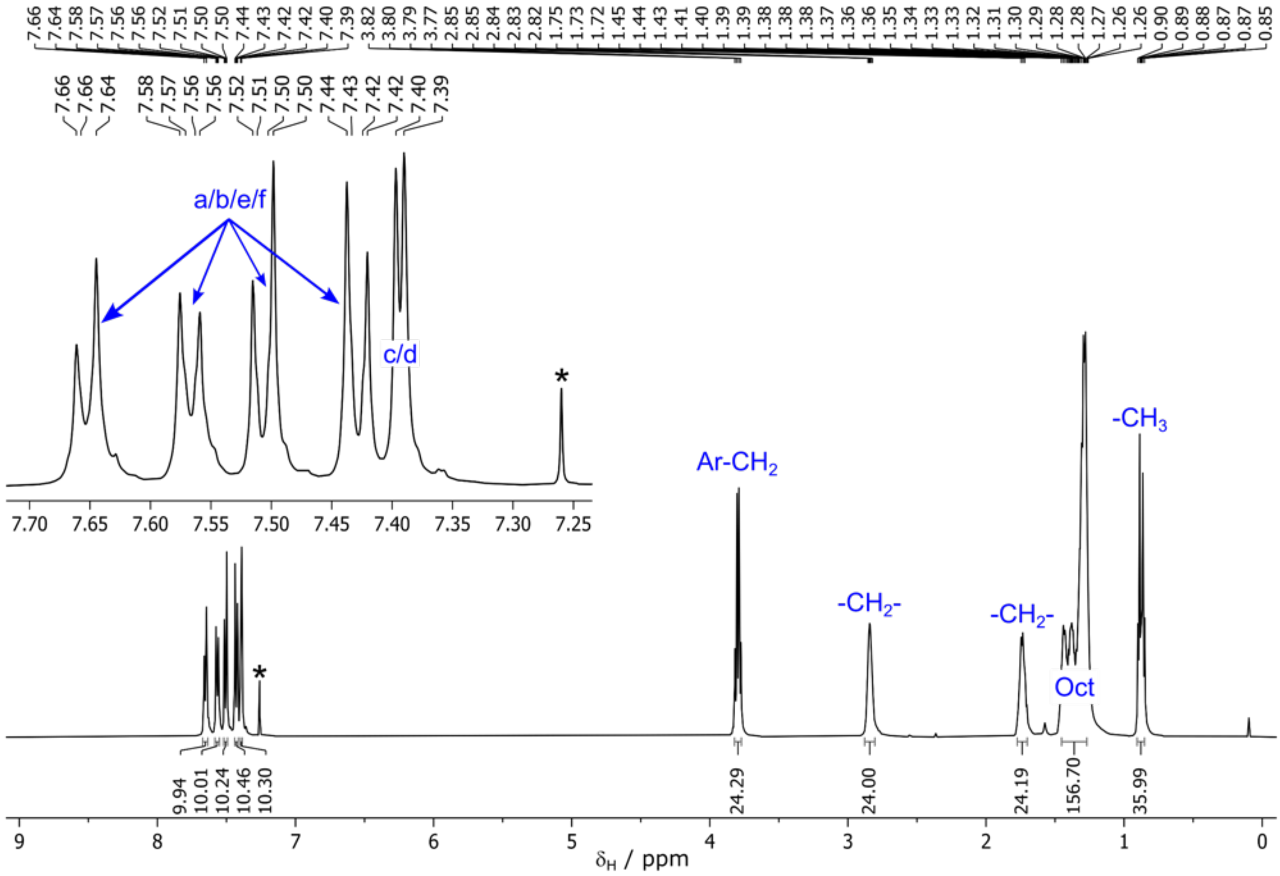

Figure S91. ^{1}H NMR spectrum of **17** (500 MHz, $CDCl_3$, 298 K). * = $CHCl_3$; w = water.

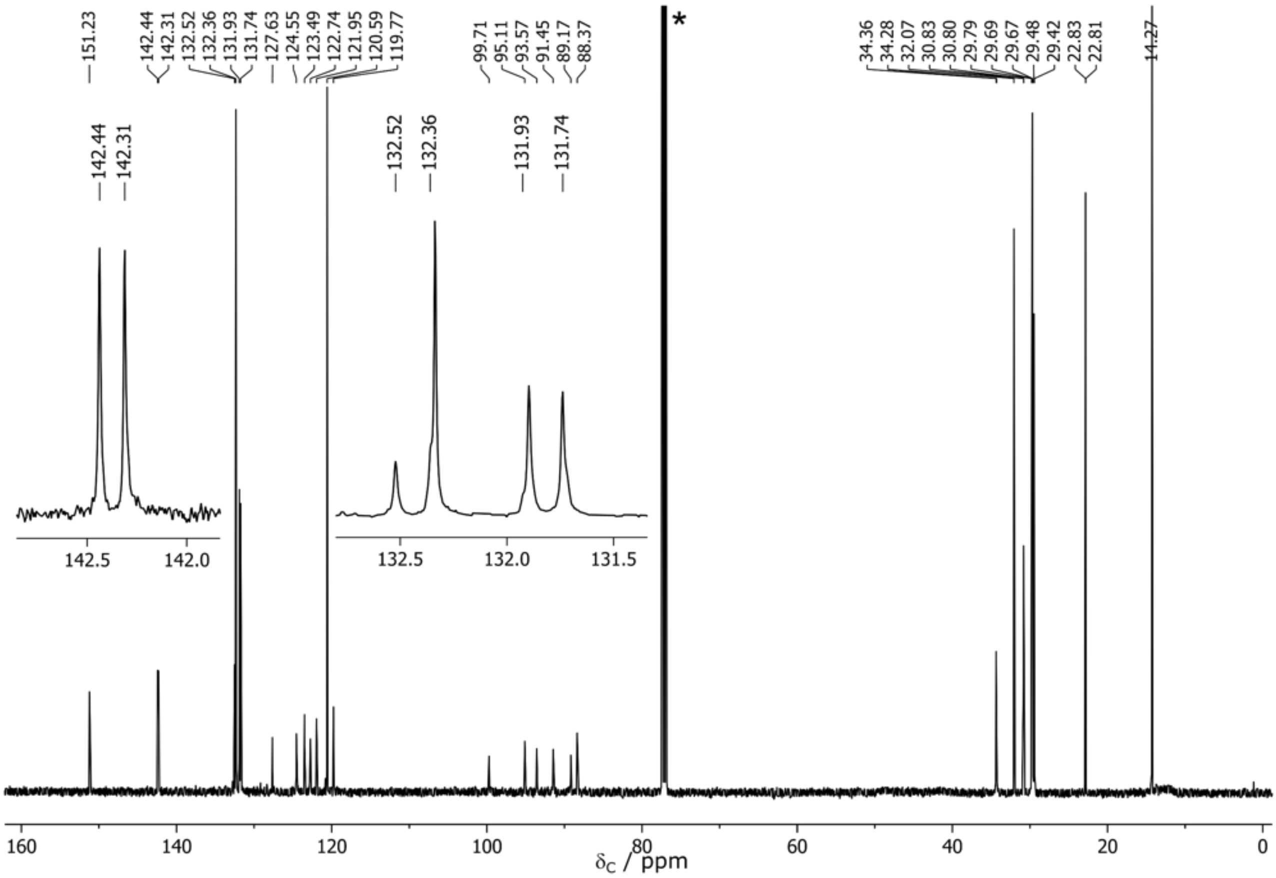

Figure S92. ^{13}C NMR spectrum of **17** (126 MHz, $CDCl_3$, 298 K). * = $CDCl_3$.

Compound 18

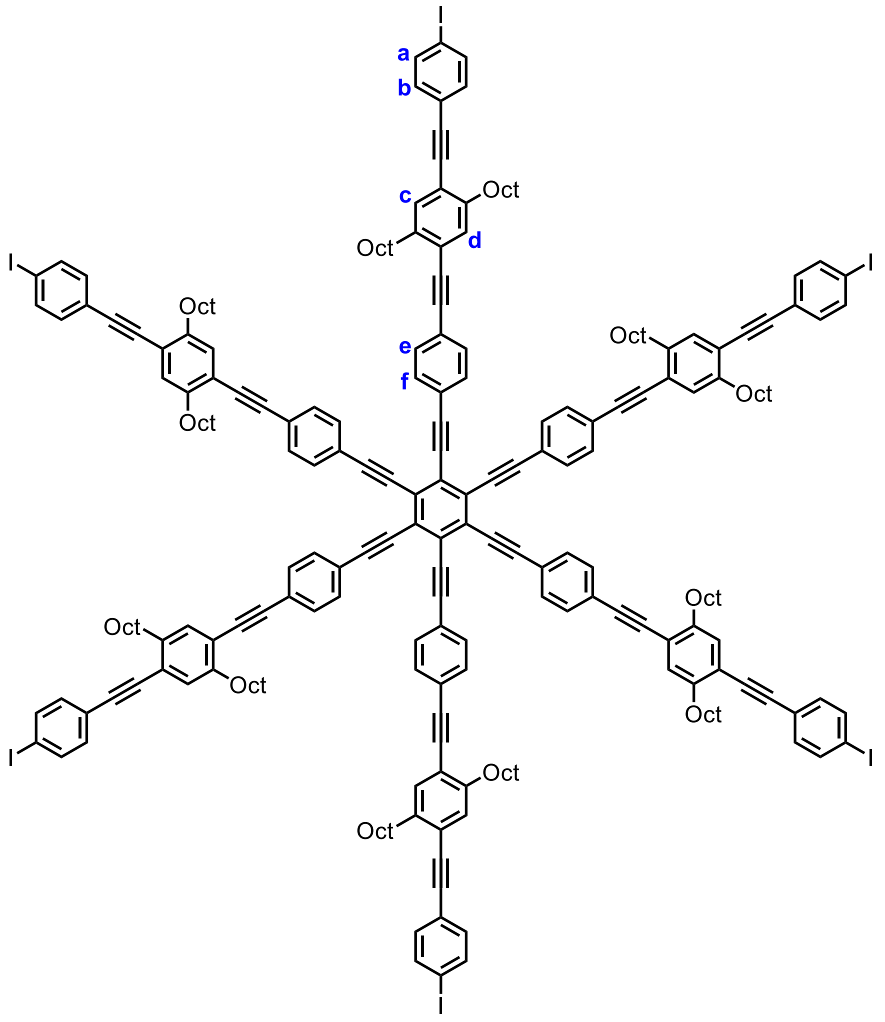

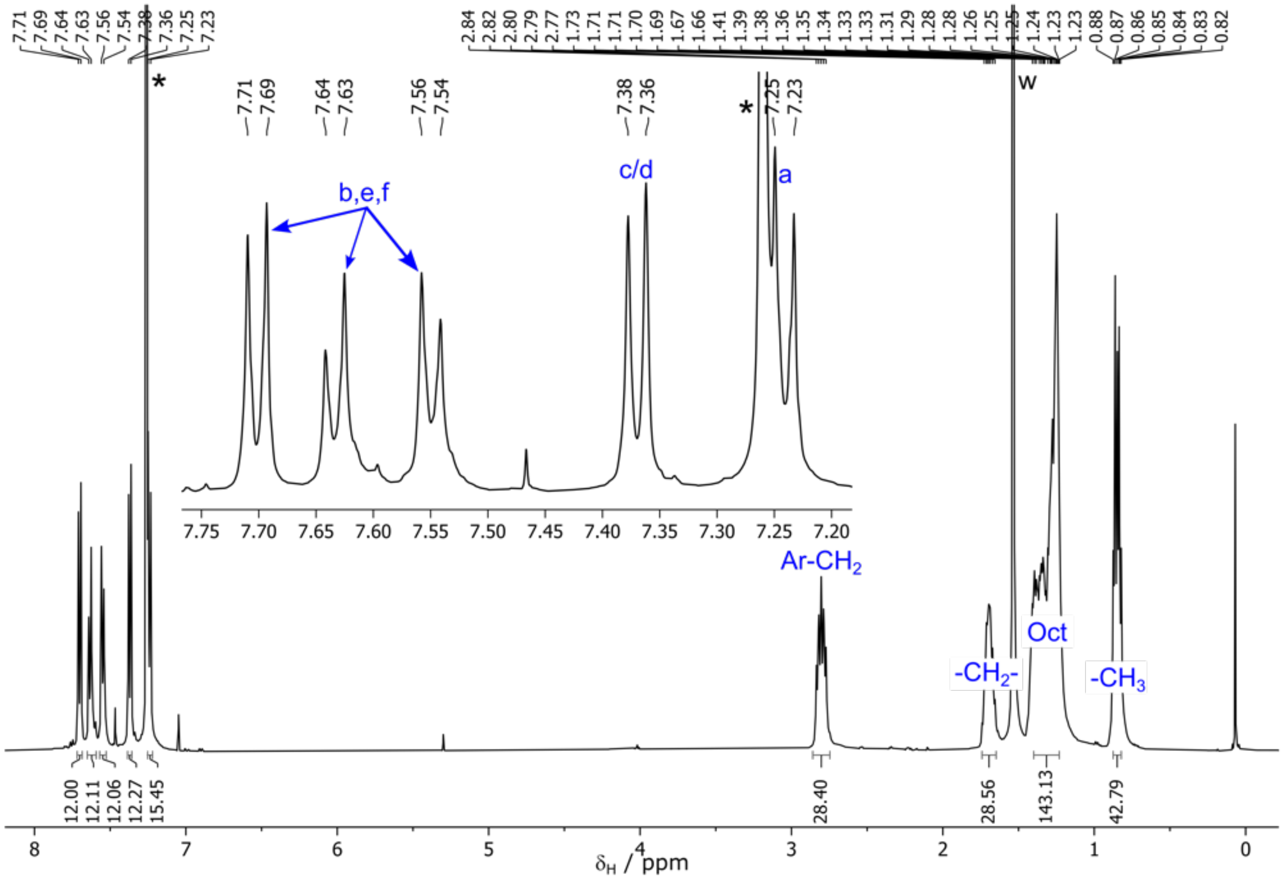

Figure S93. ^{1}H NMR spectrum of **18** (500 MHz, $CDCl_3$, 298 K). * = $CHCl_3$; w = water.

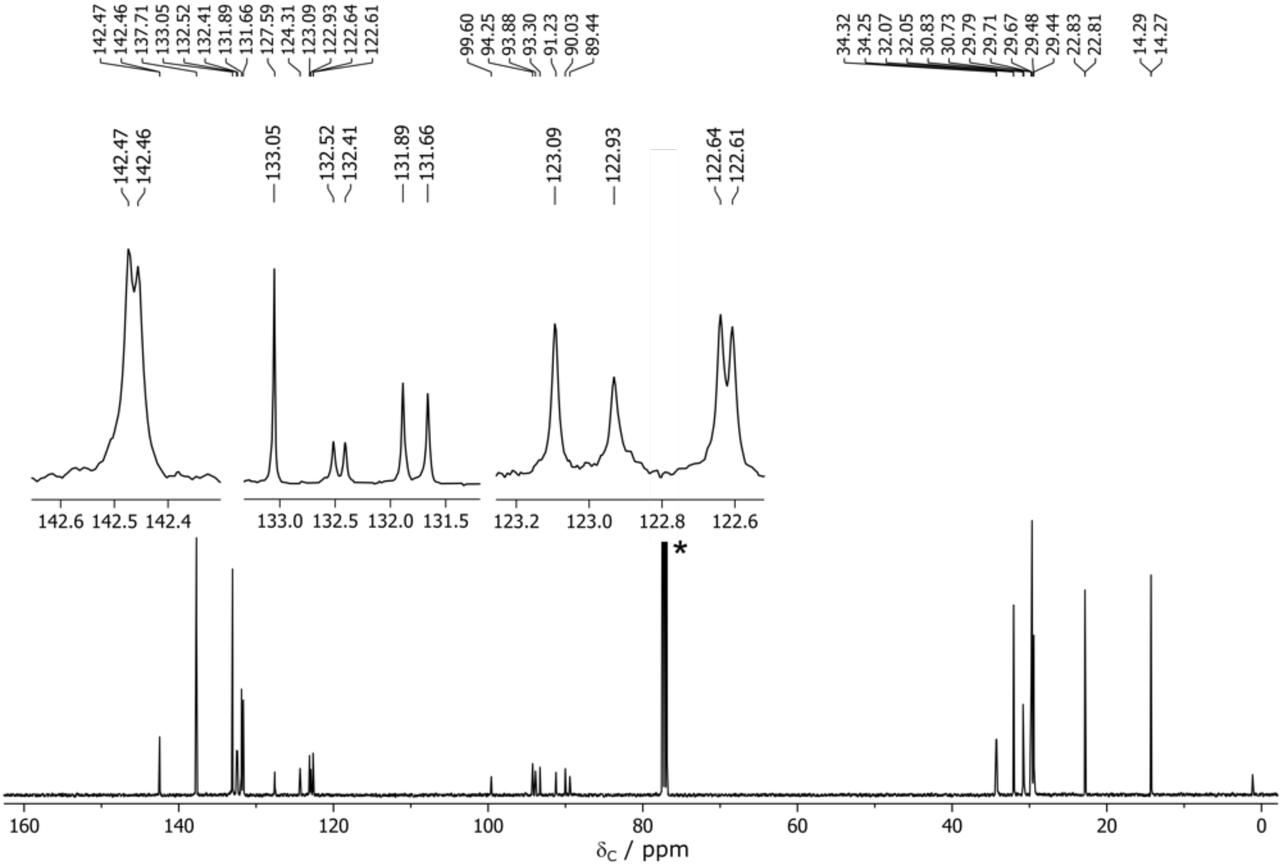

Figure S94. ^{13}C NMR spectrum of **18** (126 MHz, $CDCl_3$, 298 K). * = $CDCl_3$.

Spectra and assignment of ***T18***$_{A}$

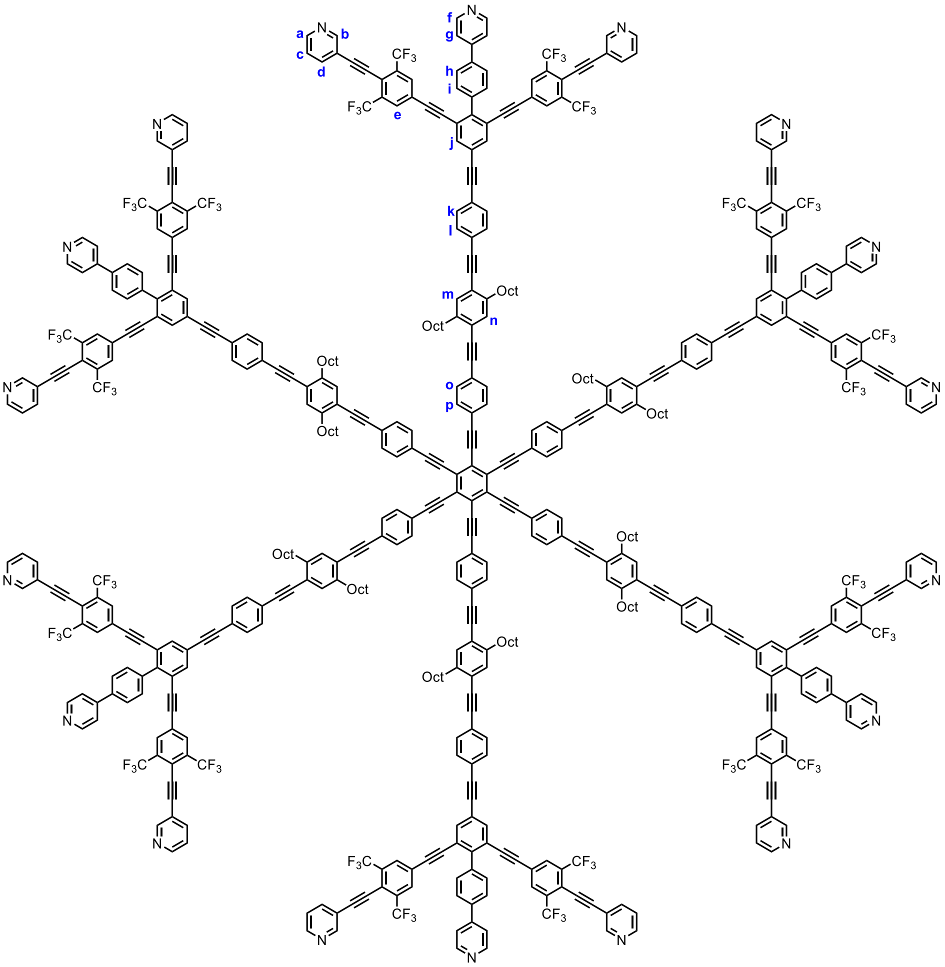

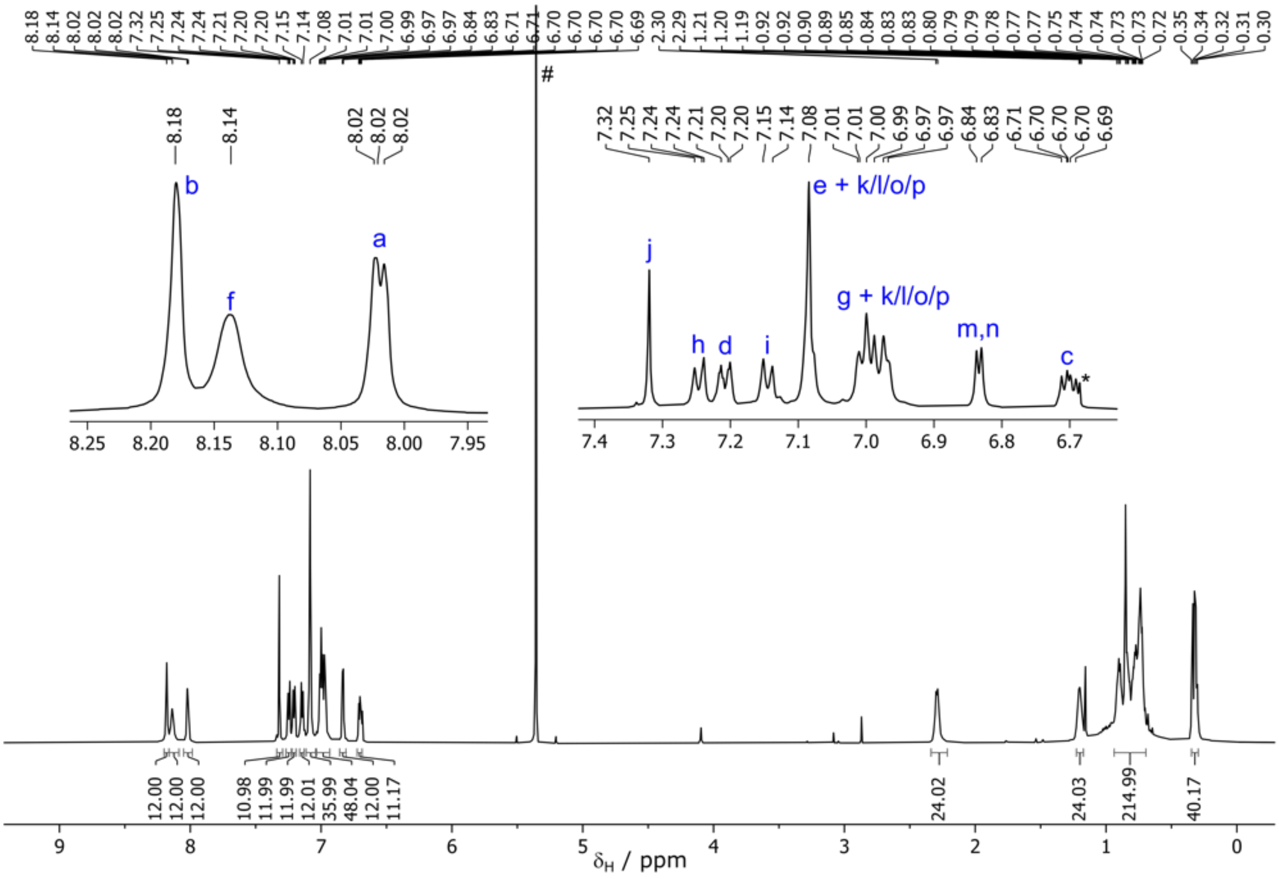

Figure S95. ^{1}H NMR spectrum of **T18$_{A}$** (600 MHz, $C_2D_2Cl_4$, 393 K). * = $CHCl_3$; # = $C_2D_2Cl_4$

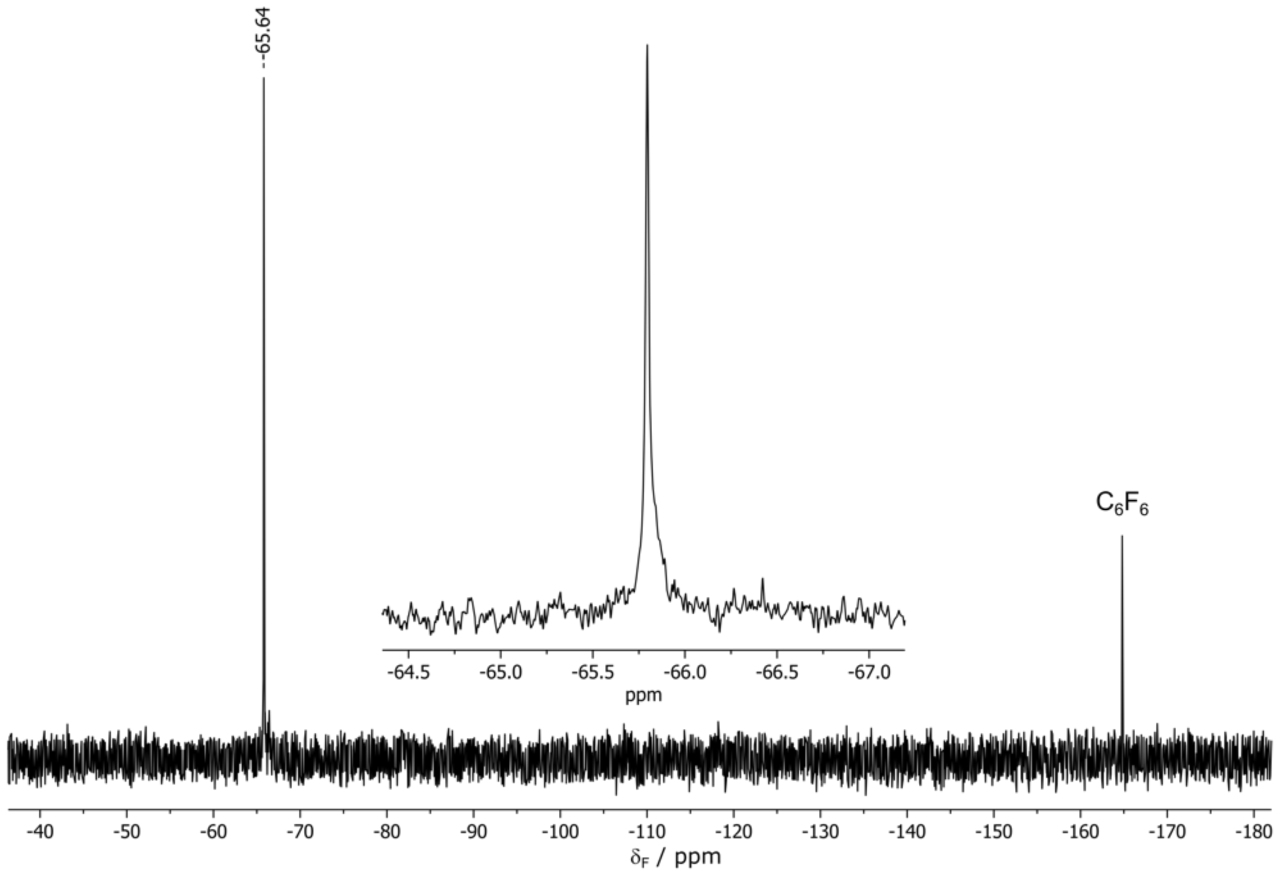

Figure S96. ^{19}F NMR spectrum of **T18**$_{\mathbf{A}}$ (470 MHz, $CDCl_3$, 298 K). Referenced against C_6F_6 (δ_F = −161.64) as an internal standard.

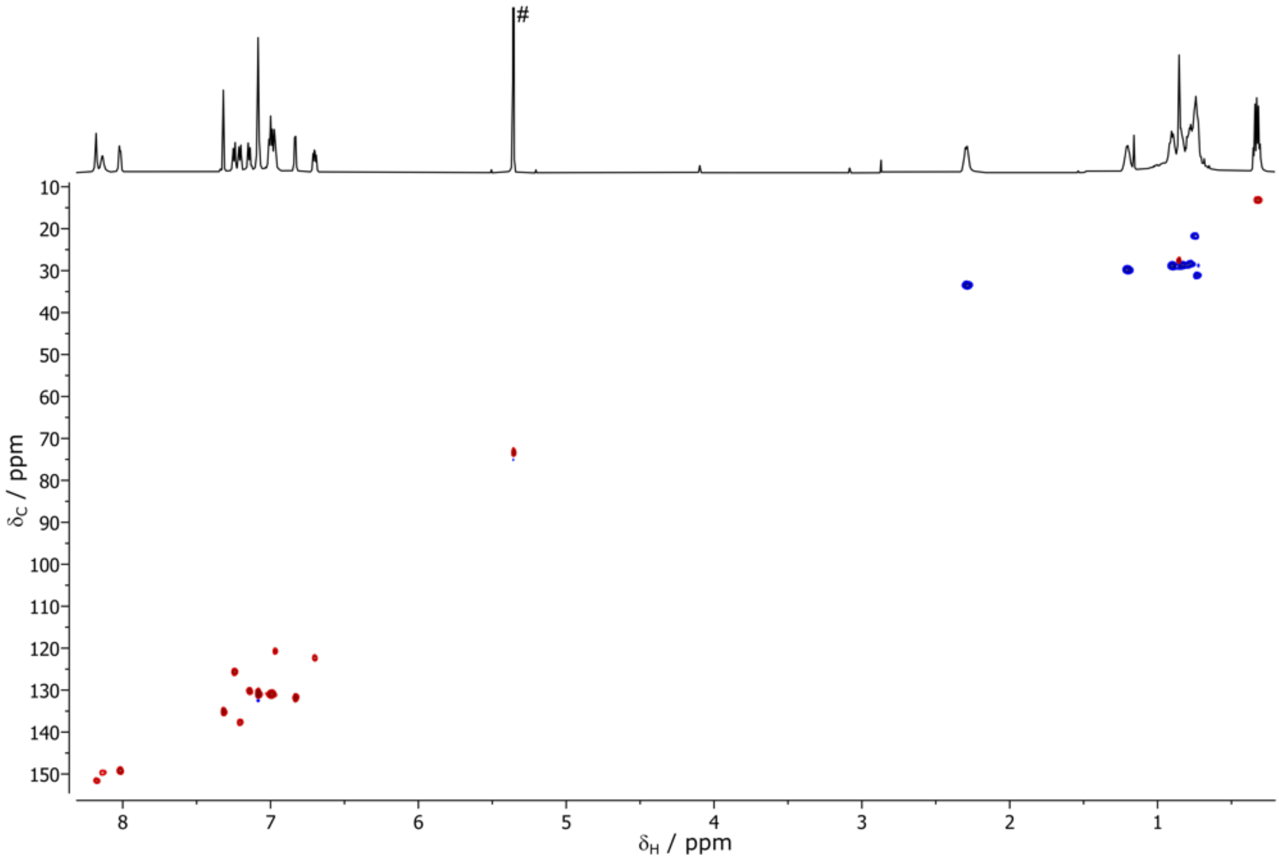

Figure S97. ^{1}H-^{13}C HSQC spectrum of **T18**$_{\mathbf{A}}$ (600 MHz, $C_2D_2Cl_4$, 393 K). # = C_2HDCl_4.

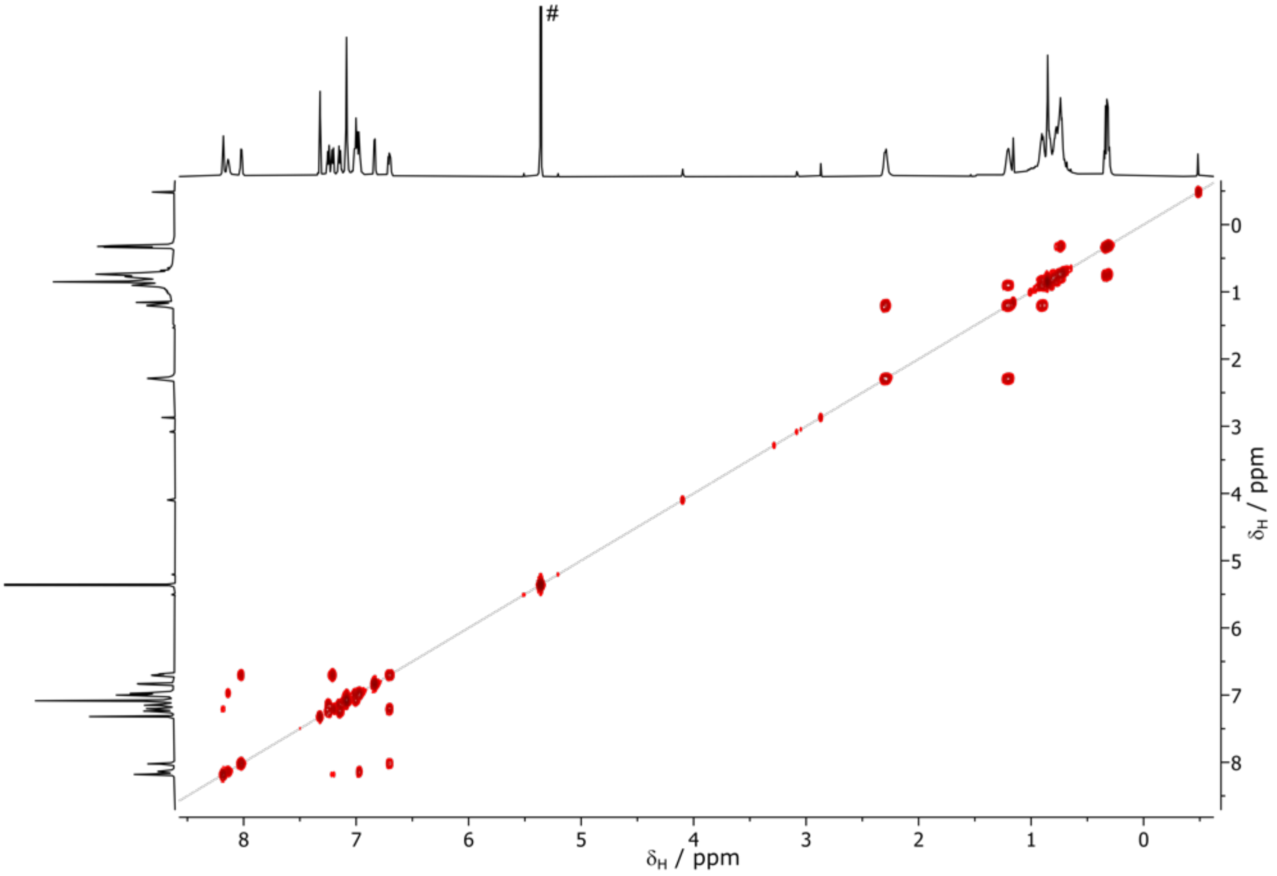

Figure S928. ^1H-^1H COSY spectrum of **T18$_A$** (600 MHz, $C_2D_2Cl_4$, 393 K). # = C_2HDCl_4.

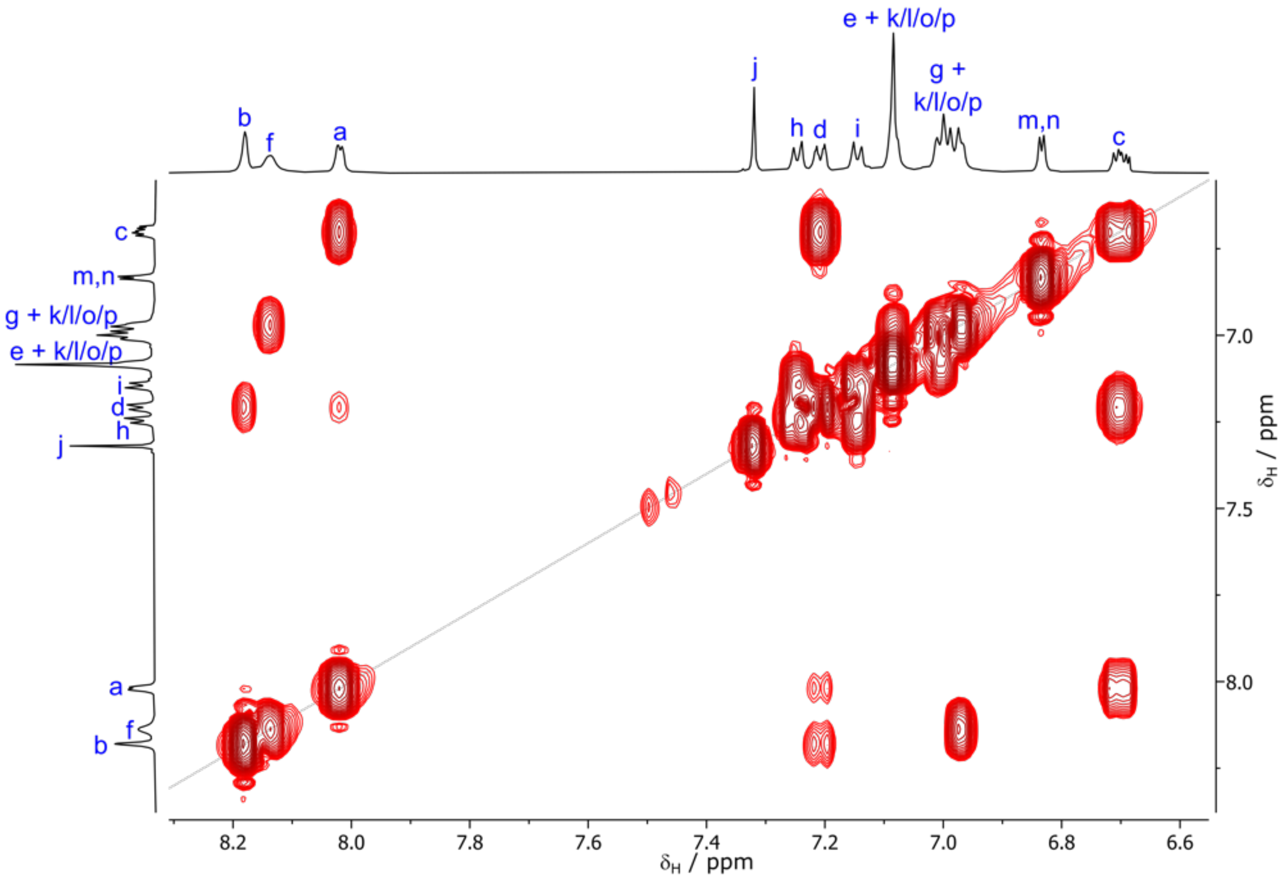

Figure S99. Aromatic region of the ^1H-^1H COSY spectrum of **T18$_A$** (600 MHz, $C_2D_2Cl_4$, 393 K).

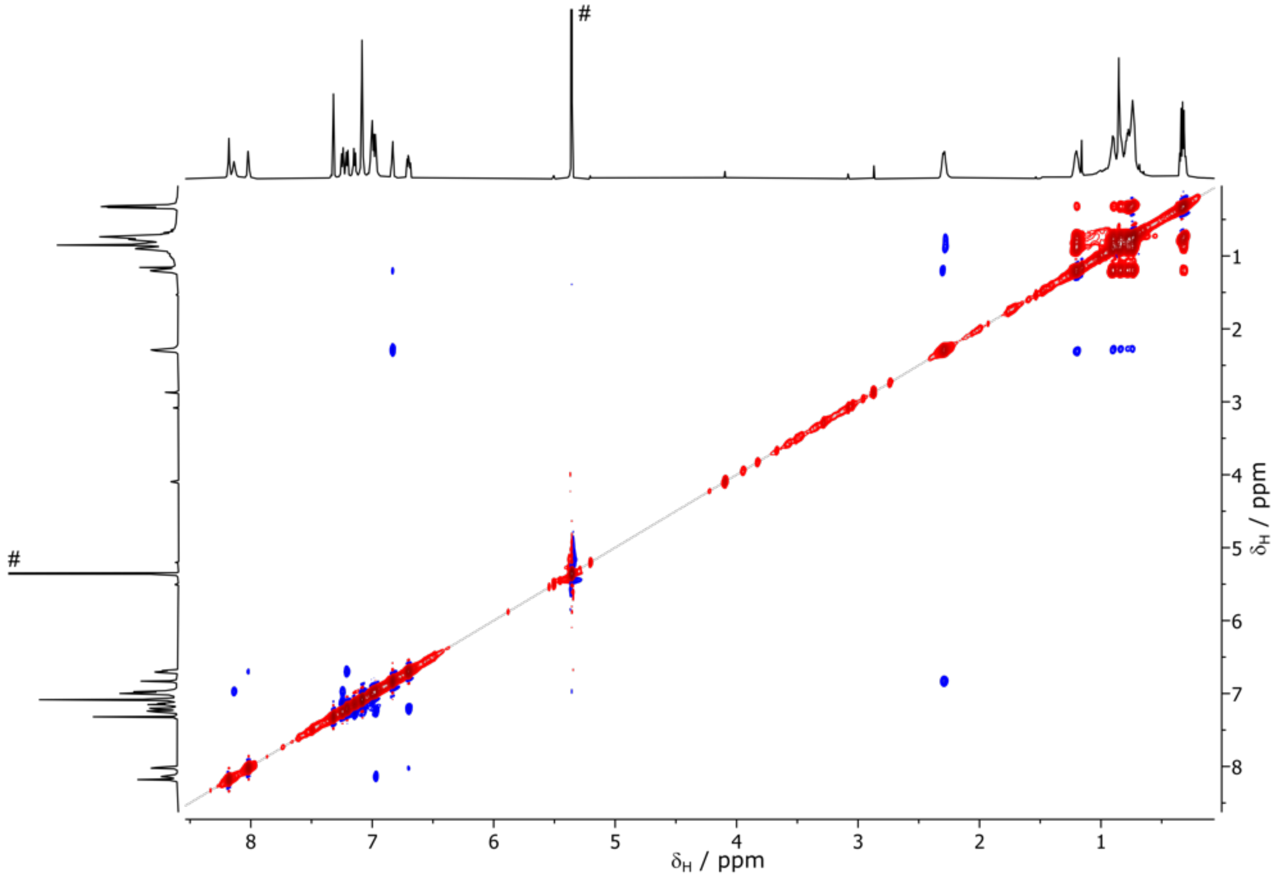

Figure S100. ^{1}H-^{1}H ROESY spectrum of **T18$_A$** (600 MHz, $C_2D_2Cl_4$, 393 K). # = C_2HDCl_4.

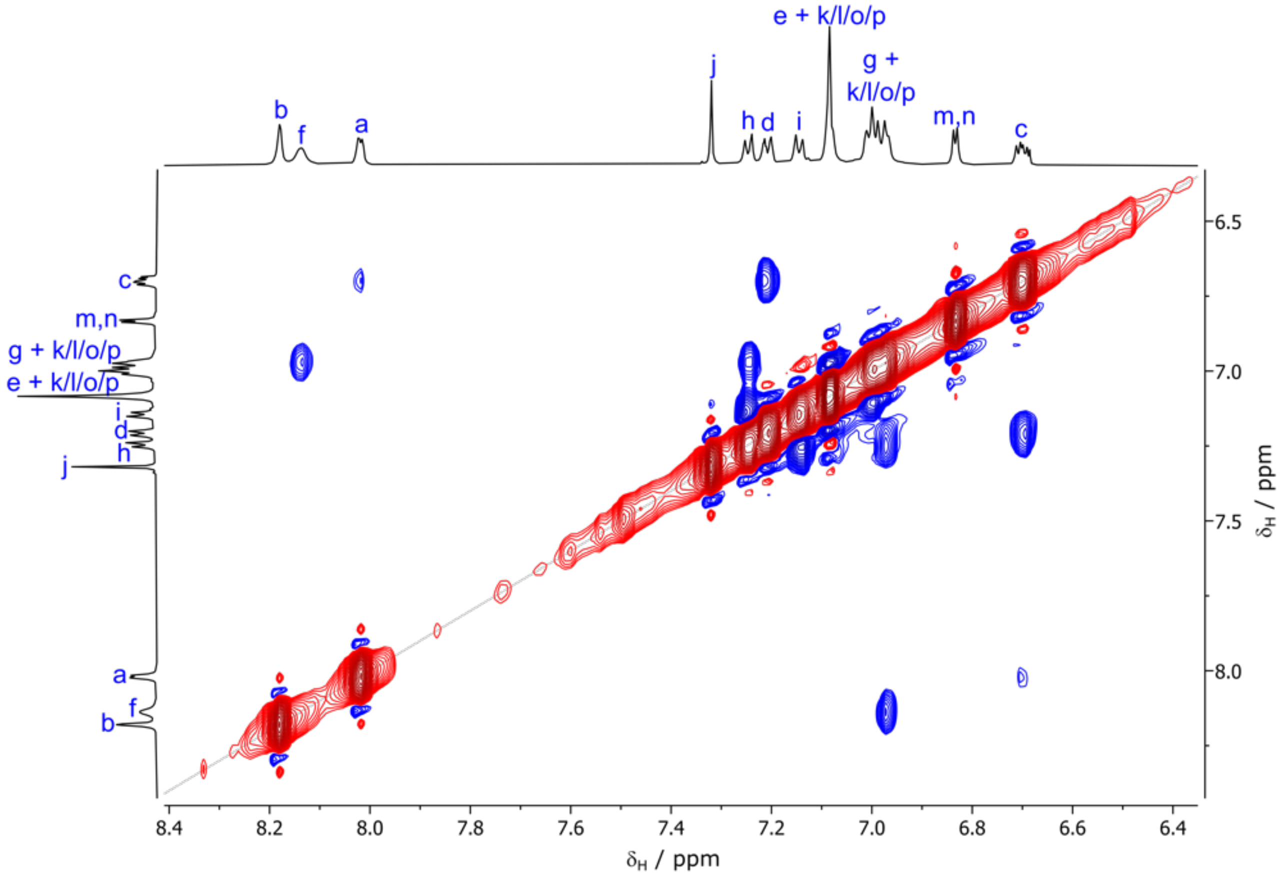

Figure S101. Aromatic region of the ^{1}H-^{1}H ROESY spectrum of **T18$_A$** (600 MHz, $C_2D_2Cl_4$, 393 K).

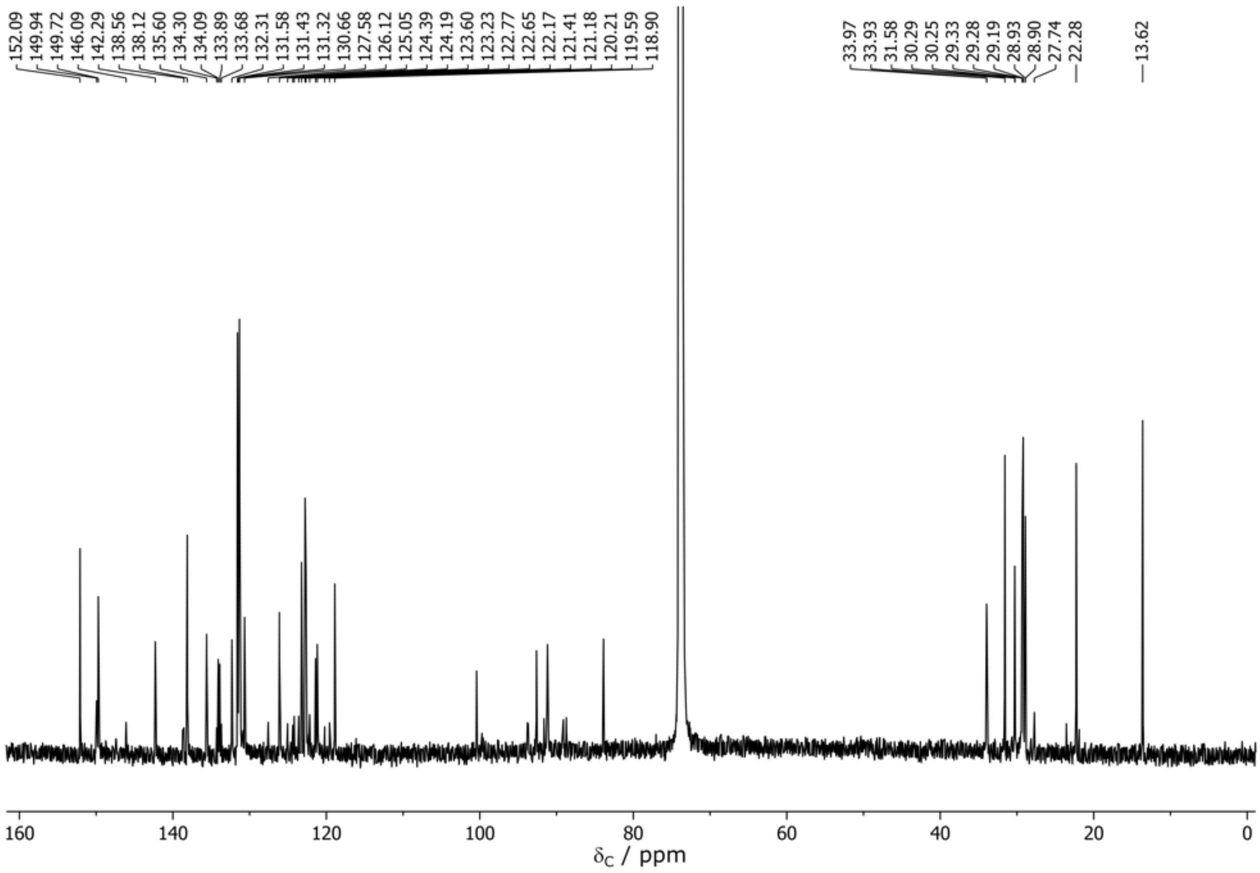

Figure S102. ^{13}C NMR spectrum of **T18$_A$** (151 MHz, $C_2D_2Cl_4$, 393 K). # = $C_2D_2Cl_4$.

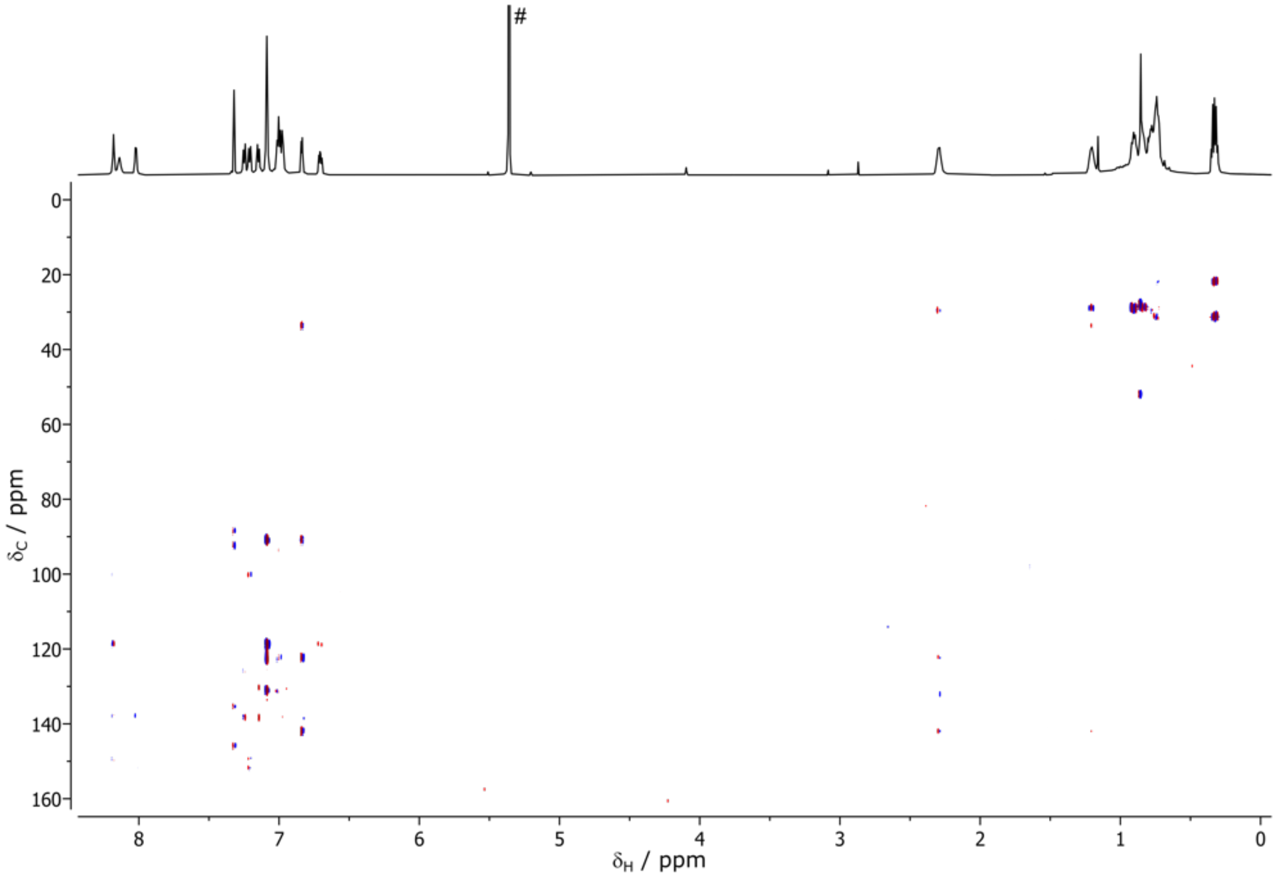

Figure S103. ^{1}H-^{13}C HMBC spectrum of **T18$_A$** (600 MHz, $C_2D_2Cl_4$, 393 K). # = C_2HDCl_4.

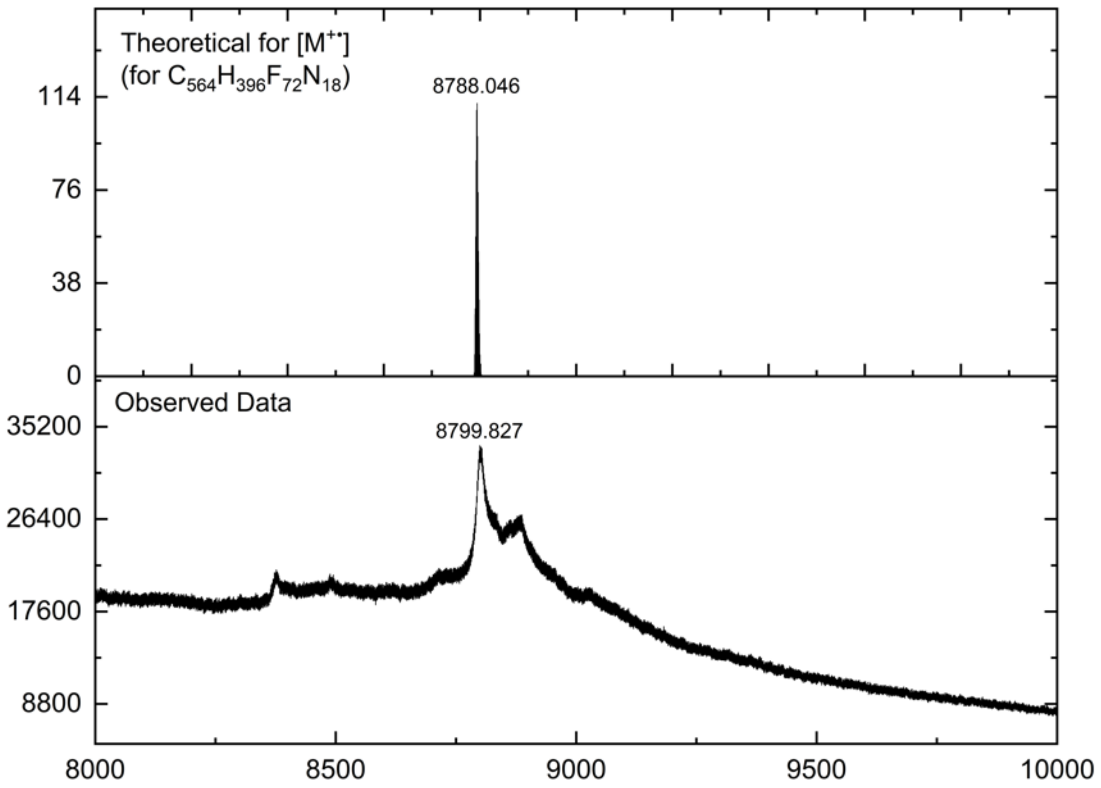

Figure S104. MALDI mass spectrum of **T18**$_{\mathbf{A}}$ (matrix: dithranol)

	a	b	c	d	e	f	g	h	i	j	k	l	m	n	o	p	Ar-CH_2
a			s	w													
b				m													
c	w			s													
d			s														
e																	
f							s										
g						m											
h							m		m								
i																	
j																	
k																	
l																	
m																	
n																	
o																	
p																	
Ar-CH_2													s	s			

Table S2. Summary of COSY (above diagonal) and ROESY (below diagonal) correlations in **T18**$_{\mathbf{A}}$. vw; very weak; w: weak; mw: medium weak; m: medium; s: strong

Explanation of Assignment

- ^{1}H NMR peaks at 8.00–8.20 ppm are protons α to nitrogen in the pyridines, **a**, **b** and **f**.
- Pattern of COSY cross peaks for **a**, **b**, **c**, and **d** match those in compound **S4**, so can assign all as: **a, b, c, d**
- Can now assign **f** as other proton α to a nitrogen.
- **e** and **j** are the only singlets, so can be assigned. (Similar chemical shifts as in **T3**$_{\mathbf{A}}$ and integration makes sense, assuming there is another peak of integral 12 hidden under **e**, also HOESY of CF_3 to **e**.)
- **f** has a COSY correlation only to **g**
- **g** has a ROSEY correlation to **f** and other peak = **h**
- **h** has a correlation COSY to **i**
- ^{1}H NMR peak at 6.84 has strong ROESY to first and second CH_2 in octyl chain, therefore contains protons **m + n** (ROESY to first CH_2 stronger than to second CH_2).
- Cannot definitively assign k, l, o, or p.

Compound 9

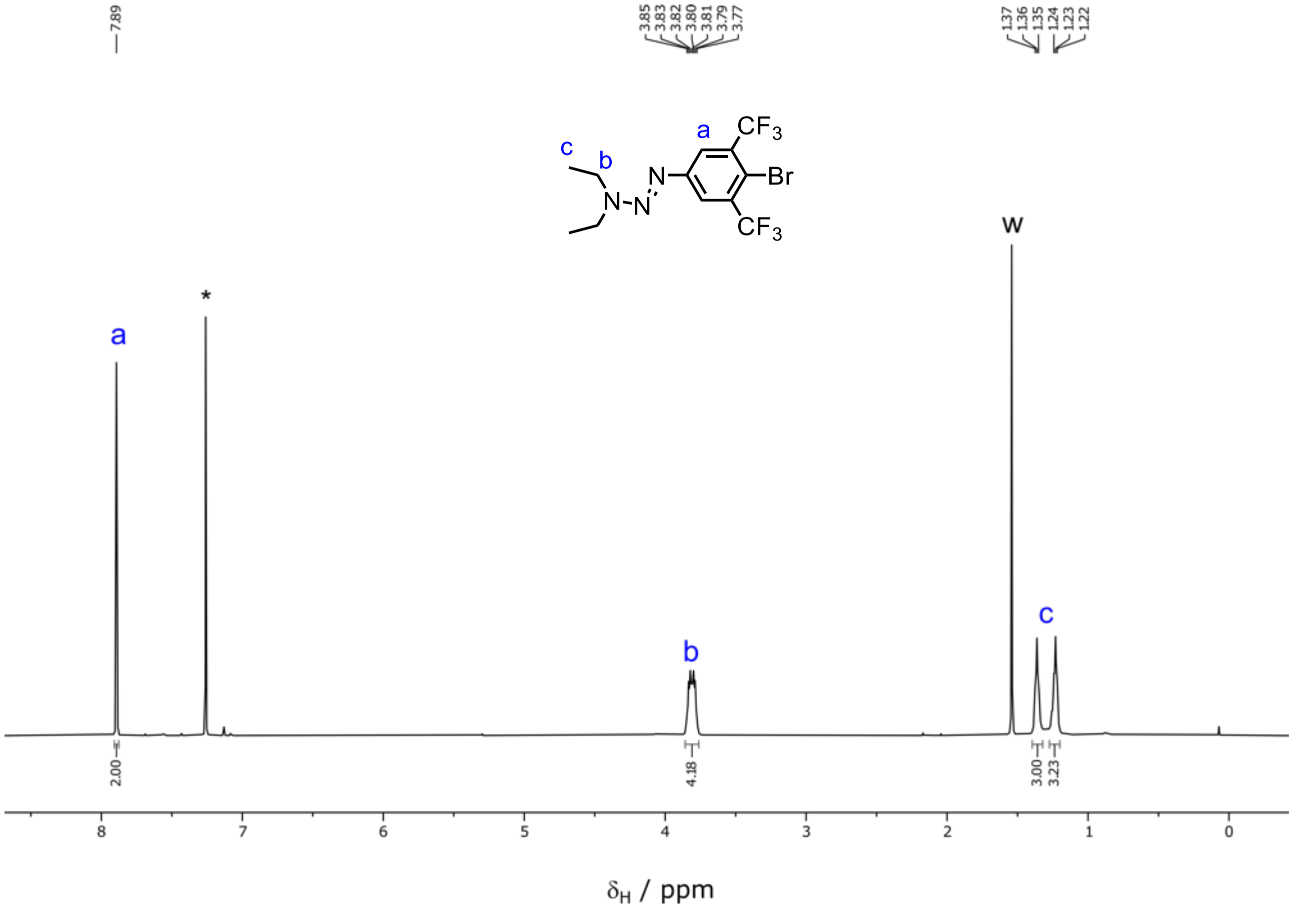

Figure S105. ^{1}H NMR spectrum of **9** (600 MHz, $CDCl_3$, 298 K). * = $CHCl_3$.

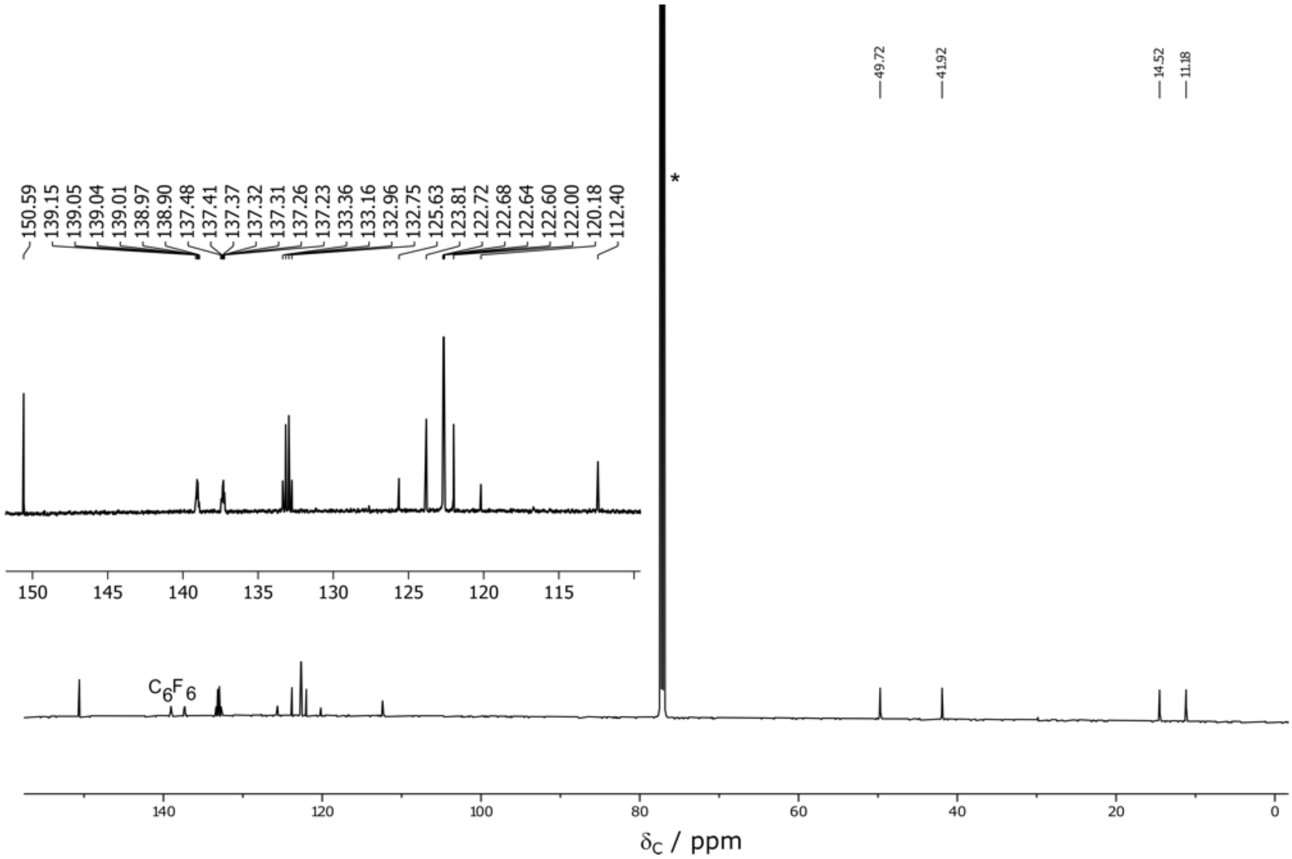

Figure S106. ^{13}C NMR spectrum of **9** (151 MHz, $CDCl_3$, 298 K). * = $CDCl_3$.

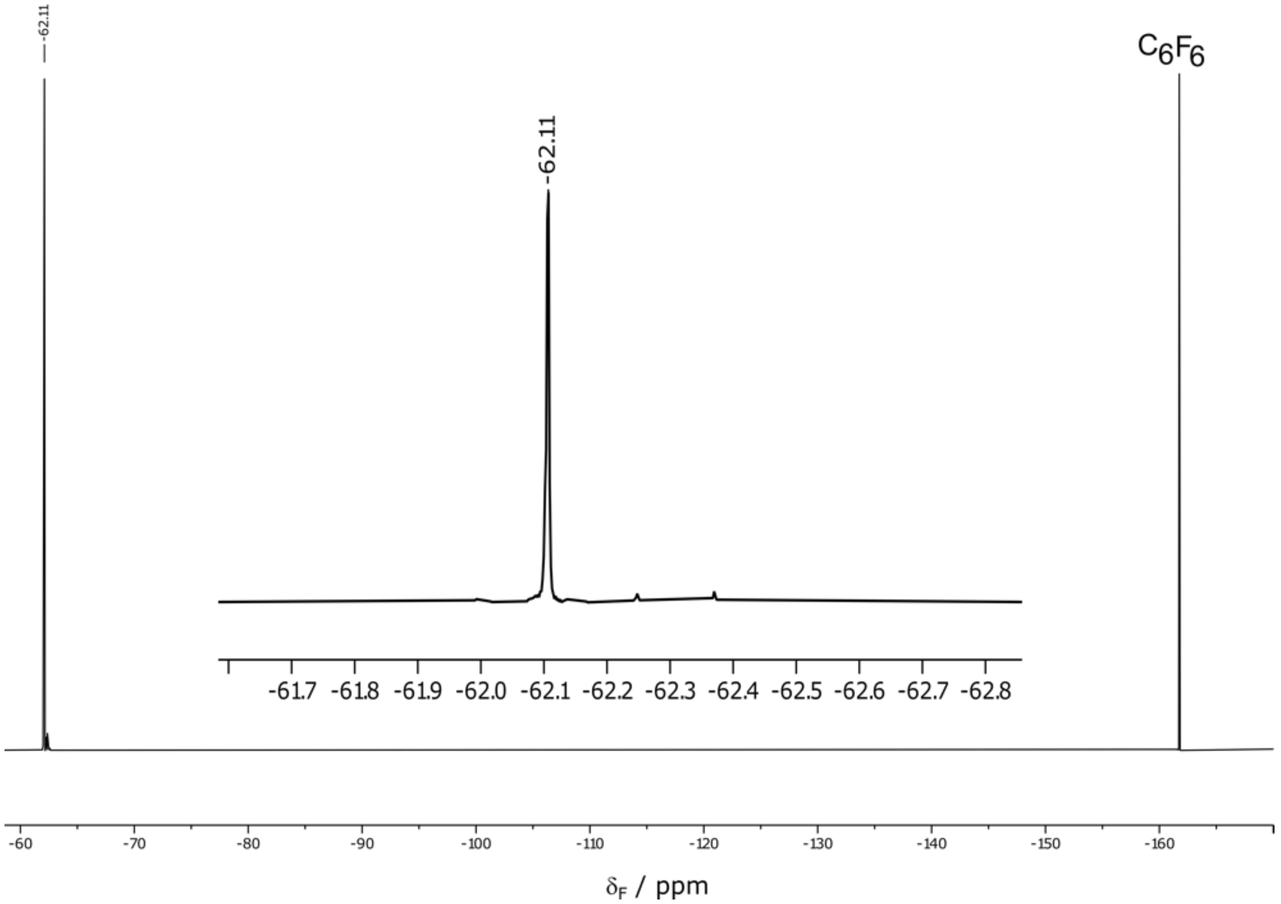

Figure S107. ^{19}F NMR spectrum of **9** (564 MHz, $CDCl_3$, 298 K). Referenced to C_6F_6 (δ_F = −161.64 ppm).

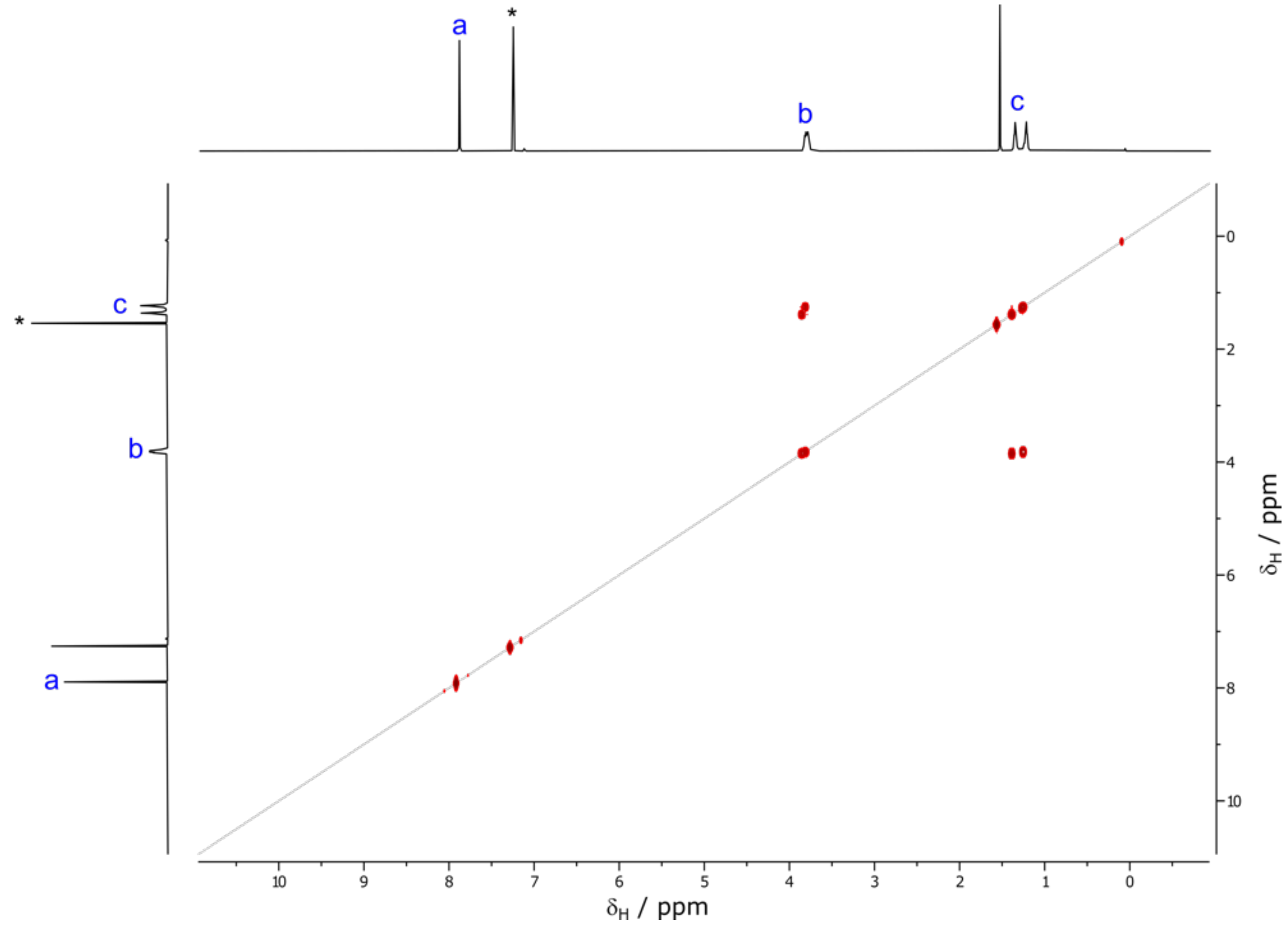

Figure S108. ^{1}H-^{1}H COSY spectrum of **9** (600 MHz, $CDCl_3$, 298 K). * = $CHCl_3$.

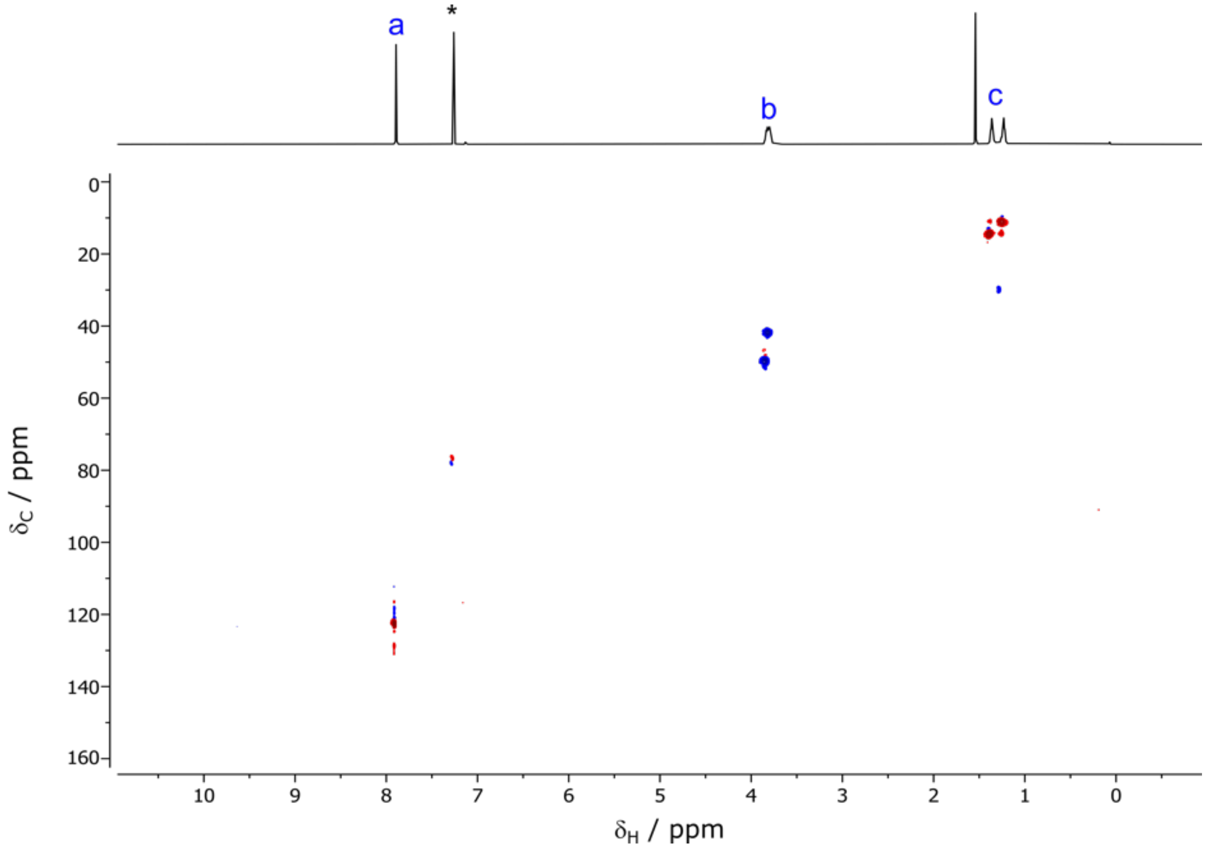

Figure S109. ^{1}H-^{13}C HSQC spectrum of **9** (600 MHz, $CDCl_3$, 298 K). * = $CHCl_3$.

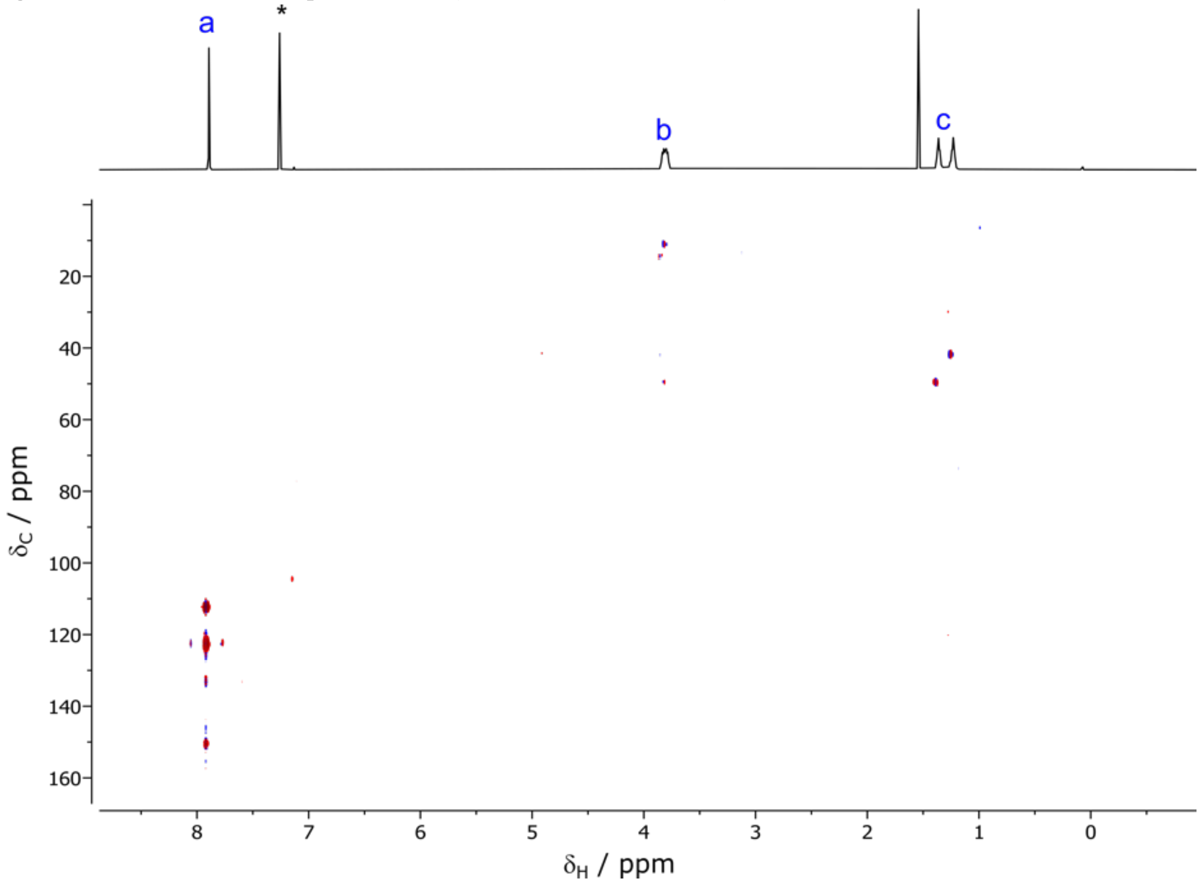

Figure S110. ^{1}H-^{13}C HMBC spectrum of **9** (600 MHz, $CDCl_3$, 298 K). * = $CHCl_3$.

Expanded Spectrum RT 0.22, NL 16655342, Peak [1], Target Mass 392.0192

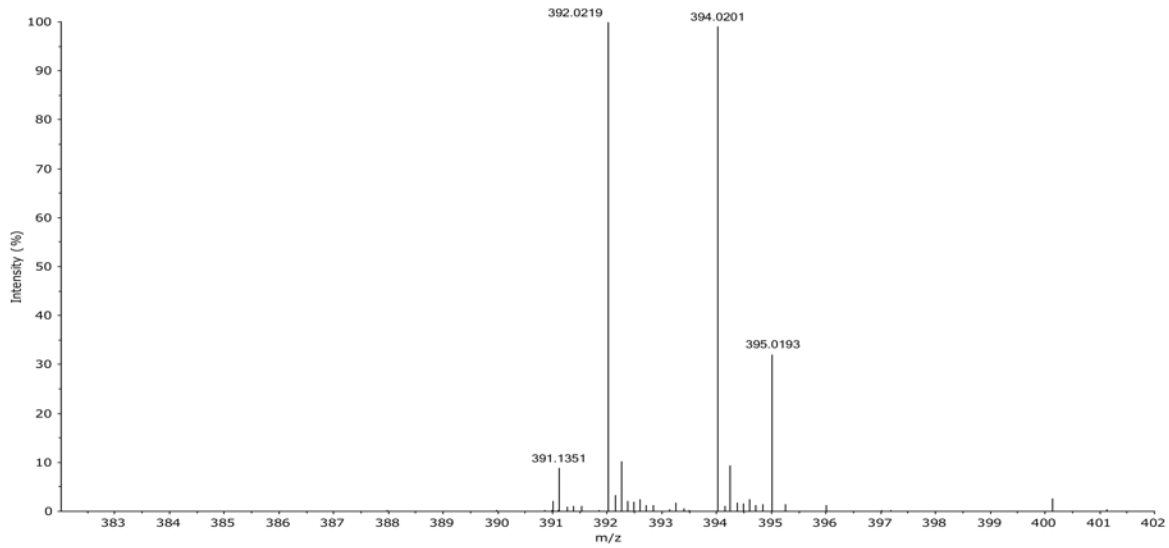

Theoretical Spectrum for C12H13BrF6N3, Minimum Abundance 0.01%

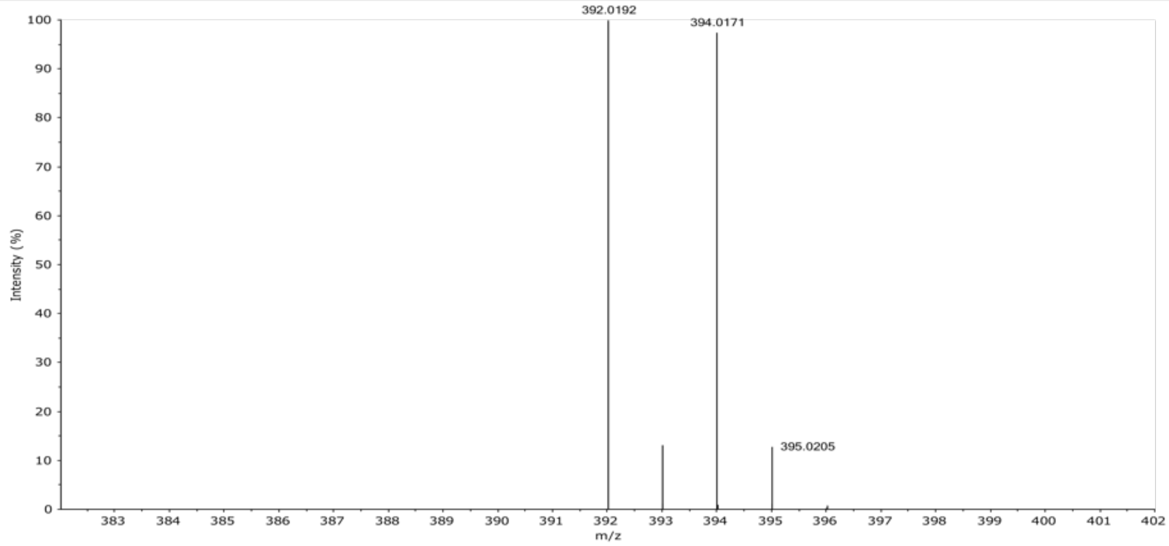

Measured Mass	Calculated Mass	Error (mDa)	Error (ppm)	Formula $[M+H]^+$	Response
392.0187	392.0192	-0.46	-1.16	$C_{12}H_{13}BrF_6N_3$	248903961

Figure S111. High-resolution ESI+ mass spectrum of **9**.

Compound 10

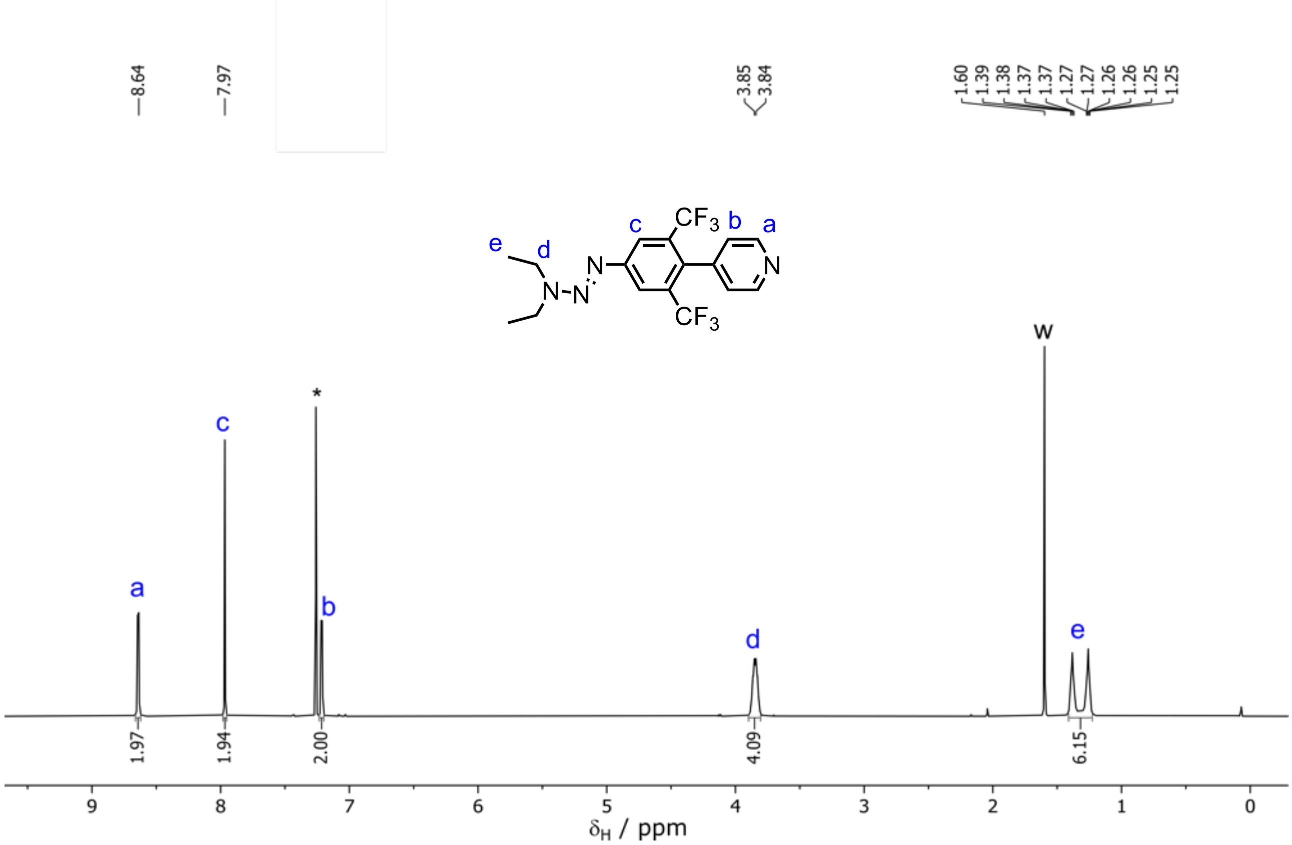

Figure S112. ^{1}H NMR spectrum of **10** (600 MHz, $CDCl_3$, 298 K). * = $CHCl_3$.

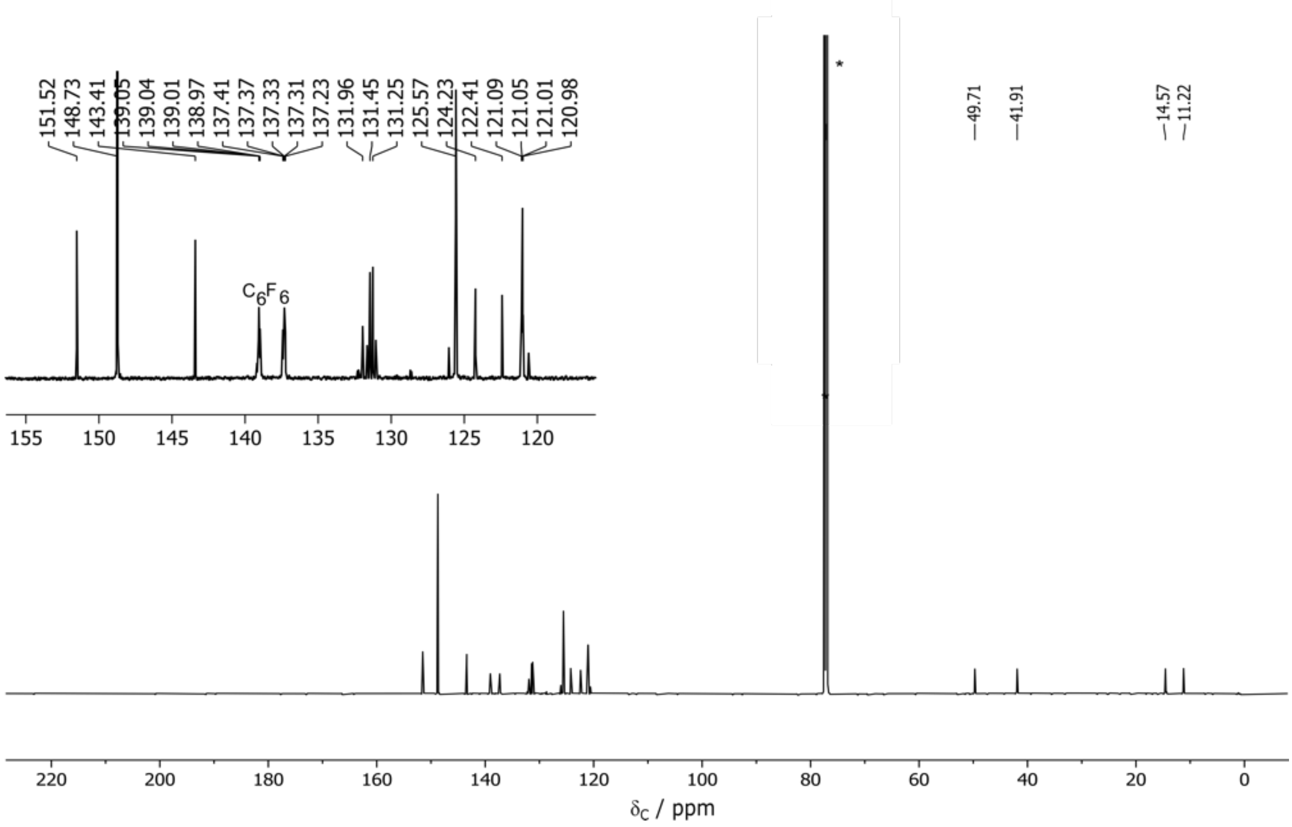

Figure S113. ^{13}C NMR spectrum of **10** (151 MHz, $CDCl_3$, 298 K). * = $CDCl_3$.

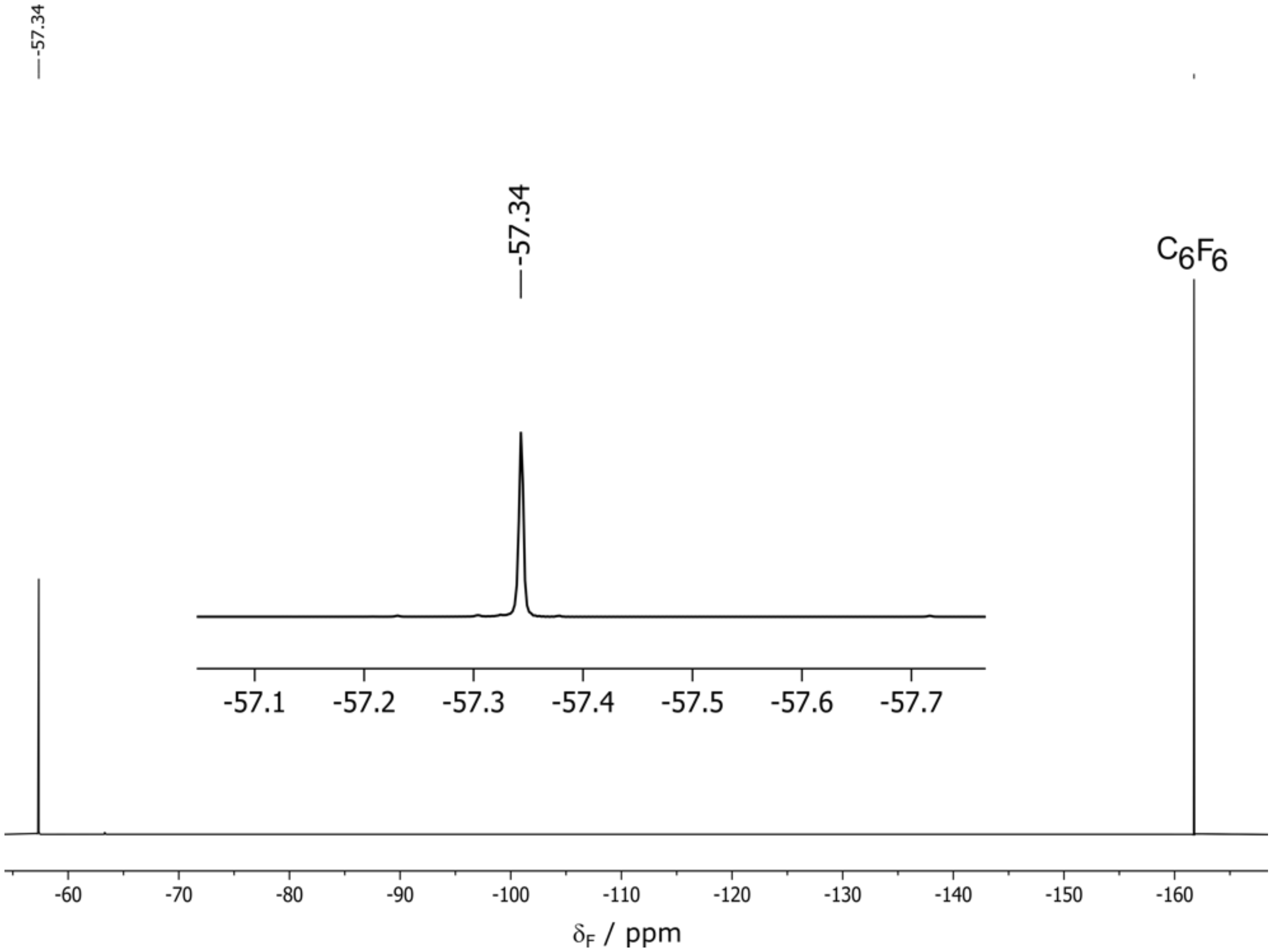

Figure S114. ^{19}F NMR spectrum of **10** (564 MHz, $CDCl_3$, 298 K). Referenced to C_6F_6 (δ_F = −161.64 ppm).

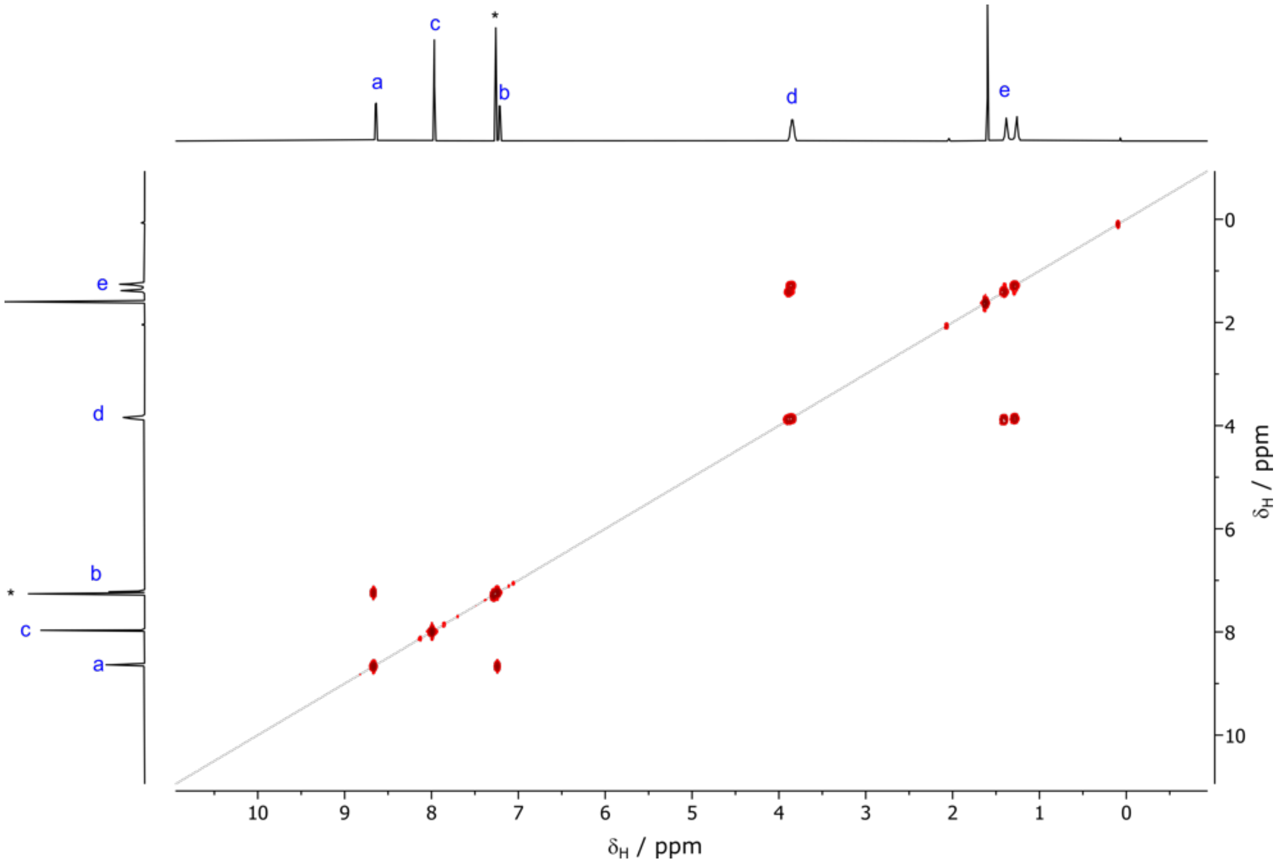

Figure S115. ^{1}H-^{1}H COSY spectrum of **10** (600 MHz, $CDCl_3$, 298 K). * = $CHCl_3$.

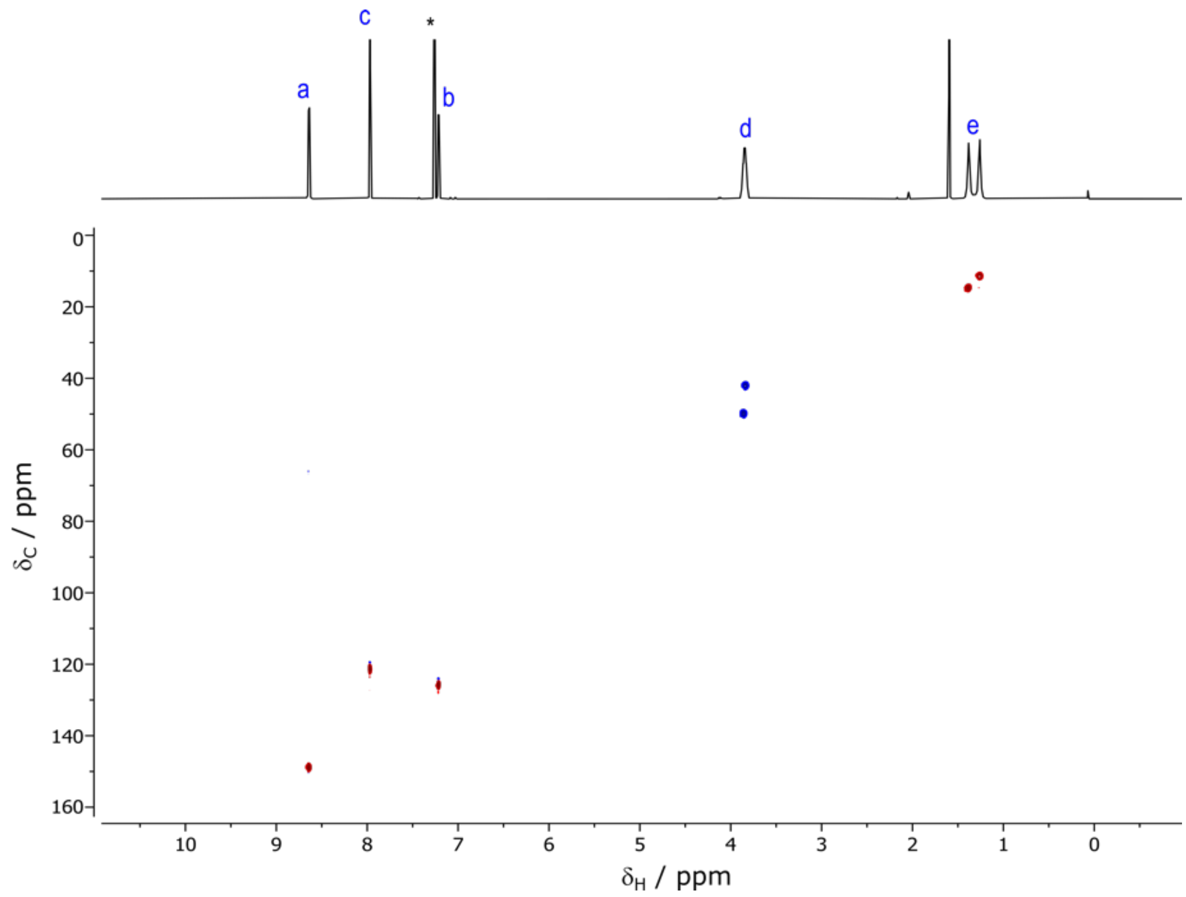

Figure S116. ^{1}H-^{13}C HSQC spectrum of **10** (600 MHz, $CDCl_3$, 298 K). * = $CHCl_3$.

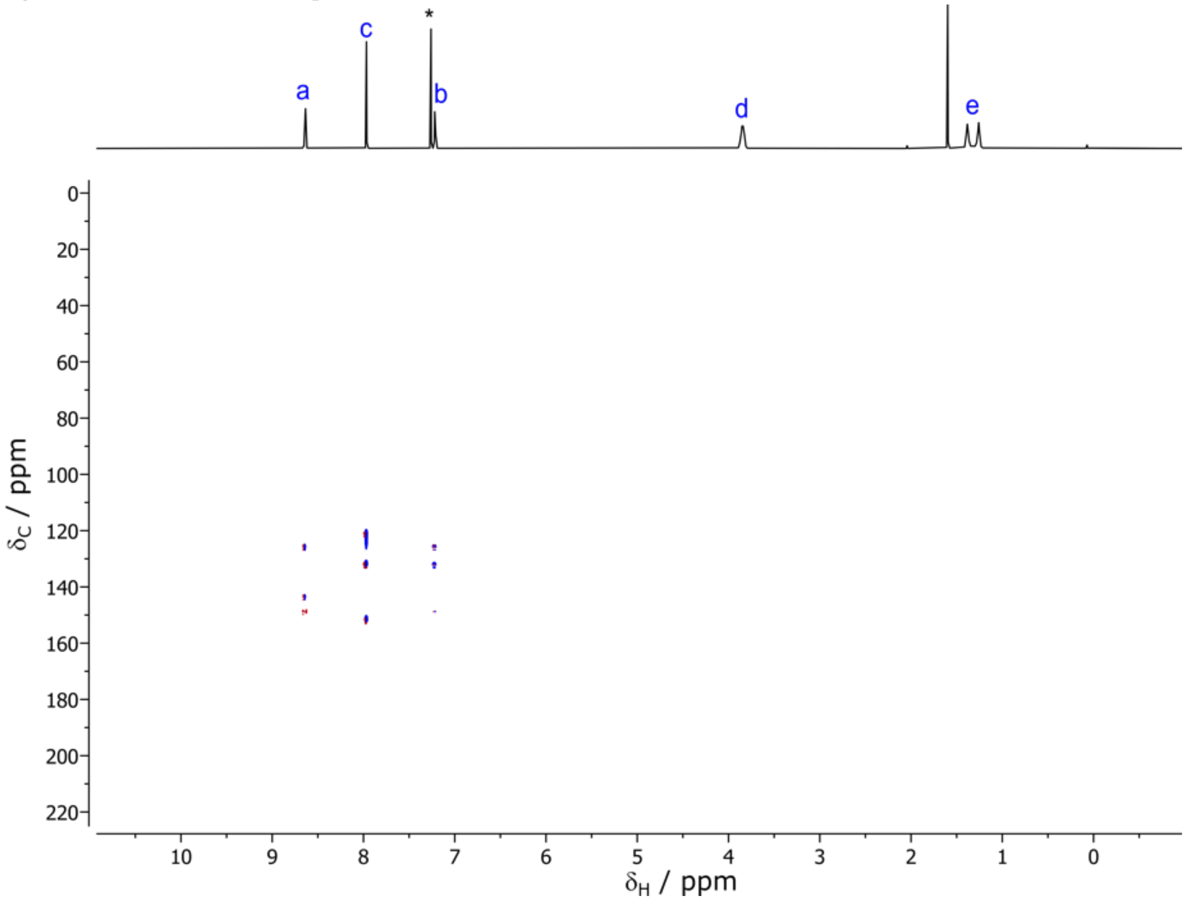

Figure S117. ^{1}H-^{13}C HMBC spectrum of **10** (600 MHz, $CDCl_3$, 298 K). * = $CHCl_3$.

Expanded Spectrum RT 0.18, NL 280871, Peak [1], Target Mass 388.0951

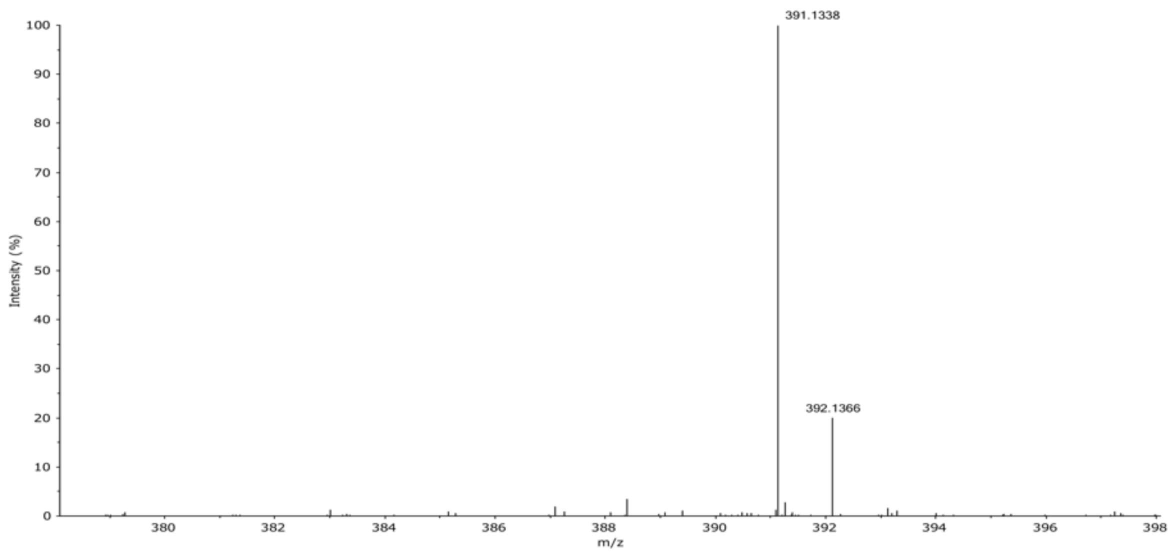

Theoretical Spectrum for C18H16F6NSi, Minimum Abundance 0.01%

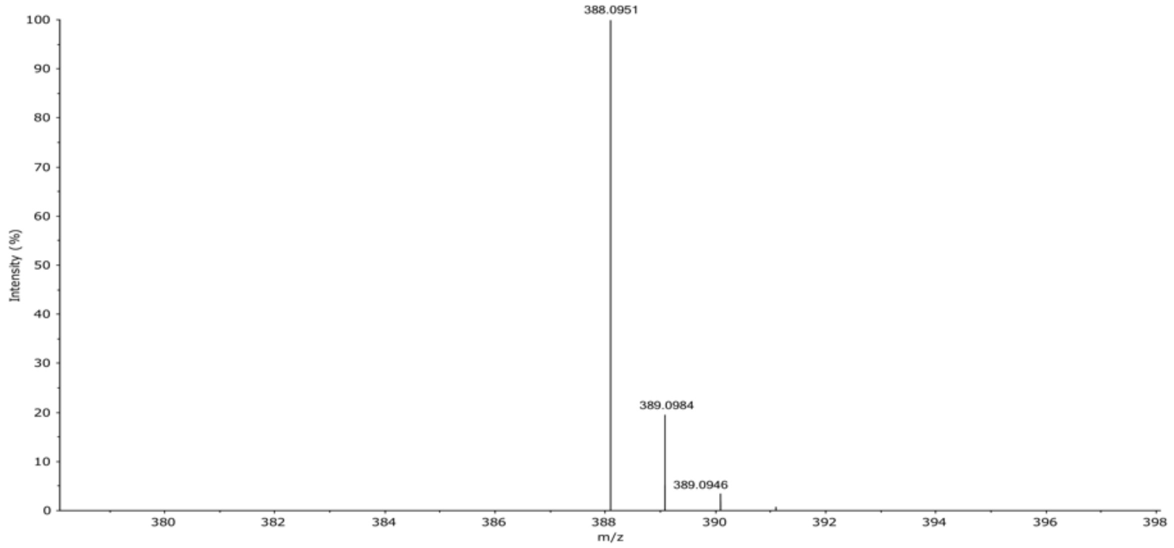

Measured Mass	Calculated Mass	Error (mDa)	Error (ppm)	Formula $[M+H]^+$	Response
388.3949	388.0951	299.82	772.55	$C_{18}H_{16}F_6NSi$	10488

Figure S118. High-resolution ESI+ mass spectrum of **10**.

Compound 11

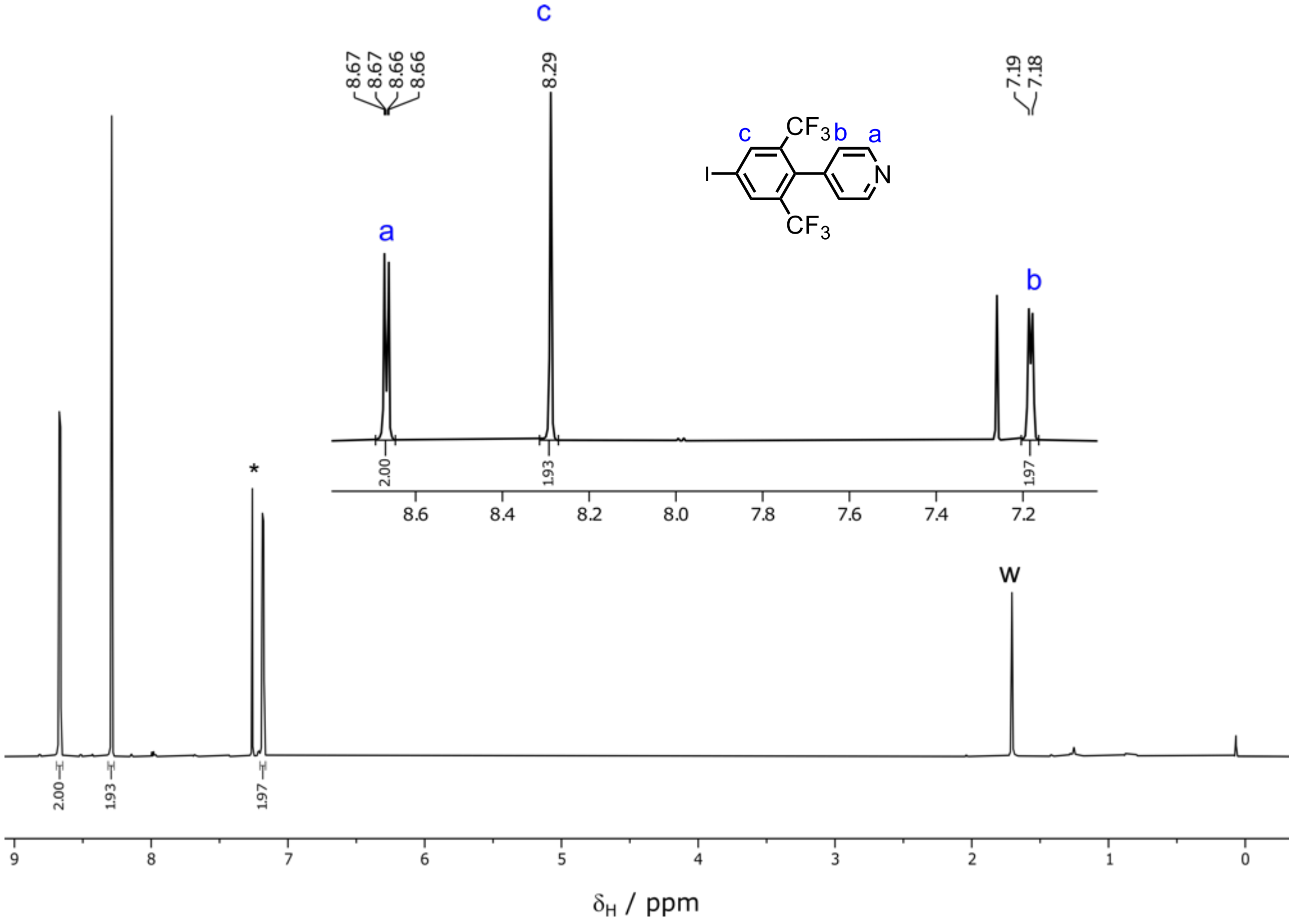

Figure S119. ^{1}H NMR spectrum of **11** (600 MHz, $CDCl_3$, 298 K). * = $CHCl_3$.

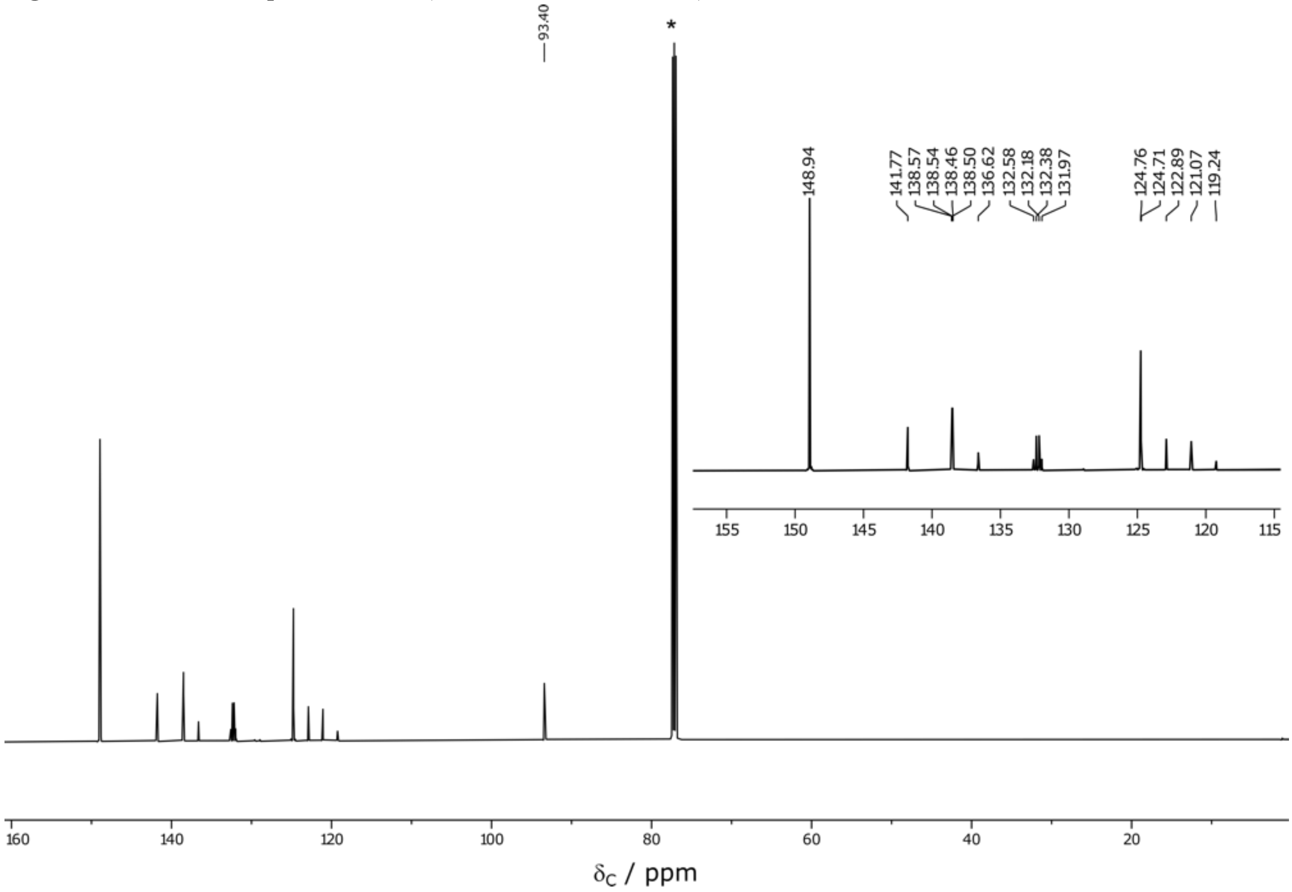

Figure S120. ^{13}C NMR spectrum of **11** (151 MHz, $CDCl_3$, 298 K). * = $CDCl_3$.

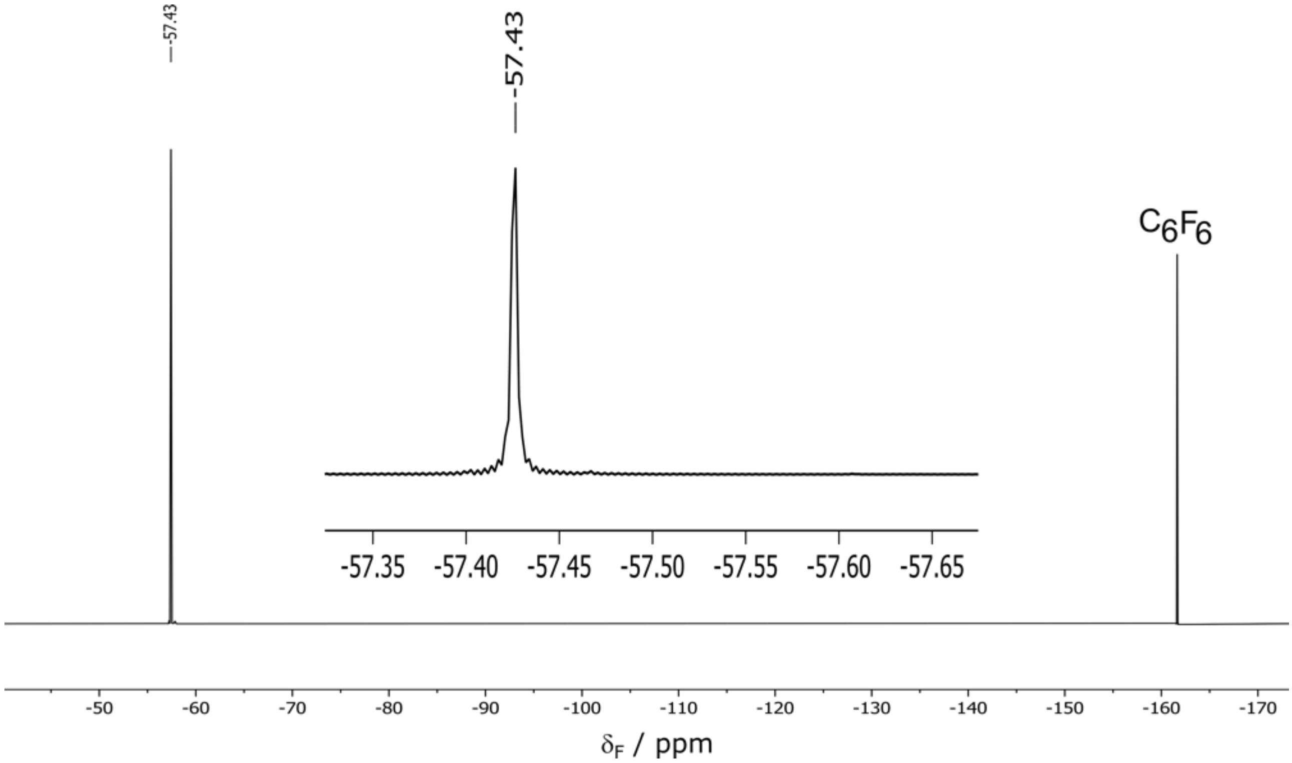

Figure S121. ^{19}F NMR spectrum of **11** (470 MHz, $CDCl_3$, 298 K). Referenced to C_6F_6 (δ_F = –161.64 ppm).

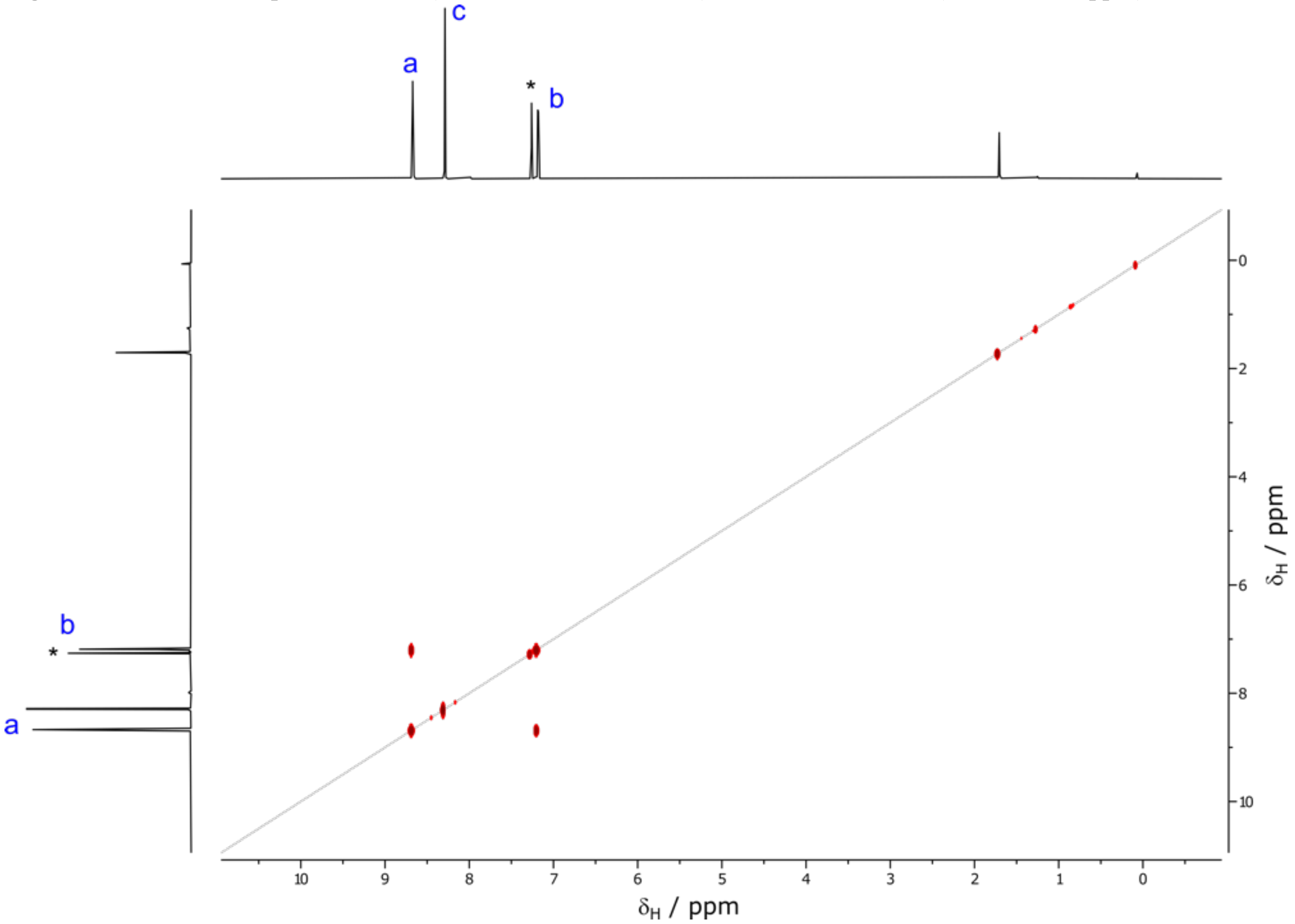

Figure S122. ^1H-^1H COSY spectrum of **11** (600 MHz, $CDCl_3$, 298 K). * = $CHCl_3$.

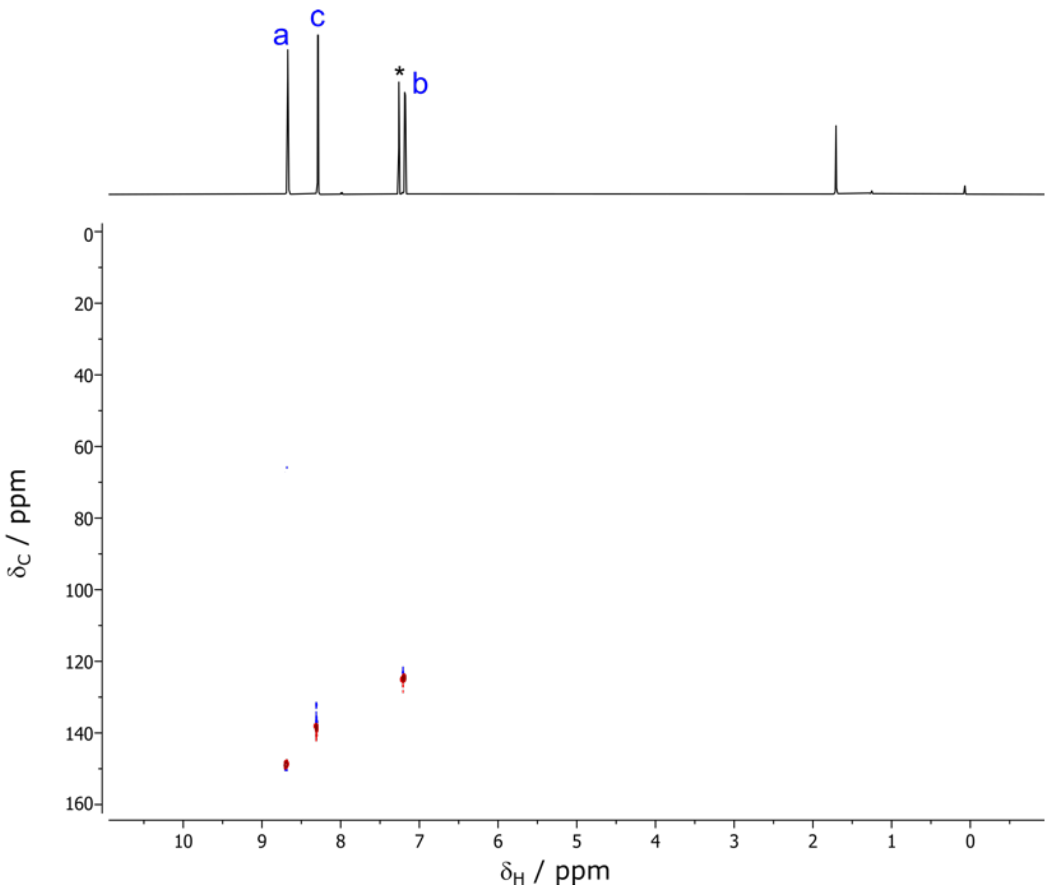

Figure S123. ^{1}H-^{13}C HSQC spectrum of **11** (600 MHz, $CDCl_3$, 298 K). * = $CHCl_3$.

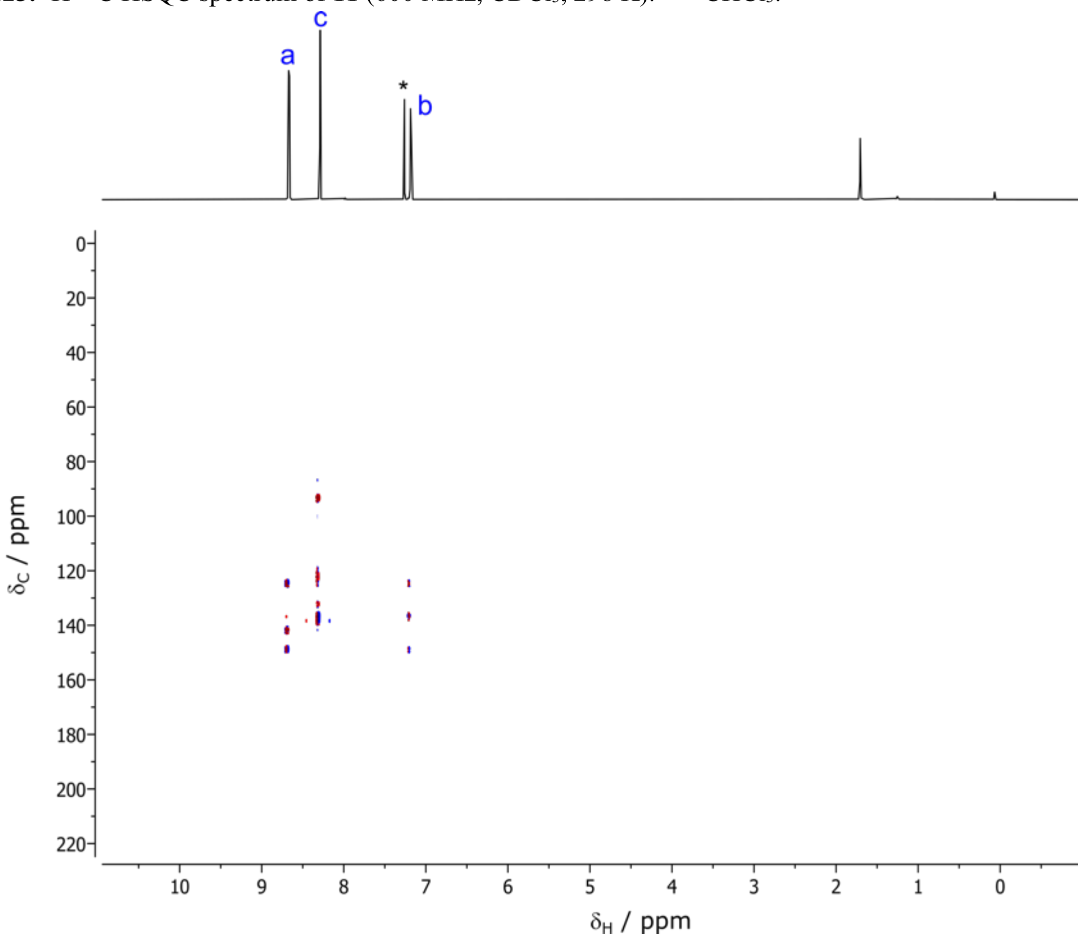

Figure S124. ^{1}H-^{13}C HMBC spectrum of **11** (600 MHz, $CDCl_3$, 298 K). * = $CHCl_3$.

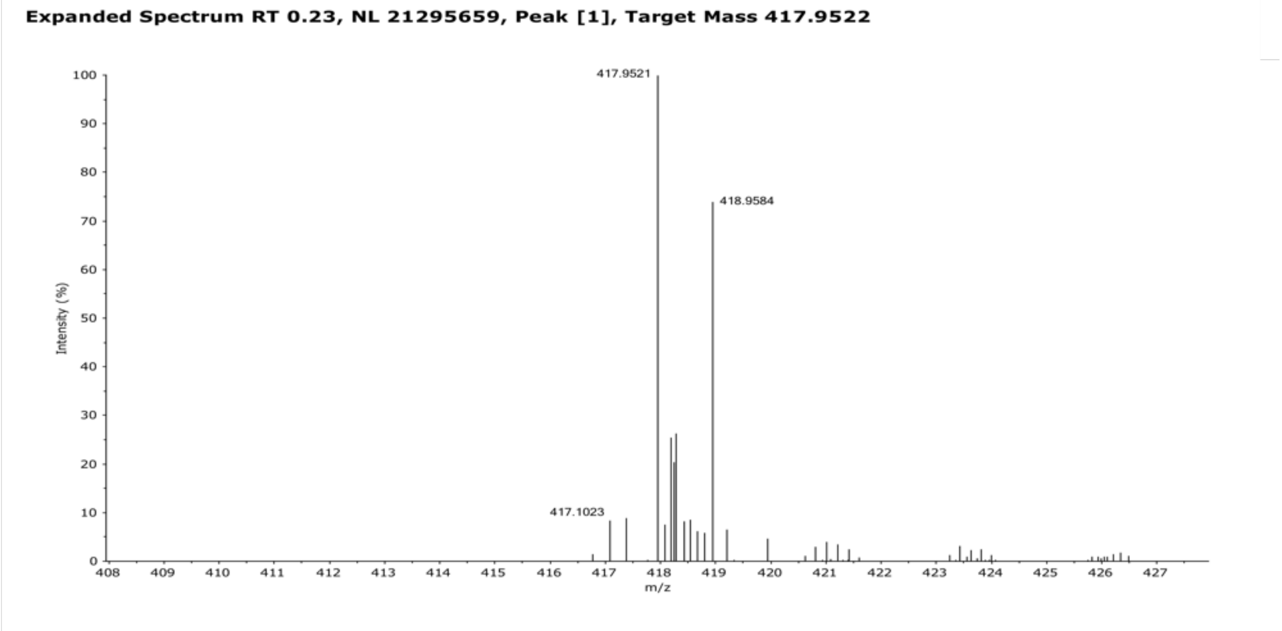

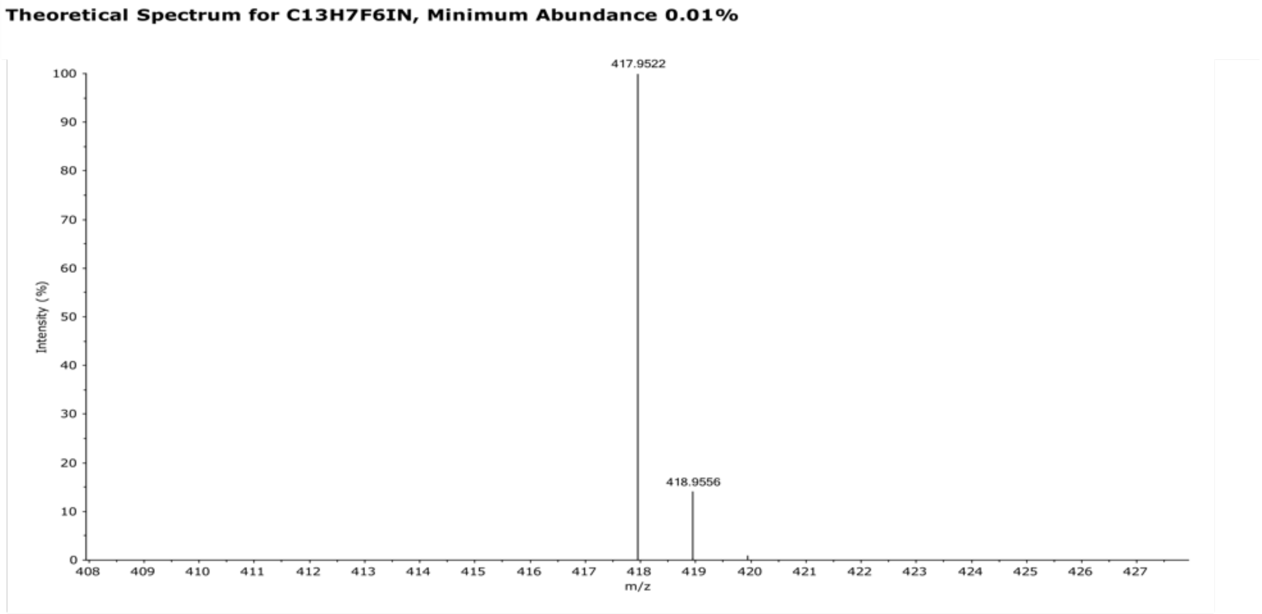

Measured Mass	Calculated Mass	Error (mDa)	Error (ppm)	Formula $[M+H]^+$	Response
417.9506	417.9522	-1.60	-3.83	$C_{13}H_7F_6IN$	334862844

Figure S125. High-resolution ESI+ mass spectrum of **11**.

Compound 12

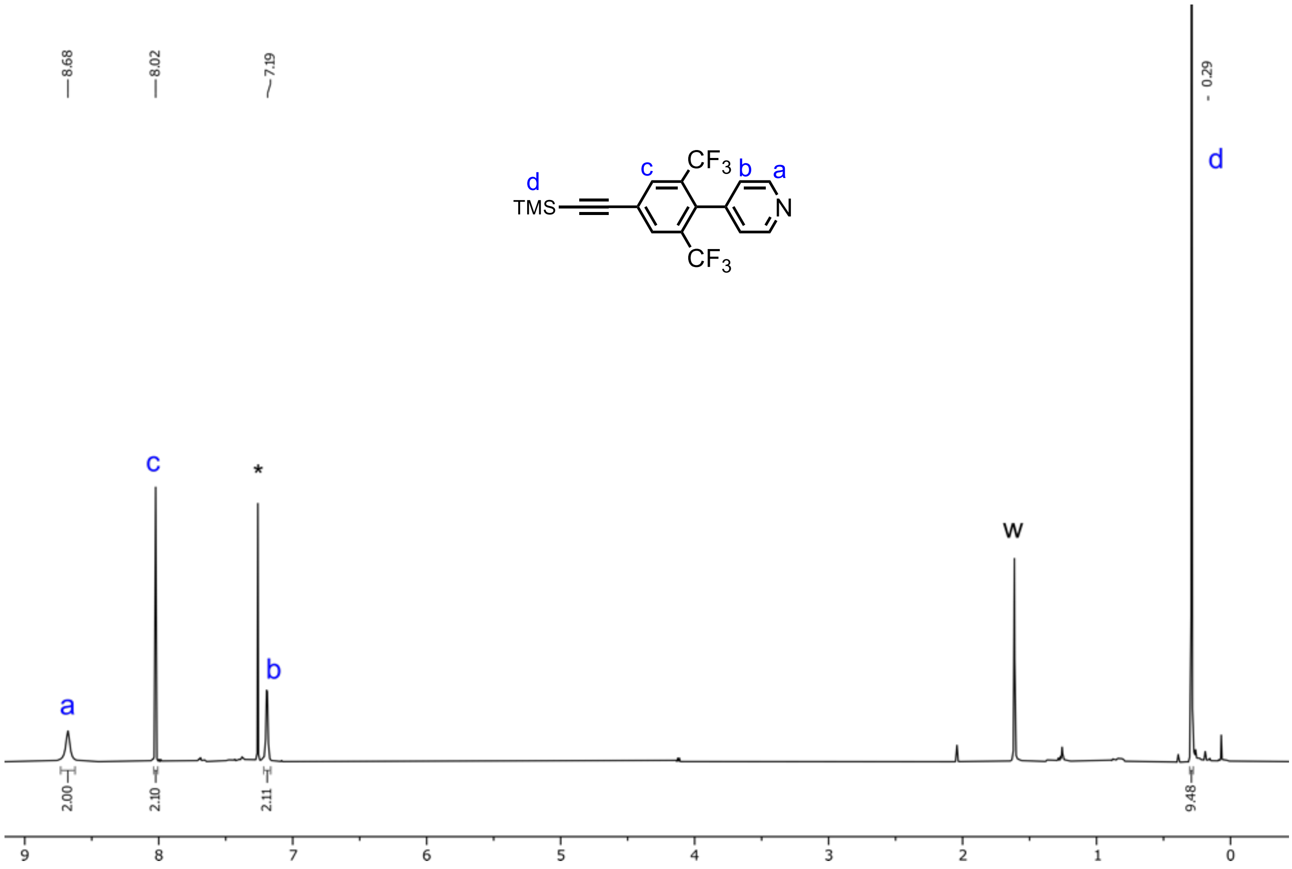

Figure S126. ^{1}H NMR spectrum of **12** (600 MHz, $CDCl_3$, 298 K). * = $CHCl_3$.

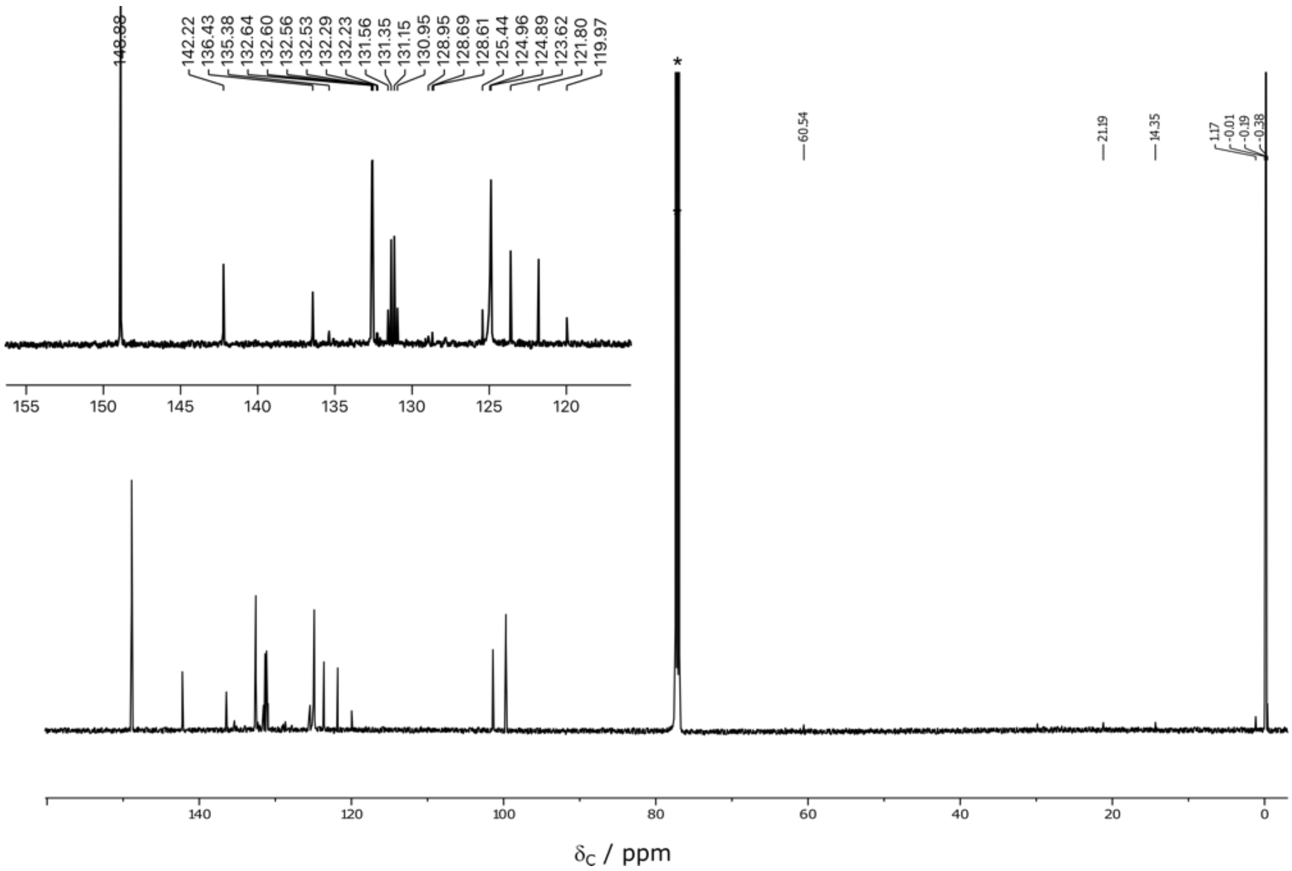

Figure S127. ^{13}C NMR spectrum of **12** (151 MHz, $CDCl_3$, 298 K). * = $CDCl_3$.

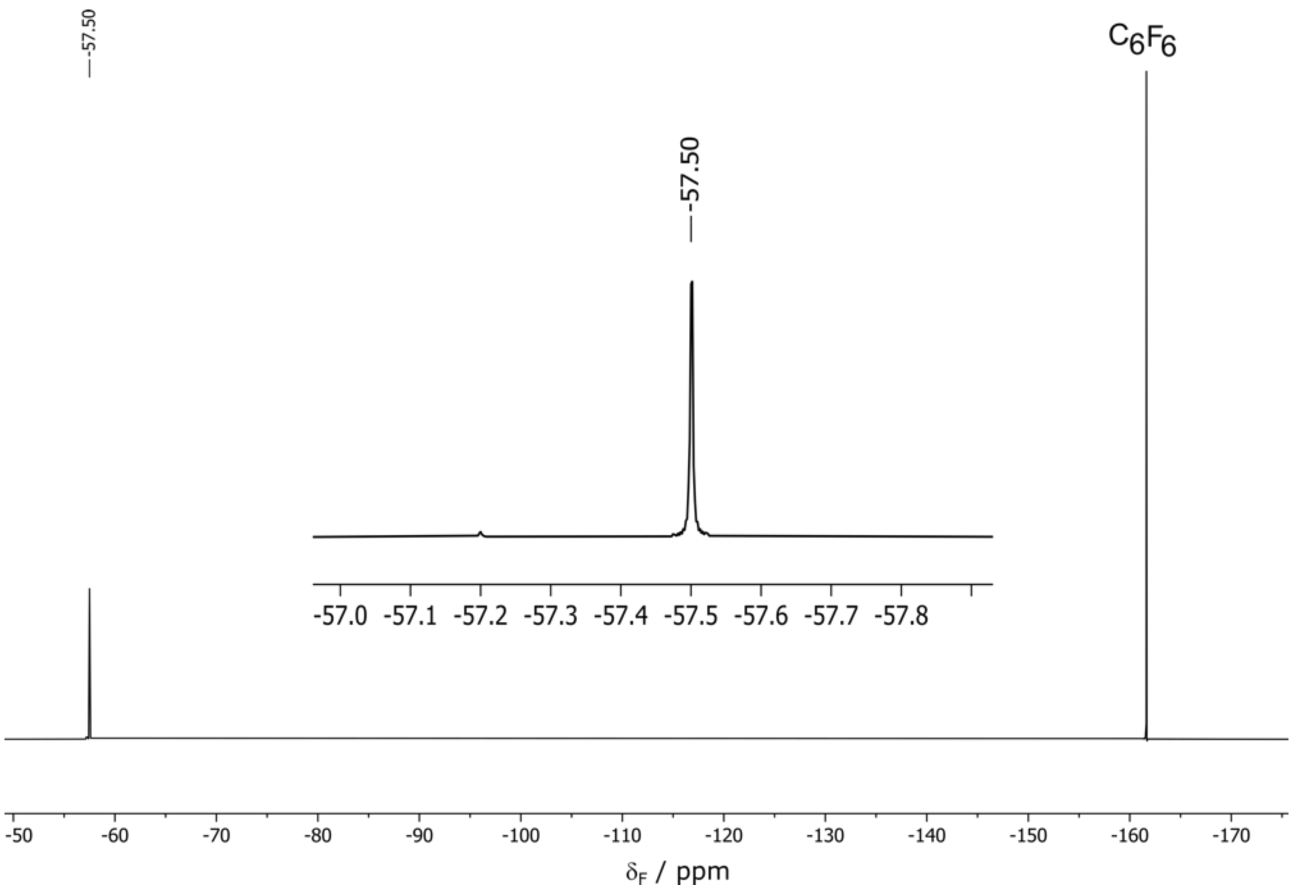

Figure S128. ^{19}F NMR spectrum of **12** (470 MHz, $CDCl_3$, 298 K). Referenced to C_6F_6 (δ_F = –161.64 ppm).

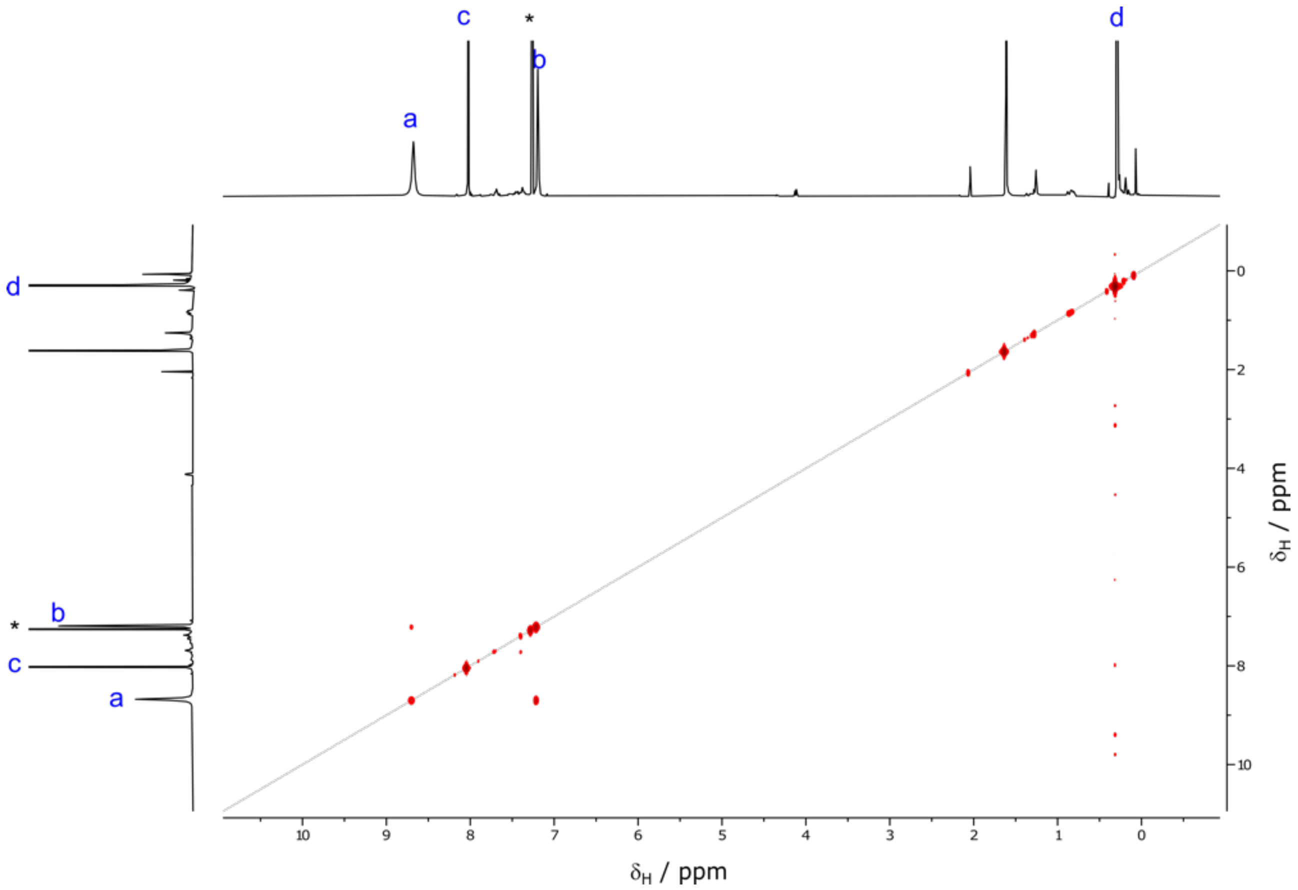

Figure S129. ^{1}H-^{1}H COSY spectrum of **12** (600 MHz, $CDCl_3$, 298 K). * = $CHCl_3$.

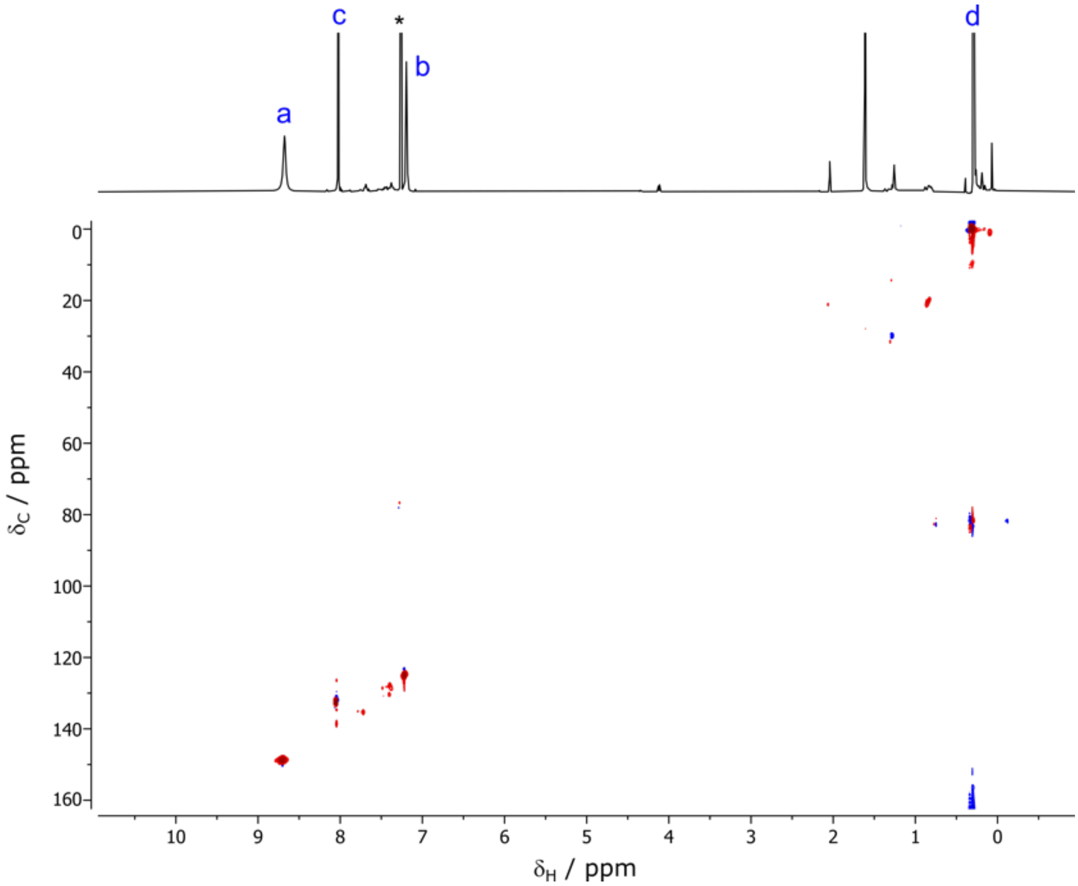

Figure S130. ^{1}H-^{13}C HSQC spectrum of **12** (600 MHz, $CDCl_3$, 298 K). * = $CHCl_3$.

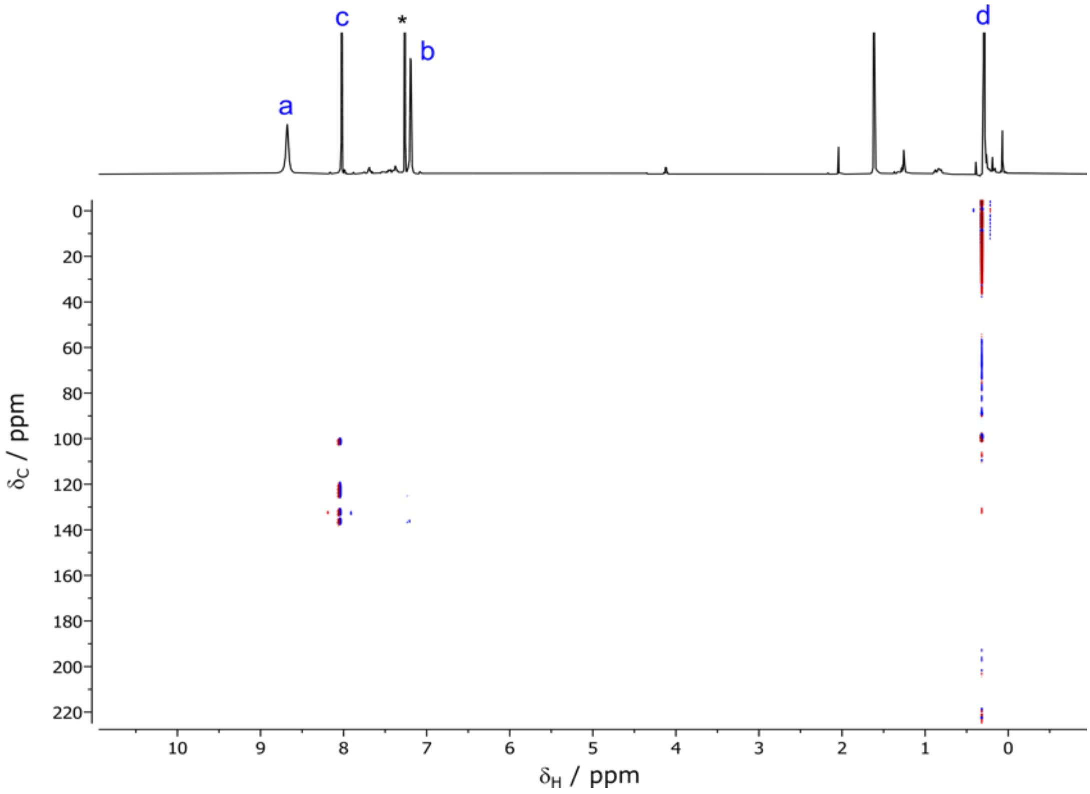

Figure S131. ^{1}H-^{13}C HMBC spectrum of **12** (600 MHz, $CDCl_3$, 298 K). * = $CHCl_3$.

Expanded Spectrum RT 0.18, NL 280871, Peak [1], Target Mass 388.0951

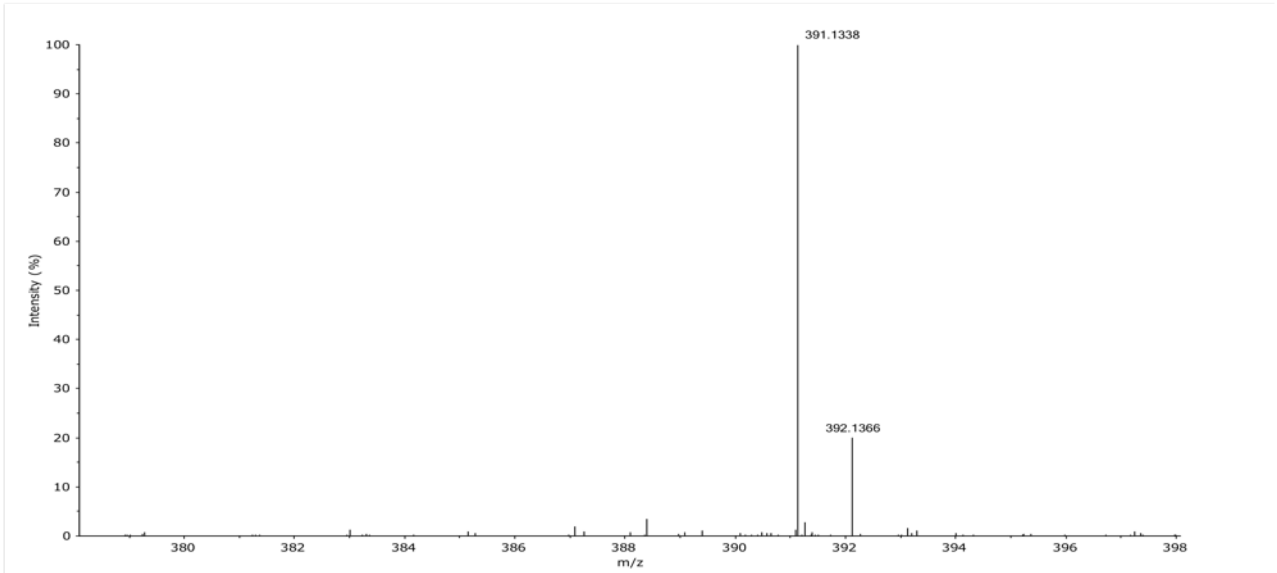

Theoretical Spectrum for C18H16F6NSi, Minimum Abundance 0.01%

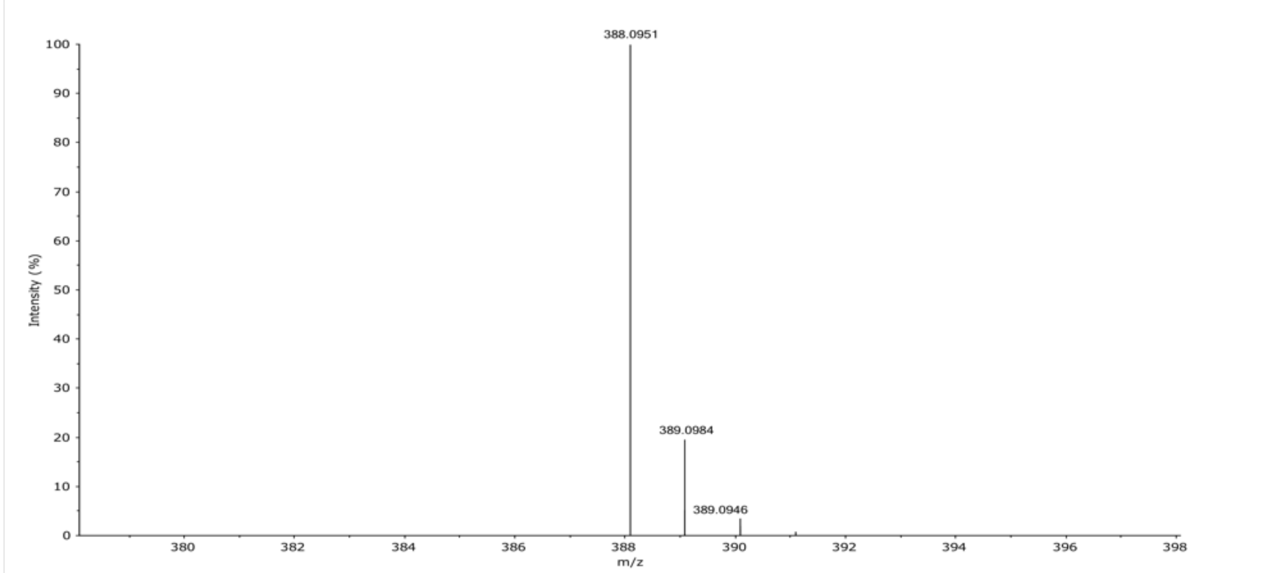

Measured Mass	Calculated Mass	Error (mDa)	Error (ppm)	Formula $[M+H]^+$	Response
388.3949	388.0951	299.82	772.55	$C_{18}H_{16}F_6NSi$	10488

Figure S132. High-resolution ESI+ mass spectrum of **12**.

Compound 13

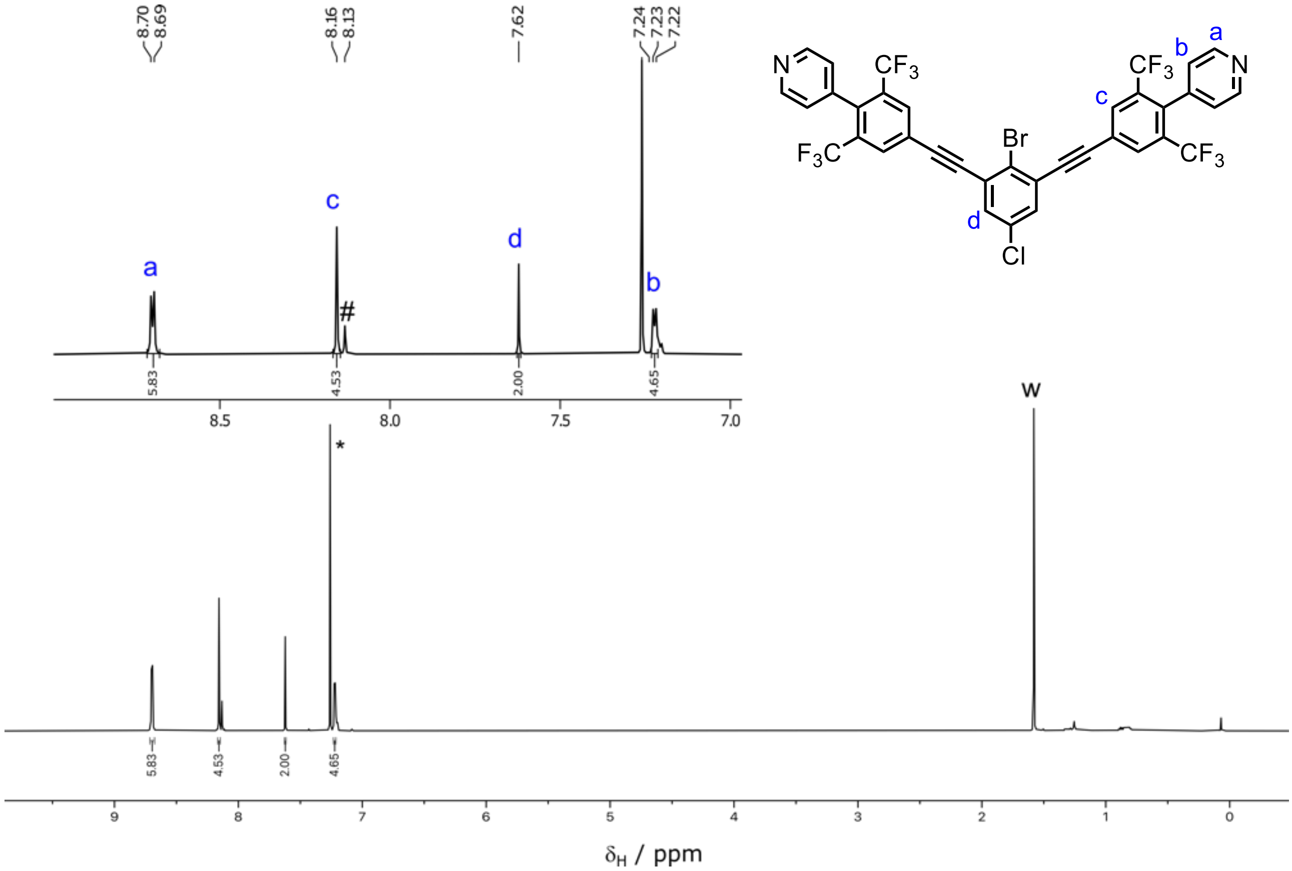

Figure S133. ^{1}H NMR spectrum of **13** (600 MHz, $CDCl_3$, 298 K). * = $CHCl_3$

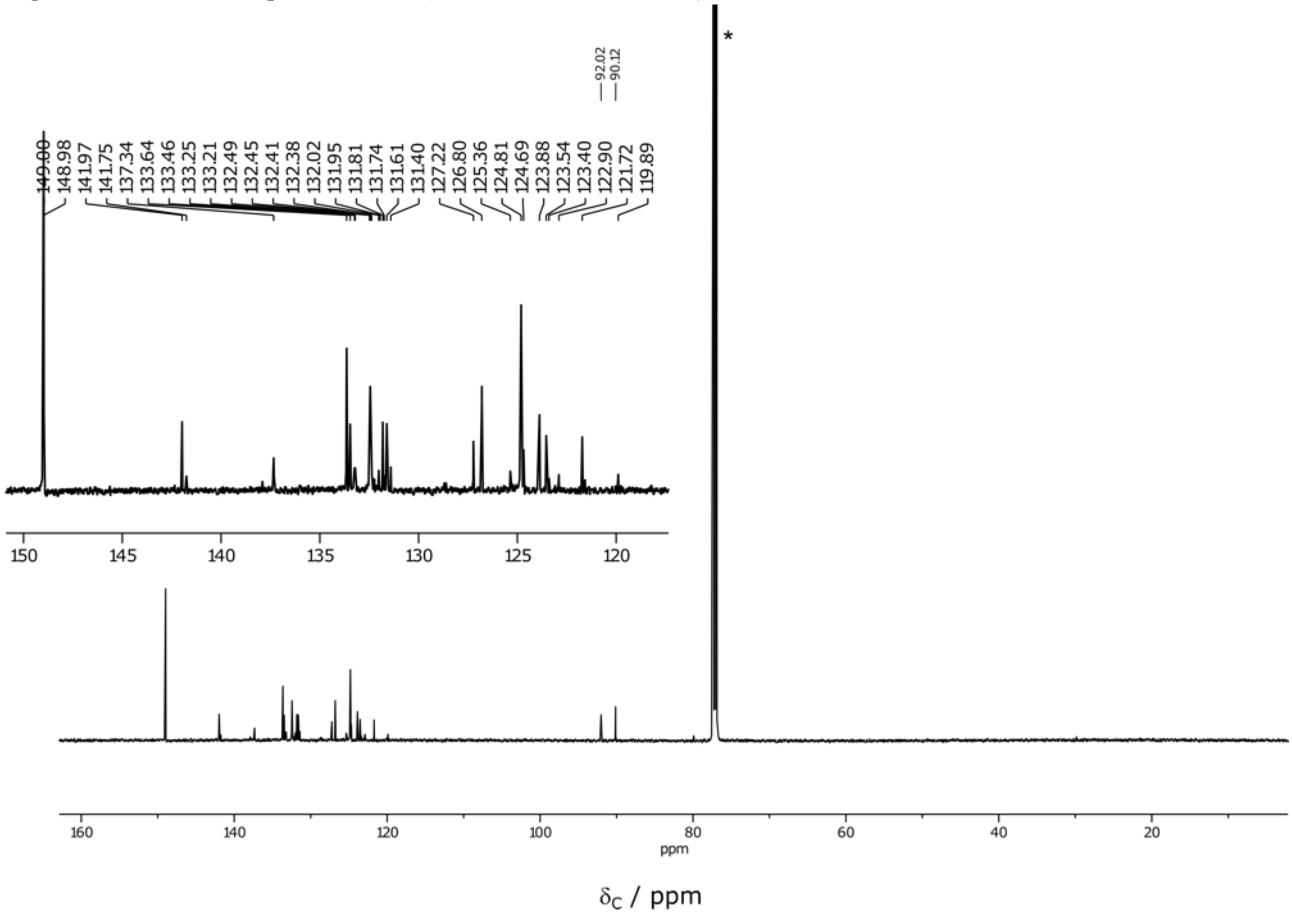

Figure S134. ^{13}C NMR spectrum of **13** (151 MHz, $CDCl_3$, 298 K). * = $CDCl_3$.

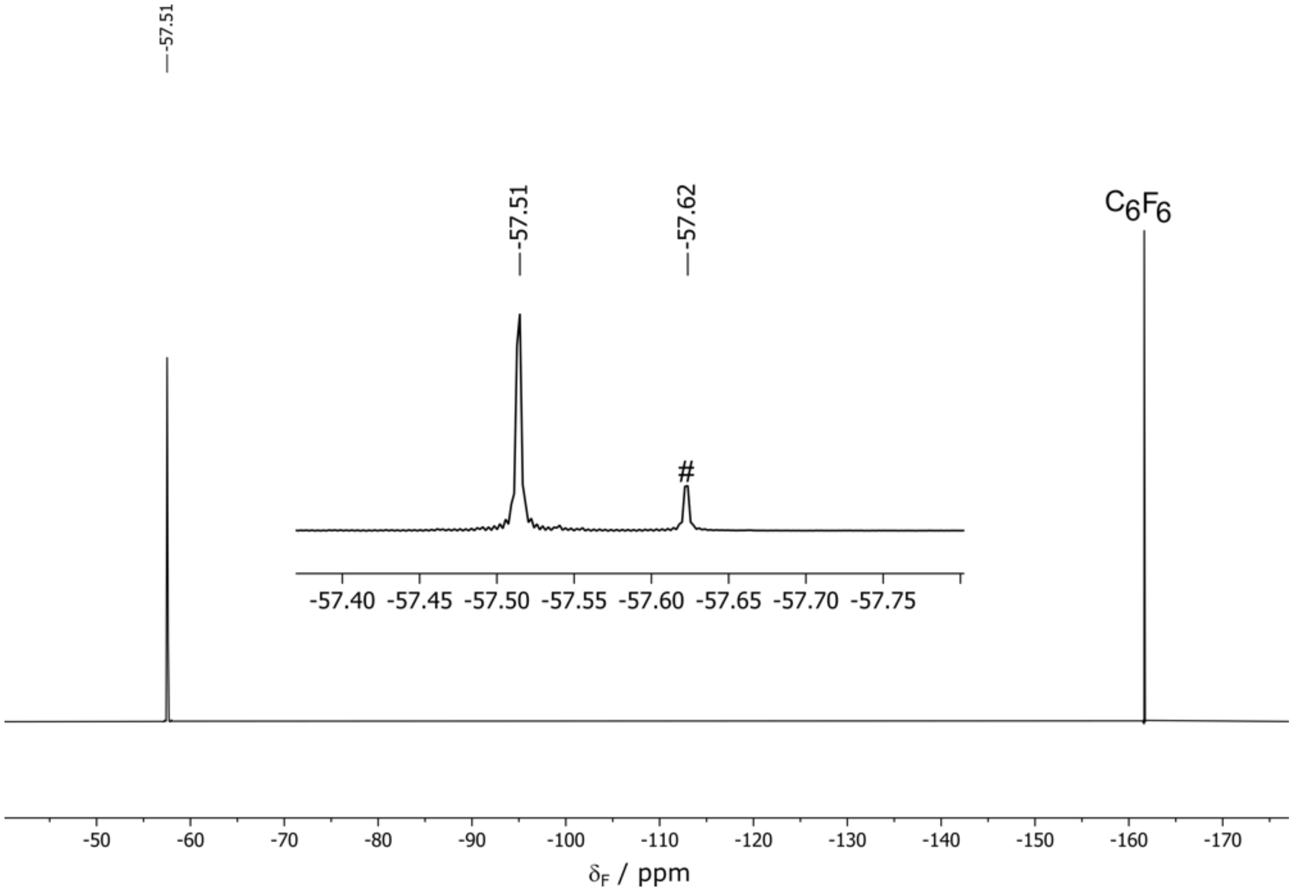

Figure S135. ^{19}F NMR spectrum of **13** (470 MHz, $CDCl_3$, 298 K). Referenced to C_6F_6 (δ_F = −161.64 ppm).

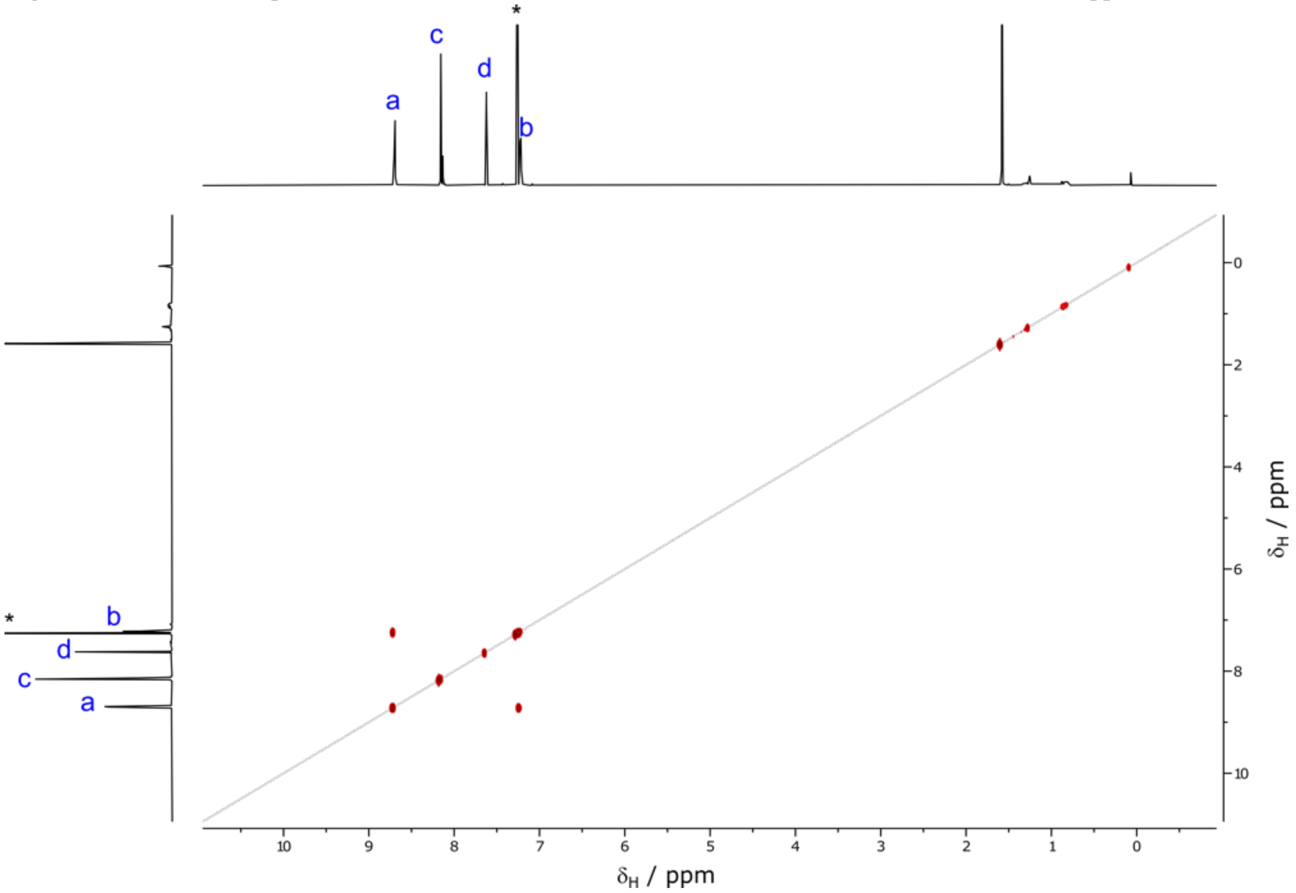

Figure S136. ^{1}H-^{1}H COSY spectrum of **13** (600 MHz, $CDCl_3$, 298 K). * = $CHCl_3$.

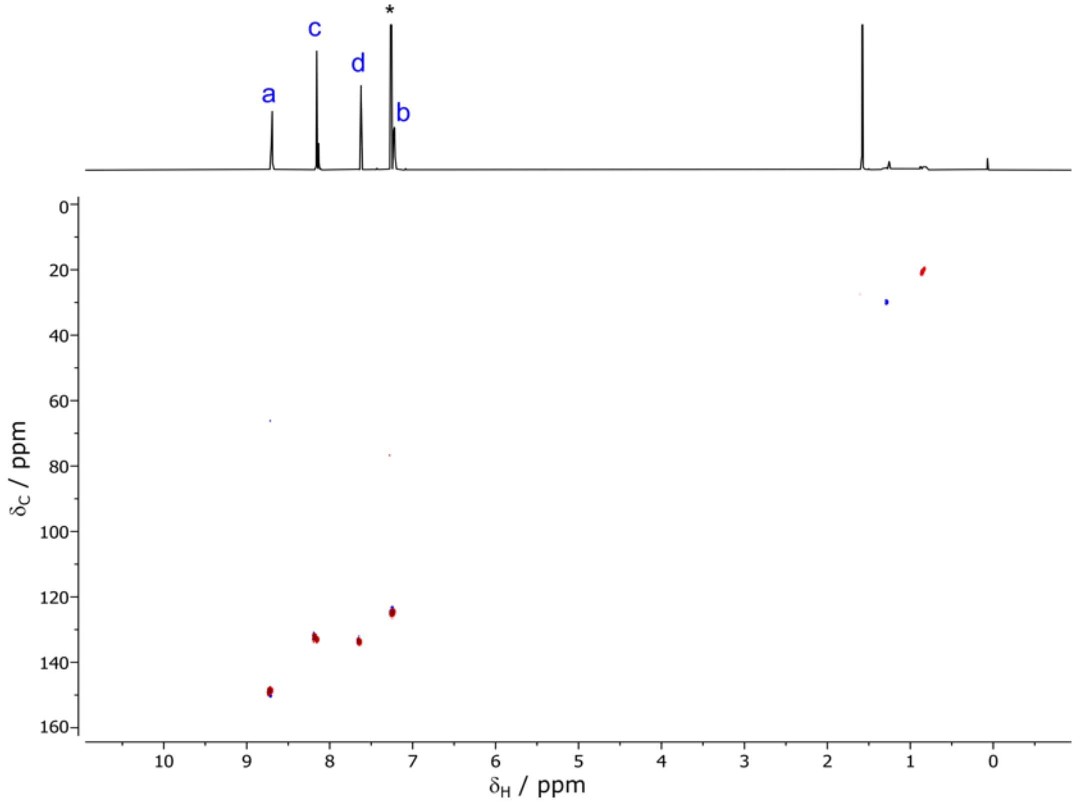

Figure S1317. ^{1}H-^{13}C HSQC spectrum of **13** (600 MHz, $CDCl_3$, 298 K). * = $CHCl_3$.

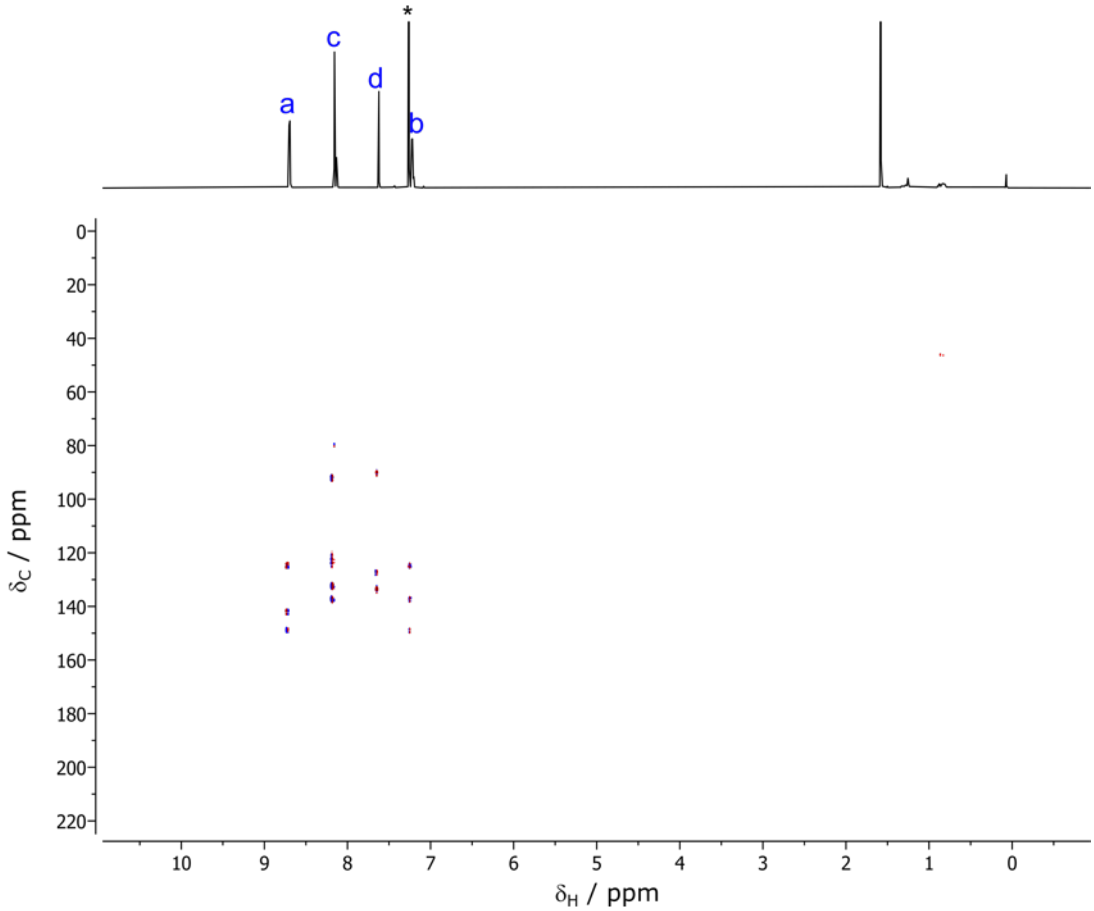

Figure S138. ^{1}H-^{13}C HMBC spectrum of **13** (600 MHz, $CDCl_3$, 298 K). * = $CHCl_3$.

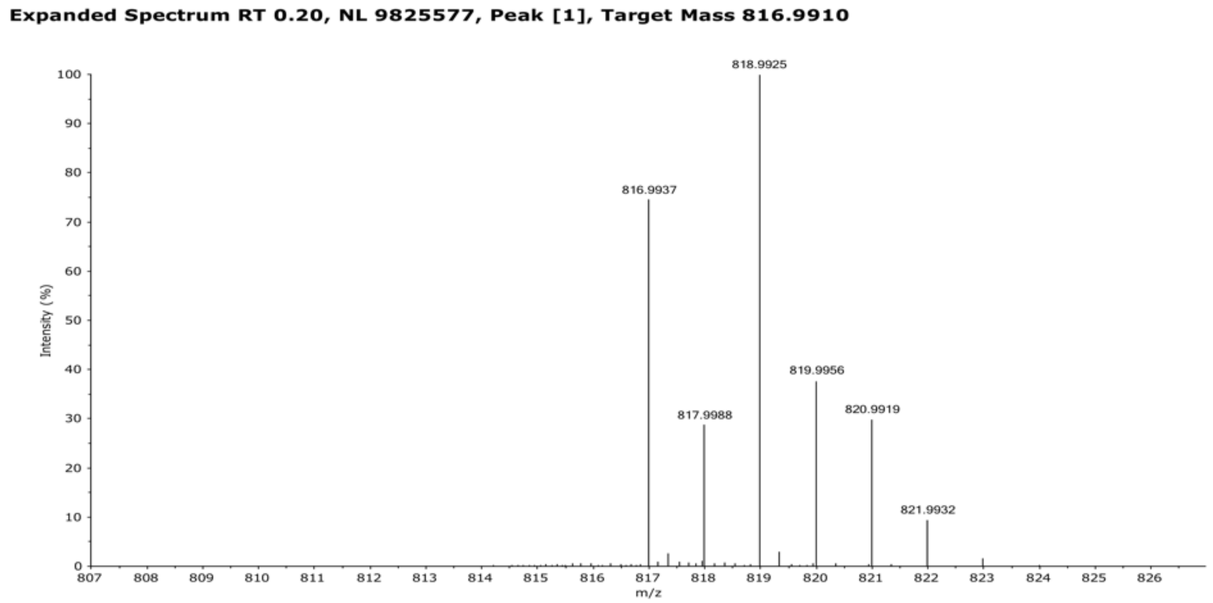

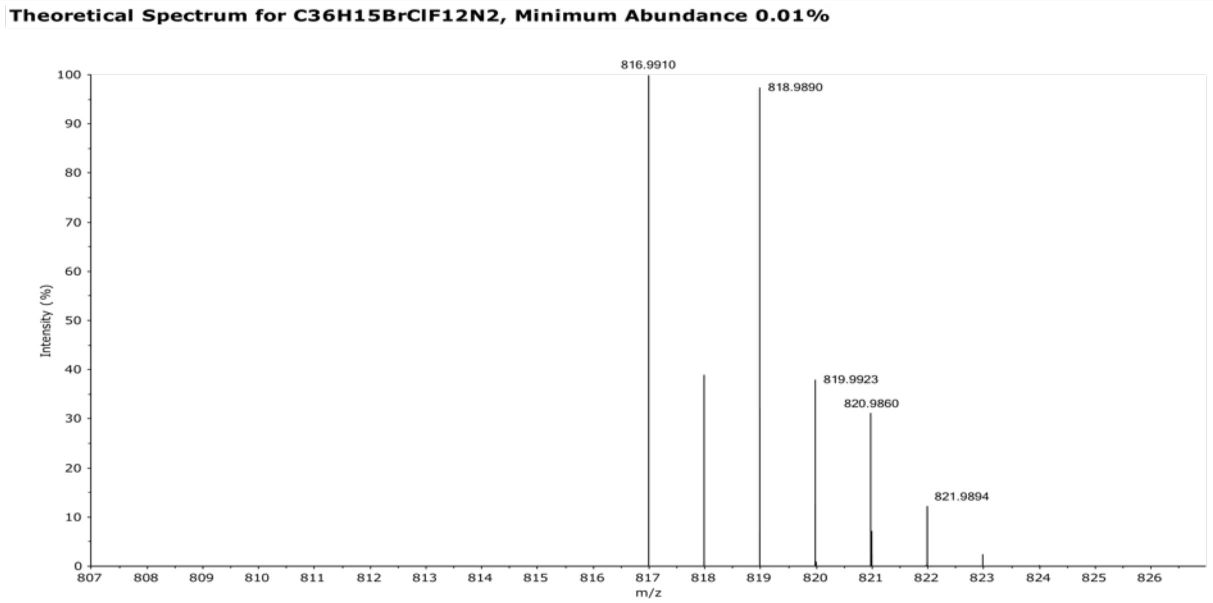

Measured Mass	Calculated Mass	Error (mDa)	Error (ppm)	Formula $[M+H]^+$	Response
816.9937	816.9910	2.70	3.30	$C_{36}H_{15}BrClF_{12}N_2$	9772780

Figure S139. High-resolution ESI+ mass spectrum of **13**.

Compound T3B

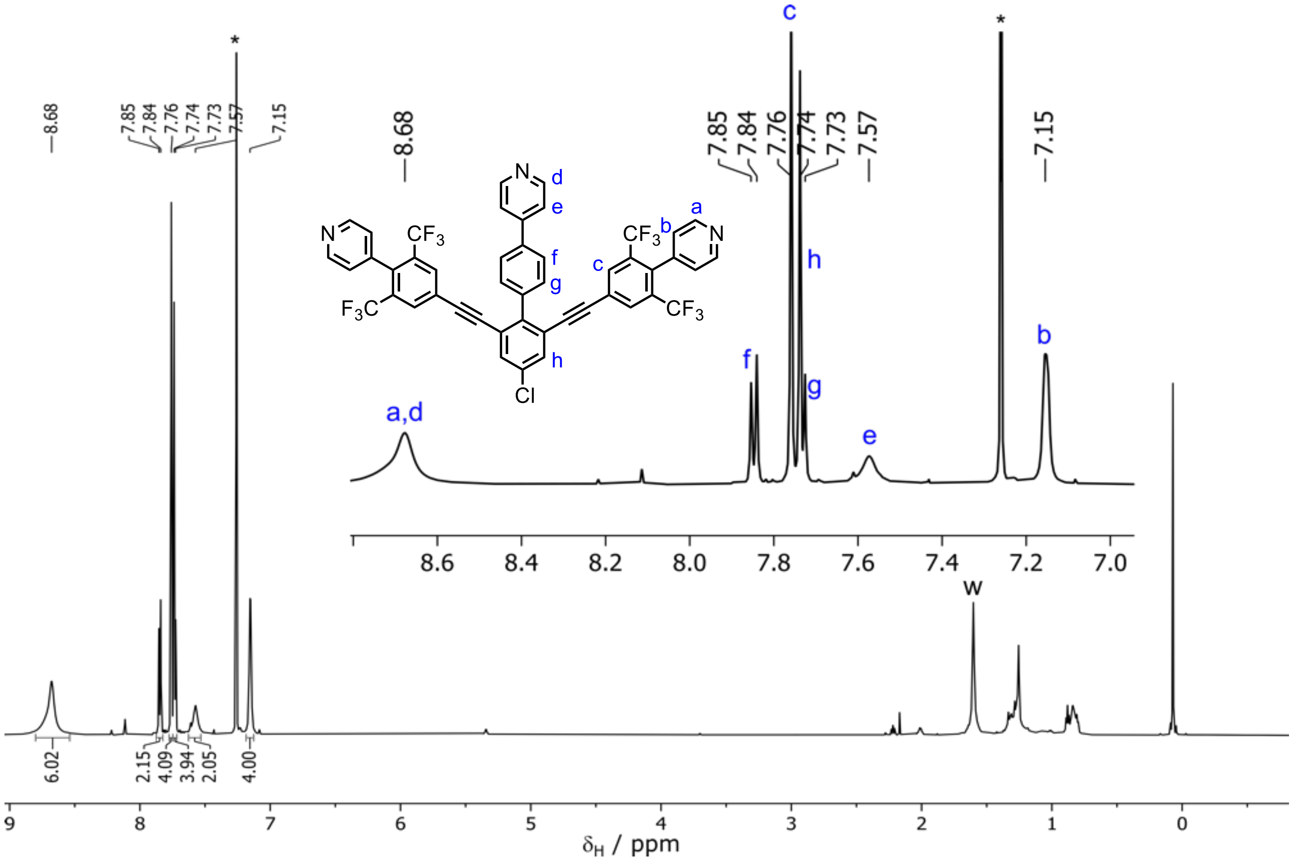

Figure S140. ^{1}H NMR spectrum of **T3B** (600 MHz, $CDCl_3$, 298 K). * = $CHCl_3$.

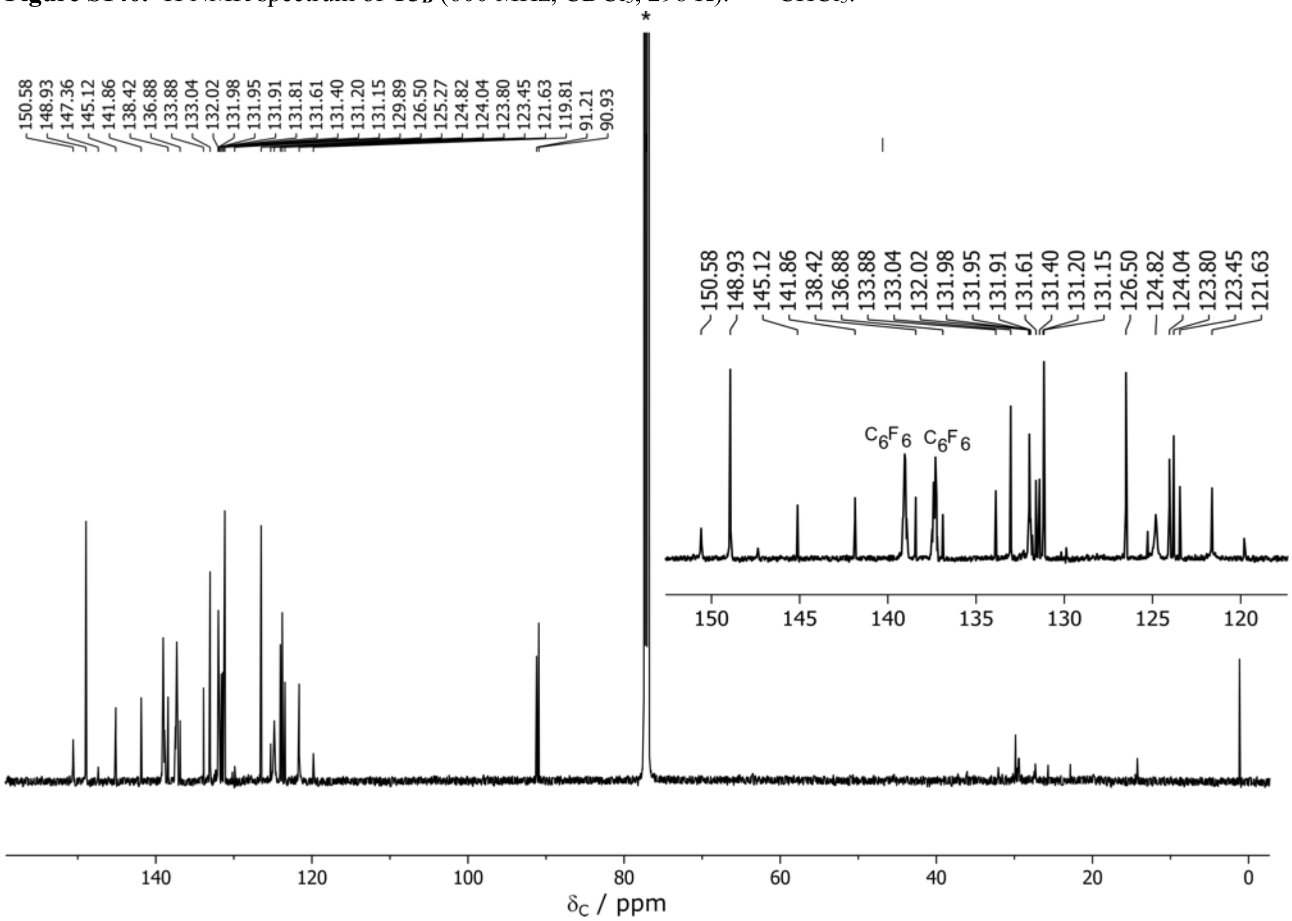

Figure S141. ^{13}C NMR spectrum of **T3B** (151 MHz, $CDCl_3$, 298 K). * = $CDCl_3$.

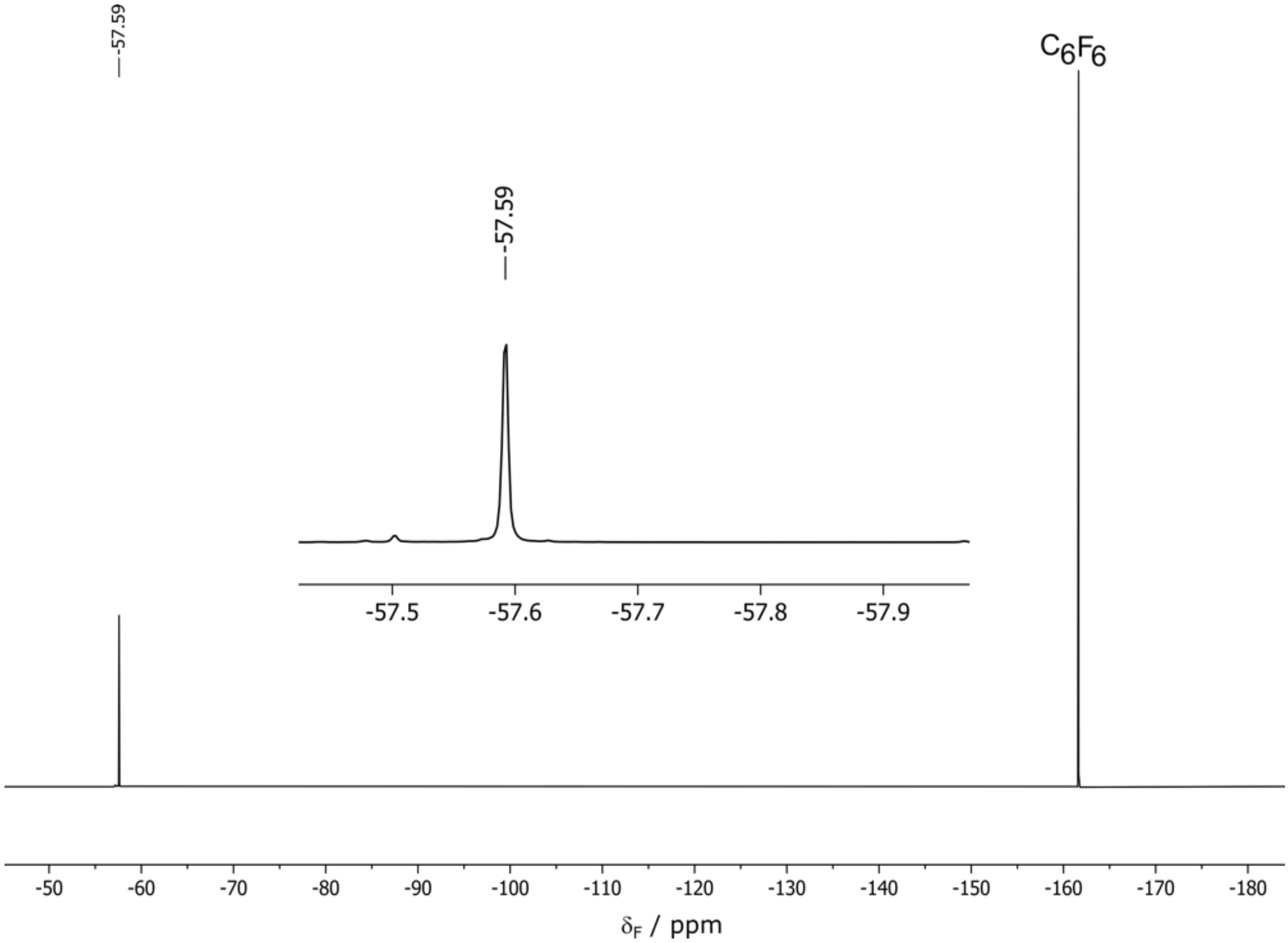

Figure S142. ^{19}F NMR spectrum of **T3B** (564 MHz, $CDCl_3$, 298 K). Referenced to C_6F_6 (δ_F = −161.64 ppm).

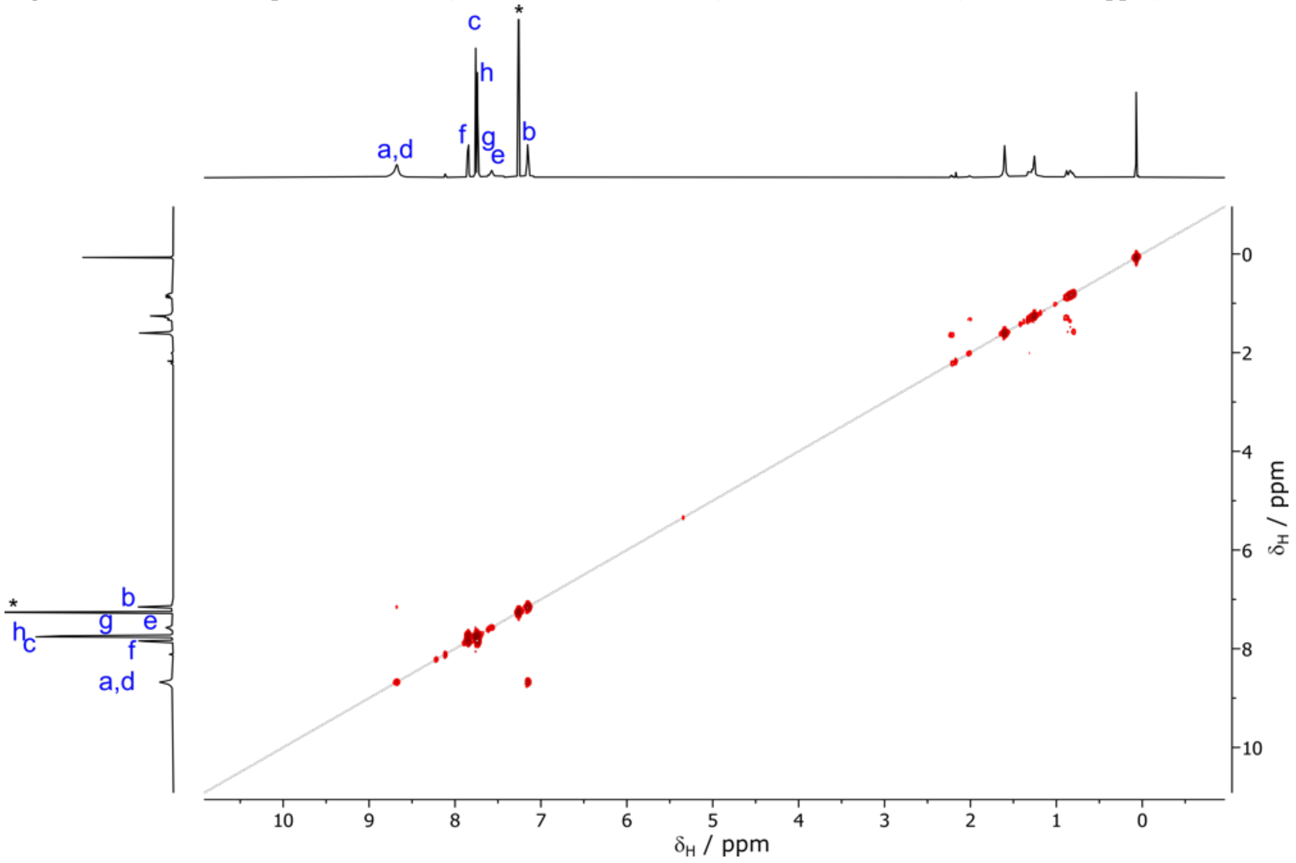

Figure S143. ^{1}H-^{1}H COSY spectrum of **T3B** (600 MHz, $CDCl_3$, 298 K). * = $CHCl_3$.

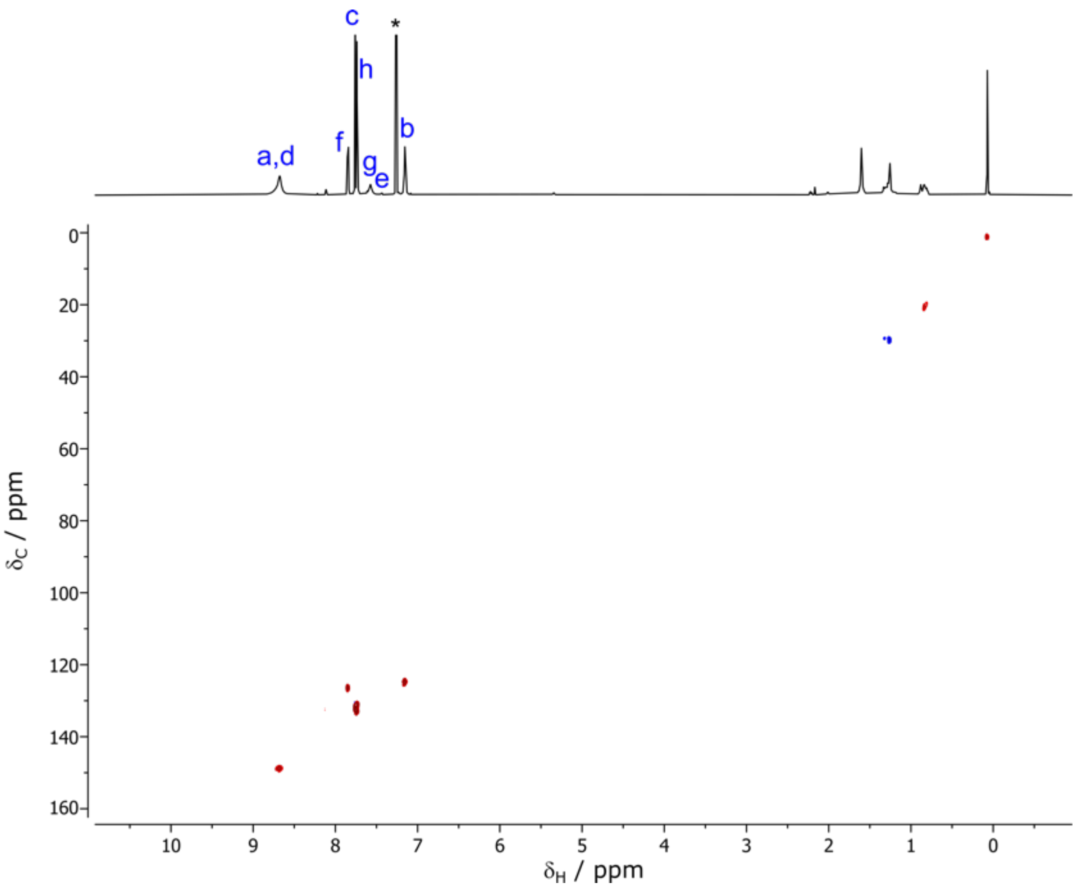

Figure S144. ^{1}H-^{13}C HSQC spectrum of **T3$_B$** (600 MHz, $CDCl_3$, 298 K). * = $CHCl_3$.

Expanded Spectrum RT 0.23, NL 9829861, Peak [1], Target Mass 892.1383

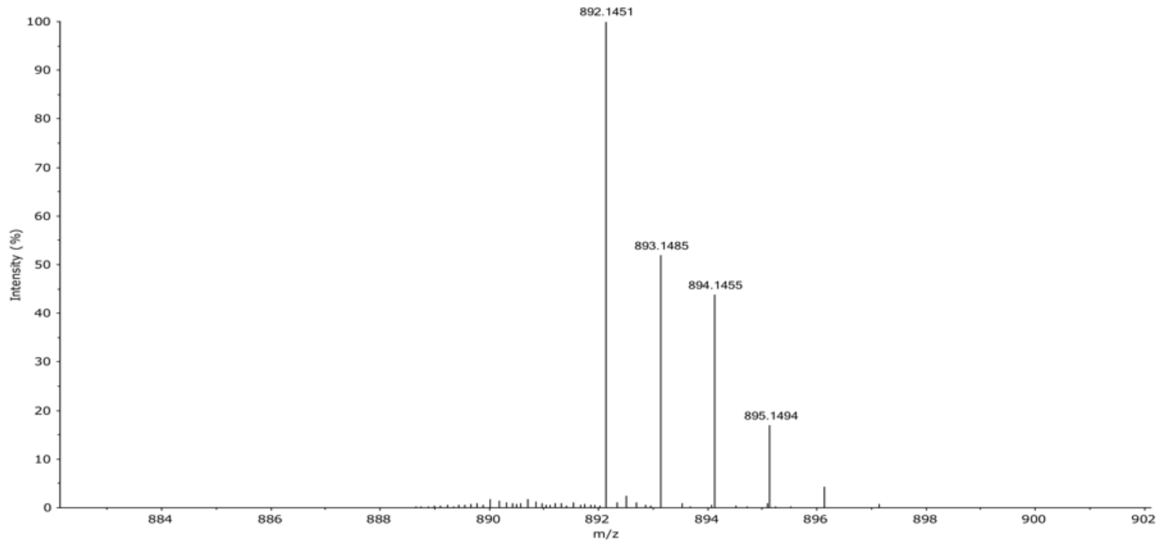

Theoretical Spectrum for C47H23ClF12N3, Minimum Abundance 0.01%

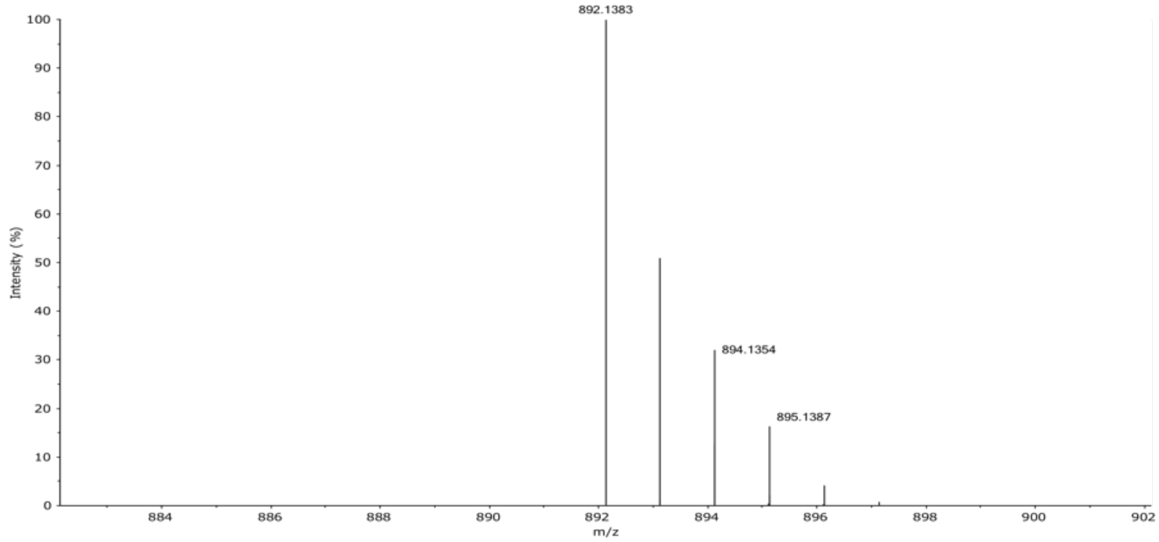

Measured Mass	Calculated Mass	Error (mDa)	Error (ppm)	Formula $[M+H]^+$	Response
892.1451	892.1383	6.76	7.58	$C_{47}H_{23}ClF_{12}N_3$	13783191

Figure S145. High-resolution ESI+ mass spectrum of **T3$_B$**.

Compound 14

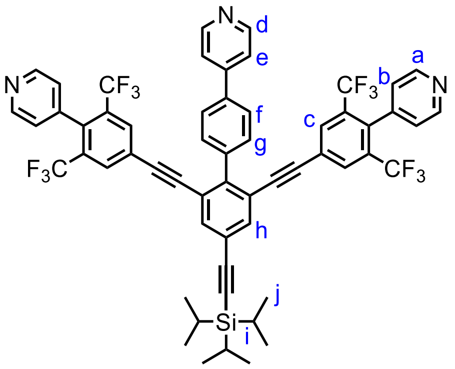

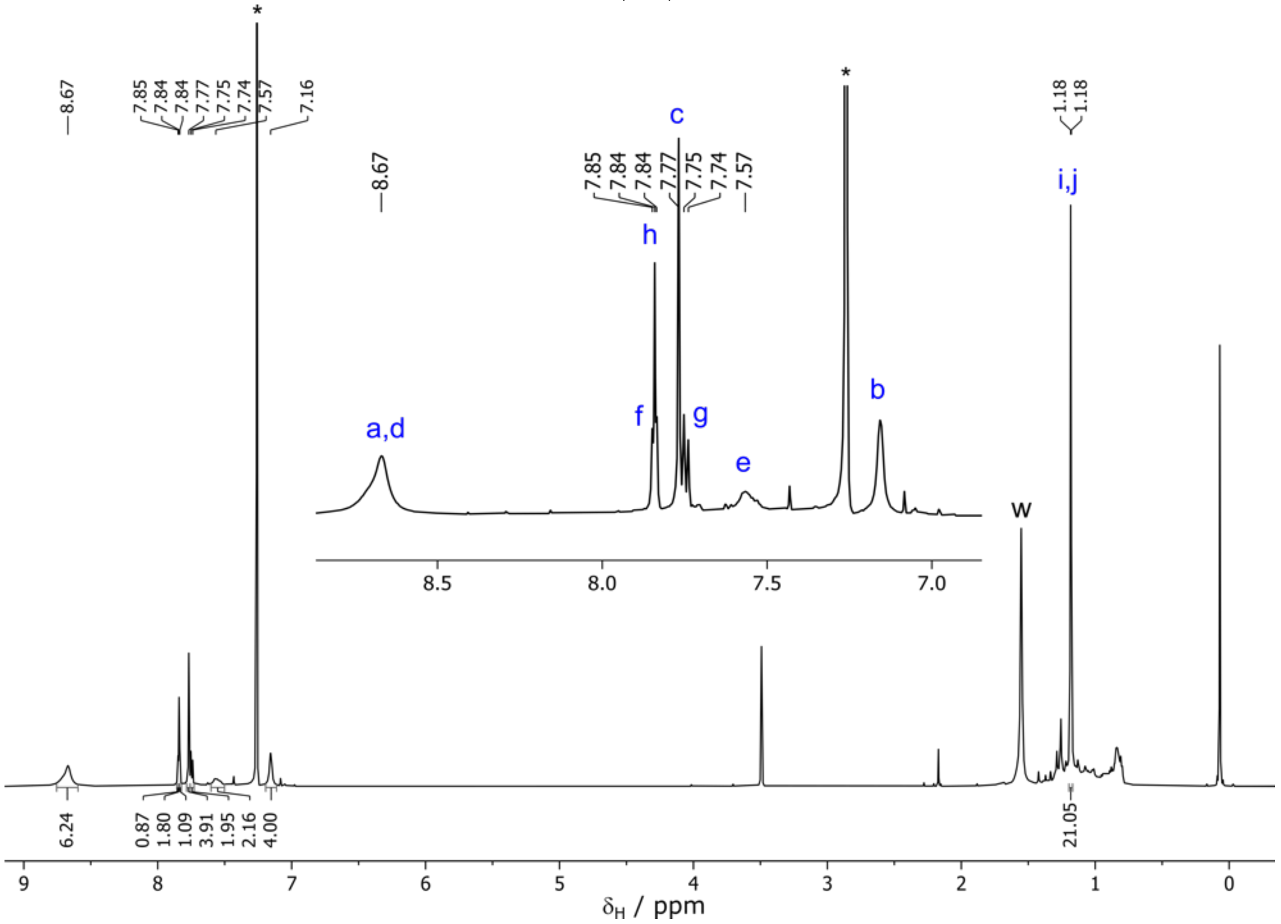

Figure S146. ^{1}H NMR spectrum of **14** (600 MHz, $CDCl_3$, 298 K). * = $CHCl_3$.

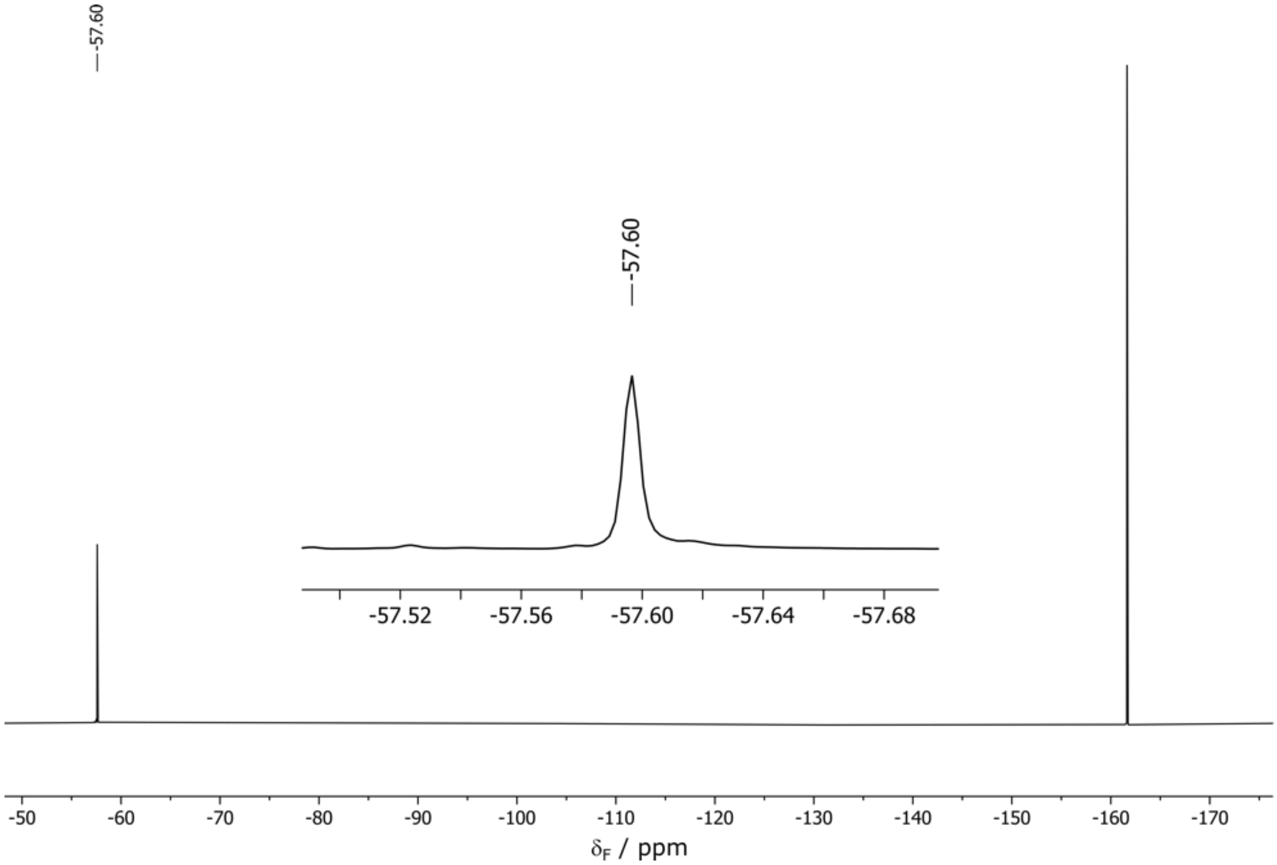

Figure S147. ^{19}F NMR spectrum of **14** (564 MHz, $CDCl_3$, 298 K). Referenced to C_6F_6 (δ_F = –161.64 ppm).

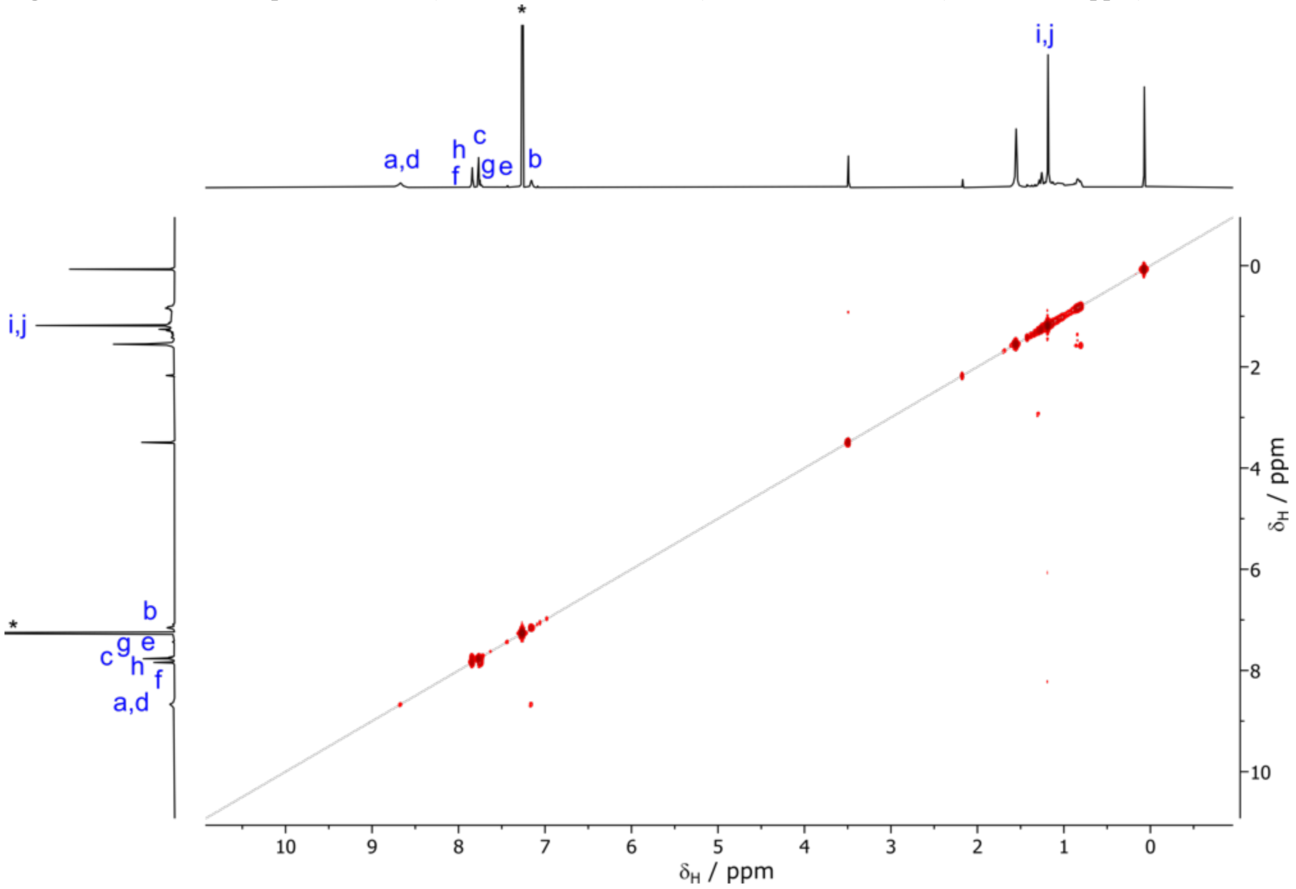

Figure S148. ^{1}H-^{1}H COSY spectrum of **14** (600 MHz, $CDCl_3$, 298 K). * = $CHCl_3$.

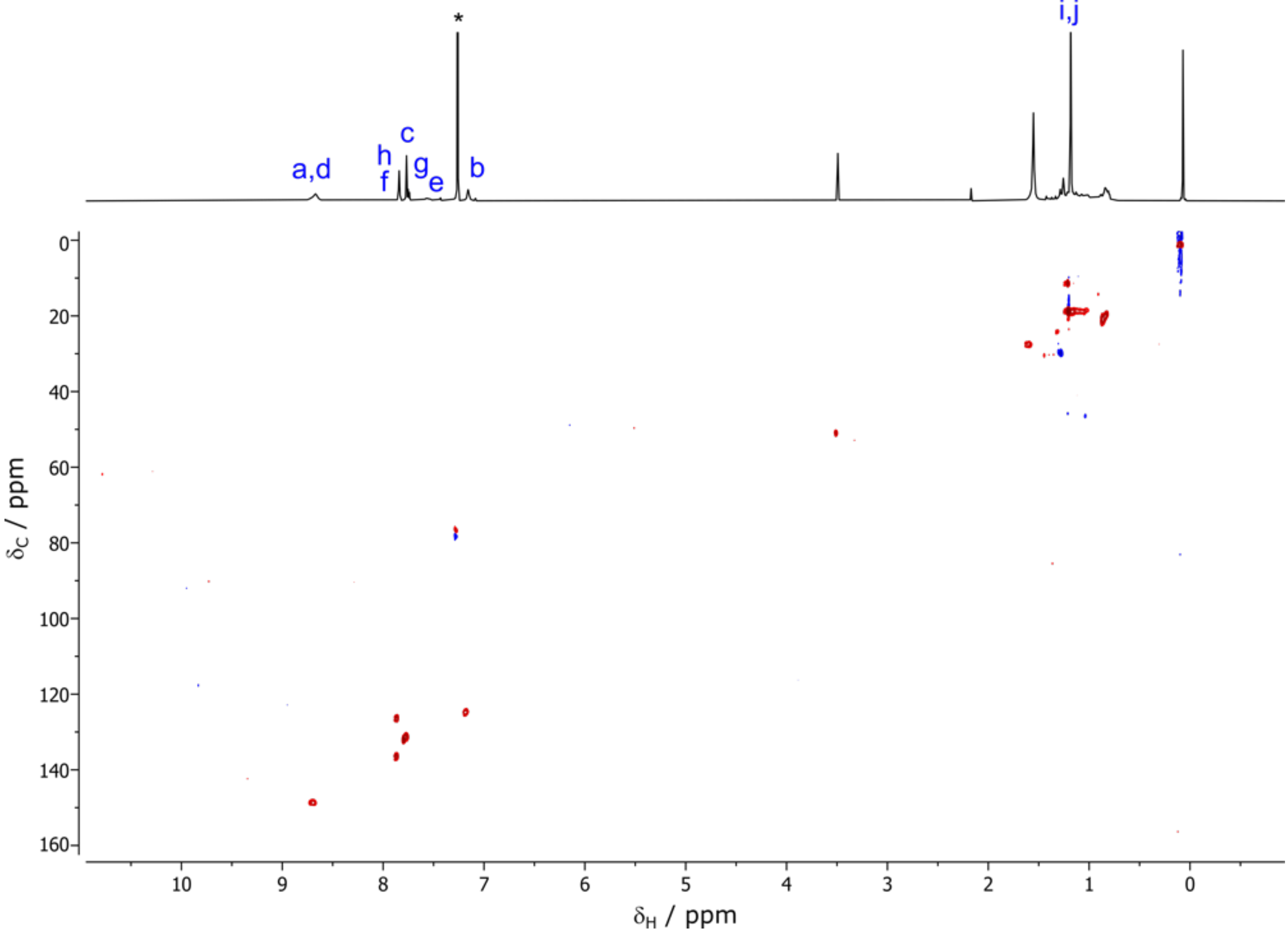

Figure S149. ^{1}H-^{13}C HSQC spectrum of **14** (600 MHz, $CDCl_3$, 298 K). * = $CHCl_3$.

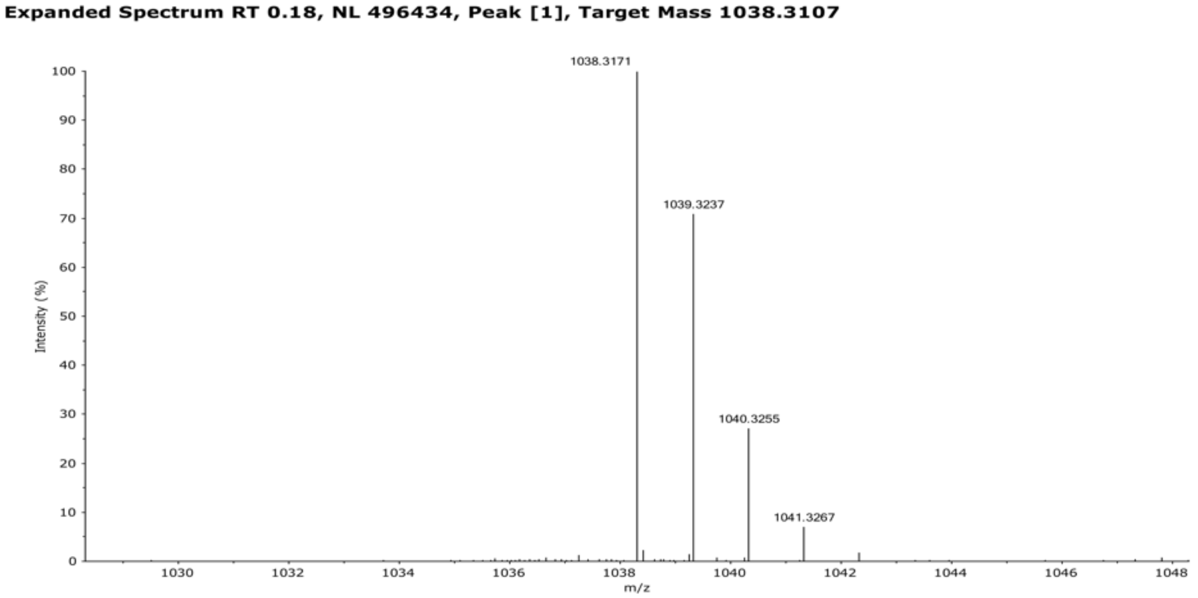

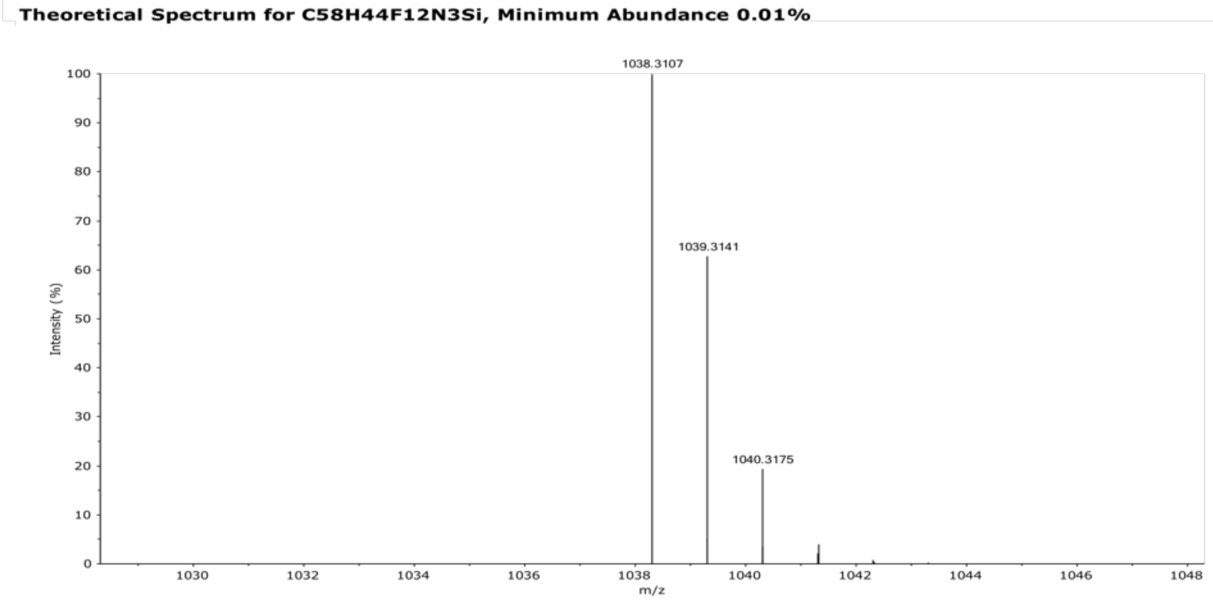

Measured Mass	Calculated Mass	Error (mDa)	Error (ppm)	Formula $[M+H]^+$	Response
1038.3171	1038.3107	6.36	6.12	$C_{58}H_{44}F_{12}N_3Si$	441494

Figure S150. High-resolution ESI+ mass spectrum of **14**.

*Spectra and assignment of **T18_B***

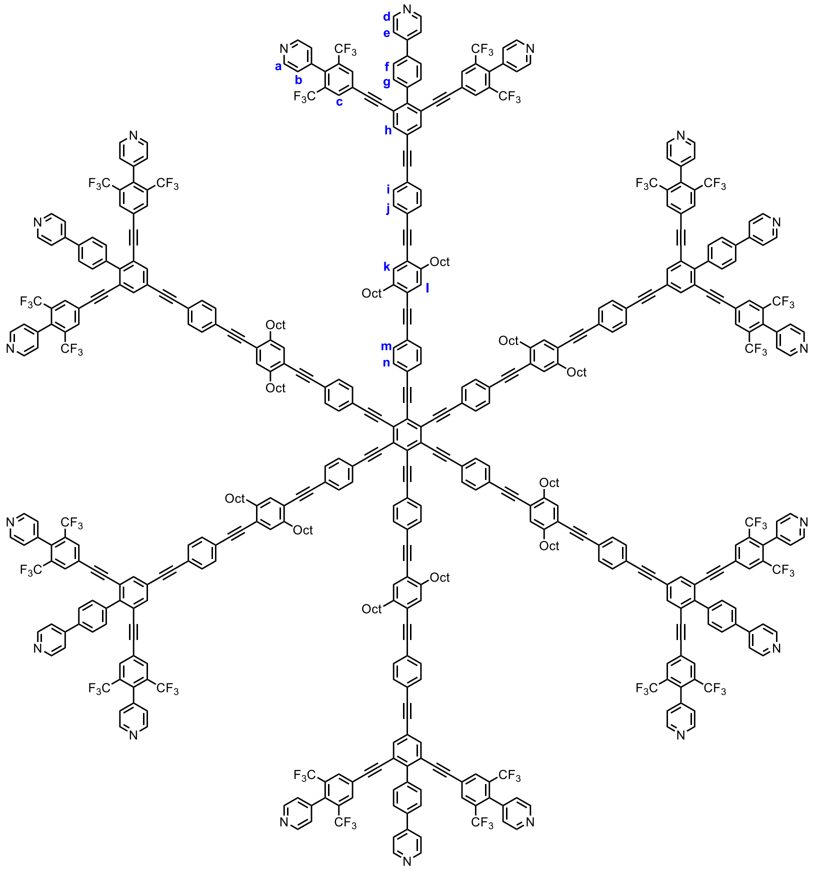

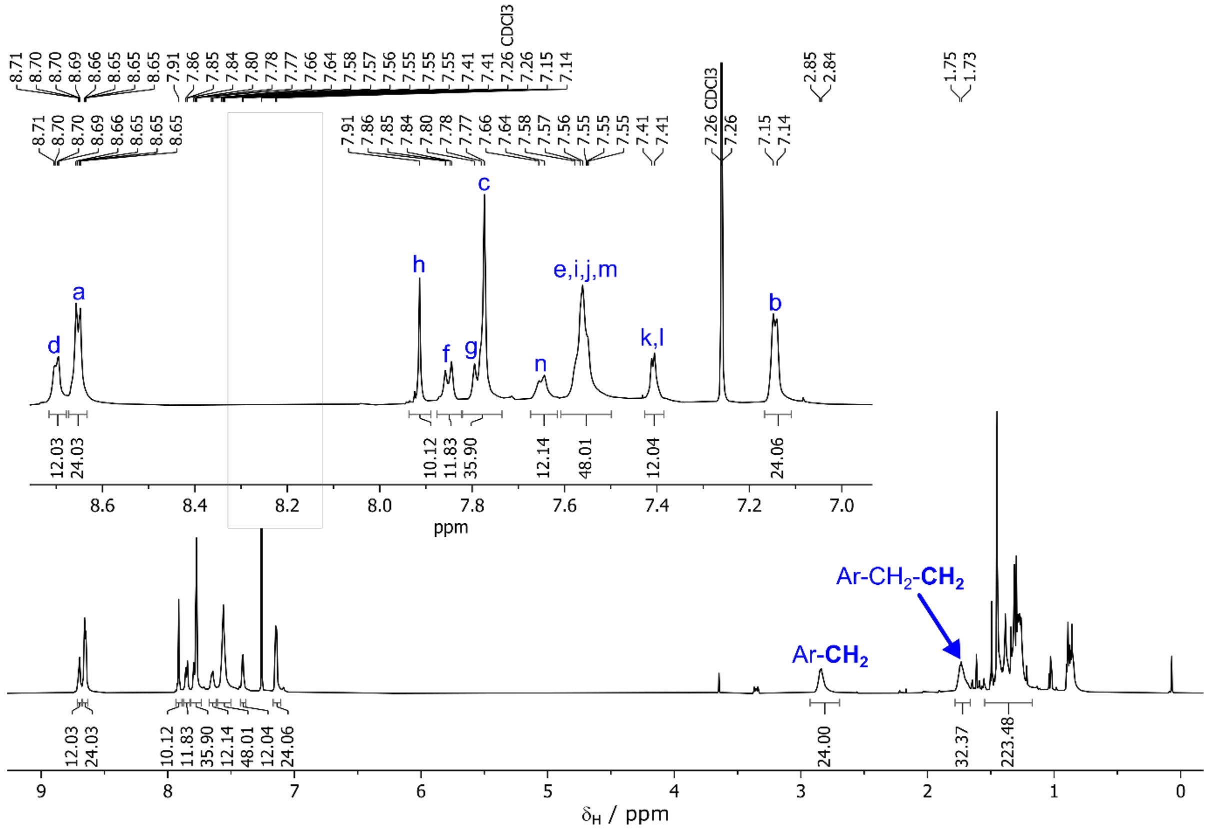

Figure S151. ^{1}H NMR spectrum of **T18$_B$** (600 MHz, $CDCl_3$, 298 K). * = $CHCl_3$

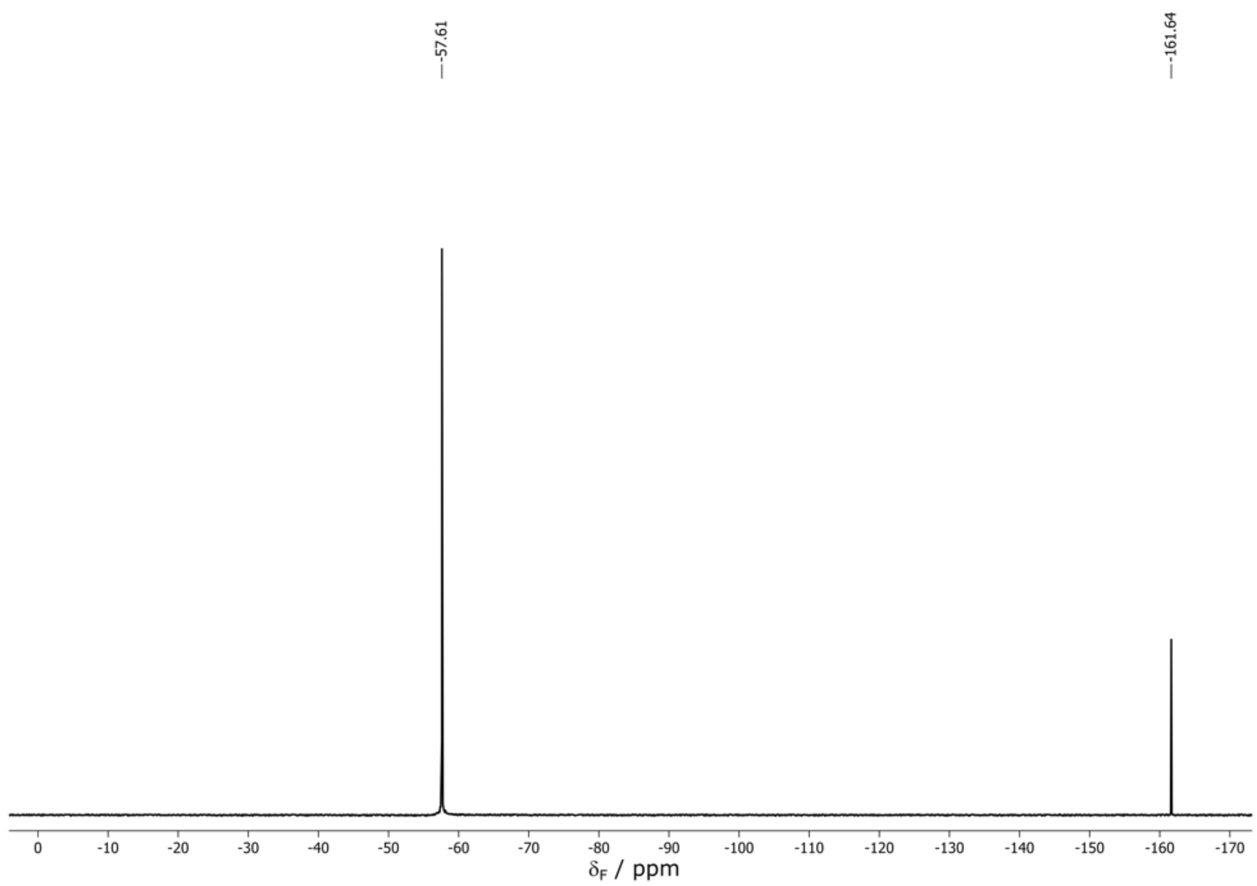

Figure S152. ^{19}F NMR spectrum of **T18B** (470 MHz, $CDCl_3$, 298 K) (Referenced against C_6F_6 ($\delta_F = -161.64$) as an internal standard).

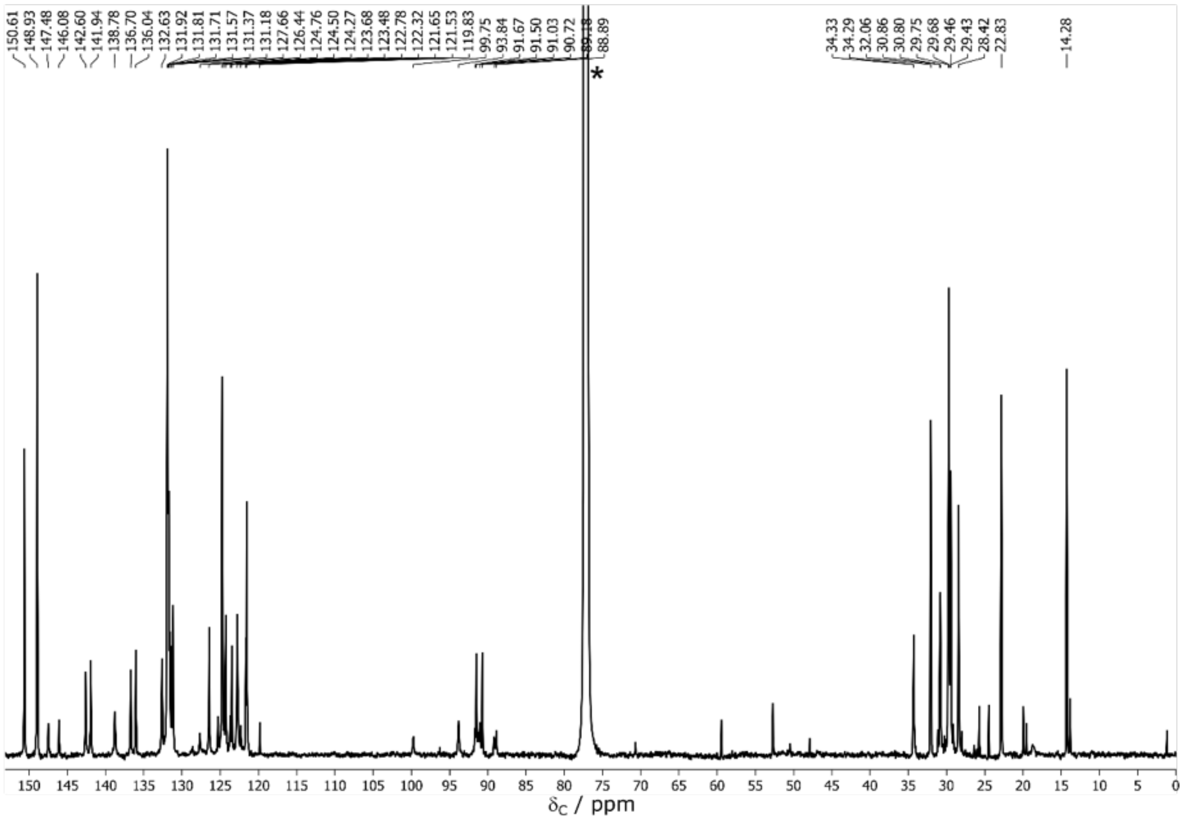

Figure S153. ^{13}C NMR spectrum of **T18B** (151 MHz, $CDCl_3$, 298 K). * = $CDCl_3$

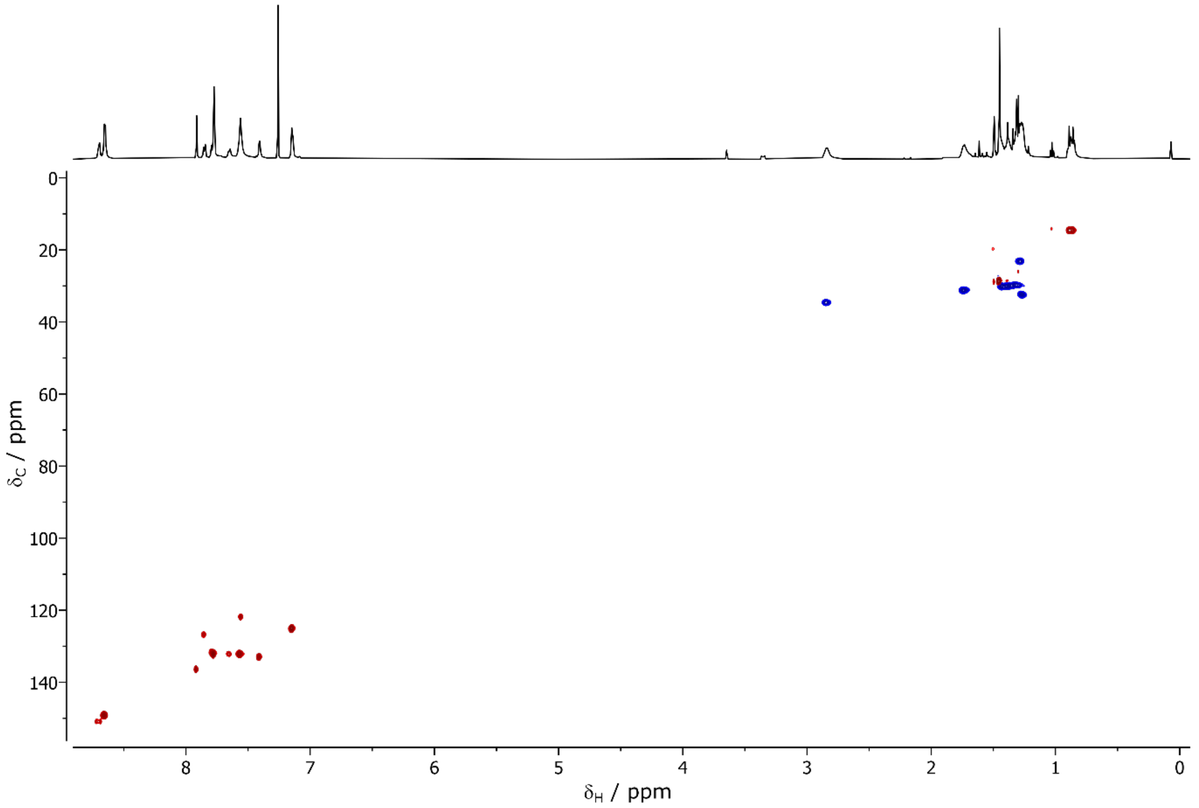

Figure S154. ^{1}H-^{13}C HSQC spectrum of **T18B** (600 MHz, $CDCl_3$, 298 K).

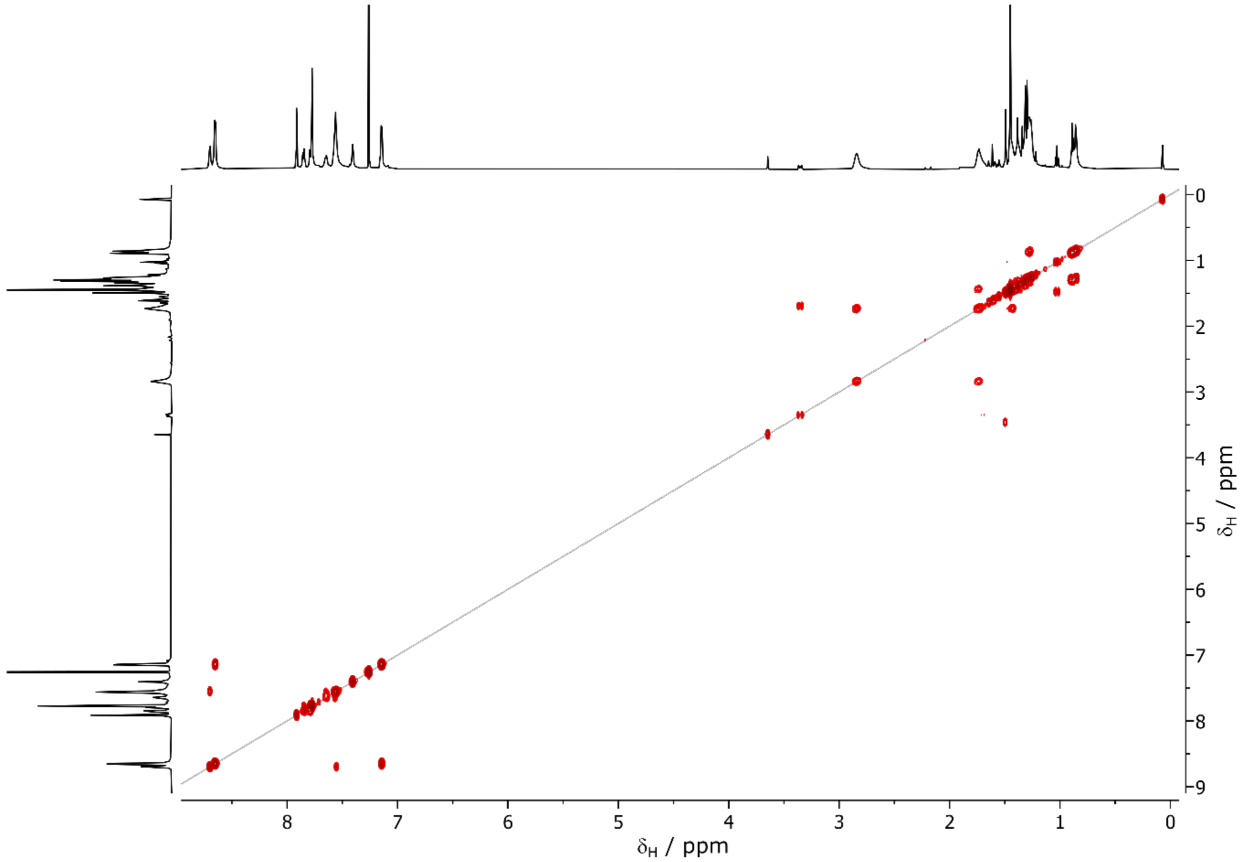

Figure S155. ^{1}H-^{1}H COSY spectrum of **T18B** (600 MHz, $CDCl_3$, 298 K).

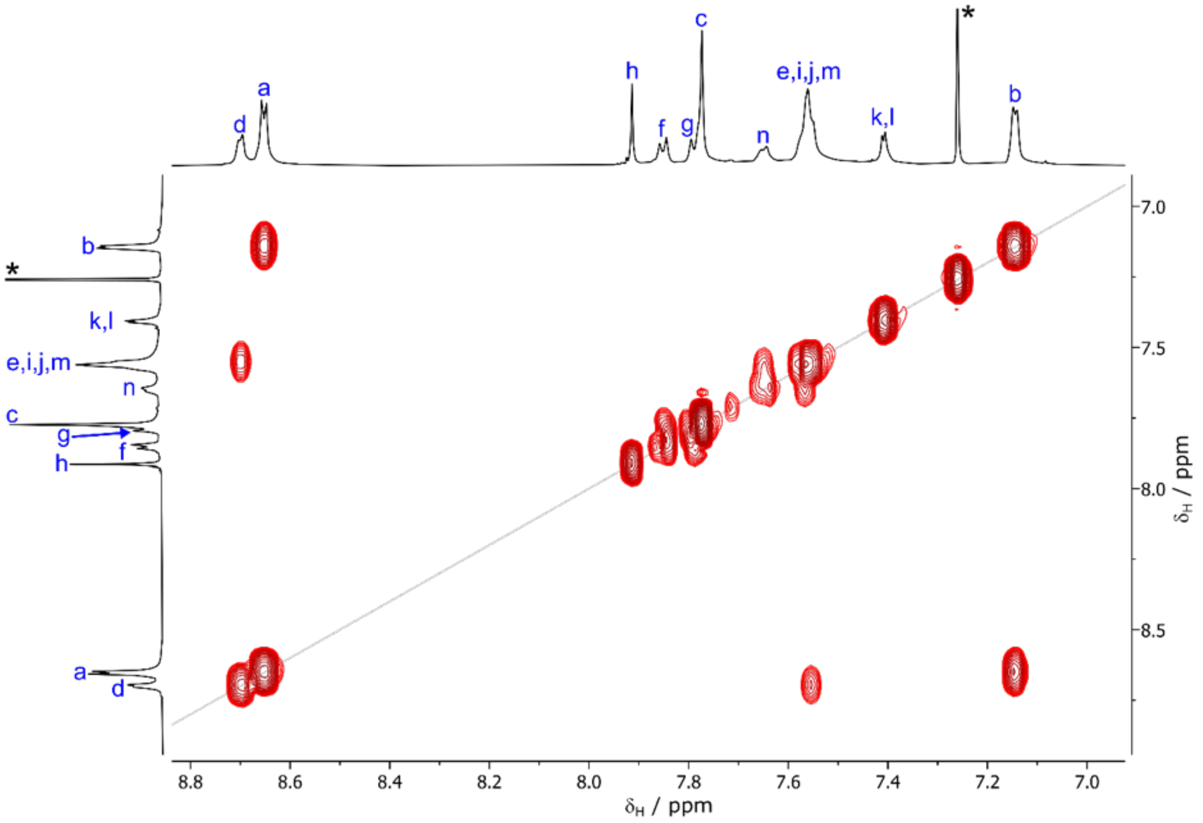

Figure S156. Aromatic region of the ^{1}H-^{1}H COSY spectrum of **T18B** (600 MHz, $CDCl_3$, 298 K). * = $CHCl_3$

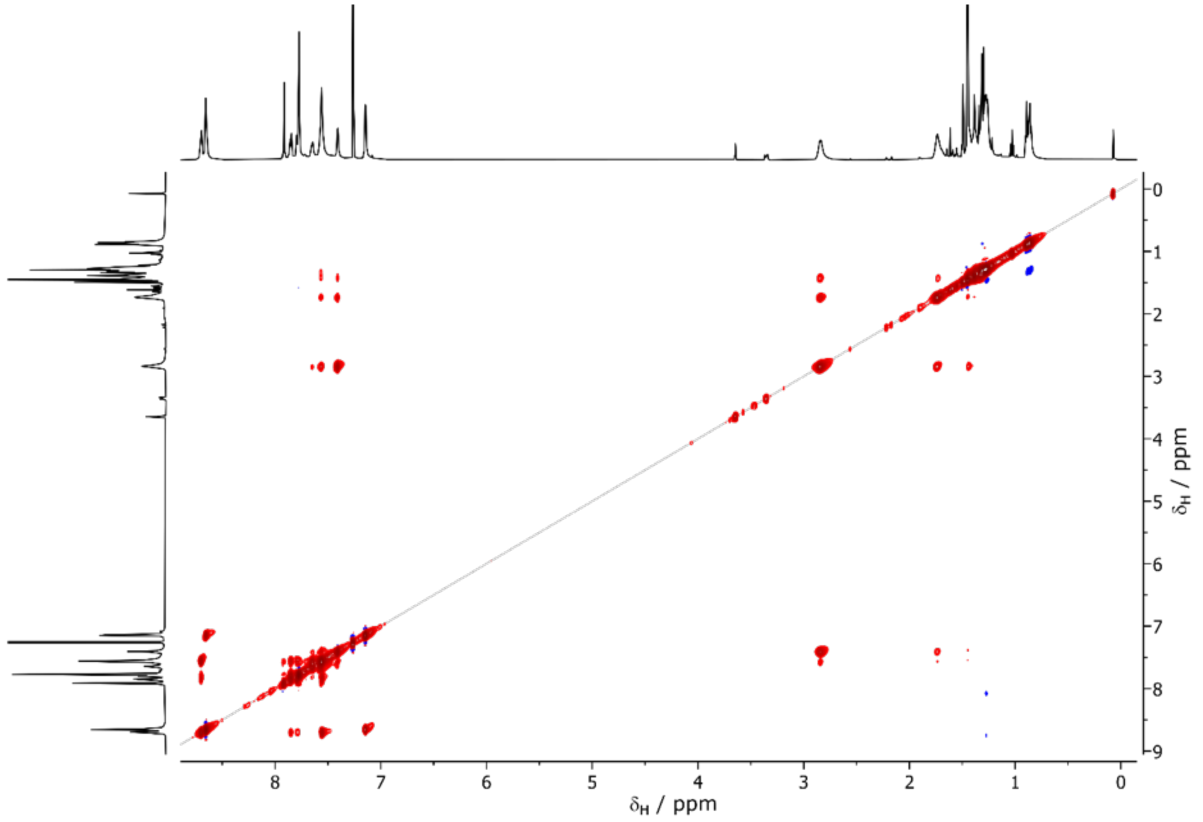

Figure S157. ^{1}H-^{1}H NOESY spectrum of **T18B** (600 MHz, $CDCl_3$, 298 K).

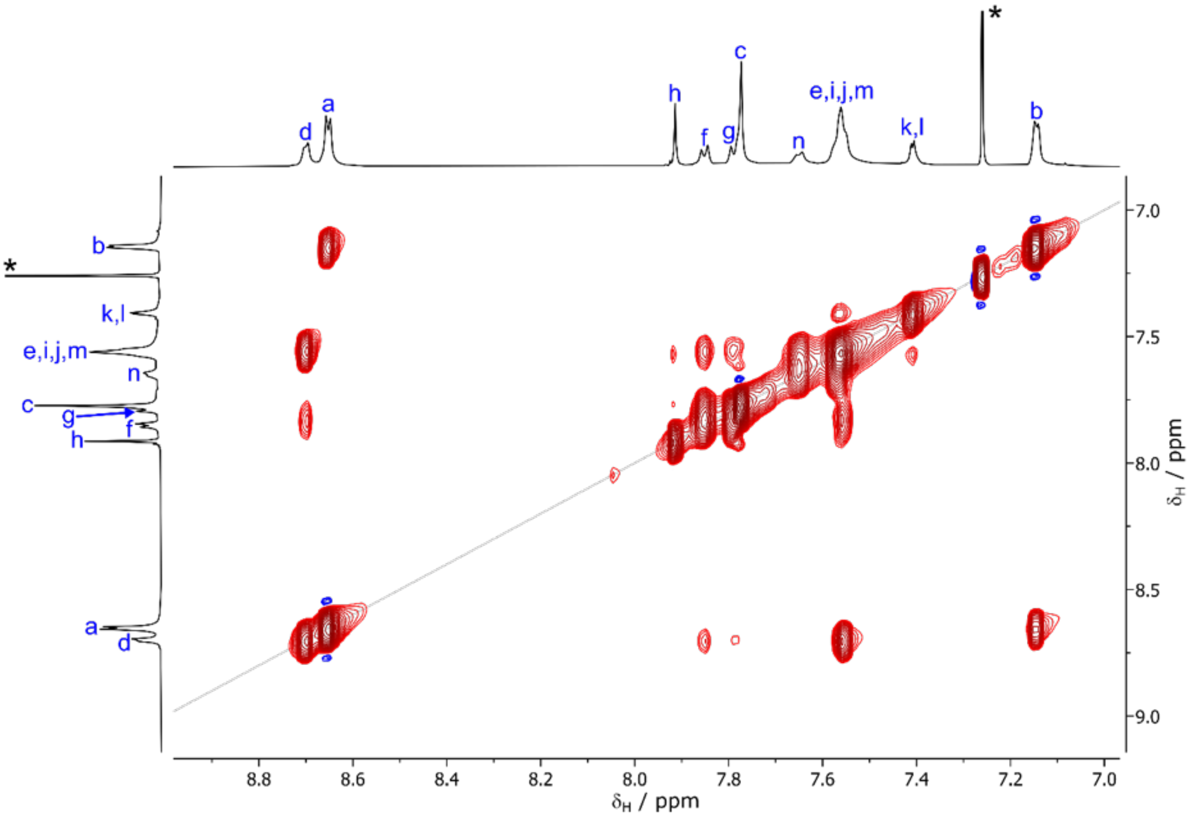

Figure S158. Aromatic region of the ^{1}H-^{1}H NOESY spectrum of **T18B** (600 MHz, $CDCl_3$, 298 K). * = $CHCl_3$

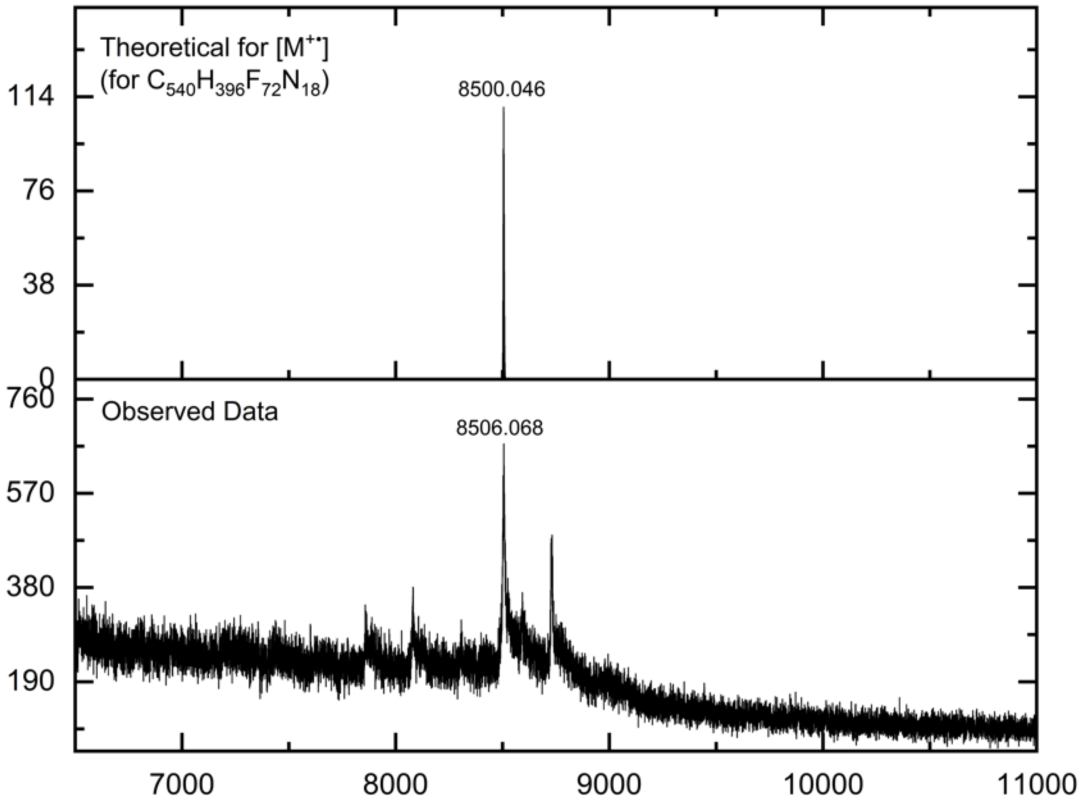

Figure S159. MALDI mass spectrum of **T18B** (matrix: dithranol)

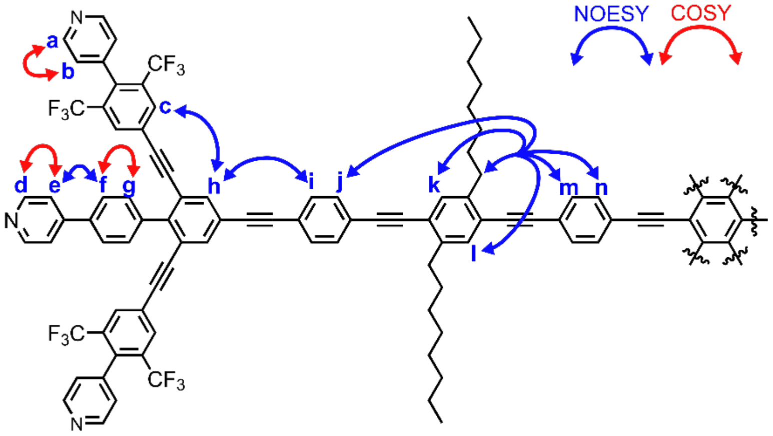

	a	b	c	d	e	f	g	h	i	j	k	l	m	n	Ar-CH2
a		s													
b	s														
c															
d					s										
e				s											
f				m	m		s								
g					w	s									
h			vw												
i								w							
j															
k										w					
l										w					
m											w	w		s	
n															
Ar-CH2										m	s	s	m	w	

Table S3. Summary of COSY (above diagonal) and NOESY (below diagonal) correlations in **T18$_B$**. vw; very weak; w: weak; mw: medium weak; m: medium; s: strong

Explanation of Assignment

- Protons a + d assigned by ppm value (protons α to nitrogen are very deshielded) and by integration intensity.
- b has COSY to a.
- k and l only have strongest NOESY of all protons to Ar-CH_2.
- Singlet at 7.91 can only be h.
- f has NOESY to e and d.
- g has NOESY to f.
- Peak at 7.78 ppm has integral of 36 – but already contains g – so 24 protons remain (looks like a singlet too) This can only be proton c. c also has NOESY to h.
- Two peaks remain, one doublet (12 protons) and one multiplet (48 protons)
- e must be in the multiplet as it is a singlet.
- Doublet at 7.65 is n:
    - Multiplet has a stronger NOESY to Ar-CH_2 (from protons m and j) so expect doublet to be n or i
    - h has weak NOESY to the multiplet and no NOESY to the doublet, so i must be in the multiplet.
    - Therefore the doublet comes from proton n.
- Multiplet is e,i,j,m

Spectra of linear porphyrin oligomers (Ar = OOct)

P3$_{OOct}$

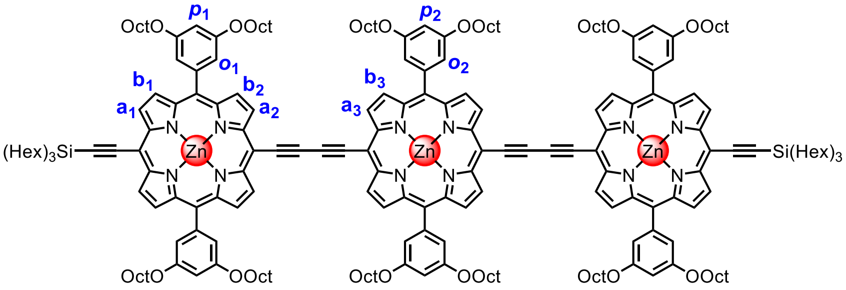

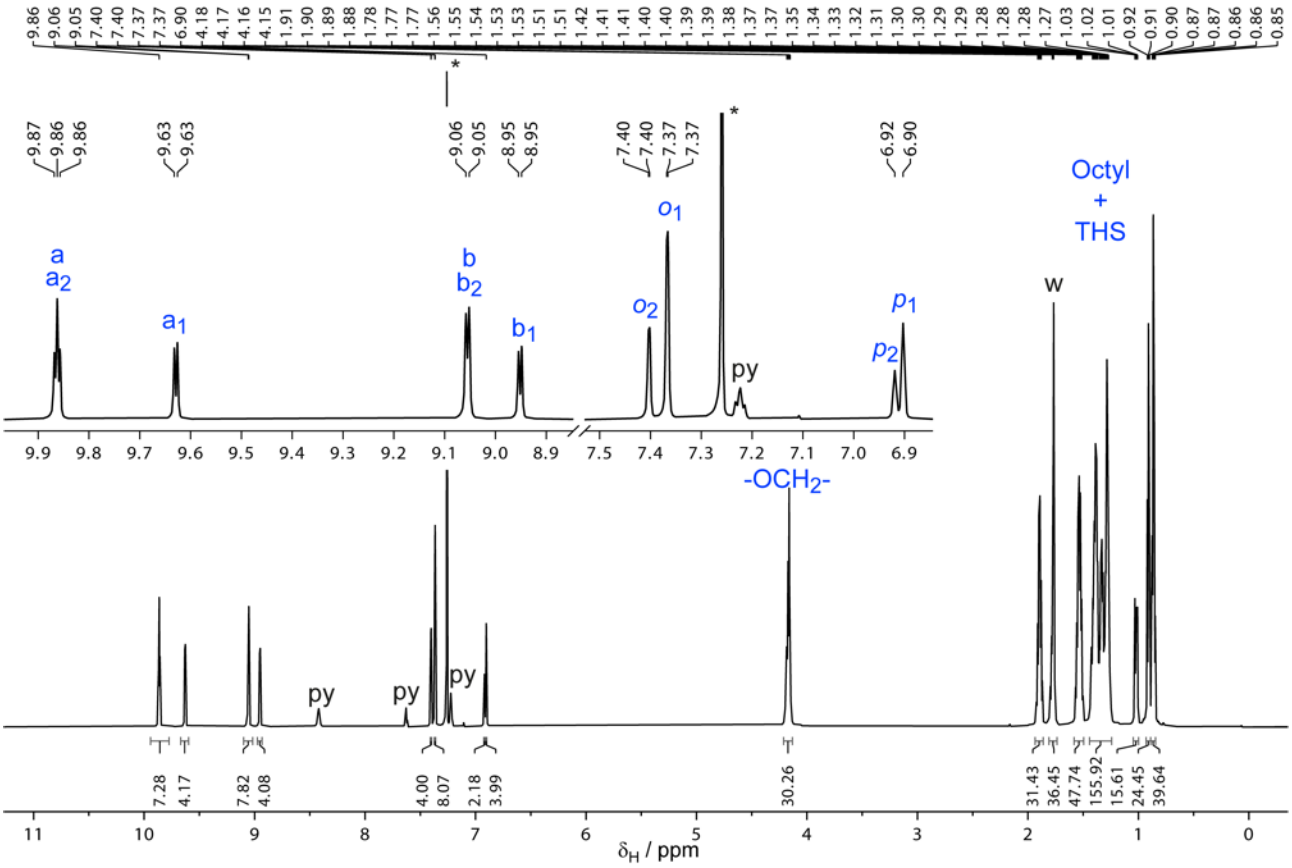

Figure S160. ^{1}H NMR spectrum of **P3$_{OOct}$** (700 MHz, $CDCl_3$, d_5-pyridine, 298 K). * = $CHCl_3$; py = pyridine; w = H_2O.

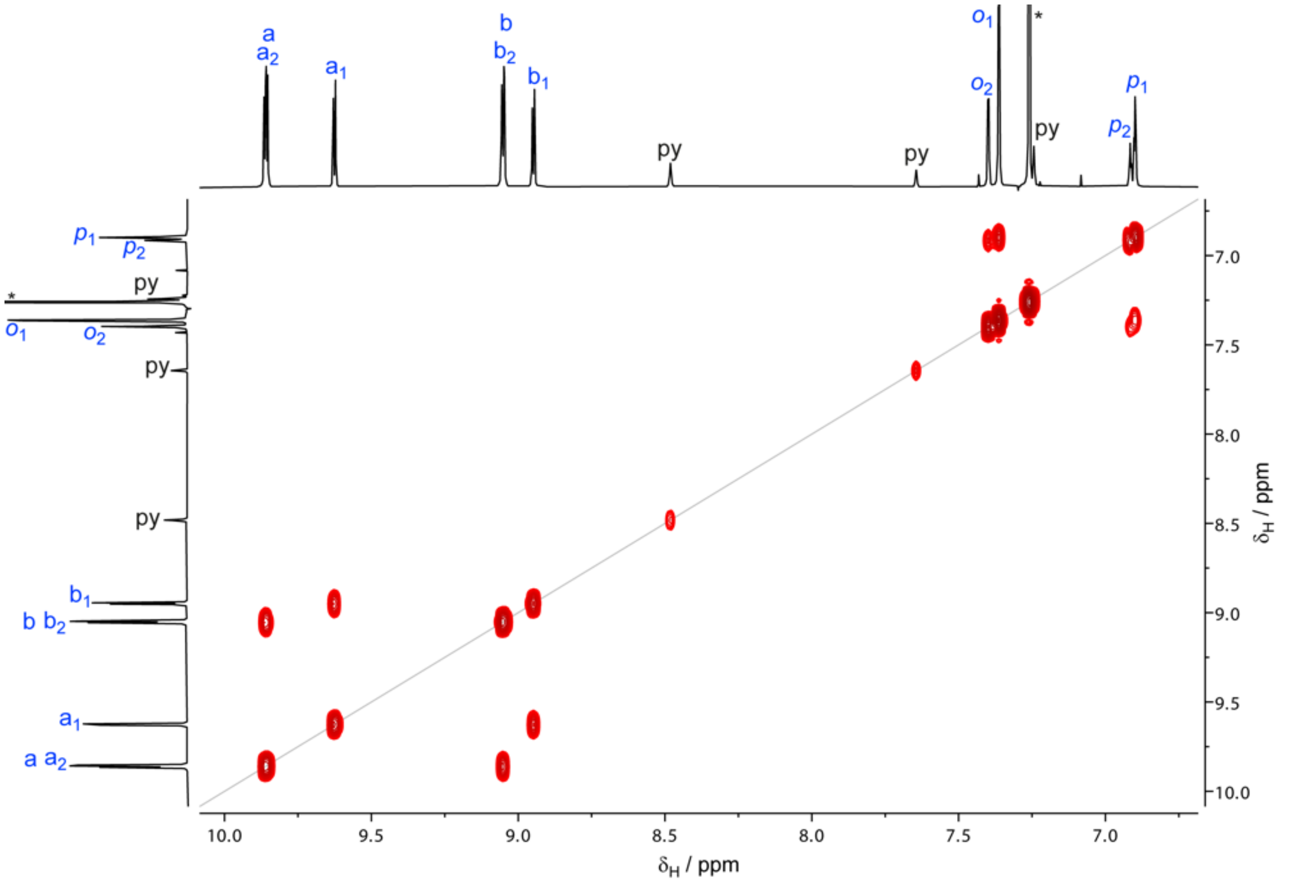

Figure S161. ^{1}H-^{1}H COSY spectrum (aromatic region) of **P3**$_{\mathbf{OOct}}$ (600 MHz, $CDCl_3$, d_5-pyridine, 298 K). * = $CHCl_3$; py = pyridine.

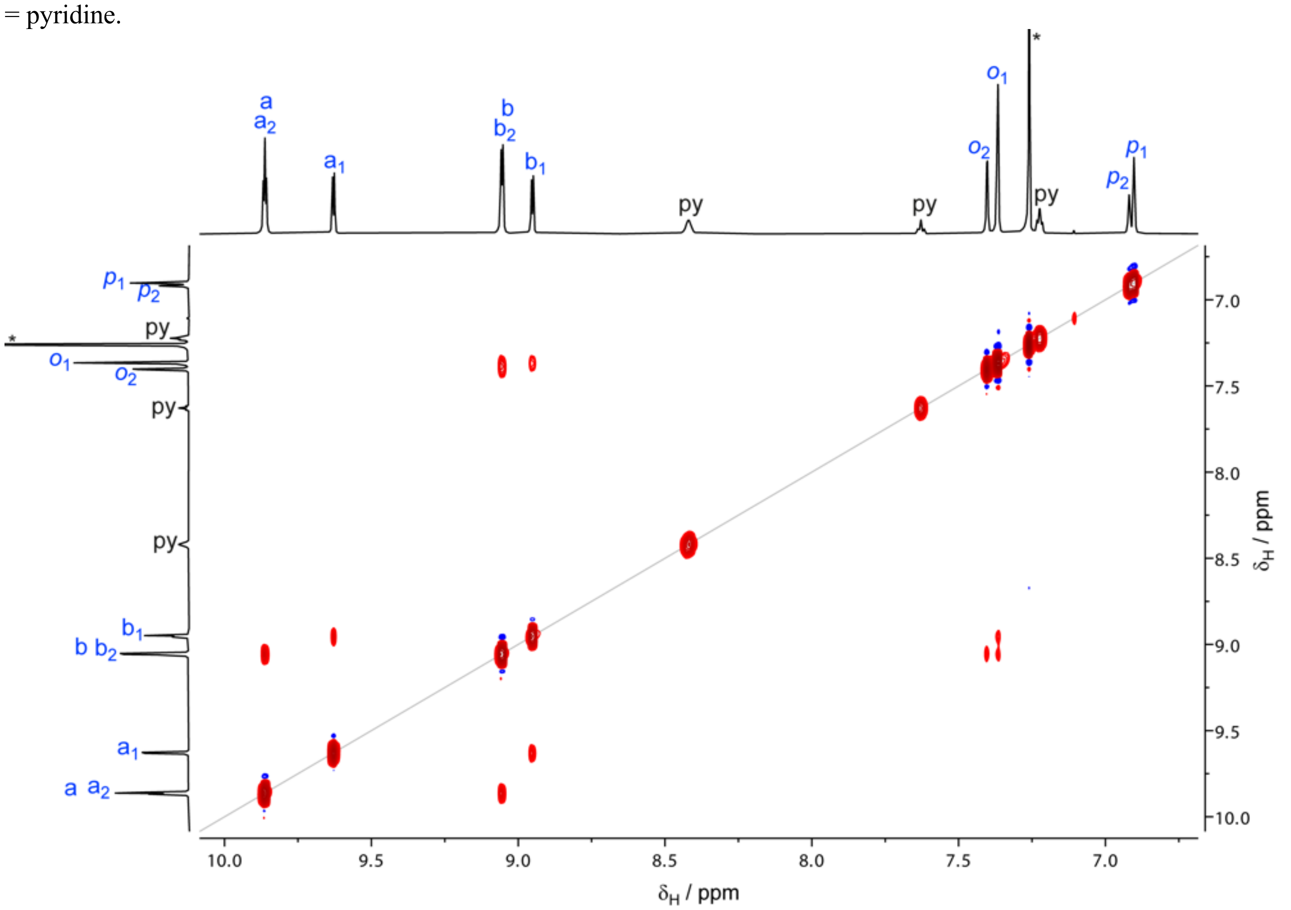

Figure S162. ^{1}H-^{1}H NOESY spectrum (aromatic region) of **P3**$_{\mathbf{OOct}}$ (700 MHz, $CDCl_3$, d_5-pyridine, 298 K). * = $CHCl_3$; py = pyridine.

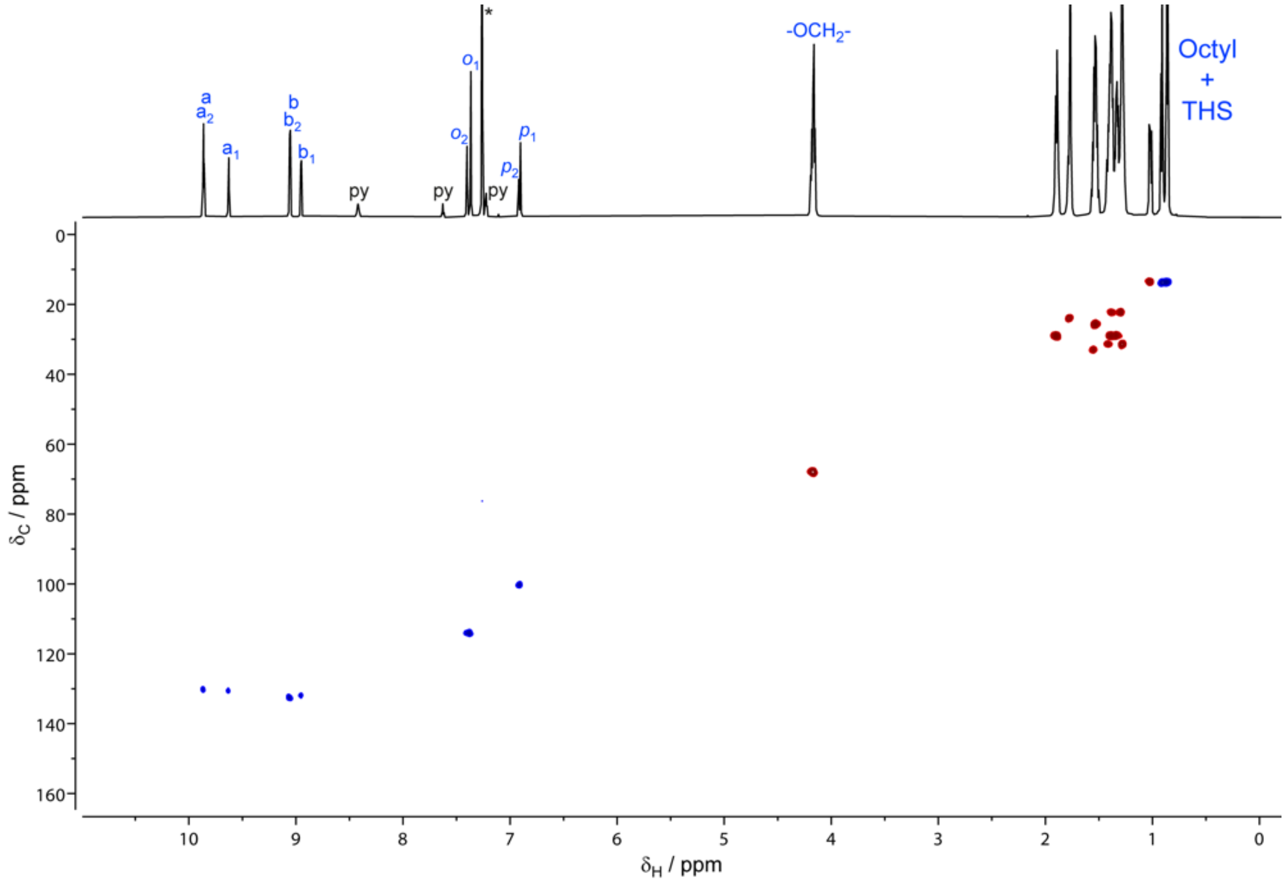

Figure S163. ^{1}H-^{13}C HSQC spectrum of **P3$_{OOct}$** (700 MHz, $CDCl_3$, d_5-pyridine, 298 K). * = $CHCl_3$; py = pyridine; w = H_2O.

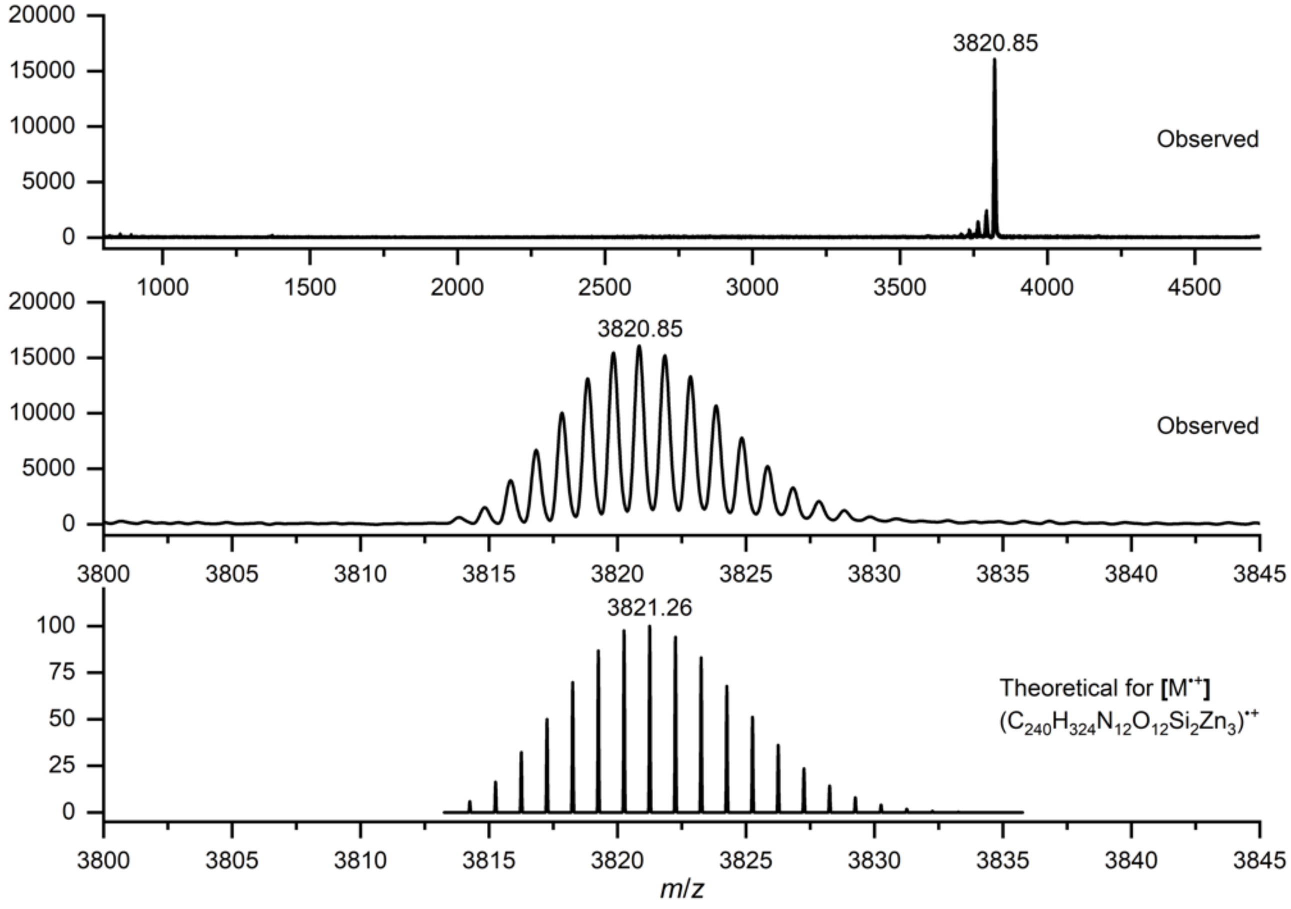

Figure S164. MALDI mass spectrum of **P3$_{OOct}$**.

$P6_{OOct}$

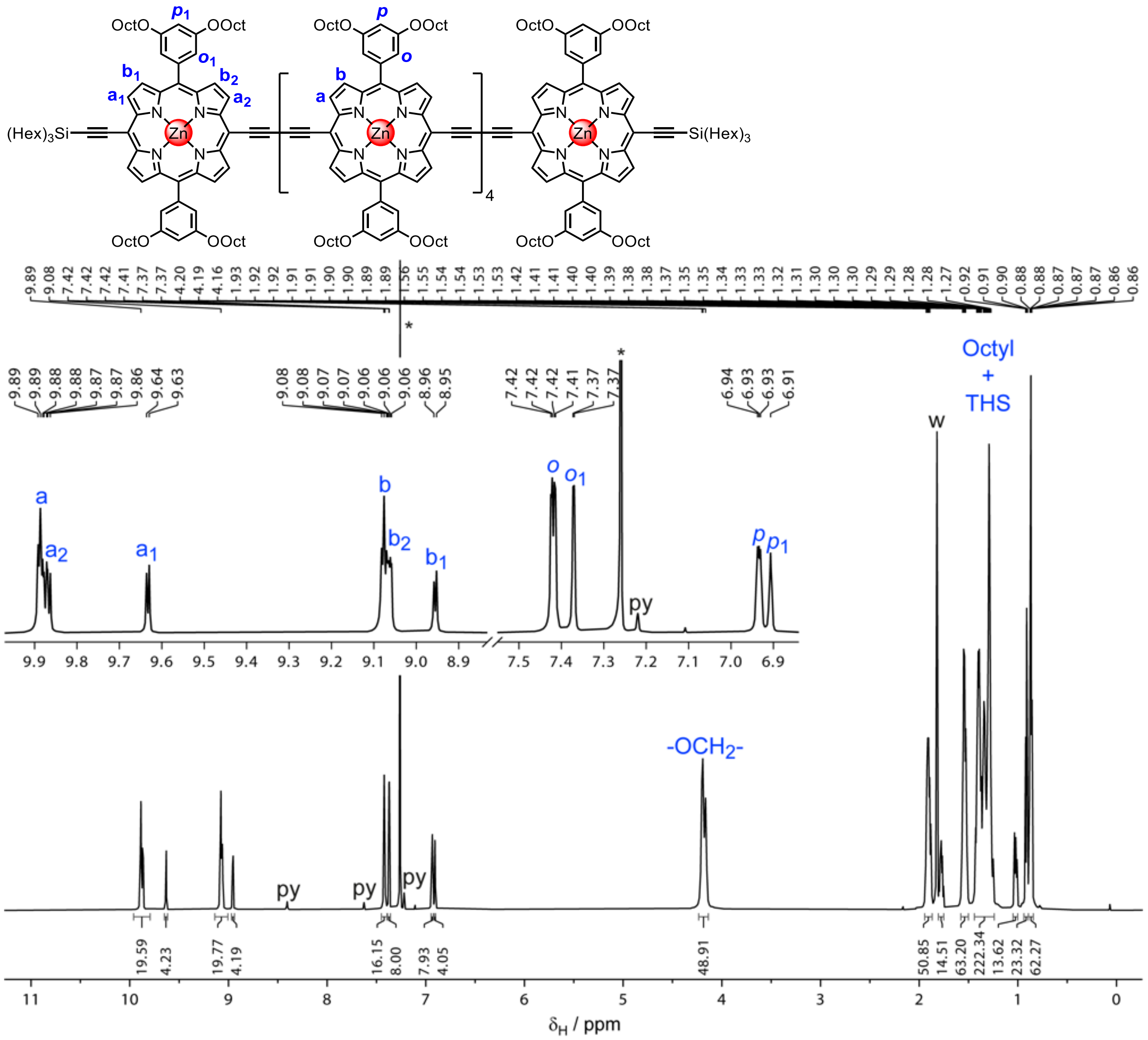

Figure S165. ^{1}H NMR spectrum of **$P6_{OOct}$** (700 MHz, $CDCl_3$, *d*5-pyridine, 298 K). * = $CHCl_3$; py = pyridine; w = H_2O.

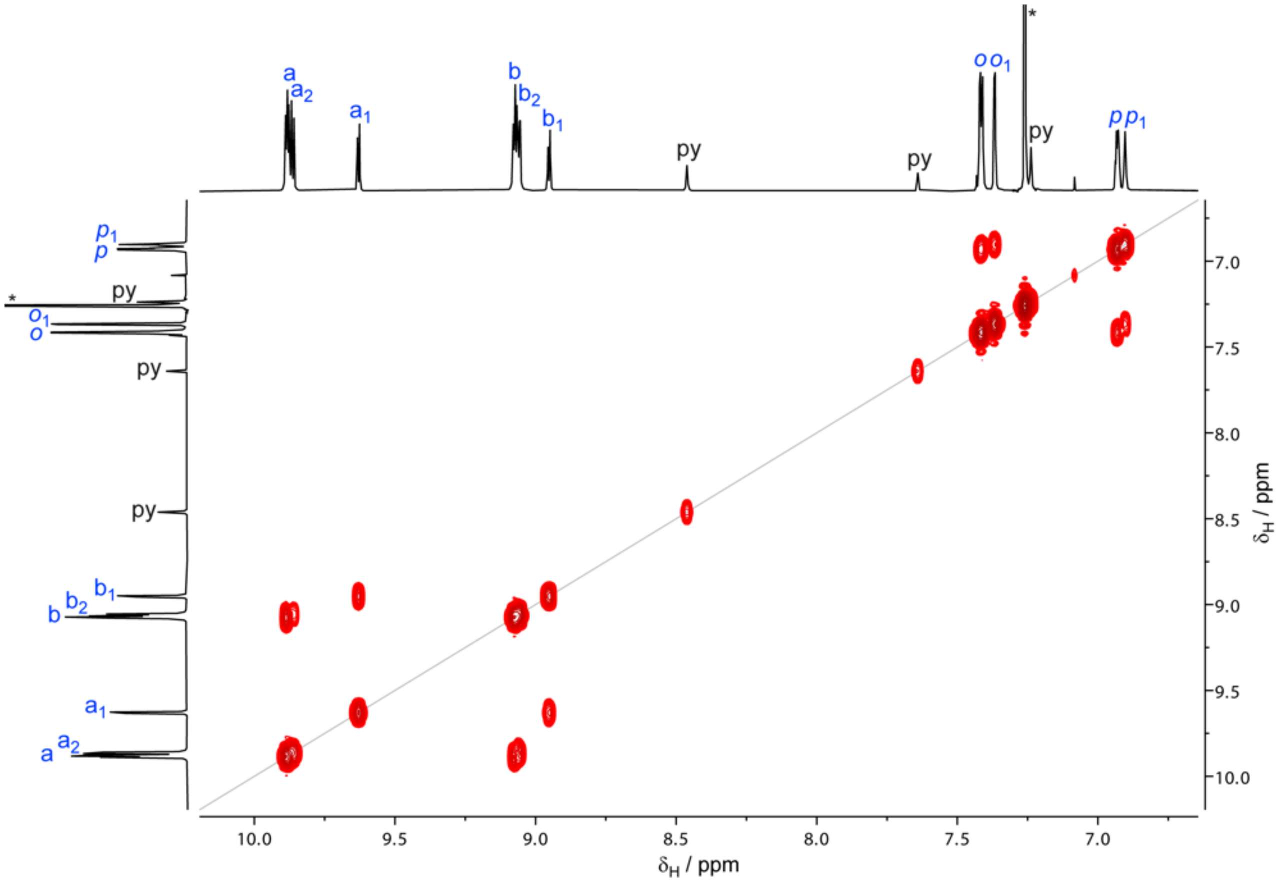

Figure S166. ^{1}H-^{1}H COSY spectrum (aromatic region) of **P6$_{OOct}$** (600 MHz, $CDCl_3$, d_5-pyridine, 298 K). * = $CHCl_3$; py = pyridine.

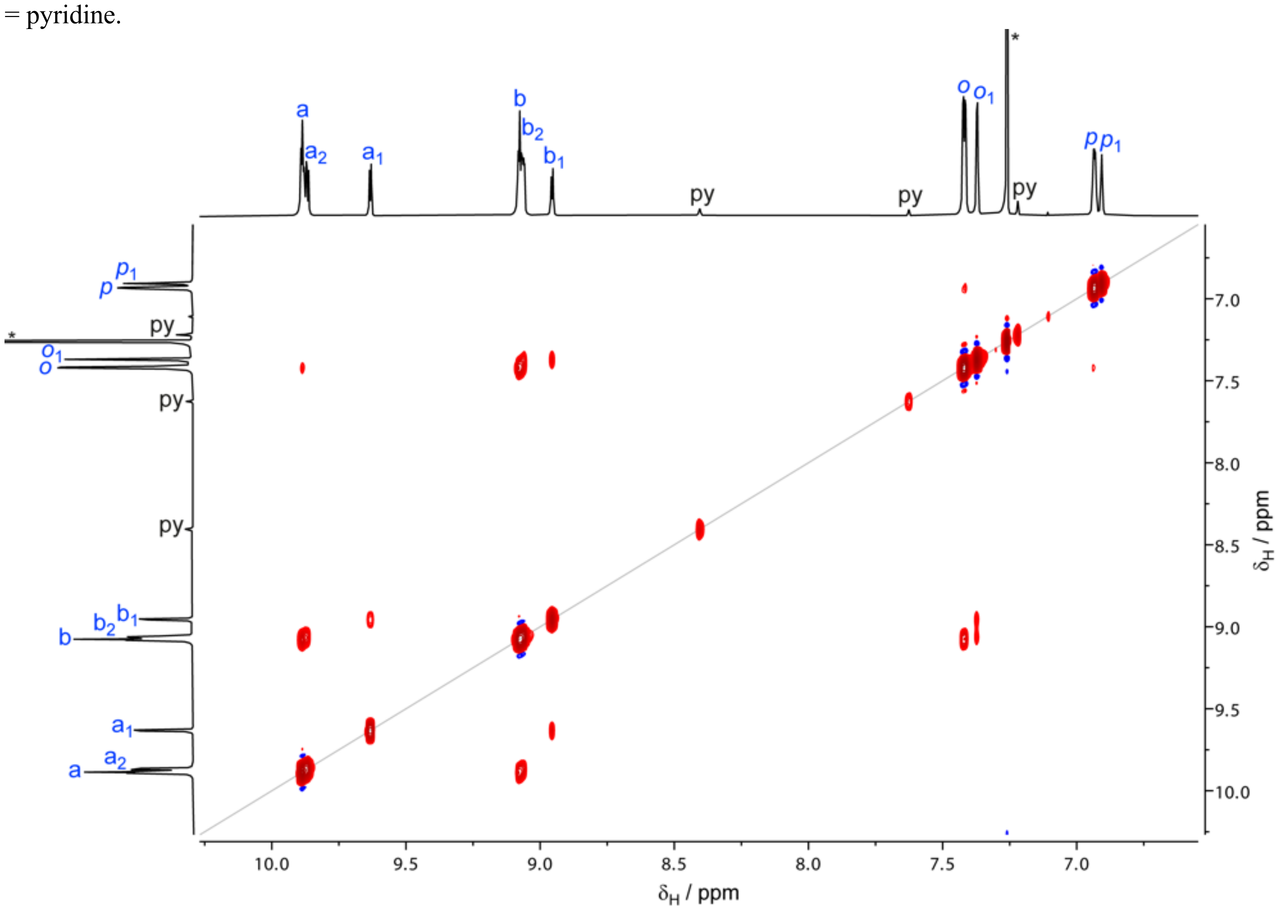

Figure S167. ^{1}H-^{1}H NOESY spectrum (aromatic region) of **P6$_{OOct}$** (700 MHz, $CDCl_3$, d_5-pyridine, 298 K). * = $CHCl_3$; py = pyridine.

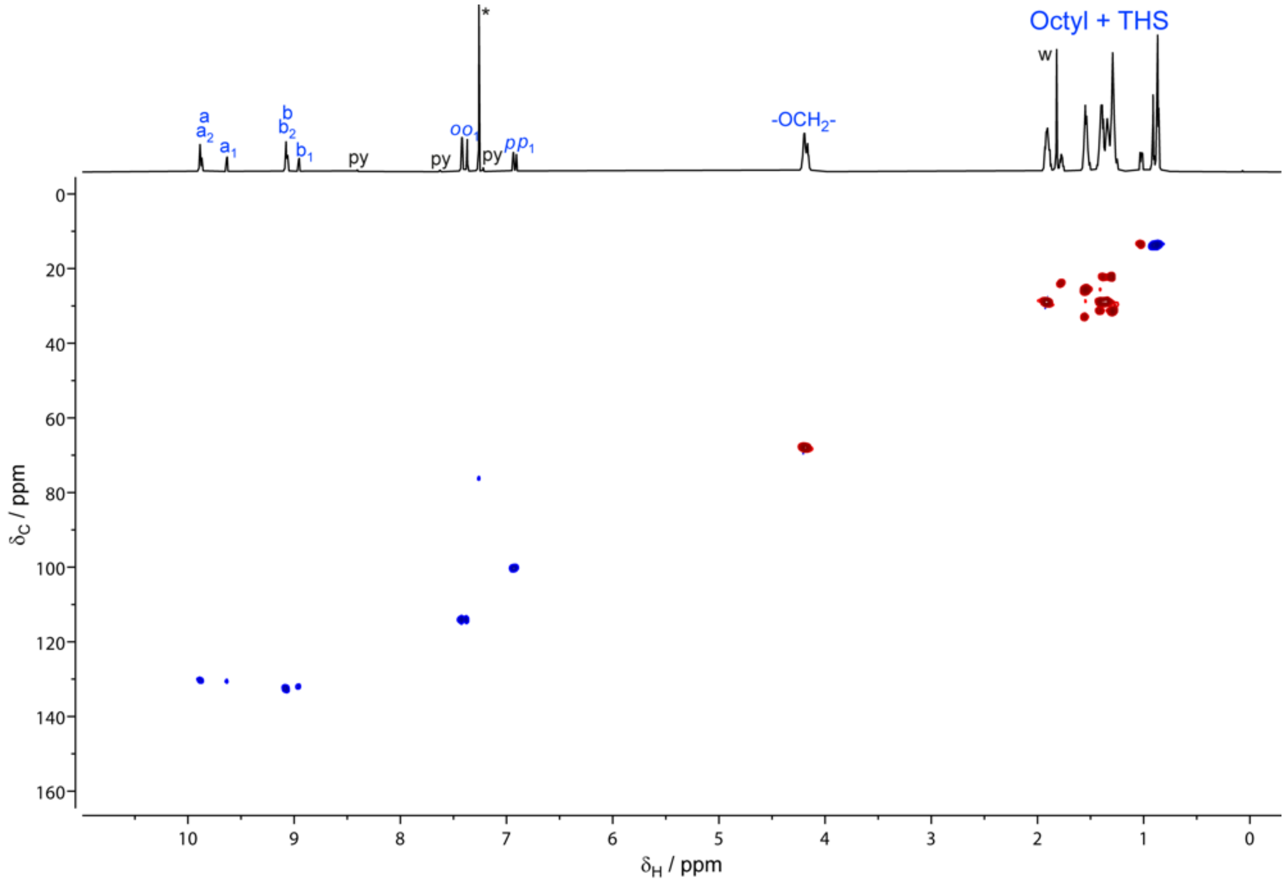

Figure S168. ^{1}H-^{13}C HSQC spectrum of **P6$_{\mathbf{OOct}}$** (700 MHz, $CDCl_3$, d_5-pyridine, 298 K). * = $CHCl_3$; py = pyridine; w = H_2O.

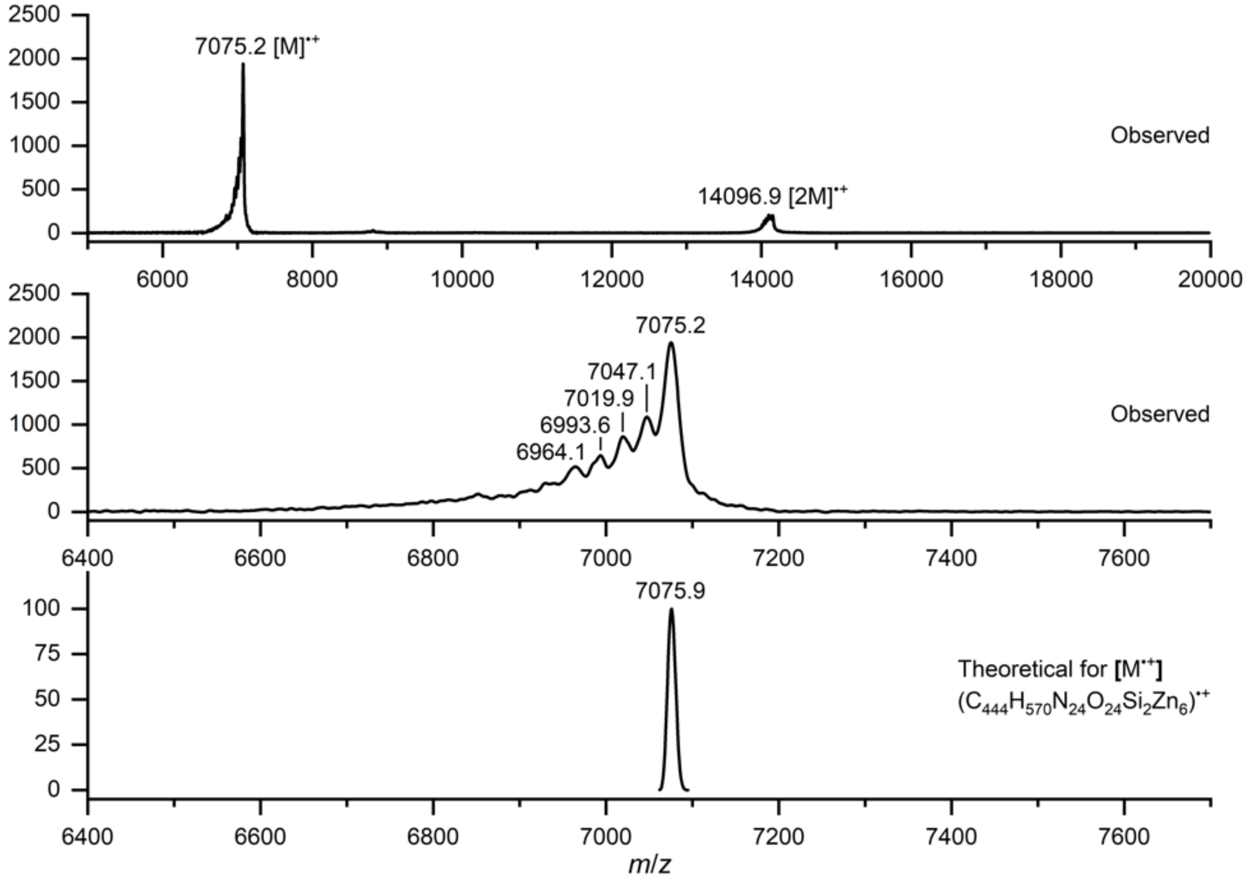

Figure S169. MALDI mass spectrum of **P6$_{\mathbf{OOct}}$**.

P9$_{OOct}$

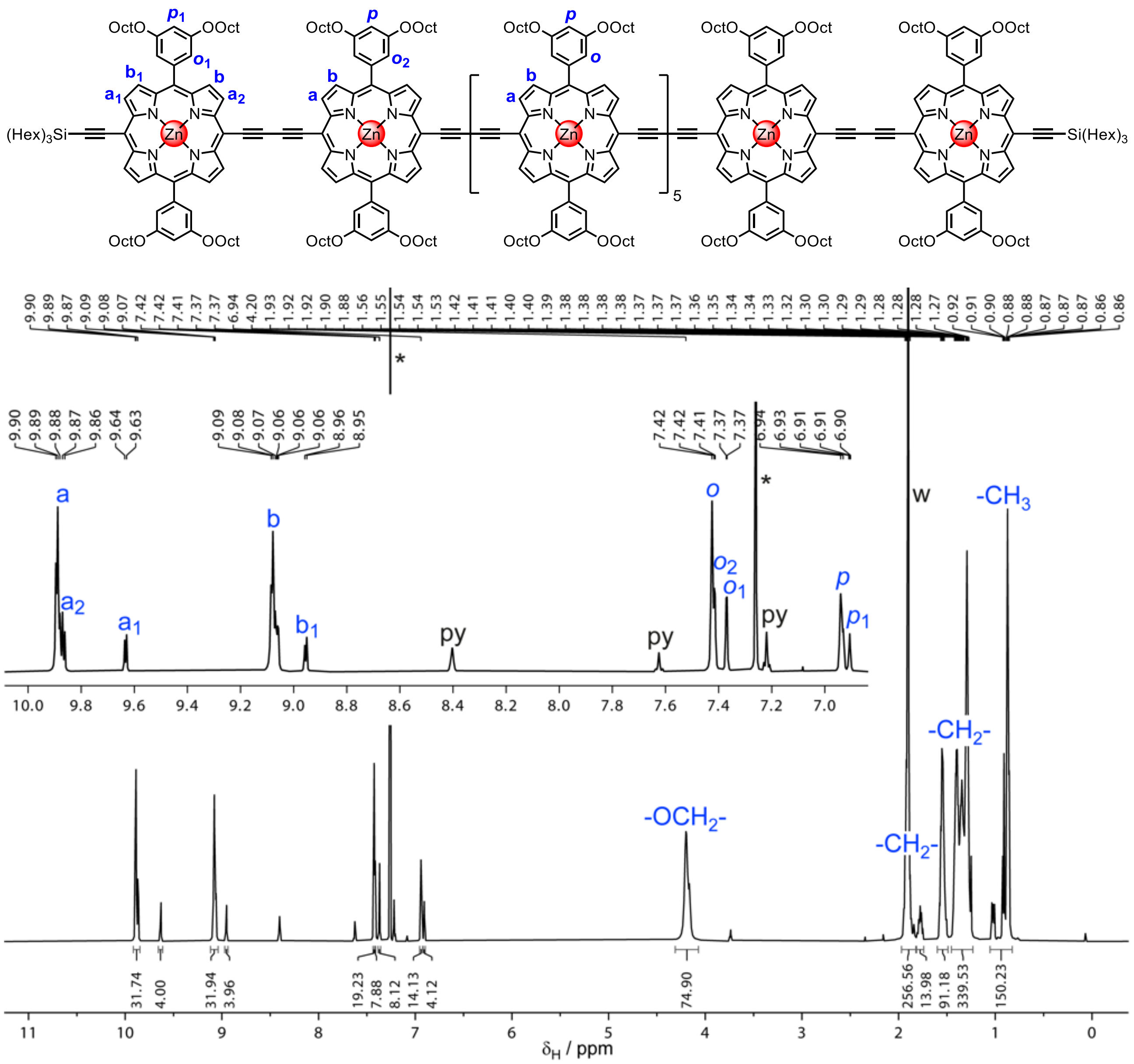

Figure S170. ^{1}H NMR spectrum of **P9$_{OOct}$** (500 MHz, $CDCl_3$, d_5-pyridine, 298 K). * = $CHCl_3$; py = pyridine; w = H_2O.

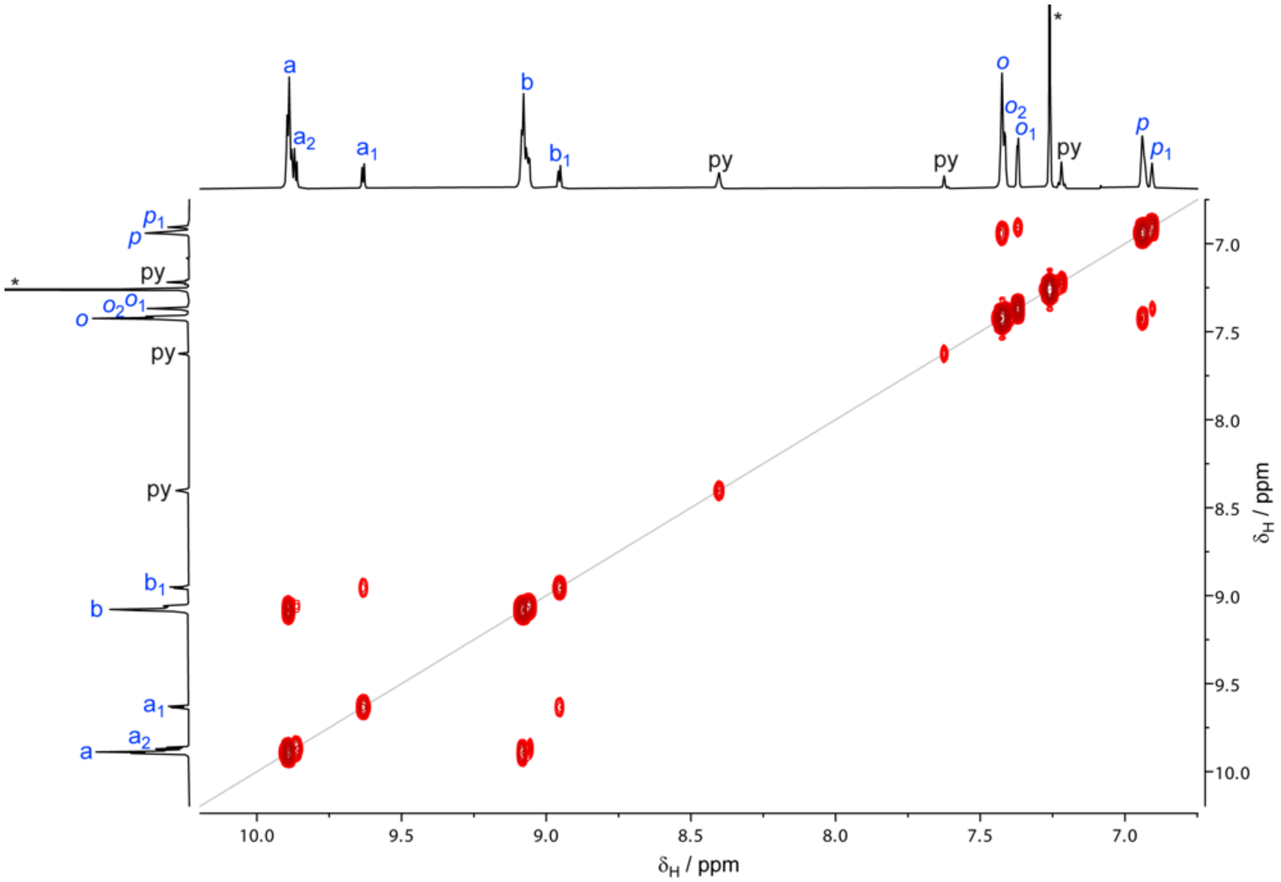

Figure S171. ^{1}H-^{1}H COSY spectrum (aromatic region) of **P9**$_{\mathbf{OOct}}$ (500 MHz, $CDCl_3$, *d*$_5$-pyridine, 298 K). * = $CHCl_3$; py = pyridine.

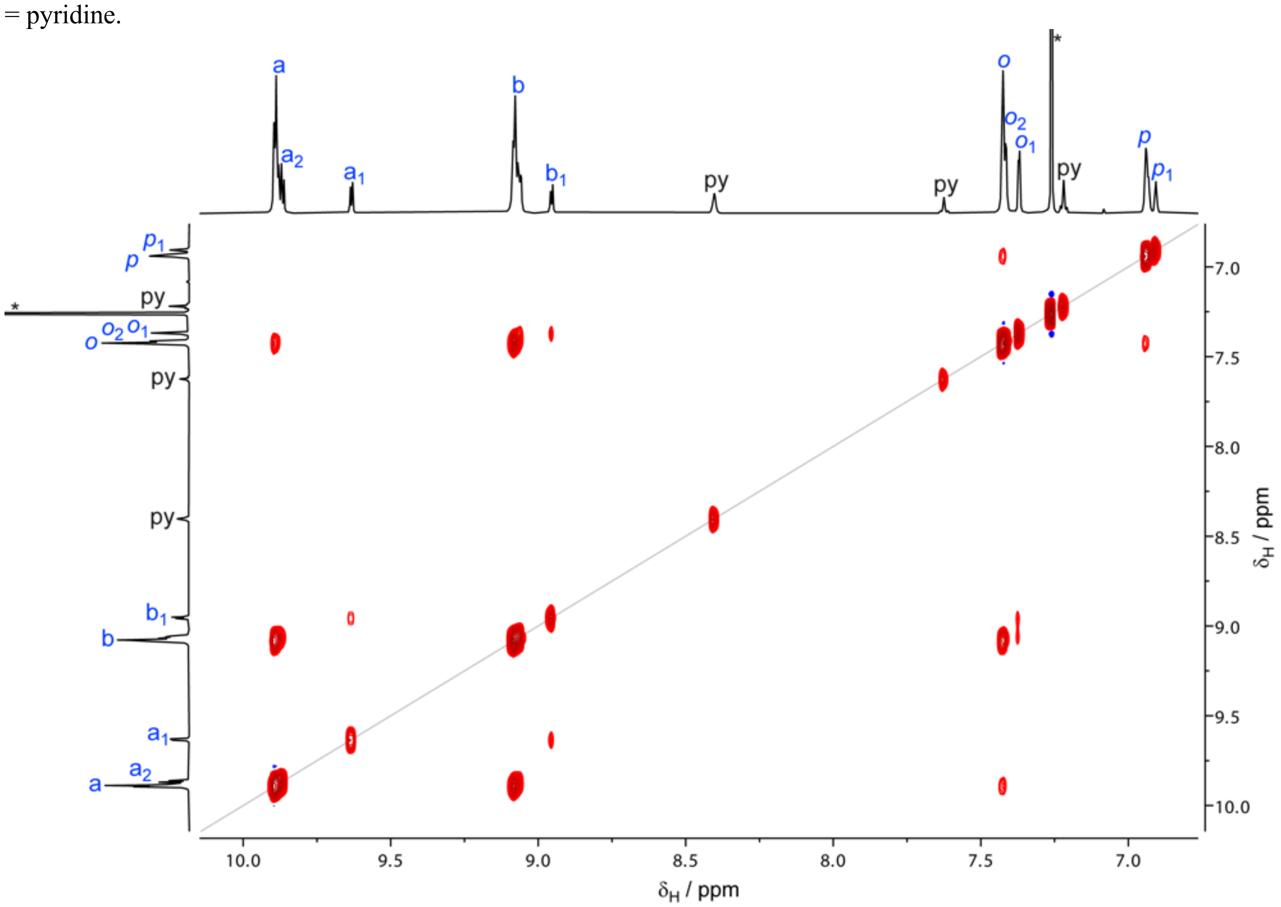

Figure S172. ^{1}H-^{1}H NOESY spectrum (aromatic region) of **P9**$_{\mathbf{OOct}}$ (500 MHz, $CDCl_3$, *d*$_5$-pyridine, 298 K). * = $CHCl_3$; py = pyridine.

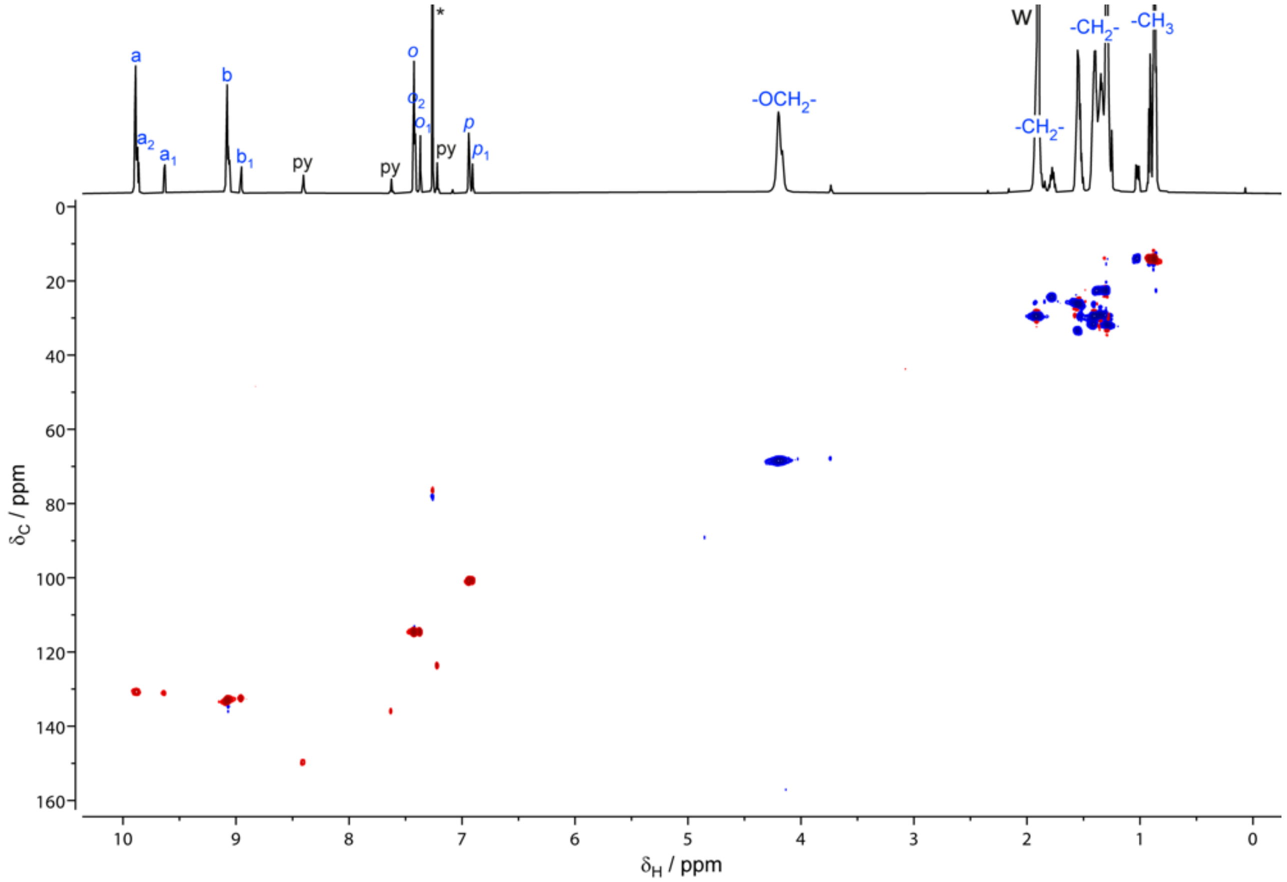

Figure S173. ^{1}H-^{13}C HSQC spectrum of **P9$_{\mathbf{OOct}}$** (500 MHz, $CDCl_3$, d_5-pyridine, 298 K). * = $CHCl_3$; py = pyridine; w = H_2O.

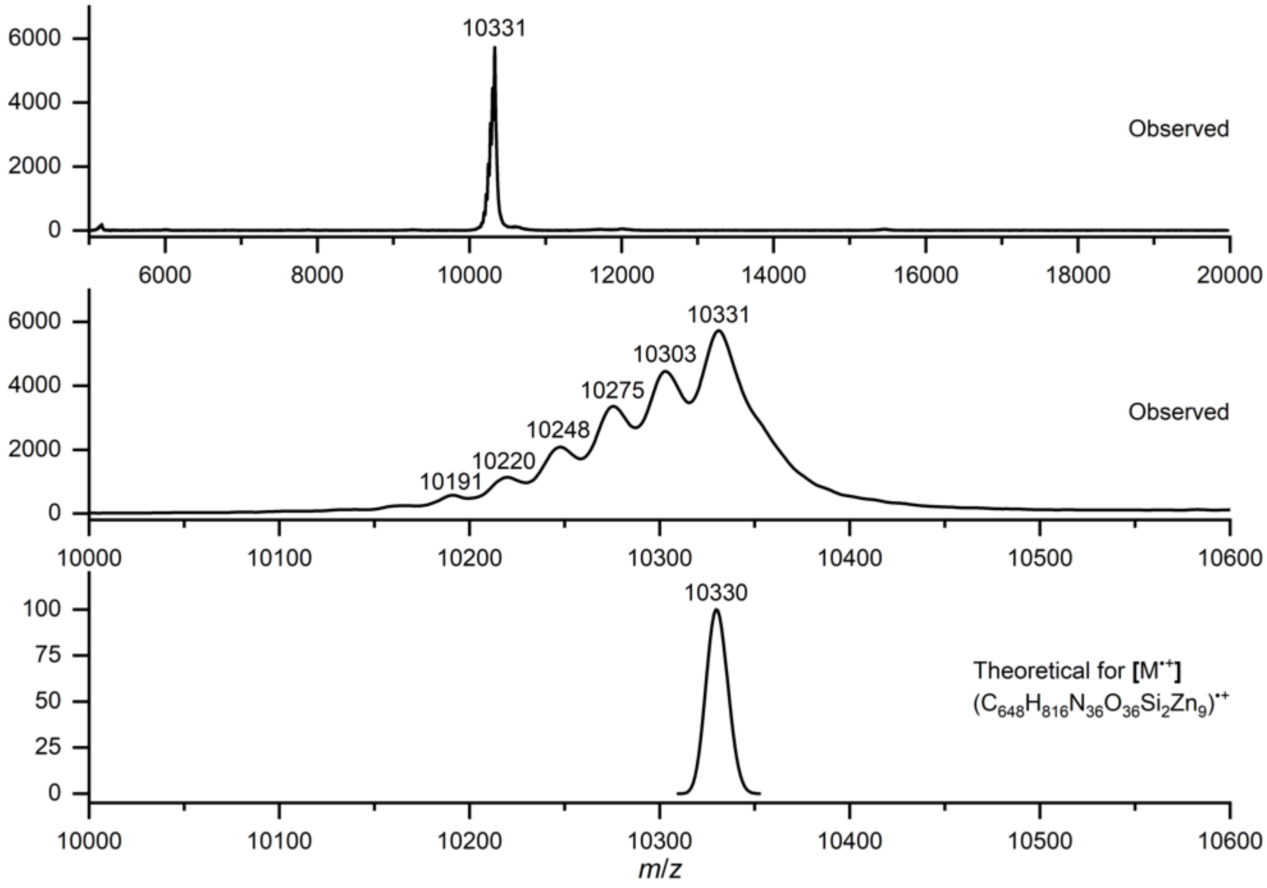

Figure S174. MALDI mass spectrum of **P9$_{\mathbf{OOct}}$**.

$P12_{OOct}$

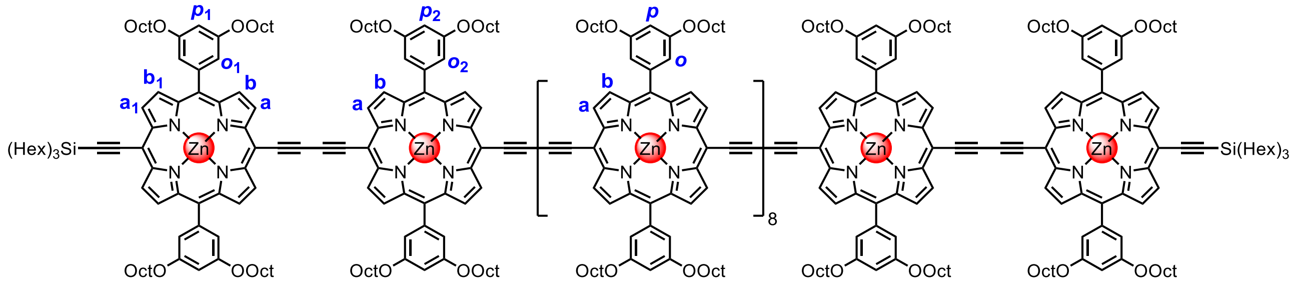

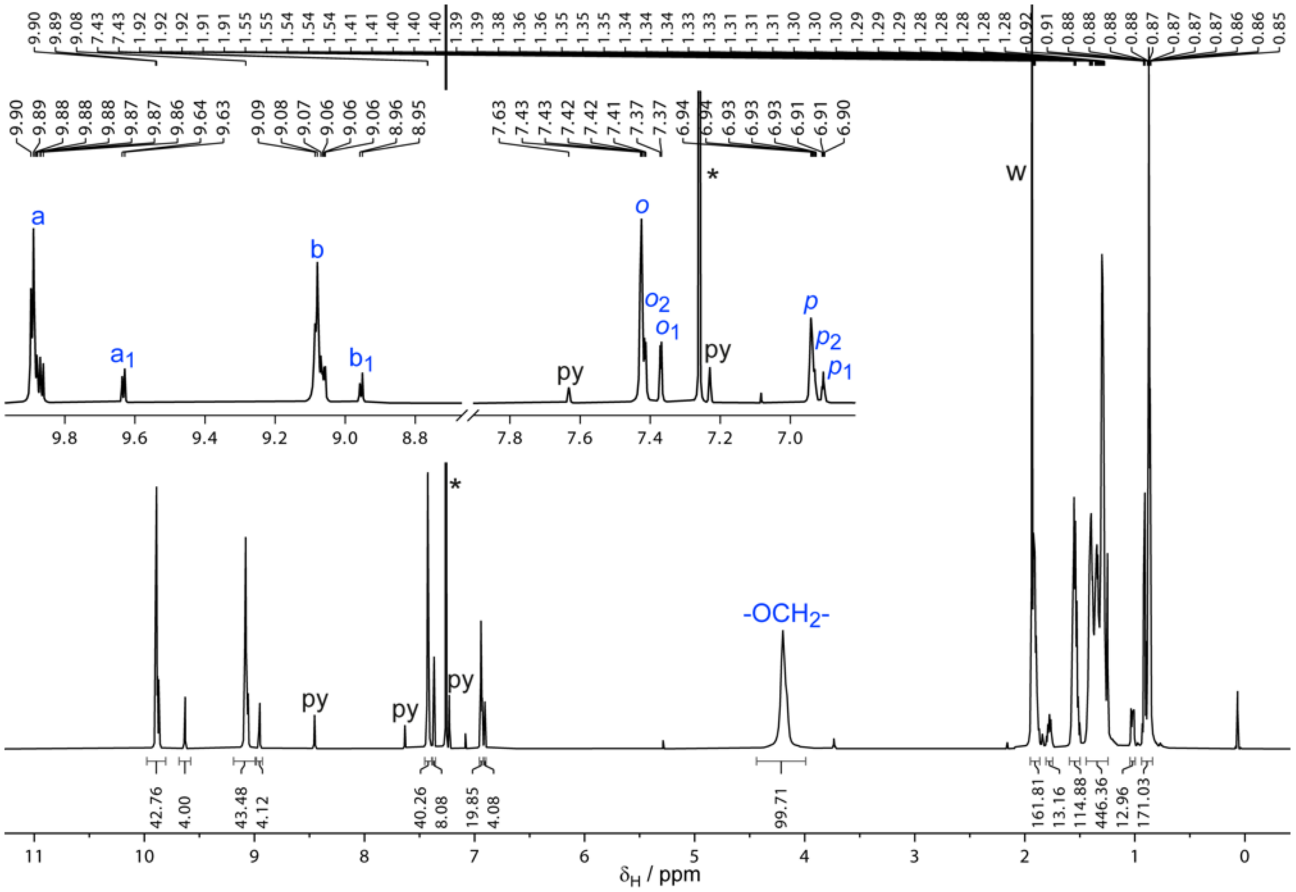

Figure S175. ^{1}H NMR spectrum of **$P12_{OOct}$** (600 MHz, $CDCl_3$, d_5-pyridine, 298 K). * = $CHCl_3$; py = pyridine; w = H_2O.

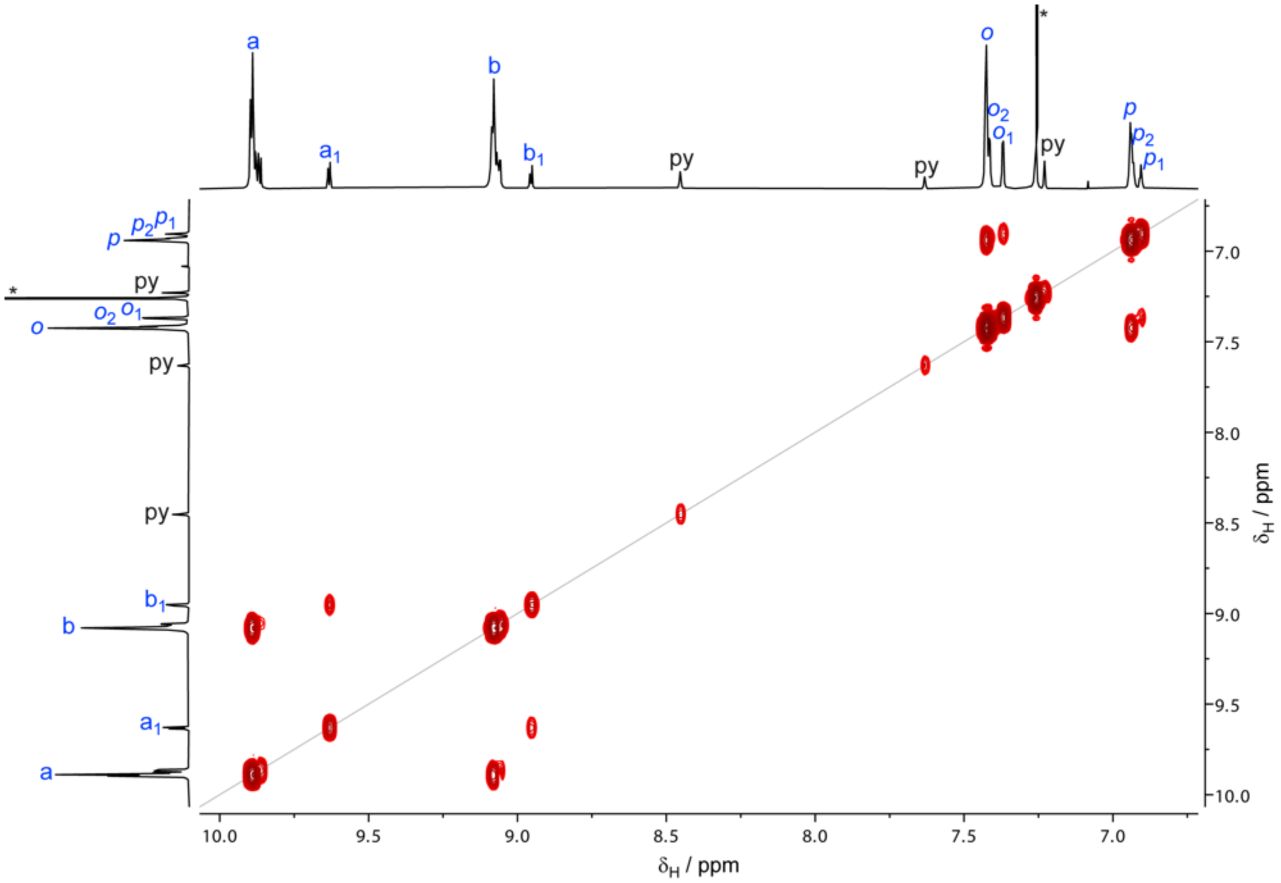

Figure S176. ^{1}H-^{1}H COSY spectrum (aromatic region) of **P12$_{OOct}$** (600 MHz, $CDCl_3$, d_5-pyridine, 298 K). * = $CHCl_3$; py = pyridine.

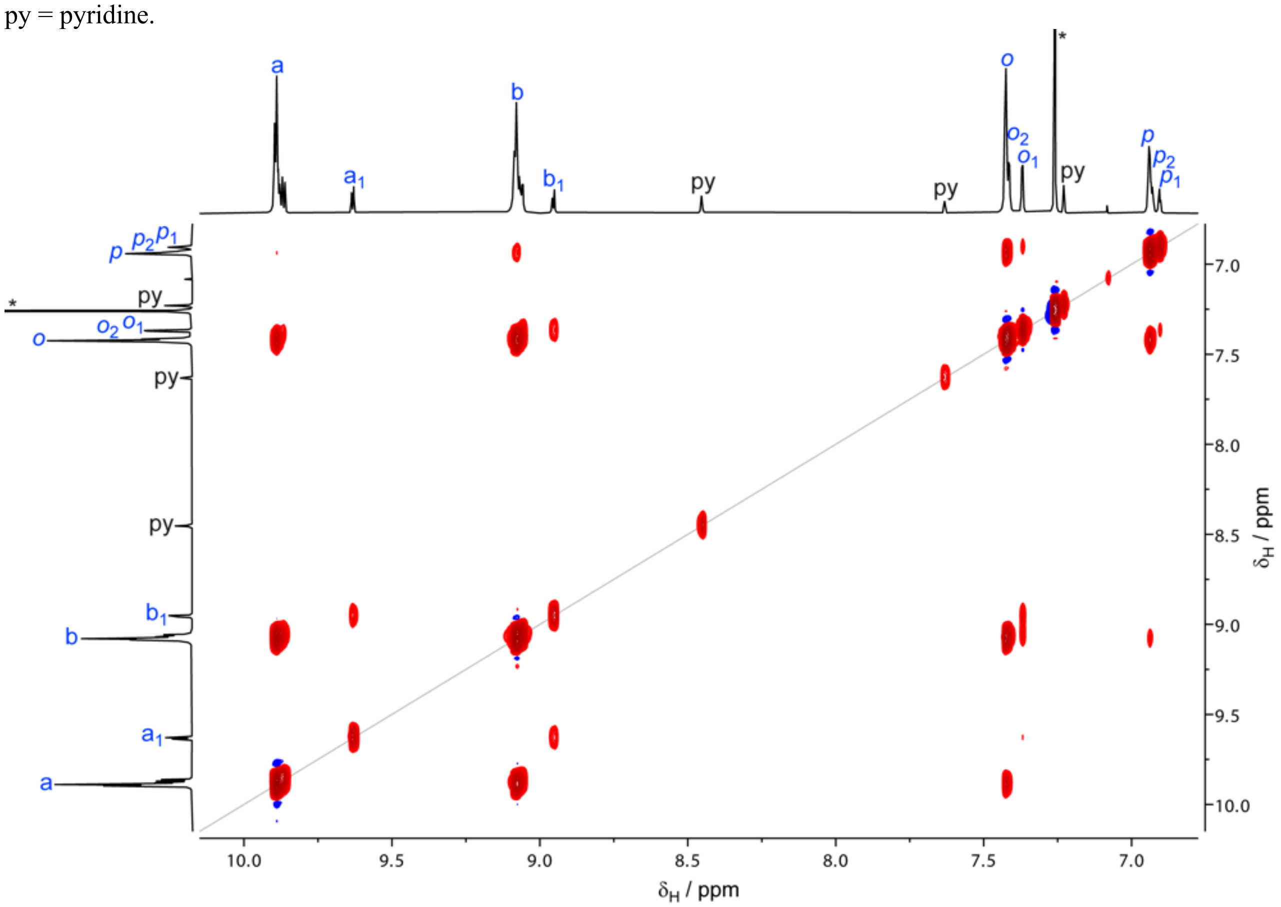

Figure S177. ^{1}H-^{1}H NOESY spectrum (aromatic region) of **P12$_{OOct}$** (600 MHz, $CDCl_3$, d_5-pyridine, 298 K). * = $CHCl_3$; py = pyridine.

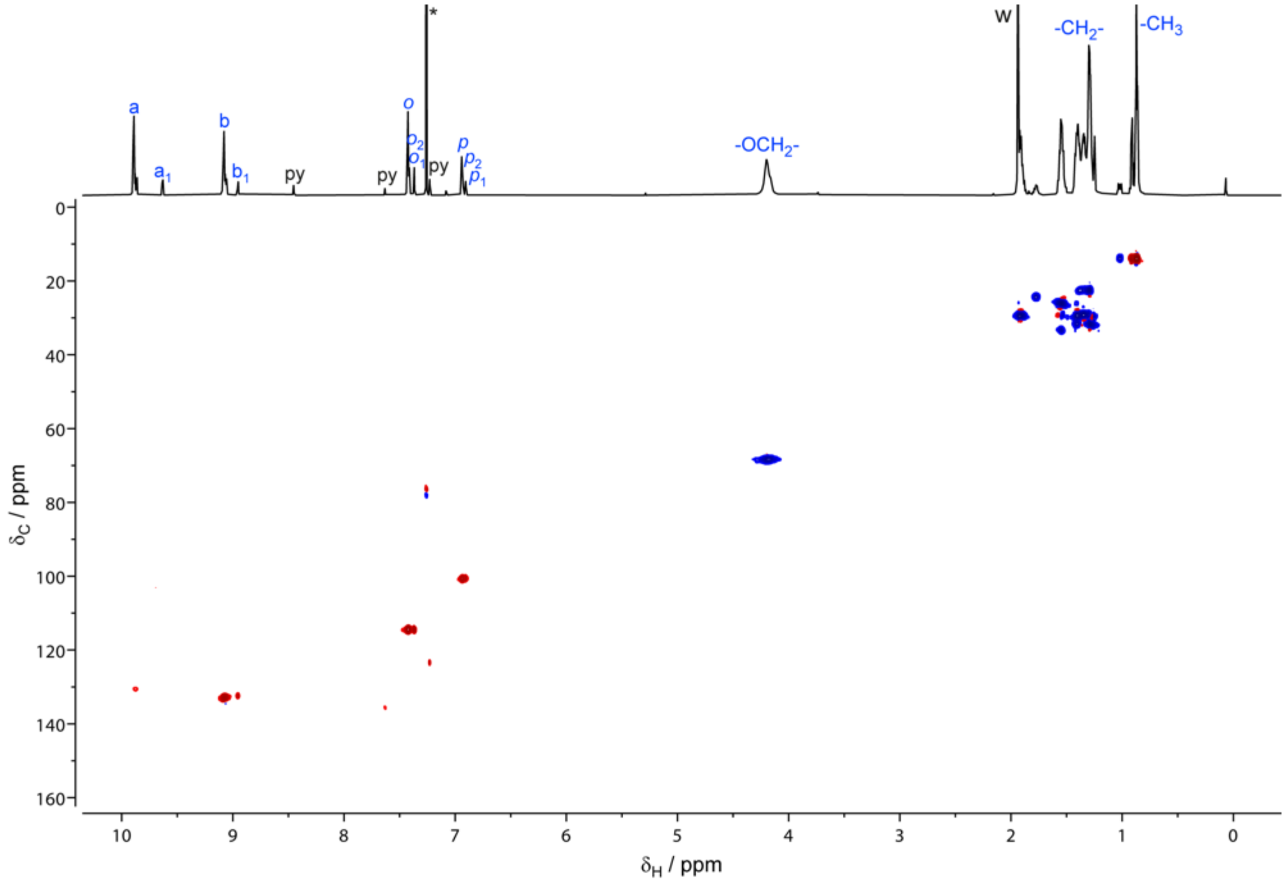

Figure S178. ^{1}H-^{13}C HSQC spectrum of **P12$_{OOct}$** (600 MHz, $CDCl_3$, *d*$_5$-pyridine, 298 K). * = $CHCl_3$; py = pyridine; w = H_2O.

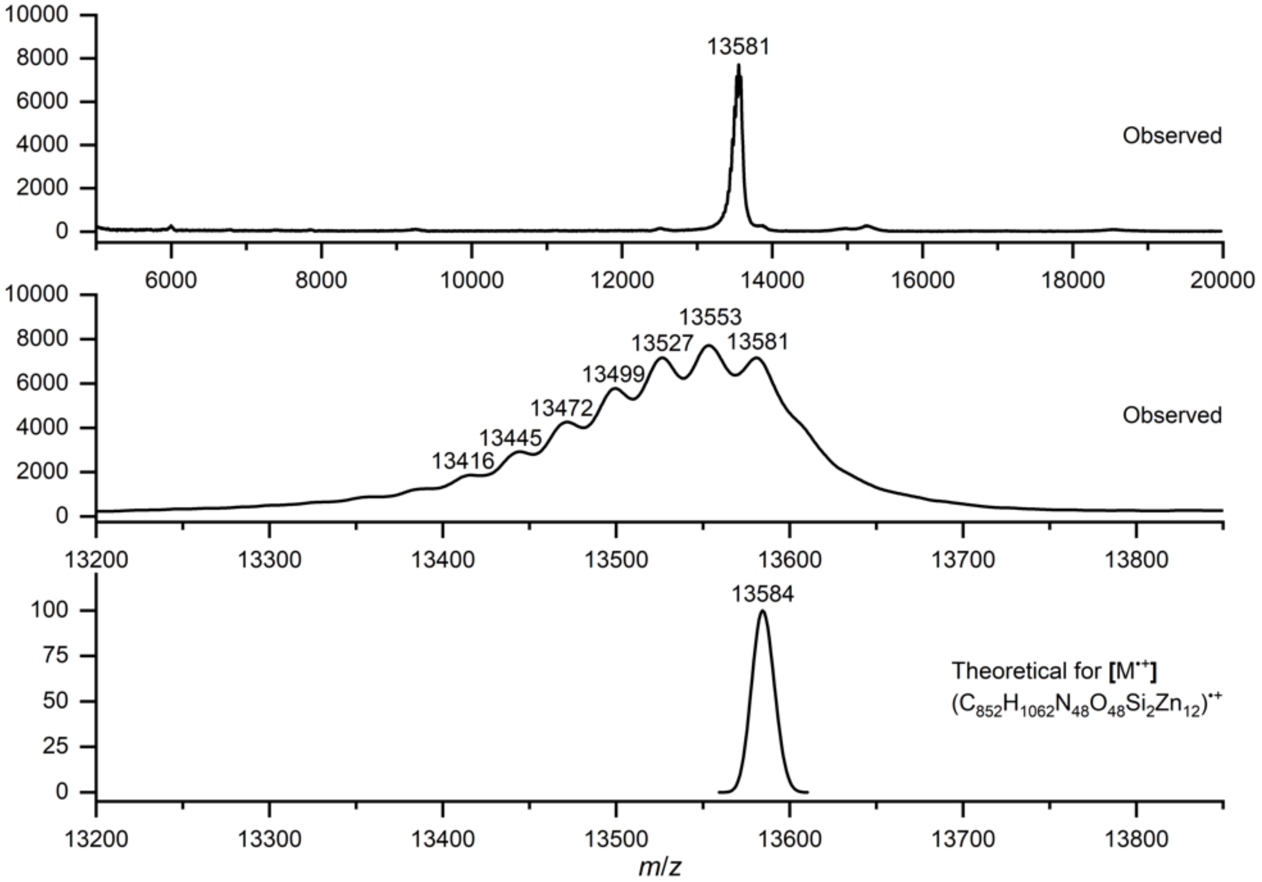

Figure S179. MALDI mass spectrum of **P12$_{OOct}$**.

P15$_{OOct}$

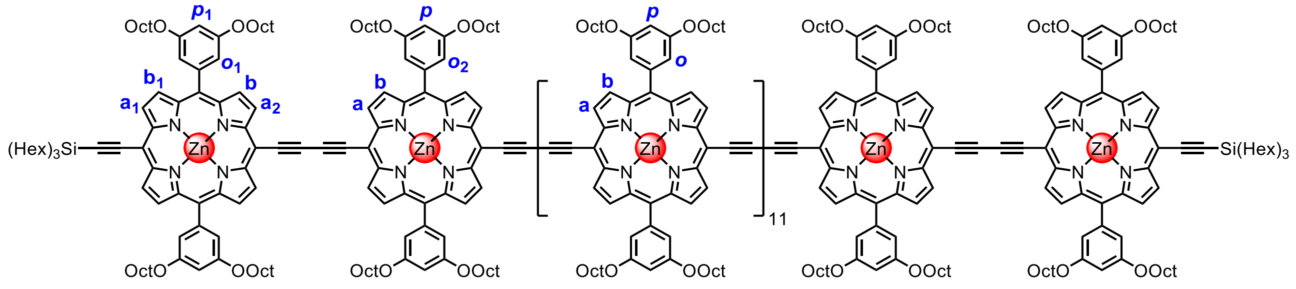

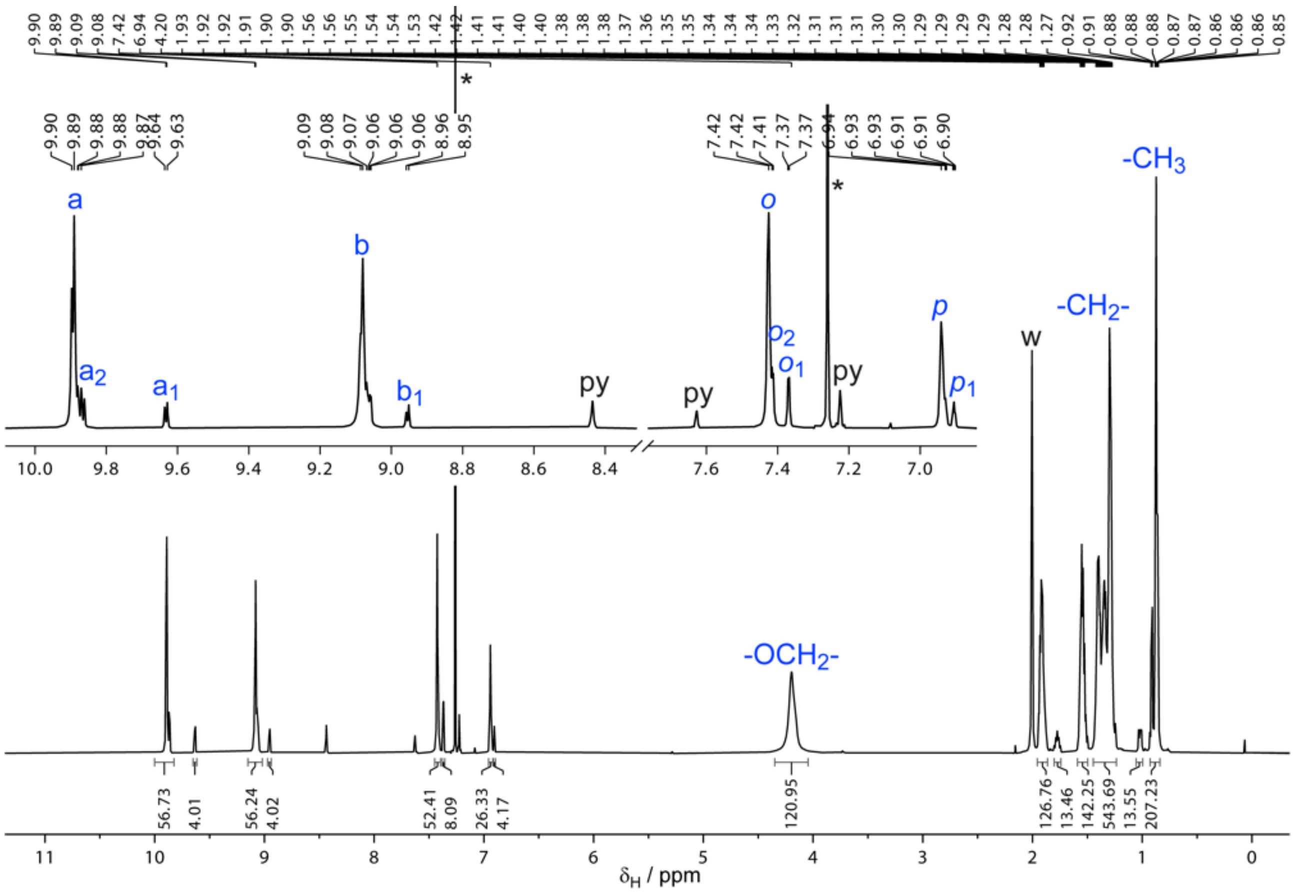

Figure S180. ^{1}H NMR spectrum of **P15$_{OOct}$** (600 MHz, $CDCl_3$, d_5-pyridine, 298 K). * = $CHCl_3$; py = pyridine; w = H_2O.

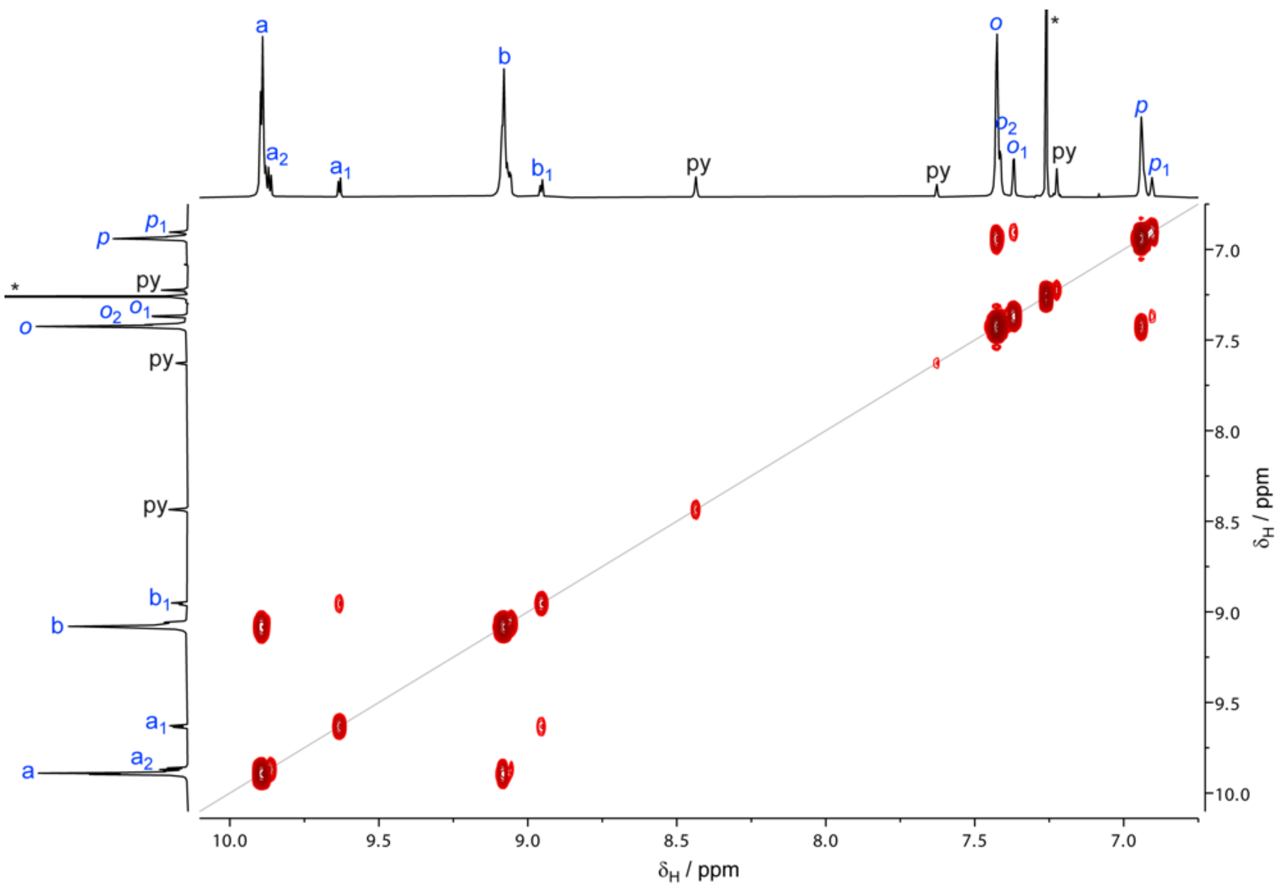

Figure S181. ^{1}H-^{1}H COSY spectrum (aromatic region) of **P15$_{\mathbf{OOct}}$** (600 MHz, $CDCl_3$, *d*$_5$-pyridine, 298 K). * = $CHCl_3$; py = pyridine.

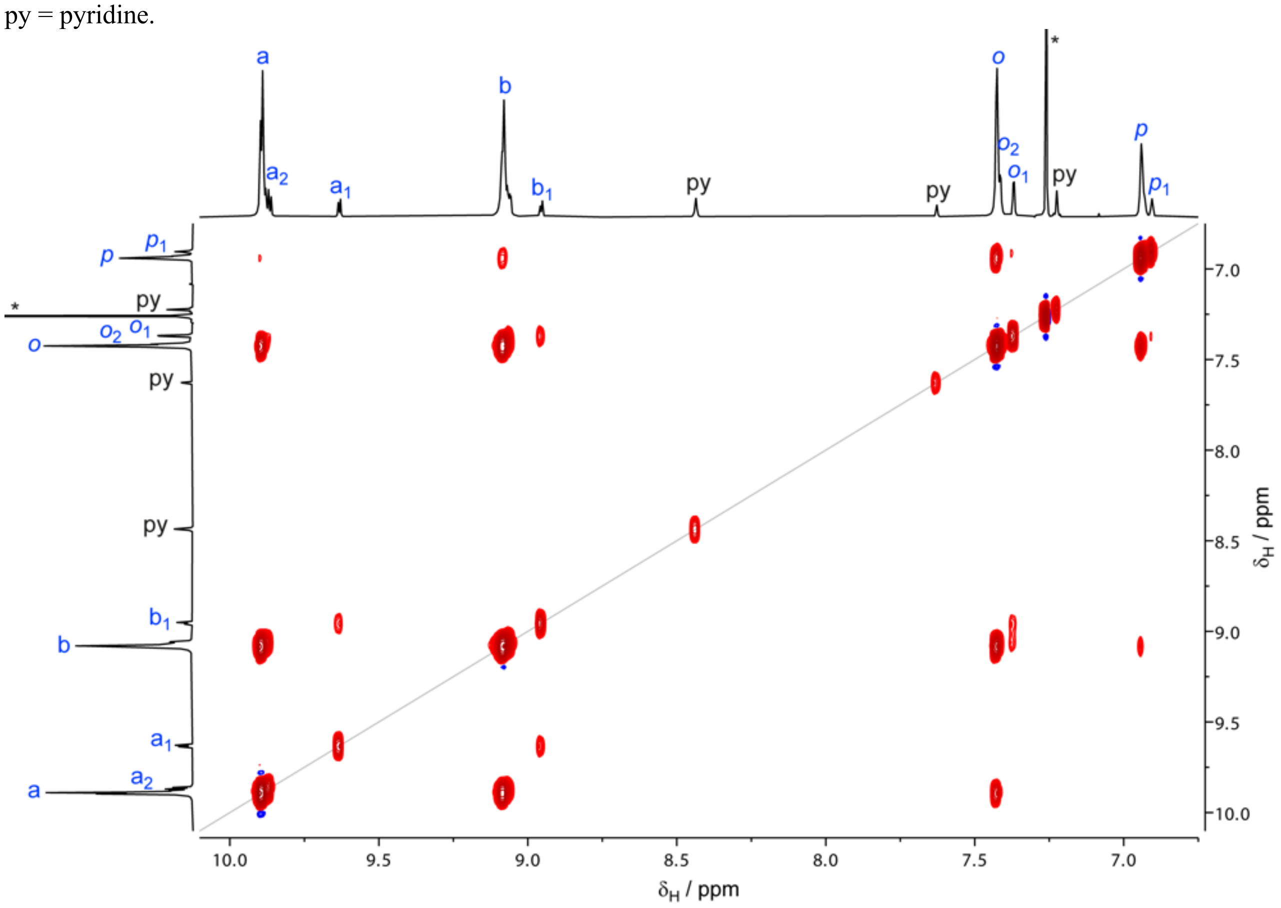

Figure S182. ^{1}H-^{1}H NOESY spectrum (aromatic region) of **P15$_{\mathbf{OOct}}$** (600 MHz, $CDCl_3$, *d*$_5$-pyridine, 298 K). * = $CHCl_3$; py = pyridine.

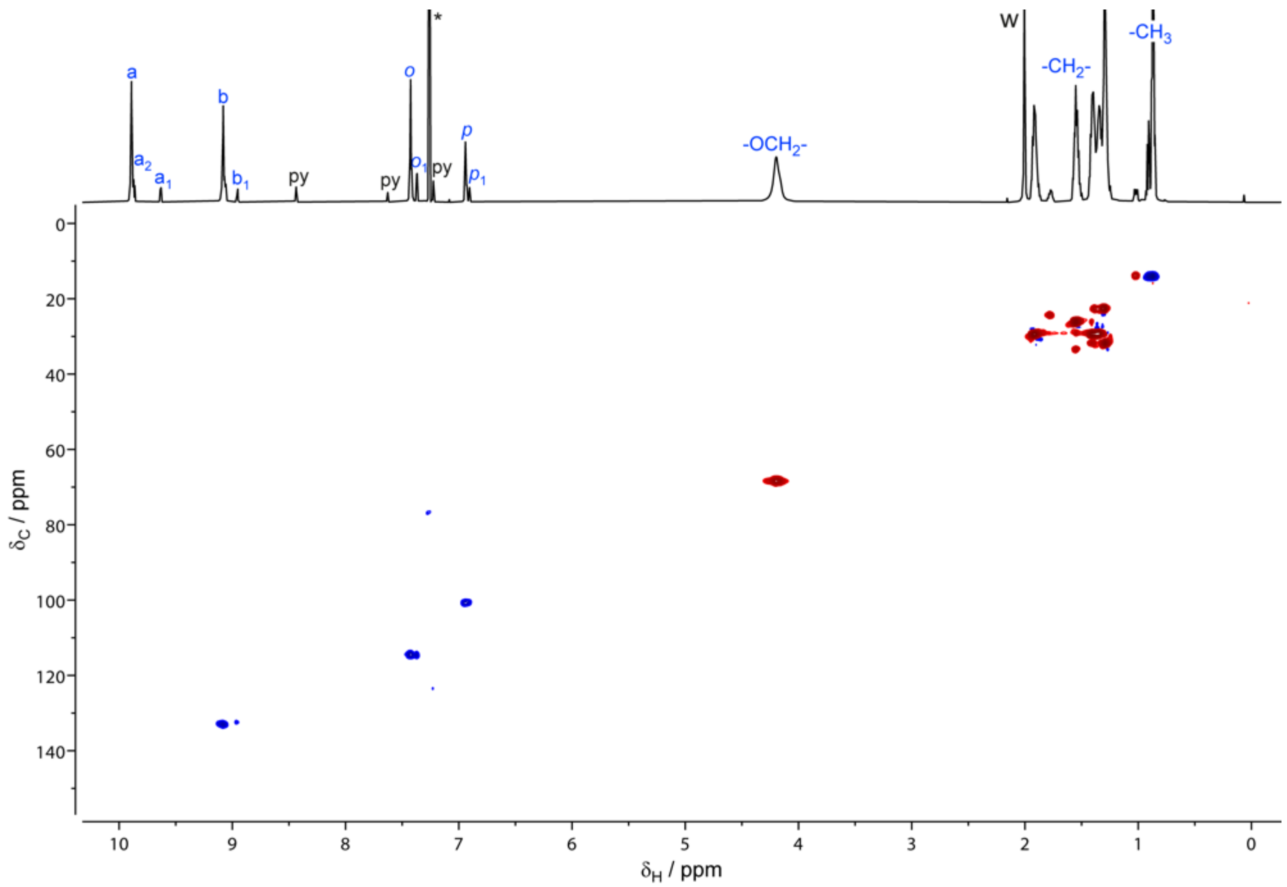

Figure S183. ^{1}H-^{13}C HSQC spectrum of **P15$_{\mathbf{OOct}}$** (600 MHz, $CDCl_3$, *d*$_5$-pyridine, 298 K). * = $CHCl_3$; py = pyridine; w = H_2O.

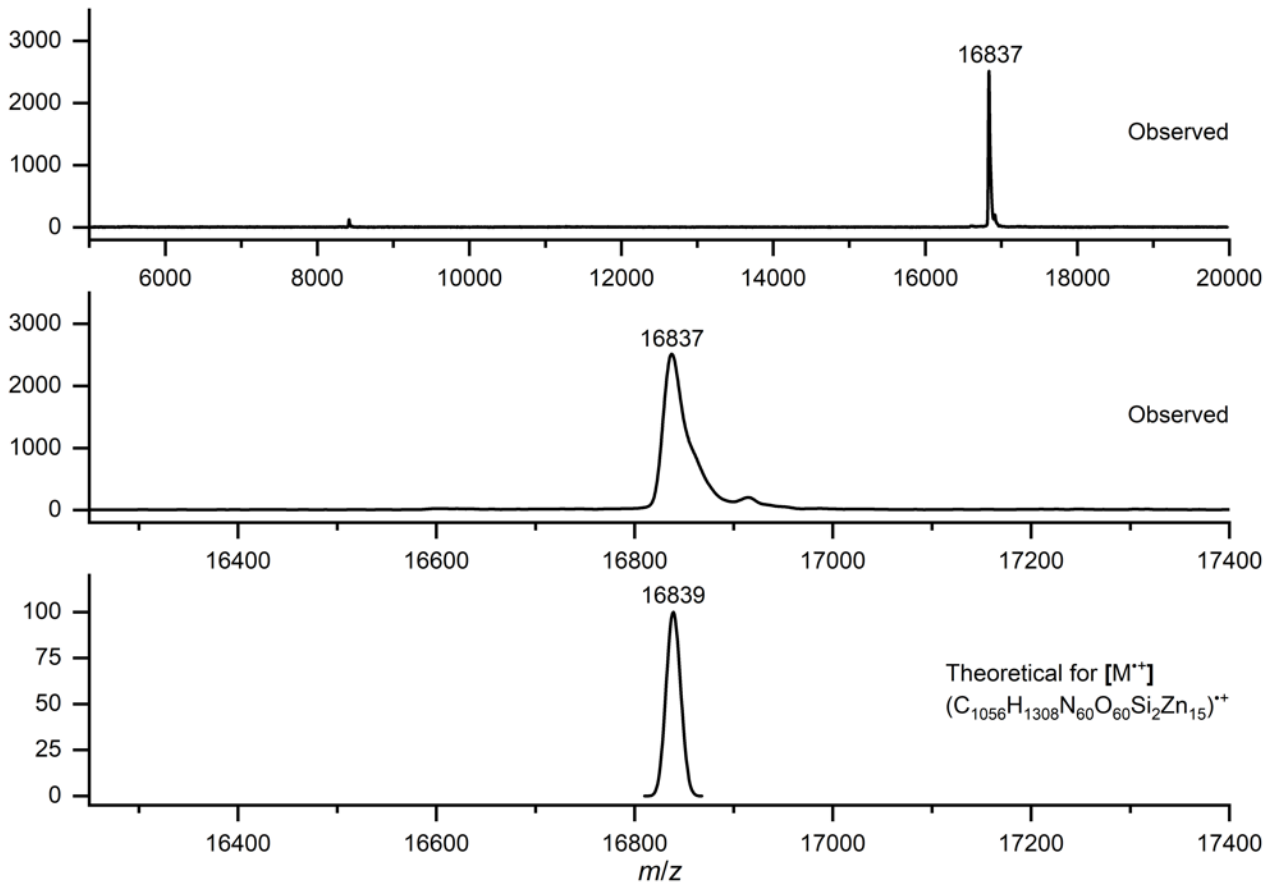

Figure S184. MALDI mass spectrum of **P15$_{\mathbf{OOct}}$**.

Spectra of linear porphyrin oligomers (Ar = THS)

P3THS

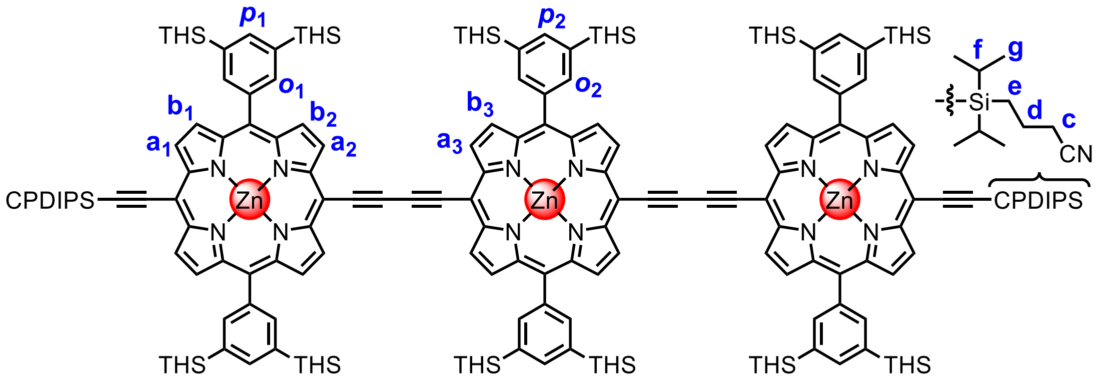

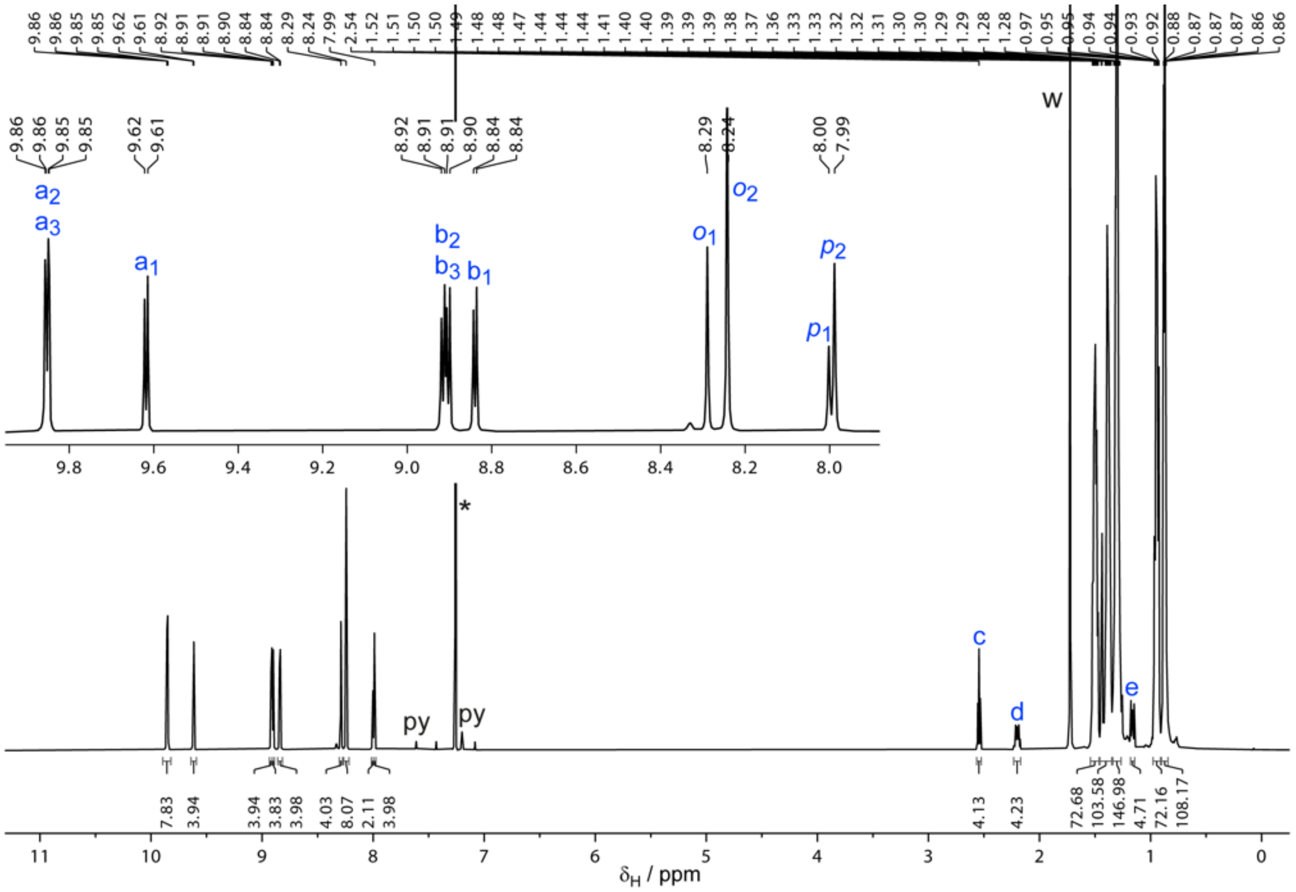

Figure S185. ^{1}H NMR spectrum of **P3THS** (600 MHz, $CDCl_3$, 298 K). * = $CHCl_3$; py = pyridine; w = H_2O.

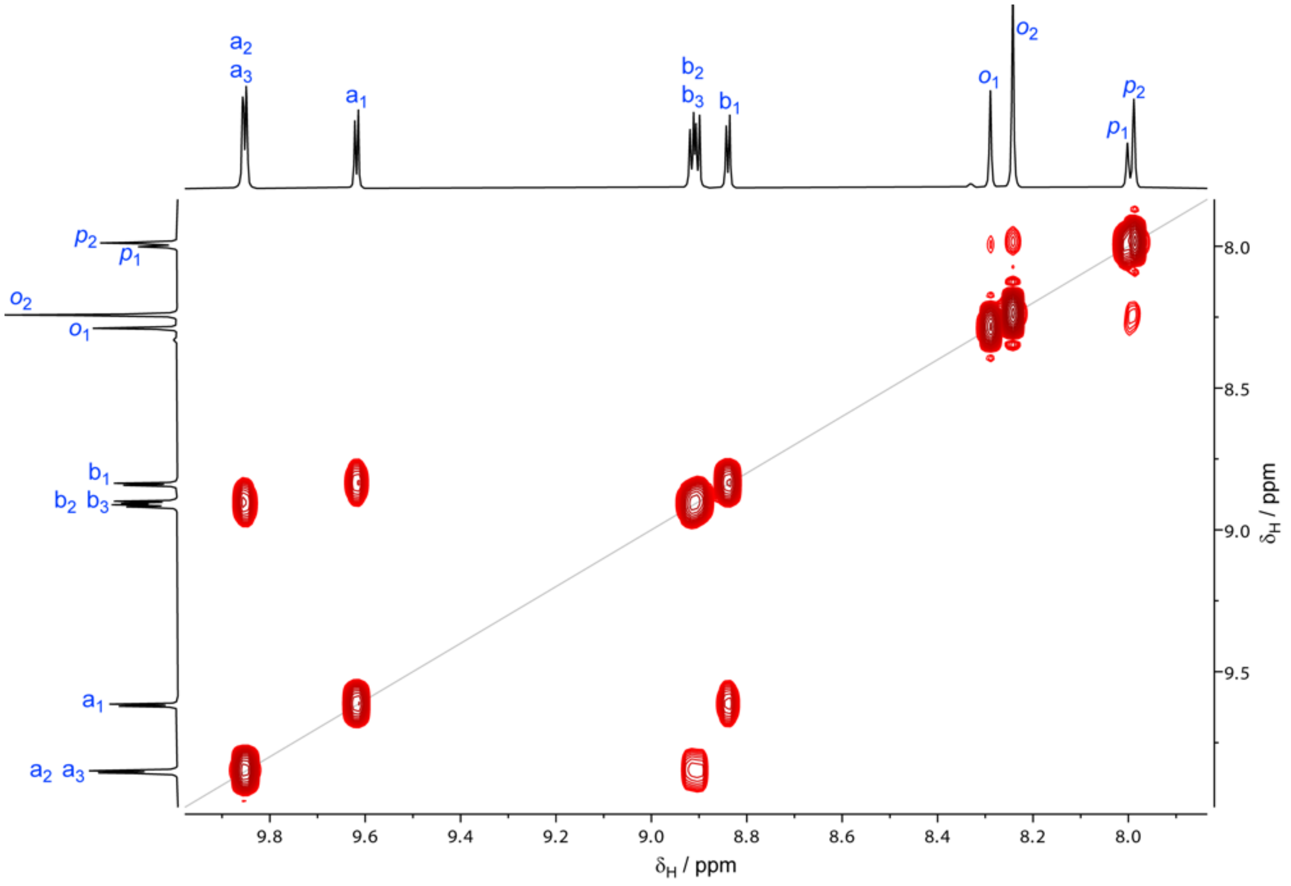

Figure S186. ^{1}H-^{1}H COSY spectrum (aromatic region) of **P3**$_{\text{THS}}$ (600 MHz, $CDCl_3$, 298 K).

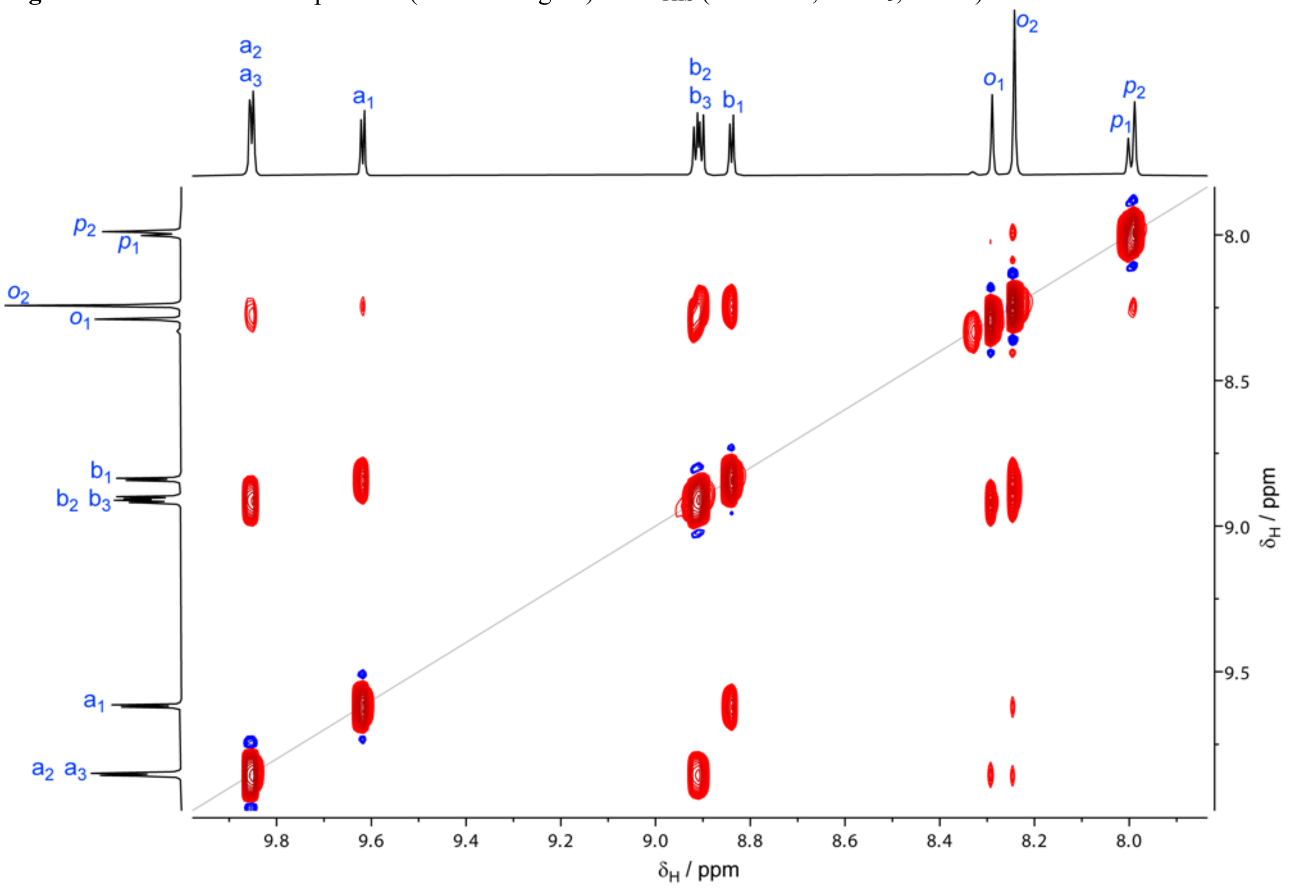

Figure S187. ^{1}H-^{1}H NOESY spectrum (aromatic region) of **P3**$_{\text{THS}}$ (600 MHz, $CDCl_3$, 298 K).

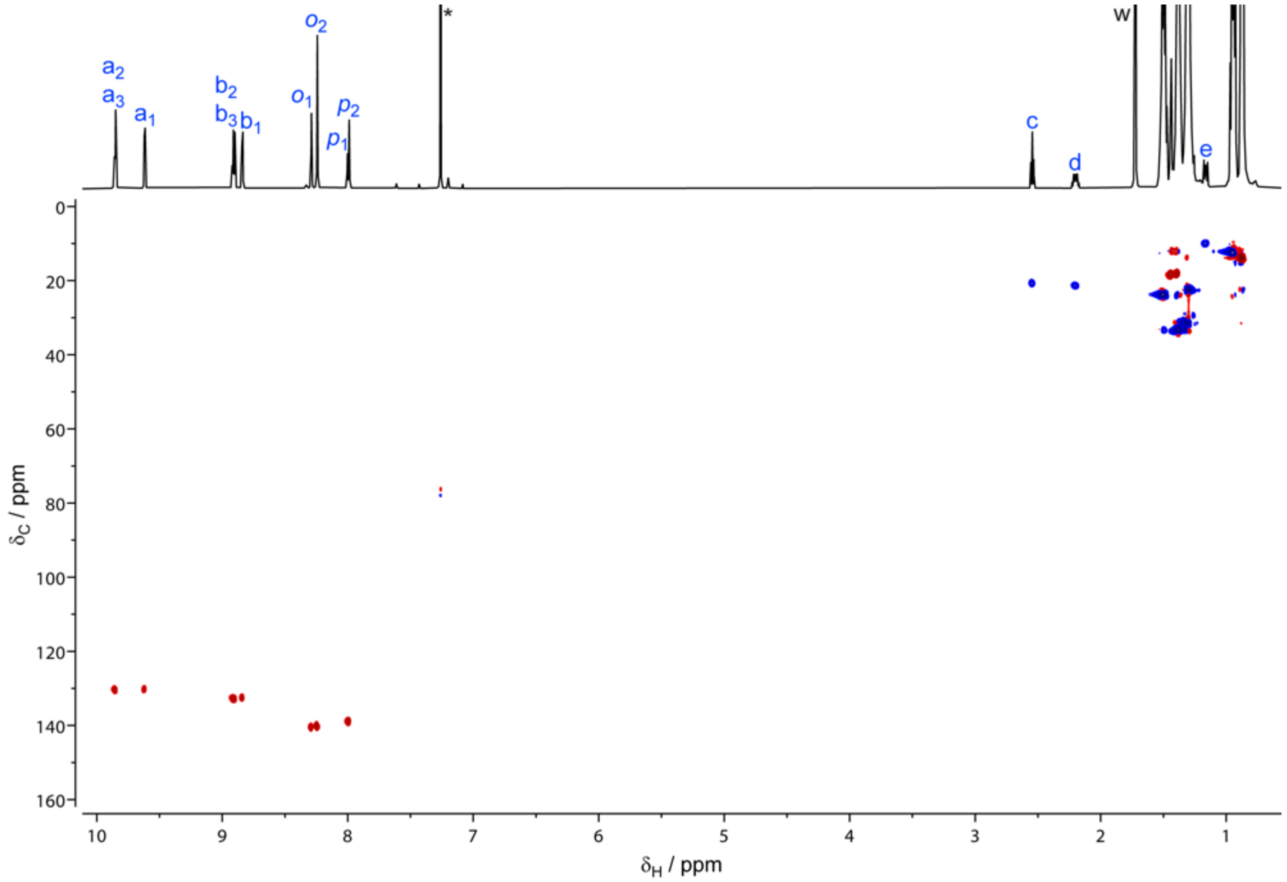

Figure S188. ^{1}H-^{13}C HSQC spectrum of **P3$_{THS}$** (600 MHz, $CDCl_3$, 298 K). * = $CHCl_3$; w = H_2O.

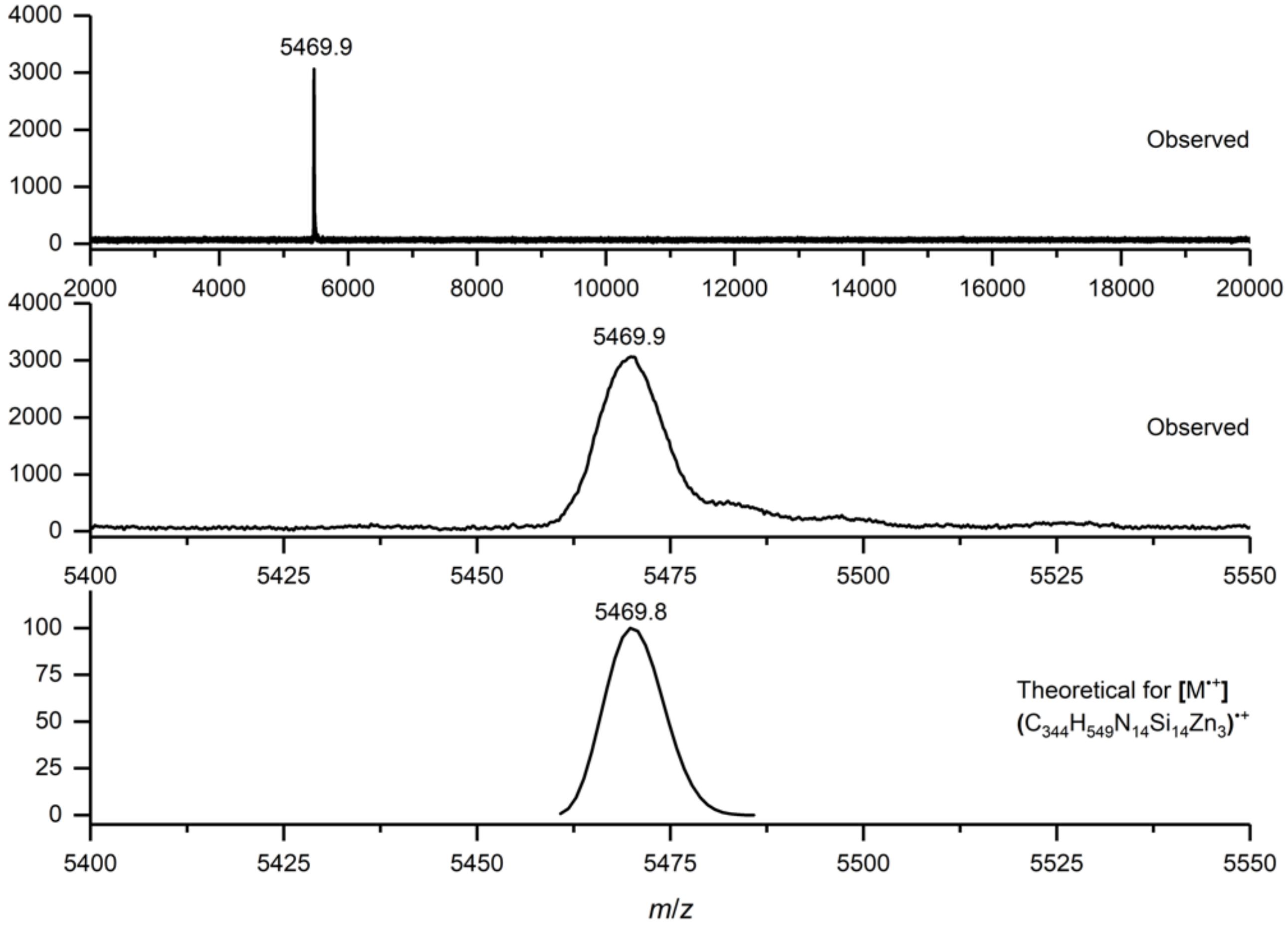

Figure S189. MALDI mass spectrum of **P3$_{THS}$**.

P6$_{THS}$

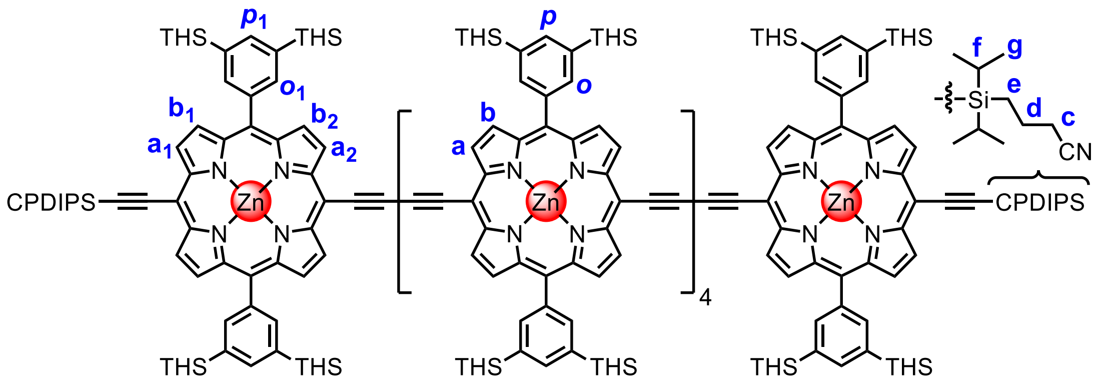

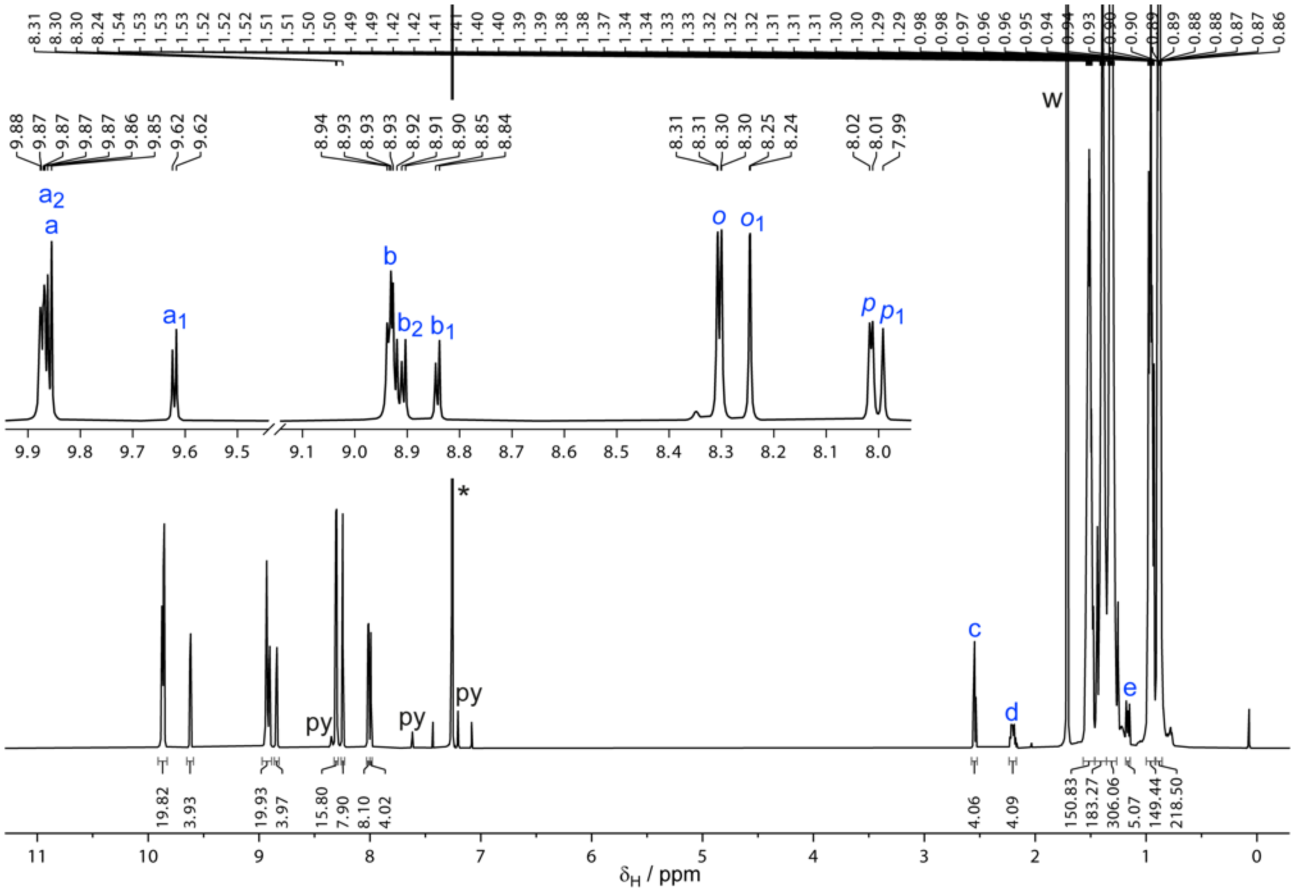

Figure S190. ^{1}H NMR spectrum of **P6$_{THS}$** (600 MHz, $CDCl_3$, *d*$_5$-pyridine, 298 K). * = $CHCl_3$; py = pyridine; w = H_2O.

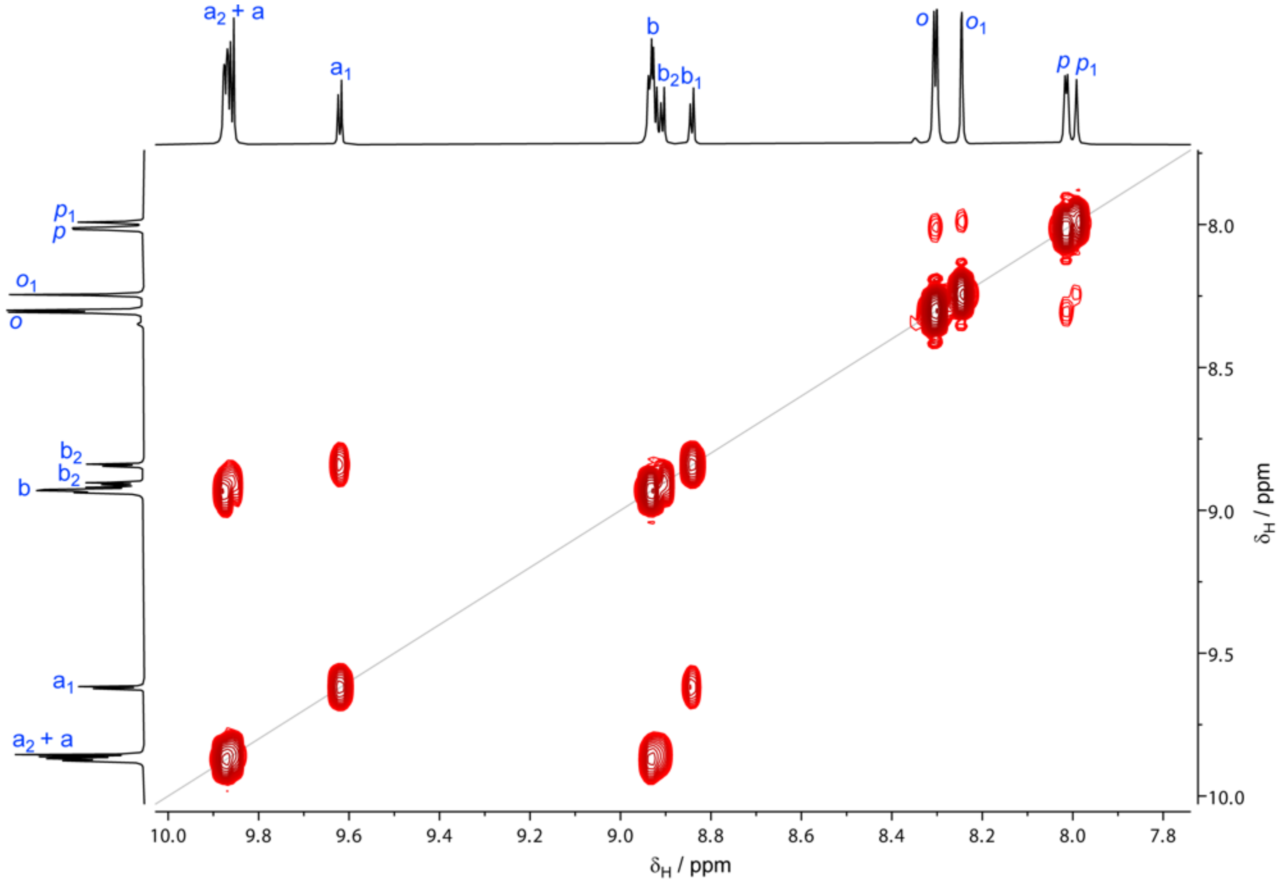

Figure S191. ^{1}H-^{1}H COSY spectrum (aromatic region) of **P6THS** (600 MHz, $CDCl_3$, *d*5-pyridine, 298 K).

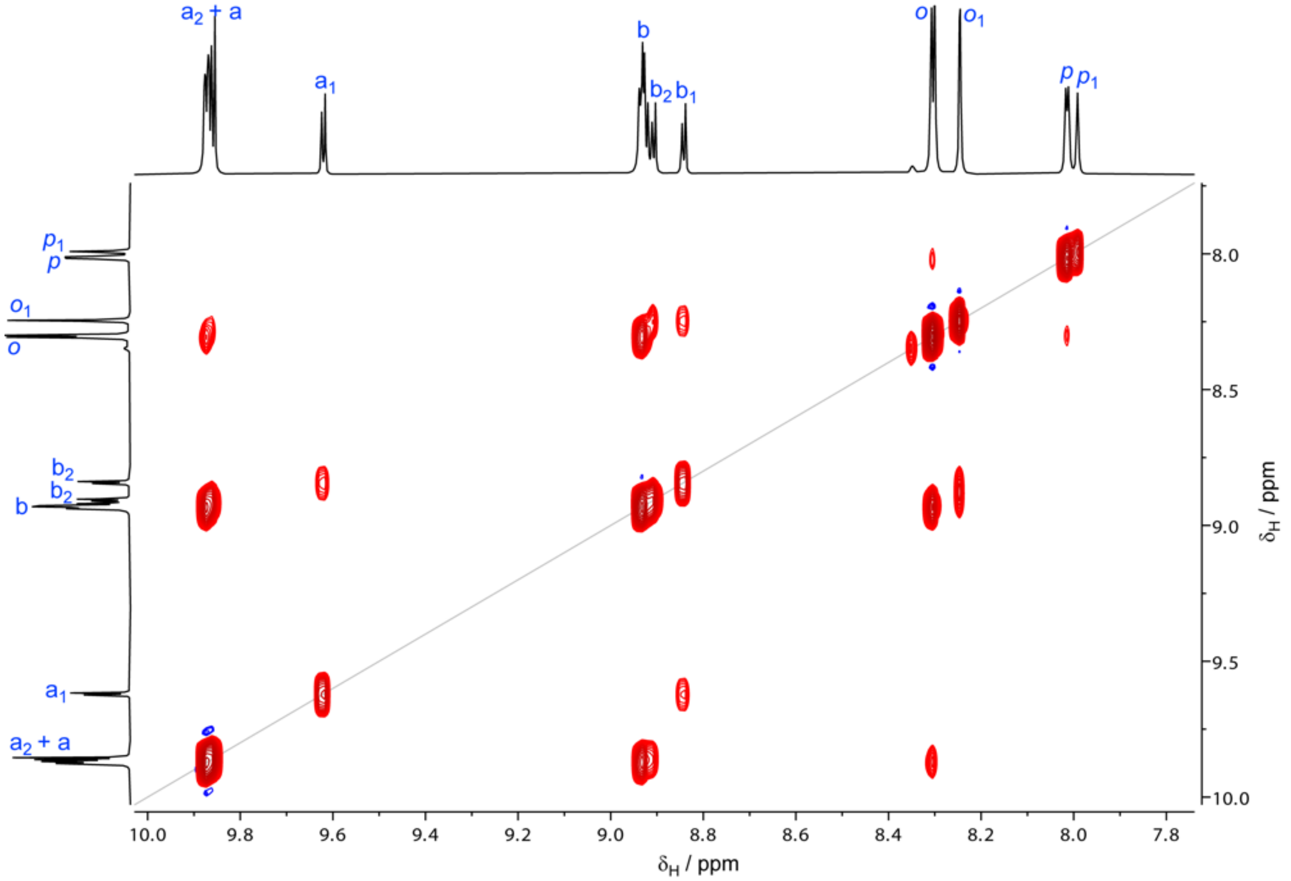

Figure S192. ^{1}H-^{1}H NOESY spectrum (aromatic region) of **P6THS** (600 MHz, $CDCl_3$, *d*5-pyridine, 298 K).

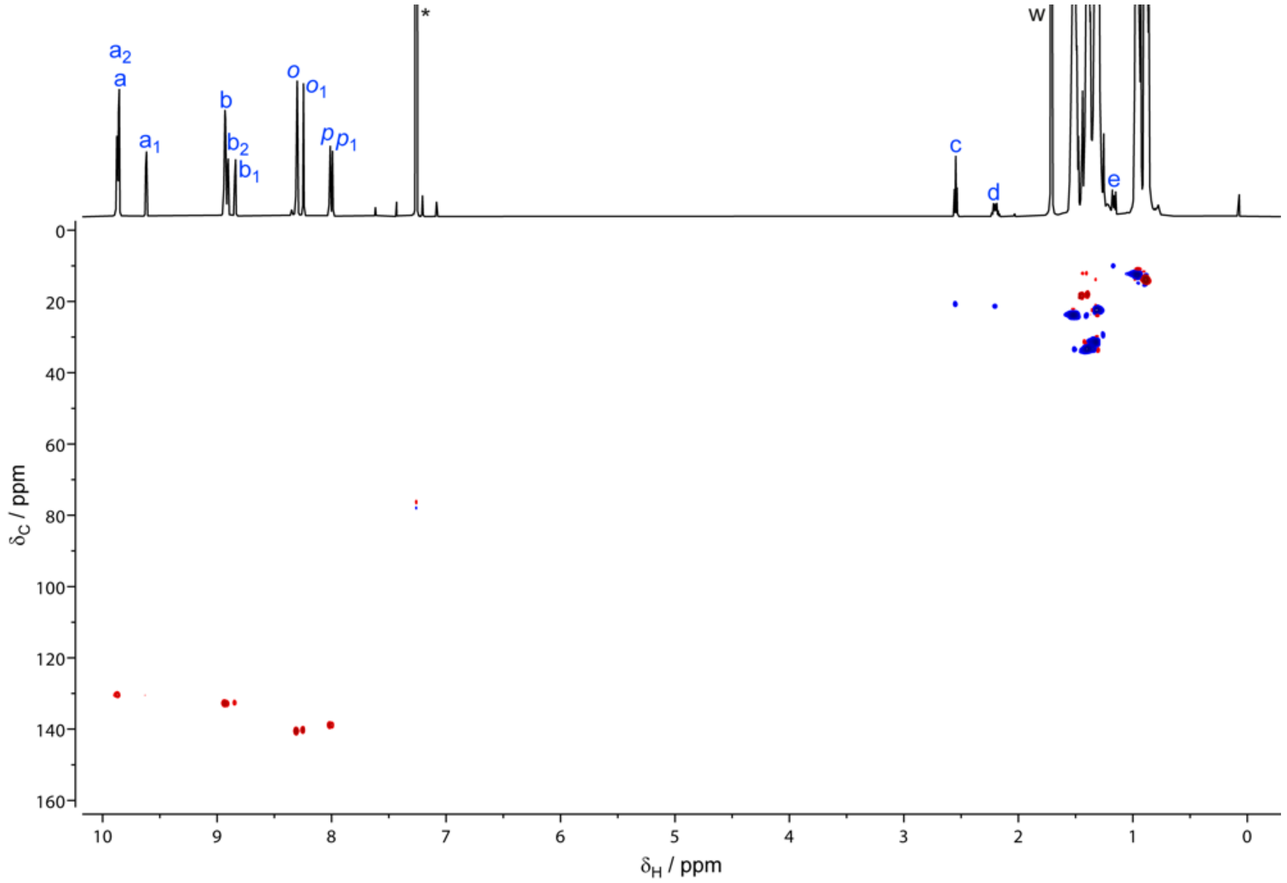

Figure S193. ^{1}H-^{13}C HSQC spectrum of **P6$_{THS}$** (600 MHz, $CDCl_3$, 298 K). * = $CHCl_3$; w = H_2O.

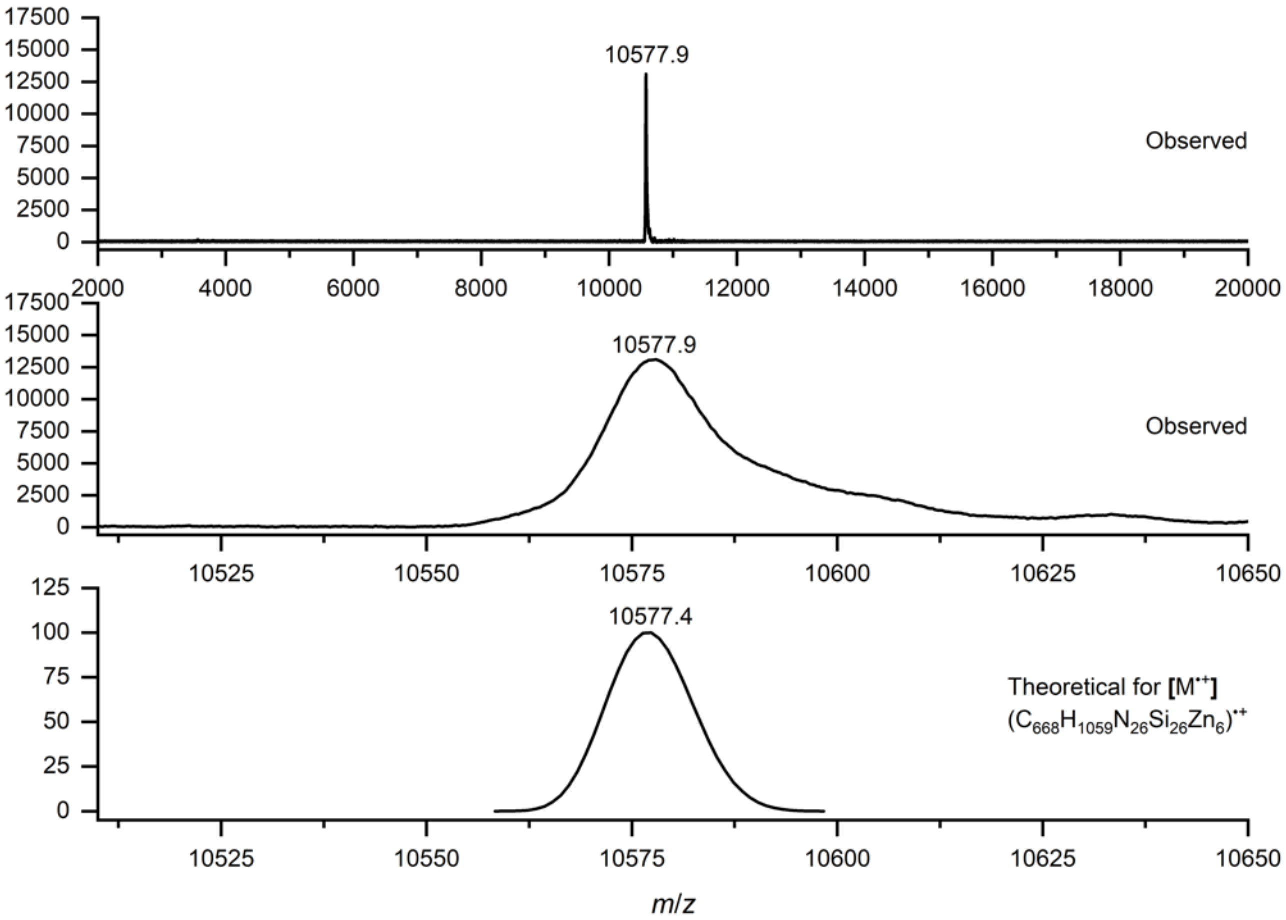

Figure S194. MALDI mass spectrum of **P6$_{THS}$**.

P9$_{THS}$

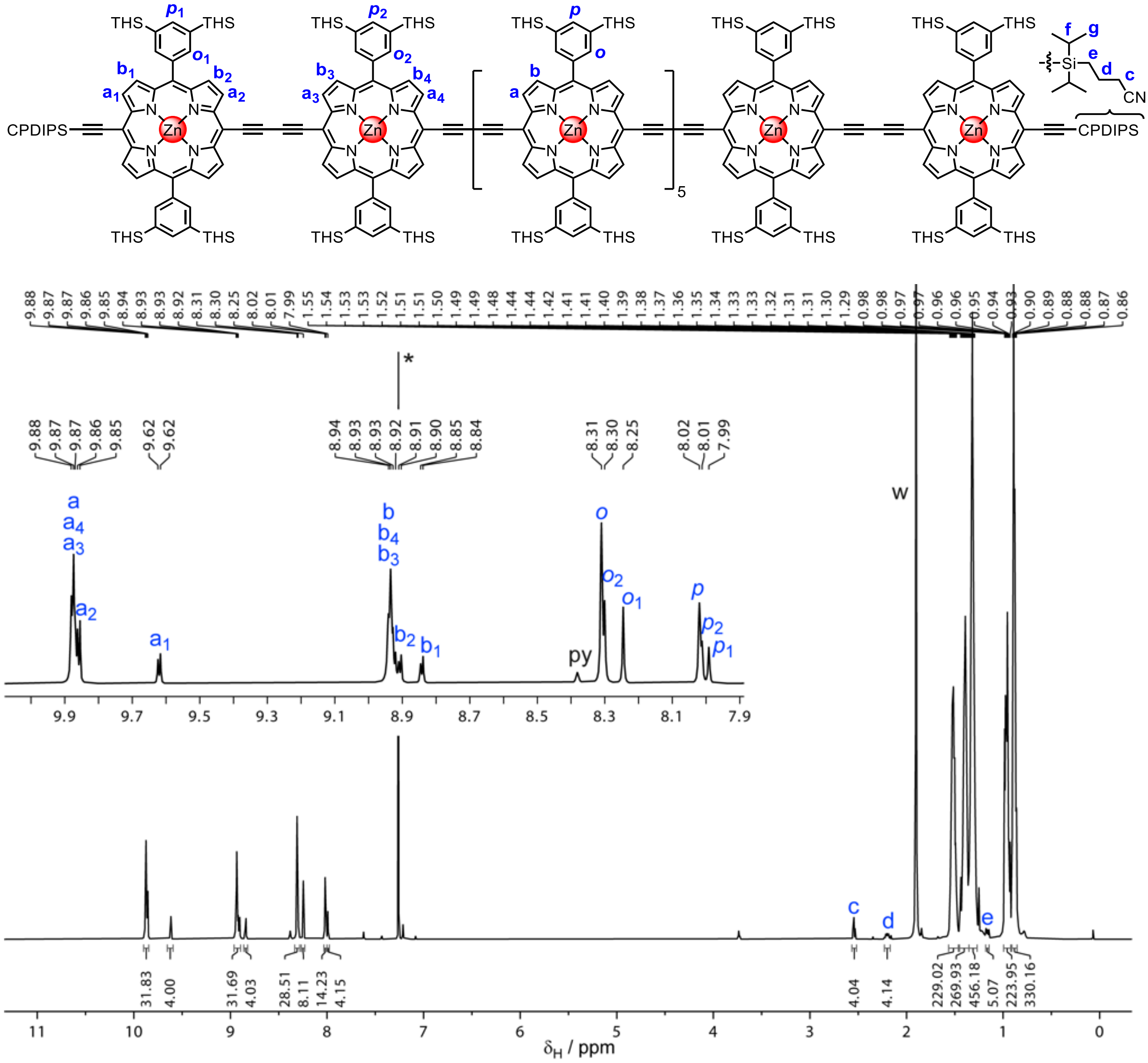

Figure S195. ^{1}H NMR spectrum of **P9$_{THS}$** (600 MHz, $CDCl_3$, d_5-pyridine, 298 K). * = $CHCl_3$; py = pyridine; w = H_2O.

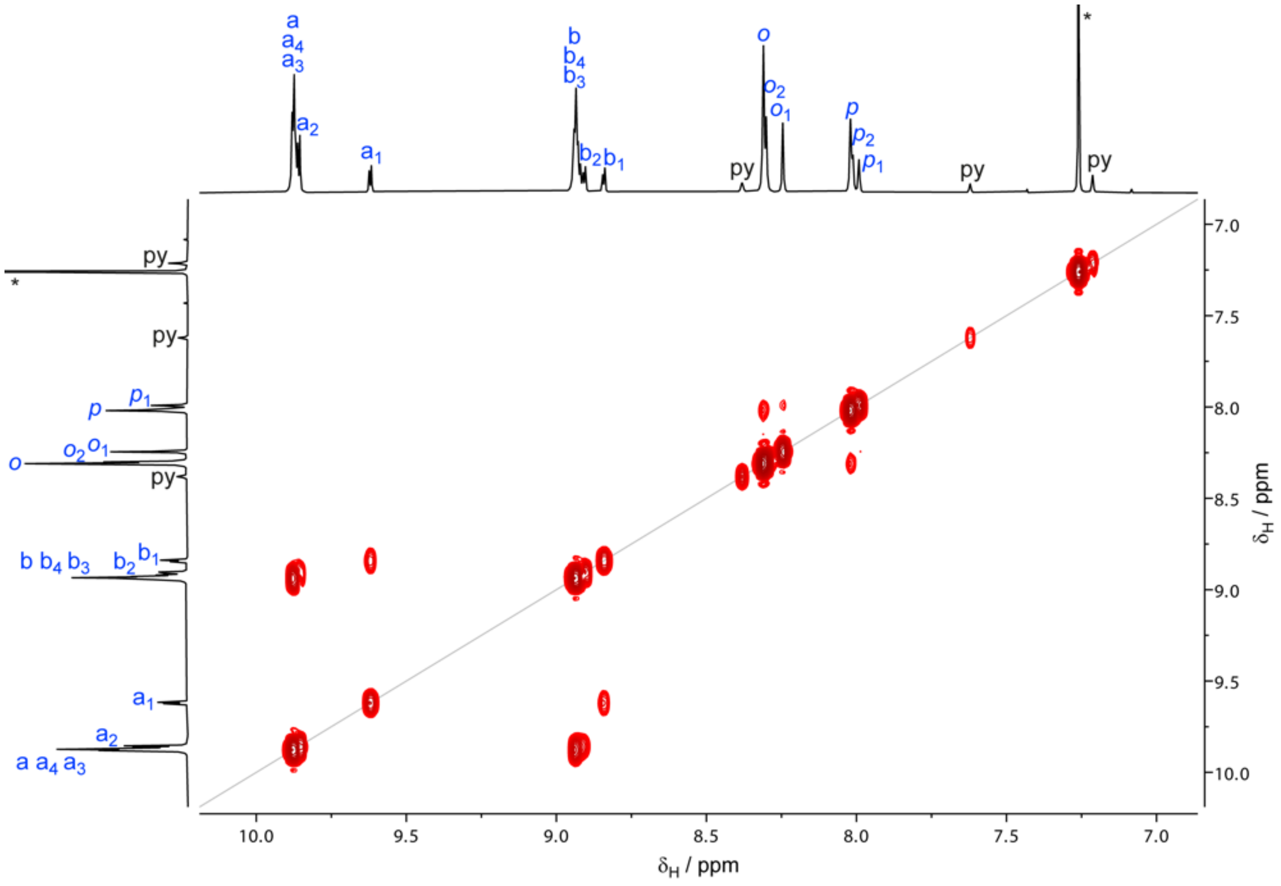

Figure S196. ^{1}H-^{1}H COSY spectrum (aromatic region) of **P9THS** (600 MHz, $CDCl_3$, *d*5-pyridine, 298 K). * = $CHCl_3$; py = pyridine.

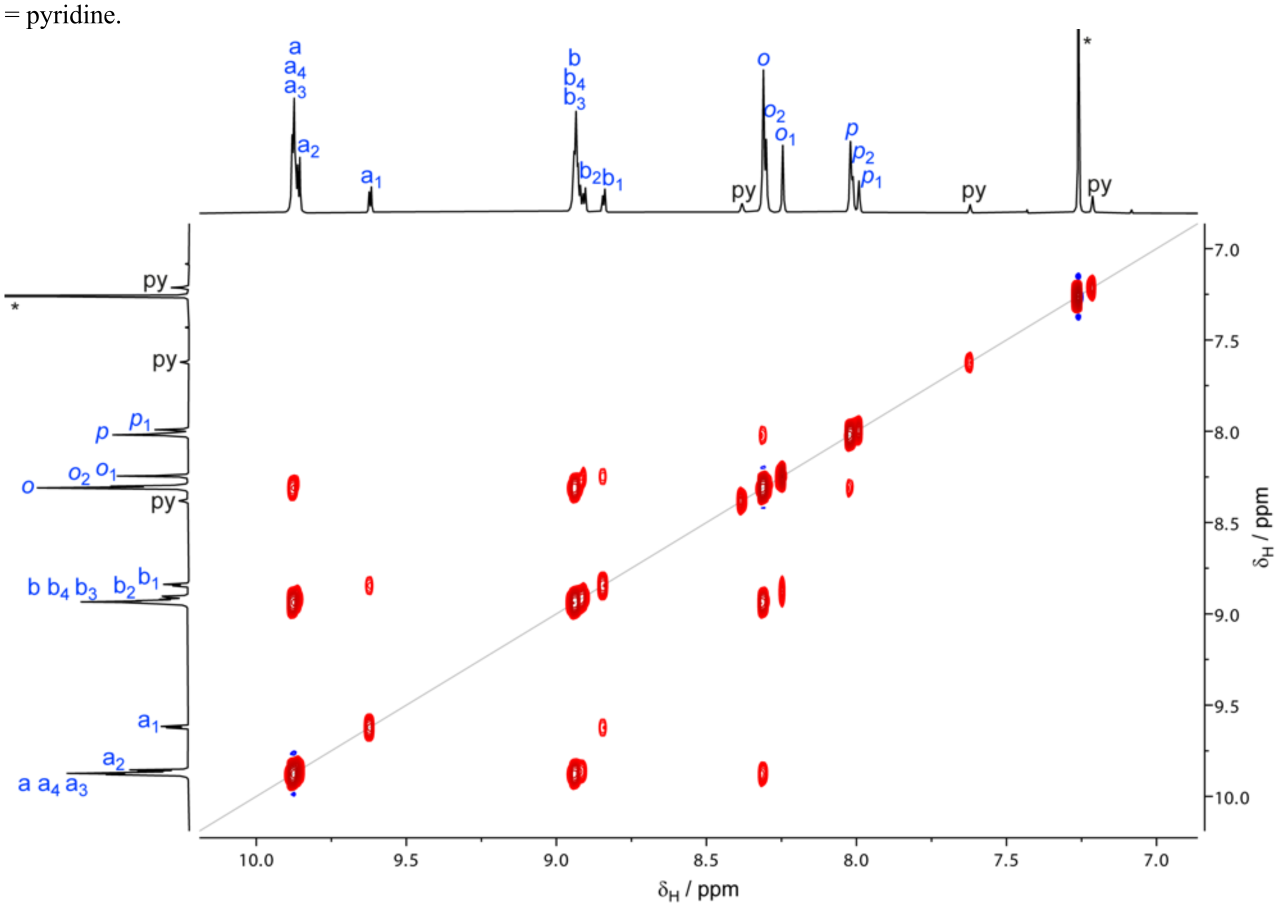

Figure S197. ^{1}H-^{1}H NOESY spectrum (aromatic region) of **P9THS** (600 MHz, $CDCl_3$, *d*5-pyridine, 298 K). * = $CHCl_3$; py = pyridine.

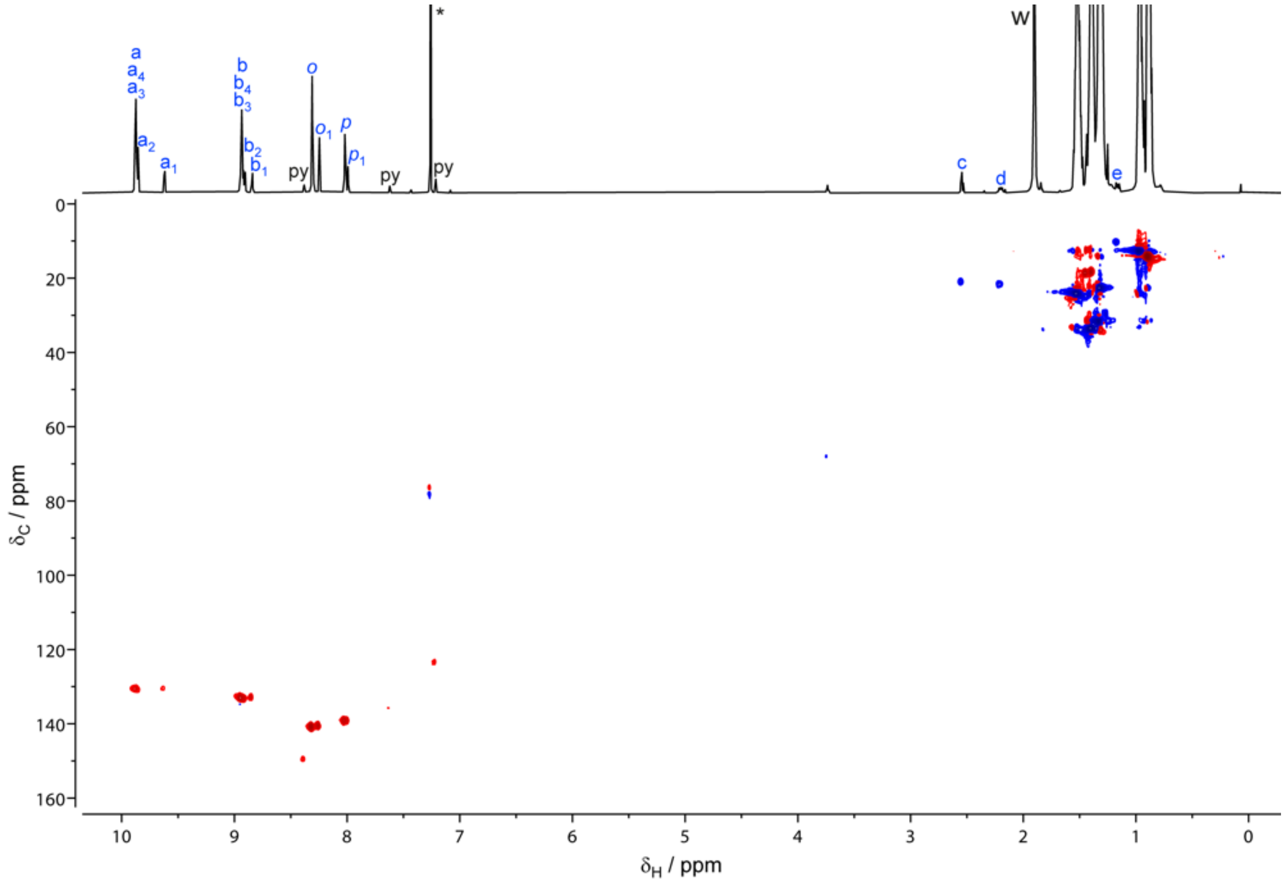

Figure S198. ^{1}H-^{13}C HSQC spectrum of **P9**$_{\mathbf{THS}}$ (600 MHz, $CDCl_3$, d_5-pyridine, 298 K). * = $CHCl_3$; py = pyridine; w = H_2O.

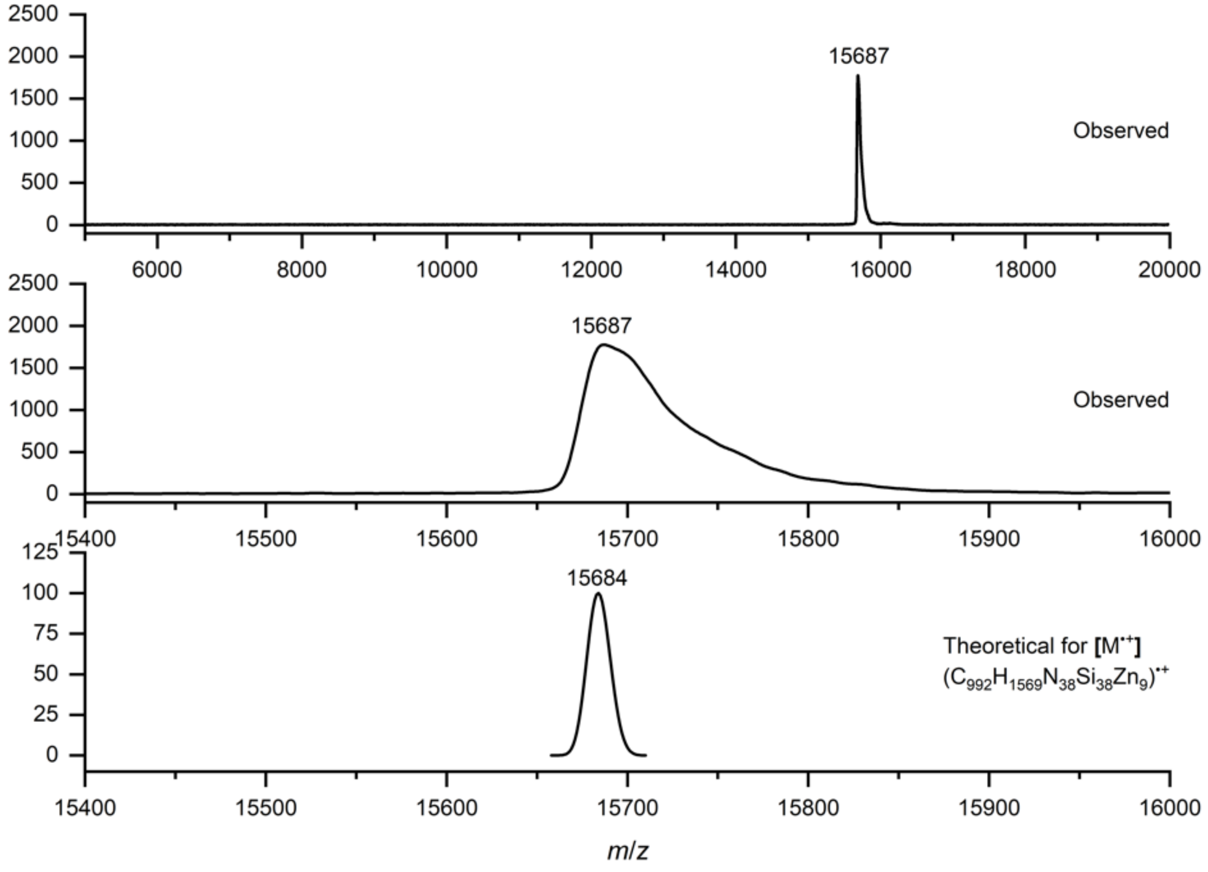

Figure S199. MALDI mass spectrum of **P9**$_{\mathbf{THS}}$.

P12$_{THS}$

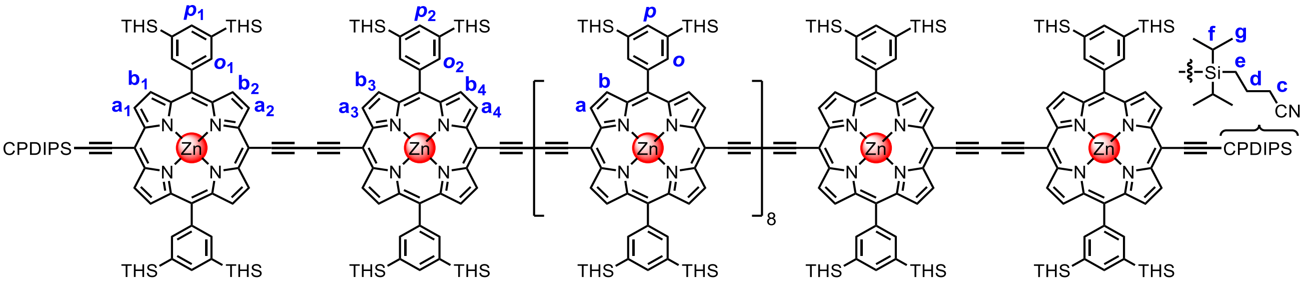

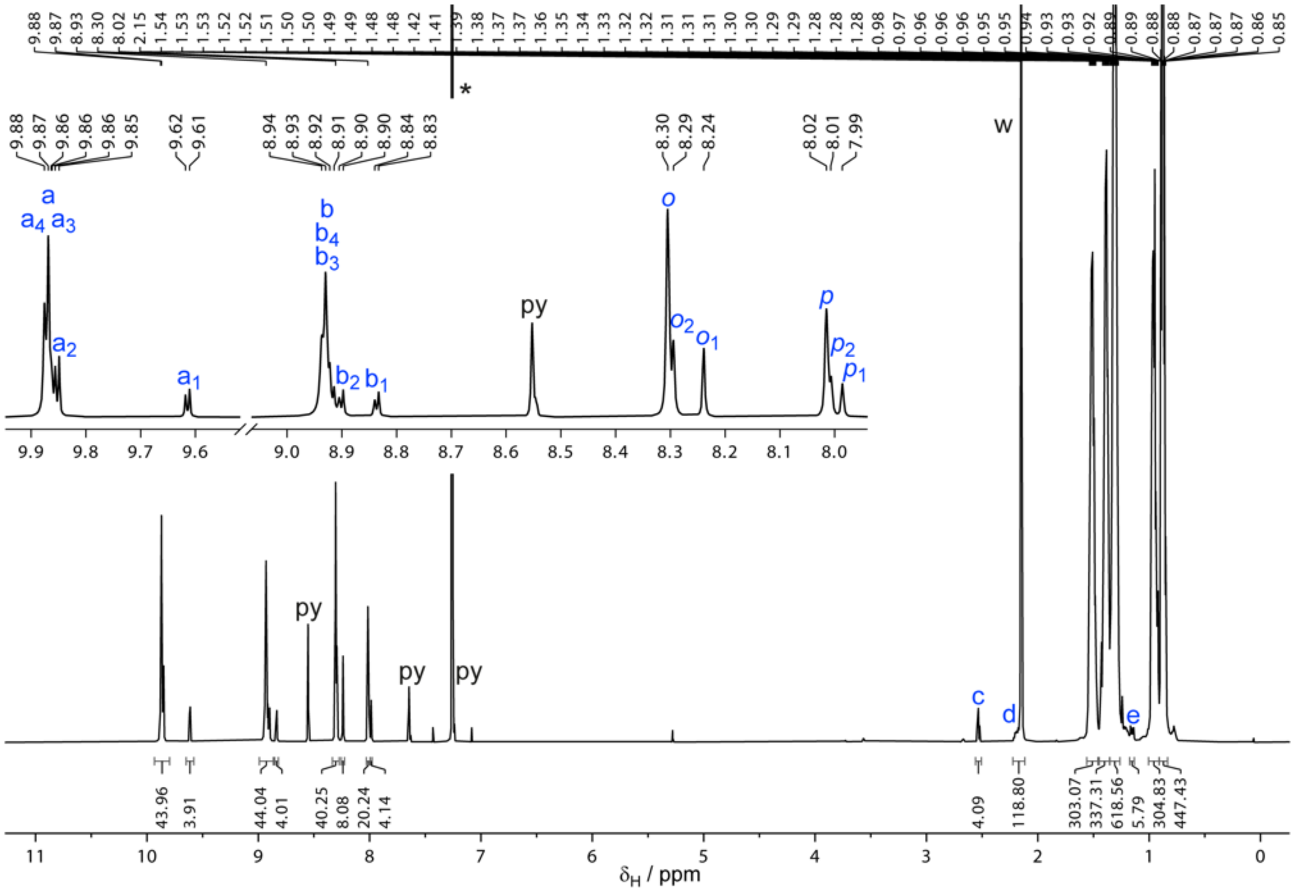

Figure S200. ^{1}H NMR spectrum of **P12$_{THS}$** (600 MHz, $CDCl_3$, d_5-pyridine, 298 K). * = $CHCl_3$; py = pyridine; w = H_2O.

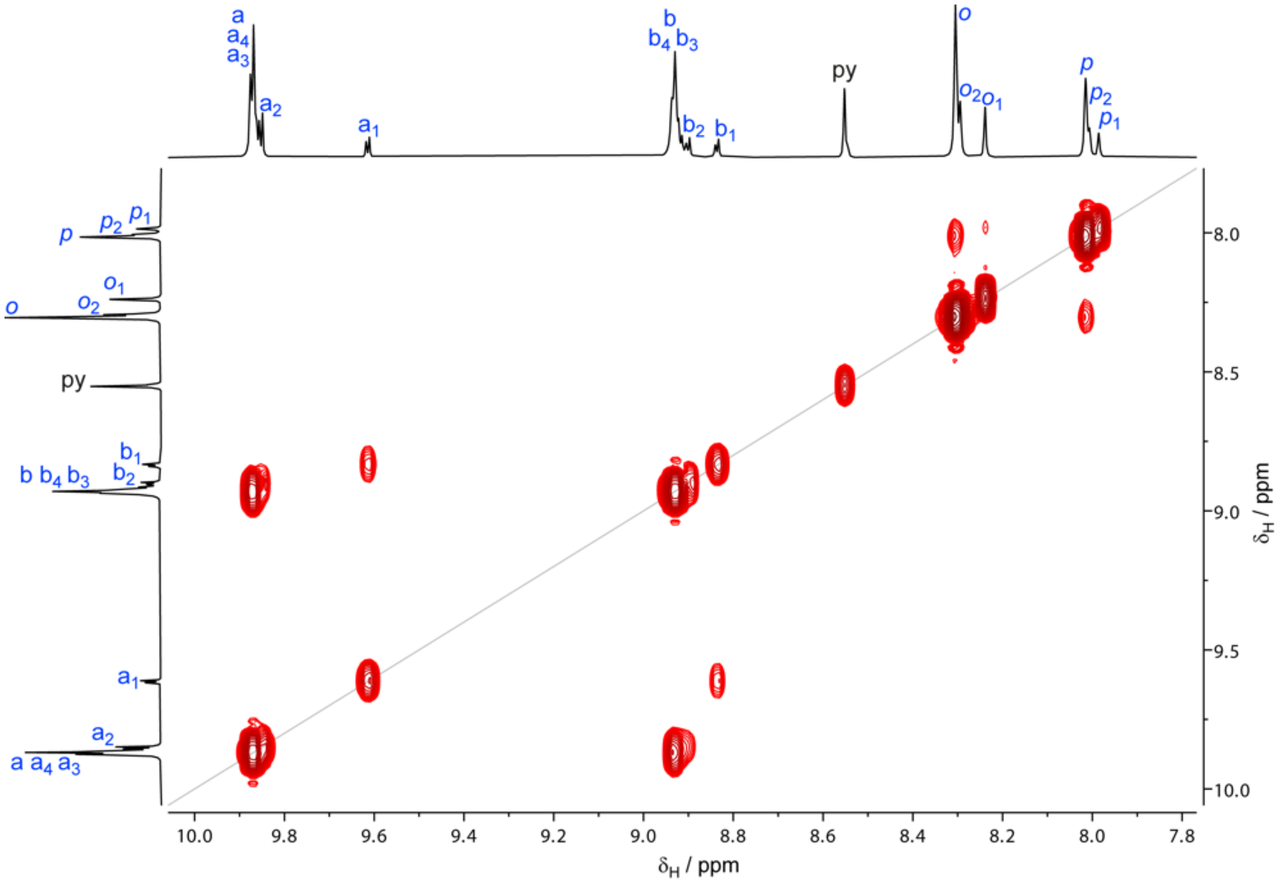

Figure S201. ^{1}H-^{1}H COSY spectrum (aromatic region) of **P12THS** (600 MHz, $CDCl_3$, *d*5-pyridine, 298 K). * = $CHCl_3$; py = pyridine.

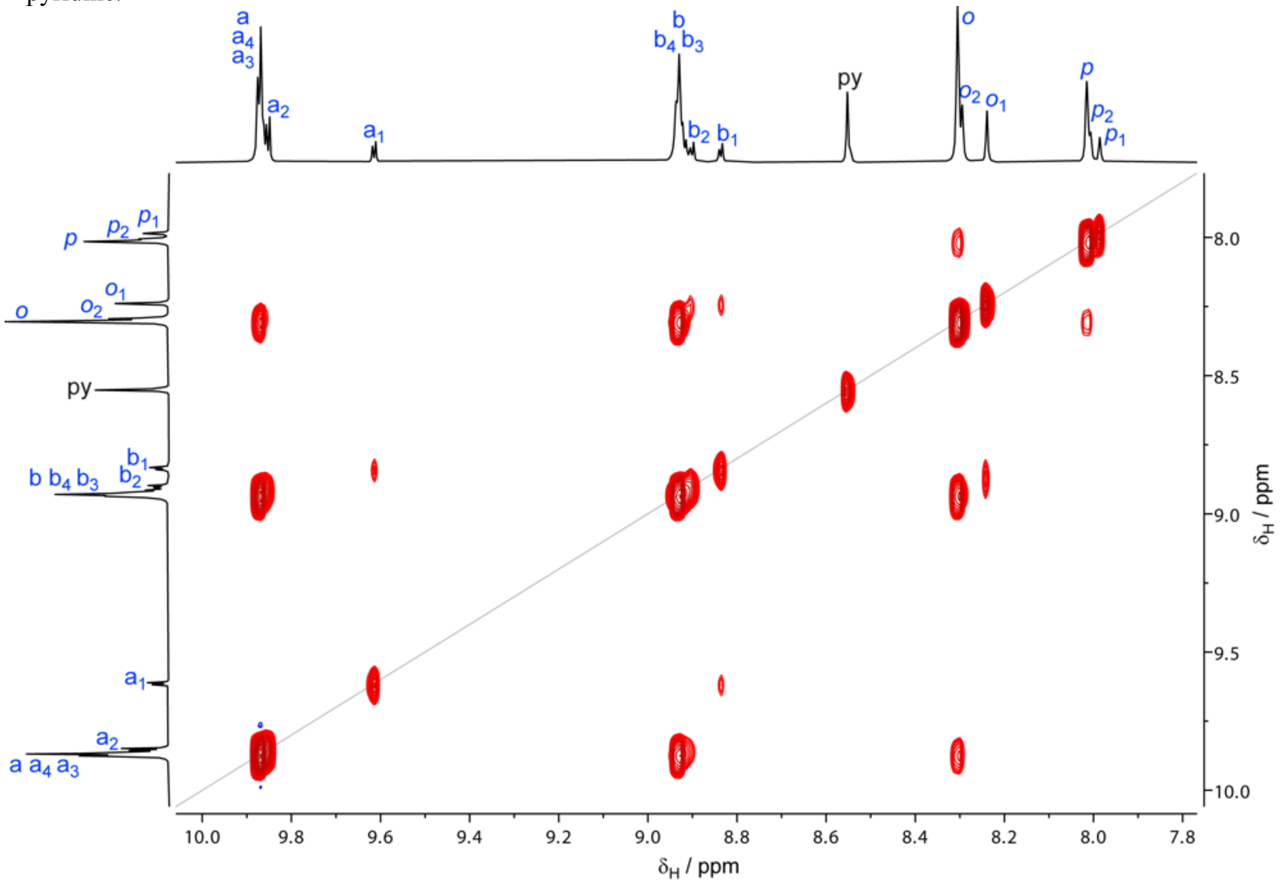

Figure S202. ^{1}H-^{1}H NOESY spectrum (aromatic region) of **P12THS** (600 MHz, $CDCl_3$, *d*5-pyridine, 298 K). * = $CHCl_3$; py = pyridine.

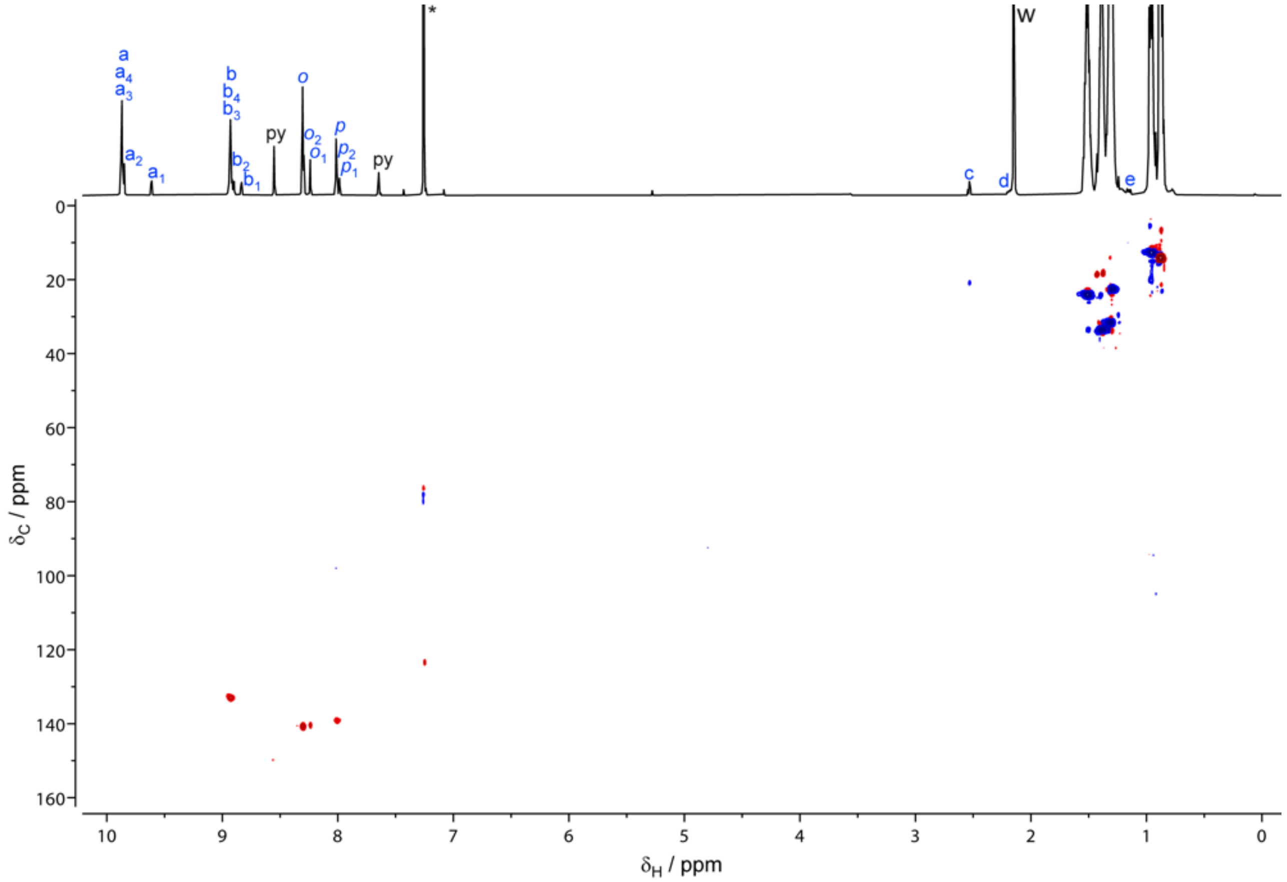

Figure S203. ^{1}H-^{13}C HSQC spectrum of **P12$_{THS}$** (600 MHz, $CDCl_3$, d_5-pyridine, 298 K). * = $CHCl_3$; py = pyridine; w = H_2O.

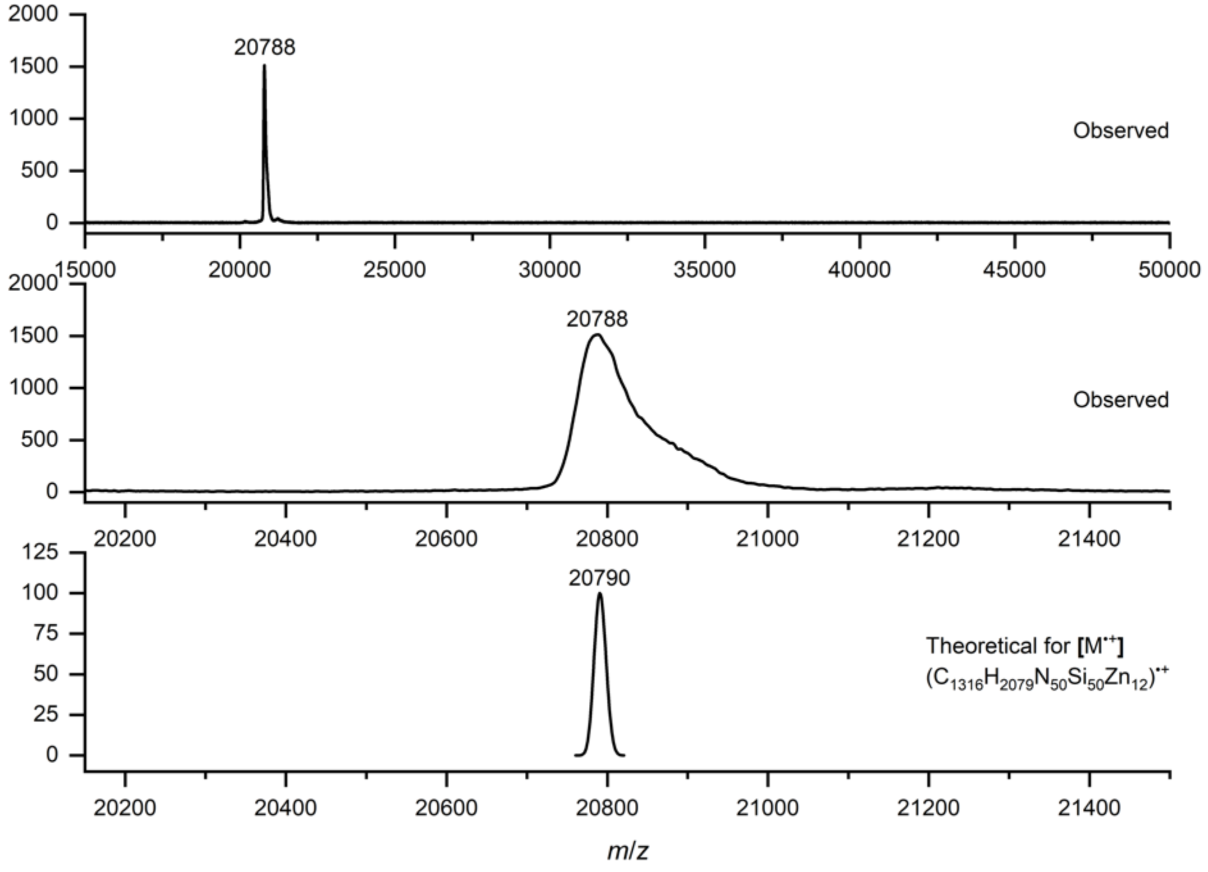

Figure S204. MALDI mass spectrum of **P12$_{THS}$**.

P15$_{THS}$

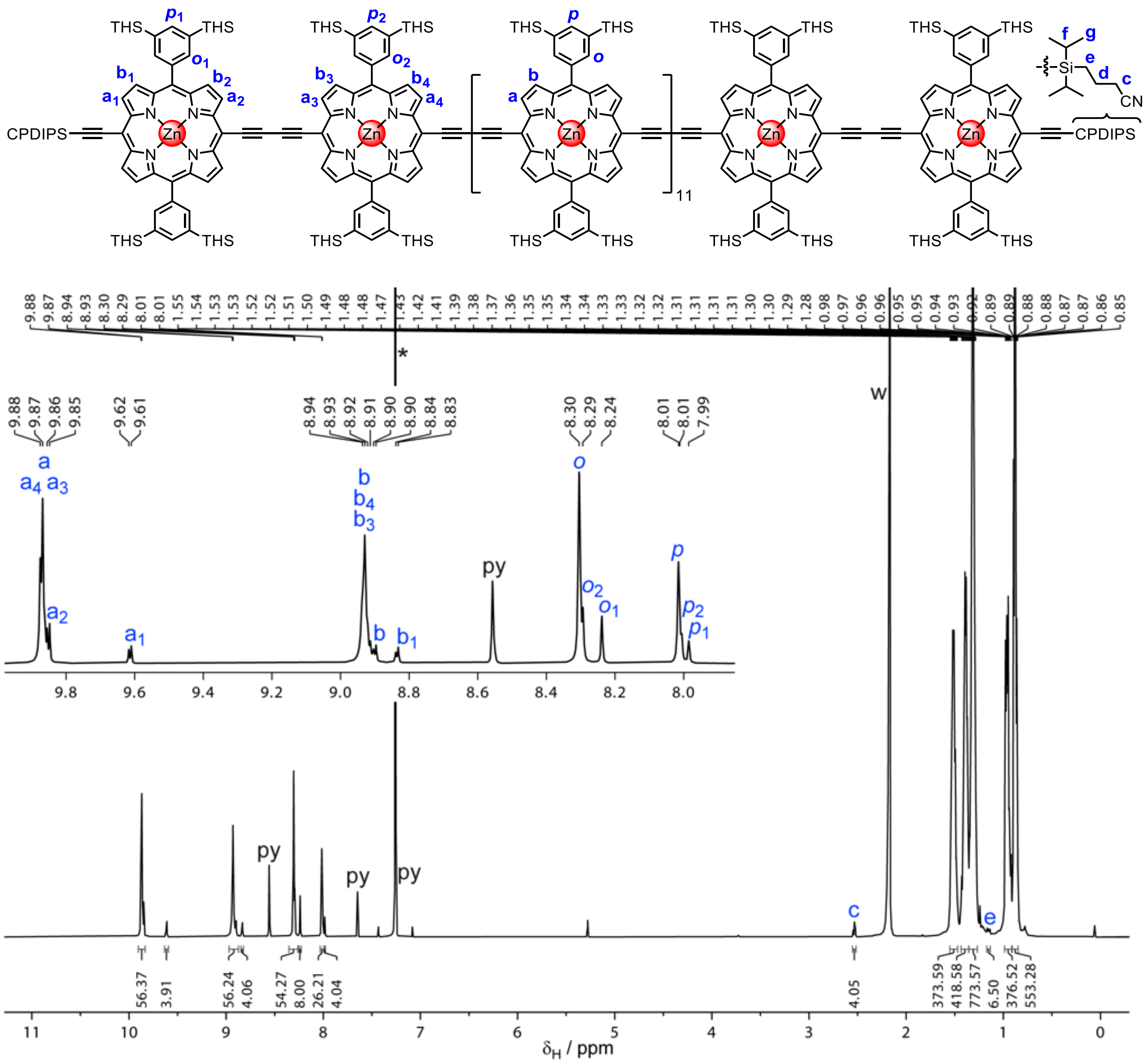

Figure S205. ^{1}H NMR spectrum of **P15$_{THS}$** (600 MHz, $CDCl_3$, d_5-pyridine, 298 K). * = $CHCl_3$; py = pyridine; w = H_2O.

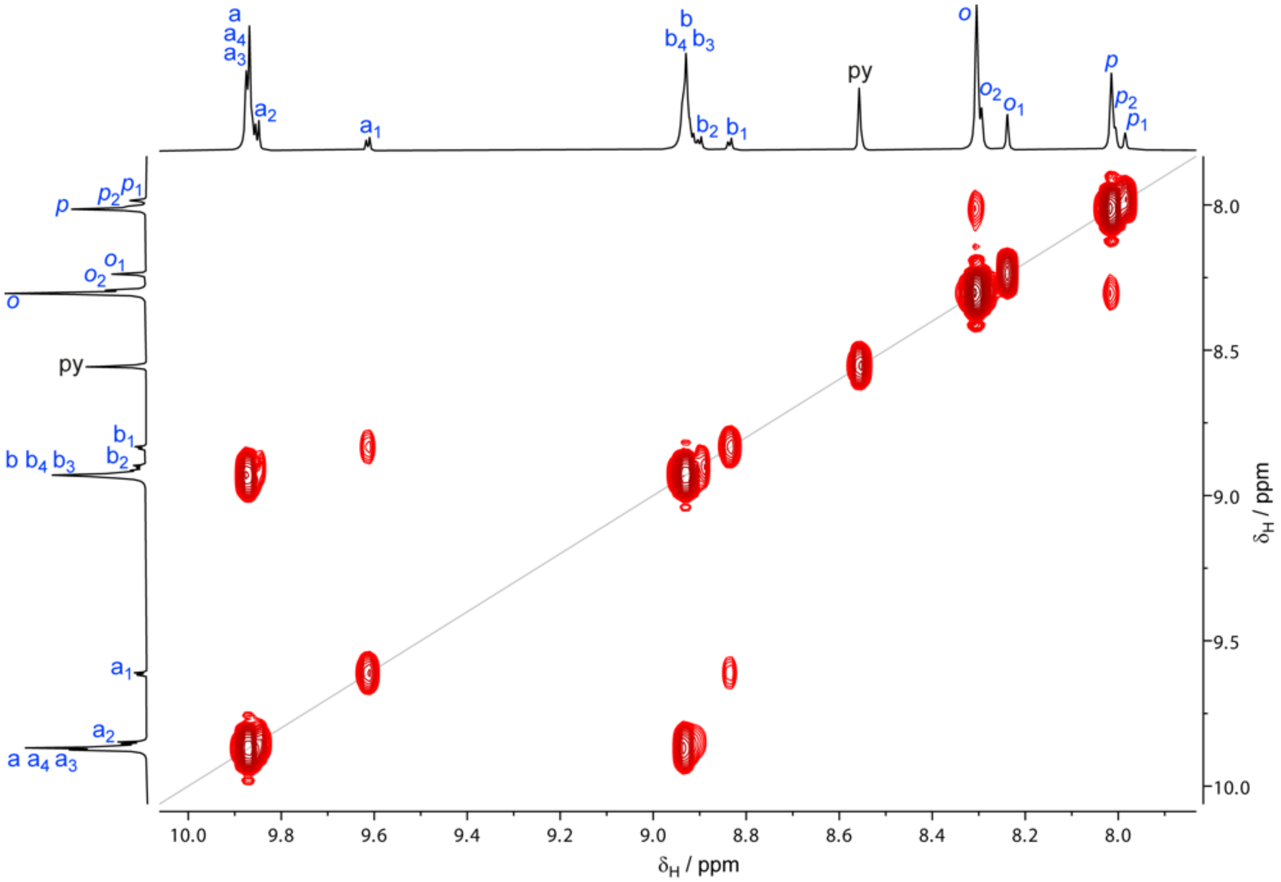

Figure S206. ^{1}H-^{1}H COSY spectrum (aromatic region) of **P15**$_{\mathbf{THS}}$ (600 MHz, $CDCl_3$, d_5-pyridine, 298 K). * = $CHCl_3$; py = pyridine.

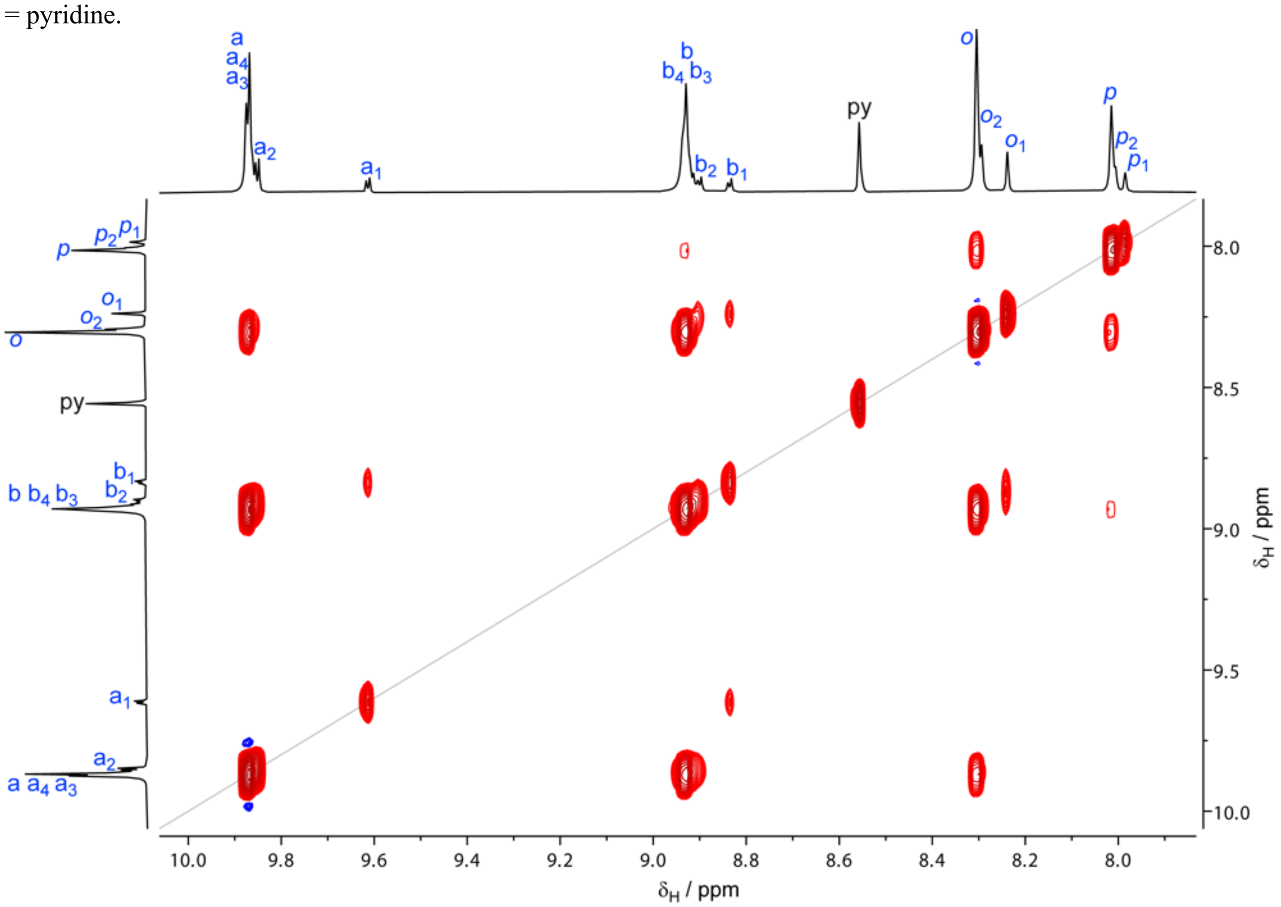

Figure S207. ^{1}H-^{1}H NOESY spectrum (aromatic region) of **P15**$_{\mathbf{THS}}$ (600 MHz, $CDCl_3$, d_5-pyridine, 298 K). * = $CHCl_3$; py = pyridine.

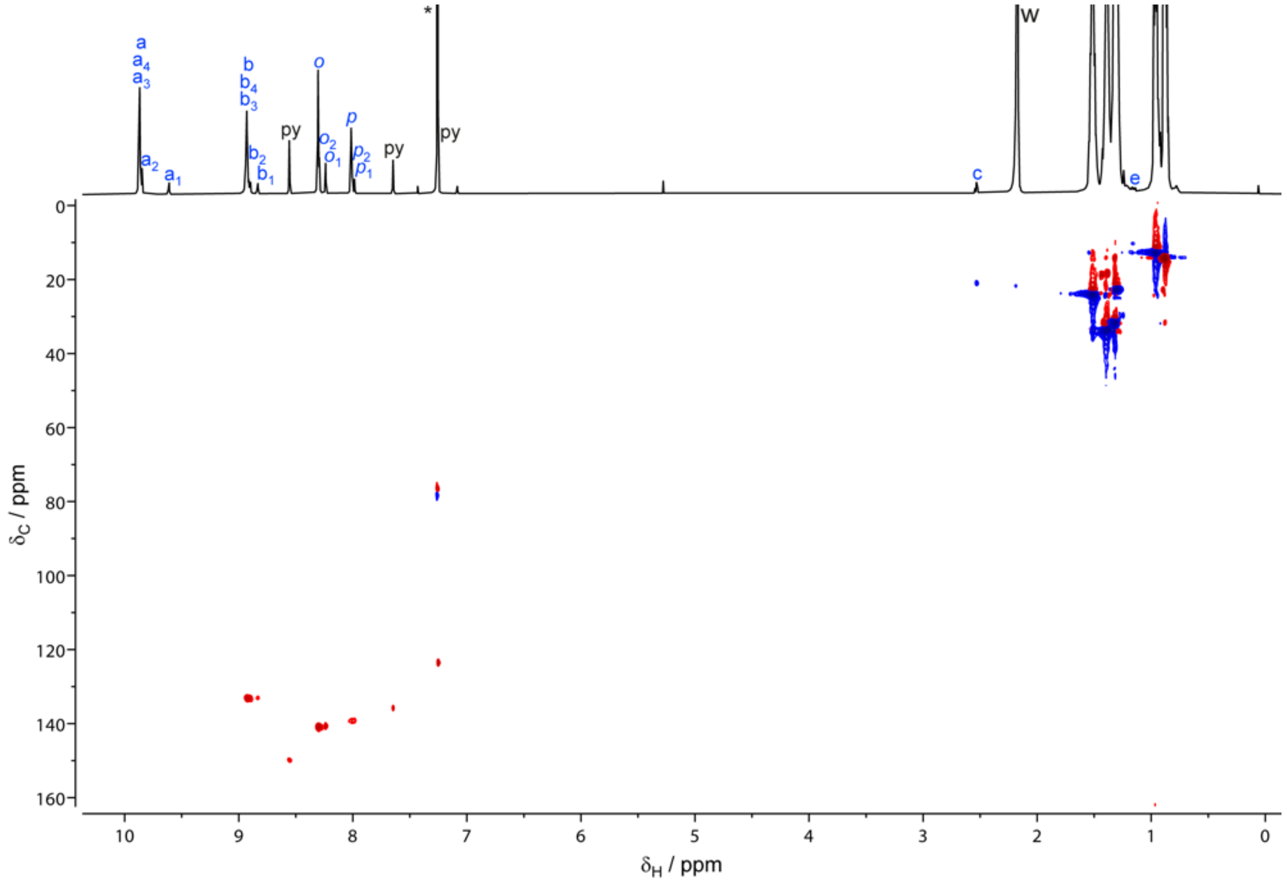

Figure S208. ^{1}H-^{13}C HSQC spectrum of **P15THS** (600 MHz, $CDCl_3$, *d*5-pyridine, 298 K). * = $CHCl_3$; py = pyridine; w = H_2O.

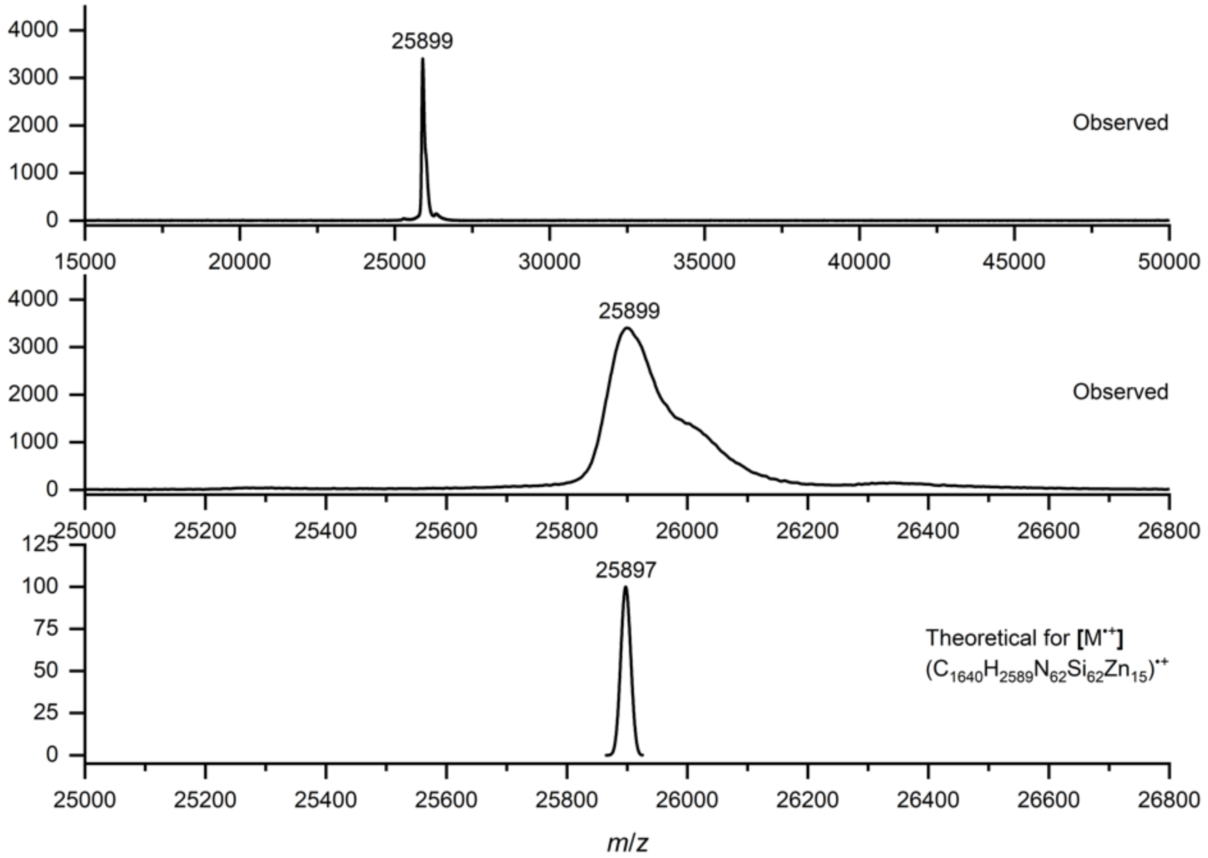

Figure S209. MALDI mass spectrum of **P15THS**.

Spectra of nanorings and nanoring-template complexes

***c*-P18$_{OOct}$**

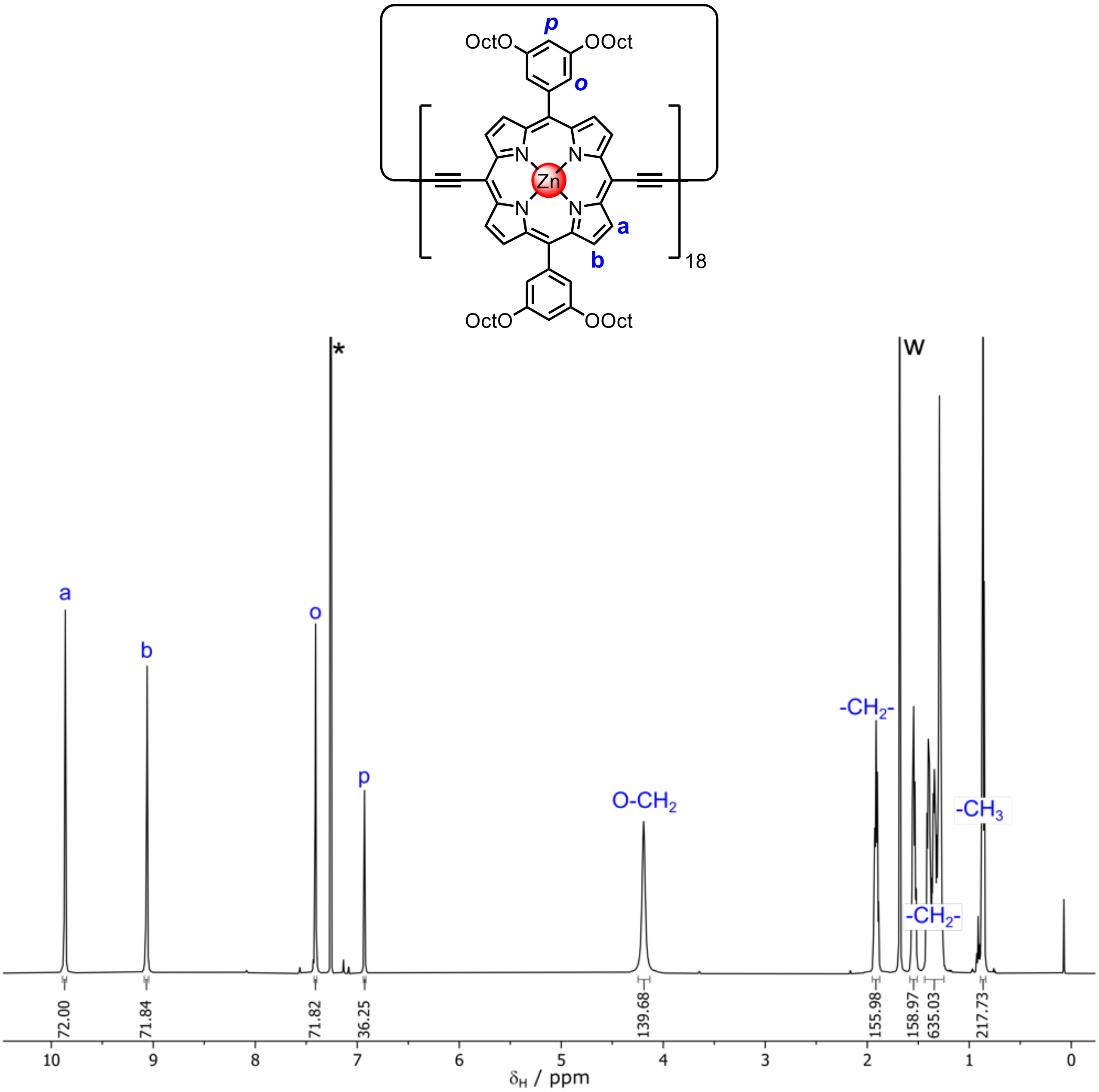

Figure S210. ^{1}H NMR spectrum of ***c*-P18$_{OOct}$** (600 MHz, $CDCl_3$, *d*$_5$-pyridine, 298 K). * = $CHCl_3$; w = water.

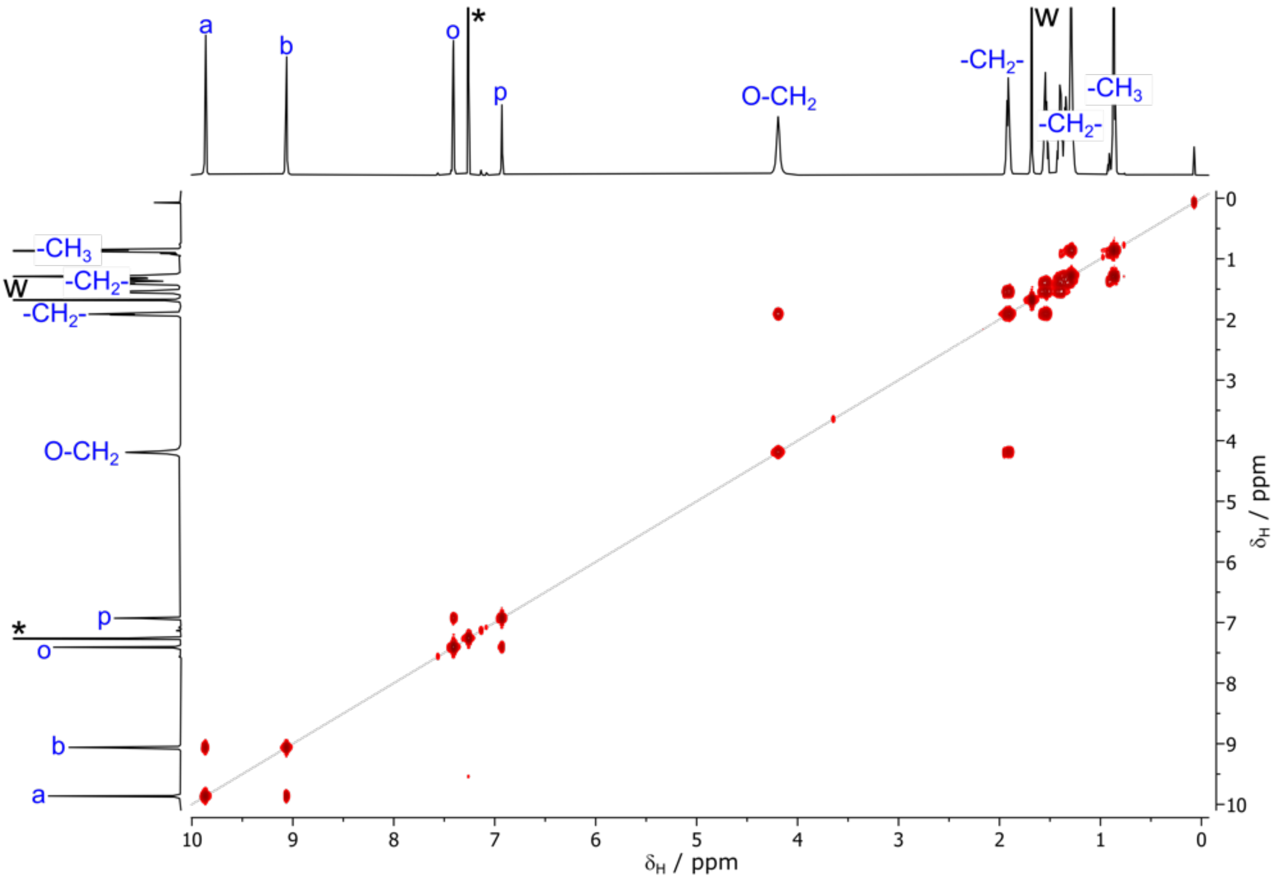

Figure S211. ^{1}H-^{1}H COSY spectrum of ***c*-P18**$_{\mathbf{OOct}}$ (600 MHz, $CDCl_3$, *d*$_5$-pyridine, 298 K). * = $CHCl_3$; w = water

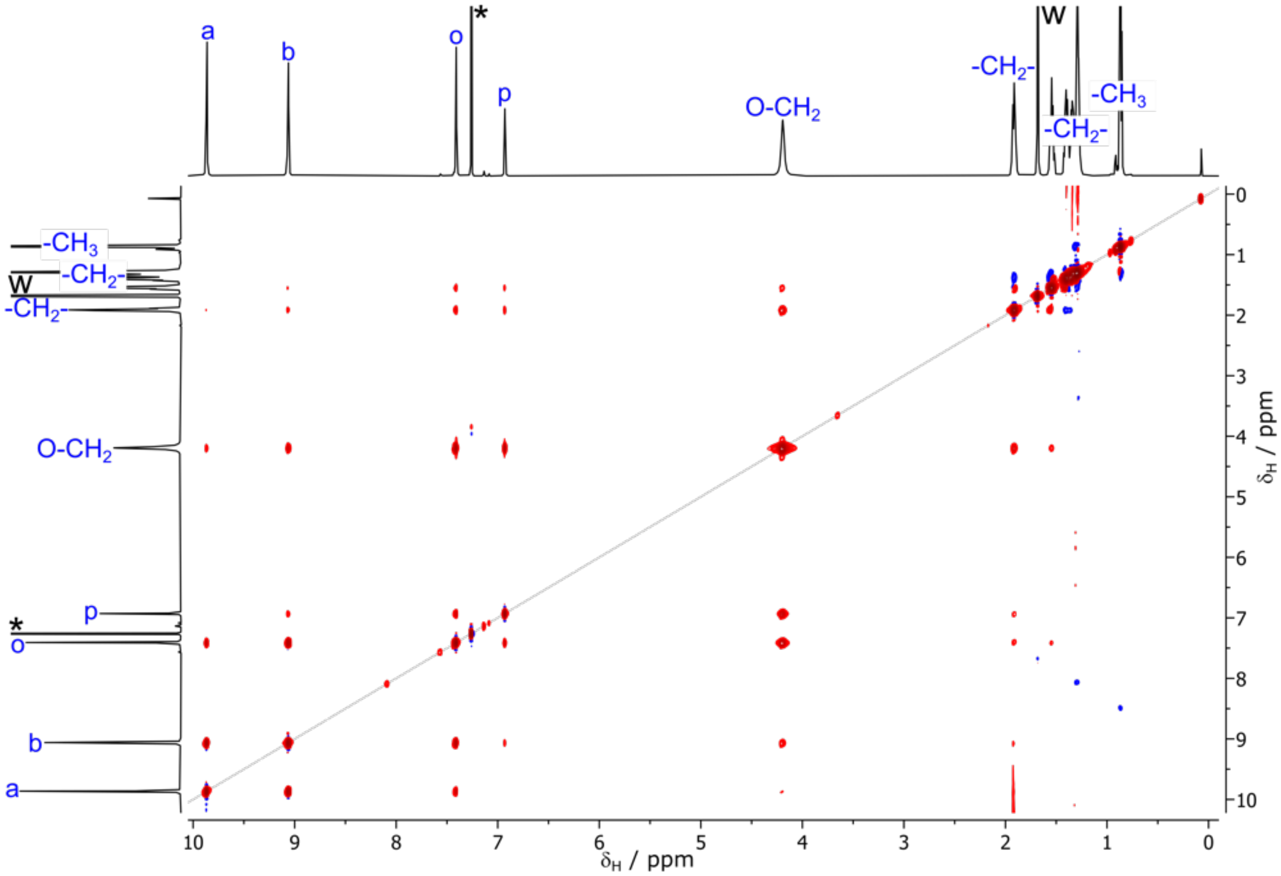

Figure S212. ^{1}H-^{1}H NOESY spectrum of ***c*-P18**$_{\mathbf{OOct}}$ (600 MHz, $CDCl_3$, *d*$_5$-pyridine, 298 K). * = $CHCl_3$.

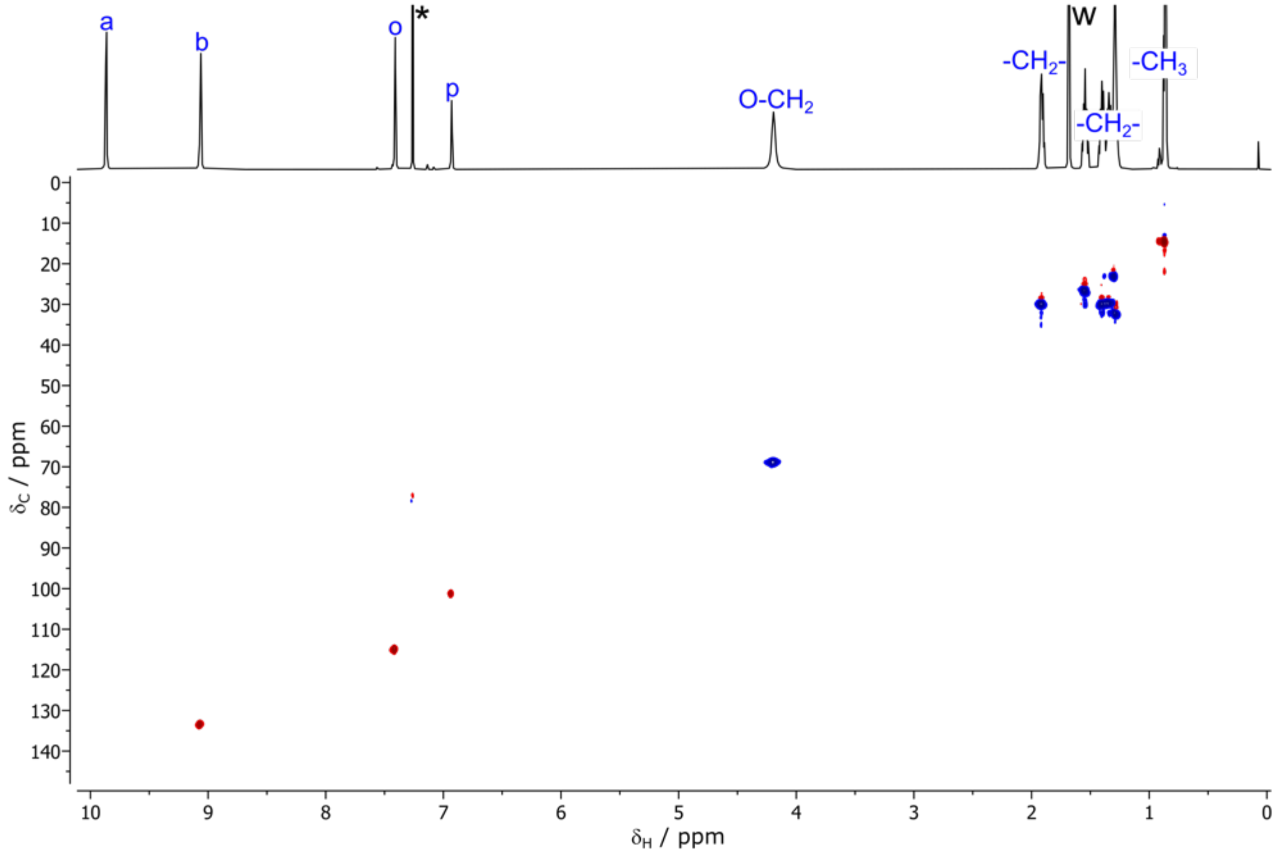

Figure S213. ^{1}H-^{13}C HSQC spectrum of ***c*-P18$_{OOct}$** (600 MHz, $CDCl_3$, *d*$_5$-pyridine, 298 K). * = $CHCl_3$; w = water.

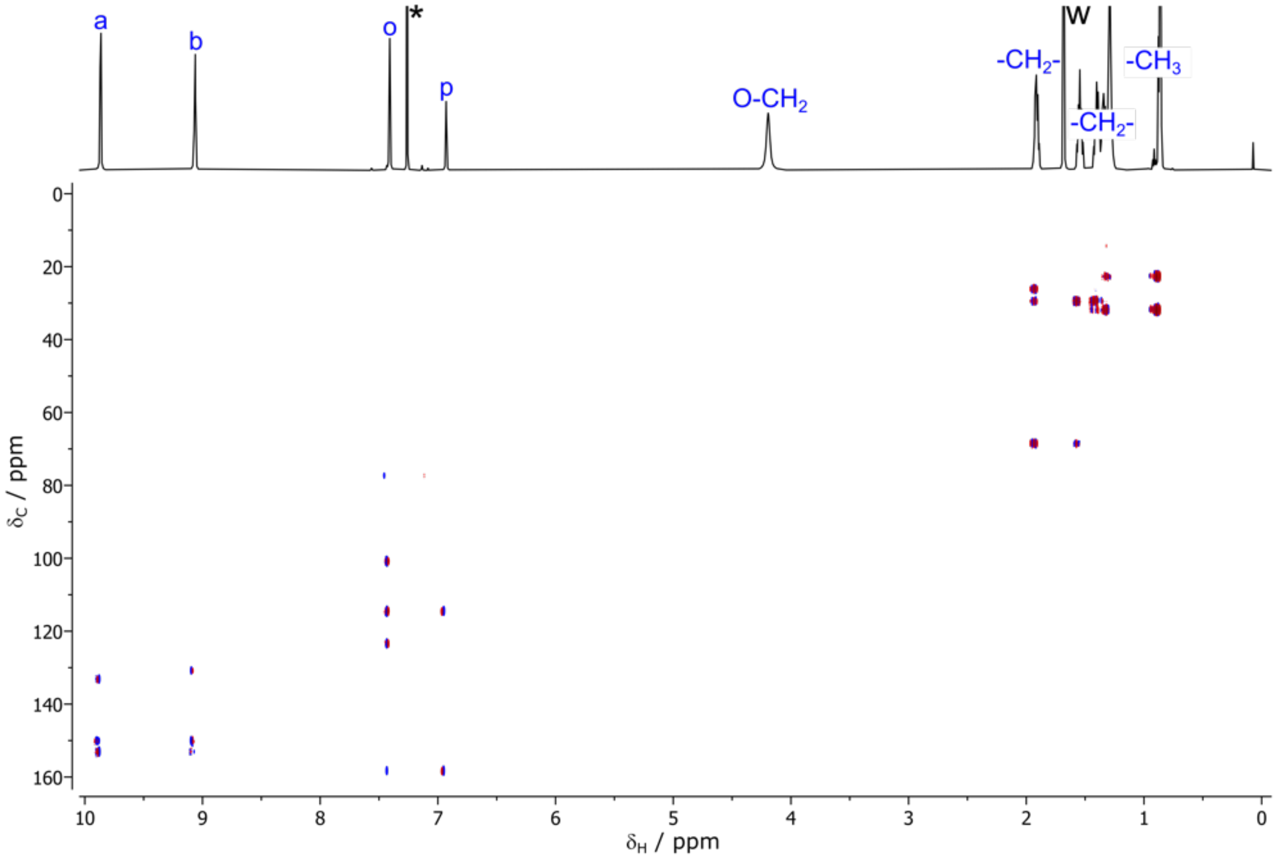

Figure S214. ^{1}H-^{13}C HMBC spectrum of ***c*-P18$_{OOct}$** (600 MHz, $CDCl_3$, *d*$_5$-pyridine, 298 K). * = $CHCl_3$; w = water.

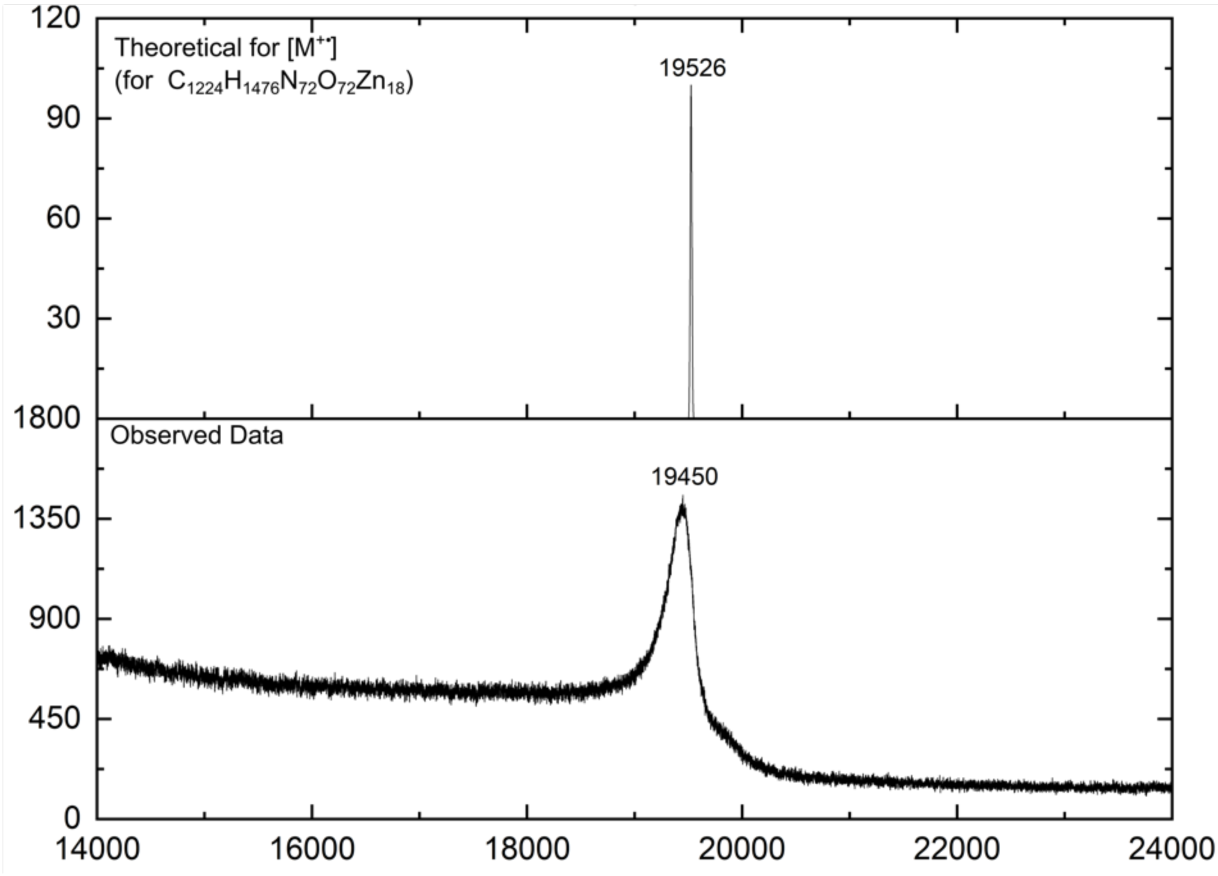

Figure S215. MALDI mass spectrum of ***c*-P18$_{\mathbf{OOct}}$** (matrix: dithranol)

***c*-P18THS**

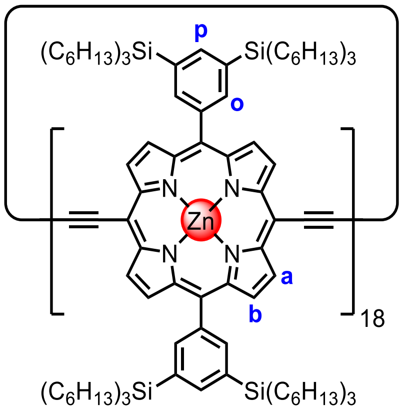

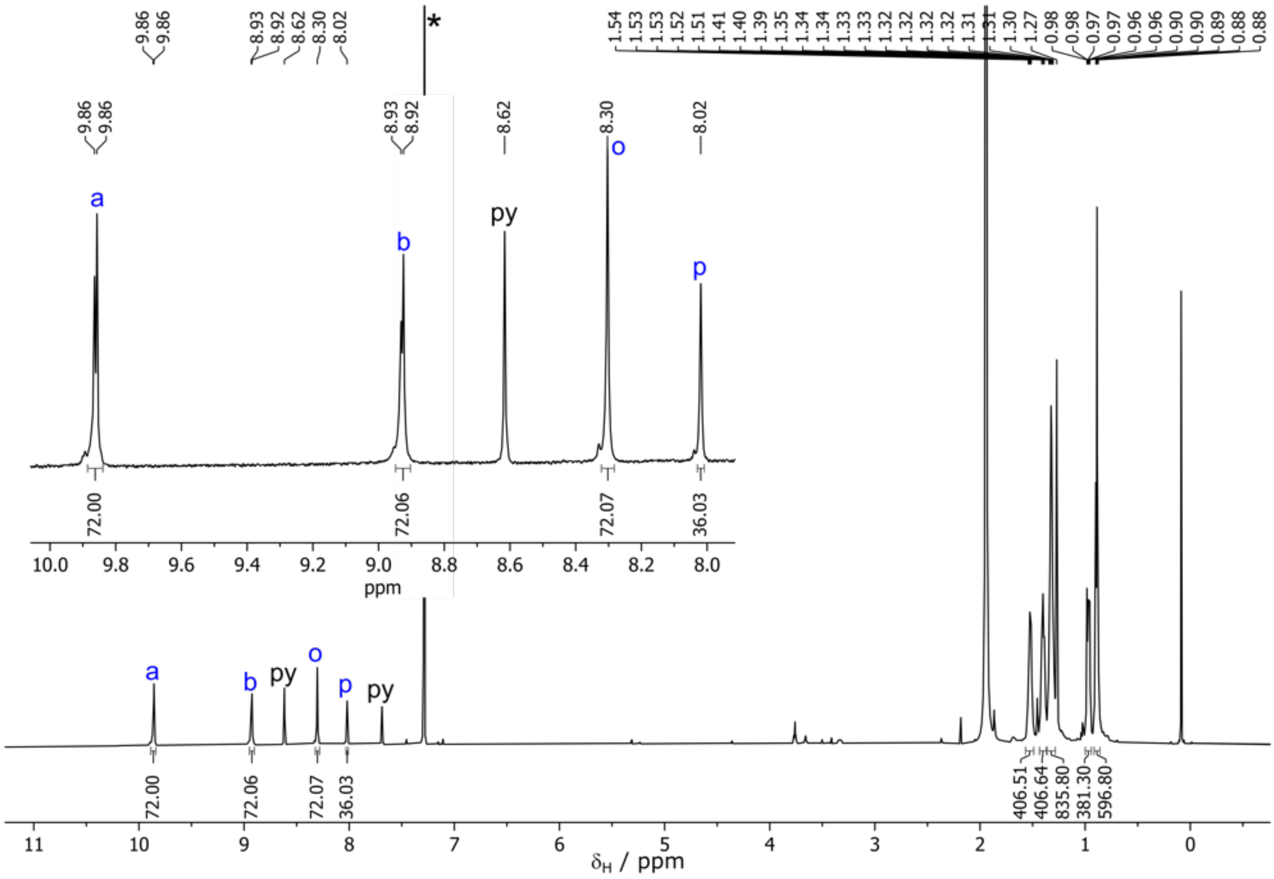

Figure S2106. ^{1}H NMR spectrum of ***c*-P18THS** (600 MHz, $CDCl_3$, *d*5-pyridine, 298 K). * = $CHCl_3$; py = pyridine.

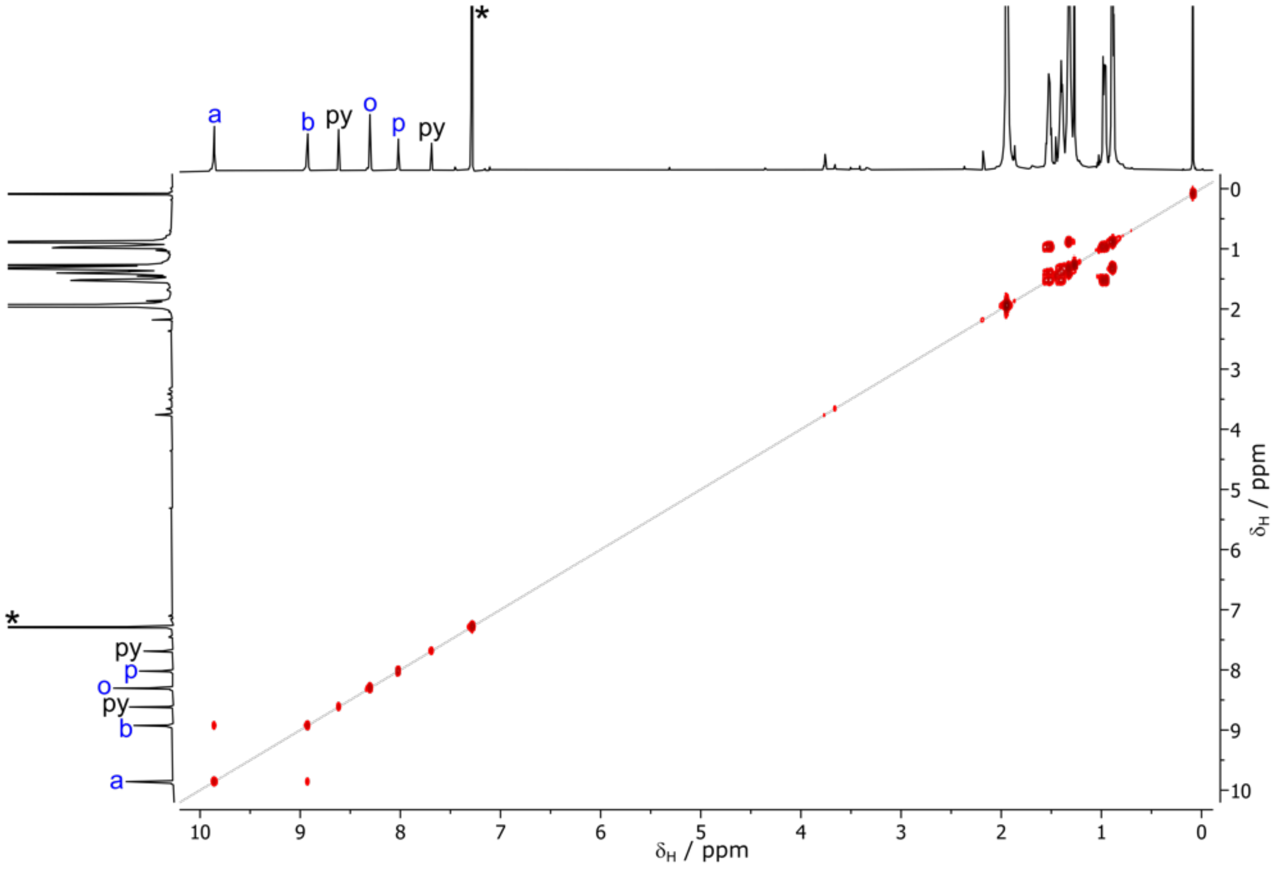

Figure S217. ^{1}H-^{1}H COSY spectrum of ***c*-P18$_{THS}$** (600 MHz, $CDCl_3$, d_5-pyridine, 298 K). * = $CHCl_3$; py = pyridine.

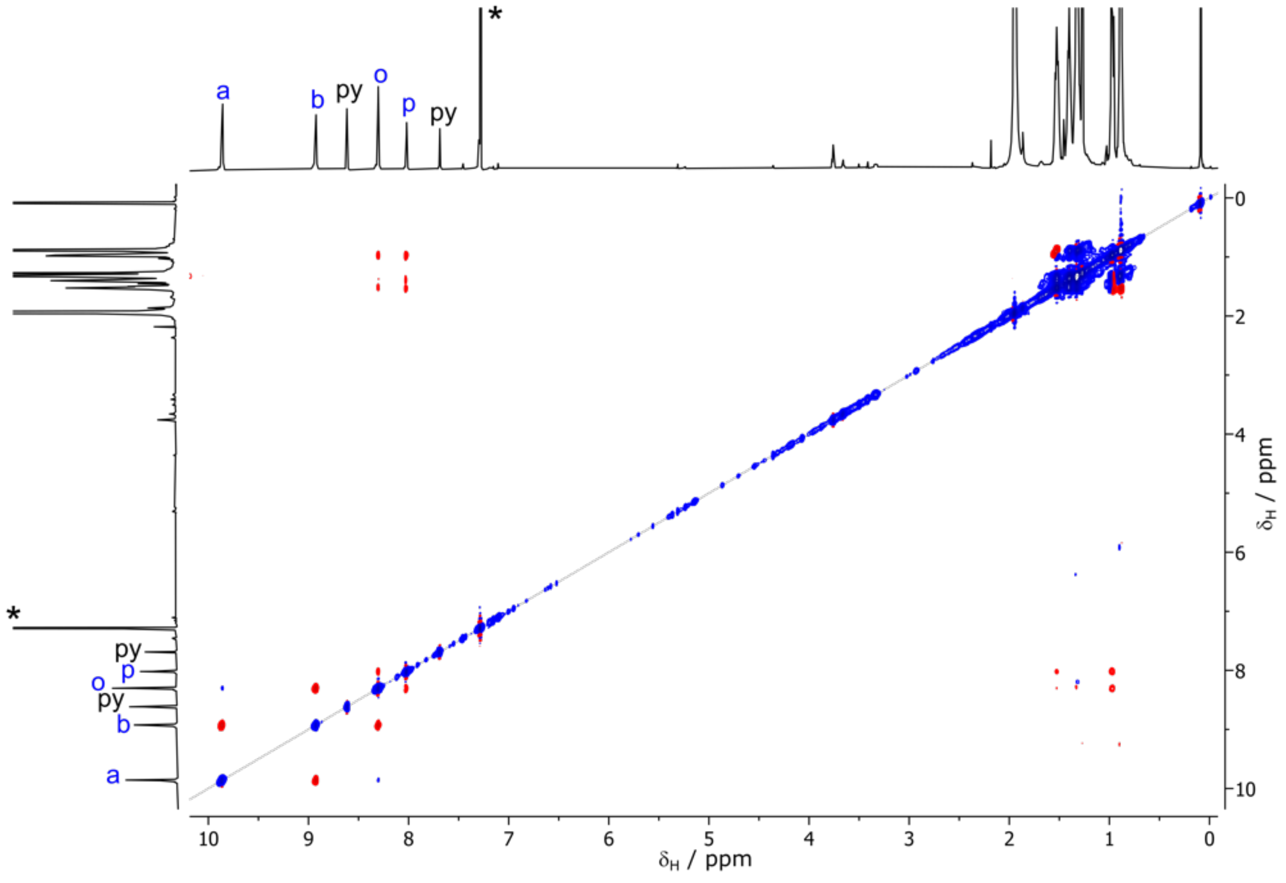

Figure S218. ^{1}H-^{1}H ROESY spectrum of ***c*-P18$_{THS}$** (600 MHz, $CDCl_3$, d_5-pyridine, 298 K). * = $CHCl_3$; py = pyridine.

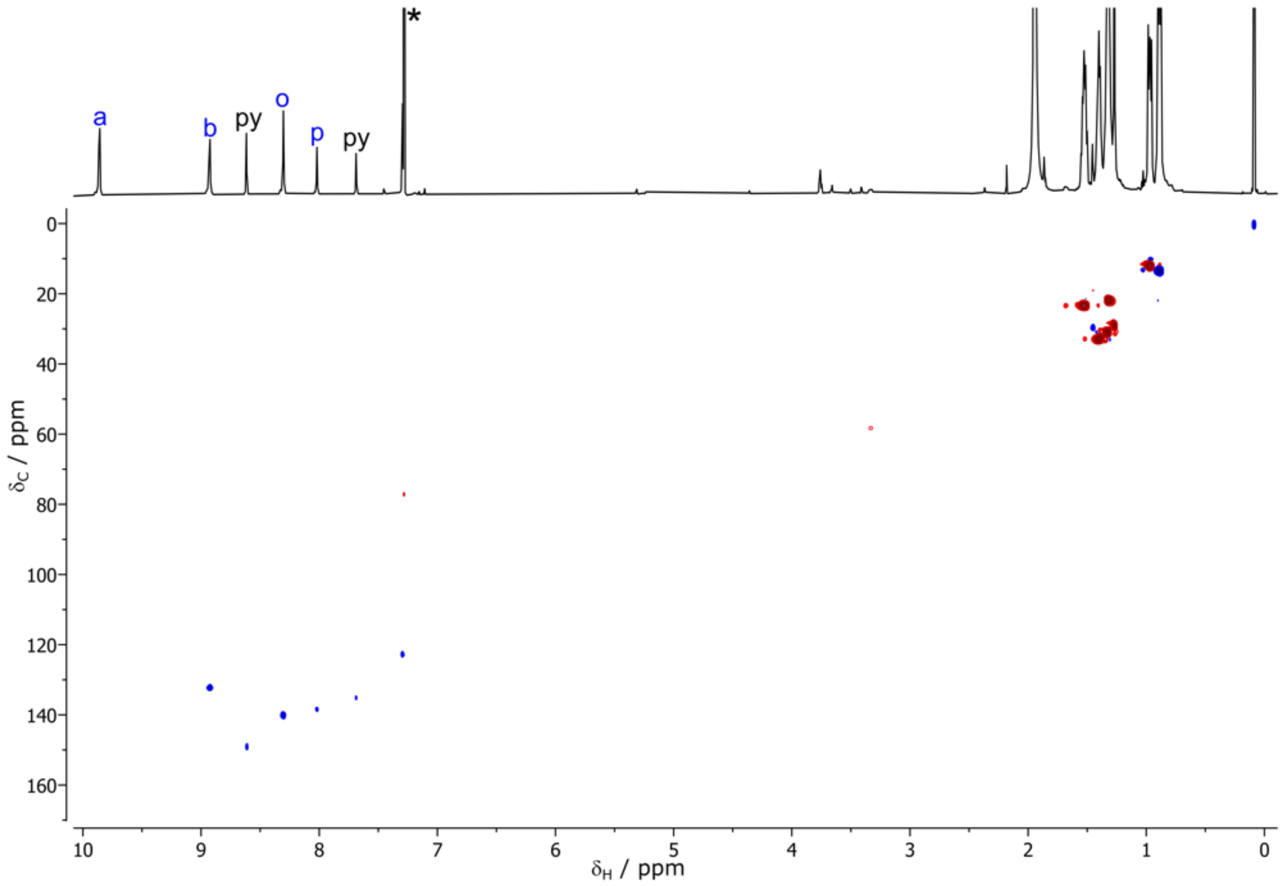

Figure S219. ^{1}H-^{13}C HSQC spectrum of ***c*-P18**$_{THS}$ (600 MHz, $CDCl_3$, d_5-pyridine, 298 K). * = $CHCl_3$; py = pyridine.

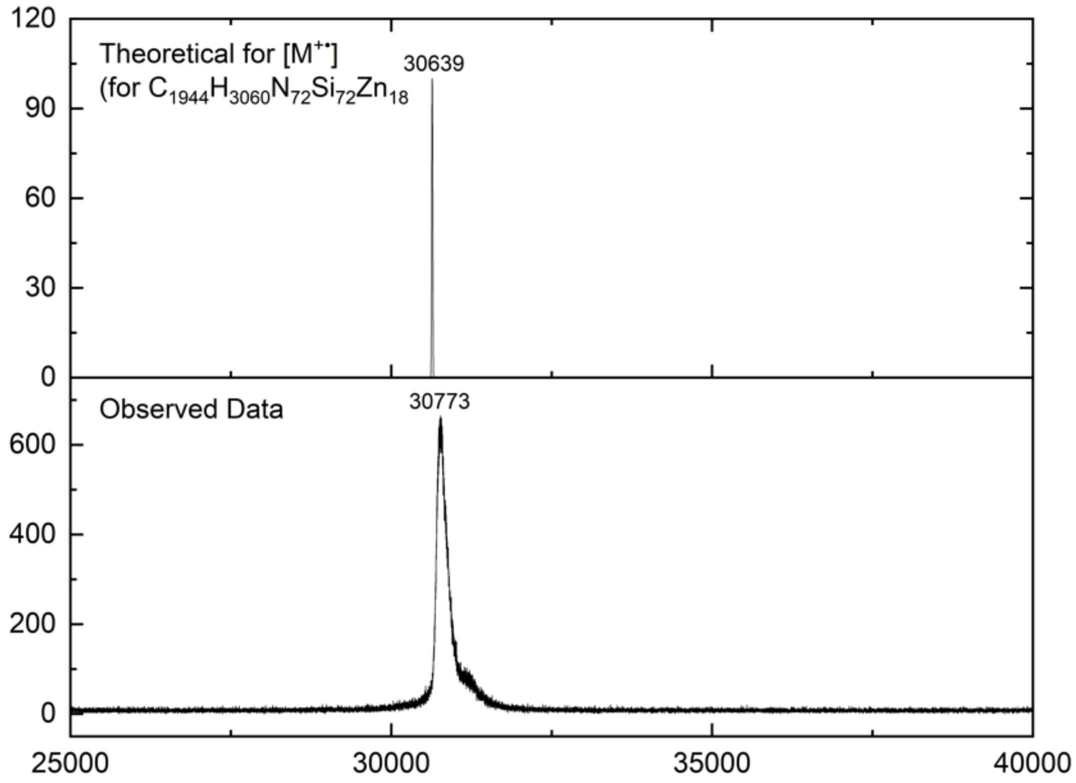

Figure S220. MALDI mass spectrum of ***c*-P18**$_{THS}$ (dithranol matrix)

***c*-P18$_{OOct}$·T18$_A$**

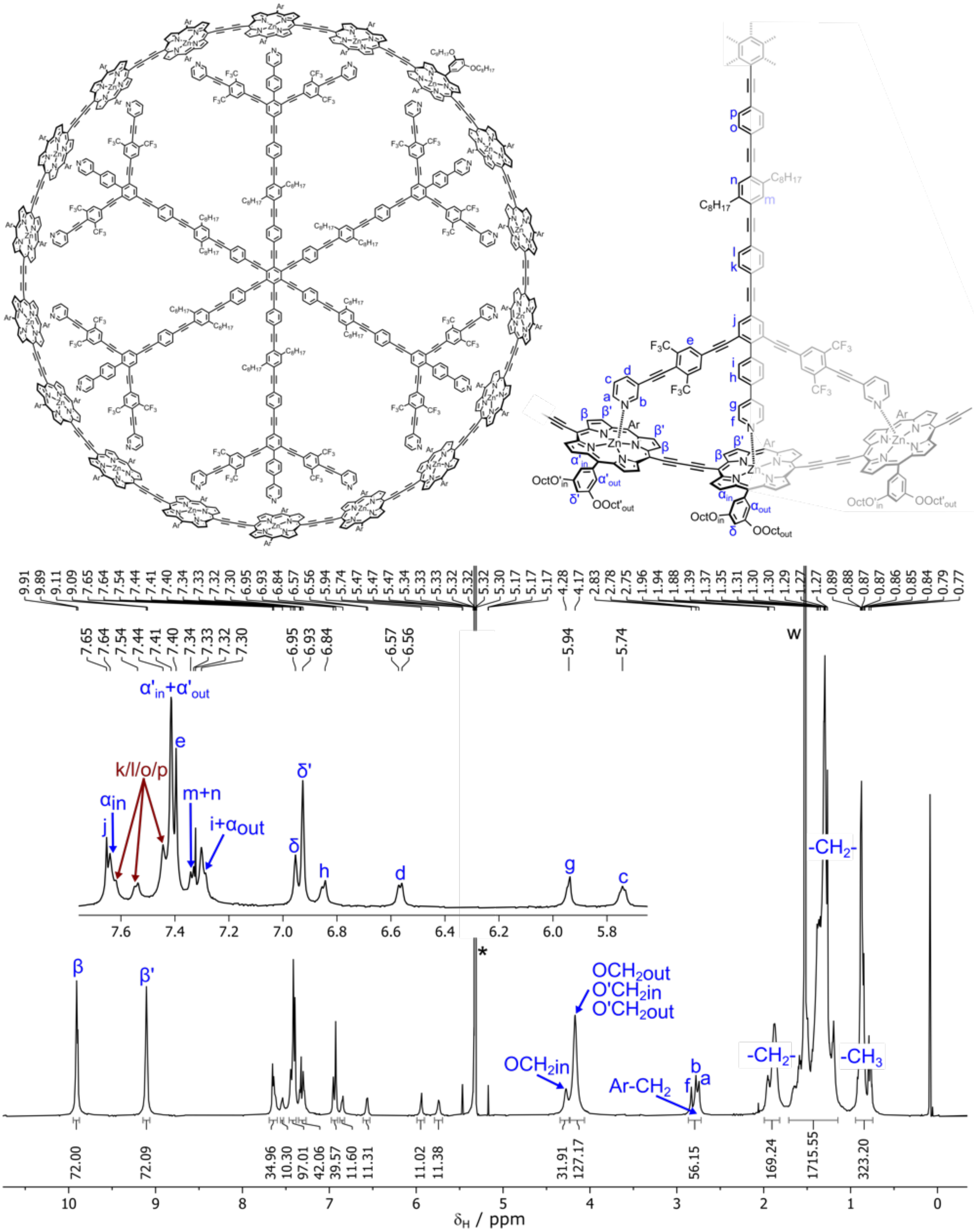

Figure S221. ^{1}H NMR spectrum of ***c*-P18$_{OOct}$·T18$_A$** (600 MHz, CD_2Cl_2, 298 K). * = $CHDCl_2$; w = water.

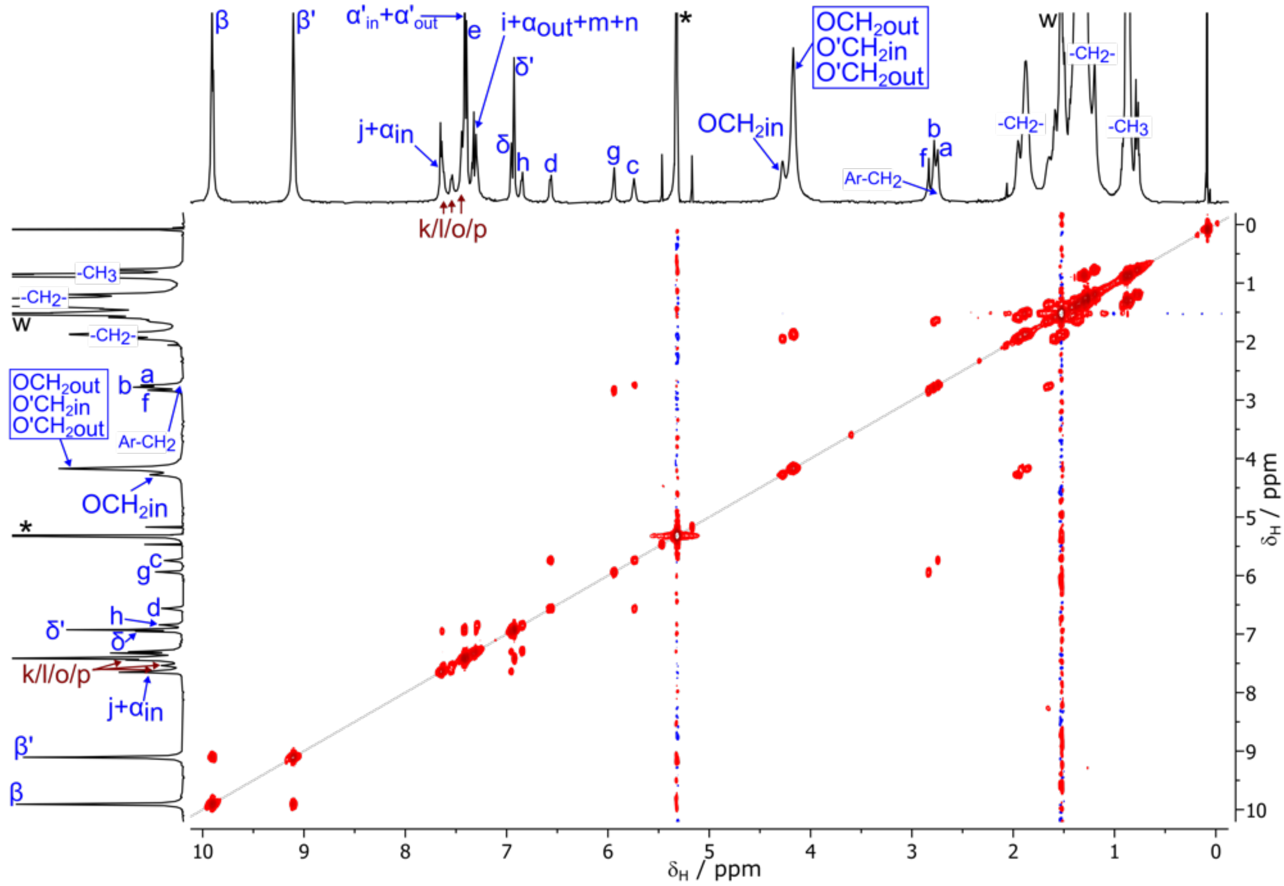

Figure S222. ^{1}H-^{1}H COSY spectrum of ***c*-P18**$_{\mathbf{OOct}}$**·T18**$_{\mathbf{A}}$ (600 MHz, CD_2Cl_2, 298 K). * = $CHDCl_2$; w = water.

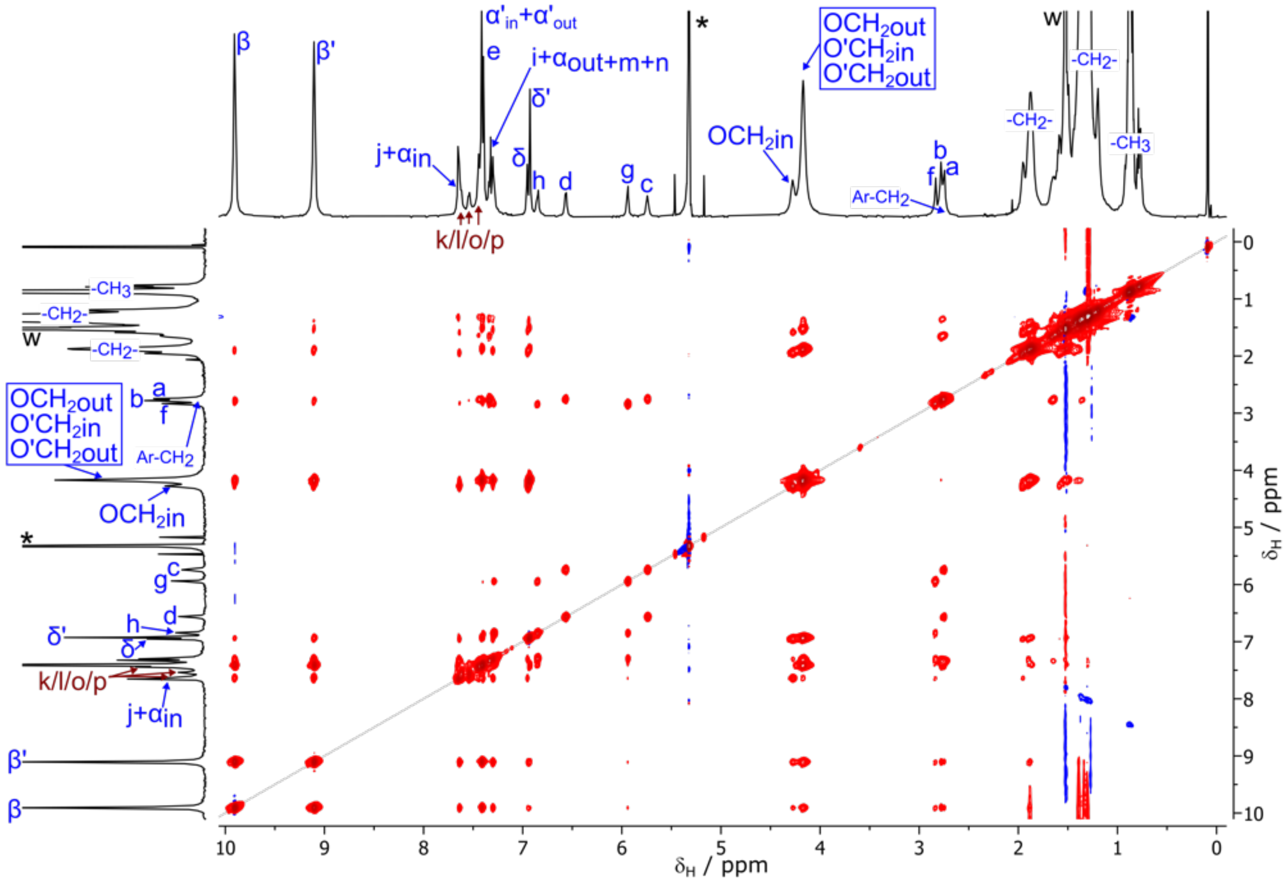

Figure S223. ^{1}H-^{1}H NOESY spectrum of ***c*-P18**$_{\mathbf{OOct}}$**·T18**$_{\mathbf{A}}$ (600 MHz, CD_2Cl_2, 298 K). * = $CHDCl_2$; w = water.

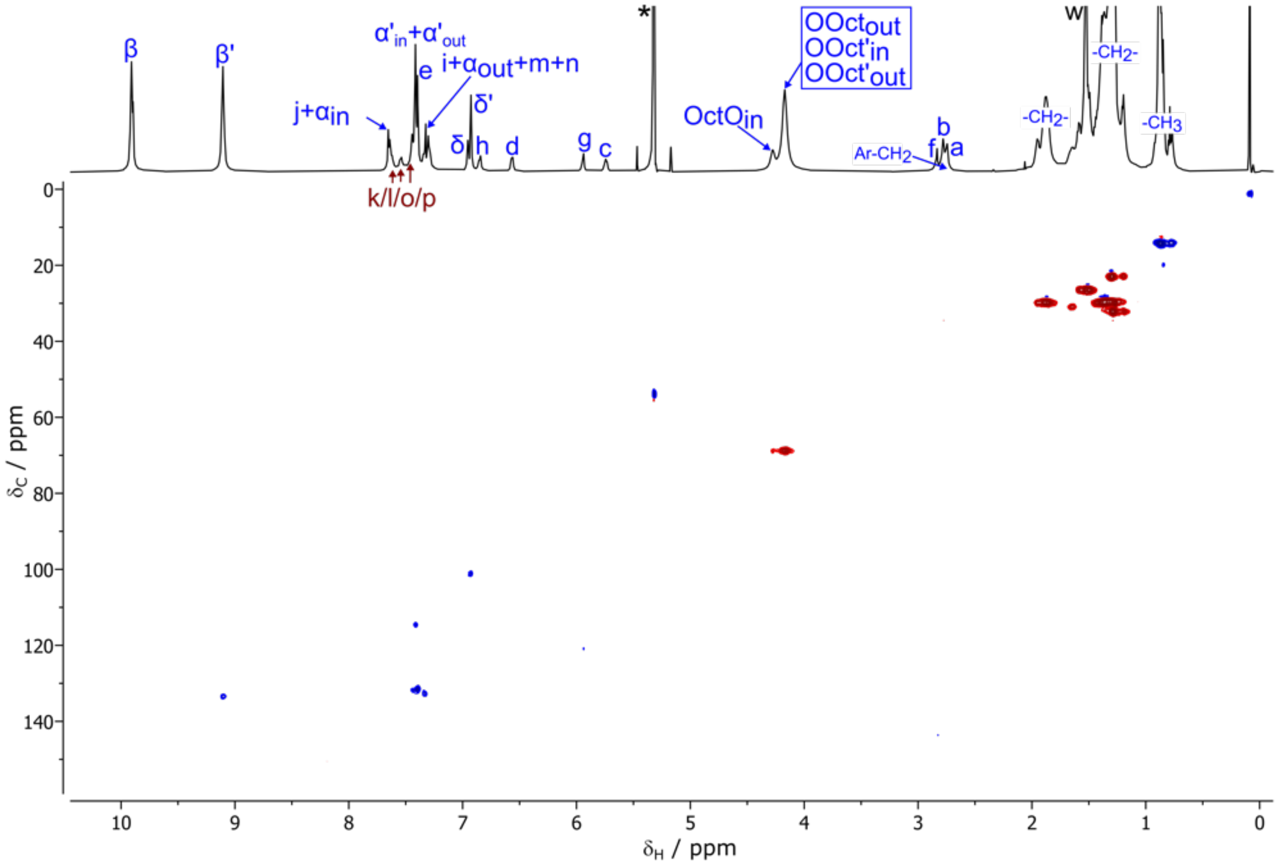

Figure S224. ^{1}H-^{13}C HSQC spectrum of ***c*-P18$_{OOct}$·T18$_A$** (600 MHz, CD_2Cl_2, 298 K). * = $CHDCl_2$; w = water.

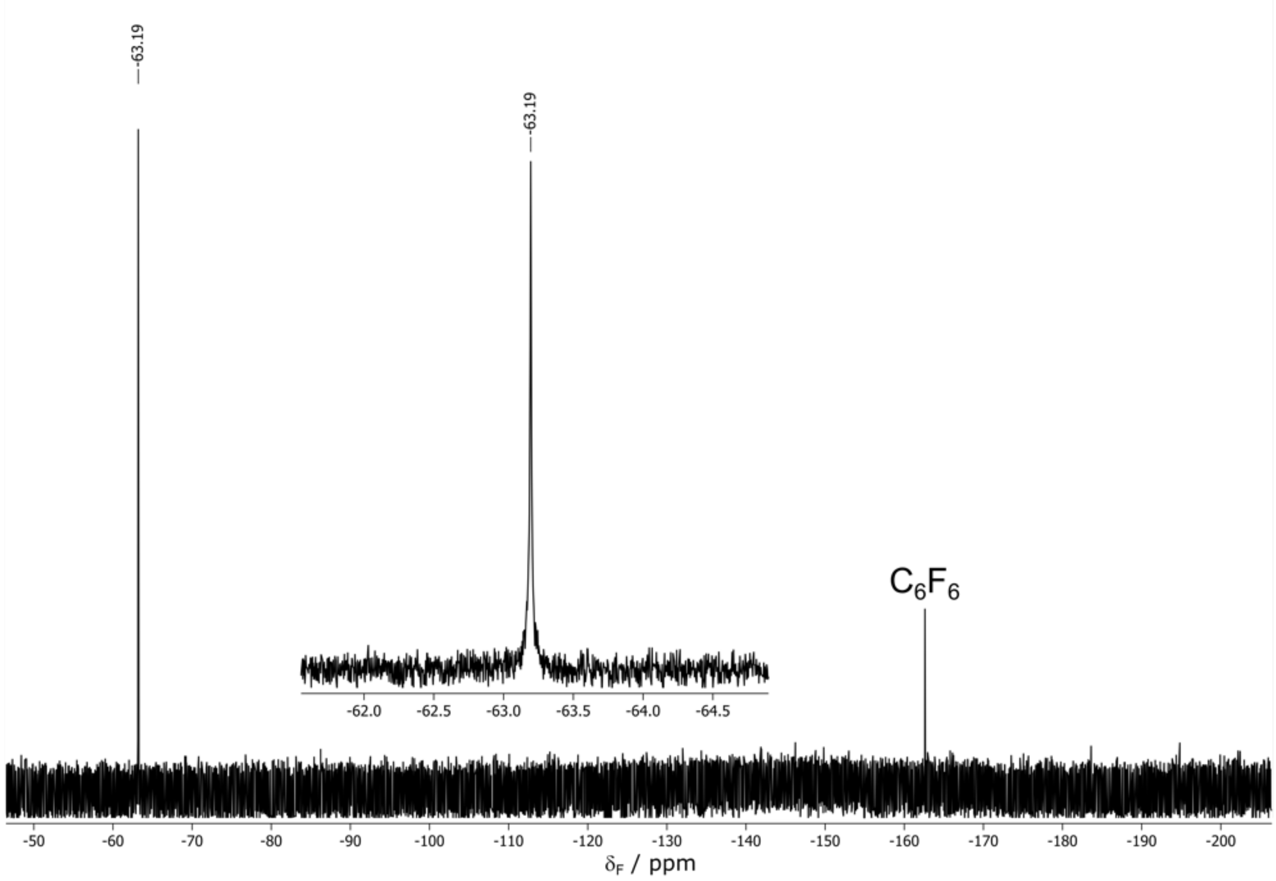

Figure S225. ^{19}F NMR spectrum of ***c*-P18$_{OOct}$·T18$_A$** (471 MHz, CD_2Cl_2, 298 K). Referenced against C_6F_6 (δ_F = −162.61) as an internal standard.

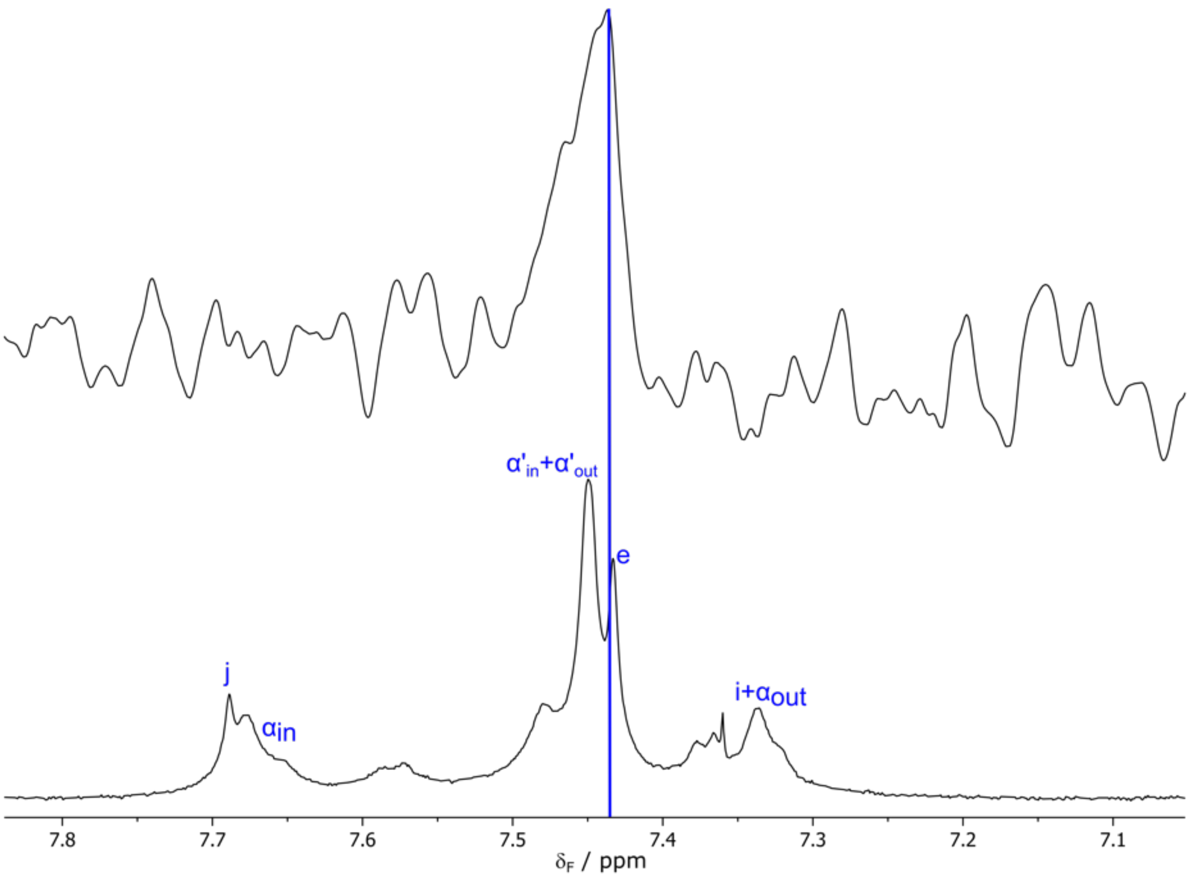

Figure S226. ^{1}H-^{19}F HOESY (top) and ^{1}H (bottom) NMR spectrum of ***c*-P18$_{OOct}$·T18$_A$** (500 MHz, CD_2Cl_2, 298 K).

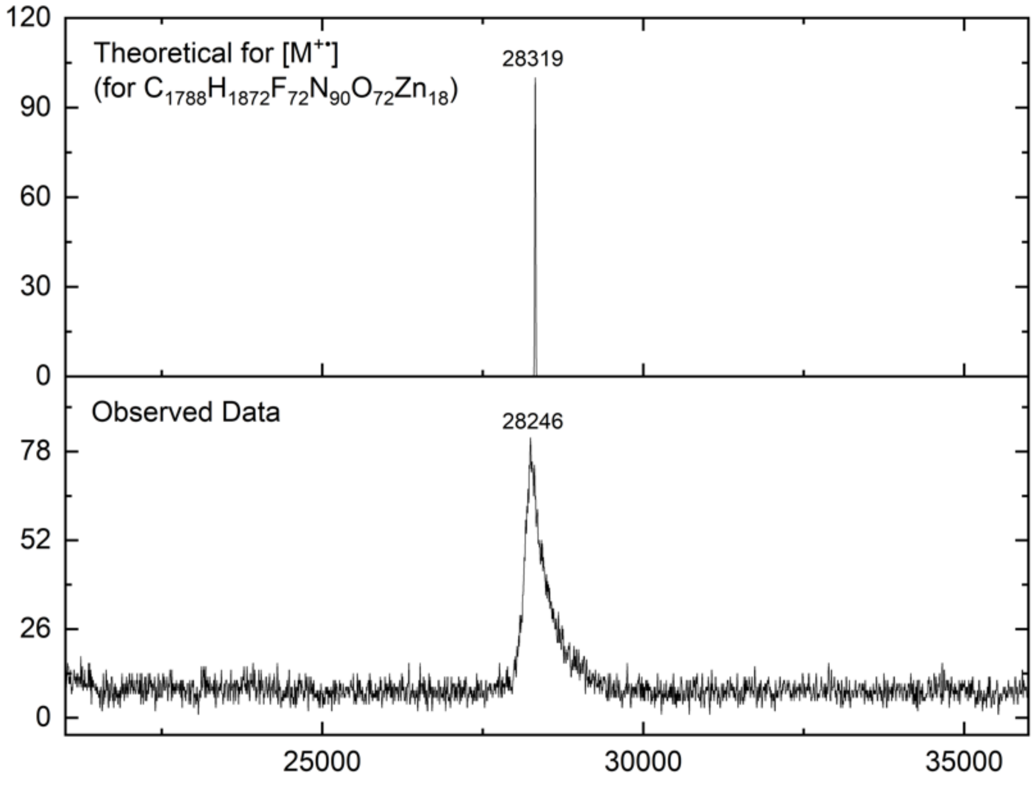

Figure S227. MALDI mass spectrum of ***c*-P18$_{OOct}$·T18$_A$** (dithranol matrix)

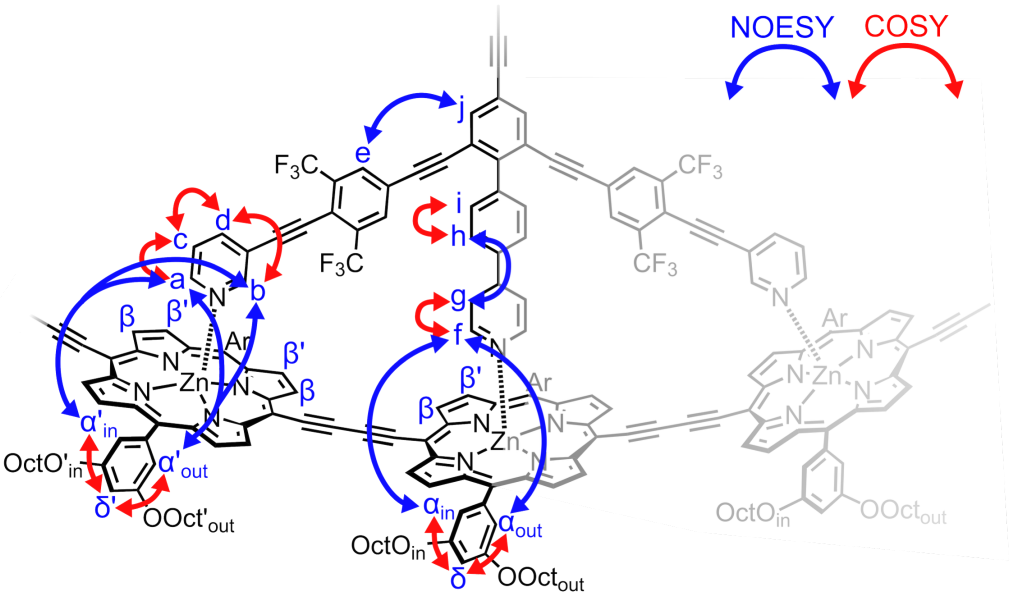

	a	b	c	d	e	f	g	h	i	j	k	l	m	n	o	p	α_{out}	α_{in}	α'_{out}	α'_{in}	δ	δ'	β	β'	$OOct_{in}$	OOct' + $OOct_{out}$	Ar-CH_2	Ar-CH_2-**CH_2**
a	■		m																									
b		■		w																								
c	m		■	m																								
d			s	■																								
e					■																							
f						■	m																					
g						m	■	w																				
h					w	m	m	■	m																			
i						w	m	s	■																			
j					w					■																		
k											■																	
l												■																
m													■															
n														■														
o															■	m												
p																■												
α_{out}						w											■	w			m							
α_{in}						w	w										m	■			m							
α'_{out}	m	m																	■			m						
α'_{in}	m	m																		■		m						
δ																	m	m			■							
δ'																			m	m		■						
β	w	m				w											m	m	m	m			■	s				
β'	w	m				w											m	m	m	m			s	■				
OCH_{2in}																		m			m			w	■			
$O'CH_{2in}$+OCH_{2out}																	m		s	s	m	s	m	m		■		
Ar-CH_2													s	s													■	m
Ar-CH_2-**CH_2**													m	m														■

Table S4. Summary of COSY (above diagonal) and NOESY (below diagonal) correlations in ***c*-P18$_{OOct}$·T18$_A$** complex. vw; very weak; w: weak; mw: medium weak; m: medium; s: strong

Explanation of Assignment

- β protons at characteristic chemical shift and correct integration. β vs β' assigned as proton closest to acetylene always has highest chemical shift. β assigned.
- Strongest NOESY correlation to β protons come from three peaks at 7.29, 7.41 and 7.63 ppm. Expect these to be *ortho* protons – tentatively assign.
- Next strongest NOESY correlations to β protons come from the multiplet at 2.70–2.87 ppm. Expect these to be protons a, b and f. These three signals have COSY to protons at 5.74, 5.94 and 6.56 ppm, which are expected to be c, d and h. (a couples to c, b couples to d, and f couples to g)
- Protons c and d should have COSY correlation – peaks at 5.74 and 6.56 do, so can work back to assign a,b,c,d,f,g
- g has weak COSY to peak at 6.84 and medium NOESY to 6.84 and 7.29. Can assign 6.84 as h and 7.29 as i.
- ^{1}H-^{19}F HOESY has only one signal which corresponds to proton e at 7.40 ppm.
- e has weak NOESY correlation to peak at 7.65 – probably j.
- *Ortho* protons tentatively assigned earlier as peaks at 7.29, 7.41 and 7.63 ppm. Peaks at 7.29 and 7.63 have NOESY to proton f (not a or b) so probably *ortho* protons from central porphyrin (α_{in} + α_{out}). The peak at 7.63 has an additional NOESY correlation to proton g, so is probably the *ortho* proton pointing towards the center of the nanoring (α_{in}). Assign 7.29 as α_{out} and 7.63 as α_{in}.
- With the same logic the peak at 7.41 has NOESY to a and b (not f) so is the *ortho* protons from the side porphyrins (α'_{in} + α'_{out}).
- δ and δ' are assigned by strong COSY to respective *ortho* protons (δ to α_{in} + α_{out}) and (δ' to α'_{in} + α'_{out})
- OCH_2 peak at 4.10–4.30 ppm is split in 1:5 ratio. Smaller peak has medium NOESY to δ and α_{in}. Larger peak has strong NOESY to δ', α'_{in} + α'_{out} and medium NOESY to δ and α_{out}. Smaller peak can be assigned to first CH_2 of $OOct_{in}$. Larger peak is assigned to first CH_2 in $OOct_{out}$, $O'Oct_{in}$, and $O'Oct_{out}$.
- Ar-CH_2 comes underneath proton a and has strong NOESY to peak at 7.33 ppm. Can therefore assign 7.33ppm as m+n.
- Cannot definitively assign protons k,l,o or p

***c*-P18$_{THS}$·T18$_A$**

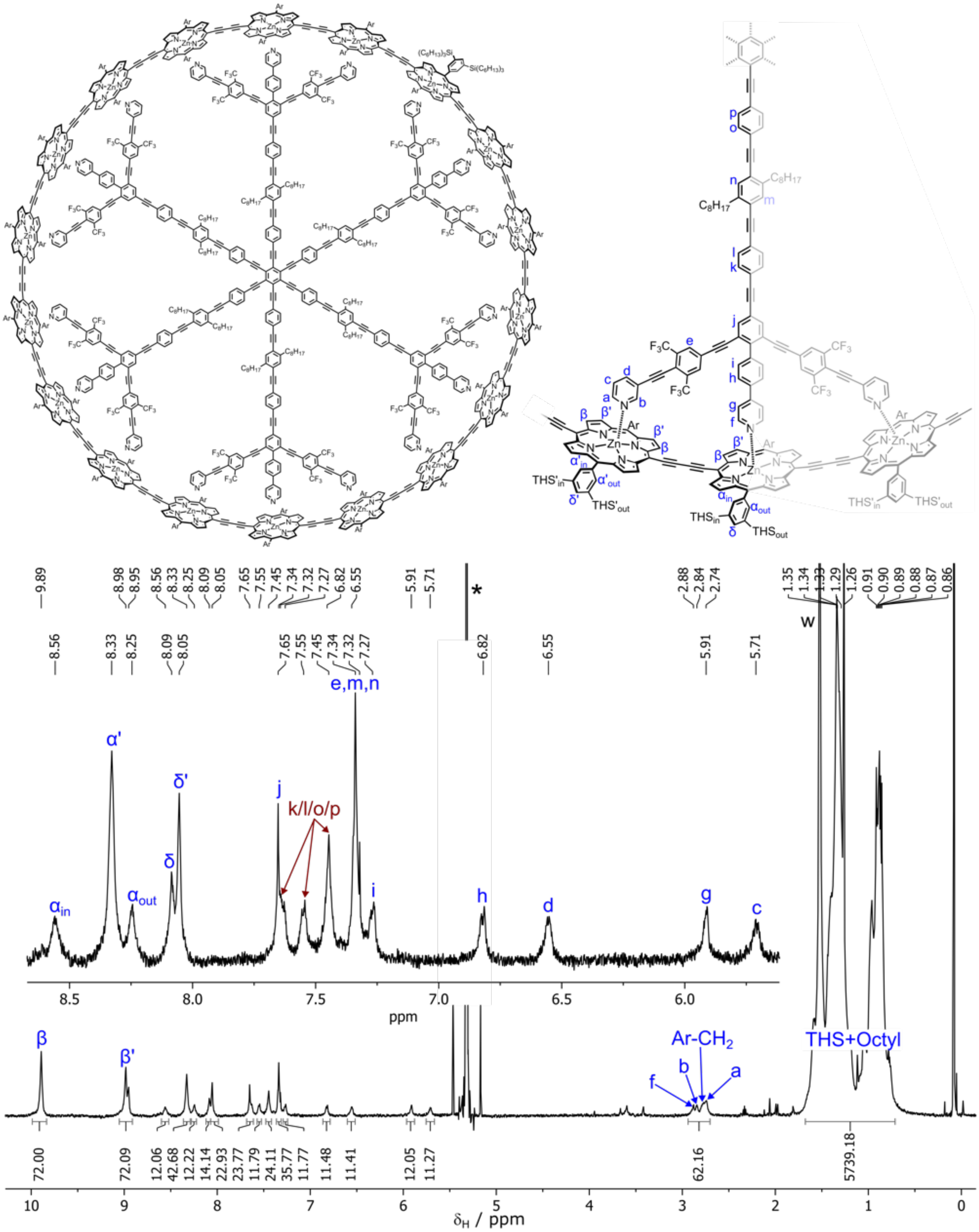

Figure S228. ^{1}H NMR spectrum of ***c*-P18$_{THS}$·T18$_A$** (600 MHz, CD_2Cl_2, 298 K). * = $CHDCl_2$; w = water.

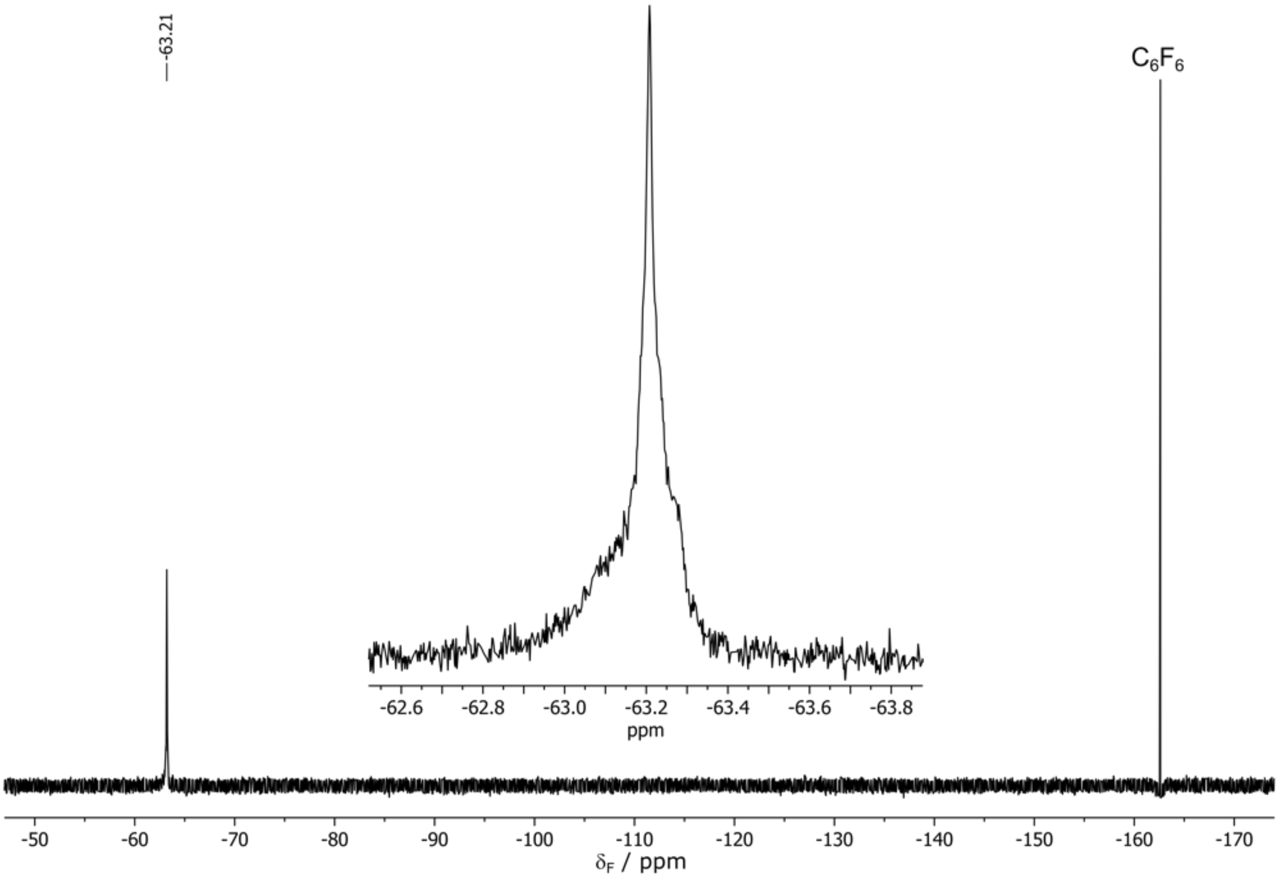

Figure S229. ^{19}F NMR spectrum of ***c*-P18THS·T18A** (470 MHz, CD_2Cl_2, 298 K). Referenced against C_6F_6 (δ_F = −162.61) as an internal standard.

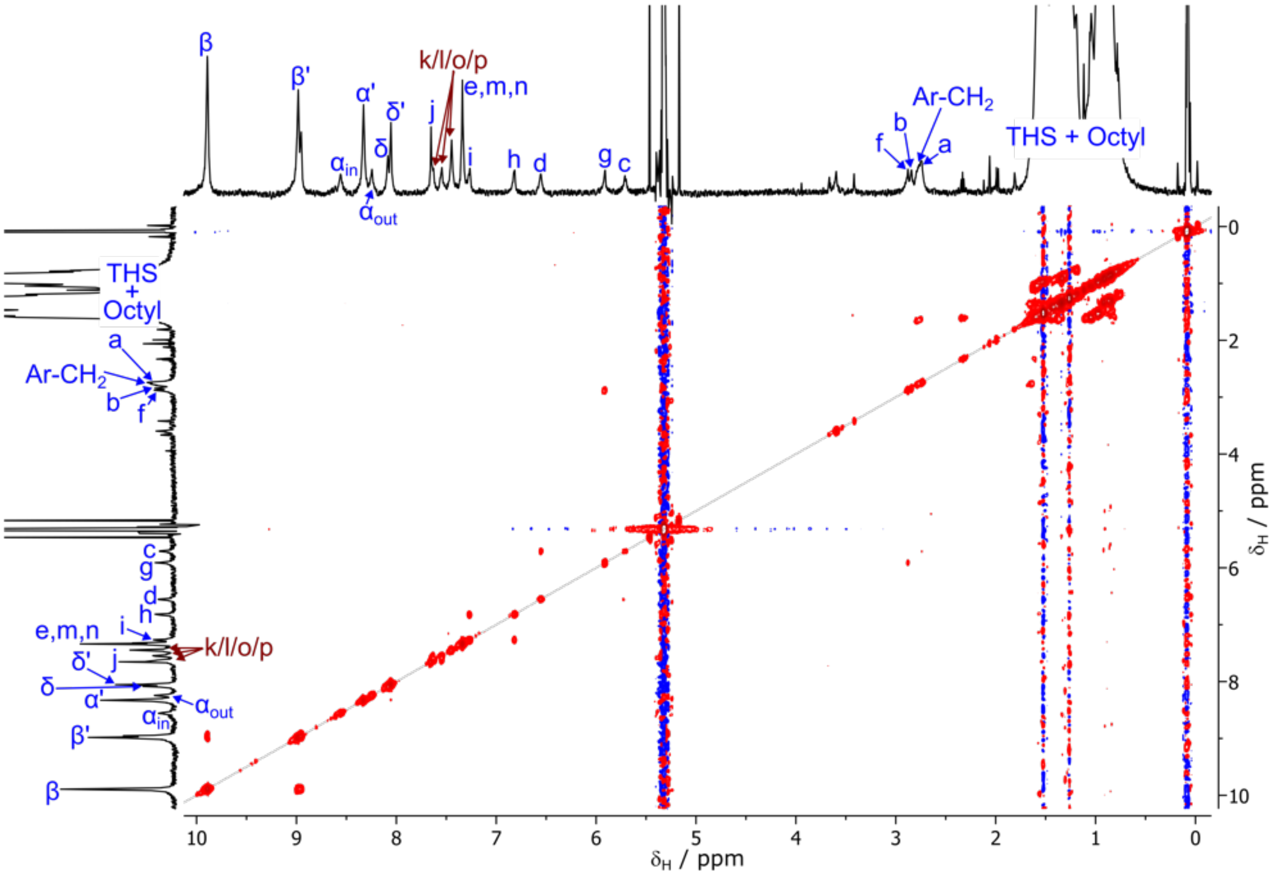

Figure S230. ^{1}H-^{1}H COSY spectrum of ***c*-P18THS·T18A** (600 MHz, CD_2Cl_2, 298 K). * = $CHDCl_2$; w = water.

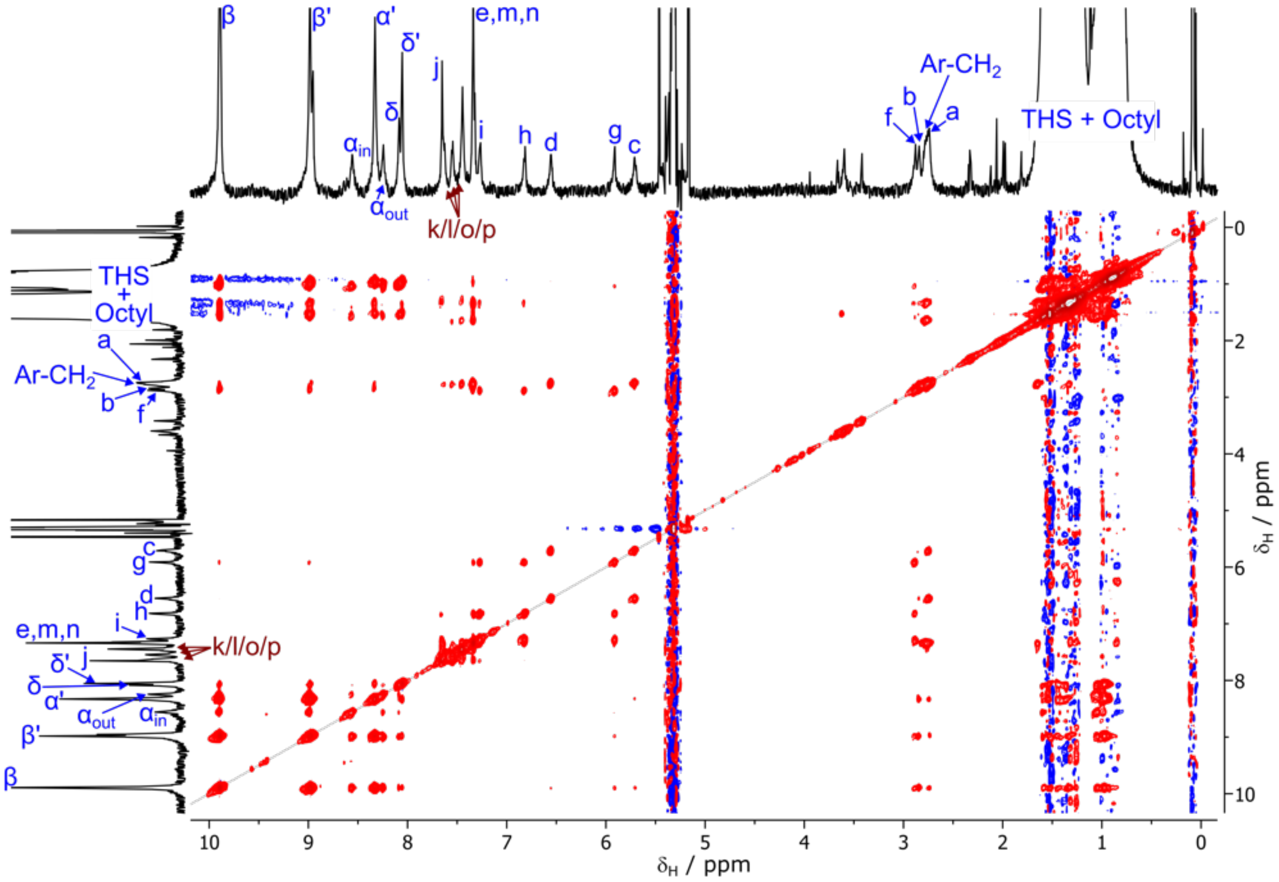

Figure S231. ^{1}H-^{1}H NOESY spectrum of ***c*-P18$_{THS}$·T18$_A$** (600 MHz, CD_2Cl_2, 298 K). * = $CHDCl_2$; w = water.

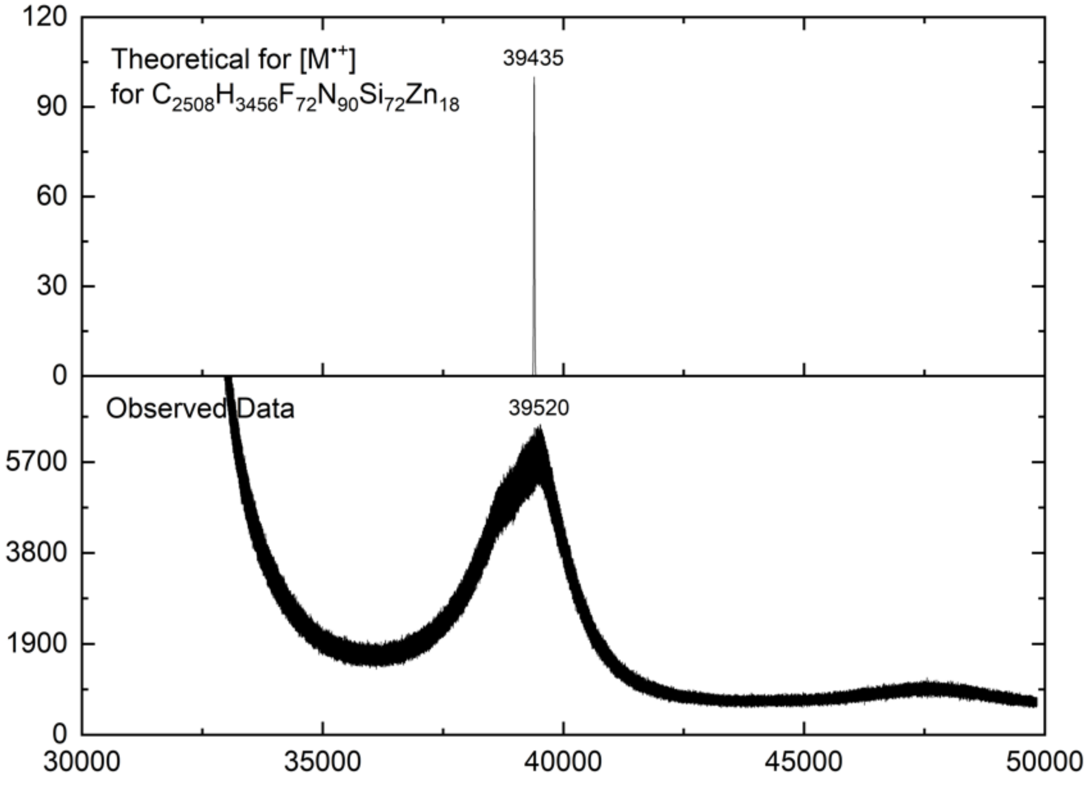

Figure S232. MALDI mass spectrum of ***c*-P18$_{THS}$·T18$_A$** (dithranol matrix)

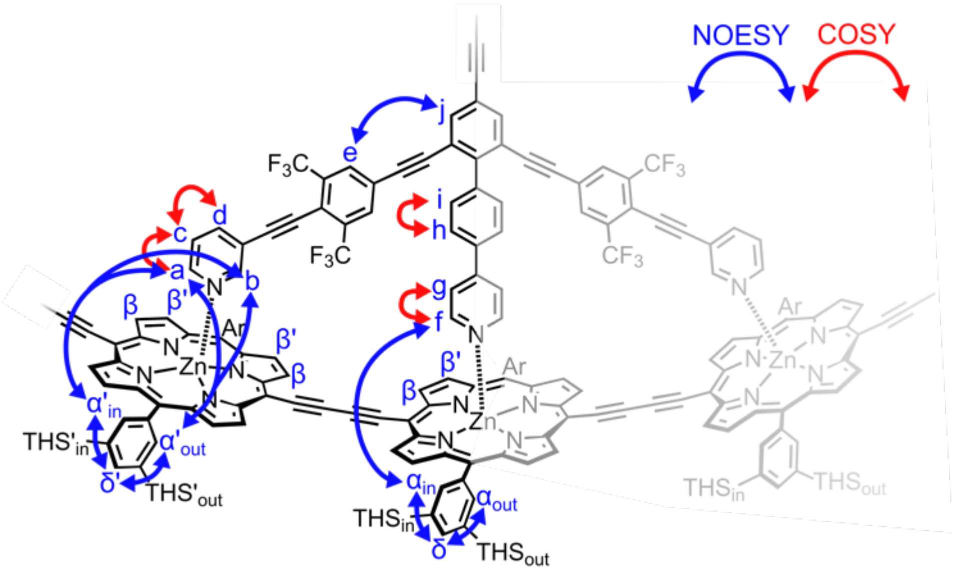

	a	b	c	d	e	f	g	h	i	j	k	l	m	n	o	p	αout	αin	α'out	α'in	δ	δ'	β	β'	Ar-CH2	Ar-CH2-CH2
a			w																							
b																										
c	m	w		s																						
d	m	w	m																							
e																										
f					w		m																			
g						m																				
h					m	m	m		s																	
i						w	m	s																		
j					w			w																		
k												m														
l											m															
m																										
n																										
o																										
p																										
αout																										
αin						w																				
α'out	w	m																								
α'in	w	m																								
δ																	w	w								
δ'																			m	m						
β	m	m				w																		s		
β'	m	m				w																				
Ar-CH2													s	s												m
Ar-CH2-CH2													m	m												

Table S5. Summary of COSY (above diagonal) and NOESY (below diagonal) correlations in ***c*-P18$_{THS}$·T18$_{A}$** complex. vw; very weak; w: weak; mw: medium weak; m: medium; s: strong.

Explanation of Assignment

- β protons at characteristic chemical shift and correct integration. β vs β' assigned as proton closest to acetylene always has highest ppm. β and β' assigned.
- Strongest NOESY to β protons come from three peaks 8.56, 8.33 and 8.24 ppm. Tentatively assign as *ortho* protons.
- Peaks at 2.88, 2.84 and 2.74 also have strong NOESY to β and β'. As in the OOct complex, expect these to be a, b and f. Assigning a, b, c, d, f, g, h and i in the same order as in the OOct complex and analyzing the COSY correlations confirms they are in the same order, so can confidently be assigned.
- Back to the tentatively assigned *ortho* protons. Peak at 8.33 has NOESY to both a and b – not f. Peaks at 8.56 has NOESY to f, peak to 8.24 shows no NOESY correlations. Assign peak at 8.33 to *ortho*' (α'_{in} + α'_{out}), peak at 8.56 to α_{in} and peak at 8.24 to α_{out}.
- Can then assign *para* protons based on NOESY to *ortho*. Therefore 8.08 (NOESY to α_{in} + α_{out}) is δ, and 8.05 (NOESY to α'_{in} + α'_{out}) is δ'.
- Singlets at 7.65 and 7.34 are likely to be e and j. HOESY confirms 7.34 is e, and weak NOESY between peaks confirms 7.65 is j.
- After all previous assignments only these protons remain and can only be at the following ppm: 7.64 (12H+12H_j), 7.55 (12H), 7.45 (24H), 7.36-7.32 (12H+24H_e)
- Medium COSY between 7.64 and 7.55 (must be either k, l or o,p), no other COSY correlations, which means the other pair must be at 7.45 (not enough protons at 7.36-7.32). Assign 7.36-7.32 as m+n.
- k, l, o, p cannot be definitively assigned

c-P18$_{\mathrm{THS}}$·T18$_{\mathrm{B}}$

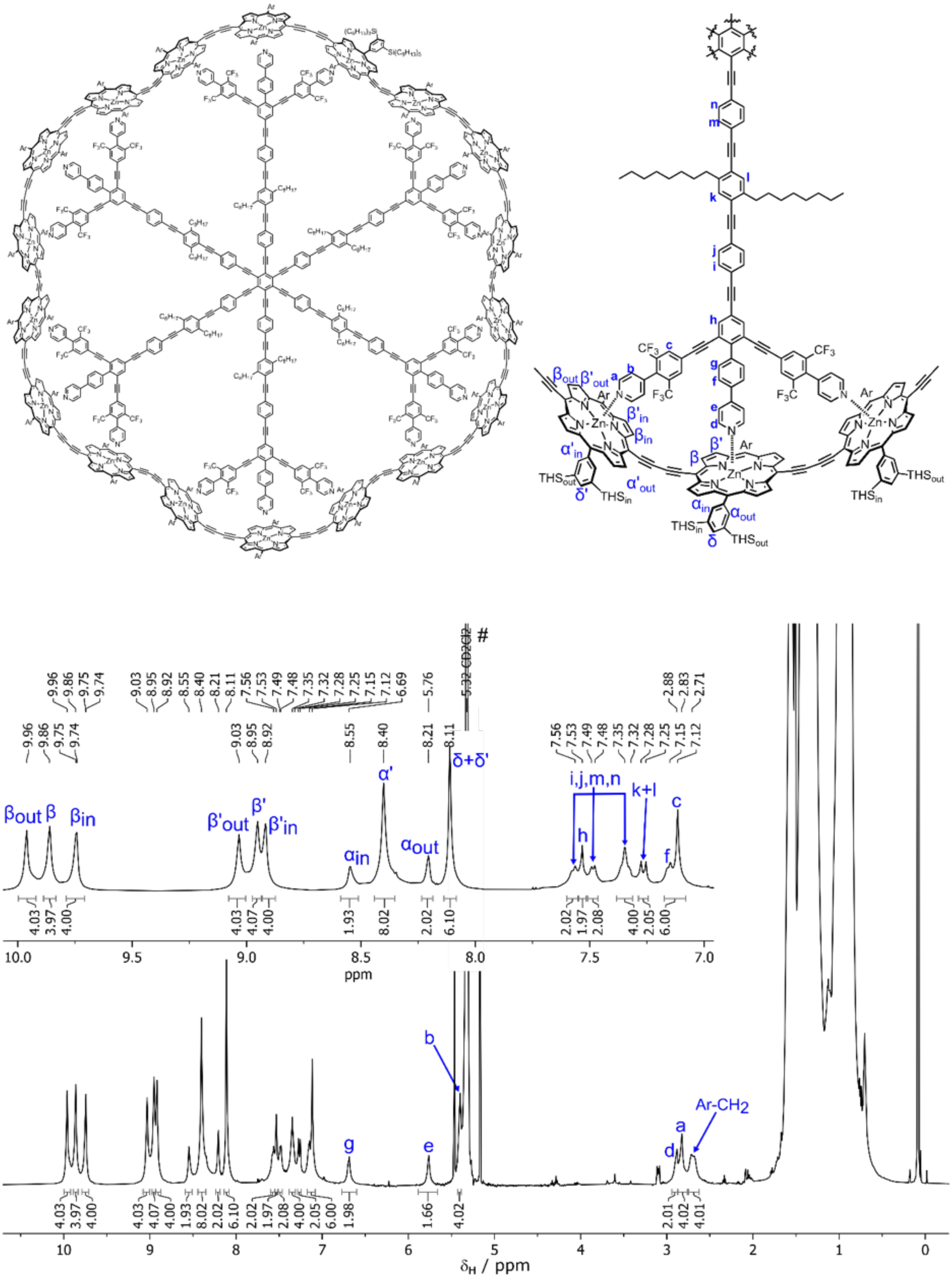

Figure S233. ^{1}H NMR spectrum of ***c*-P18$_{\mathrm{THS}}$·T18$_{\mathrm{B}}$** (600 MHz, CD_2Cl_2, 298 K). # = $CHDCl_2$.

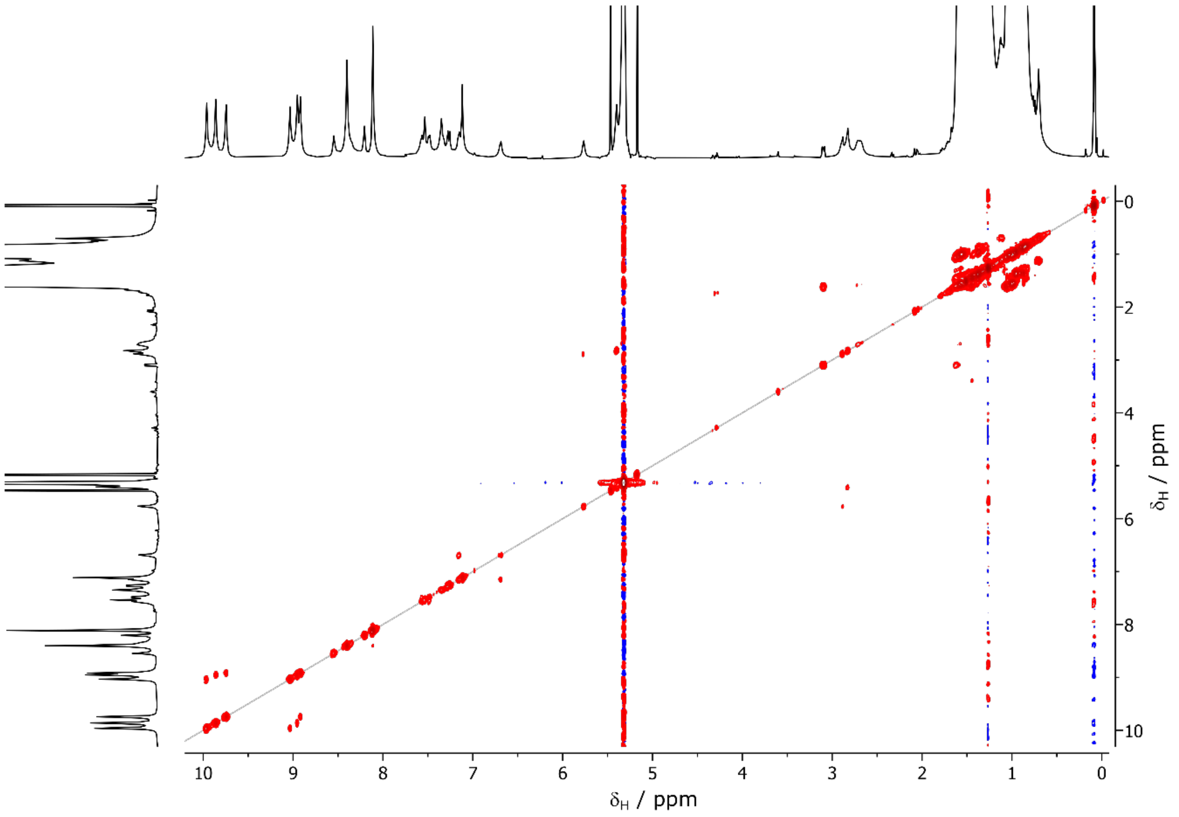

Figure S234. ^{1}H-^{1}H COSY spectrum of ***c*-P18**$_{\mathbf{THS}}$**·T18**$_{\mathbf{B}}$ (600 MHz, CD_2Cl_2, 298 K). # = $CHDCl_2$.

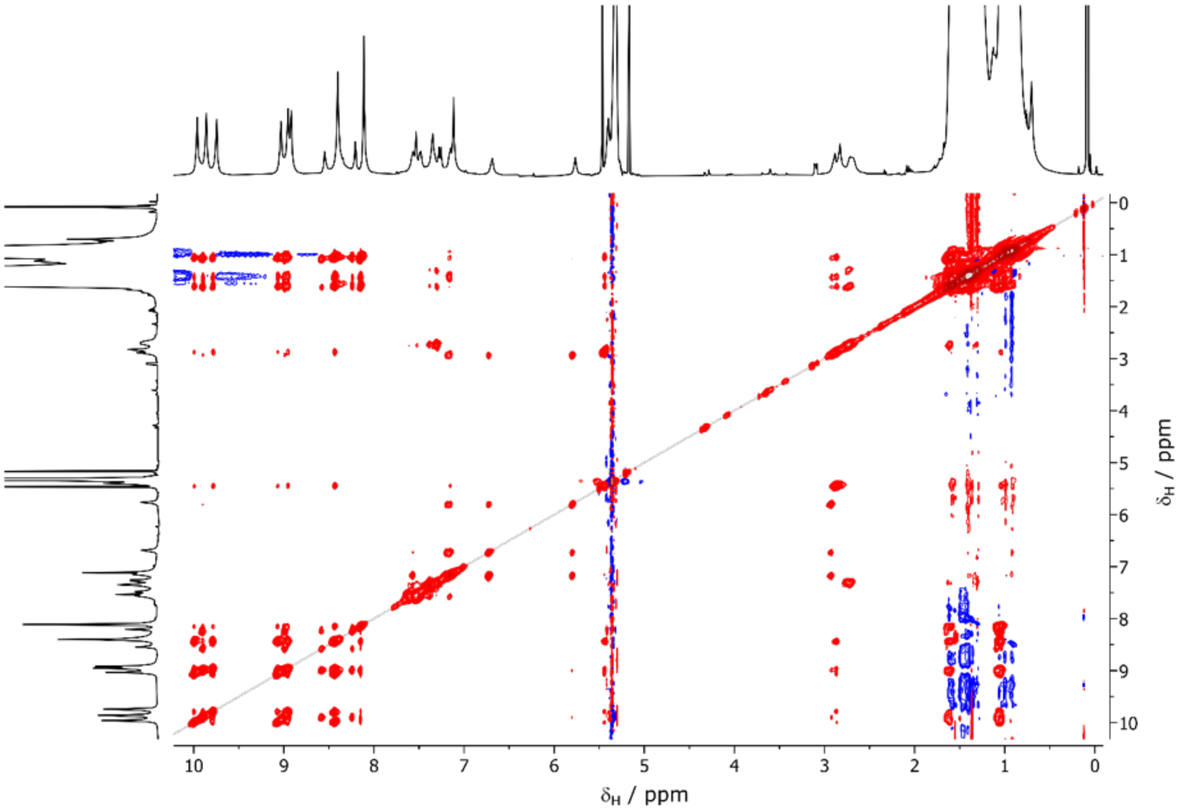

Figure S235. ^{1}H-^{1}H NOESY spectrum of ***c*-P18**$_{\mathbf{THS}}$**·T18**$_{\mathbf{B}}$ (600 MHz, CD_2Cl_2, 298 K). # = $CHDCl_2$.

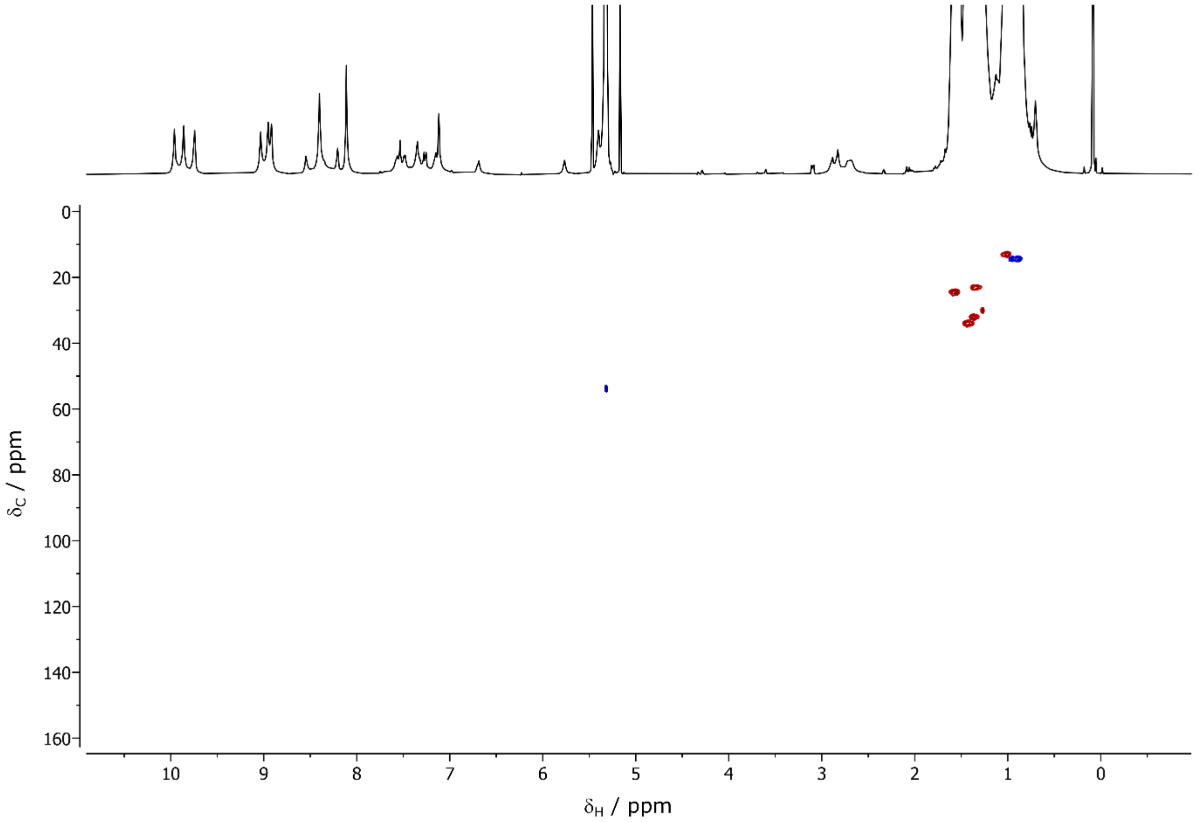

Figure S236. ^{1}H-^{13}C HSQC spectrum of ***c*-P18**$_{\mathbf{THS}}$**·T18**$_{\mathbf{B}}$ (600 MHz, CD_2Cl_2, 298 K). # = $CHDCl_2$.

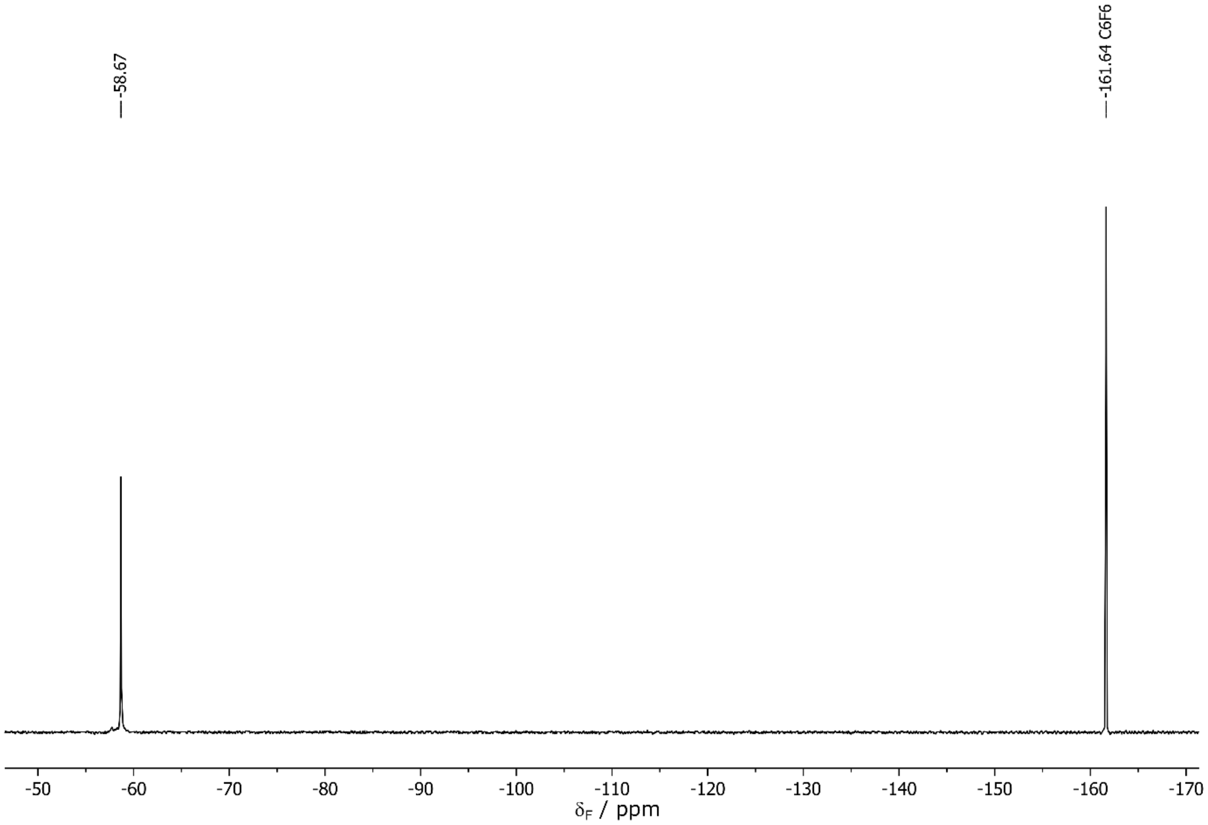

Figure S237. ^{19}F NMR spectrum of ***c*-P18**$_{\mathbf{THS}}$**·T18**$_{\mathbf{B}}$ (471 MHz, $CDCl_3$, 298 K). Referenced against C_6F_6 ($\delta_F = -161.64$) as an internal standard.

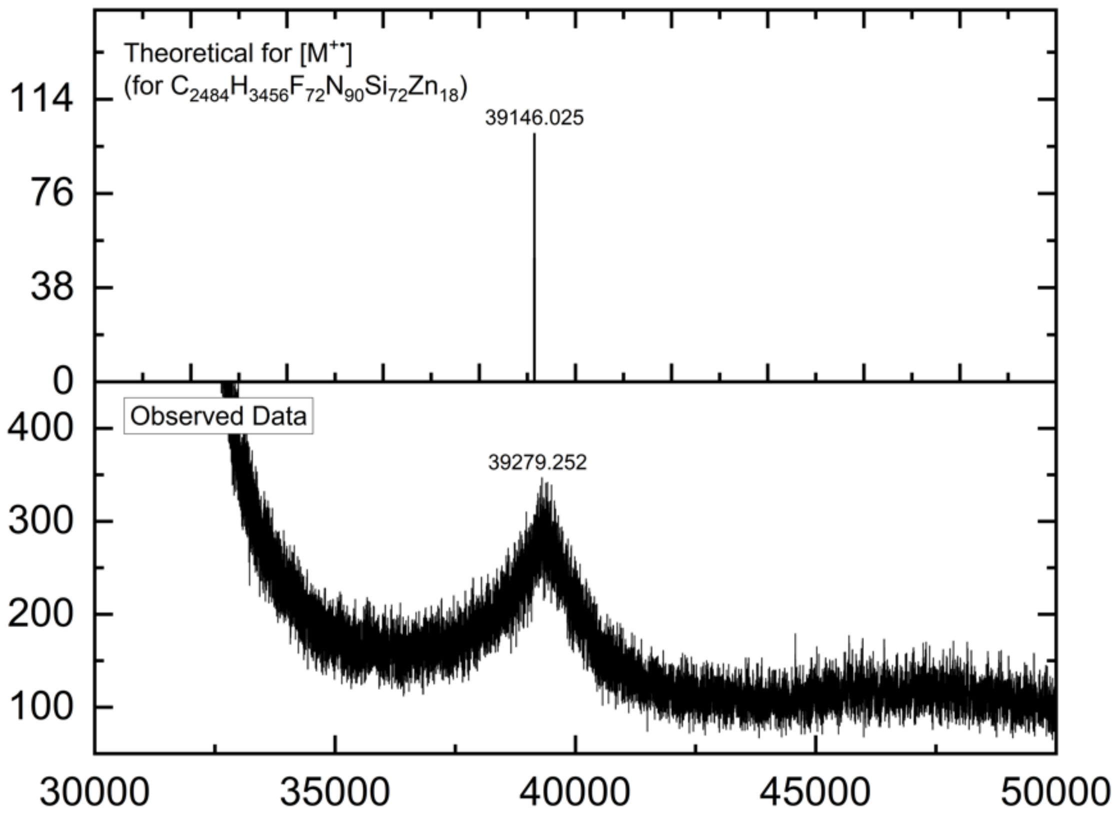

Figure S238. MALDI mass spectrum of ***c*-P18$_{THS}$·T18$_B$** (dithranol matrix)

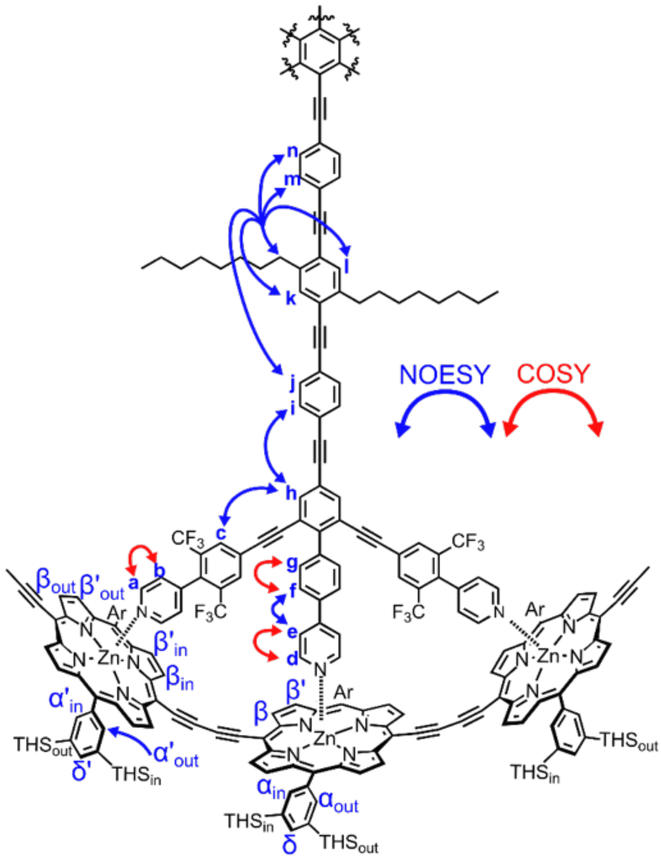

	a	b	c	d	e	f	g	h	i	j	k	l	m	n	o	p	α_{out}	α_{in}	α’	δ	δ’	β	β_{in}	β_{out}	β'	β'_{in}	β'_{out}	$Ar\text{-}CH_2$
a		s																										
b	s																											
c																												
d					s																							
e				s																								
f				m	s		s																					
g				m	m	s																						
h																												
i																												
j																												
k																												
l																												
m																												
n																												
o																												
p																												
α_{out}																												
α_{in}				w													m											
α’	m																											
δ																												
δ’																			m									
β				w													m	m							s			
β_{in}	w																		s			m				s		
β_{out}	w																		s				m				s	
β'				w													m	m				s						
β'_{in}	w																		s				s	m				
β'_{out}	w																		s				m	s		m		
$Ar\text{-}CH_2$											s	s																

Table S6. Summary of COSY (above diagonal) and NOESY (below diagonal) correlations in ***c*-P18$_{THS}$·T18$_B$** complex. vw; very weak; w: weak; mw: medium weak; m: medium; s: strong

Explanation of Assignment

- β protons at characteristic chemical shift and correct integration. Groups of β vs β' assigned as proton closest to acetylene always has highest chemical shift (ppm of β > ppm of β')
- In the β group, the NOESY pattern can be used to assign as β_{in}, β and β_{out}. (β_{in} couples to both β and β_{out}, β and β_{out} do not couple).
- COSY between β and β' protons can be used to assign β' protons.
- Of peaks in 8.6 – 8.0 ppm region, singlet at 8.1 has very weak NOESY to β and β' protons. Remaining peaks in this region have medium NOESY to β and β' protons. Singlet at 8.1 ppm is δ + δ'. Peaks in 8.6 – 8.16 region are α + α'.
- NOESY between β_{in}, β_{in}', β_{out}, β_{out}' and singlet at 8.4 ppm assigns this as α'.
- Singlets at 8.54 and 8.20 are therefore α_{in} and α_{out}. Singlet at 8.54 has stronger NOESY to H_d (assigned later) therefore is α_{in}. Singlet at 8.20 is α_{out}.
- Peaks in 2.96 – 2.61 region are expected to be H_a, H_d and Ar-CH_2 (Similar to previous complexes). Can assign these, along with H_b, H_e, H_g and H_f, based on relative integrations and COESY/NOESY correlations.
- Singlets at 7.53 and 7.12 must be H_c and H_h, can assign based on relative integrations.
- Pair of singlets at 7.27 and 7.25 are the only aromatic protons that have NOESY to Ar-CH_2 therefore must be H_k and H_l. These cannot be distinguished based on the data we have.
- The remaining multiplets (7.57, 7.48 and 7.34) are from H_i, H_j, H_m and H_n, but these cannot be distinguished based on the data here.

12. References

1) Peeks, M. D.; Claridge, T. D. W.; Anderson, H. L. Aromatic and antiaromatic ring currents in a molecular nanoring. *Nature* **2017**, *541*, 200–203.
2) Rickhaus, M.; Jirasek, M.; Tejerina, L.; Gotfredsen, H.; Peeks, M. D.; Haver, R.; Jiang, H.-W.; Claridge, T. D. W.; Anderson, H. L. Global aromaticity at the nanoscale. *Nat. Chem.* **2020**, *12*, 236–241.
3) Kopp, S. M.; Gotfredsen, H.; Hergenhahn, J.; Rodríguez-Rubio, A.; Deng, J.-R.; Zhu, H.; Stawski, W.; Anderson, H. L. Charge delocalization and global aromaticity in a partially fused 12-porphyrin nanoring. *Chem.* **2024**, *10*, 3410–3427.
4) Dennington, R.; Keith, T. M.; Millam, J. M. GaussView, Version 6.1. *Semichem Inc.* **2016**.
5) Stewart, J. J. P. Optimization of parameters for semiempirical methods VI: more modifications to the NDDO approximations and re-optimization of parameters. *J. Molec. Modeling.* **2013**, 19, 1–32.
6) Stewart, J. J. P. MOPAC2016. *Stewart Computational Chemistry*.
7) M. J. Frisch,G. W. Trucks, H. B. Schlegel, G. E. Scuseria, M. A. Robb, J. R. Cheeseman, G. Scalmani, V. Barone, G. A. Petersson, H. Nakatsuji, X. Li, M. Caricato, A. V. Marenich, J. Bloino, B. G. Janesko, R. Gomperts, B. Mennucci, H. P. Hratchian, J. V. Ortiz, A. F. Izmaylov, J. L. Sonnenberg, D. Williams-Young, F. Ding, F. Lipparini, F. Egidi, J. Goings, B. Peng, A. Petrone, T. Henderson, D. Ranasinghe, V. G. Zakrzewski, J. Gao, N. Rega, G. Zheng, W. Liang, M. Hada, M. Ehara, K. Toyota, R. Fukuda, J. Hasegawa, M. Ishida, T. Nakajima, Y. Honda, O. Kitao, H. Nakai, T. Vreven, K. Throssell, J. A. Montgomery, Jr., J. E. Peralta, F. Ogliaro, M. J. Bearpark, J. J. Heyd, E. N. Brothers, K. N. Kudin, V. N. Staroverov, T. A. Keith, R. Kobayashi, J. Normand, K. Raghavachari, A. P. Rendell, J. C. Burant, S. S. Iyengar, J. Tomasi, M. Cossi, J. M. Millam, M. Klene, C. Adamo, R. Cammi, J. W. Ochterski, R. L. Martin, K. Morokuma, O. Farkas, J. B. Foresman and D. J. Fox. Gaussian 16, Rev. C.01/C.02. *Gaussian, Inc*. **2016**.
8) Becke, A. D. Density-functional thermochemistry. III. The role of exact exchange. *J. Chem. Phys.* **1993**, *98*, 5648−5652.
9) Hehre, W. J.; Ditchfeld, R.; Pople, J. A. Self-consistent molecular orbital methods. XII. Further extensions of Gaussian-type basis sets for use in molecular orbital studies of organic molecules. *J. Chem. Phys.* **1972**, *56*, 2257–2261
10) Van Raden, J. M.; Deng, J.-R.; Gotfredsen, H.; Hergenhahn, J.; Clarke, M.; Edmondson, M.; Hart, J.; O'Shea, J. N.; Duarte, F.; Saywell, A.; Anderson, H. L. Template-directed synthesis of strained *meso-meso*-linked porphyrin nanorings. *Angew. Chem. Int. Ed.*, **2024**, *63*, e202400103.
11) Abraham, M. J.; Murtola, T.; Schulz, R.; Páll, S.; Smith, J. C.; Hess, B.; Lindahl, E. GROMACS: High performance molecular simulations through multi-level parallelism from laptops to supercomputers. *SoftwareX* **2015**, *1–2*, 19–25.
12) Wang, J.; Wolf, R. M.; Caldwell, J. W.; Kollman, P. A.; Case D. A. Development and testing of a general amber force field. *J. Comput. Chem.* **2004**, *2*, 1157–1174.
13) Gotfredsen, H.; Deng, J.-R. Van Raden, J. M. Righetto, M.; Hergenhahn, J.; Clarke, M.; Bellamy-Carter, A.; Hart, J.; O'Shea, J.; Claridge, T. D. W.; Duarte, F.; Saywell, A.; Herz, L. M.; Anderson, H. L. Bending a photonic wire into a ring. *Nat. Chem.* **2022**, *14*, 1436–1442.
14) Gotfredsen, H.; Hergenhahn, J.; Duarte, F.; Claridge, T. D. W.; Anderson, H. L. Bimolecular sandwich aggregates of porphyrin nanorings. *J. Am. Chem. Soc.* **2024**, *146*, 25232−25244.
15) Bussi, G.; Donadio, D.; Parrinello, M. Canonical sampling through velocity rescaling. *J. Chem. Phys.* **2007**, *126*, 014101.
16) Parrinello, M.; Rahman, A. Polymorphic transitions in single crystals: A new molecular dynamics method. *J. Appl. Phys.* **1981**, *52*, 7182–7190.
17) Cieplak, P.; Caldwell, J.; Kollman, P. Molecular mechanical models for organic and biological systems going beyond the atom centered two body additive approximation: aqueous solution free energies of methanol and N-methyl acetamide, nucleic acid base, and amide hydrogen bonding and chloroform/water partition coefficients of the nucleic acid bases. *J. Comput. Chem.* **2001**, *22*, 1048–1057.
18) Essmann, U.; Perera, L.; Berkowitz, M. L.; Darden, T.; Lee, H.; Pedersen, L. G. A smooth particle mesh Ewald method. *J. Chem. Phys.* **1995**, *103*, 8577–8593.

19) Hess, B.; Bekker, H.; Berendsen, H. J. C.; Fraaije, J. G. E. M. LINCS: A linear constraint solver for molecular simulations. *J. Comput. Chem.* **1997**, *18*, 1463–1472.

20) Hoffmann, M.; Kärnbratt, J.; Chang, M.-H.; Herz, L. M.; Albinsson, B.; Anderson, H. L. Enhanced π-conjugation around a porphyrin[6] nanoring. *Angew. Chem. Int. Ed.* **2008**, *47*, 4993–4996.

21) Favereau, L.; Cnossen, A.; Kelber, J. B.; Gong, J. Q.; Oetterli, R. M.; Cremers, J.; Herz, L. M.; Anderson, H. L. Six-coordinate zinc porphyrins for template-directed synthesis of spiro-fused nanorings. *J. Am. Chem. Soc.* **2015**, *137*, 14256−14259.

22) Lee, I.-Y.; Gruber, T. D.; Samuels, A.; Yun, M.; Nam, B.; Kang, M.; Crowley, K.; Winterroth, B.; Boshoff, H. I.; Barry, C. E. Structure–activity relationships of antitubercular salicylanilides consistent with disruption of the proton gradient via proton shuttling. *Bioorg. Med. Chem.* **2013**, *21*, 114–126.

23) Ozores, H. L.; Amorín, M.; Granja, J. R. Self-assembling molecular capsules based on α,γ-cyclic peptides. *J. Am. Chem. Soc.* **2017**, *139*, 776–784.

24) Mallinger, A.; Crumpler, S.; Pichowicz, M.; Waalboer, D.; Stubbs, M.; Adeniji-Popoola, O.; Wood, B.; Smith, E.; Thai, C.; Henley, A. T.; Georgi, K.; Court, W.; Hobbs, S.; Box, G.; Ortiz-Ruiz, M.-J.; Valenti, M.; De Haven Brandon, A.; TePoele, R.; Leuthner, B.; Workman, P.; Aherne, W.; Poeschke, O.; Dale, T.; Wienke, D.; Esdar, C.; Rohdich, F.; Raynaud, F.; Clarke, P. A.; Eccles, S. A.; Stieber, F.; Schiemann, K.; Blagg, J. Discovery of potent, orally bioavailable, small-molecule inhibitors of WNT signaling from a cell-based pathway screen. *J. Med. Chem.* **2015**, *58*, 1717–1735.

25) Hirano, Y.; Kojima, S.; Yamamoto, Y. A hypervalent pentacoordinate boron compound with an N−B−N three-center four-electron bond. *J. Org. Chem.* **2011**, *76*, 2123–2131.

26) Pelter, A.; Jenkins, I.; Jones, D. E. The preparations and some properties of mixed aryl-thienyl oligomers and polymers. *Tetrahedron* **1997**, *53*, 10357–10400.

27) Li, G.; Wang, X.; Li, J.; Zhao, X.; Wang, F. Rapid solution and solid phase synthesis of monodisperse oligo[(1,4-phenyleneethynylene)-alt-(2,5-thiopheneethynylene)]s. *Tetrahedron* **2006**, *62*, 2576–2582.

28) Majewski, M. A.; Stawski, W.; Van Raden, J. M.; Clarke, M.; Hart, J.; O'Shea, J. N.; Saywell, A.; Anderson, H. L. Covalent template-directed synthesis of a spoked 18-porphyrin nanoring. *Angew. Chem. Int. Ed.* **2023**, *62*, e202302114.

29) Svatek, S. A.; Perdigão, L. M. A.; Stannard, A.; Wieland, M. B.; Kondratuk, D. V.; Anderson, H. L.; O'Shea, J. N.; Beton, P. H. Mechanical stiffening of porphyrin nanorings through supramolecular columnar stacking. *Nano Lett.* **2013**, *13*, 3391–3395.

30) Kopp, S. M.; Gotfredsen, H.; Deng, J.-R.; Claridge, T. D. W.; Anderson, H. L.. Global aromaticity in a partially fused 8-porphyrin nanoring. *J. Am. Chem. Soc.* **2020**, *142*, 19393–19401.

31) Jirásek, M.; Anderson, H. L.; Peeks, M. D. From macrocycles to quantum rings: Does aromaticity have a size limit? *Acc. Chem. Res.* **2021**, *54*, 3241–3251.
