# Supplementary material for: An 18-Porphyrin Nanoring at the Size Limit for Global Aromaticity
Source: J Am Chem Soc. 2025 Aug 28;147(36):32840–50. doi: 10.1021/jacs.5c09149 (PMC12426941; doi:10.1021/jacs.5c09149)
Supplement: Supplementary file 2 [file ja5c09149_si_002.zip › c-P18 ms xyz coordinates/list of files.pdf]

Calculated xyz coordinates for the paper “An 18-porphyrin nanoring at the size limit for global aromaticity”

Jake M. Holmes, Lene Gödde and Harry L. Anderson
Department of Chemistry, University of Oxford, Chemistry Research Laboratory, Oxford OX1 3TA, UK

This data set consists of xyz coordinates for an 18-porphyrin nanoring and related structures. The following files are includes:

(1) xyz coordinates of c-P18 template complexes with CF3 groups for Biot-Savart calculations
(a) T18a_c-P18.xyz
(b) T18b_c-P18.xyz

(2) xyz coordinates of template complexes
(a) T18a_c-P18_PM7.xyz
(b) T18b_c-P18_PM7.xyz
(c) T3b_l-P3_DFT.xyz
(d) T3a_lP3_DFT.xyz

(3) xyz coordinates of c-P18 B3LYP in different charge states for NICS calculations
(a) c-P18 neutral.xyz
(b) c-P18 plus2.xyz
(c) c-P18 plus4.xyz
(d) c-P18 plus6.xyz
(e) c-P18 plus8.xyz
(f) c-P18 plus10.xyz
(g) c-P18 plus12.xyz
(h) c-P18 plus14.xyz
(i) c-P18 plus16.xyz
(j) c-P18 plus18.xyz
